# Impact of Genetic Variability on Physiological Responses to Caffeine in Humans: A Systematic Review

**DOI:** 10.3390/nu10101373

**Published:** 2018-09-25

**Authors:** Jacob L. Fulton, Petros C. Dinas, Andres E. Carrillo, Jason R. Edsall, Emily J. Ryan, Edward J. Ryan

**Affiliations:** 1 Department of Movement Science, Chatham University, Pittsburgh, PA 15232, USA; jacob.fulton@chatham.edu (J.L.F.); ACarrillo@chatham.edu (A.E.C.); J.Edsall@chatham.edu (J.R.E.); 2 FAME Laboratory, Department of Exercise Science, University of Thessaly, GR42100 Trikala, Greece; petros.cd@gmail.com; 3 Department of Exercise Physiology, West Virginia University School of Medicine, West Virginia University, Morganton, WV 26506, USA; ejfickes@hsc.wvu.edu

**Keywords:** polymorphism, anxiety, ergogenic, adenosine receptor, cytochrome P450, caffeine, pharmacogenomics

## Abstract

Emerging research has demonstrated that genetic variation may impact physiological responses to caffeine consumption. The purpose of the present review was to systematically recognize how select single nucleotide polymorphisms (SNPs) impact habitual use of caffeine as well as the ergogenic and anxiogenic consequences of caffeine. Two databases (PubMed and EBSCO) were independently searched using the same algorithm. Selected studies involved human participants and met at least one of the following inclusion criteria: (a) genetic analysis of individuals who habitually consume caffeine; (b) genetic analysis of individuals who underwent measurements of physical performance with the consumption of caffeine; (c) genetic analysis of individuals who underwent measurements of mood with the consumption of caffeine. We included 26 studies (10 randomized controlled trials, five controlled trials, seven cross-sectional studies, three single-group interventional studies and one case-control study). Single nucleotide polymorphisms in or near the cytochrome P450 (*CYP1A2*) and aryl hydrocarbon receptor (*AHR*) genes were consistently associated with caffeine consumption. Several studies demonstrated that the anxiogenic consequences of caffeine differed across adenosine 2a receptor (*ADORA2A*) genotypes, and the studies that investigated the effects of genetic variation on the ergogenic benefit of caffeine reported equivocal findings (*CYP1A2*) or warrant replication (*ADORA2A*).

## 1. Introduction

Caffeine (1,3,7-trimethylxanthine) is one of the most widely used drugs in the world and is available in many mediums for consumption. The pharmacokinetics and pharmacodynamics of caffeine have been well studied [1]. Caffeine metabolism occurs primarily in the liver via the cytochrome P450 system (CYP1A2) [2]. The CYP1A2 proteins are encoded by the *CYP1A2* gene, and CYP1A2 activity is induced when aromatic hydrocarbons bind the aryl hydrocarbon receptor [3]. Caffeine acts as an adenosine antagonist via competitive inhibition [4], and research in mice has demonstrated that blockade of adenosine 2a receptors (encoded via *ADORA2A* gene) may potentiate dopaminergic neurotransmission [5]. It is biologically plausible that variations in the *CYP1A2* and aryl hydrocarbon receptor (*AHR*) genes impact the metabolism of caffeine and thus subsequent physiological concentrations of caffeine achieved. Further, it can be hypothesized that variations in the *ADORA2A* gene may impact caffeine-adenosine 2a receptor binding characteristics and thus downstream dopaminergic neurotransmission. Recently, the effects of single nucleotide polymorphisms (SNPs) in the aforementioned genes on caffeine use and metabolism have been investigated [3,6,7].

With the widespread consumption of caffeine-containing beverages, the health consequences of these beverages are of particular interest to researchers. For example, the chronic consumption of coffee has been associated with cognitive performance and cardiovascular health [8,9]. The identification of predictors of habitual caffeine consumption may prove useful to epidemiologists and health professionals. To date, several SNPs, such as the *CYP1A2* (rs2472297) and *AHR* (rs4410790, rs6968554), have been implicated in habitual use [10]. Further, while caffeine is generally well tolerated, some individuals report feelings of anxiety following consumption [11]. Recent investigations have explored the effect of variations in the *ADORA2A* and *CYP1A2* genes as a potential explanation for caffeine’s anxiogenic impact in some individuals [6,11,12].

Athletes have long utilized caffeine as an ergogenic aid [13]. Research has demonstrated that 3–6 mg kg^−1^ of body mass mildly improves exercise/physical performance [14,15,16]. Nonetheless, investigators have reported equivocal findings, with some reporting interindividual variation in ergogenic responses to caffeine within their subject pools [17,18,19]. Earlier work has demonstrated that a Single Nucleotide Polymorphism (SNP) in the *CYP1A2* gene (rs762551) led to differing rates of caffeine metabolism across genotypes in smokers [3]. Recently, researchers have examined the influence of this specific SNP and select others on the ergogenic benefit of caffeine [20,21].

To our knowledge, investigators have not systematically recognized studies evaluating the effects of indexed and unknown SNPs in biologically plausible genes on physiological responses to caffeine across scholarly disciplines. Such a systematic review may provide a basis for further interdisciplinary approaches and future directions. Therefore, the purpose of the present review was to systematically investigate the impact of select SNPs on the ergogenic and anxiogenic consequences, and habitual use, of caffeine in humans.

## 2. Materials and Methods

### 2.1. Search Strategy

The Preferred Reporting Items for Systematic Review and Meta-Analyses (PRISMA) guidelines [22,23,24] were followed. Two databases [PubMed and Medline (EBSCO)] were independently searched by two investigators (J.L.F and P.C.D) up until 5 July 2018 using an appropriate algorithm (Figure S1). Any conflicts in the searching procedure were resolved through consensus, while the searching results were reviewed and sorted to identify relevant publications to the topic under review.

### 2.2. Selection Criteria

The studies included in this review involved human participants and met at least one of the following criteria: (a) genetic analysis of individuals who habitually consume caffeine; (b) genetic analysis of individuals who underwent measurements of physical performance with the consumption of caffeine; (c) genetic analysis of individuals who underwent measurements of mood with the consumption of caffeine. Included studies displayed outcomes regarding SNPs associated with habitual caffeine consumption, relationships between certain SNPs, and relationships between caffeine consumption and mood. We excluded animal studies, reviews, conference proceedings, and editorials; however, we screened the reference lists of such publications and of the retrieved articles for relevant papers. The list of the included studies (*n* = 26) is available in the data extraction table (Table 1), while the list of the excluded studies (*n* = 3512) is available in Figure S2.

### 2.3. Risk of Bias Assessment

Two reviewers (J.L.F. and P.C.D.) independently evaluated the risk of bias of the non- randomized controlled trials (RCT) via the 13-item tool developed by the Research Triangle Institute (RTI), Evidence-based Practice Center [24]. This tool has previously shown median interrater agreement of 75% [43] and 93.5% [44]. The risk of bias of the RCTs was assessed via the “Cochrane Collaboration’s tool for assessing risk of bias” [45]. Conflicts in the risk of bias assessment were resolved by two independent referee investigators (E.J.R. and A.E.C.).

### 2.4. Data Extraction and Analysis

The results of the data extraction procedure are shown in Table 1. Data extraction was performed independently by two investigators (J.L.F. and E.J.R.), and conflicts were resolved by a referee investigator (A.E.C.). For all included studies, we extracted the first authors’ name, year of publication, design of the studies, participants’ characteristics (i.e., number, sex, age, health status, and intervention) and the main and secondary outcomes, including results from statistical analyses. A qualitative synthesis of the retrieved evidence was completed thereafter.

## 3. Results

The reporting of the available information in this systematic review is shown in a PRISMA checklist in Figure S3.

### 3.1. Searching Procedure Results

The entire search yielded 3532 records. Of these, 2115 were duplicates; therefore, 1417 were initially screened to exclude reviews, conferences and editorials (*n* = 387). Consequently, 1033 records were assessed for eligibility, with 20 studies meeting the inclusion criteria. Finally, an additional six records were added manually. The searching outcome is presented in a PRISMA flow diagram (Figure S4).

### 3.2. Characteristics of the Included Studies

The characteristics and the results of the included studies are presented in Table 1. Of the included 26 studies, 10 were RCT (38%), five were controlled trials (CT) (19%), seven were cross-sectional studies (CSS) (27%), three were single-group interventional studies (13%), and one was a case–control study (CCS) (4%).

### 3.3. Risk of Bias Assessment

**Figure 1:**
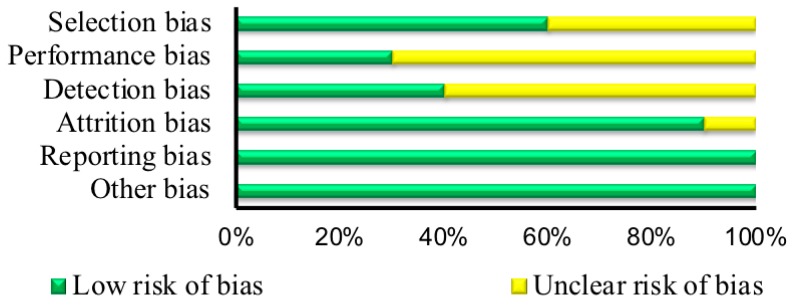
Summary of risk of bias assessment for randomized controlled trials (*n* = 10). Selection bias (random sequence generation, low risk (*n* = 2), unclear risk (*n* = 8) + allocation concealment, low risk (*n* = 4), unclear risk (*n* = 6)); Performance bias (blinding of participants and researchers, low risk (*n* = 3), unclear risk (*n* = 7)); Detection bias (blinding of outcome assessment, low risk (*n* = 4), unclear risk (*n* = 6)); Attrition bias (incomplete outcome data, low risk (*n* = 9), unclear risk (*n* = 1)); Reporting bias (selective reporting, low risk (*n* = 10)); Other bias, low risk (*n* = 10).

**Figure 2:**
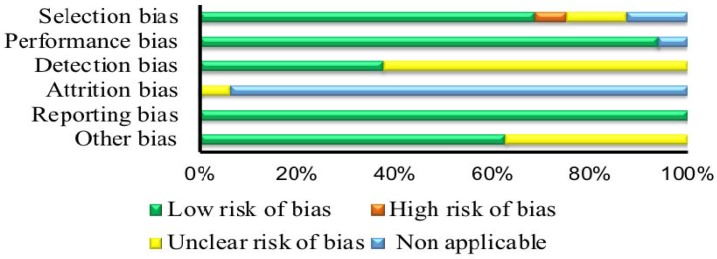
Summary of risk of bias assessment for non-randomized controlled trials. Selection bias, high risk (*n* = 1), low risk (*n* = 11), unclear risk (*n* = 2), non-applicable (*n* = 2); Performance bias, low risk (*n* = 15), non-applicable (*n* = 1); Detection bias, low risk (*n* = 6), unclear risk (*n* = 10); Attrition bias, unclear risk (*n* = 1), non-applicable (*n* = 15); Reporting bias (selective reporting, low risk (*n* = 16)); Other bias (confounding, low risk (*n* = 10), unclear risk (*n* = 6)).

The risk of bias assessment results can be found in Table 2 and Table 3, and a summary of the results are displayed in Figure 1 and Figure 2. For the RTCs, eight showed an unclear risk of bias for random sequence generation [11,12,31,36,38,39] and two showed a low risk of bias [21,33]. For allocation concealment, six studies were classified as showing an unclear risk of bias [6,11,12,20,31,36,38,39] and two showed a low risk of bias [21,33]. Seven studies demonstrated an unclear risk of bias for the blinding of participants and researchers [6,11,12,20,31,33,36], although the studies stated that the participants were “blinded”, and three displayed a low risk of bias [21,38,39]. Six studies were categorized as having an unclear risk of bias for the blinding of outcome assessment [6,12,21,31,38,39], and four were categorized as being a low risk of bias [11,20,33,36]. One out of the 10 RCTs [21] showed an unclear risk of bias for incomplete data, while the others displayed a low risk of bias. Finally, all 10 of the RCTs were categorized as having a low risk of bias for selective reporting and other bias.

Out of the 16 non-RCTs, one showed a high risk of bias [28], two were non-applicable to the category [7,35], two showed an unclear risk of bias [37,42], and the other 11 showed a low risk of bias [3,10,25,26,27,29,30,32,34,40,41] for selection bias. Of these studies, only one was non-applicable to the category [7], while the other 15 showed a low risk of bias for performance bias. For detection bias, six studies showed a low risk of bias [25,29,30,32,37,41], and the other 10 studies showed an unclear risk of bias. One of the 16 studies showed an unclear risk of bias for attrition bias [7], and the other 15 studies were non-applicable to the category. All 16 of the studies displayed a low risk of bias for selective outcome. For the confounding category, six of the 16 studies displayed an unclear risk of bias [10,27,28,34,35,37], and the remaining 10 displayed a low risk of bias.

### 3.4. Reporting of the Outcomes

#### 3.4.1. Habitual Use

Six of the included studies reported genetic variation associated with habitual use [7,10,26,27,34,37]. In a CSS, Cornelis et al. [26] examined how polymorphisms in the *CYP1A2* (rs762551) and *ADORA2A* (rs5751876) genes were associated with caffeine intake as measured via a validated food frequency questionnaire. These data demonstrated that the *CYP1A2* genotype was not associated with caffeine intake, but the *ADORA2A TT* genotype was associated with lower caffeine intake in smokers (*p* = 0.008) and nonsmokers (*p* = 0.011). Further work conducted by Cornelis et al. [27] demonstrated associations between caffeine consumption and genetic loci near the *AHR* (rs4410790, *p* = 2.4 × 10^−19^) and *CYP1A2* (rs2472304, *p* = 2.5 × 10^−7^) genes in 47,341 subjects of European descent. A CSS confirmed these associations in a distinct Costa Rican population (rs4410790, Odds Ratio = 1.41 high versus low consumers; rs2472304, Odd Ratio = 1.55 high versus low consumers) [34], and an additional CSS reported similar associations in the *AHR* gene (rs6968865, *p* range = 1.15 × 10^−1^ to 3.34 × 10^−6^ [7]. More recently, Cornelis et al. [10] demonstrated associations between caffeine consumption and several indexed SNPs (rs1260326, Log_10_ Bayes-Factor (BF) = 6.48; rs1481012, Log_10_BF = 6.08; rs7800944, Log_10_BF = 8.83; rs17685, Log_10_BF = 15.12; rs6265, Log_10_BF = 5.76; rs9902453, Log_10_BF = 6.29) in individuals of European and African-American ancestry. Further, Pirastu et al. [37] implicated a novel gene (*PDSS2*) that encodes for coenzyme Q10 in caffeine consumption.

#### 3.4.2. Anxiogenic Consequences

Eight of the included studies reported data on genetic variation and anxiety/side effects of caffeine [6,11,12,29,30,31,38,39]. Two of the included studies investigated the effects of an SNP in the *CYP1A2* (rs762551) gene on self-reported side effects of caffeine following consumption in basketball players [38,39]. The results of these studies demonstrated that self-reported feelings of anxiety were not different across genotypes. Alsene et al. [11] investigated the impact of genetic variation in the *ADORA2A* gene on anxiety following caffeine consumption in caffeine-naive subjects via a double-blind RCT. The data demonstrated that two polymorphisms (rs5751876 and rs35060421) were associated with self-reported anxiety, with the *TT* and *2592Tins/Tins* genotypes reporting higher anxiety, respectively. In an additional double blind RCT, Childs et al. [6] demonstrated that genetic variation in the *ADORA2A* (rs5751876, rs2298383, rs4822492) and dopamine receptor *DRD2* (rs1110976) genes were associated with anxiety in 102 non-to-moderate caffeine users. Further supporting the findings that individuals with the rs5751876 *TT* genotype may be prone to anxiety with caffeine, Gajewska et al. [31] and Domschke et al. [29] reported that subjects with the rs5751876 *TT* genotype exhibited impaired prepulse inhibition (female subjects) and an increased startle reflex (particularly female subgroup) with caffeine, respectively. Nonetheless, one study [12] demonstrated that the anxiogenic effect of caffeine was only apparent in subjects with the rs5751876 *TT* genotype that were caffeine naive. The authors concluded that tolerance to the anxiogenic impact of caffeine is observed when individuals habitually consume moderate to large doses [12]. Additionally, one study reported that variation in the Neuropeptide S receptor gene (rs324981) may (in conjunction with *ADORA2A* (rs5751876)) further impact the anxiogenic effects of caffeine [30].

#### 3.4.3. Ergogenic Consequences

Eight of the included studies investigated the effects of genetic variability on the ergogenic consequences of caffeine [20,21,25,32,33,36,39,46]. Five of these studies [20,25,32,33,36] assessed the impact of the *CYP1A2* (rs762551) SNP on the ergogenic consequences of caffeine using cycling time trials as a performance measure with disparate findings. Womack et al. [20] reported that male cyclists (*n* = 35) with the *CYP1A2* (rs762551) *AA* genotype demonstrated greater improvements in cycling performance (40 km time trial) versus C-allele carriers following caffeine consumption (6 mg kg^−1^ anhydrous caffeine). Similarly, Guest et al. [33] reported that male cyclists (*n* = 101) with the *CYP1A2* (rs762551) *AA* genotype demonstrated greater improvements in cycling performance (10 km time trial) relative to those with the *CYP1A2* (rs762551) *CC* genotype following caffeine treatment (2 and 4 mg kg^−1^). Equivocally, Pataky et al. [36] demonstrated that recreational cyclists (*n* = 38) with the *CYP1A2* (rs762551) *AC* genotype derived a more robust ergogenic benefit (3 km time trial) following caffeine treatment (6 mg kg^−1^ and 6 mg kg^−1^ plus caffeinated mouth rinse) relative to *CYP1A2* (rs762551) *AA* homozygotes. Algrain et al. [25] demonstrated that subject groups (*AA* vs. C-allele carriers) responded similarly to caffeine treatment (300 mg in gum vs. placebo gum) with performance measured via a 15-min performance ride. Giersch et al. [32] found that the ergogenic consequences (3 km time trial) of caffeine (6 mg kg^−1^ anhydrous caffeine) did not differ across *CYP1A2* (rs762551) genotype groups (*AA* vs. C-allele carriers). Algrain et al. [25], Pataky et al. [36], and Giersch et al. [32] all cited methodological differences as a potential explanation for the disparate findings in the literature. Studies investigating the impact of the *CYP1A2* (rs762551) SNP on the erogenicity of caffeine utilizing sports-related outcome measures (Wingate test, reaction time, basketball-specific skills) have reported that genotype groups responded equally to caffeine [38,39]. One of the included studies [21] examined the impact of the adenosine receptor *ADORA2A* (rs5751876) SNP on ergogenic responses to caffeine in females (*n* = 12) with performance assessed via a 10-min cycling time trial. These data demonstrated that subjects with the *ADORA2A* (rs5751876) *TT* genotype derived a larger ergogenic benefit from caffeine relative to heterozygotes or *CC* homozygotes [21].

### 3.5. Other Outcomes

Three of the included studies investigated the effect of the *CYP1A2* (rs762551) genotype on caffeine metabolism [3,25,32]. Sachse et al. [3] reported that, among smokers (*n* = 51), subjects who possessed the *CYP1A2* (rs762551) *AA* genotype metabolized caffeine (100 mg) faster relative to C-allele carriers while utilizing a 5 h paraxanthine/caffeine ratio as the outcome measure. The authors concluded that the *CYP1A2* (rs762551) *AA* genotype may confer high inducibility and CYP1A2 activity. More recently, confirming the findings above in 20 nonsmokers, Giersch et al. [32] reported that, 1 h following the administration of 6 mg kg^−1^ anhydrous caffeine, circulating caffeine concentrations were lower in subjects possessing the *CYP1A2* (rs762551) *AA* genotype relative to C-allele carriers. Equivocally, Algrain et al. [25] reported comparable circulating caffeine concentrations over time in nonsmokers across *CYP1A2* (rs762551) genotypes. Two of the included studies investigated the effects of the *CYP1A2* (rs762551) SNP on the cardiovascular consequences of caffeine [40,41]. Thomas et al. [41] reported that changes in postexercise heart rate variability with caffeine treatment (300 mg) were similar between *CYP1A2* genotype groups (*AA* vs. C-allele carriers). Soares et al. [40] reported that *CYP1A2* heterozygotes demonstrated increases in systolic blood pressure, while subjects with the *CYP1A2 AA* genotype did not following acute caffeine ingestion (6 mg kg^−1^).

Three additional studies were included in the present review [28,35,42]. Djordjevic [28] explored the association of multiple *CYP1A2* polymorphisms with the induction of CYP1A2 enzyme activity resultant from heavy caffeine consumption. These data demonstrated that high *CYP1A2* enzyme activity was associated with heavy coffee consumption only in subjects possessing the *CYP1A2* (rs762551) *AA* genotype. In a classical twin design study, Luciano et al. [35] demonstrated that genes not typically associated with sleep disturbance were implicated in coffee-attributed insomnia. In a CCS, Urry et al. [42] demonstrated that subjects with type 2 diabetes mellitus exhibited higher estimated *CYP1A2* enzyme activity relative to control subjects.

## 4. Discussion

The purpose of the present review was to systematically recognize how select SNPs impact habitual use of caffeine as well as the ergogenic and anxiogenic consequences of caffeine. The primary findings of our work will be discussed in the subsections below.

### 4.1. Habitual Use

Genome-wide association scans have implicated several indexed SNPs in caffeine consumption. The aforementioned SNPs occur in genes known to be involved in the pharmacokinetics and pharmacodynamics of caffeine. The results of the present review indicate that SNPs in the *CYP1A2* gene and near the *AHR* gene have been consistently associated with caffeine consumption [7,10,27,34]. Further, less conclusive evidence suggests that SNPs in the *ADORA2A* gene are associated with caffeine consumption [10,26,27]. Recently, novel genes have been implicated in caffeine consumption, with authors calling for replication of the findings and postulating biological plausibility [10,37].

### 4.2. Anxiogenic Consequences

Our search provided strong evidence that SNPs in the *ADORA2A* gene (primarily rs5751876) are associated with the anxiogenic impact of caffeine [6,11,29,31]. Particularly, caffeine-naïve females possessing the *ADORA2A* (rs5751876) *TT* genotype may be especially prone to experiencing anxiety following caffeine consumption [29,31]. Interestingly, one study demonstrated that self-reported anxiety with caffeine was not apparent in subjects possessing *ADORA2A* (rs5751876) *TT* who habitually consume large to moderate doses. Thus, the available evidence suggests that habitual use may lead to tolerance to the anxiogenic consequences of caffeine regardless of select genetic variations.

### 4.3. Ergogenic Consequences

The studies included in this section of the review focused primarily on the *CYP1A2* (rs762551) SNP and reported equivocal findings. Two studies reported that the *CYP1A2* (rs762551) *AA* genotype resulted in a more robust ergogenic benefit of caffeine [20,33] relative to C-allele carriers, while one study reported opposing findings [36], and two studies reported comparable ergogenic responses across genotype groups [25,32]. Two studies reported that the *CYP1A2* (rs762551) SNP did not influence sport-specific outcomes with and without caffeine [38,39]. Our search included one pilot study that investigated the effects of the *ADORA2A* (rs5751876) SNP on the erogenicity of caffeine; Loy et al. [21] reported that females with the *ADORA2A* (rs5751876) *TT* genotype derived a larger ergogenic benefit with caffeine relative to C-allele carriers. In general, the studies included in this section cited some methodological constraints, such as low sample size and capricious outcome measures. We recommend that future studies increase sample size, utilize more standardized outcome measures, and examine a multitude of biologically plausible SNPs to further elucidate the impact genetic variation has on the ergogenic consequences of caffeine.

### 4.4. Quality of Evidence, Limitations, and Potential Biases in the Review Process

Based on the studies selected for the aim of the current systematic review, we may form adequate conclusions regarding the impact of select SNPs on the ergogenic and anxiogenic consequences and habitual use of caffeine in humans. This is because we identified enough available evidence in the area. The included RCTs displayed unclear and low risk of bias in the selection, performance, and detection biases, while they mostly displayed low risk of bias in the attrition, reporting, and other biases. Similarly, the non-RCTs displayed mostly low risk of bias in the selection, performance, reporting, and other biases, while in the attrition bias, most studies were non-applicable for the category. This indicates that both the RCTs and the non-RCTs may provide fairly good quality evidence.

Our systematic review has a number of strengths. We used the PRISMA guidelines [22,23,24] and appropriate databases and algorithms with standardized indexing terms for our searching procedure. We also used well-established tools to evaluate the included [24,43,44,45] studies. Furthermore, to minimize bias in our systematic review process, two investigators worked independently on the searching and screening procedure, data extraction, and risk of bias assessment. Finally, we have not excluded studies based on language.

A possible limitation of the current systematic review is that we avoided the use of non-peer-review data (grey literature) and conference papers to test our research question. However, the inclusion of non-peer-review data may have itself introduced bias, given that there was available peer-reviewed evidence [47]. Another possible limitation is the small number of the included studies, especially the RCTs, which indicates the need for additional research of this topic in the future.

## 5. Conclusions

In conclusion, our search has provided evidence that the *CYP1A2*, *AHR,* and *ADORA2A* genes are associated with habitual consumption, and further exploration is warranted to clarify how these genes directly or indirectly impact physiological and/or psychological mechanisms responsible for the variability in consumption of caffeine across individuals. The literature also demonstrates that gender, habitual caffeine consumption, and variability in the *ADORA2A* gene collectively influence individual susceptibility to the anxiogenic consequences of caffeine and that variability in the *CYP1A2* gene, in conjunction with environmental factors (heavy coffee drinking, smoking), impact the metabolism of caffeine. Future work is warranted to elucidate the effects of variability in the *CYP1A2* and *ADORA2A* genes on the ergogenic impact of caffeine. We recommend all future studies in this area utilize an interdisciplinary approach as the physiological consequences of caffeine in humans are likely dependent on a complex interaction of genetic, physiological, and behavioral factors.

## Figures and Tables

**Table 1:** Characteristics of the studies included in the systematic review.

First Author	Design	Participants	Main Outcome	Secondary Outcome
Algrain [25]	Controlled Trial	Male (M) = 13 Female (F) = 7	Polymorphism in *CYP1A2* gene (*AA* and C-allele carriers) did not impact ergogenic benefit of caffeine in recreational cyclists (*p* > 0.05)	
Alsene [11]	Randomized Controlled Trial	94 healthy, infrequent caffeine users	*1976T/T* and *2592Tins/Tins* genotypes report greater increase in anxiety after caffeine administration (*p* < 0.05)	
Childs [6]	Randomized Controlled Trial	102 healthy individuals (M = 51 and F = 51) who consumed less than 300 mg caffeine per week	*ADORA2A TT* genotype reported highest anxiety (VAS) (4.6 ± 1.9) and *ADORA2A CC* (−7.5 ± 3.7) reported the least anxiety after 150 mg of caffeine but was not significant when data for European-American participants were considered (*p* = 0.1); caffeine-induced anxiety was associated with dopamine receptor 2 gene (*DRD2*) polymorphism	
Cornelis [26]	Cross-sectional	*n* = 2735	*ADORA2A*, but not *CYP1A2*, genotype was associated with different amounts of caffeine intake; compared to persons consuming <100 mg caffeine/day, odds ratios for having the *ADORA2A TT* genotype were 0.74, 0.63, and 0.57 for persons consuming 100–200, 200–400, and >400 mg caffeine/day, respectively	Association more pronounced among current smokers compared to nonsmokers
Cornelis [27]	Cross-sectional	47,341 individuals of European descent	Two loci-*7p21* (*p* = 2.4 × 10^−19^), near *AHR*, and *15q24* (*p* = 5.2 × 10^−14^), near *CYP1A1* and *CYP1A2*; both candidates as *CYP1A2* caffeine metabolizers	
Cornelis [10]	Cross-sectional	Coffee consumers of European ancestry *n* = 91,462 African American ancestry *n* = 7964	Eight loci, six being novel, met genome-wide significance (log_10_Bayes factor >5.64); loci near genes potentially involved in pharmacokinetics (*ABCG2, AHR, POR,* and *CYP1A2*) and pharmacodynamics (*BDNF* and *SLC6A4*). Loci related to metabolic traits (*GCKR* and *MLXIPL*)	
Djordjevic [28]	Single-group interventional design	126 Healthy Serbians, 64 nonsmoking (from their previous study)	Inducing effect of *CYP1A2* activity with heavy coffee consumption among Serbian (*p* = 0.022) and Swedish (*p* = 0.016) participants carrying the *CYP1A2*-163 C > A polymorphism	
Domschke [29]	Controlled Trial	M = 56 and F = 54 healthy individuals	Startle magnitude highest for unpleasant pictures and lowest for pleasant pictures across *ADORA2A* genotypes (*p* < 0.001). *TT* (risk) genotype carriers had highest startle magnitude in the caffeine condition in response to unpleasant pictures, occurring mostly among females	Females of this group had higher startle magnitudes than males
Domschke [30]	Controlled Trial	58 M and 66 F healthy proband	*ADORA2A TT* risk genotype carriers had significantly increased startle magnitude in response to neutral stimuli (*p* = 0.02) and a significant decrease in startle magnitude in response to unpleasant stimuli (*p* = 0.02) in caffeine compared to placebo condition; no change in *AA*/*AT* nonrisk genotype	
Gajewska [31]	Randomized Controlled Trial	57 M and 57 F healthy individuals controlled for anxiety sensitivity	Prepulse inhibition was influenced by genetics (*ADORA2A 1976C/T*); impaired prepulse facilitation in anxiety sensitive *ADORA2A TT* group in response to caffeine compared to placebo (t(56) =2.16, *p* = 0.04)	
Giersch [32]	Controlled Trial	20 male subjects between age of 18–45 years	*CYP1A2* C-allele carriers had higher serum caffeine one hour after caffeine ingestion (C-allele carriers = 14.2 ± 1.8 ppm, *AA* homozygotes = 11.7 ± 1.7 ppm, *p* = 0.001). No difference between genotypes in caffeine metabolites (*p* > 0.05). Main effect of caffeine on performance (*p* = 0.03); no caffeine by genotype interaction (*p* > 0.05)	
Guest [33]	Randomized Controlled Trial	In *CYP1A2 AA* genotype, cycling time decreased by 4.8% (*p* = 0.0005) and 6.8% (*p* < 0.0001) with 2 and 4 mg kg^−1^ caffeine consumption, respectively. (2 and 4 mg kg^−1^); in *CC* genotype, cycling time increased by 13.7% (*p* = 0.04) with caffeine consumption (4 mg kg^−1^), no effects were observed among *AC* genotype	Competitive male athletes *n* = 101	4 mg kg^−1^ caffeine decreased cycling time by 3% vs. placebo
Josse [34]	Cross-sectional	*n* = 1639 nonsmokers and *n* = 884 current smokers	Subjects who consumed >400 mg caffeine compared to who consumed <100 mg caffeine were more likely to be carriers of T, C, or T alleles for rs6968865, rs4410790, and rs2472297, respectively; corresponding Odds Ratios and 95% confidence intervals (CIs) were 1.41 (1.03, 1.93), 1.41 (1.04, 1.92), and 1.55 (1.01, 2.36)	
Loy [21]	Randomized Controlled Trial	Women with high self-reported caffeine sensitivity and low daily caffeine consumption, *TT n* = 6, *CT/CC n* = 6	Caffeine proved to be ergogenic for *ADORA2A TT* allele homozygotes (6.85 ± 4.41 kJ) but not ergogenic for *CT/CC* alleles (−2.70 ± 5.64 kJ) (d = −1.89)	
Luciano [35]	Cross-sectional	3808 Australian adult twin pairs (*n* = 1799 monozygous pairs and *n* = 2009 dizygous pairs)	Genes not typically associated with sleep disturbance were implicated in coffee-attributed insomnia	
McMahon [7]	Cross-sectional	4460–7520 women	Caffeine consumption was associated with *CYP1A1* (Betas = 8.7 to 21.4, *p*-values = 1.59 × 10^−3^ to 3.33 × 10^−10^) and *AHR* (Betas = 4.0 to 14.6, *p*-values = 1.1510^−1^ to 3.34 × 10^−6^) genotypes; association not strengthened with combined allelic score (1.28% of phenotypic variance)	
Pataky [36]	Randomized Controlled Trial	25 M and 13 F recreational cyclists from James Madison University	*CYP1A2* AC heterozygotes experienced greater power output (6%) with caffeine ingestion. Caffeine ingestion favored *AC* heterozygotes compared to *AA* homozygotes when performance gains were compared to placebo (5.1 ± 6.1%, *p* = 0.12)	
Pirastu [37]	Cross-sectional	370 individuals from Puglia, Italy and 843 individuals from Friuli Venezia Region, Italy	*PDSS2* gene shown in sample was linked to negative regulation of the expression of caffeine metabolism genes in several tissues (e.g., subcutaneous adipose tissue −0.27, skeletal muscle −0.52)	
Puente [38]	Randomized Controlled Trial	10 men and 9 women elite basketball players	*CYP1A2* genotype (rs762551) *AA* improved Abalakov jump height (*p* = 0.03) with caffeine consumption, while C-allele carriers remained unchanged (*p* = 0.33); Sprint was not improved in either genotype with caffeine, while number of body impacts increased in both *AA* (4.1 ± 5.3%; *p* = 0.02) and C-allele carriers (3.3 ± 3.2%; *p* = 0.01)	
Rogers [12]	Randomized Controlled Trial	162 non/low and 217 medium/high caffeine consumers	*ADORA2A* (rs5751876) *TT* genotype showed largest increase in anxiety after caffeine (mean ± standard error ) for caffeine = 1.65 ± 0.15 and for placebo = 0.95 ± 0.17, *p* < 0.01)	
Sachse [3]	Single-group interventional design	185 healthy Caucasian nonsmokers and 51 smokers	Among smokers (*n* = 51), subjects who possessed the *CYP1A2* (rs762551) *AA* genotype metabolized caffeine (100 mg) faster 1.37 (*AC* –0.88; and *CC* – 0.82) relative to C-allele carriers while utilizing a 5 h paraxanthine/caffeine ratio as the outcome measure (*p* = 0.008)	
Salinero [39]	Randomized Controlled Trial	21 healthy active participants	Caffeine ingestion increased peak and mean power in both *AA* and C-allele carriers of the *CYP1A2* gene (*p* > 0.05); no difference in Wingate test performance between *AA* and C-allele carriers (*p* > 0.05)	31.3% of C-allele carriers reported increased nervousness after caffeine ingestion
Soares [40]	Single-group interventional design	37 individuals between ages of 19–50	Systolic blood pressure (BP) increased with caffeine ingestion only among individuals with *CYP1A2 AA* genotype (*p* < 0.05); both *CYP1A2 AA* and *AC* had high diastolic BP after caffeine ingestion (*p* < 0.05); physical activity only modulated the BP responses to acute caffeine ingestion in *AC* individuals	
Thomas [41]	Controlled Trial	*CYP1A2 AA* (F = 4 and M = 7), C-allele carriers (F= 3 and M = 6)	No difference in heart rate variability between CYP1A2*1F polymorphisms (i.e., *AA* and C-allele carriers) measured at baseline and postexercise (*p* > 0.05)	
Urry [42]	Case–Control Study	57 subjects with type 2 diabetes (T2D) and 146 non-T2D	CYP1A2 enzyme activity was significantly higher in T2D compared to control group (*p* = 0.004)	
Womack [20]	Randomized Controlled Trial	Trained male cyclists *n* = 35	Caffeine supplementation reduced 40 km time greater in *CYP1A2 AA* homozygotes (4.9%) than in C-allele carriers (1.8%) (*p* < 0.05)	

**Table 2:** Risk of bias assessment using the Cochrane Collaboration’s Tool.

First Author	Random Sequence Generation	Allocation Concealment	Blinding of Participants and Researchers	Blinding of Outcome Assessment	Incomplete Outcome Data	Selective Reporting	Other Bias
Randomized Controlled Trials (RCTs)							
Gajewska [31]	**?**	**?**	**?**	**?**	**+**	**+**	**+**
Alsene [11]	**?**	**?**	**?**	**+**	**+**	**+**	**+**
Pataky [36]	**?**	**?**	**?**	**+**	**+**	**+**	**+**
Rogers [12]	**?**	**?**	**?**	**?**	**+**	**+**	**+**
Puente [38]	**?**	**+**	**+**	**?**	**+**	**+**	**+**
Guest [33]	**+**	**+**	**?**	**+**	**+**	**+**	**+**
Salinero [39]	**?**	**+**	**+**	**?**	**+**	**+**	**+**
Loy [21]	**+**	**?**	**+**	**?**	**?**	**+**	**+**
Womack [20]	**?**	**+**	**?**	**+**	**+**	**+**	**+**
Childs [6]	**?**	**?**	**?**	**?**	**+**	**+**	**+**

Key: **+**: Low risk of bias (green); **?**: Unclear risk of bias (yellow). RCTs: randomized controlled trials.

**Table 3:** Risk of bias assessment using the Research Triangle Institute (RTI) Item Bank.

First Author	Selection Bias	Performance Bias	Detection Bias	Attrition Bias	Selective Outcome	Confounding
Non-RCT						
Djordjevic [28]	**-**	**+**	**?**	**o**	**+**	**?**
McMahon [7]	**o**	**o**	**?**	**?**	**+**	**+**
Soares [40]	**+**	**+**	**?**	**o**	**+**	**+**
Giersch [32]	**+**	**+**	**+**	**o**	**+**	**+**
Domschke [30]	**+**	**+**	**+**	**o**	**+**	**+**
Sachse [3]	**+**	**+**	**?**	**o**	**+**	**+**
Urry [42]	**?**	**+**	**?**	**o**	**+**	**+**
Pirastu [37]	**?**	**+**	**+**	**o**	**+**	**?**
Josse [34]	**+**	**+**	**?**	**o**	**+**	**?**
Cornelis [27]	**+**	**+**	**?**	**o**	**+**	**?**
Domschke [29]	**+**	**+**	**+**	**o**	**+**	**+**
Cornelis [10]	**+**	**+**	**?**	**o**	**+**	**?**
Thomas [41]	**+**	**+**	**+**	**o**	**+**	**+**
Algrain [25]	**+**	**+**	**+**	**o**	**+**	**+**
Cornelis [26]	**+**	**+**	**?**	**o**	**+**	**+**
Luciano [35]	**o**	**+**	**?**	**o**	**+**	**?**

**+**: Low risk of bias (green); **-**: High risk of bias (red); **?**: Unclear risk of bias (yellow); **o**: Non-applicable (blue).

## Supplementary Materials

The following are available online at http://www.mdpi.com/2072-6643/10/10/1373/s1, Figure S1: Algorithm, Figure S2: Excluded Studies; Figure S3: PRISMA Checklist; Figure S4: PRISMA Flowchart.
