# Supplementary material for: Impact of Genetic Variability on Physiological Responses to Caffeine in Humans: A Systematic Review
Source: Nutrients. 2018 Sep 25;10(10):1373. doi: 10.3390/nu10101373 (PMC6212886; doi:10.3390/nu10101373)
Supplement: Supplementary file 1 [file nutrients-10-01373-s001.zip › FigureS1 Algorithm.pdf]

Search	Query
#64	Search (#60 NOT #63)
#63	Search (#61 NOT #62)
#62	Search humans[MeSH Terms]
#61	Search animals[MeSH Terms]
#60	Search (#16 AND #35 AND #59)
#59	Search (#36 OR #37 OR #38 OR #39 OR #40 OR #41 OR #42 OR #43 OR #44 OR #45 OR #46 OR #47 OR #48 OR #49 OR #50 OR #51 OR #52 OR #53 OR #54 OR #55 OR #56 OR #57 OR #58)
#58	Search anxiety perseverance
#57	Search anxiety sensitivity[Title/Abstract]
#56	Search anxiety[Title/Abstract]
#55	Search anxiety-like behavior[Title/Abstract]
#54	Search anxiety-related behavior[Title/Abstract]
#53	Search habitual caffeine users[Title/Abstract]
#52	Search habitual caffeine intake[Title/Abstract]
#51	Search habitual consumption[Title/Abstract]
#50	Search habitual use[Title/Abstract]
#49	Search physical activity[Title/Abstract]
#48	Search strength[Title/Abstract]
#47	Search muscle hypertrophy[Title/Abstract]
#46	Search VO2peak[Title/Abstract]
#45	Search VO2max[Title/Abstract]
#44	Search aerobic performance[Title/Abstract]
#43	Search motor activity performance[Title/Abstract]
#42	Search sport performance[Title/Abstract]
#41	Search training performance[Title/Abstract]
#40	Search resistance training performance[Title/Abstract]
#39	Search mobility performance[Title/Abstract]
#38	Search cycling performance[Title/Abstract]
#37	Search running performance[Title/Abstract]
#36	Search exercise performance[Title/Abstract]
#35	Search (#17 OR #18 OR #19 OR #20 OR #21 OR #22 OR #23 OR #24 OR #25 OR #26 OR #27 OR #28 OR #29 OR #30 OR #31 OR #32 OR #33 OR #34)
#34	Search AHR[Title/Abstract]
#33	Search aryl hydrocarbon receptor[Title/Abstract]
#32	Search BDNF[Title/Abstract]
#31	Search brain derived neurotrophic factor[Title/Abstract]
#30	Search ADORA1[Title/Abstract]
#29	Search Adenosine A1 Receptor[Title/Abstract]
#28	Search ADORA2A[Title/Abstract]

#27	Search Adenosine A2a Receptor[Title/Abstract]
#26	Search CYP1A1[Title/Abstract]
#25	Search Cytochrome P450 1A1[Title/Abstract]
#24	Search CYP1A2[Title/Abstract]
#23	Search Cytochrome P450 1A2[Title/Abstract]
#22	Search genotype[Title/Abstract]
#21	Search genome[Title/Abstract]
#20	Search single nucleotide polymorphism[Title/Abstract]
#19	Search genetic polymorphism[Title/Abstract]
#18	Search genotype variations[Title/Abstract]
#17	Search SNP's[Title/Abstract]
#16	Search (#1 OR #2 OR #3 OR #4 OR #5 OR #6 OR #7 OR #8 OR #9 OR #10 OR #11 OR #12 OR #13 OR #14 OR #15)
#15	Search caffeine mouth rinse[Title/Abstract]
#14	Search caffeine-rich beverages[Title/Abstract]
#13	Search gene-caffeine[Title/Abstract]
#12	Search dose[Title/Abstract]
#11	Search ingestion[Title/Abstract]
#10	Search caffeine-maltodextrin[Title/Abstract]
#9	Search caffeine-containing[Title/Abstract]
#8	Search alternate forms of caffeine[Title/Abstract]
#7	Search supplementation[Title/Abstract]
#6	Search intake[Title/Abstract]
#5	Search caffeinated gum[Title/Abstract]
#4	Search consumption[Title/Abstract]
#3	Search treatment[Title/Abstract]
#2	Search coffee[Title/Abstract]
#1	Search caffeine[Title/Abstract]
