# Supplementary material for: Impact of Genetic Variability on Physiological Responses to Caffeine in Humans: A Systematic Review
Source: Nutrients. 2018 Sep 25;10(10):1373. doi: 10.3390/nu10101373 (PMC6212886; doi:10.3390/nu10101373)
Supplement: Supplementary file 1 [file nutrients-10-01373-s001.zip › FigureS2 Excluded Studies.pdf]

1. Pharmacogenomic Testing for Psychotropic Medication Selection: A Systematic Review of the Assurex GeneSight Psychotropic Test. Ont Health Technol Assess Ser. 2017; **17**(4): 1-39.
2. Pharmacogenomic Testing for Psychotropic Medication Selection: A Systematic Review of the Assurex GeneSight Psychotropic Test. Ontario Health Technology Assessment Series. 2017; **17**(4): 1-39.
3. Abbasalizad Farhangi M, Mohseni F, Farajnia S, Jafarabadi M-A. Major components of metabolic syndrome and nutritional intakes in different genotype of UCP2 -866G/A gene polymorphisms in patients with NAFLD. Journal Of Translational Medicine. 2016; **14**(1): 177-.
4. Abbasalizad Farhangi M, Mohseni F, Farajnia S, Jafarabadi MA. Major components of metabolic syndrome and nutritional intakes in different genotype of UCP2 -866G/A gene polymorphisms in patients with NAFLD. J Transl Med. 2016; **14**(1): 177.
5. Aberg K, Dai F, Sun G, Keighley E, Indugula SR, Bausserman L, et al. A genome-wide linkage scan identifies multiple chromosomal regions influencing serum lipid levels in the population on the Samoan islands. J Lipid Res. 2008; **49**(10): 2169-78.
6. Aberg K, Dai F, Sun G, Keighley E, Indugula SR, Bausserman L, et al. A genome-wide linkage scan identifies multiple chromosomal regions influencing serum lipid levels in the population on the Samoan islands. Journal Of Lipid Research. 2008; **49**(10): 2169-78.
7. Aberg K, Dai F, Viali S, Tuitele J, Sun G, Indugula SR, et al. Suggestive linkage detected for blood pressure related traits on 2q and 22q in the population on the Samoan islands. BMC Med Genet. 2009; **10**: 107.
8. Aberg K, Dai F, Viali S, Tuitele J, Sun G, Indugula SR, et al. Suggestive linkage detected for blood pressure related traits on 2q and 22q in the population on the Samoan islands. BMC Medical Genetics. 2009; **10**: 107-.
9. Aboud O, Mrak RE, Boop FA, Griffin WS. Epilepsy: neuroinflammation, neurodegeneration, and APOE genotype. Acta Neuropathol Commun. 2013; **1**: 41.
10. Aboud O, Mrak RE, Boop FA, Griffin WST. Epilepsy: neuroinflammation, neurodegeneration, and APOE genotype. Acta Neuropathologica Communications. 2013; **1**: 41-.
11. Adams SV, Newcomb PA, White E. Dietary cadmium and risk of invasive postmenopausal breast cancer in the VITAL cohort. Cancer Causes Control. 2012; **23**(6): 845-54.
12. Adams SV, Newcomb PA, White E. Dietary cadmium and risk of invasive postmenopausal breast cancer in the VITAL cohort. Cancer Causes & Control: CCC. 2012; **23**(6): 845-54.
13. Ahles TA, Saykin AJ, Noll WW, Furstenberg CT, Guerin S, Cole B, et al. The relationship of APOE genotype to neuropsychological performance in long-term cancer survivors treated with standard dose chemotherapy. Psychooncology. 2003; **12**(6): 612-9.
14. Ahles TA, Saykin AJ, Noll WW, Furstenberg CT, Guerin S, Cole B, et al. The relationship of APOE genotype to neuropsychological performance in long-term cancer survivors treated with standard dose chemotherapy. Psycho-Oncology. 2003; **12**(6):

612-9.
15. Ahmad T, Lee IM, Pare G, Chasman DI, Rose L, Ridker PM, et al. Lifestyle interaction with fat mass and obesity-associated (FTO) genotype and risk of obesity in apparently healthy U.S. women. Diabetes Care. 2011; **34**(3): 675-80.
16. Ahmad T, Lee IM, Paré G, Chasman DI, Rose L, Ridker PM, et al. Lifestyle interaction with fat mass and obesity-associated (FTO) genotype and risk of obesity in apparently healthy U.S. women. Diabetes Care. 2011; **34**(3): 675-80.
17. Ahmetov I, Kulemin N, Popov D, Naumov V, Akimov E, Bravy Y, et al. Genome-wide association study identifies three novel genetic markers associated with elite endurance performance. Biol Sport. 2015; **32**(1): 3-9.
18. Ahmetov I, Kulemin N, Popov D, Naumov V, Akimov E, Bravy Y, et al. Genome-wide association study identifies three novel genetic markers associated with elite endurance performance. Biology Of Sport. 2015; **32**(1): 3-9.
19. Aja S, Moran TH. Recent advances in obesity: adiposity signaling and fat metabolism in energy homeostasis. Adv Psychosom Med. 2006; **27**: 1-23.
20. Aja S, Moran TH. Recent advances in obesity: adiposity signaling and fat metabolism in energy homeostasis. Advances In Psychosomatic Medicine. 2006; **27**: 1-23.
21. Albert PR. Transcriptional regulation of the 5-HT1A receptor: implications for mental illness. Philos Trans R Soc Lond B Biol Sci. 2012; **367**(1601): 2402-15.
22. Albert PR. Transcriptional regulation of the 5-HT1A receptor: implications for mental illness. Philosophical Transactions Of The Royal Society Of London Series B, Biological Sciences. 2012; **367**(1601): 2402-15.
23. Alharbi KK, Richardson TG, Khan IA, Syed R, Mohammed AK, Boustred CR, et al. Influence of adiposity-related genetic markers in a population of saudi arabians where other variables influencing obesity may be reduced. Dis Markers. 2014; **2014**: 758232.
24. Alharbi KK, Richardson TG, Khan IA, Syed R, Mohammed AK, Boustred CR, et al. Influence of adiposity-related genetic markers in a population of saudi arabians where other variables influencing obesity may be reduced. Disease Markers. 2014; **2014**: 758232-.
25. Allgulander C, Nilsson B. A prospective study of 86 new patients with social anxiety disorder. Acta Psychiatr Scand. 2001; **103**(6): 447-52.
26. Allgulander C, Nilsson B. A prospective study of 86 new patients with social anxiety disorder. Acta Psychiatrica Scandinavica. 2001; **103**(6): 447-52.
27. Almon R, Alvarez-Leon EE, Engfeldt P, Serra-Majem L, Magnuson A, Nilsson TK. Associations between lactase persistence and the metabolic syndrome in a cross-sectional study in the Canary Islands. Eur J Nutr. 2010; **49**(3): 141-6.
28. Almon R, Alvarez-Leon EE, Engfeldt P, Serra-Majem L, Magnuson A, Nilsson TK. Associations between lactase persistence and the metabolic syndrome in a cross-sectional study in the Canary Islands. European Journal Of Nutrition. 2010; **49**(3): 141-6.
29. Altar CA, Carhart J, Allen JD, Hall-Flavin D, Winner J, Dechairo B. Clinical Utility of Combinatorial Pharmacogenomics-Guided Antidepressant Therapy: Evidence from Three Clinical Studies. Mol Neuropsychiatry. 2015; **1**(3): 145-55.

30. Altar CA, Carhart J, Allen JD, Hall-Flavin D, Winner J, Dechairo B. Clinical Utility of Combinatorial Pharmacogenomics-Guided Antidepressant Therapy: Evidence from Three Clinical Studies. Molecular Neuropsychiatry. 2015; **1**(3): 145-55.
31. Amitai M, Kronenberg S, Carmel M, Michaelovsky E, Frisch A, Brent D, et al. Pharmacogenetics of citalopram-related side effects in children with depression and/or anxiety disorders. J Neural Transm (Vienna). 2016; **123**(11): 1347-54.
32. Amitai M, Kronenberg S, Carmel M, Michaelovsky E, Frisch A, Brent D, et al. Pharmacogenetics of citalopram-related side effects in children with depression and/or anxiety disorders. Journal Of Neural Transmission (Vienna, Austria: 1996). 2016; **123**(11): 1347-54.
33. Andero R, Choi DC, Ressler KJ. BDNF-TrkB receptor regulation of distributed adult neural plasticity, memory formation, and psychiatric disorders. Prog Mol Biol Transl Sci. 2014; **122**: 169-92.
34. Andero R, Choi DC, Ressler KJ. BDNF-TrkB receptor regulation of distributed adult neural plasticity, memory formation, and psychiatric disorders. Progress In Molecular Biology And Translational Science. 2014; **122**: 169-92.
35. Andero R, Ressler KJ. Fear extinction and BDNF: translating animal models of PTSD to the clinic. Genes Brain Behav. 2012; **11**(5): 503-12.
36. Andero R, Ressler KJ. Fear extinction and BDNF: translating animal models of PTSD to the clinic. Genes, Brain, And Behavior. 2012; **11**(5): 503-12.
37. Andersson E, Ruck C, Lavebratt C, Hedman E, Schalling M, Lindefors N, et al. Genetic polymorphisms in monoamine systems and outcome of cognitive behavior therapy for social anxiety disorder. PLoS One. 2013; **8**(11): e79015.
38. Andersson E, Rück C, Lavebratt C, Hedman E, Schalling M, Lindefors N, et al. Genetic polymorphisms in monoamine systems and outcome of cognitive behavior therapy for social anxiety disorder. PLoS One. 2013; **8**(11): e79015-e.
39. Andre K, Kampman O, Setala-Soikkeli E, Viikki M, Poutanen O, Nuolivirta T, et al. Temperament profiles, 5-HT2A genotype, and response to treatment with SSRIs in major depression. J Neural Transm (Vienna). 2010; **117**(12): 1431-4.
40. Andre K, Kampman O, Setälä-Soikkeli E, Viikki M, Poutanen O, Nuolivirta T, et al. Temperament profiles, 5-HT2A genotype, and response to treatment with SSRIs in major depression. Journal Of Neural Transmission (Vienna, Austria: 1996). 2010; **117**(12): 1431-4.
41. Angermann CE, Kaspar M, Marx A, Kittel-Schneider S, Menhofer D, Stork S, et al. A functional variant of the neuropeptide S receptor-1 gene modulates clinical outcomes and healthcare utilization in patients with systolic heart failure: results from the Interdisciplinary Network Heart Failure (INH) Study. Eur J Heart Fail. 2017; **19**(3): 314-23.
42. Angermann CE, Kaspar M, Marx A, Kittel-Schneider S, Menhofer D, Störk S, et al. A functional variant of the neuropeptide S receptor-1 gene modulates clinical outcomes and healthcare utilization in patients with systolic heart failure: results from the Interdisciplinary Network Heart Failure (INH) Study. European Journal Of Heart Failure. 2017; **19**(3): 314-23.
43. Anstey KJ, Mack HA, Christensen H, Li SC, Reglade-Meslin C, Maller J, et al. Corpus callosum size, reaction time speed and variability in mild cognitive disorders and

in a normative sample. Neuropsychologia. 2007; **45**(8): 1911-20.
44. Anstey KJ, Mack HA, Christensen H, Li S-C, Reglade-Meslin C, Maller J, et al. Corpus callosum size, reaction time speed and variability in mild cognitive disorders and in a normative sample. Neuropsychologia. 2007; **45**(8): 1911-20.
45. Antoni MH, Lutgendorf SK, Blomberg B, Carver CS, Lechner S, Diaz A, et al. Cognitive-behavioral stress management reverses anxiety-related leukocyte transcriptional dynamics. Biol Psychiatry. 2012; **71**(4): 366-72.
46. Antoni MH, Lutgendorf SK, Blomberg B, Carver CS, Lechner S, Diaz A, et al. Cognitive-behavioral stress management reverses anxiety-related leukocyte transcriptional dynamics. Biological Psychiatry. 2012; **71**(4): 366-72.
47. Anttila AK, Rasanen L, Leinonen EV. Fluvoxamine augmentation increases serum mirtazapine concentrations three- to fourfold. Ann Pharmacother. 2001; **35**(10): 1221-3.
48. Anttila AK, Rasanen L, Leinonen EV. Fluvoxamine augmentation increases serum mirtazapine concentrations three- to fourfold. The Annals Of Pharmacotherapy. 2001; **35**(10): 1221-3.
49. Aoki Y, Yoshida K, Nishizawa D, Kasai S, Ichinohe T, Ikeda K, et al. Factors that affect intravenous patient-controlled analgesia for postoperative pain following orthognathic surgery for mandibular prognathism. PLoS One. 2014; **9**(6): e98548.
50. Aoki Y, Yoshida K, Nishizawa D, Kasai S, Ichinohe T, Ikeda K, et al. Factors that affect intravenous patient-controlled analgesia for postoperative pain following orthognathic surgery for mandibular prognathism. PLoS One. 2014; **9**(6): e98548-e.
51. Archer T, Oscar-Berman M, Blum K, Gold M. Epigenetic Modulation of Mood Disorders. J Genet Syndr Gene Ther. 2013; **4**(120).
52. Archer T, Oscar-Berman M, Blum K, Gold M. Epigenetic Modulation of Mood Disorders. Journal Of Genetic Syndromes & Gene Therapy. 2013; **4**(120).
53. Ardisson Korat AV, Willett WC, Hu FB. Diet, lifestyle, and genetic risk factors for type 2 diabetes: a review from the Nurses' Health Study, Nurses' Health Study 2, and Health Professionals' Follow-up Study. Curr Nutr Rep. 2014; **3**(4): 345-54.
54. Ardisson Korat AV, Willett WC, Hu FB. Diet, lifestyle, and genetic risk factors for type 2 diabetes: a review from the Nurses' Health Study, Nurses' Health Study 2, and Health Professionals' Follow-up Study. Current Nutrition Reports. 2014; **3**(4): 345-54.
55. Arranz MJ, Munro JC. Toward understanding genetic risk for differential antipsychotic response in individuals with schizophrenia. Expert Rev Clin Pharmacol. 2011; **4**(3): 389-405.
56. Arranz MJ, Munro JC. Toward understanding genetic risk for differential antipsychotic response in individuals with schizophrenia. Expert Review Of Clinical Pharmacology. 2011; **4**(3): 389-405.
57. Arvaniti M, Polli FS, Kohlmeier KA, Thomsen MS, Andreasen JT. Loss of Lypd6 leads to reduced anxiety-like behaviour and enhanced responses to nicotine. Prog Neuropsychopharmacol Biol Psychiatry. 2018; **82**: 86-94.
58. Arvaniti M, Polli FS, Kohlmeier KA, Thomsen MS, Andreasen JT. Loss of Lypd6 leads to reduced anxiety-like behaviour and enhanced responses to nicotine. Progress In Neuro-Psychopharmacology & Biological Psychiatry. 2018; **82**: 86-94.
59. Atigari OV, Hogan C, Healy D. Doxycycline and suicidality. BMJ Case Rep. 2013;

2013.
60. Atigari OV, Hogan C, Healy D. Doxycycline and suicidality. BMJ Case Reports. 2013; **2013**.
61. Auxemery Y. [Posttraumatic stress disorder (PTSD) as a consequence of the interaction between an individual genetic susceptibility, a traumatogenic event and a social context]. Encephale. 2012; **38**(5): 373-80.
62. Auxéméry Y. [Posttraumatic stress disorder (PTSD) as a consequence of the interaction between an individual genetic susceptibility, a traumatogenic event and a social context]. L'encephale. 2012; **38**(5): 373-80.
63. Baeta-Corral R, Johansson B, Gimenez-Llort L. Long-term Treatment with Low-Dose Caffeine Worsens BPSD-Like Profile in 3xTg-AD Mice Model of Alzheimer's Disease and Affects Mice with Normal Aging. Front Pharmacol. 2018; **9**: 79.
64. Baeta-Corral R, Johansson B, Giménez-Llort L. Long-term Treatment with Low-Dose Caffeine Worsens BPSD-Like Profile in 3xTg-AD Mice Model of Alzheimer's Disease and Affects Mice with Normal Aging. Frontiers In Pharmacology. 2018; **9**: 79-.
65. Bagdy G, Juhasz G. Biomarkers for personalised treatment in psychiatric diseases. Expert Opin Med Diagn. 2013; **7**(5): 417-22.
66. Bagdy G, Juhasz G. Biomarkers for personalised treatment in psychiatric diseases. Expert Opinion On Medical Diagnostics. 2013; **7**(5): 417-22.
67. Baggio S, Mussulini BH, de Oliveira DL, Gerlai R, Rico EP. Embryonic alcohol exposure leading to social avoidance and altered anxiety responses in adult zebrafish. Behav Brain Res. 2017.
68. Baggio S, Mussulini BH, de Oliveira DL, Gerlai R, Rico EP. Embryonic alcohol exposure leading to social avoidance and altered anxiety responses in adult zebrafish. Behavioural Brain Research. 2017.
69. Bahi A, Dreyer J-L. Viral-mediated overexpression of the Myelin Transcription Factor 1 (MyT1) in the dentate gyrus attenuates anxiety- and ethanol-related behaviors in rats. Psychopharmacology. 2017; **234**(12): 1829-40.
70. Bahi A, Dreyer JL. Viral-mediated overexpression of the Myelin Transcription Factor 1 (MyT1) in the dentate gyrus attenuates anxiety- and ethanol-related behaviors in rats. Psychopharmacology (Berl). 2017; **234**(12): 1829-40.
71. Baik I, Lee S, Kim SH, Shin C. A lipoprotein lipase gene polymorphism interacts with consumption of alcohol and unsaturated fat to modulate serum HDL-cholesterol concentrations. J Nutr. 2013; **143**(10): 1618-25.
72. Baik I, Lee S, Kim SH, Shin C. A lipoprotein lipase gene polymorphism interacts with consumption of alcohol and unsaturated fat to modulate serum HDL-cholesterol concentrations. The Journal Of Nutrition. 2013; **143**(10): 1618-25.
73. Baldinger P, Hahn A, Mitterhauser M, Kranz GS, Friedl M, Wadsak W, et al. Impact of COMT genotype on serotonin-1A receptor binding investigated with PET. Brain Struct Funct. 2014; **219**(6): 2017-28.
74. Baldinger P, Hahn A, Mitterhauser M, Kranz GS, Friedl M, Wadsak W, et al. Impact of COMT genotype on serotonin-1A receptor binding investigated with PET. Brain Structure & Function. 2014; **219**(6): 2017-28.
75. Ball S, Marangell LB, Lipsius S, Russell JM. Brain-derived neurotrophic factor in generalized anxiety disorder: results from a duloxetine clinical trial. Prog

Neuropsychopharmacol Biol Psychiatry. 2013; **43**: 217-21.
76. Ball S, Marangell LB, Lipsius S, Russell JM. Brain-derived neurotrophic factor in generalized anxiety disorder: results from a duloxetine clinical trial. Progress In Neuro-Psychopharmacology & Biological Psychiatry. 2013; **43**: 217-21.
77. Barbeito S, Vega P, Ruiz de Azua S, Balanza-Martinez V, Colom F, Lorente E, et al. Integrated treatment of first episode psychosis with online training (e-learning): study protocol for a randomised controlled trial. Trials. 2014; **15**: 416.
78. Barbeito S, Vega P, Ruiz de Azua S, Balanza-Martinez V, Colom F, Lorente E, et al. Integrated treatment of first episode psychosis with online training (e-learning): study protocol for a randomised controlled trial. Trials. 2014; **15**: 416-.
79. Barber B, McNeely M, Chan KM, Beaudry R, Olson J, Harris J, et al. Intraoperative brief electrical stimulation (BES) for prevention of shoulder dysfunction after oncologic neck dissection: study protocol for a randomized controlled trial. Trials. 2015; **16**: 240.
80. Barber B, McNeely M, Chan KM, Beaudry R, Olson J, Harris J, et al. Intraoperative brief electrical stimulation (BES) for prevention of shoulder dysfunction after oncologic neck dissection: study protocol for a randomized controlled trial. Trials. 2015; **16**: 240-.
81. Barfield WL, Uaesoontrachoon K, Wu CS, Lin S, Chen Y, Wang PC, et al. Eccentric muscle challenge shows osteopontin polymorphism modulation of muscle damage. Hum Mol Genet. 2014; **23**(15): 4043-50.
82. Barfield WL, Uaesoontrachoon K, Wu C-S, Lin S, Chen Y, Wang PC, et al. Eccentric muscle challenge shows osteopontin polymorphism modulation of muscle damage. Human Molecular Genetics. 2014; **23**(15): 4043-50.
83. Baroncelli GI, Federico G, Bertelloni S, Ceccarelli C, Cupelli D, Saggese G. Vitamin-D receptor genotype does not predict bone mineral density, bone turnover, and growth in prepubertal children. Horm Res. 1999; **51**(3): 150-6.
84. Baroncelli GI, Federico G, Bertelloni S, Ceccarelli C, Cupelli D, Saggese G. Vitamin-D receptor genotype does not predict bone mineral density, bone turnover, and growth in prepubertal children. Hormone Research. 1999; **51**(3): 150-6.
85. Barr CS, Newman TK, Lindell S, Shannon C, Champoux M, Lesch KP, et al. Interaction between serotonin transporter gene variation and rearing condition in alcohol preference and consumption in female primates. Arch Gen Psychiatry. 2004; **61**(11): 1146-52.
86. Barr CS, Newman TK, Lindell S, Shannon C, Champoux M, Lesch KP, et al. Interaction between serotonin transporter gene variation and rearing condition in alcohol preference and consumption in female primates. Archives Of General Psychiatry. 2004; **61**(11): 1146-52.
87. Barros CM, Araujo-Neto AP, Lopes TR, Barros MA, Motta FJ, Canalle R, et al. Association of the rs7903146 and rs12255372 polymorphisms in the TCF7L2 gene with type 2 diabetes in a population from northeastern Brazil. Genet Mol Res. 2014; **13**(3): 7889-98.
88. Barros CMAR, Araujo-Neto AP, Lopes TR, Barros MAL, Motta FJN, Canalle R, et al. Association of the rs7903146 and rs12255372 polymorphisms in the TCF7L2 gene with type 2 diabetes in a population from northeastern Brazil. Genetics And Molecular

Research: GMR. 2014; **13**(3): 7889-98.
89. Bartolucci G, Younger J. Tentative classification of neuropsychiatric disturbances in Prader-Willi syndrome. J Intellect Disabil Res. 1994; **38 (Pt 6)**: 621-9.
90. Bartolucci G, Younger J. Tentative classification of neuropsychiatric disturbances in Prader-Willi syndrome. Journal Of Intellectual Disability Research: JIDR. 1994; **38 (Pt 6)**: 621-9.
91. Basiri MG, Sotoudeh G, Alvandi E, Djalali M, Eshraghian MR, Noorshahi N, et al. APOA2 -256T>C polymorphism interacts with saturated fatty acids intake to affect anthropometric and hormonal variables in type 2 diabetic patients. Genes Nutr. 2015; **10**(3): 464.
92. Basiri MG, Sotoudeh G, Alvandi E, Djalali M, Eshraghian MR, Noorshahi N, et al. APOA2 -256T>C polymorphism interacts with saturated fatty acids intake to affect anthropometric and hormonal variables in type 2 diabetic patients. Genes & Nutrition. 2015; **10**(3): 464-.
93. Baskar V, Gangadhar BH, Park SW, Nile SH. A simple and efficient Agrobacterium tumefaciens-mediated plant transformation of Brassica rapa ssp. pekinensis. 3 Biotech. 2016; **6**(1): 88.
94. Baskar V, Gangadhar BH, Park SW, Nile SH. A simple and efficient Agrobacterium tumefaciens-mediated plant transformation of Brassica rapa ssp. pekinensis. 3 Biotech. 2016; **6**(1): 88-.
95. Basu SK, Kumar D, Ganguly N, Rao KV, Sharma P. Mycobacterium tuberculosis secreted antigen (MTSA-10) inhibits macrophage response to lipopolysaccharide by redox regulation of phosphatases. Indian J Exp Biol. 2009; **47**(6): 505-19.
96. Basu SK, Kumar D, Ganguly N, Rao KVS, Sharma P. Mycobacterium tuberculosis secreted antigen (MTSA-10) inhibits macrophage response to lipopolysaccharide by redox regulation of phosphatases. Indian Journal Of Experimental Biology. 2009; **47**(6): 505-19.
97. Bath KG, Jing DQ, Dincheva I, Neeb CC, Pattwell SS, Chao MV, et al. BDNF Val66Met impairs fluoxetine-induced enhancement of adult hippocampus plasticity. Neuropsychopharmacology. 2012; **37**(5): 1297-304.
98. Bath KG, Jing DQ, Dincheva I, Neeb CC, Pattwell SS, Chao MV, et al. BDNF Val66Met impairs fluoxetine-induced enhancement of adult hippocampus plasticity. Neuropsychopharmacology: Official Publication Of The American College Of Neuropsychopharmacology. 2012; **37**(5): 1297-304.
99. Belalcazar LM, Papandonatos GD, McCaffery JM, Peter I, Pajewski NM, Erar B, et al. A common variant in the CLDN7/ELP5 locus predicts adiponectin change with lifestyle intervention and improved fitness in obese individuals with diabetes. Physiol Genomics. 2015; **47**(6): 215-24.
100. Belalcazar LM, Papandonatos GD, McCaffery JM, Peter I, Pajewski NM, Erar B, et al. A common variant in the CLDN7/ELP5 locus predicts adiponectin change with lifestyle intervention and improved fitness in obese individuals with diabetes. Physiological Genomics. 2015; **47**(6): 215-24.
101. Bellis C, Cox HC, Dyer TD, Charlesworth JC, Begley KN, Quinlan S, et al. Linkage mapping of CVD risk traits in the isolated Norfolk Island population. Hum Genet. 2008; **124**(5): 543-52.

102. Bellis C, Cox HC, Dyer TD, Charlesworth JC, Begley KN, Quinlan S, et al. Linkage mapping of CVD risk traits in the isolated Norfolk Island population. Human Genetics. 2008; **124**(5): 543-52.
103. Bérard A, Gaedigk A, Sheehy O, Chambers C, Roth M, Bozzo P, et al. Association between CYP2D6 Genotypes and the Risk of Antidepressant Discontinuation, Dosage Modification and the Occurrence of Maternal Depression during Pregnancy. Frontiers In Pharmacology. 2017; **8**: 402-.
104. Berard A, Gaedigk A, Sheehy O, Chambers C, Roth M, Bozzo P, et al. Association between CYP2D6 Genotypes and the Risk of Antidepressant Discontinuation, Dosage Modification and the Occurrence of Maternal Depression during Pregnancy. Front Pharmacol. 2017; **8**: 402.
105. Bergen SE, Gardner CO, Kendler KS. Age-related changes in heritability of behavioral phenotypes over adolescence and young adulthood: a meta-analysis. Twin Res Hum Genet. 2007; **10**(3): 423-33.
106. Bergen SE, Gardner CO, Kendler KS. Age-related changes in heritability of behavioral phenotypes over adolescence and young adulthood: a meta-analysis. Twin Research And Human Genetics: The Official Journal Of The International Society For Twin Studies. 2007; **10**(3): 423-33.
107. Bermejo JL. Gene-environment interactions and familial relative risks. Hum Hered. 2008; **66**(3): 170-9.
108. Bermejo JL. Gene-environment interactions and familial relative risks. Human Heredity. 2008; **66**(3): 170-9.
109. Bershad AK, Weafer JJ, Kirkpatrick MG, Wardle MC, Miller MA, de Wit H. Oxytocin receptor gene variation predicts subjective responses to MDMA. Soc Neurosci. 2016; **11**(6): 592-9.
110. Bershad AK, Weafer JJ, Kirkpatrick MG, Wardle MC, Miller MA, de Wit H. Oxytocin receptor gene variation predicts subjective responses to MDMA. Social Neuroscience. 2016; **11**(6): 592-9.
111. Bezerra AM, Sant'Ana TA, Gomes AV, de Lacerda Vidal AK, Muniz MT. Tyms double (2R) and triple repeat (3R) confers risk for human oral squamous cell carcinoma. Mol Biol Rep. 2014; **41**(12): 7737-42.
112. Bezerra AM, Sant'Ana TA, Gomes AV, de Lacerda Vidal AK, Muniz MTC. Tyms double (2R) and triple repeat (3R) confers risk for human oral squamous cell carcinoma. Molecular Biology Reports. 2014; **41**(12): 7737-42.
113. Bhattacharya SS, Kulka M, Lampel KA, Cebula TA, Goswami BB. Use of reverse transcription and PCR to discriminate between infectious and non-infectious hepatitis A virus. J Virol Methods. 2004; **116**(2): 181-7.
114. Bhattacharya SS, Kulka M, Lampel KA, Cebula TA, Goswami BB. Use of reverse transcription and PCR to discriminate between infectious and non-infectious hepatitis A virus. Journal Of Virological Methods. 2004; **116**(2): 181-7.
115. Biggin A, Munns CF. Long-Term Bisphosphonate Therapy in Osteogenesis Imperfecta. Curr Osteoporos Rep. 2017; **15**(5): 412-8.
116. Biggin A, Munns CF. Long-Term Bisphosphonate Therapy in Osteogenesis Imperfecta. Current Osteoporosis Reports. 2017; **15**(5): 412-8.
117. Bilgic A, Toker A, Isik U, Kilinc I. Serum brain-derived neurotrophic factor, glial-

derived neurotrophic factor, nerve growth factor, and neurotrophin-3 levels in children with attention-deficit/hyperactivity disorder. Eur Child Adolesc Psychiatry. 2017; **26**(3): 355-63.
118. Bilgiç A, Toker A, Işık Ü, Kılınç İ. Serum brain-derived neurotrophic factor, glial-derived neurotrophic factor, nerve growth factor, and neurotrophin-3 levels in children with attention-deficit/hyperactivity disorder. European Child & Adolescent Psychiatry. 2017; **26**(3): 355-63.
119. Binder EB, Owens MJ, Liu W, Deveau TC, Rush AJ, Trivedi MH, et al. Association of polymorphisms in genes regulating the corticotropin-releasing factor system with antidepressant treatment response. Arch Gen Psychiatry. 2010; **67**(4): 369-79.
120. Binder EB, Owens MJ, Liu W, Deveau TC, Rush AJ, Trivedi MH, et al. Association of polymorphisms in genes regulating the corticotropin-releasing factor system with antidepressant treatment response. Archives Of General Psychiatry. 2010; **67**(4): 369-79.
121. Bjorkholm C, Monteggia LM. BDNF - a key transducer of antidepressant effects. Neuropharmacology. 2016; **102**: 72-9.
122. Björkholm C, Monteggia LM. BDNF - a key transducer of antidepressant effects. Neuropharmacology. 2016; **102**: 72-9.
123. Blair RH, Trichler DL, Gaille DP. Mathematical and statistical modeling in cancer systems biology. Front Physiol. 2012; **3**: 227.
124. Blair RH, Trichler DL, Gaille DP. Mathematical and statistical modeling in cancer systems biology. Frontiers In Physiology. 2012; **3**: 227-.
125. Blaszczyk E, Gawlik A. [Neurotrophins, VEGF and matrix metalloproteinases: new markers or causative factors of metabolic syndrome components?]. Pediatr Endocrinol Diabetes Metab. 2016; **22**(3).
126. Błaszczyk E, Gawlik A. [Neurotrophins, VEGF and matrix metalloproteinases: new markers or causative factors of metabolic syndrome components?]. Pediatric Endocrinology, Diabetes, And Metabolism. 2016; **22**(3).
127. Bloch B, Reshef A, Cohen T, Tafla A, Gathas S, Israel S, et al. Preliminary effects of bupropion and the promoter region (HTTLPR) serotonin transporter (SLC6A4) polymorphism on smoking behavior in schizophrenia. Psychiatry Res. 2010; **175**(1-2): 38-42.
128. Bloch B, Reshef A, Cohen T, Tafla A, Gathas S, Israel S, et al. Preliminary effects of bupropion and the promoter region (HTTLPR) serotonin transporter (SLC6A4) polymorphism on smoking behavior in schizophrenia. Psychiatry Research. 2010; **175**(1-2): 38-42.
129. Bloss CS, Wineinger NE, Darst BF, Schork NJ, Topol EJ. Impact of direct-to-consumer genomic testing at long term follow-up. J Med Genet. 2013; **50**(6): 393-400.
130. Bloss CS, Wineinger NE, Darst BF, Schork NJ, Topol EJ. Impact of direct-to-consumer genomic testing at long term follow-up. Journal Of Medical Genetics. 2013; **50**(6): 393-400.
131. Boccia S, Persiani R, La Torre G, Rausei S, Arzani D, Gianfagna F, et al. Sulfotransferase 1A1 polymorphism and gastric cancer risk: a pilot case-control study. Cancer Lett. 2005; **229**(2): 235-43.

132. Boccia S, Persiani R, La Torre G, Rausei S, Arzani D, Gianfagna F, et al. Sulfotransferase 1A1 polymorphism and gastric cancer risk: a pilot case-control study. Cancer Letters. 2005; **229**(2): 235-43.
133. Bodhini D, Gaal S, Shatwan I, Ramya K, Ellahi B, Surendran S, et al. Interaction between TCF7L2 polymorphism and dietary fat intake on high density lipoprotein cholesterol. PLoS One. 2017; **12**(11): e0188382.
134. Bodhini D, Gaal S, Shatwan I, Ramya K, Ellahi B, Surendran S, et al. Interaction between TCF7L2 polymorphism and dietary fat intake on high density lipoprotein cholesterol. PLoS One. 2017; **12**(11): e0188382-e.
135. Boer JM, Kuivenhoven JA, Feskens EJ, Schouten EG, Havekes LM, Seidell JC, et al. Physical activity modulates the effect of a lipoprotein lipase mutation (D9N) on plasma lipids and lipoproteins. Clin Genet. 1999; **56**(2): 158-63.
136. Boer JM, Kuivenhoven JA, Feskens EJ, Schouten EG, Havekes LM, Seidell JC, et al. Physical activity modulates the effect of a lipoprotein lipase mutation (D9N) on plasma lipids and lipoproteins. Clinical Genetics. 1999; **56**(2): 158-63.
137. Bol SM, Moerland PD, Limou S, van Remmerden Y, Coulonges C, van Manen D, et al. Genome-wide association study identifies single nucleotide polymorphism in DYRK1A associated with replication of HIV-1 in monocyte-derived macrophages. PLoS One. 2011; **6**(2): e17190.
138. Bol SM, Moerland PD, Limou S, van Remmerden Y, Coulonges C, van Manen D, et al. Genome-wide association study identifies single nucleotide polymorphism in DYRK1A associated with replication of HIV-1 in monocyte-derived macrophages. PLoS One. 2011; **6**(2): e17190-e.
139. Bondareva EA, Bleer AN, Godina EZ. [Association between G/A - Polymorphism of EPAS1 gene and the maximal level of oxygen consumption in Russian athletes. Fiziol Cheloveka. 2016; **42**(3): 120-4.
140. Bondareva EA, Bleer AN, Godina EZ. [Association between G/A - Polymorphism of EPAS1 gene and the maximal level of oxygen consumption in Russian athletes. Fiziologiia Cheloveka. 2016; **42**(3): 120-4.
141. Bonnet U. Moclobemide: therapeutic use and clinical studies. CNS Drug Rev. 2003; **9**(1): 97-140.
142. Bonnet U. Moclobemide: therapeutic use and clinical studies. CNS Drug Reviews. 2003; **9**(1): 97-140.
143. Booij L, Casey KF, Antunes JM, Szyf M, Joober R, Israel M, et al. DNA methylation in individuals with anorexia nervosa and in matched normal-eater controls: A genome-wide study. Int J Eat Disord. 2015; **48**(7): 874-82.
144. Booij L, Casey KF, Antunes JM, Szyf M, Joober R, Israël M, et al. DNA methylation in individuals with anorexia nervosa and in matched normal-eater controls: A genome-wide study. The International Journal Of Eating Disorders. 2015; **48**(7): 874-82.
145. Borusenius SN, Belozerskaia NA, Merkulova NA, Vorob'ev VI. [Palindromic, repeating and unique sequences in the macronuclear DNA of Tetrahymena pyriformis GL infusoria]. Mol Biol (Mosk). 1978; **12**(3): 676-88.
146. Borusenius SN, Belozerskaia NA, Merkulova NA, Vorob'ev VI. [Palindromic, repeating and unique sequences in the macronuclear DNA of Tetrahymena pyriformis

GL infusoria]. Molekuliarnaia Biologiia. 1978; **12**(3): 676-88.
147. Bos I, De Boever P, Int Panis L, Meeusen R. Physical activity, air pollution and the brain. Sports Med. 2014; **44**(11): 1505-18.
148. Bos I, De Boever P, Int Panis L, Meeusen R. Physical activity, air pollution and the brain. Sports Medicine (Auckland, NZ). 2014; **44**(11): 1505-18.
149. Bose CK, Basu N. PARP inhibitors and more. J Turk Ger Gynecol Assoc. 2015; **16**(2): 107-10.
150. Bose CK, Basu N. PARP inhibitors and more. Journal Of The Turkish German Gynecological Association. 2015; **16**(2): 107-10.
151. Bosse Y, Amos CI. A Decade of GWAS Results in Lung Cancer. Cancer Epidemiol Biomarkers Prev. 2018; **27**(4): 363-79.
152. Bossé Y, Amos CI. A Decade of GWAS Results in Lung Cancer. Cancer Epidemiology, Biomarkers & Prevention: A Publication Of The American Association For Cancer Research, Cosponsored By The American Society Of Preventive Oncology. 2018; **27**(4): 363-79.
153. Boucher BJ. Inadequate vitamin D status: does it contribute to the disorders comprising syndrome 'X'? Br J Nutr. 1998; **79**(4): 315-27.
154. Boucher BJ. Inadequate vitamin D status: does it contribute to the disorders comprising syndrome 'X'? The British Journal Of Nutrition. 1998; **79**(4): 315-27.
155. Boulter AC, Quinlan J, Miro-Herrans AT, Pearson LN, Todd NL, Gravlee CC, et al. Interaction of Alu Polymorphisms and Novel Measures of Discrimination in Association with Blood Pressure in African Americans Living in Tallahassee, Florida. Hum Biol. 2015; **87**(4): 295-305.
156. Boulter AC, Quinlan J, Miró-Herrans AT, Pearson LN, Todd NL, Gravlee CC, et al. Interaction of Alu Polymorphisms and Novel Measures of Discrimination in Association with Blood Pressure in African Americans Living in Tallahassee, Florida. Human Biology. 2015; **87**(4): 295-305.
157. Bourin M, David DJ, Jolliet P, Gardier A. [Mechanism of action of antidepressants and therapeutic perspectives]. Therapie. 2002; **57**(4): 385-96.
158. Bourin M, David DJP, Jolliet P, Gardier A. [Mechanism of action of antidepressants and therapeutic perspectives]. Therapie. 2002; **57**(4): 385-96.
159. Bownik H, Saab S. The effects of hepatitis C recurrence on health-related quality of life in liver transplant recipients. Liver Int. 2010; **30**(1): 19-30.
160. Bownik H, Saab S. The effects of hepatitis C recurrence on health-related quality of life in liver transplant recipients. Liver International: Official Journal Of The International Association For The Study Of The Liver. 2010; **30**(1): 19-30.
161. Brackett J, Krull KR, Scheurer ME, Liu W, Srivastava DK, Stovall M, et al. Antioxidant enzyme polymorphisms and neuropsychological outcomes in medulloblastoma survivors: a report from the Childhood Cancer Survivor Study. Neuro Oncol. 2012; **14**(8): 1018-25.
162. Brackett J, Krull KR, Scheurer ME, Liu W, Srivastava DK, Stovall M, et al. Antioxidant enzyme polymorphisms and neuropsychological outcomes in medulloblastoma survivors: a report from the Childhood Cancer Survivor Study. Neuro-Oncology. 2012; **14**(8): 1018-25.
163. Bradley LA, Haddow HRM, Palomaki GE. Treatment of mucopolysaccharidosis

type II (Hunter syndrome): results from a systematic evidence review. Genet Med. 2017; **19**(11): 1187-201.
164. Bradley LA, Haddow HRM, Palomaki GE. Treatment of mucopolysaccharidosis type II (Hunter syndrome): results from a systematic evidence review. Genetics In Medicine: Official Journal Of The American College Of Medical Genetics. 2017; **19**(11): 1187-201.
165. Brady ML, Pilli J, Lorenz-Guertin JM, Das S, Moon CE, Graff N, et al. Depolarizing, inhibitory GABA type A receptor activity regulates GABAergic synapse plasticity via ERK and BDNF signaling. Neuropharmacology. 2018; **128**: 324-39.
166. Brady ML, Pilli J, Lorenz-Guertin JM, Das S, Moon CE, Graff N, et al. Depolarizing, inhibitory GABA type A receptor activity regulates GABAergic synapse plasticity via ERK and BDNF signaling. Neuropharmacology. 2018; **128**: 324-39.
167. Braga V, Mottes M, Mirandola S, Lisi V, Malerba G, Sartori L, et al. Association of CTR and COLIA1 alleles with BMD values in peri- and postmenopausal women. Calcif Tissue Int. 2000; **67**(5): 361-6.
168. Braga V, Mottes M, Mirandola S, Lisi V, Malerba G, Sartori L, et al. Association of CTR and COLIA1 alleles with BMD values in peri- and postmenopausal women. Calcified Tissue International. 2000; **67**(5): 361-6.
169. Braun D, Feinstein DL. The locus coeruleus neuroprotective drug vindeburnol normalizes behavior in the 5xFAD transgenic mouse model of Alzheimer's disease. Brain Res. 2017.
170. Braun D, Feinstein DL. The locus coeruleus neuroprotective drug vindeburnol normalizes behavior in the 5xFAD transgenic mouse model of Alzheimer's disease. Brain Research. 2017.
171. Brockmoller J, Cascorbi I, Kerb R, Sachse C, Roots I. Polymorphisms in xenobiotic conjugation and disease predisposition. Toxicol Lett. 1998; **102-103**: 173-83.
172. Brockmöller J, Cascorbi I, Kerb R, Sachse C, Roots I. Polymorphisms in xenobiotic conjugation and disease predisposition. Toxicology Letters. 1998; **102-103**: 173-83.
173. Broocks A, Ahrendt U, Sommer M. [Physical training in the treatment of depressive disorders]. Psychiatr Prax. 2007; **34 Suppl 3**: S300-4.
174. Broocks A, Ahrendt U, Sommer M. [Physical training in the treatment of depressive disorders]. Psychiatrische Praxis. 2007; **34 Suppl 3**: S300-S4.
175. Browne CA, Erickson RL, Blendy JA, Lucki I. Genetic variation in the behavioral effects of buprenorphine in female mice derived from a murine model of the OPRM1 A118G polymorphism. Neuropharmacology. 2017; **117**: 401-7.
176. Browne CA, Erickson RL, Blendy JA, Lucki I. Genetic variation in the behavioral effects of buprenorphine in female mice derived from a murine model of the OPRM1 A118G polymorphism. Neuropharmacology. 2017; **117**: 401-7.
177. Bruch JP, Alvares DASMR, Alves BC, Dall'alba V. REDUCED HAND GRIP STRENGTH IN OVERWEIGHT AND OBESE CHRONIC HEPATITIS C PATIENTS. Arq Gastroenterol. 2016; **53**(1): 31-5.
178. Bruch JP, Álvares-Da-Silva MR, Alves BC, Dall'alba V. REDUCED HAND GRIP STRENGTH IN OVERWEIGHT AND OBESE CHRONIC HEPATITIS C PATIENTS. Arquivos De Gastroenterologia. 2016; **53**(1): 31-5.

179. Bruinenberg VM, van der Goot E, van Vliet D, de Groot MJ, Mazzola PN, Heiner-Fokkema MR, et al. The Behavioral Consequence of Phenylketonuria in Mice Depends on the Genetic Background. Front Behav Neurosci. 2016; **10**: 233.
180. Bruinenberg VM, van der Goot E, van Vliet D, de Groot MJ, Mazzola PN, Heiner-Fokkema MR, et al. The Behavioral Consequence of Phenylketonuria in Mice Depends on the Genetic Background. Frontiers In Behavioral Neuroscience. 2016; **10**: 233-.
181. Bruneau ML, Jr., Johnson BT, Huedo-Medina TB, Larson KA, Ash GI, Pescatello LS. The blood pressure response to acute and chronic aerobic exercise: A meta-analysis of candidate gene association studies. J Sci Med Sport. 2016; **19**(5): 424-31.
182. Bruneau ML, Jr., Johnson BT, Huedo-Medina TB, Larson KA, Ash GI, Pescatello LS. The blood pressure response to acute and chronic aerobic exercise: A meta-analysis of candidate gene association studies. Journal Of Science And Medicine In Sport. 2016; **19**(5): 424-31.
183. Brunoni AR, Boggio PS, Fregni F. Can the 'yin and yang' BDNF hypothesis be used to predict the effects of rTMS treatment in neuropsychiatry? Med Hypotheses. 2008; **71**(2): 279-82.
184. Brunoni AR, Boggio PS, Fregni F. Can the 'yin and yang' BDNF hypothesis be used to predict the effects of rTMS treatment in neuropsychiatry? Medical Hypotheses. 2008; **71**(2): 279-82.
185. Buford TW, Hsu FC, Brinkley TE, Carter CS, Church TS, Dodson JA, et al. Genetic influence on exercise-induced changes in physical function among mobility-limited older adults. Physiol Genomics. 2014; **46**(5): 149-58.
186. Buford TW, Hsu F-C, Brinkley TE, Carter CS, Church TS, Dodson JA, et al. Genetic influence on exercise-induced changes in physical function among mobility-limited older adults. Physiological Genomics. 2014; **46**(5): 149-58.
187. Buren S, Young EM, Sweeny EA, Lopez-Torrejon G, Veldhuizen M, Voigt CA, et al. Formation of Nitrogenase NifDK Tetramers in the Mitochondria of Saccharomyces cerevisiae. ACS Synth Biol. 2017; **6**(6): 1043-55.
188. Burén S, Young EM, Sweeny EA, Lopez-Torrejón G, Veldhuizen M, Voigt CA, et al. Formation of Nitrogenase NifDK Tetramers in the Mitochondria of Saccharomyces cerevisiae. ACS Synthetic Biology. 2017; **6**(6): 1043-55.
189. Bus BA, Molendijk ML, Penninx BJ, Buitelaar JK, Kenis G, Prickaerts J, et al. Determinants of serum brain-derived neurotrophic factor. Psychoneuroendocrinology. 2011; **36**(2): 228-39.
190. Bus BAA, Molendijk ML, Penninx BJWH, Buitelaar JK, Kenis G, Prickaerts J, et al. Determinants of serum brain-derived neurotrophic factor. Psychoneuroendocrinology. 2011; **36**(2): 228-39.
191. Cabrera Figueroa S, Iglesias Gomez A, Sanchez Martin A, de la Paz Valverde Merino M, Dominguez-Gil Hurle A, Cordero Sanchez M. Long-term efficacy and safety of efavirenz dose reduction to 200 mg once daily in a Caucasian patient with HIV. Clin Drug Investig. 2010; **30**(6): 405-11.
192. Cabrera Figueroa S, Iglesias Gómez A, Sánchez Martín A, de la Paz Valverde Merino M, Domínguez-Gil Hurlé A, Cordero Sánchez M. Long-term efficacy and safety of efavirenz dose reduction to 200 mg once daily in a Caucasian patient with HIV. Clinical Drug Investigation. 2010; **30**(6): 405-11.

193. Cacabelos R. Molecular pathology and pharmacogenomics in Alzheimer's disease: polygenic-related effects of multifactorial treatments on cognition, anxiety and depression. Methods Find Exp Clin Pharmacol. 2007; **29 Suppl A**: 1-91.
194. Cacabelos R. Molecular pathology and pharmacogenomics in Alzheimer's disease: polygenic-related effects of multifactorial treatments on cognition, anxiety and depression. Methods And Findings In Experimental And Clinical Pharmacology. 2007; **29 Suppl A**: 1-91.
195. Cai G, Cole SA, Butte N, Bacino C, Diego V, Tan K, et al. A quantitative trait locus on chromosome 18q for physical activity and dietary intake in Hispanic children. Obesity (Silver Spring). 2006; **14**(9): 1596-604.
196. Cai G, Cole SA, Butte N, Bacino C, Diego V, Tan K, et al. A quantitative trait locus on chromosome 18q for physical activity and dietary intake in Hispanic children. Obesity (Silver Spring, Md). 2006; **14**(9): 1596-604.
197. Calarge CA, Ellingrod VL, Zimmerman B, Bliziotes MM, Schlechte JA. Variants of the serotonin transporter gene, selective serotonin reuptake inhibitors, and bone mineral density in risperidone-treated boys: a reanalysis of data from a cross-sectional study with emphasis on pharmacogenetics. J Clin Psychiatry. 2011; **72**(12): 1685-90.
198. Calarge CA, Ellingrod VL, Zimmerman B, Bliziotes MM, Schlechte JA. Variants of the serotonin transporter gene, selective serotonin reuptake inhibitors, and bone mineral density in risperidone-treated boys: a reanalysis of data from a cross-sectional study with emphasis on pharmacogenetics. The Journal Of Clinical Psychiatry. 2011; **72**(12): 1685-90.
199. Cameron JD, Riou ME, Tesson F, Goldfield GS, Rabasa-Lhoret R, Brochu M, et al. The TaqIA RFLP is associated with attenuated intervention-induced body weight loss and increased carbohydrate intake in post-menopausal obese women. Appetite. 2013; **60**(1): 111-6.
200. Cameron JD, Riou M-È, Tesson F, Goldfield GS, Rabasa-Lhoret R, Brochu M, et al. The TaqIA RFLP is associated with attenuated intervention-induced body weight loss and increased carbohydrate intake in post-menopausal obese women. Appetite. 2013; **60**(1): 111-6.
201. Camma C, Giunta M, Chemello L, Alberti A, Toyoda H, Trepo C, et al. Chronic hepatitis C: interferon retreatment of relapsers. A meta-analysis of individual patient data. European Concerted Action on Viral Hepatitis (EUROHEP). Hepatology. 1999; **30**(3): 801-7.
202. Cammà C, Giunta M, Chemello L, Alberti A, Toyoda H, Trepo C, et al. Chronic hepatitis C: interferon retreatment of relapsers. A meta-analysis of individual patient data. European Concerted Action on Viral Hepatitis (EUROHEP). Hepatology (Baltimore, Md). 1999; **30**(3): 801-7.
203. Capers PL, Hyacinth HI, Cue S, Chappa P, Vikulina T, Roser-Page S, et al. Body composition and grip strength are improved in transgenic sickle mice fed a high-protein diet. J Nutr Sci. 2015; **4**: e6.
204. Capers PL, Hyacinth HI, Cue S, Chappa P, Vikulina T, Roser-Page S, et al. Body composition and grip strength are improved in transgenic sickle mice fed a high-protein diet. Journal Of Nutritional Science. 2015; **4**: e6-e.
205. Capozzo MA, Schillani G, Aguglia E, De Vanna M, Grassi L, Conte MA, et al.

Serotonin transporter 5-HTTLPR polymorphism and response to citalopram in terminally ill cancer patients: report of twenty-one cases. Tumori. 2009; **95**(4): 479-83.
206. Capozzo MA, Schillani G, Aguglia E, De Vanna M, Grassi L, Conte MA, et al. Serotonin transporter 5-HTTLPR polymorphism and response to citalopram in terminally ill cancer patients: report of twenty-one cases. Tumori. 2009; **95**(4): 479-83.
207. Carey GB. The swine as a model for studying exercise-induced changes in lipid metabolism. Med Sci Sports Exerc. 1997; **29**(11): 1437-43.
208. Carey GB. The swine as a model for studying exercise-induced changes in lipid metabolism. Medicine And Science In Sports And Exercise. 1997; **29**(11): 1437-43.
209. Carreras I, Aytan N, Mellott T, Choi JK, Lehar M, Crabtree L, et al. Anxiety, neuroinflammation, cholinergic and GABAergic abnormalities are early markers of Gulf War illness in a mouse model of the disease. Brain Res. 2018; **1681**: 34-43.
210. Carreras I, Aytan N, Mellott T, Choi J-K, Lehar M, Crabtree L, et al. Anxiety, neuroinflammation, cholinergic and GABAergic abnormalities are early markers of Gulf War illness in a mouse model of the disease. Brain Research. 2018; **1681**: 34-43.
211. Carrillo JA, Herraiz AG, Ramos SI, Benitez J. Effects of caffeine withdrawal from the diet on the metabolism of clozapine in schizophrenic patients. J Clin Psychopharmacol. 1998; **18**(4): 311-6.
212. Carrillo JA, Herraiz AG, Ramos SI, Benítez J. Effects of caffeine withdrawal from the diet on the metabolism of clozapine in schizophrenic patients. Journal Of Clinical Psychopharmacology. 1998; **18**(4): 311-6.
213. Carrion JA, Gonzalez-Colominas E, Garcia-Retortillo M, Canete N, Cirera I, Coll S, et al. A multidisciplinary support programme increases the efficiency of pegylated interferon alfa-2a and ribavirin in hepatitis C. J Hepatol. 2013; **59**(5): 926-33.
214. Carrión JA, Gonzalez-Colominas E, García-Retortillo M, Cañete N, Cirera I, Coll S, et al. A multidisciplinary support programme increases the efficiency of pegylated interferon alfa-2a and ribavirin in hepatitis C. Journal Of Hepatology. 2013; **59**(5): 926-33.
215. Carter NJ, McCormack PL. Duloxetine: a review of its use in the treatment of generalized anxiety disorder. CNS Drugs. 2009; **23**(6): 523-41.
216. Carter NJ, McCormack PL. Duloxetine: a review of its use in the treatment of generalized anxiety disorder. CNS Drugs. 2009; **23**(6): 523-41.
217. Casanello P, Krause BJ, Castro-Rodriguez JA, Uauy R. [Epigenetics and obesity]. Rev Chil Pediatr. 2016; **87**(5): 335-42.
218. Casanello P, Krause BJ, Castro-Rodríguez JA, Uauy R. [Epigenetics and obesity]. Revista Chilena De Pediatria. 2016; **87**(5): 335-42.
219. Casarotto PC, de Bortoli VC, Correa FM, Resstel LB, Zangrossi H, Jr. Panicolytic-like effect of BDNF in the rat dorsal periaqueductal grey matter: the role of 5-HT and GABA. Int J Neuropsychopharmacol. 2010; **13**(5): 573-82.
220. Casarotto PC, de Bortoli VC, Corrêa FMdA, Resstel LBM, Zangrossi H, Jr. Panicolytic-like effect of BDNF in the rat dorsal periaqueductal grey matter: the role of 5-HT and GABA. The International Journal Of Neuropsychopharmacology. 2010; **13**(5): 573-82.
221. Castren E, Kojima M. Brain-derived neurotrophic factor in mood disorders and antidepressant treatments. Neurobiol Dis. 2017; **97**(Pt B): 119-26.

222. Castrén E, Kojima M. Brain-derived neurotrophic factor in mood disorders and antidepressant treatments. Neurobiology Of Disease. 2017; **97**(Pt B): 119-26.
223. Cavalcante LN, Lyra AC. Predictive factors associated with hepatitis C antiviral therapy response. World J Hepatol. 2015; **7**(12): 1617-31.
224. Cavalcante LN, Lyra AC. Predictive factors associated with hepatitis C antiviral therapy response. World Journal Of Hepatology. 2015; **7**(12): 1617-31.
225. Cazorla M, Premont J, Mann A, Girard N, Kellendonk C, Rognan D. Identification of a low-molecular weight TrkB antagonist with anxiolytic and antidepressant activity in mice. J Clin Invest. 2011; **121**(5): 1846-57.
226. Cazorla M, Prémont J, Mann A, Girard N, Kellendonk C, Rognan D. Identification of a low-molecular weight TrkB antagonist with anxiolytic and antidepressant activity in mice. The Journal Of Clinical Investigation. 2011; **121**(5): 1846-57.
227. Cervilla JA, Rivera M, Molina E, Torres-Gonzalez F, Bellon JA, Moreno B, et al. The 5-HTTLPR s/s genotype at the serotonin transporter gene (SLC6A4) increases the risk for depression in a large cohort of primary care attendees: the PREDICT-gene study. Am J Med Genet B Neuropsychiatr Genet. 2006; **141B**(8): 912-7.
228. Cervilla JA, Rivera M, Molina E, Torres-González F, Bellón JA, Moreno B, et al. The 5-HTTLPR s/s genotype at the serotonin transporter gene (SLC6A4) increases the risk for depression in a large cohort of primary care attendees: the PREDICT-gene study. American Journal Of Medical Genetics Part B, Neuropsychiatric Genetics: The Official Publication Of The International Society Of Psychiatric Genetics. 2006; **141B**(8): 912-7.
229. Chagnon YC, Merette C, Bouchard RH, Emond C, Roy MA, Maziade M. A genome wide linkage study of obesity as secondary effect of antipsychotics in multigenerational families of eastern Quebec affected by psychoses. Mol Psychiatry. 2004; **9**(12): 1067-74.
230. Chagnon YC, Mérette C, Bouchard RH, Emond C, Roy MA, Maziade M. A genome wide linkage study of obesity as secondary effect of antipsychotics in multigenerational families of eastern Quebec affected by psychoses. Molecular Psychiatry. 2004; **9**(12): 1067-74.
231. Chakraborty R, Little MP, Sankaranarayanan K. Cancer predisposition, radiosensitivity and the risk of radiation-induced cancers. III. Effects of incomplete penetrance and dose-dependent radiosensitivity on cancer risks in populations. Radiat Res. 1997; **147**(3): 309-20.
232. Chakraborty R, Little MP, Sankaranarayanan K. Cancer predisposition, radiosensitivity and the risk of radiation-induced cancers. III. Effects of incomplete penetrance and dose-dependent radiosensitivity on cancer risks in populations. Radiation Research. 1997; **147**(3): 309-20.
233. Chakravarthy MV, Booth FW. Eating, exercise, and "thrifty" genotypes: connecting the dots toward an evolutionary understanding of modern chronic diseases. J Appl Physiol (1985). 2004; **96**(1): 3-10.
234. Chakravarthy MV, Booth FW. Eating, exercise, and "thrifty" genotypes: connecting the dots toward an evolutionary understanding of modern chronic diseases. Journal Of Applied Physiology (Bethesda, Md: 1985). 2004; **96**(1): 3-10.
235. Chandra Sekhar Y, Phani Kumar G, Anilakumar KR. Terminalia arjuna bark

extract attenuates picrotoxin-induced behavioral changes by activation of serotonergic, dopaminergic, GABAergic and antioxidant systems. Chin J Nat Med. 2017; **15**(8): 584-96.
236. Chandra Sekhar Y, Phani Kumar G, Anilakumar KR. Terminalia arjuna bark extract attenuates picrotoxin-induced behavioral changes by activation of serotonergic, dopaminergic, GABAergic and antioxidant systems. Chinese Journal Of Natural Medicines. 2017; **15**(8): 584-96.
237. Chang HA, Wang YH, Tung CS, Yeh CB, Liu YP. 7,8-Dihydroxyflavone, a Tropomyosin-Kinase Related Receptor B Agonist, Produces Fast-Onset Antidepressant-Like Effects in Rats Exposed to Chronic Mild Stress. Psychiatry Investig. 2016; **13**(5): 531-40.
238. Chang H-A, Wang Y-H, Tung C-S, Yeh C-B, Liu Y-P. 7,8-Dihydroxyflavone, a Tropomyosin-Kinase Related Receptor B Agonist, Produces Fast-Onset Antidepressant-Like Effects in Rats Exposed to Chronic Mild Stress. Psychiatry Investigation. 2016; **13**(5): 531-40.
239. Chapko MK, Dominitz JA. Cost-effectiveness of growth factors during hepatitis C anti-viral therapy. Aliment Pharmacol Ther. 2006; **24**(7): 1067-77.
240. Chapko MK, Dominitz JA. Cost-effectiveness of growth factors during hepatitis C anti-viral therapy. Alimentary Pharmacology & Therapeutics. 2006; **24**(7): 1067-77.
241. Chaudhari K, Wong JM, Vann PH, Sumien N. Exercise, but not antioxidants, reversed ApoE4-associated motor impairments in adult GFAP-ApoE mice. Behav Brain Res. 2016; **305**: 37-45.
242. Chaudhari K, Wong JM, Vann PH, Sumien N. Exercise, but not antioxidants, reversed ApoE4-associated motor impairments in adult GFAP-ApoE mice. Behavioural Brain Research. 2016; **305**: 37-45.
243. Chedraui P, Perez-Lopez FR. Nutrition and health during mid-life: searching for solutions and meeting challenges for the aging population. Climacteric. 2013; **16 Suppl 1**: 85-95.
244. Chedraui P, Pérez-López FR. Nutrition and health during mid-life: searching for solutions and meeting challenges for the aging population. Climacteric: The Journal Of The International Menopause Society. 2013; **16 Suppl 1**: 85-95.
245. Chen J, Yu J, Liu Y, Zhang L, Zhang J. BDNF Val66Met, stress, and positive mothering: Differential susceptibility model of adolescent trait anxiety. J Anxiety Disord. 2015; **34**: 68-75.
246. Chen J, Yu J, Liu Y, Zhang L, Zhang J. BDNF Val66Met, stress, and positive mothering: Differential susceptibility model of adolescent trait anxiety. Journal Of Anxiety Disorders. 2015; **34**: 68-75.
247. Chen Y, Zhu LL, Zhou Q. Effects of drug pharmacokinetic/pharmacodynamic properties, characteristics of medication use, and relevant pharmacological interventions on fall risk in elderly patients. Ther Clin Risk Manag. 2014; **10**: 437-48.
248. Chen Y, Zhu L-L, Zhou Q. Effects of drug pharmacokinetic/pharmacodynamic properties, characteristics of medication use, and relevant pharmacological interventions on fall risk in elderly patients. Therapeutics And Clinical Risk Management. 2014; **10**: 437-48.
249. Cherif A, Roelands B, Meeusen R, Chamari K. Effects of Intermittent Fasting,

Caloric Restriction, and Ramadan Intermittent Fasting on Cognitive Performance at Rest and During Exercise in Adults. Sports Med. 2016; **46**(1): 35-47.
250. Cherif A, Roelands B, Meeusen R, Chamari K. Effects of Intermittent Fasting, Caloric Restriction, and Ramadan Intermittent Fasting on Cognitive Performance at Rest and During Exercise in Adults. Sports Medicine (Auckland, NZ). 2016; **46**(1): 35-47.
251. Childs E, Hohoff C, Deckert J, Xu K, Badner J, de Wit H. Association between ADORA2A and DRD2 polymorphisms and caffeine-induced anxiety. Neuropsychopharmacology: Official Publication Of The American College Of Neuropsychopharmacology. 2008; **33**(12): 2791-800.
252. Cho NH, Cho AK, Kim HK, Kim JB, Lee KE, Kim SS, et al. Carbohydrate Composition Associated with the 2-Year Incidence of Metabolic Syndrome in Korean Adults. Clin Nutr Res. 2017; **6**(2): 122-9.
253. Cho NH, Cho AK, Kim HK, Kim JB, Lee KE, Kim SS, et al. Carbohydrate Composition Associated with the 2-Year Incidence of Metabolic Syndrome in Korean Adults. Clinical Nutrition Research. 2017; **6**(2): 122-9.
254. Choi JS, Kim W, Sohn BK, Lee JY, Jung HY, Oh S, et al. Association of Changes in Mood Status and Psychosocial Well-Being with Depression During Interferon-Based Treatment for Hepatitis C. Psychiatry Investig. 2017; **14**(3): 314-24.
255. Choi J-S, Kim W, Sohn BK, Lee J-Y, Jung HY, Oh S, et al. Association of Changes in Mood Status and Psychosocial Well-Being with Depression During Interferon-Based Treatment for Hepatitis C. Psychiatry Investigation. 2017; **14**(3): 314-24.
256. Choi MJ, Kang RH, Ham BJ, Jeong HY, Lee MS. Serotonin receptor 2A gene polymorphism (-1438A/G) and short-term treatment response to citalopram. Neuropsychobiology. 2005; **52**(3): 155-62.
257. Choi MJ, Kang RH, Lim SW, Oh KS, Lee MS. Brain-derived neurotrophic factor gene polymorphism (Val66Met) and citalopram response in major depressive disorder. Brain Res. 2006; **1118**(1): 176-82.
258. Choi M-J, Kang R-H, Ham B-J, Jeong H-Y, Lee M-S. Serotonin receptor 2A gene polymorphism (-1438A/G) and short-term treatment response to citalopram. Neuropsychobiology. 2005; **52**(3): 155-62.
259. Choi M-J, Kang R-H, Lim S-W, Oh K-S, Lee M-S. Brain-derived neurotrophic factor gene polymorphism (Val66Met) and citalopram response in major depressive disorder. Brain Research. 2006; **1118**(1): 176-82.
260. Chourbaji S, Brandwein C, Gass P. Altering BDNF expression by genetics and/or environment: impact for emotional and depression-like behaviour in laboratory mice. Neurosci Biobehav Rev. 2011; **35**(3): 599-611.
261. Chourbaji S, Brandwein C, Gass P. Altering BDNF expression by genetics and/or environment: impact for emotional and depression-like behaviour in laboratory mice. Neuroscience And Biobehavioral Reviews. 2011; **35**(3): 599-611.
262. Chow WH, Devesa SS. Contemporary epidemiology of renal cell cancer. Cancer J. 2008; **14**(5): 288-301.
263. Chow WH, Dong LM, Devesa SS. Epidemiology and risk factors for kidney cancer. Nat Rev Urol. 2010; **7**(5): 245-57.

264. Chow W-H, Devesa SS. Contemporary epidemiology of renal cell cancer. Cancer Journal (Sudbury, Mass). 2008; **14**(5): 288-301.
265. Chow W-H, Dong LM, Devesa SS. Epidemiology and risk factors for kidney cancer. Nature Reviews Urology. 2010; **7**(5): 245-57.
266. Christensen DH, Nicolaisen SK, Berencsi K, Beck-Nielsen H, Rungby J, Friborg S, et al. Danish Centre for Strategic Research in Type 2 Diabetes (DD2) project cohort of newly diagnosed patients with type 2 diabetes: a cohort profile. BMJ Open. 2018; **8**(4): e017273.
267. Christensen DH, Nicolaisen SK, Berencsi K, Beck-Nielsen H, Rungby J, Friborg S, et al. Danish Centre for Strategic Research in Type 2 Diabetes (DD2) project cohort of newly diagnosed patients with type 2 diabetes: a cohort profile. BMJ Open. 2018; **8**(4): e017273-e.
268. Chui DH, Marcellino M, Marotta F, Sweed H, Solimene U, Vignali AI, et al. A double-blind, rct testing beneficial modulation of BDNF in middle-aged, life style-stressed subjects: a clue to brain protection? J Clin Diagn Res. 2014; **8**(11): MC01-6.
269. Chui DH, Marcellino M, Marotta F, Sweed H, Solimene U, Vignali AI, et al. A double-blind, rct testing beneficial modulation of BDNF in middle-aged, life style-stressed subjects: a clue to brain protection? Journal Of Clinical And Diagnostic Research: JCDR. 2014; **8**(11): MC01-MC6.
270. Chung CS, Lee YC, Liou JM, Wang CP, Ko JY, Lee JM, et al. Tag single nucleotide polymorphisms of alcohol-metabolizing enzymes modify the risk of upper aerodigestive tract cancers: HapMap database analysis. Dis Esophagus. 2014; **27**(5): 493-503.
271. Chung CS, Lee YC, Liou JM, Wang CP, Ko JY, Lee JM, et al. Tag single nucleotide polymorphisms of alcohol-metabolizing enzymes modify the risk of upper aerodigestive tract cancers: HapMap database analysis. Diseases Of The Esophagus: Official Journal Of The International Society For Diseases Of The Esophagus. 2014; **27**(5): 493-503.
272. Cicek IE, Cicek E, Kayhan F, Uguz F, Erayman I, Kurban S, et al. The roles of BDNF, S100B, and oxidative stress in interferon-induced depression and the effect of antidepressant treatment in patients with chronic viral hepatitis: a prospective study. J Psychosom Res. 2014; **76**(3): 227-32.
273. Cicek IE, Cicek E, Kayhan F, Uguz F, Erayman I, Kurban S, et al. The roles of BDNF, S100B, and oxidative stress in interferon-induced depression and the effect of antidepressant treatment in patients with chronic viral hepatitis: a prospective study. Journal Of Psychosomatic Research. 2014; **76**(3): 227-32.
274. Cimino S, Cerniglia L, Ballarotto G, Marzilli E, Pascale E, D'Addario C, et al. DNA Methylation at the DAT Promoter and Risk for Psychopathology: Intergenerational Transmission between School-Age Youths and Their Parents in a Community Sample. Front Psychiatry. 2017; **8**: 303.
275. Cimino S, Cerniglia L, Ballarotto G, Marzilli E, Pascale E, D'Addario C, et al. DNA Methylation at the DAT Promoter and Risk for Psychopathology: Intergenerational Transmission between School-Age Youths and Their Parents in a Community Sample. Frontiers In Psychiatry. 2018; **8**: 303-.
276. Ciuculete DM, Bostrom AE, Tuunainen AK, Sohrabi F, Kular L, Jagodic M, et al.

Changes in methylation within the STK32B promoter are associated with an increased risk for generalized anxiety disorder in adolescents. J Psychiatr Res. 2018; **102**: 44-51.
277. Ciuculete DM, Boström AE, Tuunainen A-K, Sohrabi F, Kular L, Jagodic M, et al. Changes in methylation within the STK32B promoter are associated with an increased risk for generalized anxiety disorder in adolescents. Journal Of Psychiatric Research. 2018; **102**: 44-51.
278. Clarkson PM, Devaney JM, Gordish-Dressman H, Thompson PD, Hubal MJ, Urso M, et al. ACTN3 genotype is associated with increases in muscle strength in response to resistance training in women. J Appl Physiol (1985). 2005; **99**(1): 154-63.
279. Clarkson PM, Devaney JM, Gordish-Dressman H, Thompson PD, Hubal MJ, Urso M, et al. ACTN3 genotype is associated with increases in muscle strength in response to resistance training in women. Journal Of Applied Physiology (Bethesda, Md: 1985). 2005; **99**(1): 154-63.
280. Cohen H, Liu T, Kozlovsky N, Kaplan Z, Zohar J, Mathe AA. The neuropeptide Y (NPY)-ergic system is associated with behavioral resilience to stress exposure in an animal model of post-traumatic stress disorder. Neuropsychopharmacology. 2012; **37**(2): 350-63.
281. Cohen H, Liu T, Kozlovsky N, Kaplan Z, Zohar J, Mathé AA. The neuropeptide Y (NPY)-ergic system is associated with behavioral resilience to stress exposure in an animal model of post-traumatic stress disorder. Neuropsychopharmacology: Official Publication Of The American College Of Neuropsychopharmacology. 2012; **37**(2): 350-63.
282. Cole SA, Butte NF, Voruganti VS, Cai G, Haack K, Kent JW, Jr., et al. Evidence that multiple genetic variants of MC4R play a functional role in the regulation of energy expenditure and appetite in Hispanic children. Am J Clin Nutr. 2010; **91**(1): 191-9.
283. Cole SA, Butte NF, Voruganti VS, Cai G, Haack K, Kent JW, Jr., et al. Evidence that multiple genetic variants of MC4R play a functional role in the regulation of energy expenditure and appetite in Hispanic children. The American Journal Of Clinical Nutrition. 2010; **91**(1): 191-9.
284. Coleman JR, Lester KJ, Keers R, Roberts S, Curtis C, Arendt K, et al. Genome-wide association study of response to cognitive-behavioural therapy in children with anxiety disorders. Br J Psychiatry. 2016; **209**(3): 236-43.
285. Coleman JR, Lester KJ, Roberts S, Keers R, Lee SH, De Jong S, et al. Separate and combined effects of genetic variants and pre-treatment whole blood gene expression on response to exposure-based cognitive behavioural therapy for anxiety disorders. World J Biol Psychiatry. 2017; **18**(3): 215-26.
286. Coleman JRI, Lester KJ, Keers R, Roberts S, Curtis C, Arendt K, et al. Genome-wide association study of response to cognitive-behavioural therapy in children with anxiety disorders. The British Journal Of Psychiatry: The Journal Of Mental Science. 2016; **209**(3): 236-43.
287. Coleman JRI, Lester KJ, Roberts S, Keers R, Lee SH, De Jong S, et al. Separate and combined effects of genetic variants and pre-treatment whole blood gene expression on response to exposure-based cognitive behavioural therapy for anxiety disorders. The World Journal Of Biological Psychiatry: The Official Journal Of The World Federation Of Societies Of Biological Psychiatry. 2017; **18**(3): 215-26.

288. Colzato LS, Van der Does AJ, Kouwenhoven C, Elzinga BM, Hommel B. BDNF Val66Met polymorphism is associated with higher anticipatory cortisol stress response, anxiety, and alcohol consumption in healthy adults. Psychoneuroendocrinology. 2011; **36**(10): 1562-9.
289. Colzato LS, Van der Does AJW, Kouwenhoven C, Elzinga BM, Hommel B. BDNF Val66Met polymorphism is associated with higher anticipatory cortisol stress response, anxiety, and alcohol consumption in healthy adults. Psychoneuroendocrinology. 2011; **36**(10): 1562-9.
290. Cong WN, Wang R, Cai H, Daimon CM, Scheibye-Knudsen M, Bohr VA, et al. Long-term artificial sweetener acesulfame potassium treatment alters neurometabolic functions in C57BL/6J mice. PLoS One. 2013; **8**(8): e70257.
291. Cong W-n, Wang R, Cai H, Daimon CM, Scheibye-Knudsen M, Bohr VA, et al. Long-term artificial sweetener acesulfame potassium treatment alters neurometabolic functions in C57BL/6J mice. PLoS One. 2013; **8**(8): e70257-e.
292. Conlisk AJ, Stein AD, Schroeder DG, Torun B, Grajeda R, Martorell R. Determinants of fasting glucose in young Guatemalan adults. Ethn Dis. 2001; **11**(4): 585-97.
293. Conlisk AJ, Stein AD, Schroeder DG, Torun B, Grajeda R, Martorell R. Determinants of fasting glucose in young Guatemalan adults. Ethnicity & Disease. 2001; **11**(4): 585-97.
294. Conway CC, Hammen C, Brennan PA, Lind PA, Najman JM. Interaction of chronic stress with serotonin transporter and catechol-O-methyltransferase polymorphisms in predicting youth depression. Depress Anxiety. 2010; **27**(8): 737-45.
295. Conway CC, Hammen C, Brennan PA, Lind PA, Najman JM. Interaction of chronic stress with serotonin transporter and catechol-O-methyltransferase polymorphisms in predicting youth depression. Depression And Anxiety. 2010; **27**(8): 737-45.
296. Cookson WO. Disease taxonomy--polygenic. Br Med Bull. 1999; **55**(2): 358-65.
297. Cookson WO. Disease taxonomy--polygenic. British Medical Bulletin. 1999; **55**(2): 358-65.
298. Cordain L, Gotshall RW, Eaton SB, Eaton SB, 3rd. Physical activity, energy expenditure and fitness: an evolutionary perspective. Int J Sports Med. 1998; **19**(5): 328-35.
299. Cordain L, Gotshall RW, Eaton SB, Eaton SB, 3rd. Physical activity, energy expenditure and fitness: an evolutionary perspective. International Journal Of Sports Medicine. 1998; **19**(5): 328-35.
300. Corella D, Arregui M, Coltell O, Portoles O, Guillem-Saiz P, Carrasco P, et al. Association of the LCT-13910C>T polymorphism with obesity and its modulation by dairy products in a Mediterranean population. Obesity (Silver Spring). 2011; **19**(8): 1707-14.
301. Corella D, Arregui M, Coltell O, Portolés O, Guillem-Sáiz P, Carrasco P, et al. Association of the LCT-13910C>T polymorphism with obesity and its modulation by dairy products in a Mediterranean population. Obesity (Silver Spring, Md). 2011; **19**(8): 1707-14.
302. Corella D, Guillen M, Portoles O, Sabater A, Cortina S, Folch J, et al.

[Apolipoprotein E gene polymorphism and risk of hypercholesterolemia: a case control study in a working population of Valencia]. Med Clin (Barc). 2000; **115**(5): 170-5.
303. Corella D, Guillén M, Portolés O, Sabater A, Cortina S, Folch J, et al. [Apolipoprotein E gene polymorphism and risk of hypercholesterolemia: a case control study in a working population of Valencia]. Medicina Clinica. 2000; **115**(5): 170-5.
304. Corella D, Guillen M, Saiz C, Portoles O, Sabater A, Cortina S, et al. Environmental factors modulate the effect of the APOE genetic polymorphism on plasma lipid concentrations: ecogenetic studies in a Mediterranean Spanish population. Metabolism. 2001; **50**(8): 936-44.
305. Corella D, Guillén M, Sáiz C, Portolés O, Sabater A, Cortina S, et al. Environmental factors modulate the effect of the APOE genetic polymorphism on plasma lipid concentrations: ecogenetic studies in a Mediterranean Spanish population. Metabolism: Clinical And Experimental. 2001; **50**(8): 936-44.
306. Corella D, Ordovas JM. Can genotype be used to tailor treatment of obesity? State of the art and guidelines for future studies and applications. Minerva Endocrinol. 2013; **38**(3): 219-35.
307. Corella D, Ordovas JM. Basic Concepts in Molecular Biology Related to Genetics and Epigenetics. Rev Esp Cardiol (Engl Ed). 2017; **70**(9): 744-53.
308. Corella D, Ordovas JM. Basic Concepts in Molecular Biology Related to Genetics and Epigenetics. Revista Espanola De Cardiologia (English Ed). 2017; **70**(9): 744-53.
309. Corella D, Ordovás JM. Can genotype be used to tailor treatment of obesity? State of the art and guidelines for future studies and applications. Minerva Endocrinologica. 2013; **38**(3): 219-35.
310. Coric V, Feldman HH, Oren DA, Shekhar A, Pultz J, Dockens RC, et al. Multicenter, randomized, double-blind, active comparator and placebo-controlled trial of a corticotropin-releasing factor receptor-1 antagonist in generalized anxiety disorder. Depress Anxiety. 2010; **27**(5): 417-25.
311. Coric V, Feldman HH, Oren DA, Shekhar A, Pultz J, Dockens RC, et al. Multicenter, randomized, double-blind, active comparator and placebo-controlled trial of a corticotropin-releasing factor receptor-1 antagonist in generalized anxiety disorder. Depression And Anxiety. 2010; **27**(5): 417-25.
312. Cornelis MC, Monda KL, Yu K, Paynter N, Azzato EM, Bennett SN, et al. Genome-wide meta-analysis identifies regions on 7p21 (AHR) and 15q24 (CYP1A2) as determinants of habitual caffeine consumption. PLoS Genet. 2011; **7**(4): e1002033.
313. Corominas-Roso M, Roncero C, Eiroa-Orosa FJ, Gonzalvo B, Grau-Lopez L, Ribases M, et al. Brain-derived neurotrophic factor serum levels in cocaine-dependent patients during early abstinence. Eur Neuropsychopharmacol. 2013; **23**(9): 1078-84.
314. Corominas-Roso M, Roncero C, Eiroa-Orosa FJ, Gonzalvo B, Grau-Lopez L, Ribases M, et al. Brain-derived neurotrophic factor serum levels in cocaine-dependent patients during early abstinence. European Neuropsychopharmacology: The Journal Of The European College Of Neuropsychopharmacology. 2013; **23**(9): 1078-84.
315. Correa-Rodriguez M, Schmidt-RioValle J, Rueda-Medina B. The rs3736228 polymorphism in the LRP5 gene is associated with calcaneal ultrasound parameter but not with body composition in a cohort of young Caucasian adults. J Bone Miner Metab. 2017; **35**(6): 694-700.

316. Correa-Rodríguez M, Schmidt-RioValle J, Rueda-Medina B. The rs3736228 polymorphism in the LRP5 gene is associated with calcaneal ultrasound parameter but not with body composition in a cohort of young Caucasian adults. Journal Of Bone And Mineral Metabolism. 2017; **35**(6): 694-700.
317. Corripio R, Gonzalez-Clemente JM, Jacobo PS, Silvia N, Lluis G, Joan V, et al. Plasma brain-derived neurotrophic factor in prepubertal obese children: results from a 2-year lifestyle intervention programme. Clin Endocrinol (Oxf). 2012; **77**(5): 715-20.
318. Corripio R, Gónzalez-Clemente J-M, Jacobo P-S, Silvia N, Lluis G, Joan V, et al. Plasma brain-derived neurotrophic factor in prepubertal obese children: results from a 2-year lifestyle intervention programme. Clinical Endocrinology. 2012; **77**(5): 715-20.
319. Crous-Bou M, Harrington LB, Kabrhel C. Environmental and Genetic Risk Factors Associated with Venous Thromboembolism. Semin Thromb Hemost. 2016; **42**(8): 808-20.
320. Crous-Bou M, Harrington LB, Kabrhel C. Environmental and Genetic Risk Factors Associated with Venous Thromboembolism. Seminars In Thrombosis And Hemostasis. 2016; **42**(8): 808-20.
321. Cunha MP, Pazini FL, Lieberknecht V, Budni J, Oliveira A, Rosa JM, et al. MPP(+)-Lesioned Mice: an Experimental Model of Motor, Emotional, Memory/Learning, and Striatal Neurochemical Dysfunctions. Mol Neurobiol. 2017; **54**(8): 6356-77.
322. Cunha MP, Pazini FL, Lieberknecht V, Budni J, Oliveira Á, Rosa JM, et al. MPP+-Lesioned Mice: an Experimental Model of Motor, Emotional, Memory/Learning, and Striatal Neurochemical Dysfunctions. Molecular Neurobiology. 2017; **54**(8): 6356-77.
323. Dahabreh IJ, Moorthy D, Lamont JL, Chen ML, Kent DM, Lau J. 2013.
324. Dai S, Dieterich K, Jaeger M, Wuyam B, Jouk PS, Perennou D. Disability in adults with arthrogryposis is severe, partly invisible, and varies by genotype. Neurology. 2018.
325. Dai S, Dieterich K, Jaeger M, Wuyam B, Jouk P-S, Pérennou D. Disability in adults with arthrogryposis is severe, partly invisible, and varies by genotype. Neurology. 2018; **90**(18): e1596-e604.
326. Daily JW, Park S. Interaction of BDNF rs6265 variants and energy and protein intake in the risk for glucose intolerance and type 2 diabetes in middle-aged adults. Nutrition. 2017; **33**: 187-94.
327. Daily JW, Park S. Interaction of BDNF rs6265 variants and energy and protein intake in the risk for glucose intolerance and type 2 diabetes in middle-aged adults. Nutrition (Burbank, Los Angeles County, Calif). 2017; **33**: 187-94.
328. Dallongeville J, Delcroix AG, Wagner A, Ducimetiere P, Ruidavets JB, Arveiler D, et al. The APOA4 Thr347->Ser347 polymorphism is not a major risk factor of obesity. Obes Res. 2005; **13**(12): 2132-8.
329. Dallongeville J, Delcroix A-G, Wagner A, Ducimetière P, Ruidavets J-B, Arveiler D, et al. The APOA4 Thr347->Ser347 polymorphism is not a major risk factor of obesity. Obesity Research. 2005; **13**(12): 2132-8.
330. Daly RM, Gianoudis J, Prosser M, Kidgell D, Ellis KA, O'Connell S, et al. The effects of a protein enriched diet with lean red meat combined with a multi-modal exercise program on muscle and cognitive health and function in older adults: study protocol for a randomised controlled trial. Trials. 2015; **16**: 339.

331. Daly RM, Gianoudis J, Prosser M, Kidgell D, Ellis KA, O'Connell S, et al. The effects of a protein enriched diet with lean red meat combined with a multi-modal exercise program on muscle and cognitive health and function in older adults: study protocol for a randomised controlled trial. Trials. 2015; **16**: 339-.
332. Damcott CM, Sack P, Shuldiner AR. The genetics of obesity. Endocrinol Metab Clin North Am. 2003; **32**(4): 761-86.
333. Damcott CM, Sack P, Shuldiner AR. The genetics of obesity. Endocrinology And Metabolism Clinics Of North America. 2003; **32**(4): 761-86.
334. Damirchi A, Tehrani BS, Alamdari KA, Babaei P. Influence of aerobic training and detraining on serum BDNF, insulin resistance, and metabolic risk factors in middle-aged men diagnosed with metabolic syndrome. Clin J Sport Med. 2014; **24**(6): 513-8.
335. Damirchi A, Tehrani BS, Alamdari KA, Babaei P. Influence of aerobic training and detraining on serum BDNF, insulin resistance, and metabolic risk factors in middle-aged men diagnosed with metabolic syndrome. Clinical Journal Of Sport Medicine: Official Journal Of The Canadian Academy Of Sport Medicine. 2014; **24**(6): 513-8.
336. Damkier P, Hansen LL, Brosen K. Effect of diclofenac, disulfiram, itraconazole, grapefruit juice and erythromycin on the pharmacokinetics of quinidine. Br J Clin Pharmacol. 1999; **48**(6): 829-38.
337. Damkier P, Hansen LL, Brosen K. Effect of diclofenac, disulfiram, itraconazole, grapefruit juice and erythromycin on the pharmacokinetics of quinidine. British Journal Of Clinical Pharmacology. 1999; **48**(6): 829-38.
338. Damsgaard CT, Lauritzen L, Hauger H, Vuholm S, Teisen MN, Ritz C, et al. Effects of oily fish intake on cardiovascular risk markers, cognitive function, and behavior in school-aged children: study protocol for a randomized controlled trial. Trials. 2016; **17**(1): 510.
339. Damsgaard CT, Lauritzen L, Hauger H, Vuholm S, Teisen MN, Ritz C, et al. Effects of oily fish intake on cardiovascular risk markers, cognitive function, and behavior in school-aged children: study protocol for a randomized controlled trial. Trials. 2016; **17**(1): 510-.
340. Dankner R, Chetrit A, Dror GK, Sela B-A. Physical activity is inversely associated with total homocysteine levels, independent of C677T MTHFR genotype and plasma B vitamins. Age (Dordrecht, Netherlands). 2007; **29**(4): 219-27.
341. Dankner R, Chetrit A, Dror GK, Sela BA. Physical activity is inversely associated with total homocysteine levels, independent of C677T MTHFR genotype and plasma B vitamins. Age (Dordr). 2007; **29**(4): 219-27.
342. Data SA, Roltsch MH, Hand B, Ferrell RE, Park JJ, Brown MD. eNOS T-786C genotype, physical activity, and peak forearm blood flow in females. Med Sci Sports Exerc. 2003; **35**(12): 1991-7.
343. Data SA, Roltsch MH, Hand B, Ferrell RE, Park J-j, Brown MD. eNOS T-786C genotype, physical activity, and peak forearm blood flow in females. Medicine And Science In Sports And Exercise. 2003; **35**(12): 1991-7.
344. Dausset J. [Predictive medicine and its ethics]. Pathol Biol (Paris). 1997; **45**(3): 199-204.
345. Dausset J. [Predictive medicine and its ethics]. Pathologie-Biologie. 1997; **45**(3): 199-204.

346. David SP, Murthy NV, Rabiner EA, Munafo MR, Johnstone EC, Jacob R, et al. A functional genetic variation of the serotonin (5-HT) transporter affects 5-HT1A receptor binding in humans. J Neurosci. 2005; **25**(10): 2586-90.
347. David SP, Murthy NV, Rabiner EA, Munafó MR, Johnstone EC, Jacob R, et al. A functional genetic variation of the serotonin (5-HT) transporter affects 5-HT1A receptor binding in humans. The Journal Of Neuroscience: The Official Journal Of The Society For Neuroscience. 2005; **25**(10): 2586-90.
348. David SP, Niaura R, Papandonatos GD, Shadel WG, Burkholder GJ, Britt DM, et al. Does the DRD2-Taq1 A polymorphism influence treatment response to bupropion hydrochloride for reduction of the nicotine withdrawal syndrome? Nicotine Tob Res. 2003; **5**(6): 935-42.
349. David SP, Niaura R, Papandonatos GD, Shadel WG, Burkholder GJ, Britt DM, et al. Does the DRD2-Taq1 A polymorphism influence treatment response to bupropion hydrochloride for reduction of the nicotine withdrawal syndrome? Nicotine & Tobacco Research: Official Journal Of The Society For Research On Nicotine And Tobacco. 2003; **5**(6): 935-42.
350. De Jesus-Cortes H, Rajadhyaksha AM, Pieper AA. Cacna1c: Protecting young hippocampal neurons in the adult brain. Neurogenesis (Austin). 2016; **3**(1): e1231160.
351. De Jesús-Cortés H, Rajadhyaksha AM, Pieper AA. Cacna1c: Protecting young hippocampal neurons in the adult brain. Neurogenesis (Austin, Tex). 2016; **3**(1): e1231160-e.
352. de la Tremblaye PB, Linares NN, Schock S, Plamondon H. Activation of CRHR1 receptors regulates social and depressive-like behaviors and expression of BDNF and TrkB in mesocorticolimbic regions following global cerebral ischemia. Exp Neurol. 2016; **284**(Pt A): 84-97.
353. de la Tremblaye PB, Linares NN, Schock S, Plamondon H. Activation of CRHR1 receptors regulates social and depressive-like behaviors and expression of BDNF and TrkB in mesocorticolimbic regions following global cerebral ischemia. Experimental Neurology. 2016; **284**(Pt A): 84-97.
354. De Nicola AF, Labombarda F, Gonzalez Deniselle MC, Gonzalez SL, Garay L, Meyer M, et al. Progesterone neuroprotection in traumatic CNS injury and motoneuron degeneration. Front Neuroendocrinol. 2009; **30**(2): 173-87.
355. De Nicola AF, Labombarda F, Gonzalez Deniselle MC, Gonzalez SL, Garay L, Meyer M, et al. Progesterone neuroprotection in traumatic CNS injury and motoneuron degeneration. Frontiers In Neuroendocrinology. 2009; **30**(2): 173-87.
356. de Oliveira MR, da Rocha RF, Stertz L, Fries GR, de Oliveira DL, Kapczinski F, et al. Total and mitochondrial nitrosative stress, decreased brain-derived neurotrophic factor (BDNF) levels and glutamate uptake, and evidence of endoplasmic reticulum stress in the hippocampus of vitamin A-treated rats. Neurochem Res. 2011; **36**(3): 506-17.
357. de Oliveira MR, da Rocha RF, Stertz L, Fries GR, de Oliveira DL, Kapczinski F, et al. Total and mitochondrial nitrosative stress, decreased brain-derived neurotrophic factor (BDNF) levels and glutamate uptake, and evidence of endoplasmic reticulum stress in the hippocampus of vitamin A-treated rats. Neurochemical Research. 2011; **36**(3): 506-17.

358. de Paz B, Alperi-Lopez M, Ballina-Garcia FJ, Prado C, Mozo L, Gutierrez C, et al. Interleukin 10 and tumor necrosis factor-alpha genotypes in rheumatoid arthritis--association with clinical response to glucocorticoids. J Rheumatol. 2010; **37**(3): 503-11.
359. de Paz B, Alperi-López M, Ballina-García FJ, Prado C, Mozo L, Gutiérrez C, et al. Interleukin 10 and tumor necrosis factor-alpha genotypes in rheumatoid arthritis--association with clinical response to glucocorticoids. The Journal Of Rheumatology. 2010; **37**(3): 503-11.
360. de Vries JM, Kuperus E, Hoogeveen-Westerveld M, Kroos MA, Wens SC, Stok M, et al. Pompe disease in adulthood: effects of antibody formation on enzyme replacement therapy. Genet Med. 2017; **19**(1): 90-7.
361. de Vries JM, Kuperus E, Hoogeveen-Westerveld M, Kroos MA, Wens SCA, Stok M, et al. Pompe disease in adulthood: effects of antibody formation on enzyme replacement therapy. Genetics In Medicine: Official Journal Of The American College Of Medical Genetics. 2017; **19**(1): 90-7.
362. Dean L. Carisoprodol Therapy and CYP2C19 Genotype. 2012.
363. Dean L. Amitriptyline Therapy and CYP2D6 and CYP2C19 Genotype. 2012.
364. Dean L. Imipramine Therapy and CYP2D6 and CYP2C19 Genotype. 2012.
365. Dean L. Venlafaxine Therapy and CYP2D6 Genotype. 2012.
366. Dean L. Amitriptyline Therapy and CYP2D6 and CYP2C19 Genotype. Medical Genetics Summaries. 2012.
367. Dean L. Carisoprodol Therapy and CYP2C19 Genotype. Medical Genetics Summaries. 2012.
368. Dean L. Imipramine Therapy and CYP2D6 and CYP2C19 Genotype. Medical Genetics Summaries. 2012.
369. Dean L. Venlafaxine Therapy and CYP2D6 Genotype. Medical Genetics Summaries. 2012.
370. Dedoussis GV, Manios Y, Choumerianou DM, Yiannakouris N, Panagiotakos DB, Skenderi K, et al. The IL-6 gene G-174C polymorphism related to health indices in Greek primary school children. Obes Res. 2004; **12**(7): 1037-41.
371. Dedoussis GVZ, Manios Y, Choumerianou DM, Yiannakouris N, Panagiotakos DB, Skenderi K, et al. The IL-6 gene G-174C polymorphism related to health indices in Greek primary school children. Obesity Research. 2004; **12**(7): 1037-41.
372. Deng C, He X, Hsueh AJ. A single-nucleotide polymorphism of human neuropeptide s gene originated from Europe shows decreased bioactivity. PLoS One. 2013; **8**(12): e83009.
373. Deng C, He X, Hsueh AJW. A single-nucleotide polymorphism of human neuropeptide s gene originated from Europe shows decreased bioactivity. PLoS One. 2013; **8**(12): e83009-e.
374. Deng QF, Zhou CC, Su CX. Clinicopathological features and epidermal growth factor receptor mutations associated with epithelial-mesenchymal transition in non-small cell lung cancer. Respirology. 2009; **14**(3): 371-6.
375. Deng Q-F, Zhou C-C, Su C-X. Clinicopathological features and epidermal growth factor receptor mutations associated with epithelial-mesenchymal transition in non-small cell lung cancer. Respirology (Carlton, Vic). 2009; **14**(3): 371-6.
376. Dennison EM, Syddall HE, Jameson KA, Sayer AA, Gaunt TR, Rodriguez S, et

al. A study of relationships between single nucleotide polymorphisms from the growth hormone-insulin-like growth factor axis and bone mass: the Hertfordshire cohort study. J Rheumatol. 2009; **36**(7): 1520-6.
377. Dennison EM, Syddall HE, Jameson KA, Sayer AA, Gaunt TR, Rodriguez S, et al. A study of relationships between single nucleotide polymorphisms from the growth hormone-insulin-like growth factor axis and bone mass: the Hertfordshire cohort study. The Journal Of Rheumatology. 2009; **36**(7): 1520-6.
378. Desmeules J, Piguet V, Besson M, Chabert J, Rapiti E, Rebsamen M, et al. Psychological distress in fibromyalgia patients: a role for catechol-O-methyl-transferase Val158met polymorphism. Health Psychol. 2012; **31**(2): 242-9.
379. Desmeules J, Piguet V, Besson M, Chabert J, Rapiti E, Rebsamen M, et al. Psychological distress in fibromyalgia patients: a role for catechol-O-methyl-transferase Val158met polymorphism. Health Psychology: Official Journal Of The Division Of Health Psychology, American Psychological Association. 2012; **31**(2): 242-9.
380. Deterding K, Gruner N, Buggisch P, Galle PR, Spengler U, Hinrichsen H, et al. Symptoms of anxiety and depression are frequent in patients with acute hepatitis C and are not associated with disease severity. Eur J Gastroenterol Hepatol. 2016; **28**(2): 187-92.
381. Deterding K, Grüner N, Buggisch P, Galle PR, Spengler U, Hinrichsen H, et al. Symptoms of anxiety and depression are frequent in patients with acute hepatitis C and are not associated with disease severity. European Journal Of Gastroenterology & Hepatology. 2016; **28**(2): 187-92.
382. DeVane CL, Stowe ZN, Donovan JL, Newport DJ, Pennell PB, Ritchie JC, et al. Therapeutic drug monitoring of psychoactive drugs during pregnancy in the genomic era: challenges and opportunities. Journal Of Psychopharmacology (Oxford, England). 2006; **20**(4 Suppl): 54-9.
383. DeVane CL, Stowe ZN, Donovan JL, Newport DJ, Pennell PB, Ritchie JC, et al. Therapeutic drug monitoring of psychoactive drugs during pregnancy in the genomic era: challenges and opportunities. J Psychopharmacol. 2006; **20**(4 Suppl): 54-9.
384. Dhalwani NN, Zaccardi F, O'Donovan G, Carter P, Hamer M, Yates T, et al. Association Between Lifestyle Factors and the Incidence of Multimorbidity in an Older English Population. J Gerontol A Biol Sci Med Sci. 2017; **72**(4): 528-34.
385. Dhalwani NN, Zaccardi F, O'Donovan G, Carter P, Hamer M, Yates T, et al. Association Between Lifestyle Factors and the Incidence of Multimorbidity in an Older English Population. The Journals Of Gerontology Series A, Biological Sciences And Medical Sciences. 2017; **72**(4): 528-34.
386. D'Hulst C, De Geest N, Reeve SP, Van Dam D, De Deyn PP, Hassan BA, et al. Decreased expression of the GABAA receptor in fragile X syndrome. Brain Res. 2006; **1121**(1): 238-45.
387. D'Hulst C, De Geest N, Reeve SP, Van Dam D, De Deyn PP, Hassan BA, et al. Decreased expression of the GABAA receptor in fragile X syndrome. Brain Research. 2006; **1121**(1): 238-45.
388. Di Marco F, Guazzi M, Vicenzi M, Santus P, Cazzola M, Pappalettera M, et al. Effect of enalapril on exercise cardiopulmonary performance in chronic obstructive pulmonary disease: A pilot study. Pulm Pharmacol Ther. 2010; **23**(3): 159-64.

389. Di Marco F, Guazzi M, Vicenzi M, Santus P, Cazzola M, Pappalettera M, et al. Effect of enalapril on exercise cardiopulmonary performance in chronic obstructive pulmonary disease: A pilot study. Pulmonary Pharmacology & Therapeutics. 2010; **23**(3): 159-64.
390. di Nuzzo L, Orlando R, Tognoli C, Di Pietro P, Bertini G, Miele J, et al. Antidepressant activity of fingolimod in mice. Pharmacol Res Perspect. 2015; **3**(3): e00135.
391. di Nuzzo L, Orlando R, Tognoli C, Di Pietro P, Bertini G, Miele J, et al. Antidepressant activity of fingolimod in mice. Pharmacology Research & Perspectives. 2015; **3**(3): e00135-e.
392. Diane A, Pierce WD, Mangat R, Borthwick F, Nelson R, Russell JC, et al. Differential expression of hypothalamic, metabolic and inflammatory genes in response to short-term calorie restriction in juvenile obese- and lean-prone JCR rats. Nutr Diabetes. 2015; **5**: e178.
393. Diane A, Pierce WD, Mangat R, Borthwick F, Nelson R, Russell JC, et al. Differential expression of hypothalamic, metabolic and inflammatory genes in response to short-term calorie restriction in juvenile obese- and lean-prone JCR rats. Nutrition & Diabetes. 2015; **5**: e178-e.
394. Diemer J, Domschke K, Muhlberger A, Winter B, Zavorotnyy M, Notzon S, et al. Acute anxiolytic effects of quetiapine during virtual reality exposure--a double-blind placebo-controlled trial in patients with specific phobia. Eur Neuropsychopharmacol. 2013; **23**(11): 1551-60.
395. Diemer J, Domschke K, Mühlberger A, Winter B, Zavorotnyy M, Notzon S, et al. Acute anxiolytic effects of quetiapine during virtual reality exposure--a double-blind placebo-controlled trial in patients with specific phobia. European Neuropsychopharmacology: The Journal Of The European College Of Neuropsychopharmacology. 2013; **23**(11): 1551-60.
396. Do-Monte FH, Rodriguez-Romaguera J, Rosas-Vidal LE, Quirk GJ. Deep brain stimulation of the ventral striatum increases BDNF in the fear extinction circuit. Front Behav Neurosci. 2013; **7**: 102.
397. Do-Monte FH, Rodriguez-Romaguera J, Rosas-Vidal LE, Quirk GJ. Deep brain stimulation of the ventral striatum increases BDNF in the fear extinction circuit. Frontiers In Behavioral Neuroscience. 2013; **7**: 102-.
398. Domschke K, Baune BT, Havlik L, Stuhrmann A, Suslow T, Kugel H, et al. Catechol-O-methyltransferase gene variation: impact on amygdala response to aversive stimuli. Neuroimage. 2012; **60**(4): 2222-9.
399. Domschke K, Baune BT, Havlik L, Stuhrmann A, Suslow T, Kugel H, et al. Catechol-O-methyltransferase gene variation: impact on amygdala response to aversive stimuli. Neuroimage. 2012; **60**(4): 2222-9.
400. Domschke K, Gajewska A, Winter B, Herrmann MJ, Warrings B, Muhlberger A, et al. ADORA2A Gene variation, caffeine, and emotional processing: a multi-level interaction on startle reflex. Neuropsychopharmacology. 2012; **37**(3): 759-69.
401. Domschke K, Klauke B, Winter B, Gajewska A, Herrmann MJ, Warrings B, et al. Modification of caffeine effects on the affect-modulated startle by neuropeptide S receptor gene variation. Psychopharmacology (Berl). 2012; **222**(3): 533-41.

402. Domschke K, Reif A. Behavioral genetics of affective and anxiety disorders. Curr Top Behav Neurosci. 2012; **12**: 463-502.
403. Domschke K, Reif A. Behavioral genetics of affective and anxiety disorders. Current Topics In Behavioral Neurosciences. 2012; **12**: 463-502.
404. Domschke K, Winter B, Gajewska A, Unterecker S, Warrings B, Dlugos A, et al. Multilevel impact of the dopamine system on the emotion-potentiated startle reflex. Psychopharmacology (Berl). 2015; **232**(11): 1983-93.
405. Domschke K, Winter B, Gajewska A, Unterecker S, Warrings B, Dlugos A, et al. Multilevel impact of the dopamine system on the emotion-potentiated startle reflex. Psychopharmacology. 2015; **232**(11): 1983-93.
406. Dong JM, Wang LC, Chen Q, Yu H, Yang J, Guo ZR, et al. [A nested case-control study on the relationship between occupational physical activity, heredity factors and metabolic syndrome]. Zhonghua Liu Xing Bing Xue Za Zhi. 2010; **31**(4): 379-83.
407. Dong J-m, Wang L-c, Chen Q, Yu H, Yang J, Guo Z-r, et al. [A nested case-control study on the relationship between occupational physical activity, heredity factors and metabolic syndrome]. Zhonghua Liu Xing Bing Xue Za Zhi = Zhonghua Liuxingbingxue Zazhi. 2010; **31**(4): 379-83.
408. Donmez C, Konac E, Aydogan BT, Bilen CY. Might E-cadherin promoter polymorphisms of rs16260 and rs5030625 associate with the risk of nephrolithiasis? Springerplus. 2016; **5**(1): 1673.
409. Donmez C, Konac E, Aydogan BT, Bilen CY. Might E-cadherin promoter polymorphisms of rs16260 and rs5030625 associate with the risk of nephrolithiasis? Springerplus. 2016; **5**(1): 1673-.
410. Dorado P, Penas-Lledo EM, Llerena A. CYP2D6 polymorphism: implications for antipsychotic drug response, schizophrenia and personality traits. Pharmacogenomics. 2007; **8**(11): 1597-608.
411. Dorado P, Peñas-Lledó EM, Llerena A. CYP2D6 polymorphism: implications for antipsychotic drug response, schizophrenia and personality traits. Pharmacogenomics. 2007; **8**(11): 1597-608.
412. Dorjgochoo T, Shi J, Gao YT, Long J, Delahanty R, Xiang YB, et al. Genetic variants in vitamin D metabolism-related genes and body mass index: analysis of genome-wide scan data of approximately 7000 Chinese women. Int J Obes (Lond). 2012; **36**(9): 1252-5.
413. Dorjgochoo T, Shi J, Gao YT, Long J, Delahanty R, Xiang YB, et al. Genetic variants in vitamin D metabolism-related genes and body mass index: analysis of genome-wide scan data of approximately 7000 Chinese women. International Journal Of Obesity (2005). 2012; **36**(9): 1252-5.
414. Dotson VM, Hsu FC, Langaee TY, McDonough CW, King AC, Cohen RA, et al. Genetic Moderators of the Impact of Physical Activity on Depressive Symptoms. J Frailty Aging. 2016; **5**(1): 6-14.
415. Dotson VM, Hsu FC, Langaee TY, McDonough CW, King AC, Cohen RA, et al. Genetic Moderators of the Impact of Physical Activity on Depressive Symptoms. The Journal Of Frailty & Aging. 2016; **5**(1): 6-14.
416. Drabant EM, Hariri AR, Meyer-Lindenberg A, Munoz KE, Mattay VS, Kolachana BS, et al. Catechol O-methyltransferase val158met genotype and neural mechanisms

related to affective arousal and regulation. Arch Gen Psychiatry. 2006; **63**(12): 1396-406.
417. Drabant EM, Hariri AR, Meyer-Lindenberg A, Munoz KE, Mattay VS, Kolachana BS, et al. Catechol O-methyltransferase val158met genotype and neural mechanisms related to affective arousal and regulation. Archives Of General Psychiatry. 2006; **63**(12): 1396-406.
418. Drago A, Liappas I, Petio C, Albani D, Forloni G, Malitas P, et al. Epistasis between IL1A, IL1B, TNF, HTR2A, 5-HTTLPR and TPH2 variations does not impact alcohol dependence disorder features. Int J Environ Res Public Health. 2009; **6**(7): 1980-90.
419. Drago A, Liappas I, Petio C, Albani D, Forloni G, Malitas P, et al. Epistasis between IL1A, IL1B, TNF, HTR2A, 5-HTTLPR and TPH2 variations does not impact alcohol dependence disorder features. International Journal Of Environmental Research And Public Health. 2009; **6**(7): 1980-90.
420. Dreimuller N, Schlicht KF, Wagner S, Peetz D, Borysenko L, Hiemke C, et al. Early reactions of brain-derived neurotrophic factor in plasma (pBDNF) and outcome to acute antidepressant treatment in patients with Major Depression. Neuropharmacology. 2012; **62**(1): 264-9.
421. Dreimüller N, Schlicht KF, Wagner S, Peetz D, Borysenko L, Hiemke C, et al. Early reactions of brain-derived neurotrophic factor in plasma (pBDNF) and outcome to acute antidepressant treatment in patients with Major Depression. Neuropharmacology. 2012; **62**(1): 264-9.
422. Duncan WC, Jr., Ballard ED, Zarate CA. Ketamine-Induced Glutamatergic Mechanisms of Sleep and Wakefulness: Insights for Developing Novel Treatments for Disturbed Sleep and Mood. Handb Exp Pharmacol. 2017.
423. Duncan WC, Jr., Ballard ED, Zarate CA. Ketamine-Induced Glutamatergic Mechanisms of Sleep and Wakefulness: Insights for Developing Novel Treatments for Disturbed Sleep and Mood. Handbook Of Experimental Pharmacology. 2017.
424. Duncan WC, Sarasso S, Ferrarelli F, Selter J, Riedner BA, Hejazi NS, et al. Concomitant BDNF and sleep slow wave changes indicate ketamine-induced plasticity in major depressive disorder. Int J Neuropsychopharmacol. 2013; **16**(2): 301-11.
425. Duncan WC, Sarasso S, Ferrarelli F, Selter J, Riedner BA, Hejazi NS, et al. Concomitant BDNF and sleep slow wave changes indicate ketamine-induced plasticity in major depressive disorder. The International Journal Of Neuropsychopharmacology. 2013; **16**(2): 301-11.
426. Dunlop BW, Binder EB, Iosifescu D, Mathew SJ, Neylan TC, Pape JC, et al. Corticotropin-Releasing Factor Receptor 1 Antagonism Is Ineffective for Women With Posttraumatic Stress Disorder. Biol Psychiatry. 2017; **82**(12): 866-74.
427. Dunlop BW, Binder EB, Iosifescu D, Mathew SJ, Neylan TC, Pape JC, et al. Corticotropin-Releasing Factor Receptor 1 Antagonism Is Ineffective for Women With Posttraumatic Stress Disorder. Biological Psychiatry. 2017; **82**(12): 866-74.
428. Dykens EM, Roof E, Bittel D, Butler MG. TPH2 G/T polymorphism is associated with hyperphagia, IQ, and internalizing problems in Prader-Willi syndrome. J Child Psychol Psychiatry. 2011; **52**(5): 580-7.
429. Dykens EM, Roof E, Bittel D, Butler MG. TPH2 G/T polymorphism is associated

with hyperphagia, IQ, and internalizing problems in Prader-Willi syndrome. Journal Of Child Psychology And Psychiatry, And Allied Disciplines. 2011; **52**(5): 580-7.
430. Ebrahim MS, Lawson ML, Geraghty MT. A novel heterozygous mutation in the glucokinase gene conferring exercise-induced symptomatic hyperglycaemia responsive to sulfonylurea. Diabetes Metab. 2014; **40**(4): 310-3.
431. Ebrahim MSE, Lawson ML, Geraghty MT. A novel heterozygous mutation in the glucokinase gene conferring exercise-induced symptomatic hyperglycaemia responsive to sulfonylurea. Diabetes & Metabolism. 2014; **40**(4): 310-3.
432. Eckdahl TT, Campbell AM, Heyer LJ, Poet JL, Blauch DN, Snyder NL, et al. Programmed evolution for optimization of orthogonal metabolic output in bacteria. PLoS One. 2015; **10**(2): e0118322.
433. Eckdahl TT, Campbell AM, Heyer LJ, Poet JL, Blauch DN, Snyder NL, et al. Programmed evolution for optimization of orthogonal metabolic output in bacteria. PLoS One. 2015; **10**(2): e0118322-e.
434. Eisenhardt A, Sperling H, Rubben H. [Genetic aspects of erectile dysfunction]. Urologe A. 2015; **54**(5): 662-7.
435. Eisenhardt A, Sperling H, Rübben H. [Genetic aspects of erectile dysfunction]. Der Urologe Ausg A. 2015; **54**(5): 662-7.
436. Eisenmann JC. Physical activity and cardiovascular disease risk factors in children and adolescents: an overview. Can J Cardiol. 2004; **20**(3): 295-301.
437. Eisenmann JC. Physical activity and cardiovascular disease risk factors in children and adolescents: an overview. The Canadian Journal Of Cardiology. 2004; **20**(3): 295-301.
438. Eisman JA. Genetics of osteoporosis. Endocr Rev. 1999; **20**(6): 788-804.
439. Eisman JA. Genetics of osteoporosis. Endocrine Reviews. 1999; **20**(6): 788-804.
440. Ekladious SM, Issac MS, El-Atty Sharaf SA, Abou-Youssef HS. Validation of a proposed warfarin dosing algorithm based on the genetic make-up of Egyptian patients. Mol Diagn Ther. 2013; **17**(6): 381-90.
441. Ekladious SMM, Issac MSM, El-Atty Sharaf SA, Abou-Youssef HS. Validation of a proposed warfarin dosing algorithm based on the genetic make-up of Egyptian patients. Molecular Diagnosis & Therapy. 2013; **17**(6): 381-90.
442. Eklof V, Van Guelpen B, Hultdin J, Johansson I, Hallmans G, Palmqvist R. The reduced folate carrier (RFC1) 80G > A and folate hydrolase 1 (FOLH1) 1561C > T polymorphisms and the risk of colorectal cancer: a nested case-referent study. Scand J Clin Lab Invest. 2008; **68**(5): 393-401.
443. Eklöf V, Van Guelpen B, Hultdin J, Johansson I, Hallmans G, Palmqvist R. The reduced folate carrier (RFC1) 80G > A and folate hydrolase 1 (FOLH1) 1561C > T polymorphisms and the risk of colorectal cancer: a nested case-referent study. Scandinavian Journal Of Clinical And Laboratory Investigation. 2008; **68**(5): 393-401.
444. ElHefnawi M, Kim T, Kamar MA, Min S, Hassan NM, El-Ahwany E, et al. In Silico Design and Experimental Validation of siRNAs Targeting Conserved Regions of Multiple Hepatitis C Virus Genotypes. PLoS One. 2016; **11**(7): e0159211.
445. ElHefnawi M, Kim T, Kamar MA, Min S, Hassan NM, El-Ahwany E, et al. In Silico Design and Experimental Validation of siRNAs Targeting Conserved Regions of Multiple Hepatitis C Virus Genotypes. PLoS One. 2016; **11**(7): e0159211-e.

446. Elzinga BM, Molendijk ML, Oude Voshaar RC, Bus BA, Prickaerts J, Spinhoven P, et al. The impact of childhood abuse and recent stress on serum brain-derived neurotrophic factor and the moderating role of BDNF Val66Met. Psychopharmacology (Berl). 2011; **214**(1): 319-28.
447. Elzinga BM, Molendijk ML, Oude Voshaar RC, Bus BAA, Prickaerts J, Spinhoven P, et al. The impact of childhood abuse and recent stress on serum brain-derived neurotrophic factor and the moderating role of BDNF Val66Met. Psychopharmacology. 2011; **214**(1): 319-28.
448. Enoch M-A. Pharmacogenomics of alcohol response and addiction. American Journal Of Pharmacogenomics: Genomics-Related Research In Drug Development And Clinical Practice. 2003; **3**(4): 217-32.
449. Enoch MA. Pharmacogenomics of alcohol response and addiction. Am J Pharmacogenomics. 2003; **3**(4): 217-32.
450. Eny KM, Corey PN, El-Sohemy A. Dopamine D2 receptor genotype (C957T) and habitual consumption of sugars in a free-living population of men and women. J Nutrigenet Nutrigenomics. 2009; **2**(4-5): 235-42.
451. Eny KM, Corey PN, El-Sohemy A. Dopamine D2 receptor genotype (C957T) and habitual consumption of sugars in a free-living population of men and women. Journal Of Nutrigenetics And Nutrigenomics. 2009; **2**(4-5): 235-42.
452. Eny KM, Wolever TM, Corey PN, El-Sohemy A. Genetic variation in TAS1R2 (Ile191Val) is associated with consumption of sugars in overweight and obese individuals in 2 distinct populations. Am J Clin Nutr. 2010; **92**(6): 1501-10.
453. Eny KM, Wolever TM, Corey PN, El-Sohemy A. Genetic variation in TAS1R2 (Ile191Val) is associated with consumption of sugars in overweight and obese individuals in 2 distinct populations. The American Journal Of Clinical Nutrition. 2010; **92**(6): 1501-10.
454. Eny KM, Wolever TM, Fontaine-Bisson B, El-Sohemy A. Genetic variant in the glucose transporter type 2 is associated with higher intakes of sugars in two distinct populations. Physiol Genomics. 2008; **33**(3): 355-60.
455. Eny KM, Wolever TMS, Fontaine-Bisson B, El-Sohemy A. Genetic variant in the glucose transporter type 2 is associated with higher intakes of sugars in two distinct populations. Physiological Genomics. 2008; **33**(3): 355-60.
456. Etnier JL, Wideman L, Labban JD, Piepmeier AT, Pendleton DM, Dvorak KK, et al. The Effects of Acute Exercise on Memory and Brain-Derived Neurotrophic Factor (BDNF). J Sport Exerc Psychol. 2016; **38**(4): 331-40.
457. Etnier JL, Wideman L, Labban JD, Piepmeier AT, Pendleton DM, Dvorak KK, et al. The Effects of Acute Exercise on Memory and Brain-Derived Neurotrophic Factor (BDNF). Journal Of Sport & Exercise Psychology. 2016; **38**(4): 331-40.
458. Eussen SJ, Vollset SE, Igland J, Meyer K, Fredriksen A, Ueland PM, et al. Plasma folate, related genetic variants, and colorectal cancer risk in EPIC. Cancer Epidemiol Biomarkers Prev. 2010; **19**(5): 1328-40.
459. Eussen SJPM, Vollset SE, Igland J, Meyer K, Fredriksen A, Ueland PM, et al. Plasma folate, related genetic variants, and colorectal cancer risk in EPIC. Cancer Epidemiology, Biomarkers & Prevention: A Publication Of The American Association For Cancer Research, Cosponsored By The American Society Of Preventive Oncology.

2010; **19**(5): 1328-40.
460. Evans M, Andresen BS, Nation J, Boneh A. VLCAD deficiency: Follow-up and outcome of patients diagnosed through newborn screening in Victoria. Mol Genet Metab. 2016; **118**(4): 282-7.
461. Evans M, Andresen BS, Nation J, Boneh A. VLCAD deficiency: Follow-up and outcome of patients diagnosed through newborn screening in Victoria. Molecular Genetics And Metabolism. 2016; **118**(4): 282-7.
462. Faludi G, Gonda X, Bagdy G, Dome P. Pharmaco- and therapygenetic aspects in the treatment of anxiety disorders beyond the serotonergic system: a brief review. Neuropsychopharmacol Hung. 2012; **14**(4): 221-9.
463. Faludi G, Gonda X, Bagdy G, Dome P. Pharmaco- and therapygenetic aspects in the treatment of anxiety disorders beyond the serotonergic system: a brief review. Neuropsychopharmacologia Hungarica: A Magyar Pszichofarmakologiai Egyesulet Lapja = Official Journal Of The Hungarian Association Of Psychopharmacology. 2012; **14**(4): 221-9.
464. Fan YM, Raitakari OT, Kahonen M, Hutri-Kahonen N, Juonala M, Marniemi J, et al. Hepatic lipase promoter C-480T polymorphism is associated with serum lipids levels, but not subclinical atherosclerosis: the Cardiovascular Risk in Young Finns Study. Clin Genet. 2009; **76**(1): 46-53.
465. Fan YM, Raitakari OT, Kähönen M, Hutri-Kähönen N, Juonala M, Marniemi J, et al. Hepatic lipase promoter C-480T polymorphism is associated with serum lipids levels, but not subclinical atherosclerosis: the Cardiovascular Risk in Young Finns Study. Clinical Genetics. 2009; **76**(1): 46-53.
466. Fanale D, Amodeo V, Corsini LR, Rizzo S, Bazan V, Russo A. Breast cancer genome-wide association studies: there is strength in numbers. Oncogene. 2012; **31**(17): 2121-8.
467. Fanale D, Amodeo V, Corsini LR, Rizzo S, Bazan V, Russo A. Breast cancer genome-wide association studies: there is strength in numbers. Oncogene. 2012; **31**(17): 2121-8.
468. Farrelly LA, Savage NT, O'Callaghan C, Toulouse A, Yilmazer-Hanke DM. Therapeutic concentrations of valproate but not amitriptyline increase neuropeptide Y (NPY) expression in the human SH-SY5Y neuroblastoma cell line. Regul Pept. 2013; **186**: 123-30.
469. Farrelly LA, Savage NTP, O'Callaghan C, Toulouse A, Yilmazer-Hanke DM. Therapeutic concentrations of valproate but not amitriptyline increase neuropeptide Y (NPY) expression in the human SH-SY5Y neuroblastoma cell line. Regulatory Peptides. 2013; **186**: 123-30.
470. Feart C, Samieri C, Rondeau V, Amieva H, Portet F, Dartigues JF, et al. Adherence to a Mediterranean diet, cognitive decline, and risk of dementia. JAMA. 2009; **302**(6): 638-48.
471. Féart C, Samieri C, Rondeau V, Amieva H, Portet F, Dartigues J-F, et al. Adherence to a Mediterranean diet, cognitive decline, and risk of dementia. JAMA. 2009; **302**(6): 638-48.
472. Feart C, Torres MJ, Samieri C, Jutand MA, Peuchant E, Simopoulos AP, et al. Adherence to a Mediterranean diet and plasma fatty acids: data from the Bordeaux

sample of the Three-City study. Br J Nutr. 2011; **106**(1): 149-58.
473. Féart C, Torrès MJM, Samieri C, Jutand M-A, Peuchant E, Simopoulos AP, et al. Adherence to a Mediterranean diet and plasma fatty acids: data from the Bordeaux sample of the Three-City study. The British Journal Of Nutrition. 2011; **106**(1): 149-58.
474. Federico A, Masarone M, Romano M, Dallio M, Rosato V, Persico M. Rapid Virological Response Represents the Highest Prediction Factor of Response to Antiviral Treatment in HCV-Related Chronic Hepatitis: a Multicenter Retrospective Study. Hepat Mon. 2015; **15**(6): e18640.
475. Federico A, Masarone M, Romano M, Dallio M, Rosato V, Persico M. Rapid Virological Response Represents the Highest Prediction Factor of Response to Antiviral Treatment in HCV-Related Chronic Hepatitis: a Multicenter Retrospective Study. Hepatitis Monthly. 2015; **15**(6): e18640-e.
476. Fellerhoff B, Laumbacher B, Wank R. Responsiveness of a patient in a persistent vegetative state after a coma to weekly injections of autologous activated immune cells: a case report. J Med Case Rep. 2012; **6**: 6.
477. Fellerhoff B, Laumbacher B, Wank R. Responsiveness of a patient in a persistent vegetative state after a coma to weekly injections of autologous activated immune cells: a case report. Journal Of Medical Case Reports. 2012; **6**: 6-.
478. Feodorova YN, Sarafian VS. Psychological stress--cellular and molecular mechanisms. Folia Med (Plovdiv). 2012; **54**(3): 5-13.
479. Feodorova YN, Sarafian VS. Psychological stress--cellular and molecular mechanisms. Folia Medica. 2012; **54**(3): 5-13.
480. Ferenci P, Aires R, Beavers KL, Curescu M, Abrao Ferreira PR, Gschwantler M, et al. Predictive value of FIB-4 and APRI versus METAVIR on sustained virologic response in genotype 1 hepatitis C patients. Hepatol Int. 2014; **8**(1): 83-93.
481. Ferenci P, Aires R, Beavers KL, Curescu M, Abrão Ferreira PR, Gschwantler M, et al. Predictive value of FIB-4 and APRI versus METAVIR on sustained virologic response in genotype 1 hepatitis C patients. Hepatology International. 2014; **8**(1): 83-93.
482. Ferrari SL, Karasik D, Liu J, Karamohamed S, Herbert AG, Cupples LA, et al. Interactions of interleukin-6 promoter polymorphisms with dietary and lifestyle factors and their association with bone mass in men and women from the Framingham Osteoporosis Study. J Bone Miner Res. 2004; **19**(4): 552-9.
483. Ferrari SL, Karasik D, Liu J, Karamohamed S, Herbert AG, Cupples LA, et al. Interactions of interleukin-6 promoter polymorphisms with dietary and lifestyle factors and their association with bone mass in men and women from the Framingham Osteoporosis Study. Journal Of Bone And Mineral Research: The Official Journal Of The American Society For Bone And Mineral Research. 2004; **19**(4): 552-9.
484. Ferreira CF, Bernardi JR, Bosa VL, Schuch I, Goldani MZ, Kapczinski F, et al. Correlation between n-3 polyunsaturated fatty acids consumption and BDNF peripheral levels in adolescents. Lipids Health Dis. 2014; **13**: 44.
485. Ferreira CF, Bernardi JR, Bosa VL, Schuch I, Goldani MZ, Kapczinski F, et al. Correlation between n-3 polyunsaturated fatty acids consumption and BDNF peripheral levels in adolescents. Lipids In Health And Disease. 2014; **13**: 44-.
486. Ferreira RN, de Miranda AS, Rocha NP, Silva A, Teixeira AL, da Silva Camargos

ER. Neurotrophic Factors in Parkinson's Disease: What have we Learned from Pre-Clinical and Clinical Studies? Curr Med Chem. 2018.
487. Ferreira RN, de Miranda AS, Rocha NP, Silva ACSE, Teixeira AL, da Silva Camargos ER. Neurotrophic Factors in Parkinson's Disease: What have we Learned from Pre-Clinical and Clinical Studies? Current Medicinal Chemistry. 2018.
488. Fields CT, Chassaing B, Castillo-Ruiz A, Osan R, Gewirtz AT, de Vries GJ. Effects of gut-derived endotoxin on anxiety-like and repetitive behaviors in male and female mice. Biol Sex Differ. 2018; **9**(1): 7.
489. Fields CT, Chassaing B, Castillo-Ruiz A, Osan R, Gewirtz AT, de Vries GJ. Effects of gut-derived endotoxin on anxiety-like and repetitive behaviors in male and female mice. Biology Of Sex Differences. 2018; **9**(1): 7-.
490. Figgitt DP, McClellan KJ. Fluvoxamine. An updated review of its use in the management of adults with anxiety disorders. Drugs. 2000; **60**(4): 925-54.
491. Figgitt DP, McClellan KJ. Fluvoxamine. An updated review of its use in the management of adults with anxiety disorders. Drugs. 2000; **60**(4): 925-54.
492. Finckh U, Rommelspacher H, Kuhn S, Dufeu P, Otto G, Heinz A, et al. Influence of the dopamine D2 receptor (DRD2) genotype on neuroadaptive effects of alcohol and the clinical outcome of alcoholism. Pharmacogenetics. 1997; **7**(4): 271-81.
493. Finckh U, Rommelspacher H, Kuhn S, Dufeu P, Otto G, Heinz A, et al. Influence of the dopamine D2 receptor (DRD2) genotype on neuroadaptive effects of alcohol and the clinical outcome of alcoholism. Pharmacogenetics. 1997; **7**(4): 271-81.
494. Fischer AG, Endrass T, Goebel I, Reuter M, Montag C, Kubisch C, et al. Interactive effects of citalopram and serotonin transporter genotype on neural correlates of response inhibition and attentional orienting. Neuroimage. 2015; **116**: 59-67.
495. Fischer AG, Endrass T, Goebel I, Reuter M, Montag C, Kubisch C, et al. Interactive effects of citalopram and serotonin transporter genotype on neural correlates of response inhibition and attentional orienting. Neuroimage. 2015; **116**: 59-67.
496. Fond G, Capdevielle D, Macgregor A, Attal J, Larue A, Brittner M, et al. [Toxoplasma gondii: a potential role in the genesis of psychiatric disorders]. Encephale. 2013; **39**(1): 38-43.
497. Fond G, Capdevielle D, Macgregor A, Attal J, Larue A, Brittner M, et al. [Toxoplasma gondii: a potential role in the genesis of psychiatric disorders]. L'encephale. 2013; **39**(1): 38-43.
498. Fontenelle LF, Barbosa IG, Luna JV, Rocha NP, Silva Miranda A, Teixeira AL. Neurotrophic factors in obsessive-compulsive disorder. Psychiatry Res. 2012; **199**(3): 195-200.
499. Fontenelle LF, Barbosa IG, Luna JV, Rocha NP, Silva Miranda A, Teixeira AL. Neurotrophic factors in obsessive-compulsive disorder. Psychiatry Research. 2012; **199**(3): 195-200.
500. Ford ES, Zhao G, Tsai J, Li C. Low-risk lifestyle behaviors and all-cause mortality: findings from the National Health and Nutrition Examination Survey III Mortality Study. Am J Public Health. 2011; **101**(10): 1922-9.
501. Ford ES, Zhao G, Tsai J, Li C. Low-risk lifestyle behaviors and all-cause mortality: findings from the National Health and Nutrition Examination Survey III Mortality Study. American Journal Of Public Health. 2011; **101**(10): 1922-9.

502. Forstner AJ, Rambau S, Friedrich N, Ludwig KU, Bohmer AC, Mangold E, et al. Further evidence for genetic variation at the serotonin transporter gene SLC6A4 contributing toward anxiety. Psychiatr Genet. 2017; **27**(3): 96-102.
503. Forstner AJ, Rambau S, Friedrich N, Ludwig KU, Böhmer AC, Mangold E, et al. Further evidence for genetic variation at the serotonin transporter gene SLC6A4 contributing toward anxiety. Psychiatric Genetics. 2017; **27**(3): 96-102.
504. Fossum E, Gleim GW, Kjeldsen SE, Kizer JR, Julius S, Devereux RB, et al. The effect of baseline physical activity on cardiovascular outcomes and new-onset diabetes in patients treated for hypertension and left ventricular hypertrophy: the LIFE study. J Intern Med. 2007; **262**(4): 439-48.
505. Fossum E, Gleim GW, Kjeldsen SE, Kizer JR, Julius S, Devereux RB, et al. The effect of baseline physical activity on cardiovascular outcomes and new-onset diabetes in patients treated for hypertension and left ventricular hypertrophy: the LIFE study. Journal Of Internal Medicine. 2007; **262**(4): 439-48.
506. Foster RH, Goa KL. Paroxetine : a review of its pharmacology and therapeutic potential in the management of panic disorder. CNS Drugs. 1997; **8**(2): 163-88.
507. Foster RH, Goa KL. Paroxetine : a review of its pharmacology and therapeutic potential in the management of panic disorder. CNS Drugs. 1997; **8**(2): 163-88.
508. Franceschini N, Rose KM, Storti KL, Rutherford S, Voruganti VS, Laston S, et al. Social- and behavioral-specific genetic effects on blood pressure traits: the Strong Heart Family Study. Circ Cardiovasc Genet. 2009; **2**(4): 396-401.
509. Franceschini N, Rose KM, Storti KL, Rutherford S, Voruganti VS, Laston S, et al. Social- and behavioral-specific genetic effects on blood pressure traits: the Strong Heart Family Study. Circulation Cardiovascular Genetics. 2009; **2**(4): 396-401.
510. Franks PW, Bhattacharyya S, Luan J, Montague C, Brennand J, Challis B, et al. Association between physical activity and blood pressure is modified by variants in the G-protein coupled receptor 10. Hypertension. 2004; **43**(2): 224-8.
511. Franks PW, Bhattacharyya S, Luan Ja, Montague C, Brennand J, Challis B, et al. Association between physical activity and blood pressure is modified by variants in the G-protein coupled receptor 10. Hypertension (Dallas, Tex: 1979). 2004; **43**(2): 224-8.
512. Franks PW, Jablonski KA, Delahanty LM, McAteer JB, Kahn SE, Knowler WC, et al. Assessing gene-treatment interactions at the FTO and INSIG2 loci on obesity-related traits in the Diabetes Prevention Program. Diabetologia. 2008; **51**(12): 2214-23.
513. Franks PW, Jablonski KA, Delahanty LM, McAteer JB, Kahn SE, Knowler WC, et al. Assessing gene-treatment interactions at the FTO and INSIG2 loci on obesity-related traits in the Diabetes Prevention Program. Diabetologia. 2008; **51**(12): 2214-23.
514. Fu LM. Exploring drug action on Mycobacterium tuberculosis using affymetrix oligonucleotide genechips. Tuberculosis (Edinb). 2006; **86**(2): 134-43.
515. Fu LM. Exploring drug action on Mycobacterium tuberculosis using affymetrix oligonucleotide genechips. Tuberculosis (Edinburgh, Scotland). 2006; **86**(2): 134-43.
516. Fulton JE, Dai S, Grunbaum JA, Boerwinkle E, Labarthe DR. Effects of apolipoprotein E genotype on blood cholesterol in adolescent girls. Am J Prev Med. 2009; **37**(1 Suppl): S78-85.
517. Fulton JE, Dai S, Grunbaum JA, Boerwinkle E, Labarthe DR. Effects of apolipoprotein E genotype on blood cholesterol in adolescent girls. American Journal Of

Preventive Medicine. 2009; **37**(1 Suppl): S78-S85.
518. Gajewska A, Blumenthal TD, Winter B, Herrmann MJ, Conzelmann A, Mühlberger A, et al. Effects of ADORA2A gene variation and caffeine on prepulse inhibition: a multi-level risk model of anxiety. Progress In Neuro-Psychopharmacology & Biological Psychiatry. 2013; **40**: 115-21.
519. Gajski G, Geric M, Orescanin V, Garaj-Vrhovac V. Cytokinesis-block micronucleus cytome assay parameters in peripheral blood lymphocytes of the general population: Contribution of age, sex, seasonal variations and lifestyle factors. Ecotoxicol Environ Saf. 2018; **148**: 561-70.
520. Gajski G, Gerić M, Oreščanin V, Garaj-Vrhovac V. Cytokinesis-block micronucleus cytome assay parameters in peripheral blood lymphocytes of the general population: Contribution of age, sex, seasonal variations and lifestyle factors. Ecotoxicology And Environmental Safety. 2018; **148**: 561-70.
521. Gale P. The infectivity of transmissible spongiform encephalopathy agent at low doses: the importance of phospholipid. J Appl Microbiol. 2006; **101**(2): 261-74.
522. Gale P. The infectivity of transmissible spongiform encephalopathy agent at low doses: the importance of phospholipid. Journal Of Applied Microbiology. 2006; **101**(2): 261-74.
523. Galjaard S, Devlieger R, Van Assche FA. Fetal growth and developmental programming. J Perinat Med. 2013; **41**(1): 101-5.
524. Galjaard S, Devlieger R, Van Assche FA. Fetal growth and developmental programming. Journal Of Perinatal Medicine. 2013; **41**(1): 101-5.
525. Gallagher PM, Meleady R, Shields DC, Tan KS, McMaster D, Rozen R, et al. Homocysteine and risk of premature coronary heart disease. Evidence for a common gene mutation. Circulation. 1996; **94**(9): 2154-8.
526. Gallagher PM, Meleady R, Shields DC, Tan KS, McMaster D, Rozen R, et al. Homocysteine and risk of premature coronary heart disease. Evidence for a common gene mutation. Circulation. 1996; **94**(9): 2154-8.
527. Galvao-de Almeida A, Quarantini LC, Sampaio AS, Lyra AC, Parise CL, Parana R, et al. Lack of association of indoleamine 2,3-dioxygenase polymorphisms with interferon-alpha-related depression in hepatitis C. Brain Behav Immun. 2011; **25**(7): 1491-7.
528. Galvão-de Almeida A, Quarantini LC, Sampaio AS, Lyra AC, Parise CL, Paraná R, et al. Lack of association of indoleamine 2,3-dioxygenase polymorphisms with interferon-alpha-related depression in hepatitis C. Brain, Behavior, And Immunity. 2011; **25**(7): 1491-7.
529. Gamble KL, Motsinger-Reif AA, Hida A, Borsetti HM, Servick SV, Ciarleglio CM, et al. Shift work in nurses: contribution of phenotypes and genotypes to adaptation. PLoS One. 2011; **6**(4): e18395.
530. Gamble KL, Motsinger-Reif AA, Hida A, Borsetti HM, Servick SV, Ciarleglio CM, et al. Shift work in nurses: contribution of phenotypes and genotypes to adaptation. PLoS One. 2011; **6**(4): e18395-e.
531. Garaulet M, Smith CE, Gomez-Abellan P, Ordovas-Montanes M, Lee YC, Parnell LD, et al. REV-ERB-ALPHA circadian gene variant associates with obesity in two independent populations: Mediterranean and North American. Mol Nutr Food Res.

2014; **58**(4): 821-9.
532. Garaulet M, Smith CE, Gomez-Abellán P, Ordovás-Montañés M, Lee Y-C, Parnell LD, et al. REV-ERB-ALPHA circadian gene variant associates with obesity in two independent populations: Mediterranean and North American. Molecular Nutrition & Food Research. 2014; **58**(4): 821-9.
533. Gardier AM. Antidepressant activity: contribution of brain microdialysis in knock-out mice to the understanding of BDNF/5-HT transporter/5-HT autoreceptor interactions. Front Pharmacol. 2013; **4**: 98.
534. Gardier AM. Antidepressant activity: contribution of brain microdialysis in knock-out mice to the understanding of BDNF/5-HT transporter/5-HT autoreceptor interactions. Frontiers In Pharmacology. 2013; **4**: 98-.
535. Gardner RC, Valcour V, Yaffe K. Dementia in the oldest old: a multi-factorial and growing public health issue. Alzheimers Res Ther. 2013; **5**(4): 27.
536. Gardner RC, Valcour V, Yaffe K. Dementia in the oldest old: a multi-factorial and growing public health issue. Alzheimer's Research & Therapy. 2013; **5**(4): 27-.
537. Garnero P, Munoz F, Borel O, Sornay-Rendu E, Delmas PD. Vitamin D receptor gene polymorphisms are associated with the risk of fractures in postmenopausal women, independently of bone mineral density. J Clin Endocrinol Metab. 2005; **90**(8): 4829-35.
538. Garnero P, Munoz F, Borel O, Sornay-Rendu E, Delmas PD. Vitamin D receptor gene polymorphisms are associated with the risk of fractures in postmenopausal women, independently of bone mineral density. The Journal Of Clinical Endocrinology And Metabolism. 2005; **90**(8): 4829-35.
539. Gattere G, Stojanovic-Perez A, Monseny R, Martorell L, Ortega L, Montalvo I, et al. Gene-environment interaction between the brain-derived neurotrophic factor Val66Met polymorphism, psychosocial stress and dietary intake in early psychosis. Early Interv Psychiatry. 2016.
540. Gattere G, Stojanovic-Pérez A, Monseny R, Martorell L, Ortega L, Montalvo I, et al. Gene-environment interaction between the brain-derived neurotrophic factor Val66Met polymorphism, psychosocial stress and dietary intake in early psychosis. Early Intervention In Psychiatry. 2016.
541. Gee P, Richardson S, Woltersdorf W, Moore G. Toxic effects of BZP-based herbal party pills in humans: a prospective study in Christchurch, New Zealand. N Z Med J. 2005; **118**(1227): U1784.
542. Gee P, Richardson S, Woltersdorf W, Moore G. Toxic effects of BZP-based herbal party pills in humans: a prospective study in Christchurch, New Zealand. The New Zealand Medical Journal. 2005; **118**(1227): U1784-U.
543. Geiger MJ, Domschke K, Homola GA, Schulz SM, Nowak J, Akhrif A, et al. ADORA2A genotype modulates interoceptive and exteroceptive processing in a fronto-insular network. Eur Neuropsychopharmacol. 2016; **26**(8): 1274-85.
544. Geiger MJ, Domschke K, Homola GA, Schulz SM, Nowak J, Akhrif A, et al. ADORA2A genotype modulates interoceptive and exteroceptive processing in a fronto-insular network. European Neuropsychopharmacology: The Journal Of The European College Of Neuropsychopharmacology. 2016; **26**(8): 1274-85.
545. Gentile MA, Nantermet PV, Vogel RL, Phillips R, Holder D, Hodor P, et al.

Androgen-mediated improvement of body composition and muscle function involves a novel early transcriptional program including IGF1, mechano growth factor, and induction of {beta}-catenin. J Mol Endocrinol. 2010; **44**(1): 55-73.
546. Gentile MA, Nantermet PV, Vogel RL, Phillips R, Holder D, Hodor P, et al. Androgen-mediated improvement of body composition and muscle function involves a novel early transcriptional program including IGF1, mechano growth factor, and induction of {beta}-catenin. Journal Of Molecular Endocrinology. 2010; **44**(1): 55-73.
547. George SZ, Wallace MR, Wright TW, Moser MW, Greenfield WH, 3rd, Sack BK, et al. Evidence for a biopsychosocial influence on shoulder pain: pain catastrophizing and catechol-O-methyltransferase (COMT) diplotype predict clinical pain ratings. Pain. 2008; **136**(1-2): 53-61.
548. George SZ, Wallace MR, Wright TW, Moser MW, Greenfield WH, 3rd, Sack BK, et al. Evidence for a biopsychosocial influence on shoulder pain: pain catastrophizing and catechol-O-methyltransferase (COMT) diplotype predict clinical pain ratings. Pain. 2008; **136**(1-2): 53-61.
549. George SZ, Wu SS, Wallace MR, Moser MW, Wright TW, Farmer KW, et al. Biopsychosocial Influence on Shoulder Pain: Influence of Genetic and Psychological Combinations on Twelve-Month Postoperative Pain and Disability Outcomes. Arthritis Care Res (Hoboken). 2016; **68**(11): 1671-80.
550. George SZ, Wu SS, Wallace MR, Moser MW, Wright TW, Farmer KW, et al. Biopsychosocial Influence on Shoulder Pain: Influence of Genetic and Psychological Combinations on Twelve-Month Postoperative Pain and Disability Outcomes. Arthritis Care & Research. 2016; **68**(11): 1671-80.
551. German-Ponciano LJ, Rosas-Sanchez GU, Rivadeneyra-Dominguez E, Rodriguez-Landa JF. Advances in the Preclinical Study of Some Flavonoids as Potential Antidepressant Agents. Scientifica (Cairo). 2018; **2018**: 2963565.
552. German-Ponciano LJ, Rosas-Sánchez GU, Rivadeneyra-Domínguez E, Rodríguez-Landa JF. Advances in the Preclinical Study of Some Flavonoids as Potential Antidepressant Agents. Scientifica. 2018; **2018**: 2963565-.
553. Giaccaglia V, Nicklas B, Kritchevsky S, Mychalecky J, Messier S, Bleecker E, et al. Interaction between angiotensin converting enzyme insertion/deletion genotype and exercise training on knee extensor strength in older individuals. Int J Sports Med. 2008; **29**(1): 40-4.
554. Giaccaglia V, Nicklas B, Kritchevsky S, Mychalecky J, Messier S, Bleecker E, et al. Interaction between angiotensin converting enzyme insertion/deletion genotype and exercise training on knee extensor strength in older individuals. International Journal Of Sports Medicine. 2008; **29**(1): 40-4.
555. Gibson EL. Tryptophan supplementation and serotonin function: genetic variations in behavioural effects. Proc Nutr Soc. 2018: 1-15.
556. Gibson EL. Tryptophan supplementation and serotonin function: genetic variations in behavioural effects. The Proceedings Of The Nutrition Society. 2018; **77**(2): 174-88.
557. Gilbert DG, Zuo Y, Rabinovich NE, Riise H, Needham R, Huggenvik JI. Neurotransmission-related genetic polymorphisms, negative affectivity traits, and gender predict tobacco abstinence symptoms across 44 days with and without nicotine

patch. J Abnorm Psychol. 2009; **118**(2): 322-34.
558. Gilbert DG, Zuo Y, Rabinovich NE, Riise H, Needham R, Huggenvik JI. Neurotransmission-related genetic polymorphisms, negative affectivity traits, and gender predict tobacco abstinence symptoms across 44 days with and without nicotine patch. Journal Of Abnormal Psychology. 2009; **118**(2): 322-34.
559. Gillesberg Lassen S, Ethelberg S, Bjorkman JT, Jensen T, Sorensen G, Kvistholm Jensen A, et al. Two listeria outbreaks caused by smoked fish consumption-using whole-genome sequencing for outbreak investigations. Clin Microbiol Infect. 2016; **22**(7): 620-4.
560. Gillesberg Lassen S, Ethelberg S, Björkman JT, Jensen T, Sørensen G, Kvistholm Jensen A, et al. Two listeria outbreaks caused by smoked fish consumption-using whole-genome sequencing for outbreak investigations. Clinical Microbiology And Infection: The Official Publication Of The European Society Of Clinical Microbiology And Infectious Diseases. 2016; **22**(7): 620-4.
561. Giroud C, Augsburger M, Favrat B, Menetrey A, Pin MA, Rothuizen LE, et al. [Effects of oral cannabis and dronabinol on driving capacity]. Ann Pharm Fr. 2006; **64**(3): 161-72.
562. Giroud C, Augsburger M, Favrat B, Menetrey A, Pin MA, Rothuizen LE, et al. [Effects of oral cannabis and dronabinol on driving capacity]. Annales Pharmaceutiques Francaises. 2006; **64**(3): 161-72.
563. Glatz K, Mossner R, Heils A, Lesch KP. Glucocorticoid-regulated human serotonin transporter (5-HTT) expression is modulated by the 5-HTT gene-promotor-linked polymorphic region. J Neurochem. 2003; **86**(5): 1072-8.
564. Glatz K, Mössner R, Heils A, Lesch KP. Glucocorticoid-regulated human serotonin transporter (5-HTT) expression is modulated by the 5-HTT gene-promotor-linked polymorphic region. Journal Of Neurochemistry. 2003; **86**(5): 1072-8.
565. Gloster AT, Gerlach AL, Hamm A, Hofler M, Alpers GW, Kircher T, et al. 5HTT is associated with the phenotype psychological flexibility: results from a randomized clinical trial. Eur Arch Psychiatry Clin Neurosci. 2015; **265**(5): 399-406.
566. Gloster AT, Gerlach AL, Hamm A, Höfler M, Alpers GW, Kircher T, et al. 5HTT is associated with the phenotype psychological flexibility: results from a randomized clinical trial. European Archives Of Psychiatry And Clinical Neuroscience. 2015; **265**(5): 399-406.
567. Gmiat A, Jaworska J, Micielska K, Kortas J, Prusik K, Lipowski M, et al. Improvement of cognitive functions in response to a regular Nordic walking training in elderly women - A change dependent on the training experience. Exp Gerontol. 2018; **104**: 105-12.
568. Gmiąt A, Jaworska J, Micielska K, Kortas J, Prusik K, Lipowski M, et al. Improvement of cognitive functions in response to a regular Nordic walking training in elderly women - A change dependent on the training experience. Experimental Gerontology. 2018; **104**: 105-12.
569. Gochee PA, Powell EE, Purdie DM, Pandeya N, Kelemen L, Shorthouse C, et al. Association between apolipoprotein E epsilon4 and neuropsychiatric symptoms during interferon alpha treatment for chronic hepatitis C. Psychosomatics. 2004; **45**(1): 49-57.
570. Gochee PA, Powell EE, Purdie DM, Pandeya N, Kelemen L, Shorthouse C, et al.

Association between apolipoprotein E epsilon4 and neuropsychiatric symptoms during interferon alpha treatment for chronic hepatitis C. Psychosomatics. 2004; **45**(1): 49-57.
571. Godoy LD, Umeoka EHL, Ribeiro DE, Santos VR, Antunes-Rodrigues J, Joca SRL, et al. Multimodal early-life stress induces biological changes associated to psychopathologies. Horm Behav. 2018; **100**: 69-80.
572. Godoy LD, Umeoka EHL, Ribeiro DE, Santos VR, Antunes-Rodrigues J, Joca SRL, et al. Multimodal early-life stress induces biological changes associated to psychopathologies. Hormones And Behavior. 2018; **100**: 69-80.
573. Gökalp O, Gunes A, Cam H, Cure E, Aydın O, Tamer MN, et al. Mild hypoglycaemic attacks induced by sulphonylureas related to CYP2C9, CYP2C19 and CYP2C8 polymorphisms in routine clinical setting. European Journal Of Clinical Pharmacology. 2011; **67**(12): 1223-9.
574. Gokalp O, Gunes A, Cam H, Cure E, Aydin O, Tamer MN, et al. Mild hypoglycaemic attacks induced by sulphonylureas related to CYP2C9, CYP2C19 and CYP2C8 polymorphisms in routine clinical setting. Eur J Clin Pharmacol. 2011; **67**(12): 1223-9.
575. Golka K, Roemer HC, Weistenhofer W, Blaszkewicz M, Hammad S, Reckwitz T, et al. N-Acetyltransferase 2 and glutathione s-transferase M1 in colon and rectal cancer cases from an industrialized area. J Toxicol Environ Health A. 2012; **75**(8-10): 572-81.
576. Golka K, Roemer HC, Weistenhöfer W, Blaszkewicz M, Hammad S, Reckwitz T, et al. N-Acetyltransferase 2 and glutathione s-transferase M1 in colon and rectal cancer cases from an industrialized area. Journal Of Toxicology And Environmental Health Part A. 2012; **75**(8-10): 572-81.
577. Gomes MV, Toffoli LV, Arruda DW, Soldera LM, Pelosi GG, Neves-Souza RD, et al. Age-related changes in the global DNA methylation profile of leukocytes are linked to nutrition but are not associated with the MTHFR C677T genotype or to functional capacities. PLoS One. 2012; **7**(12): e52570.
578. Gomes MVM, Toffoli LV, Arruda DW, Soldera LM, Pelosi GG, Neves-Souza RD, et al. Age-related changes in the global DNA methylation profile of leukocytes are linked to nutrition but are not associated with the MTHFR C677T genotype or to functional capacities. PLoS One. 2012; **7**(12): e52570-e.
579. Goni L, Cuervo M, Milagro FI, Martinez JA. Gene-Gene Interplay and Gene-Diet Interactions Involving the MTNR1B rs10830963 Variant with Body Weight Loss. J Nutrigenet Nutrigenomics. 2014; **7**(4-6): 232-42.
580. Goni L, Cuervo M, Milagro FI, Martinez JA. Influence of fat intake and BMI on the association of rs1799983 NOS3 polymorphism with blood pressure levels in an Iberian population. Eur J Nutr. 2017; **56**(4): 1589-96.
581. Goni L, Cuervo M, Milagro FI, Martínez JA. Gene-Gene Interplay and Gene-Diet Interactions Involving the MTNR1B rs10830963 Variant with Body Weight Loss. Journal Of Nutrigenetics And Nutrigenomics. 2014; **7**(4-6): 232-42.
582. Goni L, Cuervo M, Milagro FI, Martínez JA. Influence of fat intake and BMI on the association of rs1799983 NOS3 polymorphism with blood pressure levels in an Iberian population. European Journal Of Nutrition. 2017; **56**(4): 1589-96.
583. Goodwill AM, Campbell S, Simpson S, Jr., Bisignano M, Chiang C, Dennerstein L, et al. Vitamin D status is associated with executive function a decade later: Data from

the Women's Healthy Ageing Project. Maturitas. 2018; **107**: 56-62.
584. Goodwill AM, Campbell S, Simpson S, Jr., Bisignano M, Chiang C, Dennerstein L, et al. Vitamin D status is associated with executive function a decade later: Data from the Women's Healthy Ageing Project. Maturitas. 2018; **107**: 56-62.
585. Gorman GS, Elson JL, Newman J, Payne B, McFarland R, Newton JL, et al. Perceived fatigue is highly prevalent and debilitating in patients with mitochondrial disease. Neuromuscul Disord. 2015; **25**(7): 563-6.
586. Gorman GS, Elson JL, Newman J, Payne B, McFarland R, Newton JL, et al. Perceived fatigue is highly prevalent and debilitating in patients with mitochondrial disease. Neuromuscular Disorders: NMD. 2015; **25**(7): 563-6.
587. Gorman JM. New molecular targets for antianxiety interventions. J Clin Psychiatry. 2003; **64 Suppl 3**: 28-35.
588. Gorman JM. New molecular targets for antianxiety interventions. The Journal Of Clinical Psychiatry. 2003; **64 Suppl 3**: 28-35.
589. Görtz B, Fassbender WJ. [Genetics of osteoporosis]. Der Orthopade. 2001; **30**(7): 412-7.
590. Gottschalk MG, Domschke K. Genetics of generalized anxiety disorder and related traits. Dialogues Clin Neurosci. 2017; **19**(2): 159-68.
591. Gottschalk MG, Domschke K. Genetics of generalized anxiety disorder and related traits. Dialogues In Clinical Neuroscience. 2017; **19**(2): 159-68.
592. Goumidi L, Spengler D, Cottel D, Wagner A, Ducimetiere P, Ruidavets JB, et al. Study of the genetic variability of ZAC1 (PLAGL1) in French population-based samples. J Hypertens. 2009; **27**(2): 314-21.
593. Goumidi L, Spengler D, Cottel D, Wagner A, Ducimetière P, Ruidavets J-B, et al. Study of the genetic variability of ZAC1 (PLAGL1) in French population-based samples. Journal Of Hypertension. 2009; **27**(2): 314-21.
594. Goverdhan SV, Hannan S, Newsom RB, Luff AJ, Griffiths H, Lotery AJ. An analysis of the CFH Y402H genotype in AMD patients and controls from the UK, and response to PDT treatment. Eye (Lond). 2008; **22**(6): 849-54.
595. Goverdhan SV, Hannan S, Newsom RB, Luff AJ, Griffiths H, Lotery AJ. An analysis of the CFH Y402H genotype in AMD patients and controls from the UK, and response to PDT treatment. Eye (London, England). 2008; **22**(6): 849-54.
596. Grady BJ, Ritchie MD. Statistical Optimization of Pharmacogenomics Association Studies: Key Considerations from Study Design to Analysis. Curr Pharmacogenomics Person Med. 2011; **9**(1): 41-66.
597. Grady BJ, Ritchie MD. Statistical Optimization of Pharmacogenomics Association Studies: Key Considerations from Study Design to Analysis. Current Pharmacogenomics And Personalized Medicine. 2011; **9**(1): 41-66.
598. Graeff FG, Sant'Ana AB, Vilela-Costa HH, Zangrossi H, Jr. New Findings on the Neurotransmitter Modulation of Defense in the Dorsal Periaqueductal Gray. CNS Neurol Disord Drug Targets. 2015; **14**(8): 988-95.
599. Graeff FG, Sant'Ana AB, Vilela-Costa HH, Zangrossi H, Jr. New Findings on the Neurotransmitter Modulation of Defense in the Dorsal Periaqueductal Gray. CNS & Neurological Disorders Drug Targets. 2015; **14**(8): 988-95.
600. Grange B, Callet-Bauchu E, Salles G, Sujobert P. Advances in the role of

cytogenetic analysis in the molecular diagnosis of B-cell lymphomas. Expert Rev Mol Diagn. 2017; **17**(6): 623-32.
601. Grange B, Callet-Bauchu E, Salles G, Sujobert P. Advances in the role of cytogenetic analysis in the molecular diagnosis of B-cell lymphomas. Expert Review Of Molecular Diagnostics. 2017; **17**(6): 623-32.
602. Greenberg GD, Laman-Maharg A, Campi KL, Voigt H, Orr VN, Schaal L, et al. Sex differences in stress-induced social withdrawal: role of brain derived neurotrophic factor in the bed nucleus of the stria terminalis. Front Behav Neurosci. 2013; **7**: 223.
603. Greenberg GD, Laman-Maharg A, Campi KL, Voigt H, Orr VN, Schaal L, et al. Sex differences in stress-induced social withdrawal: role of brain derived neurotrophic factor in the bed nucleus of the stria terminalis. Frontiers In Behavioral Neuroscience. 2014; **7**: 223-.
604. Grotzinger KM, Younossi ZM, Giannini EG, Chen PJ, Rendas-Baum R, Theodore D. Health-related quality of life in thrombocytopenic patients with chronic hepatitis C with or without cirrhosis in the ENABLE-1 and ENABLE-2 studies. Health Qual Life Outcomes. 2016; **14**: 49.
605. Grotzinger KM, Younossi ZM, Giannini EG, Chen P-J, Rendas-Baum R, Theodore D. Health-related quality of life in thrombocytopenic patients with chronic hepatitis C with or without cirrhosis in the ENABLE-1 and ENABLE-2 studies. Health And Quality Of Life Outcomes. 2016; **14**: 49-.
606. Grzegorzewska AE, Izdebska A, Niepolski L, Warchol W, Jagodzinski PP. Self-Reported Physical Activity, Quality of Life, and Psychological Status in Relation to Plasma 25-Hydroxyvitamin D Concentration in Patients Treated with Hemodialysis. Kidney Blood Press Res. 2016; **41**(6): 886-900.
607. Grzegorzewska AE, Izdebska A, Niepolski L, Warchoł W, Jagodziński PP. Self-Reported Physical Activity, Quality of Life, and Psychological Status in Relation to Plasma 25-Hydroxyvitamin D Concentration in Patients Treated with Hemodialysis. Kidney & Blood Pressure Research. 2016; **41**(6): 886-900.
608. Gu SJ, Liu MM, Guo ZR, Wu M, Chen Q, Zhou ZY, et al. [Gene-gene interactions among the peroxisome proliferator-activated receptor polymorphisms for hypertriglyceridemia]. Zhonghua Yu Fang Yi Xue Za Zhi. 2012; **46**(10): 916-21.
609. Gu S-j, Liu M-m, Guo Z-r, Wu M, Chen Q, Zhou Z-y, et al. [Gene-gene interactions among the peroxisome proliferator-activated receptor polymorphisms for hypertriglyceridemia]. Zhonghua Yu Fang Yi Xue Za Zhi [Chinese Journal Of Preventive Medicine]. 2012; **46**(10): 916-21.
610. Guerrero JA, Rivera J, Quiroga T, Martinez-Perez A, Anton AI, Martinez C, et al. Novel loci involved in platelet function and platelet count identified by a genome-wide study performed in children. Haematologica. 2011; **96**(9): 1335-43.
611. Guerrero JA, Rivera J, Quiroga T, Martinez-Perez A, Antón AI, Martínez C, et al. Novel loci involved in platelet function and platelet count identified by a genome-wide study performed in children. Haematologica. 2011; **96**(9): 1335-43.
612. Guest N, Corey P, Vescovi J, El-Sohemy A. Caffeine, CYP1A2 Genotype, and Endurance Performance in Athletes. Med Sci Sports Exerc. 2018.
613. Guidi C, Potenza L, Sestili P, Martinelli C, Guescini M, Stocchi L, et al. Differential effect of creatine on oxidatively-injured mitochondrial and nuclear DNA. Biochim

Biophys Acta. 2008; **1780**(1): 16-26.
614. Guidi C, Potenza L, Sestili P, Martinelli C, Guescini M, Stocchi L, et al. Differential effect of creatine on oxidatively-injured mitochondrial and nuclear DNA. Biochimica Et Biophysica Acta. 2008; **1780**(1): 16-26.
615. Gulyasar T, Ozturk L, Sipahi T, Bayraktar B, Metin G, Yucesir I, et al. GNB3 gene c.825C>T polymorphism and performance parameters in professional basketball players. Acta Physiol Hung. 2014; **101**(2): 176-84.
616. Gülyaşar T, Oztürk L, Sipahi T, Bayraktar B, Metin G, Yücesir I, et al. GNB3 gene c.825C>T polymorphism and performance parameters in professional basketball players. Acta Physiologica Hungarica. 2014; **101**(2): 176-84.
617. Gungor K, Beydagi H, Bekir N, Arslan C, Suer C, Erbagci I, et al. The impact of acute dynamic exercise on intraocular pressure: role of the beta 2-adrenergic receptor polymorphism. J Int Med Res. 2002; **30**(1): 26-33.
618. Güngör K, Beydaği H, Bekir N, Arslan C, Süer C, Erbağci I, et al. The impact of acute dynamic exercise on intraocular pressure: role of the beta 2-adrenergic receptor polymorphism. The Journal Of International Medical Research. 2002; **30**(1): 26-33.
619. Gunnes M, Berg JP, Halse J, Lehmann EH. Lack of relationship between vitamin D receptor genotype and forearm bone gain in healthy children, adolescents, and young adults. J Clin Endocrinol Metab. 1997; **82**(3): 851-5.
620. Gunnes M, Berg JP, Halse J, Lehmann EH. Lack of relationship between vitamin D receptor genotype and forearm bone gain in healthy children, adolescents, and young adults. The Journal Of Clinical Endocrinology And Metabolism. 1997; **82**(3): 851-5.
621. Haerian BS, Roslan H, Raymond AA, Tan CT, Lim KS, Zulkifli SZ, et al. ABCB1 C3435T polymorphism and the risk of resistance to antiepileptic drugs in epilepsy: a systematic review and meta-analysis. Seizure. 2010; **19**(6): 339-46.
622. Haerian BS, Roslan H, Raymond AA, Tan CT, Lim KS, Zulkifli SZ, et al. ABCB1 C3435T polymorphism and the risk of resistance to antiepileptic drugs in epilepsy: a systematic review and meta-analysis. Seizure. 2010; **19**(6): 339-46.
623. Hagberg JM, Ferrell RE, McCole SD, Wilund KR, Moore GE. VO2 max is associated with ACE genotype in postmenopausal women. J Appl Physiol (1985). 1998; **85**(5): 1842-6.
624. Hagberg JM, Ferrell RE, McCole SD, Wilund KR, Moore GE. VO2 max is associated with ACE genotype in postmenopausal women. Journal Of Applied Physiology (Bethesda, Md: 1985). 1998; **85**(5): 1842-6.
625. Hagberg JM, McCole SD, Ferrell RE, Zmuda JM, Rodgers KS, Wilund KR, et al. Physical activity, hormone replacement therapy and plasma lipoprotein-lipid levels in postmenopausal women. Int J Sports Med. 2003; **24**(1): 22-9.
626. Hagberg JM, McCole SD, Ferrell RE, Zmuda JM, Rodgers KS, Wilund KR, et al. Physical activity, hormone replacement therapy and plasma lipoprotein-lipid levels in postmenopausal women. International Journal Of Sports Medicine. 2003; **24**(1): 22-9.
627. Hakanen M, Raitakari OT, Lehtimaki T, Peltonen N, Pahkala K, Sillanmaki L, et al. FTO genotype is associated with body mass index after the age of seven years but not with energy intake or leisure-time physical activity. J Clin Endocrinol Metab. 2009; **94**(4): 1281-7.
628. Hakanen M, Raitakari OT, Lehtimäki T, Peltonen N, Pahkala K, Sillanmäki L, et

al. FTO genotype is associated with body mass index after the age of seven years but not with energy intake or leisure-time physical activity. The Journal Of Clinical Endocrinology And Metabolism. 2009; **94**(4): 1281-7.
629. Hall KT, Tolkin BR, Chinn GM, Kirsch I, Kelley JM, Lembo AJ, et al. Conscientiousness is modified by genetic variation in catechol-O-methyltransferase to reduce symptom complaints in IBS patients. Brain Behav. 2015; **5**(1): 39-44.
630. Hall KT, Tolkin BR, Chinn GM, Kirsch I, Kelley JM, Lembo AJ, et al. Conscientiousness is modified by genetic variation in catechol-O-methyltransferase to reduce symptom complaints in IBS patients. Brain And Behavior. 2015; **5**(1): 39-44.
631. Halsall DJ, Luan J, Saker P, Huxtable S, Farooqi IS, Keogh J, et al. Uncoupling protein 3 genetic variants in human obesity: the c-55t promoter polymorphism is negatively correlated with body mass index in a UK Caucasian population. Int J Obes Relat Metab Disord. 2001; **25**(4): 472-7.
632. Halsall DJ, Luan J, Saker P, Huxtable S, Farooqi IS, Keogh J, et al. Uncoupling protein 3 genetic variants in human obesity: the c-55t promoter polymorphism is negatively correlated with body mass index in a UK Caucasian population. International Journal Of Obesity And Related Metabolic Disorders: Journal Of The International Association For The Study Of Obesity. 2001; **25**(4): 472-7.
633. Halverstadt A, Phares DA, Ferrell RE, Wilund KR, Goldberg AP, Hagberg JM. High-density lipoprotein-cholesterol, its subfractions, and responses to exercise training are dependent on endothelial lipase genotype. Metabolism. 2003; **52**(11): 1505-11.
634. Halverstadt A, Phares DA, Ferrell RE, Wilund KR, Goldberg AP, Hagberg JM. High-density lipoprotein-cholesterol, its subfractions, and responses to exercise training are dependent on endothelial lipase genotype. Metabolism: Clinical And Experimental. 2003; **52**(11): 1505-11.
635. Hamstra DA, de Kloet ER, Quataert I, Jansen M, Van der Does W. Mineralocorticoid receptor haplotype, estradiol, progesterone and emotional information processing. Psychoneuroendocrinology. 2017; **76**: 162-73.
636. Hamstra DA, de Kloet ER, Quataert I, Jansen M, Van der Does W. Mineralocorticoid receptor haplotype, estradiol, progesterone and emotional information processing. Psychoneuroendocrinology. 2017; **76**: 162-73.
637. Han CJ, Kohen R, Jun S, Jarrett ME, Cain KC, Burr R, et al. COMT Val158Met Polymorphism and Symptom Improvement Following a Cognitively Focused Intervention for Irritable Bowel Syndrome. Nurs Res. 2017; **66**(2): 75-84.
638. Han CJ, Kohen R, Jun S, Jarrett ME, Cain KC, Burr R, et al. COMT Val158Met Polymorphism and Symptom Improvement Following a Cognitively Focused Intervention for Irritable Bowel Syndrome. Nursing Research. 2017; **66**(2): 75-84.
639. Han CZ, Du LL, Jing JX, Zhao XW, Tian FG, Shi J, et al. Associations among lipids, leptin, and leptin receptor gene Gin223Arg polymorphisms and breast cancer in China. Biol Trace Elem Res. 2008; **126**(1-3): 38-48.
640. Han C-Z, Du L-L, Jing J-X, Zhao X-W, Tian F-G, Shi J, et al. Associations among lipids, leptin, and leptin receptor gene Gin223Arg polymorphisms and breast cancer in China. Biological Trace Element Research. 2008; **126**(1-3): 38-48.
641. Hansell NK, Agrawal A, Whitfield JB, Morley KI, Gordon SD, Lind PA, et al. Can we identify genes for alcohol consumption in samples ascertained for heterogeneous

purposes? Alcohol Clin Exp Res. 2009; **33**(4): 729-39.
642. Hansell NK, Agrawal A, Whitfield JB, Morley KI, Gordon SD, Lind PA, et al. Can we identify genes for alcohol consumption in samples ascertained for heterogeneous purposes? Alcoholism, Clinical And Experimental Research. 2009; **33**(4): 729-39.
643. Hansen JS, Zhao X, Irmler M, Liu X, Hoene M, Scheler M, et al. Type 2 diabetes alters metabolic and transcriptional signatures of glucose and amino acid metabolism during exercise and recovery. Diabetologia. 2015; **58**(8): 1845-54.
644. Hansen JS, Zhao X, Irmler M, Liu X, Hoene M, Scheler M, et al. Type 2 diabetes alters metabolic and transcriptional signatures of glucose and amino acid metabolism during exercise and recovery. Diabetologia. 2015; **58**(8): 1845-54.
645. Hansen MV. Chronobiology, cognitive function and depressive symptoms in surgical patients. Dan Med J. 2014; **61**(9): B4914.
646. Hansen MV. Chronobiology, cognitive function and depressive symptoms in surgical patients. Danish Medical Journal. 2014; **61**(9): B4914-B.
647. Hansen MV, Madsen MT, Hageman I, Rasmussen LS, Bokmand S, Rosenberg J, et al. The effect of MELatOnin on Depression, anxietY, cognitive function and sleep disturbances in patients with breast cancer. The MELODY trial: protocol for a randomised, placebo-controlled, double-blinded trial. BMJ Open. 2012; **2**(1): e000647.
648. Hansen MV, Madsen MT, Hageman I, Rasmussen LS, Bokmand S, Rosenberg J, et al. The effect of MELatOnin on Depression, anxietY, cognitive function and sleep disturbances in patients with breast cancer. The MELODY trial: protocol for a randomised, placebo-controlled, double-blinded trial. BMJ Open. 2012; **2**(1): e000647-e.
649. Hara M, Higaki Y, Taguchi N, Shinchi K, Morita E, Naito M, et al. Effect of the PPARG2 Pro12Ala polymorphism and clinical risk factors for diabetes mellitus on HbA1c in the Japanese general population. J Epidemiol. 2012; **22**(6): 523-31.
650. Hara M, Higaki Y, Taguchi N, Shinchi K, Morita E, Naito M, et al. Effect of the PPARG2 Pro12Ala polymorphism and clinical risk factors for diabetes mellitus on HbA1c in the Japanese general population. Journal Of Epidemiology. 2012; **22**(6): 523-31.
651. Hara M, Nakamura K, Nanri H, Nishida Y, Hishida A, Kawai S, et al. Associations between hOGG1 Ser326Cys polymorphism and increased body mass index and fasting glucose level in the Japanese general population. J Epidemiol. 2014; **24**(5): 379-84.
652. Hara M, Nakamura K, Nanri H, Nishida Y, Hishida A, Kawai S, et al. Associations between hOGG1 Ser326Cys polymorphism and increased body mass index and fasting glucose level in the Japanese general population. Journal Of Epidemiology. 2014; **24**(5): 379-84.
653. Hariri AR, Drabant EM, Munoz KE, Kolachana BS, Mattay VS, Egan MF, et al. A susceptibility gene for affective disorders and the response of the human amygdala. Arch Gen Psychiatry. 2005; **62**(2): 146-52.
654. Hariri AR, Drabant EM, Munoz KE, Kolachana BS, Mattay VS, Egan MF, et al. A susceptibility gene for affective disorders and the response of the human amygdala. Archives Of General Psychiatry. 2005; **62**(2): 146-52.
655. Hassan AM, Jain P, Reichmann F, Mayerhofer R, Farzi A, Schuligoi R, et al. Repeated predictable stress causes resilience against colitis-induced behavioral changes in mice. Front Behav Neurosci. 2014; **8**: 386.

656. Hassan AM, Jain P, Reichmann F, Mayerhofer R, Farzi A, Schuligoi R, et al. Repeated predictable stress causes resilience against colitis-induced behavioral changes in mice. Frontiers In Behavioral Neuroscience. 2014; **8**: 386-.
657. Hauer D, Weis F, Papassotiropoulos A, Schmoeckel M, Beiras-Fernandez A, Lieke J, et al. Relationship of a common polymorphism of the glucocorticoid receptor gene to traumatic memories and posttraumatic stress disorder in patients after intensive care therapy. Crit Care Med. 2011; **39**(4): 643-50.
658. Hauer D, Weis F, Papassotiropoulos A, Schmoeckel M, Beiras-Fernandez A, Lieke J, et al. Relationship of a common polymorphism of the glucocorticoid receptor gene to traumatic memories and posttraumatic stress disorder in patients after intensive care therapy. Critical Care Medicine. 2011; **39**(4): 643-50.
659. Hayakawa Y, Yanagi H, Hara S, Amagai H, Endo K, Hamaguchi H, et al. Genetic and environmental factors affecting peak bone mass in premenopausal Japanese women. Environ Health Prev Med. 2001; **6**(3): 177-83.
660. Hayakawa Y, Yanagi H, Hara S, Amagai H, Endo K, Hamaguchi H, et al. Genetic and environmental factors affecting peak bone mass in premenopausal Japanese women. Environmental Health And Preventive Medicine. 2001; **6**(3): 177-83.
661. He Q, Yuan Z, Liu Y, Zhang J, Yan H, Shen L, et al. Correlation between cytochrome P450 2C19 genetic polymorphism and treatment response to escitalopram in panic disorder. Pharmacogenet Genomics. 2017; **27**(8): 279-84.
662. He Q, Yuan Z, Liu Y, Zhang J, Yan H, Shen L, et al. Correlation between cytochrome P450 2C19 genetic polymorphism and treatment response to escitalopram in panic disorder. Pharmacogenetics And Genomics. 2017; **27**(8): 279-84.
663. Hearing CM, Chang WC, Szuhany KL, Deckersbach T, Nierenberg AA, Sylvia LG. Physical Exercise for Treatment of Mood Disorders: A Critical Review. Curr Behav Neurosci Rep. 2016; **3**(4): 350-9.
664. Hearing CM, Chang WC, Szuhany KL, Deckersbach T, Nierenberg AA, Sylvia LG. Physical Exercise for Treatment of Mood Disorders: A Critical Review. Current Behavioral Neuroscience Reports. 2016; **3**(4): 350-9.
665. Heberlein A, Kaser M, Lichtinghagen R, Rhein M, Lenz B, Kornhuber J, et al. TNF-alpha and IL-6 serum levels: neurobiological markers of alcohol consumption in alcohol-dependent patients? Alcohol. 2014; **48**(7): 671-6.
666. Heck DH, Zhao Y, Roy S, LeDoux MS, Reiter LT. Analysis of cerebellar function in Ube3a-deficient mice reveals novel genotype-specific behaviors. Hum Mol Genet. 2008; **17**(14): 2181-9.
667. Heck DH, Zhao Y, Roy S, LeDoux MS, Reiter LT. Analysis of cerebellar function in Ube3a-deficient mice reveals novel genotype-specific behaviors. Human Molecular Genetics. 2008; **17**(14): 2181-9.
668. Hendrickx G, Boudin E, Van Hul W. A look behind the scenes: the risk and pathogenesis of primary osteoporosis. Nat Rev Rheumatol. 2015; **11**(8): 462-74.
669. Hendrickx G, Boudin E, Van Hul W. A look behind the scenes: the risk and pathogenesis of primary osteoporosis. Nature Reviews Rheumatology. 2015; **11**(8): 462-74.
670. Hennis PJ, O'Doherty AF, Levett DZ, Grocott MP, Montgomery HM. Genetic factors associated with exercise performance in atmospheric hypoxia. Sports Med.

2015; **45**(5): 745-61.
671. Hennis PJ, O'Doherty AF, Levett DZH, Grocott MPW, Montgomery HM. Genetic factors associated with exercise performance in atmospheric hypoxia. Sports Medicine (Auckland, NZ). 2015; **45**(5): 745-61.
672. Hiatt RA, Porco TC, Liu F, Balke K, Balmain A, Barlow J, et al. A multilevel model of postmenopausal breast cancer incidence. Cancer Epidemiol Biomarkers Prev. 2014; **23**(10): 2078-92.
673. Hiatt RA, Porco TC, Liu F, Balke K, Balmain A, Barlow J, et al. A multilevel model of postmenopausal breast cancer incidence. Cancer Epidemiology, Biomarkers & Prevention: A Publication Of The American Association For Cancer Research, Cosponsored By The American Society Of Preventive Oncology. 2014; **23**(10): 2078-92.
674. Hicken BL, Calhoun DA, Barton JC, Tucker DC. Attitudes about and psychosocial outcomes of HFE genotyping for hemochromatosis. Genet Test. 2004; **8**(2): 90-7.
675. Hicken BL, Calhoun DA, Barton JC, Tucker DC. Attitudes about and psychosocial outcomes of HFE genotyping for hemochromatosis. Genetic Testing. 2004; **8**(2): 90-7.
676. Hicks JK, Bishop JR, Sangkuhl K, Muller DJ, Ji Y, Leckband SG, et al. Clinical Pharmacogenetics Implementation Consortium (CPIC) Guideline for CYP2D6 and CYP2C19 Genotypes and Dosing of Selective Serotonin Reuptake Inhibitors. Clin Pharmacol Ther. 2015; **98**(2): 127-34.
677. Hicks JK, Bishop JR, Sangkuhl K, Müller DJ, Ji Y, Leckband SG, et al. Clinical Pharmacogenetics Implementation Consortium (CPIC) Guideline for CYP2D6 and CYP2C19 Genotypes and Dosing of Selective Serotonin Reuptake Inhibitors. Clinical Pharmacology And Therapeutics. 2015; **98**(2): 127-34.
678. Hidayatallah N, Silverstein LB, Stolerman M, McDonald T, Walsh CA, Paljevic E, et al. Psychological stress associated with cardiogenetic conditions. Per Med. 2014; **11**(7): 631-40.
679. Hidayatallah N, Silverstein LB, Stolerman M, McDonald T, Walsh CA, Paljevic E, et al. Psychological stress associated with cardiogenetic conditions. Personalized Medicine. 2014; **11**(7): 631-40.
680. Hietaranta-Luoma HL, Tahvonen R, Iso-Touru T, Puolijoki H, Hopia A. An intervention study of individual, apoE genotype-based dietary and physical-activity advice: impact on health behavior. J Nutrigenet Nutrigenomics. 2014; **7**(3): 161-74.
681. Hietaranta-Luoma H-L, Tahvonen R, Iso-Touru T, Puolijoki H, Hopia A. An intervention study of individual, apoE genotype-based dietary and physical-activity advice: impact on health behavior. Journal Of Nutrigenetics And Nutrigenomics. 2014; **7**(3): 161-74.
682. Hill JL, Martinowich K. Activity-dependent signaling: influence on plasticity in circuits controlling fear-related behavior. Curr Opin Neurobiol. 2016; **36**: 59-65.
683. Hill JL, Martinowich K. Activity-dependent signaling: influence on plasticity in circuits controlling fear-related behavior. Current Opinion In Neurobiology. 2016; **36**: 59-65.
684. Hill JO. Understanding and addressing the epidemic of obesity: an energy balance perspective. Endocr Rev. 2006; **27**(7): 750-61.
685. Hill JO. Understanding and addressing the epidemic of obesity: an energy balance perspective. Endocrine Reviews. 2006; **27**(7): 750-61.

686. Hill JO, Melanson EL. Overview of the determinants of overweight and obesity: current evidence and research issues. Med Sci Sports Exerc. 1999; **31**(11 Suppl): S515-21.
687. Hill JO, Melanson EL. Overview of the determinants of overweight and obesity: current evidence and research issues. Medicine And Science In Sports And Exercise. 1999; **31**(11 Suppl): S515-S21.
688. Hiramatsu L, Kay JC, Thompson Z, Singleton JM, Claghorn GC, Albuquerque RL, et al. Maternal exposure to Western diet affects adult body composition and voluntary wheel running in a genotype-specific manner in mice. Physiology & Behavior. 2017; **179**: 235-45.
689. Hiramatsu L, Kay JC, Thompson Z, Singleton JM, Claghorn GC, Albuquerque RL, et al. Maternal exposure to Western diet affects adult body composition and voluntary wheel running in a genotype-specific manner in mice. Physiol Behav. 2017; **179**: 235-45.
690. Hirsch I, Suchankova A, Zavadova H, Vonka V. Study of Epstein-Barr virus-determined nuclear antigen (EBNA) by chromatography on fixed cell nuclei. Int J Cancer. 1978; **22**(5): 535-41.
691. Hirsch I, Suchánková A, Závadová H, Vonka V. Study of Epstein-Barr virus-determined nuclear antigen (EBNA) by chromatography on fixed cell nuclei. International Journal Of Cancer. 1978; **22**(5): 535-41.
692. Ho SB, Monto A, Peyton A, Kaplan DE, Byrne S, Moon S, et al. Efficacy of Sofosbuvir Plus Ribavirin in Veterans With Hepatitis C Virus Genotype 2 Infection, Compensated Cirrhosis, and Multiple Comorbidities. Clin Gastroenterol Hepatol. 2017; **15**(2): 282-8.
693. Ho SB, Monto A, Peyton A, Kaplan DE, Byrne S, Moon S, et al. Efficacy of Sofosbuvir Plus Ribavirin in Veterans With Hepatitis C Virus Genotype 2 Infection, Compensated Cirrhosis, and Multiple Comorbidities. Clinical Gastroenterology And Hepatology: The Official Clinical Practice Journal Of The American Gastroenterological Association. 2017; **15**(2): 282-8.
694. Hochstatter KR, Stockman LJ, Holzmacher R, Greer J, Seal DW, Taylor QA, et al. The continuum of hepatitis C care for criminal justice involved adults in the DAA era: a retrospective cohort study demonstrating limited treatment uptake and inconsistent linkage to community-based care. Health Justice. 2017; **5**(1): 10.
695. Hochstatter KR, Stockman LJ, Holzmacher R, Greer J, Seal DW, Taylor QA, et al. The continuum of hepatitis C care for criminal justice involved adults in the DAA era: a retrospective cohort study demonstrating limited treatment uptake and inconsistent linkage to community-based care. Health & Justice. 2017; **5**(1): 10-.
696. Hoeffer CA, Wong H, Cain P, Levenga J, Cowansage KK, Choi Y, et al. Regulator of calcineurin 1 modulates expression of innate anxiety and anxiogenic responses to selective serotonin reuptake inhibitor treatment. J Neurosci. 2013; **33**(43): 16930-44.
697. Hoeffer CA, Wong H, Cain P, Levenga J, Cowansage KK, Choi Y, et al. Regulator of calcineurin 1 modulates expression of innate anxiety and anxiogenic responses to selective serotonin reuptake inhibitor treatment. The Journal Of Neuroscience: The Official Journal Of The Society For Neuroscience. 2013; **33**(43): 16930-44.
698. Hoffman EP, Rao D, Pachman LM. Clarifying the boundaries between the

inflammatory and dystrophic myopathies: insights from molecular diagnostics and microarrays. Rheum Dis Clin North Am. 2002; **28**(4): 743-57.
699. Hoffman EP, Rao D, Pachman LM. Clarifying the boundaries between the inflammatory and dystrophic myopathies: insights from molecular diagnostics and microarrays. Rheumatic Diseases Clinics Of North America. 2002; **28**(4): 743-57.
700. Holmes JA, Desmond PV, Thompson AJ. Does IL28B genotyping still have a role in the era of direct-acting antiviral therapy for chronic hepatitis C infection? J Viral Hepat. 2012; **19**(10): 677-84.
701. Holmes JA, Desmond PV, Thompson AJ. Does IL28B genotyping still have a role in the era of direct-acting antiviral therapy for chronic hepatitis C infection? Journal Of Viral Hepatitis. 2012; **19**(10): 677-84.
702. Holmes PV. Trophic Mechanisms for Exercise-Induced Stress Resilience: Potential Role of Interactions between BDNF and Galanin. Front Psychiatry. 2014; **5**: 90.
703. Holmes PV. Trophic Mechanisms for Exercise-Induced Stress Resilience: Potential Role of Interactions between BDNF and Galanin. Frontiers In Psychiatry. 2014; **5**: 90-.
704. Holzer P. Neuropeptides, Microbiota, and Behavior. Int Rev Neurobiol. 2016; **131**: 67-89.
705. Holzer P. Neuropeptides, Microbiota, and Behavior. International Review Of Neurobiology. 2016; **131**: 67-89.
706. Homberg JR, Karel P, Verheij MM. Individual differences in cocaine addiction: maladaptive behavioural traits. Addict Biol. 2014; **19**(4): 517-28.
707. Homberg JR, Karel P, Verheij MMM. Individual differences in cocaine addiction: maladaptive behavioural traits. Addiction Biology. 2014; **19**(4): 517-28.
708. Hommers LG, Domschke K, Deckert J. Heterogeneity and individuality: microRNAs in mental disorders. J Neural Transm (Vienna). 2015; **122**(1): 79-97.
709. Hommers LG, Domschke K, Deckert J. Heterogeneity and individuality: microRNAs in mental disorders. Journal Of Neural Transmission (Vienna, Austria: 1996). 2015; **122**(1): 79-97.
710. Hong LE, Hodgkinson CA, Yang Y, Sampath H, Ross TJ, Buchholz B, et al. A genetically modulated, intrinsic cingulate circuit supports human nicotine addiction. Proc Natl Acad Sci U S A. 2010; **107**(30): 13509-14.
711. Hong LE, Hodgkinson CA, Yang Y, Sampath H, Ross TJ, Buchholz B, et al. A genetically modulated, intrinsic cingulate circuit supports human nicotine addiction. Proceedings Of The National Academy Of Sciences Of The United States Of America. 2010; **107**(30): 13509-14.
712. Hoque MO. DNA methylation changes in prostate cancer: current developments and future clinical implementation. Expert Rev Mol Diagn. 2009; **9**(3): 243-57.
713. Hoque MO. DNA methylation changes in prostate cancer: current developments and future clinical implementation. Expert Review Of Molecular Diagnostics. 2009; **9**(3): 243-57.
714. Horie NC, Serrao VT, Simon SS, Gascon MR, Dos Santos AX, Zambone MA, et al. Cognitive Effects of Intentional Weight Loss in Elderly Obese Individuals With Mild Cognitive Impairment. J Clin Endocrinol Metab. 2016; **101**(3): 1104-12.

715. Horie NC, Serrao VT, Simon SS, Gascon MRP, Dos Santos AX, Zambone MA, et al. Cognitive Effects of Intentional Weight Loss in Elderly Obese Individuals With Mild Cognitive Impairment. The Journal Of Clinical Endocrinology And Metabolism. 2016; **101**(3): 1104-12.
716. Hou L, Qi Y, Sun H, Wang G, Li Q, Wang Y, et al. Applying ketamine to alleviate the PTSD-like effects by regulating the HCN1-related BDNF. Prog Neuropsychopharmacol Biol Psychiatry. 2018.
717. Hou L, Qi Y, Sun H, Wang G, Li Q, Wang Y, et al. Applying ketamine to alleviate the PTSD-like effects by regulating the HCN1-related BDNF. Progress In Neuro-Psychopharmacology & Biological Psychiatry. 2018; **86**: 313-21.
718. Hoybye C. Endocrine and metabolic aspects of adult Prader-Willi syndrome with special emphasis on the effect of growth hormone treatment. Growth Horm IGF Res. 2004; **14**(1): 1-15.
719. Höybye C. Endocrine and metabolic aspects of adult Prader-Willi syndrome with special emphasis on the effect of growth hormone treatment. Growth Hormone & IGF Research: Official Journal Of The Growth Hormone Research Society And The International IGF Research Society. 2004; **14**(1): 1-15.
720. Hoybye C, Thoren M. Somatropin therapy in adults with Prader-Willi syndrome. Treat Endocrinol. 2004; **3**(3): 153-60.
721. Höybye C, Thorén M. Somatropin therapy in adults with Prader-Willi syndrome. Treatments In Endocrinology. 2004; **3**(3): 153-60.
722. Hoyer D, Bartfai T. Neuropeptides and neuropeptide receptors: drug targets, and peptide and non-peptide ligands: a tribute to Prof. Dieter Seebach. Chem Biodivers. 2012; **9**(11): 2367-87.
723. Hoyer D, Bartfai T. Neuropeptides and neuropeptide receptors: drug targets, and peptide and non-peptide ligands: a tribute to Prof. Dieter Seebach. Chemistry & Biodiversity. 2012; **9**(11): 2367-87.
724. Hruba D. [Smoking and breast cancer]. Klin Onkol. 2013; **26**(6): 389-93.
725. Hrubá D. [Smoking and breast cancer]. Klinicka Onkologie: Casopis Ceske A Slovenske Onkologicke Spolecnosti. 2013; **26**(6): 389-93.
726. Hsu YH, Xu X, Terwedow HA, Niu T, Hong X, Wu D, et al. Large-scale genome-wide linkage analysis for loci linked to BMD at different skeletal sites in extreme selected sibships. J Bone Miner Res. 2007; **22**(2): 184-94.
727. Hsu Y-H, Xu X, Terwedow HA, Niu T, Hong X, Wu D, et al. Large-scale genome-wide linkage analysis for loci linked to BMD at different skeletal sites in extreme selected sibships. Journal Of Bone And Mineral Research: The Official Journal Of The American Society For Bone And Mineral Research. 2007; **22**(2): 184-94.
728. Hu DS, Xie J, Yu DH, Xu GH, Lu J, Yang JX, et al. Perilipin gene 1237 T > C polymorphism is not associated with obesity risk in northern Chinese Han adults. Biomed Environ Sci. 2009; **22**(5): 442-7.
729. Hu D-S, Xie J, Yu D-H, Xu G-H, Lu J, Yang J-X, et al. Perilipin gene 1237 T > C polymorphism is not associated with obesity risk in northern Chinese Han adults. Biomedical And Environmental Sciences: BES. 2009; **22**(5): 442-7.
730. Hu FB. Globalization of diabetes: the role of diet, lifestyle, and genes. Diabetes Care. 2011; **34**(6): 1249-57.

731. Hu FB. Globalization of diabetes: the role of diet, lifestyle, and genes. Diabetes Care. 2011; **34**(6): 1249-57.
732. Huang CI, Lin LC, Tien HC, Que J, Ting WC, Chen PC, et al. Hyperlipidemia and statins use for the risk of new-onset anxiety/depression in patients with head and neck cancer: A population-based study. PLoS One. 2017; **12**(3): e0174574.
733. Huang C-I, Lin L-C, Tien H-C, Que J, Ting WC, Chen P-C, et al. Hyperlipidemia and statins use for the risk of new-onset anxiety/depression in patients with head and neck cancer: A population-based study. PLoS One. 2017; **12**(3): e0174574-e.
734. Huang T, Tucker KL, Lee YC, Crott JW, Parnell LD, Shen J, et al. Interactions between genetic variants of folate metabolism genes and lifestyle affect plasma homocysteine concentrations in the Boston Puerto Rican population. Public Health Nutr. 2011; **14**(10): 1805-12.
735. Huang T, Tucker KL, Lee Y-C, Crott JW, Parnell LD, Shen J, et al. Interactions between genetic variants of folate metabolism genes and lifestyle affect plasma homocysteine concentrations in the Boston Puerto Rican population. Public Health Nutrition. 2011; **14**(10): 1805-12.
736. Hubacek JA, Pikhart H, Kubinova R, Peasey A, Malyutina S, Pajak A, et al. Lack of Association between NYD-SP18 Variant and Obesity. The Health Alcohol and Psychosocial Factors in Eastern Europe Study. Ann Nutr Metab. 2016; **68**(4): 244-8.
737. Hubacek JA, Pikhart H, Kubinova R, Peasey A, Malyutina S, Pajak A, et al. Lack of Association between NYD-SP18 Variant and Obesity. The Health Alcohol and Psychosocial Factors in Eastern Europe Study. Annals Of Nutrition & Metabolism. 2016; **68**(4): 244-8.
738. Huber SE, Zoicas I, Reichel M, Muhle C, Buttner C, Ekici AB, et al. Prenatal androgen receptor activation determines adult alcohol and water drinking in a sex-specific way. Addict Biol. 2017.
739. Huber SE, Zoicas I, Reichel M, Mühle C, Büttner C, Ekici AB, et al. Prenatal androgen receptor activation determines adult alcohol and water drinking in a sex-specific way. Addiction Biology. 2018; **23**(3): 904-20.
740. Hughes RA, Pritchard J, Hadden RD. Pharmacological treatment other than corticosteroids, intravenous immunoglobulin and plasma exchange for Guillain Barré syndrome. The Cochrane Database Of Systematic Reviews. 2011; (3): CD008630.
741. Hughes RA, Pritchard J, Hadden RD. Pharmacological treatment other than corticosteroids, intravenous immunoglobulin and plasma exchange for Guillain-Barre syndrome. Cochrane Database Syst Rev. 2013; (2): CD008630.
742. Huh JY. The role of exercise-induced myokines in regulating metabolism. Arch Pharm Res. 2018; **41**(1): 14-29.
743. Huh JY. The role of exercise-induced myokines in regulating metabolism. Archives Of Pharmacal Research. 2018; **41**(1): 14-29.
744. Hull M, Klein M, Shafran S, Tseng A, Giguere P, Cote P, et al. CIHR Canadian HIV Trials Network Coinfection and Concurrent Diseases Core: Canadian guidelines for management and treatment of HIV/hepatitis C coinfection in adults. Can J Infect Dis Med Microbiol. 2013; **24**(4): 217-38.
745. Hull M, Klein M, Shafran S, Tseng A, Giguère P, Côté P, et al. CIHR Canadian HIV Trials Network Coinfection and Concurrent Diseases Core: Canadian guidelines for

management and treatment of HIV/hepatitis C coinfection in adults. The Canadian Journal Of Infectious Diseases & Medical Microbiology = Journal Canadien Des Maladies Infectieuses Et De La Microbiologie Medicale. 2013; **24**(4): 217-38.
746. Hunnerkopf R, Strobel A, Gutknecht L, Brocke B, Lesch KP. Interaction between BDNF Val66Met and dopamine transporter gene variation influences anxiety-related traits. Neuropsychopharmacology. 2007; **32**(12): 2552-60.
747. Hünnerkopf R, Strobel A, Gutknecht L, Brocke B, Lesch KP. Interaction between BDNF Val66Met and dopamine transporter gene variation influences anxiety-related traits. Neuropsychopharmacology: Official Publication Of The American College Of Neuropsychopharmacology. 2007; **32**(12): 2552-60.
748. Hurley BF, Hanson ED, Sheaff AK. Strength training as a countermeasure to aging muscle and chronic disease. Sports Med. 2011; **41**(4): 289-306.
749. Hurley BF, Hanson ED, Sheaff AK. Strength training as a countermeasure to aging muscle and chronic disease. Sports Medicine (Auckland, NZ). 2011; **41**(4): 289-306.
750. Husemoen LL, Linneberg A, Fenger M, Thuesen BH, Jorgensen T. Changes in lifestyle, biological risk factors and total homocysteine in relation to MTHFR C677T genotype: a 5-year follow-up study. Eur J Clin Nutr. 2009; **63**(10): 1233-40.
751. Husemoen LL, Thomsen TF, Fenger M, Jorgensen T. Effect of lifestyle factors on plasma total homocysteine concentrations in relation to MTHFR(C677T) genotype. Inter99 (7). Eur J Clin Nutr. 2004; **58**(8): 1142-50.
752. Husemoen LL, Thomsen TF, Fenger M, Jorgensen T. Changes in lifestyle and total homocysteine in relation to MTHFR(C677T) genotype: the Inter99 study. Eur J Clin Nutr. 2006; **60**(5): 614-22.
753. Husemoen LLN, Linneberg A, Fenger M, Thuesen BH, Jørgensen T. Changes in lifestyle, biological risk factors and total homocysteine in relation to MTHFR C677T genotype: a 5-year follow-up study. European Journal Of Clinical Nutrition. 2009; **63**(10): 1233-40.
754. Husemoen LLN, Thomsen TF, Fenger M, Jørgensen T. Effect of lifestyle factors on plasma total homocysteine concentrations in relation to MTHFR(C677T) genotype. Inter99 (7). European Journal Of Clinical Nutrition. 2004; **58**(8): 1142-50.
755. Husemoen LLN, Thomsen TF, Fenger M, Jørgensen T. Changes in lifestyle and total homocysteine in relation to MTHFR(C677T) genotype: the Inter99 study. European Journal Of Clinical Nutrition. 2006; **60**(5): 614-22.
756. Huuskonen A, Lappalainen J, Oksala N, Santtila M, Hakkinen K, Kyrolainen H, et al. Common genetic variation in the IGF1 associates with maximal force output. Med Sci Sports Exerc. 2011; **43**(12): 2368-74.
757. Huuskonen A, Lappalainen J, Oksala N, Santtila M, Hakkinen K, Kyrolainen H, et al. Aerobic fitness does not modify the effect of FTO variation on body composition traits. PLoS One. 2012; **7**(12): e51635.
758. Huuskonen A, Lappalainen J, Oksala N, Santtila M, Häkkinen K, Kyröläinen H, et al. Common genetic variation in the IGF1 associates with maximal force output. Medicine And Science In Sports And Exercise. 2011; **43**(12): 2368-74.
759. Huuskonen A, Lappalainen J, Oksala N, Santtila M, Häkkinen K, Kyröläinen H, et al. Aerobic fitness does not modify the effect of FTO variation on body composition

traits. PLoS One. 2012; **7**(12): e51635-e.
760. Hypponen E, Berry DJ, Wjst M, Power C. Serum 25-hydroxyvitamin D and IgE - a significant but nonlinear relationship. Allergy. 2009; **64**(4): 613-20.
761. Hyppönen E, Berry DJ, Wjst M, Power C. Serum 25-hydroxyvitamin D and IgE - a significant but nonlinear relationship. Allergy. 2009; **64**(4): 613-20.
762. Idewaki Y, Iwase M, Fujii H, Ohkuma T, Ide H, Kaizu S, et al. Association of Genetically Determined Aldehyde Dehydrogenase 2 Activity with Diabetic Complications in Relation to Alcohol Consumption in Japanese Patients with Type 2 Diabetes Mellitus: The Fukuoka Diabetes Registry. PLoS One. 2015; **10**(11): e0143288.
763. Idewaki Y, Iwase M, Fujii H, Ohkuma T, Ide H, Kaizu S, et al. Association of Genetically Determined Aldehyde Dehydrogenase 2 Activity with Diabetic Complications in Relation to Alcohol Consumption in Japanese Patients with Type 2 Diabetes Mellitus: The Fukuoka Diabetes Registry. PLoS One. 2015; **10**(11): e0143288-e.
764. Iki M, Saito Y, Dohi Y, Kajita E, Nishino H, Yonemasu K, et al. Greater trunk muscle torque reduces postmenopausal bone loss at the spine independently of age, body size, and vitamin D receptor genotype in Japanese women. Calcif Tissue Int. 2002; **71**(4): 300-7.
765. Iki M, Saito Y, Dohi Y, Kajita E, Nishino H, Yonemasu K, et al. Greater trunk muscle torque reduces postmenopausal bone loss at the spine independently of age, body size, and vitamin D receptor genotype in Japanese women. Calcified Tissue International. 2002; **71**(4): 300-7.
766. Ilvesaro J, Pohjanvirta R, Tuomisto J, Viluksela M, Tuukkanen J. Bone resorption by aryl hydrocarbon receptor-expressing osteoclasts is not disturbed by TCDD in short-term cultures. Life Sci. 2005; **77**(12): 1351-66.
767. Ilvesaro J, Pohjanvirta R, Tuomisto J, Viluksela M, Tuukkanen J. Bone resorption by aryl hydrocarbon receptor-expressing osteoclasts is not disturbed by TCDD in short-term cultures. Life Sciences. 2005; **77**(12): 1351-66.
768. Itkin M, Davidovich-Rikanati R, Cohen S, Portnoy V, Doron-Faigenboim A, Oren E, et al. The biosynthetic pathway of the nonsugar, high-intensity sweetener mogroside V from Siraitia grosvenorii. Proc Natl Acad Sci U S A. 2016; **113**(47): E7619-E28.
769. Itkin M, Davidovich-Rikanati R, Cohen S, Portnoy V, Doron-Faigenboim A, Oren E, et al. The biosynthetic pathway of the nonsugar, high-intensity sweetener mogroside V from Siraitia grosvenorii. Proceedings Of The National Academy Of Sciences Of The United States Of America. 2016; **113**(47): E7619-E28.
770. Iwasaki Y, Ishikawa M, Okada N, Koizumi S. Induction of a distinct morphology and signal transduction in TrkB/PC12 cells by nerve growth factor and brain-derived neurotrophic factor. J Neurochem. 1997; **68**(3): 927-34.
771. Iwasaki Y, Ishikawa M, Okada N, Koizumi S. Induction of a distinct morphology and signal transduction in TrkB/PC12 cells by nerve growth factor and brain-derived neurotrophic factor. Journal Of Neurochemistry. 1997; **68**(3): 927-34.
772. Jaakkola U, Kuusela T, Jartti T, Pesonen U, Koulu M, Vahlberg T, et al. The Leu7Pro polymorphism of preproNPY is associated with decreased insulin secretion, delayed ghrelin suppression, and increased cardiovascular responsiveness to norepinephrine during oral glucose tolerance test. J Clin Endocrinol Metab. 2005; **90**(6): 3646-52.

773. Jaakkola U, Kuusela T, Jartti T, Pesonen U, Koulu M, Vahlberg T, et al. The Leu7Pro polymorphism of preproNPY is associated with decreased insulin secretion, delayed ghrelin suppression, and increased cardiovascular responsiveness to norepinephrine during oral glucose tolerance test. The Journal Of Clinical Endocrinology And Metabolism. 2005; **90**(6): 3646-52.
774. Jabbi M, Chen Q, Turner N, Kohn P, White M, Kippenhan JS, et al. Variation in the Williams syndrome GTF2I gene and anxiety proneness interactively affect prefrontal cortical response to aversive stimuli. Transl Psychiatry. 2015; **5**: e622.
775. Jabbi M, Chen Q, Turner N, Kohn P, White M, Kippenhan JS, et al. Variation in the Williams syndrome GTF2I gene and anxiety proneness interactively affect prefrontal cortical response to aversive stimuli. Translational Psychiatry. 2015; **5**: e622-e.
776. Jacobson TA. Lipoprotein(a), cardiovascular disease, and contemporary management. Mayo Clin Proc. 2013; **88**(11): 1294-311.
777. Jacobson TA. Lipoprotein(a), cardiovascular disease, and contemporary management. Mayo Clinic Proceedings. 2013; **88**(11): 1294-311.
778. Jaehne EJ, Ameti D, Paiva T, van den Buuse M. Investigating the Role of Serotonin in Methamphetamine Psychosis: Unaltered Behavioral Effects of Chronic Methamphetamine in 5-HT1A Knockout Mice. Front Psychiatry. 2017; **8**: 61.
779. Jaehne EJ, Ameti D, Paiva T, van den Buuse M. Investigating the Role of Serotonin in Methamphetamine Psychosis: Unaltered Behavioral Effects of Chronic Methamphetamine in 5-HT1A Knockout Mice. Frontiers In Psychiatry. 2017; **8**: 61-.
780. Jani R, Coakley K, Douglas T, Singh R. Protein intake and physical activity are associated with body composition in individuals with phenylalanine hydroxylase deficiency. Mol Genet Metab. 2017; **121**(2): 104-10.
781. Jani R, Coakley K, Douglas T, Singh R. Protein intake and physical activity are associated with body composition in individuals with phenylalanine hydroxylase deficiency. Molecular Genetics And Metabolism. 2017; **121**(2): 104-10.
782. Jansen D, Zerbi V, Janssen CI, van Rooij D, Zinnhardt B, Dederen PJ, et al. Impact of a multi-nutrient diet on cognition, brain metabolism, hemodynamics, and plasticity in apoE4 carrier and apoE knockout mice. Brain Struct Funct. 2014; **219**(5): 1841-68.
783. Jansen D, Zerbi V, Janssen CIF, van Rooïj D, Zinnhardt B, Dederen PJ, et al. Impact of a multi-nutrient diet on cognition, brain metabolism, hemodynamics, and plasticity in apoE4 carrier and apoE knockout mice. Brain Structure & Function. 2014; **219**(5): 1841-68.
784. Jarrett ME, Kohen R, Cain KC, Burr RL, Poppe A, Navaja GP, et al. Relationship of SERT polymorphisms to depressive and anxiety symptoms in irritable bowel syndrome. Biol Res Nurs. 2007; **9**(2): 161-9.
785. Jarrett ME, Kohen R, Cain KC, Burr RL, Poppe A, Navaja GP, et al. Relationship of SERT polymorphisms to depressive and anxiety symptoms in irritable bowel syndrome. Biological Research For Nursing. 2007; **9**(2): 161-9.
786. Jefferson A, Fyfe S, Downs J, Woodhead H, Jacoby P, Leonard H. Longitudinal bone mineral content and density in Rett syndrome and their contributing factors. Bone. 2015; **74**: 191-8.
787. Jefferson A, Fyfe S, Downs J, Woodhead H, Jacoby P, Leonard H. Longitudinal

bone mineral content and density in Rett syndrome and their contributing factors. Bone. 2015; **74**: 191-8.
788. Jellema A, Mensink RP, Kromhout D, Saris WH, Feskens EJ. Metabolic risk markers in an overweight and normal weight population with oversampling of carriers of the IRS-1 972Arg-variant. Atherosclerosis. 2003; **171**(1): 75-81.
789. Jellema A, Mensink RP, Kromhout D, Saris WHM, Feskens EJM. Metabolic risk markers in an overweight and normal weight population with oversampling of carriers of the IRS-1 972Arg-variant. Atherosclerosis. 2003; **171**(1): 75-81.
790. Jenner P. An overview of adenosine A2A receptor antagonists in Parkinson's disease. Int Rev Neurobiol. 2014; **119**: 71-86.
791. Jenner P. An overview of adenosine A2A receptor antagonists in Parkinson's disease. International Review Of Neurobiology. 2014; **119**: 71-86.
792. Jeon HJ, Kang ES, Lee EH, Jeong EG, Jeon JR, Mischoulon D, et al. Childhood trauma and platelet brain-derived neurotrophic factor (BDNF) after a three month follow-up in patients with major depressive disorder. J Psychiatr Res. 2012; **46**(7): 966-72.
793. Jeon HJ, Kang E-S, Lee EH, Jeong E-G, Jeon J-R, Mischoulon D, et al. Childhood trauma and platelet brain-derived neurotrophic factor (BDNF) after a three month follow-up in patients with major depressive disorder. Journal Of Psychiatric Research. 2012; **46**(7): 966-72.
794. Jeong YH, Bliden KP, Shuldiner AR, Tantry US, Gurbel PA. Thrombin-induced platelet-fibrin clot strength: relation to high on-clopidogrel platelet reactivity, genotype, and post-percutaneous coronary intervention outcomes. Thromb Haemost. 2014; **111**(4): 713-24.
795. Jeong Y-H, Bliden KP, Shuldiner AR, Tantry US, Gurbel PA. Thrombin-induced platelet-fibrin clot strength: relation to high on-clopidogrel platelet reactivity, genotype, and post-percutaneous coronary intervention outcomes. Thrombosis And Haemostasis. 2014; **111**(4): 713-24.
796. Jetten MS, Cirpus I, Kartal B, van Niftrik L, van de Pas-Schoonen KT, Sliekers O, et al. 1994-2004: 10 years of research on the anaerobic oxidation of ammonium. Biochem Soc Trans. 2005; **33**(Pt 1): 119-23.
797. Jetten MSM, Cirpus I, Kartal B, van Niftrik L, van de Pas-Schoonen KT, Sliekers O, et al. 1994-2004: 10 years of research on the anaerobic oxidation of ammonium. Biochemical Society Transactions. 2005; **33**(Pt 1): 119-23.
798. Jiang T, Yu JT, Tian Y, Tan L. Epidemiology and etiology of Alzheimer's disease: from genetic to non-genetic factors. Curr Alzheimer Res. 2013; **10**(8): 852-67.
799. Jiang T, Yu J-T, Tian Y, Tan L. Epidemiology and etiology of Alzheimer's disease: from genetic to non-genetic factors. Current Alzheimer Research. 2013; **10**(8): 852-67.
800. Jiao K, Sahaboglu A, Zrenner E, Ueffing M, Ekstrom PA, Paquet-Durand F. Efficacy of PARP inhibition in Pde6a mutant mouse models for retinitis pigmentosa depends on the quality and composition of individual human mutations. Cell Death Discov. 2016; **2**: 16040.
801. Jiao K, Sahaboglu A, Zrenner E, Ueffing M, Ekström PAR, Paquet-Durand F. Efficacy of PARP inhibition in Pde6a mutant mouse models for retinitis pigmentosa depends on the quality and composition of individual human mutations. Cell Death Discovery. 2016; **2**: 16040-.

802. JiaWen W, Hong S, ShengXiang X, Jing L. Depression- and anxiety-like behaviour is related to BDNF/TrkB signalling in a mouse model of psoriasis. Clin Exp Dermatol. 2018; **43**(3): 254-61.
803. JiaWen W, Hong S, ShengXiang X, Jing L. Depression- and anxiety-like behaviour is related to BDNF/TrkB signalling in a mouse model of psoriasis. Clinical And Experimental Dermatology. 2018; **43**(3): 254-61.
804. Jimenez-Chillaron JC, Nijland MJ, Ascensao AA, Sardao VA, Magalhaes J, Hitchler MJ, et al. Back to the future: transgenerational transmission of xenobiotic-induced epigenetic remodeling. Epigenetics. 2015; **10**(4): 259-73.
805. Jiménez-Chillarón JC, Nijland MJ, Ascensão AA, Sardão VA, Magalhães J, Hitchler MJ, et al. Back to the future: transgenerational transmission of xenobiotic-induced epigenetic remodeling. Epigenetics. 2015; **10**(4): 259-73.
806. Jin K, Shen J, Ashton RW, White RP, Dodd IC, Phillips AL, et al. The effect of impedance to root growth on plant architecture in wheat. Plant Soil. 2015; **392**(1-2): 323-32.
807. Jin K, Shen J, Ashton RW, White RP, Dodd IC, Phillips AL, et al. The effect of impedance to root growth on plant architecture in wheat. Plant And Soil. 2015; **392**(1-2): 323-32.
808. Jin UH, Park H, Li X, Davidson LA, Allred C, Patil B, et al. Structure-Dependent Modulation of Aryl Hydrocarbon Receptor-Mediated Activities by Flavones. Toxicol Sci. 2018.
809. Joe KH, Kim YK, Kim TS, Roh SW, Choi SW, Kim YB, et al. Decreased plasma brain-derived neurotrophic factor levels in patients with alcohol dependence. Alcohol Clin Exp Res. 2007; **31**(11): 1833-8.
810. Joe K-H, Kim Y-K, Kim T-S, Roh S-W, Choi S-W, Kim Y-B, et al. Decreased plasma brain-derived neurotrophic factor levels in patients with alcohol dependence. Alcoholism, Clinical And Experimental Research. 2007; **31**(11): 1833-8.
811. Johnson DC, Casey BJ. Easy to remember, difficult to forget: the development of fear regulation. Dev Cogn Neurosci. 2015; **11**: 42-55.
812. Johnson DC, Casey BJ. Easy to remember, difficult to forget: the development of fear regulation. Developmental Cognitive Neuroscience. 2015; **11**: 42-55.
813. Johnson ML, Lara N, Kamel MA. How genomics has informed our understanding of the pathogenesis of osteoporosis. Genome Med. 2009; **1**(9): 84.
814. Johnson ML, Lara N, Kamel MA. How genomics has informed our understanding of the pathogenesis of osteoporosis. Genome Medicine. 2009; **1**(9): 84-.
815. Jolliffe DA, James WY, Hooper RL, Barnes NC, Greiller CL, Islam K, et al. Prevalence, determinants and clinical correlates of vitamin D deficiency in patients with Chronic Obstructive Pulmonary Disease in London, UK. J Steroid Biochem Mol Biol. 2018; **175**: 138-45.
816. Jolliffe DA, James WY, Hooper RL, Barnes NC, Greiller CL, Islam K, et al. Prevalence, determinants and clinical correlates of vitamin D deficiency in patients with Chronic Obstructive Pulmonary Disease in London, UK. The Journal Of Steroid Biochemistry And Molecular Biology. 2018; **175**: 138-45.
817. Jozkow P, Slowinska-Lisowska M, Laczmanski L, Jakubiec D, Medras M. Melanocortin-4 receptor gene polymorphism and the level of physical activity in men

(HALS Study). Endocrine. 2011; **39**(1): 62-8.
818. Jóźków P, Słowińska-Lisowska M, Łaczmański Ł, Jakubiec D, Mędraś M. Melanocortin-4 receptor gene polymorphism and the level of physical activity in men (HALS Study). Endocrine. 2011; **39**(1): 62-8.
819. Ju LS, Yang JJ, Lei L, Xia JY, Luo D, Ji MH, et al. The Combination of Long-term Ketamine and Extinction Training Contributes to Fear Erasure by Bdnf Methylation. Front Cell Neurosci. 2017; **11**: 100.
820. Ju L-S, Yang J-J, Lei L, Xia J-Y, Luo D, Ji M-H, et al. The Combination of Long-term Ketamine and Extinction Training Contributes to Fear Erasure by Bdnf Methylation. Frontiers In Cellular Neuroscience. 2017; **11**: 100-.
821. Jugessur A, Lie RT, Wilcox AJ, Murray JC, Taylor JA, Saugstad OD, et al. Cleft palate, transforming growth factor alpha gene variants, and maternal exposures: assessing gene-environment interactions in case-parent triads. Genet Epidemiol. 2003; **25**(4): 367-74.
822. Jugessur A, Lie RT, Wilcox AJ, Murray JC, Taylor JA, Saugstad OD, et al. Cleft palate, transforming growth factor alpha gene variants, and maternal exposures: assessing gene-environment interactions in case-parent triads. Genetic Epidemiology. 2003; **25**(4): 367-74.
823. Jung AY, van Duijnhoven FJ, Nagengast FM, Botma A, Heine-Broring RC, Kleibeuker JH, et al. Dietary B vitamin and methionine intake and MTHFR C677T genotype on risk of colorectal tumors in Lynch syndrome: the GEOLynch cohort study. Cancer Causes Control. 2014; **25**(9): 1119-29.
824. Jung AY, van Duijnhoven FJB, Nagengast FM, Botma A, Heine-Bröring RC, Kleibeuker JH, et al. Dietary B vitamin and methionine intake and MTHFR C677T genotype on risk of colorectal tumors in Lynch syndrome: the GEOLynch cohort study. Cancer Causes & Control: CCC. 2014; **25**(9): 1119-29.
825. Jung J, Tawa EA, Muench C, Rosen AD, Rickels K, Lohoff FW. Genome-wide association study of treatment response to venlafaxine XR in generalized anxiety disorder. Psychiatry Res. 2017; **254**: 8-11.
826. Jung J, Tawa EA, Muench C, Rosen AD, Rickels K, Lohoff FW. Genome-wide association study of treatment response to venlafaxine XR in generalized anxiety disorder. Psychiatry Research. 2017; **254**: 8-11.
827. Jungbauer A, Medjakovic S. Phytoestrogens and the metabolic syndrome. J Steroid Biochem Mol Biol. 2014; **139**: 277-89.
828. Jungbauer A, Medjakovic S. Phytoestrogens and the metabolic syndrome. The Journal Of Steroid Biochemistry And Molecular Biology. 2014; **139**: 277-89.
829. Juonala M, Viikari JS, Kahonen M, Taittonen L, Laitinen T, Hutri-Kahonen N, et al. Life-time risk factors and progression of carotid atherosclerosis in young adults: the Cardiovascular Risk in Young Finns study. Eur Heart J. 2010; **31**(14): 1745-51.
830. Juonala M, Viikari JSA, Kähönen M, Taittonen L, Laitinen T, Hutri-Kähönen N, et al. Life-time risk factors and progression of carotid atherosclerosis in young adults: the Cardiovascular Risk in Young Finns study. European Heart Journal. 2010; **31**(14): 1745-51.
831. Justesen JM, Allin KH, Sandholt CH, Borglykke A, Krarup NT, Grarup N, et al. Interactions of Lipid Genetic Risk Scores With Estimates of Metabolic Health in a

Danish Population. Circ Cardiovasc Genet. 2015; **8**(3): 465-72.
832. Justesen JM, Allin KH, Sandholt CH, Borglykke A, Krarup NT, Grarup N, et al. Interactions of Lipid Genetic Risk Scores With Estimates of Metabolic Health in a Danish Population. Circulation Cardiovascular Genetics. 2015; **8**(3): 465-72.
833. Kabayama M, Sakoori K, Yamada K, Ornthanalai VG, Ota M, Morimura N, et al. Rines E3 ubiquitin ligase regulates MAO-A levels and emotional responses. J Neurosci. 2013; **33**(32): 12940-53.
834. Kabayama M, Sakoori K, Yamada K, Ornthanalai VG, Ota M, Morimura N, et al. Rines E3 ubiquitin ligase regulates MAO-A levels and emotional responses. The Journal Of Neuroscience: The Official Journal Of The Society For Neuroscience. 2013; **33**(32): 12940-53.
835. Kalantari N, Doaei S, Keshavarz-Mohammadi N, Gholamalizadeh M, Pazan N. Review of studies on the fat mass and obesity-associated (FTO) gene interactions with environmental factors affecting on obesity and its impact on lifestyle interventions. ARYA Atheroscler. 2016; **12**(6): 281-90.
836. Kalantari N, Doaei S, Keshavarz-Mohammadi N, Gholamalizadeh M, Pazan N. Review of studies on the fat mass and obesity-associated (FTO) gene interactions with environmental factors affecting on obesity and its impact on lifestyle interventions. ARYA Atherosclerosis. 2016; **12**(6): 281-90.
837. Kambouris M, Ntalouka F, Ziogas G, Maffulli N. Predictive genomics DNA profiling for athletic performance. Recent Pat DNA Gene Seq. 2012; **6**(3): 229-39.
838. Kambouris M, Ntalouka F, Ziogas G, Maffulli N. Predictive genomics DNA profiling for athletic performance. Recent Patents On DNA & Gene Sequences. 2012; **6**(3): 229-39.
839. Kang HJ, Kim JM, Lee JY, Kim SY, Bae KY, Kim SW, et al. BDNF promoter methylation and suicidal behavior in depressive patients. J Affect Disord. 2013; **151**(2): 679-85.
840. Kang H-J, Kim J-M, Lee J-Y, Kim S-Y, Bae K-Y, Kim S-W, et al. BDNF promoter methylation and suicidal behavior in depressive patients. Journal Of Affective Disorders. 2013; **151**(2): 679-85.
841. Kang I, Wang Y, Reagan C, Fu Y, Wang MX, Gu L-Q. Designing DNA interstrand lock for locus-specific methylation detection in a nanopore. Scientific Reports. 2013; **3**: 2381-.
842. Kang I, Wang Y, Reagan C, Fu Y, Wang MX, Gu LQ. Designing DNA interstrand lock for locus-specific methylation detection in a nanopore. Sci Rep. 2013; **3**: 2381.
843. Kang RH, Chang HS, Wong ML, Choi MJ, Park JY, Lee HY, et al. Brain-derived neurotrophic factor gene polymorphisms and mirtazapine responses in Koreans with major depression. J Psychopharmacol. 2010; **24**(12): 1755-63.
844. Kang RH, Chang HS, Wong ML, Choi MJ, Park JY, Lee HY, et al. Brain-derived neurotrophic factor gene polymorphisms and mirtazapine responses in Koreans with major depression. Journal Of Psychopharmacology (Oxford, England). 2010; **24**(12): 1755-63.
845. Kaniwa N, Saito Y. The risk of cutaneous adverse reactions among patients with the HLA-A* 31:01 allele who are given carbamazepine, oxcarbazepine or eslicarbazepine: a perspective review. Ther Adv Drug Saf. 2013; **4**(6): 246-53.

846. Kaniwa N, Saito Y. The risk of cutaneous adverse reactions among patients with the HLA-A* 31:01 allele who are given carbamazepine, oxcarbazepine or eslicarbazepine: a perspective review. Therapeutic Advances In Drug Safety. 2013; **4**(6): 246-53.
847. Kao AC, Harty S, Burnet PW. The Influence of Prebiotics on Neurobiology and Behavior. Int Rev Neurobiol. 2016; **131**: 21-48.
848. Kao ACC, Harty S, Burnet PWJ. The Influence of Prebiotics on Neurobiology and Behavior. International Review Of Neurobiology. 2016; **131**: 21-48.
849. Kaplan GB, Moore KA. The use of cognitive enhancers in animal models of fear extinction. Pharmacol Biochem Behav. 2011; **99**(2): 217-28.
850. Kaplan GB, Moore KA. The use of cognitive enhancers in animal models of fear extinction. Pharmacology, Biochemistry, And Behavior. 2011; **99**(2): 217-28.
851. Kaplan GB, Vasterling JJ, Vedak PC. Brain-derived neurotrophic factor in traumatic brain injury, post-traumatic stress disorder, and their comorbid conditions: role in pathogenesis and treatment. Behav Pharmacol. 2010; **21**(5-6): 427-37.
852. Kaplan GB, Vasterling JJ, Vedak PC. Brain-derived neurotrophic factor in traumatic brain injury, post-traumatic stress disorder, and their comorbid conditions: role in pathogenesis and treatment. Behavioural Pharmacology. 2010; **21**(5-6): 427-37.
853. Karasawa S, Daimon M, Sasaki S, Toriyama S, Oizumi T, Susa S, et al. Association of the common fat mass and obesity associated (FTO) gene polymorphism with obesity in a Japanese population. Endocr J. 2010; **57**(4): 293-301.
854. Karasawa S, Daimon M, Sasaki S, Toriyama S, Oizumi T, Susa S, et al. Association of the common fat mass and obesity associated (FTO) gene polymorphism with obesity in a Japanese population. Endocrine Journal. 2010; **57**(4): 293-301.
855. Karasik D, Cupples LA, Hannan MT, Kiel DP. Genome screen for a combined bone phenotype using principal component analysis: the Framingham study. Bone. 2004; **34**(3): 547-56.
856. Karasik D, Cupples LA, Hannan MT, Kiel DP. Genome screen for a combined bone phenotype using principal component analysis: the Framingham study. Bone. 2004; **34**(3): 547-56.
857. Karasik D, Myers RH, Cupples LA, Hannan MT, Gagnon DR, Herbert A, et al. Genome screen for quantitative trait loci contributing to normal variation in bone mineral density: the Framingham Study. J Bone Miner Res. 2002; **17**(9): 1718-27.
858. Karasik D, Myers RH, Cupples LA, Hannan MT, Gagnon DR, Herbert A, et al. Genome screen for quantitative trait loci contributing to normal variation in bone mineral density: the Framingham Study. Journal Of Bone And Mineral Research: The Official Journal Of The American Society For Bone And Mineral Research. 2002; **17**(9): 1718-27.
859. Karasik D, Myers RH, Hannan MT, Gagnon D, McLean RR, Cupples LA, et al. Mapping of quantitative ultrasound of the calcaneus bone to chromosome 1 by genome-wide linkage analysis. Osteoporos Int. 2002; **13**(10): 796-802.
860. Karasik D, Myers RH, Hannan MT, Gagnon D, McLean RR, Cupples LA, et al. Mapping of quantitative ultrasound of the calcaneus bone to chromosome 1 by genome-wide linkage analysis. Osteoporosis International: A Journal Established As Result Of Cooperation Between The European Foundation For Osteoporosis And The National

Osteoporosis Foundation Of The USA. 2002; **13**(10): 796-802.
861. Karlsson L, Nordenstrom A, Hirvikoski T, Lajic S. Prenatal dexamethasone treatment in the context of at risk CAH pregnancies: Long-term behavioral and cognitive outcome. Psychoneuroendocrinology. 2018; **91**: 68-74.
862. Karlsson L, Nordenström A, Hirvikoski T, Lajic S. Prenatal dexamethasone treatment in the context of at risk CAH pregnancies: Long-term behavioral and cognitive outcome. Psychoneuroendocrinology. 2018; **91**: 68-74.
863. Karpova NN, Lindholm JS, Kulesskaya N, Onishchenko N, Vahter M, Popova D, et al. TrkB overexpression in mice buffers against memory deficits and depression-like behavior but not all anxiety- and stress-related symptoms induced by developmental exposure to methylmercury. Front Behav Neurosci. 2014; **8**: 315.
864. Karpova NN, Lindholm JSO, Kulesskaya N, Onishchenko N, Vahter M, Popova D, et al. TrkB overexpression in mice buffers against memory deficits and depression-like behavior but not all anxiety- and stress-related symptoms induced by developmental exposure to methylmercury. Frontiers In Behavioral Neuroscience. 2014; **8**: 315-.
865. Karunasinghe N, Zhu Y, Han DY, Lange K, Zhu S, Wang A, et al. Quality of life effects of androgen deprivation therapy in a prostate cancer cohort in New Zealand: can we minimize effects using a stratification based on the aldo-keto reductase family 1, member C3 rs12529 gene polymorphism? BMC Urol. 2016; **16**(1): 48.
866. Karunasinghe N, Zhu Y, Han DY, Lange K, Zhu S, Wang A, et al. Quality of life effects of androgen deprivation therapy in a prostate cancer cohort in New Zealand: can we minimize effects using a stratification based on the aldo-keto reductase family 1, member C3 rs12529 gene polymorphism? BMC Urology. 2016; **16**(1): 48-.
867. Kasikcioglu E, Kayserilioglu A, Ciloglu F, Akhan H, Oflaz H, Yildiz S, et al. Angiotensin-converting enzyme gene polymorphism, left ventricular remodeling, and exercise capacity in strength-trained athletes. Heart Vessels. 2004; **19**(6): 287-93.
868. Kasikcioglu E, Kayserilioglu A, Ciloglu F, Akhan H, Oflaz H, Yildiz S, et al. Angiotensin-converting enzyme gene polymorphism, left ventricular remodeling, and exercise capacity in strength-trained athletes. Heart And Vessels. 2004; **19**(6): 287-93.
869. Kasper S, Praschak-Rieder N, Tauscher J, Wolf R. A risk-benefit assessment of mirtazapine in the treatment of depression. Drug Saf. 1997; **17**(4): 251-64.
870. Kasper S, Praschak-Rieder N, Tauscher J, Wolf R. A risk-benefit assessment of mirtazapine in the treatment of depression. Drug Safety. 1997; **17**(4): 251-64.
871. Kasurinen A, Biasi C, Holopainen T, Rousi M, Maenpaa M, Oksanen E. Interactive effects of elevated ozone and temperature on carbon allocation of silver birch (Betula pendula) genotypes in an open-air field exposure. Tree Physiol. 2012; **32**(6): 737-51.
872. Kasurinen A, Biasi C, Holopainen T, Rousi M, Mäenpää M, Oksanen E. Interactive effects of elevated ozone and temperature on carbon allocation of silver birch (Betula pendula) genotypes in an open-air field exposure. Tree Physiology. 2012; **32**(6): 737-51.
873. Kautzky A, Baldinger P, Souery D, Montgomery S, Mendlewicz J, Zohar J, et al. The combined effect of genetic polymorphisms and clinical parameters on treatment outcome in treatment-resistant depression. Eur Neuropsychopharmacol. 2015; **25**(4):

441-53.
874. Kautzky A, Baldinger P, Souery D, Montgomery S, Mendlewicz J, Zohar J, et al. The combined effect of genetic polymorphisms and clinical parameters on treatment outcome in treatment-resistant depression. European Neuropsychopharmacology: The Journal Of The European College Of Neuropsychopharmacology. 2015; **25**(4): 441-53.
875. Keers R, Coleman JR, Lester KJ, Roberts S, Breen G, Thastum M, et al. A Genome-Wide Test of the Differential Susceptibility Hypothesis Reveals a Genetic Predictor of Differential Response to Psychological Treatments for Child Anxiety Disorders. Psychother Psychosom. 2016; **85**(3): 146-58.
876. Keers R, Coleman JRI, Lester KJ, Roberts S, Breen G, Thastum M, et al. A Genome-Wide Test of the Differential Susceptibility Hypothesis Reveals a Genetic Predictor of Differential Response to Psychological Treatments for Child Anxiety Disorders. Psychotherapy And Psychosomatics. 2016; **85**(3): 146-58.
877. Kelishadi R, Haghjooy Javanmard S, Tajadini MH, Mansourian M, Motlagh ME, Ardalan G, et al. Genetic association with low concentrations of high density lipoprotein-cholesterol in a pediatric population of the Middle East and North Africa: the CASPIAN-III study. Atherosclerosis. 2014; **237**(1): 273-8.
878. Kelishadi R, Haghjooy Javanmard S, Tajadini MH, Mansourian M, Motlagh ME, Ardalan G, et al. Genetic association with low concentrations of high density lipoprotein-cholesterol in a pediatric population of the Middle East and North Africa: the CASPIAN-III study. Atherosclerosis. 2014; **237**(1): 273-8.
879. Kellner M, Demiralay C, Muhtz C, Husemann J, Kolsch W, Hiemke C, et al. No effect of six weeks of treatment with escitalopram on mood in healthy volunteers--irrespective of genotype for the promoter of the serotonin transporter. Psychiatry Res. 2008; **161**(3): 339-43.
880. Kellner M, Demiralay C, Muhtz C, Husemann J, Kölsch W, Hiemke C, et al. No effect of six weeks of treatment with escitalopram on mood in healthy volunteers--irrespective of genotype for the promoter of the serotonin transporter. Psychiatry Research. 2008; **161**(3): 339-43.
881. Kellner M, Muhtz C, Demiralay C, Husemann J, Koelsch W, Yassouridis A, et al. The selective serotonin re-uptake inhibitor escitalopram modulates the panic response to cholecystokinin tetrapeptide in healthy men depending on 5-HTTLPR genotype. J Psychiatr Res. 2009; **43**(6): 642-8.
882. Kellner M, Muhtz C, Demiralay C, Husemann J, Koelsch W, Yassouridis A, et al. The selective serotonin re-uptake inhibitor escitalopram modulates the panic response to cholecystokinin tetrapeptide in healthy men depending on 5-HTTLPR genotype. Journal Of Psychiatric Research. 2009; **43**(6): 642-8.
883. Kempton CL, Soucie JM, Miller CH, Hooper C, Escobar MA, Cohen AJ, et al. In non-severe hemophilia A the risk of inhibitor after intensive factor treatment is greater in older patients: a case-control study. J Thromb Haemost. 2010; **8**(10): 2224-31.
884. Kempton CL, Soucie JM, Miller CH, Hooper C, Escobar MA, Cohen AJ, et al. In non-severe hemophilia A the risk of inhibitor after intensive factor treatment is greater in older patients: a case-control study. Journal Of Thrombosis And Haemostasis: JTH. 2010; **8**(10): 2224-31.
885. Kerb R, Brinkmann U. Cambridge Healthtech Institute's 2nd Annual Conference

on Pharmacogenomics Europe: presaging profits. Pharmacogenomics. 2001; **2**(3): 303-5.
886. Kerb R, Brinkmann U. Cambridge Healthtech Institute's 2nd Annual Conference on Pharmacogenomics Europe: presaging profits. Pharmacogenomics. 2001; **2**(3): 303-5.
887. Kettner NM, Katchy CA, Fu L. Circadian gene variants in cancer. Ann Med. 2014; **46**(4): 208-20.
888. Kettner NM, Katchy CA, Fu L. Circadian gene variants in cancer. Annals Of Medicine. 2014; **46**(4): 208-20.
889. Khalil I, Deschamps I, Lepage V, al-Daccak R, Degos L, Hors J. Dose effect of cis- and trans-encoded HLA-DQ alpha beta heterodimers in IDDM susceptibility. Diabetes. 1992; **41**(3): 378-84.
890. Khalil I, Deschamps I, Lepage V, al-Daccak R, Degos L, Hors J. Dose effect of cis- and trans-encoded HLA-DQ alpha beta heterodimers in IDDM susceptibility. Diabetes. 1992; **41**(3): 378-84.
891. Khan RJ, Riestra P, Gebreab SY, Wilson JG, Gaye A, Xu R, et al. Vitamin D Receptor Gene Polymorphisms Are Associated with Abdominal Visceral Adipose Tissue Volume and Serum Adipokine Concentrations but Not with Body Mass Index or Waist Circumference in African Americans: The Jackson Heart Study. J Nutr. 2016; **146**(8): 1476-82.
892. Khan RJ, Riestra P, Gebreab SY, Wilson JG, Gaye A, Xu R, et al. Vitamin D Receptor Gene Polymorphisms Are Associated with Abdominal Visceral Adipose Tissue Volume and Serum Adipokine Concentrations but Not with Body Mass Index or Waist Circumference in African Americans: The Jackson Heart Study. The Journal Of Nutrition. 2016; **146**(8): 1476-82.
893. Kim DH, Ahn YO. Molecular epidemiology of colon cancer. Cancer Res Treat. 2004; **36**(2): 93-9.
894. Kim D-H, Ahn Y-O. Molecular epidemiology of colon cancer. Cancer Research And Treatment: Official Journal Of Korean Cancer Association. 2004; **36**(2): 93-9.
895. Kim HJ, Song BK, So B, Lee O, Song W, Kim Y. Increase of circulating BDNF levels and its relation to improvement of physical fitness following 12 weeks of combined exercise in chronic patients with schizophrenia: a pilot study. Psychiatry Res. 2014; **220**(3): 792-6.
896. Kim H-j, Song B-k, So B, Lee O, Song W, Kim Y. Increase of circulating BDNF levels and its relation to improvement of physical fitness following 12 weeks of combined exercise in chronic patients with schizophrenia: a pilot study. Psychiatry Research. 2014; **220**(3): 792-6.
897. Kim HS, Kim JS, Kim NS, Kim JH, Lee BK. Association of vitamin D receptor polymorphism with calcaneal broadband ultrasound attenuation in Korean postmenopausal women with low calcium intake. Br J Nutr. 2007; **98**(5): 878-81.
898. Kim HS, Newcomb PA, Ulrich CM, Keener CL, Bigler J, Farin FM, et al. Vitamin D receptor polymorphism and the risk of colorectal adenomas: evidence of interaction with dietary vitamin D and calcium. Cancer Epidemiol Biomarkers Prev. 2001; **10**(8): 869-74.
899. Kim HS, Newcomb PA, Ulrich CM, Keener CL, Bigler J, Farin FM, et al. Vitamin D receptor polymorphism and the risk of colorectal adenomas: evidence of interaction with

dietary vitamin D and calcium. Cancer Epidemiology, Biomarkers & Prevention: A Publication Of The American Association For Cancer Research, Cosponsored By The American Society Of Preventive Oncology. 2001; **10**(8): 869-74.
900. Kim H-S, Kim J-S, Kim NS, Kim JH, Lee B-K. Association of vitamin D receptor polymorphism with calcaneal broadband ultrasound attenuation in Korean postmenopausal women with low calcium intake. The British Journal Of Nutrition. 2007; **98**(5): 878-81.
901. Kim JM, Kim SW, Shin IS, Yang SJ, Park WY, Kim SJ, et al. Folate, vitamin b(12), and homocysteine as risk factors for cognitive decline in the elderly. Psychiatry Investig. 2008; **5**(1): 36-40.
902. Kim J-M, Kim S-W, Shin I-S, Yang S-J, Park W-Y, Kim S-J, et al. Folate, vitamin b(12), and homocysteine as risk factors for cognitive decline in the elderly. Psychiatry Investigation. 2008; **5**(1): 36-40.
903. Kim JY, Kim D, Park K, Lee JH, Jahng JW. Highly palatable food access during adolescence increased anxiety-/depression-like behaviors in male, but not in female, rats. Nutr Neurosci. 2017: 1-9.
904. Kim JY, Kim D, Park K, Lee J-H, Jahng JW. Highly palatable food access during adolescence increased anxiety-/depression-like behaviors in male, but not in female, rats. Nutritional Neuroscience. 2017: 1-9.
905. Kim MS, Park JS, Kim KH. Optimal place of a foreign gene in the genome of viral haemorrhagic septicaemia virus (VHSV) for development of VHSV-based viral-vectored vaccines. J Appl Microbiol. 2013; **114**(6): 1866-73.
906. Kim MS, Park JS, Kim KH. Optimal place of a foreign gene in the genome of viral haemorrhagic septicaemia virus (VHSV) for development of VHSV-based viral-vectored vaccines. Journal Of Applied Microbiology. 2013; **114**(6): 1866-73.
907. Kim S, Myers L, Ravussin E, Cherry KE, Jazwinski SM. Single nucleotide polymorphisms linked to mitochondrial uncoupling protein genes UCP2 and UCP3 affect mitochondrial metabolism and healthy aging in female nonagenarians. Biogerontology. 2016; **17**(4): 725-36.
908. Kim S, Myers L, Ravussin E, Cherry KE, Jazwinski SM. Single nucleotide polymorphisms linked to mitochondrial uncoupling protein genes UCP2 and UCP3 affect mitochondrial metabolism and healthy aging in female nonagenarians. Biogerontology. 2016; **17**(4): 725-36.
909. Kim W, Choi YH, Yoon KS, Cho DY, Pae CU, Woo JM. Tryptophan hydroxylase and serotonin transporter gene polymorphism does not affect the diagnosis, clinical features and treatment outcome of panic disorder in the Korean population. Prog Neuropsychopharmacol Biol Psychiatry. 2006; **30**(8): 1413-8.
910. Kim W, Choi YH, Yoon K-S, Cho D-Y, Pae C-U, Woo J-M. Tryptophan hydroxylase and serotonin transporter gene polymorphism does not affect the diagnosis, clinical features and treatment outcome of panic disorder in the Korean population. Progress In Neuro-Psychopharmacology & Biological Psychiatry. 2006; **30**(8): 1413-8.
911. Kindie E, Alamrew Anteneh Z, Worku E. Time to development of adverse drug reactions and associated factors among adult HIV positive patients on antiretroviral treatment in Bahir Dar City, Northwest Ethiopia. PLoS One. 2017; **12**(12): e0189322.

912. Kindie E, Alamrew Anteneh Z, Worku E. Time to development of adverse drug reactions and associated factors among adult HIV positive patients on antiretroviral treatment in Bahir Dar City, Northwest Ethiopia. PLoS One. 2017; **12**(12): e0189322-e.
913. Kirac D, Kasimay Cakir O, Avcilar T, Deyneli O, Kurtel H, Yazici D, et al. Effects of MC4R, FTO, and NMB gene variants to obesity, physical activity, and eating behavior phenotypes. IUBMB Life. 2016; **68**(10): 806-16.
914. Kirac D, Kasimay Cakir O, Avcilar T, Deyneli O, Kurtel H, Yazici D, et al. Effects of MC4R, FTO, and NMB gene variants to obesity, physical activity, and eating behavior phenotypes. IUBMB Life. 2016; **68**(10): 806-16.
915. Kitagawa I, Kitagawa Y, Nagaya T, Tokudome S. Interplay of physical activity and vitamin D receptor gene polymorphism on bone mineral density. J Epidemiol. 2001; **11**(5): 229-32.
916. Kitagawa I, Kitagawa Y, Nagaya T, Tokudome S. Interplay of physical activity and vitamin D receptor gene polymorphism on bone mineral density. Journal Of Epidemiology. 2001; **11**(5): 229-32.
917. Klauke B, Winter B, Gajewska A, Zwanzger P, Reif A, Herrmann MJ, et al. Affect-modulated startle: interactive influence of catechol-O-methyltransferase Val158Met genotype and childhood trauma. PLoS One. 2012; **7**(6): e39709.
918. Klauke B, Winter B, Gajewska A, Zwanzger P, Reif A, Herrmann MJ, et al. Affect-modulated startle: interactive influence of catechol-O-methyltransferase Val158Met genotype and childhood trauma. PLoS One. 2012; **7**(6): e39709-e.
919. Klok MD, Vreeburg SA, Penninx BW, Zitman FG, de Kloet ER, DeRijk RH. Common functional mineralocorticoid receptor polymorphisms modulate the cortisol awakening response: Interaction with SSRIs. Psychoneuroendocrinology. 2011; **36**(4): 484-94.
920. Klok MD, Vreeburg SA, Penninx BWJH, Zitman FG, de Kloet ER, DeRijk RH. Common functional mineralocorticoid receptor polymorphisms modulate the cortisol awakening response: Interaction with SSRIs. Psychoneuroendocrinology. 2011; **36**(4): 484-94.
921. Klump KL, Culbert KM. Molecular Genetic Studies of Eating Disorders: Current Status and Future Directions. Curr Dir Psychol Sci. 2007; **16**(1): 37-41.
922. Klump KL, Culbert KM. Molecular Genetic Studies of Eating Disorders: Current Status and Future Directions. Current Directions In Psychological Science. 2007; **16**(1): 37-41.
923. Knadler MP, Lobo E, Chappell J, Bergstrom R. Duloxetine: clinical pharmacokinetics and drug interactions. Clin Pharmacokinet. 2011; **50**(5): 281-94.
924. Knadler MP, Lobo E, Chappell J, Bergstrom R. Duloxetine: clinical pharmacokinetics and drug interactions. Clinical Pharmacokinetics. 2011; **50**(5): 281-94.
925. Knight JM, Kim E, Ivanov I, Davidson LA, Goldsby JS, Hullar MA, et al. Comprehensive site-specific whole genome profiling of stromal and epithelial colonic gene signatures in human sigmoid colon and rectal tissue. Physiol Genomics. 2016; **48**(9): 651-9.
926. Knight JM, Kim E, Ivanov I, Davidson LA, Goldsby JS, Hullar MAJ, et al. Comprehensive site-specific whole genome profiling of stromal and epithelial colonic gene signatures in human sigmoid colon and rectal tissue. Physiological Genomics.

2016; **48**(9): 651-9.
927. Knoll AT, Halladay LR, Holmes AJ, Levitt P. Quantitative Trait Loci and a Novel Genetic Candidate for Fear Learning. J Neurosci. 2016; **36**(23): 6258-68.
928. Knoll AT, Halladay LR, Holmes AJ, Levitt P. Quantitative Trait Loci and a Novel Genetic Candidate for Fear Learning. The Journal Of Neuroscience: The Official Journal Of The Society For Neuroscience. 2016; **36**(23): 6258-68.
929. Knoll N, Volckmar AL, Putter C, Scherag A, Kleber M, Hebebrand J, et al. The fatty acid amide hydrolase (FAAH) gene variant rs324420 AA/AC is not associated with weight loss in a 1-year lifestyle intervention for obese children and adolescents. Horm Metab Res. 2012; **44**(1): 75-7.
930. Knoll N, Volckmar AL, Pütter C, Scherag A, Kleber M, Hebebrand J, et al. The fatty acid amide hydrolase (FAAH) gene variant rs324420 AA/AC is not associated with weight loss in a 1-year lifestyle intervention for obese children and adolescents. Hormone And Metabolic Research = Hormon- Und Stoffwechselforschung = Hormones Et Metabolisme. 2012; **44**(1): 75-7.
931. Knox SS, Guo X, Zhang Y, Weidner G, Williams S, Ellison RC. AGT M235T genotype/anxiety interaction and gender in the HyperGEN study. PLoS One. 2010; **5**(10): e13353.
932. Knox SS, Guo X, Zhang Y, Weidner G, Williams S, Ellison RC. AGT M235T genotype/anxiety interaction and gender in the HyperGEN study. PLoS One. 2010; **5**(10): e13353-e.
933. Kobayashi K, Shimizu E, Hashimoto K, Mitsumori M, Koike K, Okamura N, et al. Serum brain-derived neurotrophic factor (BDNF) levels in patients with panic disorder: as a biological predictor of response to group cognitive behavioral therapy. Prog Neuropsychopharmacol Biol Psychiatry. 2005; **29**(5): 658-63.
934. Kobayashi K, Shimizu E, Hashimoto K, Mitsumori M, Koike K, Okamura N, et al. Serum brain-derived neurotrophic factor (BDNF) levels in patients with panic disorder: as a biological predictor of response to group cognitive behavioral therapy. Progress In Neuro-Psychopharmacology & Biological Psychiatry. 2005; **29**(5): 658-63.
935. Koch S, MacInnis MJ, Rupert JL, Sporer BC, Koehle MS. Pharmacogenetic Effects of Inhaled Salbutamol on 10-km Time Trial Performance in Competitive Male and Female Cyclists. Clin J Sport Med. 2016; **26**(2): 145-51.
936. Koch S, MacInnis MJ, Rupert JL, Sporer BC, Koehle MS. Pharmacogenetic Effects of Inhaled Salbutamol on 10-km Time Trial Performance in Competitive Male and Female Cyclists. Clinical Journal Of Sport Medicine: Official Journal Of The Canadian Academy Of Sport Medicine. 2016; **26**(2): 145-51.
937. Koh MJ, Jeung HC, Namkoong K, Chung HC, Kang JI. Influence of the BDNF Val66Met polymorphism on coping response to stress in patients with advanced gastric cancer. J Psychosom Res. 2014; **77**(1): 76-80.
938. Koh MJ, Jeung H-C, Namkoong K, Chung HC, Kang JI. Influence of the BDNF Val66Met polymorphism on coping response to stress in patients with advanced gastric cancer. Journal Of Psychosomatic Research. 2014; **77**(1): 76-80.
939. Kohli A, Shaffer A, Sherman A, Kottilil S. Treatment of hepatitis C: a systematic review. JAMA. 2014; **312**(6): 631-40.
940. Kohli A, Shaffer A, Sherman A, Kottilil S. Treatment of hepatitis C: a systematic

review. JAMA. 2014; **312**(6): 631-40.
941. Kohlmeier M, De Caterina R, Ferguson LR, Gorman U, Allayee H, Prasad C, et al. Guide and Position of the International Society of Nutrigenetics/Nutrigenomics on Personalized Nutrition: Part 2 - Ethics, Challenges and Endeavors of Precision Nutrition. J Nutrigenet Nutrigenomics. 2016; **9**(1): 28-46.
942. Kohlmeier M, De Caterina R, Ferguson LR, Görman U, Allayee H, Prasad C, et al. Guide and Position of the International Society of Nutrigenetics/Nutrigenomics on Personalized Nutrition: Part 2 - Ethics, Challenges and Endeavors of Precision Nutrition. Journal Of Nutrigenetics And Nutrigenomics. 2016; **9**(1): 28-46.
943. Kolassa IT, Kolassa S, Ertl V, Papassotiropoulos A, De Quervain DJ. The risk of posttraumatic stress disorder after trauma depends on traumatic load and the catechol-o-methyltransferase Val(158)Met polymorphism. Biol Psychiatry. 2010; **67**(4): 304-8.
944. Kolassa I-T, Kolassa S, Ertl V, Papassotiropoulos A, De Quervain DJF. The risk of posttraumatic stress disorder after trauma depends on traumatic load and the catechol-o-methyltransferase Val(158)Met polymorphism. Biological Psychiatry. 2010; **67**(4): 304-8.
945. Kostek MC, Delmonico MJ, Reichel JB, Roth SM, Douglass L, Ferrell RE, et al. Muscle strength response to strength training is influenced by insulin-like growth factor 1 genotype in older adults. J Appl Physiol (1985). 2005; **98**(6): 2147-54.
946. Kostek MC, Delmonico MJ, Reichel JB, Roth SM, Douglass L, Ferrell RE, et al. Muscle strength response to strength training is influenced by insulin-like growth factor 1 genotype in older adults. Journal Of Applied Physiology (Bethesda, Md: 1985). 2005; **98**(6): 2147-54.
947. Kovacs Z, D'Agostino DP, Ari C. Anxiolytic Effect of Exogenous Ketone Supplementation Is Abolished by Adenosine A1 Receptor Inhibition in Wistar Albino Glaxo/Rijswijk Rats. Front Behav Neurosci. 2018; **12**: 29.
948. Kovács Z, D'Agostino DP, Ari C. Anxiolytic Effect of Exogenous Ketone Supplementation Is Abolished by Adenosine A1 Receptor Inhibition in Wistar Albino Glaxo/Rijswijk Rats. Frontiers In Behavioral Neuroscience. 2018; **12**: 29-.
949. Kozisek ME, Middlemas D, Bylund DB. Brain-derived neurotrophic factor and its receptor tropomyosin-related kinase B in the mechanism of action of antidepressant therapies. Pharmacol Ther. 2008; **117**(1): 30-51.
950. Kozisek ME, Middlemas D, Bylund DB. Brain-derived neurotrophic factor and its receptor tropomyosin-related kinase B in the mechanism of action of antidepressant therapies. Pharmacology & Therapeutics. 2008; **117**(1): 30-51.
951. Kramer JR, Kanwal F, Richardson P, Giordano TP, Petersen LA, El-Serag HB. Importance of patient, provider, and facility predictors of hepatitis C virus treatment in veterans: a national study. Am J Gastroenterol. 2011; **106**(3): 483-91.
952. Kramer JR, Kanwal F, Richardson P, Giordano TP, Petersen LA, El-Serag HB. Importance of patient, provider, and facility predictors of hepatitis C virus treatment in veterans: a national study. The American Journal Of Gastroenterology. 2011; **106**(3): 483-91.
953. Kraus MR, Al-Taie O, Schafer A, Pfersdorff M, Lesch KP, Scheurlen M. Serotonin-1A receptor gene HTR1A variation predicts interferon-induced depression in chronic hepatitis C. Gastroenterology. 2007; **132**(4): 1279-86.

954. Kraus MR, Al-Taie O, Schäfer A, Pfersdorff M, Lesch K-P, Scheurlen M. Serotonin-1A receptor gene HTR1A variation predicts interferon-induced depression in chronic hepatitis C. Gastroenterology. 2007; **132**(4): 1279-86.
955. Kravatsky YV, Chechetkin VR, Fedoseeva DM, Gorbacheva MA, Kretova OV, Tchurikov NA. [Mutation frequencies in HIV-1 subtype-A genome in regions containing efficient RNAi targets]. Mol Biol (Mosk). 2016; **50**(3): 480-5.
956. Kravatsky YV, Chechetkin VR, Fedoseeva DM, Gorbacheva MA, Kretova OV, Tchurikov NA. [Mutation frequencies in HIV-1 subtype-A genome in regions containing efficient RNAi targets]. Molekuliarnaia Biologiia. 2016; **50**(3): 480-5.
957. Krawczyk M, Stachowska E, Milkiewicz P, Lammert F, Milkiewicz M. Reduction of Caloric Intake Might Override the Prosteatotic Effects of the PNPLA3 p.I148M and TM6SF2 p.E167K Variants in Patients with Fatty Liver: Ultrasound-Based Prospective Study. Digestion. 2016; **93**(2): 139-48.
958. Krawczyk M, Stachowska E, Milkiewicz P, Lammert F, Milkiewicz M. Reduction of Caloric Intake Might Override the Prosteatotic Effects of the PNPLA3 p.I148M and TM6SF2 p.E167K Variants in Patients with Fatty Liver: Ultrasound-Based Prospective Study. Digestion. 2016; **93**(2): 139-48.
959. Kretowski A, Adamska E, Maliszewska K, Wawrusiewicz-Kurylonek N, Citko A, Goscik J, et al. The rs340874 PROX1 type 2 diabetes mellitus risk variant is associated with visceral fat accumulation and alterations in postprandial glucose and lipid metabolism. Genes Nutr. 2015; **10**(2): 4.
960. Kretowski A, Adamska E, Maliszewska K, Wawrusiewicz-Kurylonek N, Citko A, Goscik J, et al. The rs340874 PROX1 type 2 diabetes mellitus risk variant is associated with visceral fat accumulation and alterations in postprandial glucose and lipid metabolism. Genes & Nutrition. 2015; **10**(2): 4-.
961. Kumar A, Yadav AK, Sreekrishnan TR, Satya S, Kaushik CP. Treatment of low strength industrial cluster wastewater by anaerobic hybrid reactor. Bioresour Technol. 2008; **99**(8): 3123-9.
962. Kumar A, Yadav AK, Sreekrishnan TR, Satya S, Kaushik CP. Treatment of low strength industrial cluster wastewater by anaerobic hybrid reactor. Bioresource Technology. 2008; **99**(8): 3123-9.
963. Kumar P, Kumar A, Misra S, Faruq M, Vivekanandhan S, Srivastava AK, et al. Association between lymphotoxin alpha (-252 A/G and -804 C/A) gene polymorphisms and risk of stroke in North Indian population: a hospital-based case-control study. Int J Neurosci. 2016; **126**(12): 1127-35.
964. Kumar P, Kumar A, Misra S, Faruq M, Vivekanandhan S, Srivastava AK, et al. Association between lymphotoxin alpha (-252 A/G and -804 C/A) gene polymorphisms and risk of stroke in North Indian population: a hospital-based case-control study. The International Journal Of Neuroscience. 2016; **126**(12): 1127-35.
965. Kumar P, Kumar A, Sagar R, Misra S, Faruq M, Suroliya V, et al. Association between Interleukin-10 -1082G/A Gene Polymorphism and Risk of Stroke in the North Indian Population: A Case-Control Study. J Stroke Cerebrovasc Dis. 2016; **25**(2): 461-8.
966. Kumar P, Kumar A, Sagar R, Misra S, Faruq M, Suroliya V, et al. Association between Interleukin-10 -1082G/A Gene Polymorphism and Risk of Stroke in the North Indian Population: A Case-Control Study. Journal Of Stroke And Cerebrovascular

Diseases: The Official Journal Of National Stroke Association. 2016; **25**(2): 461-8.
967. Kumar R, Mamrutha HM, Kaur A, Venkatesh K, Grewal A, Kumar R, et al. Development of an efficient and reproducible regeneration system in wheat (Triticum aestivum L.). Physiology And Molecular Biology Of Plants: An International Journal Of Functional Plant Biology. 2017; **23**(4): 945-54.
968. Kumar R, Mamrutha HM, Kaur A, Venkatesh K, Grewal A, Tiwari V. Development of an efficient and reproducible regeneration system in wheat (Triticum aestivum L.). Physiol Mol Biol Plants. 2017; **23**(4): 945-54.
969. Kuncl RW. Agents and mechanisms of toxic myopathy. Curr Opin Neurol. 2009; **22**(5): 506-15.
970. Kuncl RW. Agents and mechanisms of toxic myopathy. Current Opinion In Neurology. 2009; **22**(5): 506-15.
971. Kunin-Batson AS, Shapiro EG, Rudser KD, Lavery CA, Bjoraker KJ, Jones SA, et al. Long-Term Cognitive and Functional Outcomes in Children with Mucopolysaccharidosis (MPS)-IH (Hurler Syndrome) Treated with Hematopoietic Cell Transplantation. JIMD Rep. 2016; **29**: 95-102.
972. Kunin-Batson AS, Shapiro EG, Rudser KD, Lavery CA, Bjoraker KJ, Jones SA, et al. Long-Term Cognitive and Functional Outcomes in Children with Mucopolysaccharidosis (MPS)-IH (Hurler Syndrome) Treated with Hematopoietic Cell Transplantation. JIMD Reports. 2016; **29**: 95-102.
973. Kuo YT, Chang TT, Muo CH, Wu MY, Sun MF, Yeh CC, et al. Use of Complementary Traditional Chinese Medicines by Adult Cancer Patients in Taiwan: A Nationwide Population-Based Study. Integr Cancer Ther. 2017: 1534735417716302.
974. Kuo Y-T, Chang T-T, Muo C-H, Wu M-Y, Sun M-F, Yeh C-C, et al. Use of Complementary Traditional Chinese Medicines by Adult Cancer Patients in Taiwan: A Nationwide Population-Based Study. Integrative Cancer Therapies. 2018; **17**(2): 531-41.
975. Kupari M, Hautanen A, Lankinen L, Koskinen P, Virolainen J, Nikkila H, et al. Associations between human aldosterone synthase (CYP11B2) gene polymorphisms and left ventricular size, mass, and function. Circulation. 1998; **97**(6): 569-75.
976. Kupari M, Hautanen A, Lankinen L, Koskinen P, Virolainen J, Nikkila H, et al. Associations between human aldosterone synthase (CYP11B2) gene polymorphisms and left ventricular size, mass, and function. Circulation. 1998; **97**(6): 569-75.
977. Kuypers KPC, de la Torre R, Farre M, Xicota L, de Sousa Fernandes Perna EB, Theunissen EL, et al. Depressive mood ratings are reduced by MDMA in female polydrug ecstasy users homozygous for the l-allele of the serotonin transporter. Sci Rep. 2018; **8**(1): 1061.
978. Kuypers KPC, de la Torre R, Farre M, Xicota L, de Sousa Fernandes Perna EB, Theunissen EL, et al. Depressive mood ratings are reduced by MDMA in female polydrug ecstasy users homozygous for the l-allele of the serotonin transporter. Scientific Reports. 2018; **8**(1): 1061-.
979. Laaksonen MM, Impivaara O, Sievanen H, Viikari JS, Lehtimaki TJ, Lamberg-Allardt CJ, et al. Associations of genetic lactase non-persistence and sex with bone loss in young adulthood. Bone. 2009; **44**(5): 1003-9.
980. Laaksonen MM, Karkkainen MU, Outila TA, Rita HJ, Lamberg-Allardt CJ. Vitamin D receptor gene start codon polymorphism (FokI) is associated with forearm bone

mineral density and calcaneal ultrasound in Finnish adolescent boys but not in girls. J Bone Miner Metab. 2004; **22**(5): 479-85.
981. Laaksonen MM, Mikkila V, Rasanen L, Rontu R, Lehtimaki TJ, Viikari JS, et al. Genetic lactase non-persistence, consumption of milk products and intakes of milk nutrients in Finns from childhood to young adulthood. Br J Nutr. 2009; **102**(1): 8-17.
982. Laaksonen MML, Impivaara O, Sievänen H, Viikari JSA, Lehtimäki TJ, Lamberg-Allardt CJE, et al. Associations of genetic lactase non-persistence and sex with bone loss in young adulthood. Bone. 2009; **44**(5): 1003-9.
983. Laaksonen MML, Kärkkäinen MUM, Outila TA, Rita HJ, Lamberg-Allardt CJE. Vitamin D receptor gene start codon polymorphism (FokI) is associated with forearm bone mineral density and calcaneal ultrasound in Finnish adolescent boys but not in girls. Journal Of Bone And Mineral Metabolism. 2004; **22**(5): 479-85.
984. Laaksonen MML, Mikkilä V, Räsänen L, Rontu R, Lehtimäki TJ, Viikari JSA, et al. Genetic lactase non-persistence, consumption of milk products and intakes of milk nutrients in Finns from childhood to young adulthood. The British Journal Of Nutrition. 2009; **102**(1): 8-17.
985. Lam P, Cheng CY, Hong CJ, Tsai SJ. Association study of a brain-derived neurotrophic factor (Val66Met) genetic polymorphism and panic disorder. Neuropsychobiology. 2004; **49**(4): 178-81.
986. Lam P, Cheng C-Y, Hong C-J, Tsai S-J. Association study of a brain-derived neurotrophic factor (Val66Met) genetic polymorphism and panic disorder. Neuropsychobiology. 2004; **49**(4): 178-81.
987. Lam P, Hong CJ, Tsai SJ. Association study of A2a adenosine receptor genetic polymorphism in panic disorder. Neurosci Lett. 2005; **378**(2): 98-101.
988. Lam P, Hong C-J, Tsai S-J. Association study of A2a adenosine receptor genetic polymorphism in panic disorder. Neuroscience Letters. 2005; **378**(2): 98-101.
989. Lammert F, Matern S. [Evidence-based prevention of cholecystolithiasis]. Dtsch Med Wochenschr. 2004; **129**(28-29): 1548-50.
990. Lammert F, Matern S. [Evidence-based prevention of cholecystolithiasis]. Deutsche Medizinische Wochenschrift (1946). 2004; **129**(28-29): 1548-50.
991. Lane HY, Jann MW, Chang YC, Chiu CC, Huang MC, Lee SH, et al. Repeated ingestion of grapefruit juice does not alter clozapine's steady-state plasma levels, effectiveness, and tolerability. J Clin Psychiatry. 2001; **62**(10): 812-7.
992. Lane HY, Jann MW, Chang YC, Chiu CC, Huang MC, Lee SH, et al. Repeated ingestion of grapefruit juice does not alter clozapine's steady-state plasma levels, effectiveness, and tolerability. The Journal Of Clinical Psychiatry. 2001; **62**(10): 812-7.
993. Lane HY, Lin CC, Huang CH, Chang YC, Hsu SK, Chang WH. Risperidone response and 5-HT6 receptor gene variance: genetic association analysis with adjustment for nongenetic confounders. Schizophr Res. 2004; **67**(1): 63-70.
994. Lane H-Y, Lin C-C, Huang C-H, Chang Y-C, Hsu S-K, Chang W-H. Risperidone response and 5-HT6 receptor gene variance: genetic association analysis with adjustment for nongenetic confounders. Schizophrenia Research. 2004; **67**(1): 63-70.
995. Lapice E, Monticelli A, Cocozza S, Pinelli M, Giacco A, Rivellese AA, et al. The energy intake modulates the association of the -55CT polymorphism of UCP3 with body weight in type 2 diabetic patients. Int J Obes (Lond). 2014; **38**(6): 873-7.

996. Lapice E, Monticelli A, Cocozza S, Pinelli M, Giacco A, Rivellese AA, et al. The energy intake modulates the association of the -55CT polymorphism of UCP3 with body weight in type 2 diabetic patients. International Journal Of Obesity (2005). 2014; **38**(6): 873-7.
997. Lardner AL. Neurobiological effects of the green tea constituent theanine and its potential role in the treatment of psychiatric and neurodegenerative disorders. Nutr Neurosci. 2014; **17**(4): 145-55.
998. Lardner AL. Neurobiological effects of the green tea constituent theanine and its potential role in the treatment of psychiatric and neurodegenerative disorders. Nutritional Neuroscience. 2014; **17**(4): 145-55.
999. Lardoeyt R, Vargas G, Lumpuy J, Garcia R, Torres Y. Contribution of genome-environment interaction to pre-eclampsia in a Havana Maternity Hospital. MEDICC Rev. 2013; **15**(3): 22-9.
1000. Lardoeyt R, Vargas G, Lumpuy J, García R, Torres Y. Contribution of genome-environment interaction to pre-eclampsia in a Havana Maternity Hospital. MEDICC Review. 2013; **15**(3): 22-9.
1001. Laroche F, Coste J, Medkour T, Cottu PH, Pierga JY, Lotz JP, et al. Classification of and risk factors for estrogen deprivation pain syndromes related to aromatase inhibitor treatments in women with breast cancer: a prospective multicenter cohort study. J Pain. 2014; **15**(3): 293-303.
1002. Laroche F, Coste J, Medkour T, Cottu PH, Pierga J-Y, Lotz J-P, et al. Classification of and risk factors for estrogen deprivation pain syndromes related to aromatase inhibitor treatments in women with breast cancer: a prospective multicenter cohort study. The Journal Of Pain: Official Journal Of The American Pain Society. 2014; **15**(3): 293-303.
1003. Laske C, Eschweiler GW. [Brain-derived neurotrophic factor: from nerve growth factor to modulator of brain plasticity in cognitive processes and psychiatric diseases]. Nervenarzt. 2006; **77**(5): 523-37.
1004. Laske C, Eschweiler GW. [Brain-derived neurotrophic factor: from nerve growth factor to modulator of brain plasticity in cognitive processes and psychiatric diseases]. Der Nervenarzt. 2006; **77**(5): 523-37.
1005. Lau HH, Ng MY, Ho AY, Luk KD, Kung AW. Genetic and environmental determinants of bone mineral density in Chinese women. Bone. 2005; **36**(4): 700-9.
1006. Lau HHL, Ng MYM, Ho AYY, Luk KDK, Kung AWC. Genetic and environmental determinants of bone mineral density in Chinese women. Bone. 2005; **36**(4): 700-9.
1007. Laucht M, Treutlein J, Schmid B, Blomeyer D, Becker K, Buchmann AF, et al. Impact of psychosocial adversity on alcohol intake in young adults: moderation by the LL genotype of the serotonin transporter polymorphism. Biol Psychiatry. 2009; **66**(2): 102-9.
1008. Laucht M, Treutlein J, Schmid B, Blomeyer D, Becker K, Buchmann AF, et al. Impact of psychosocial adversity on alcohol intake in young adults: moderation by the LL genotype of the serotonin transporter polymorphism. Biological Psychiatry. 2009; **66**(2): 102-9.
1009. Laudenslager ML, Simoneau TL, Philips S, Benitez P, Natvig C, Cole S. A randomized controlled pilot study of inflammatory gene expression in response to a

stress management intervention for stem cell transplant caregivers. J Behav Med. 2016; **39**(2): 346-54.
1010. Laudenslager ML, Simoneau TL, Philips S, Benitez P, Natvig C, Cole S. A randomized controlled pilot study of inflammatory gene expression in response to a stress management intervention for stem cell transplant caregivers. Journal Of Behavioral Medicine. 2016; **39**(2): 346-54.
1011. Lawford BR, Mc DYR, Noble EP, Kann B, Arnold L, Rowell J, et al. D2 dopamine receptor gene polymorphism: paroxetine and social functioning in posttraumatic stress disorder. Eur Neuropsychopharmacol. 2003; **13**(5): 313-20.
1012. Lawford BR, McD Young R, Noble EP, Kann B, Arnold L, Rowell J, et al. D2 dopamine receptor gene polymorphism: paroxetine and social functioning in posttraumatic stress disorder. European Neuropsychopharmacology: The Journal Of The European College Of Neuropsychopharmacology. 2003; **13**(5): 313-20.
1013. Lawford BR, Young RM, Rowell JA, Qualichefski J, Fletcher BH, Syndulko K, et al. Bromocriptine in the treatment of alcoholics with the D2 dopamine receptor A1 allele. Nat Med. 1995; **1**(4): 337-41.
1014. Lawford BR, Young RM, Rowell JA, Qualichefski J, Fletcher BH, Syndulko K, et al. Bromocriptine in the treatment of alcoholics with the D2 dopamine receptor A1 allele. Nature Medicine. 1995; **1**(4): 337-41.
1015. Le Dily F, Bau D, Pohl A, Vicent GP, Serra F, Soronellas D, et al. Distinct structural transitions of chromatin topological domains correlate with coordinated hormone-induced gene regulation. Genes Dev. 2014; **28**(19): 2151-62.
1016. Le Dily F, Baù D, Pohl A, Vicent GP, Serra F, Soronellas D, et al. Distinct structural transitions of chromatin topological domains correlate with coordinated hormone-induced gene regulation. Genes & Development. 2014; **28**(19): 2151-62.
1017. Le Marchand L, Franke AA, Custer L, Wilkens LR, Cooney RV. Lifestyle and nutritional correlates of cytochrome CYP1A2 activity: inverse associations with plasma lutein and alpha-tocopherol. Pharmacogenetics. 1997; **7**(1): 11-9.
1018. Le Marchand L, Franke AA, Custer L, Wilkens LR, Cooney RV. Lifestyle and nutritional correlates of cytochrome CYP1A2 activity: inverse associations with plasma lutein and alpha-tocopherol. Pharmacogenetics. 1997; **7**(1): 11-9.
1019. Le Marchand L, Sivaraman L, Franke AA, Custer LJ, Wilkens LR, Lau AF, et al. Predictors of N-acetyltransferase activity: should caffeine phenotyping and NAT2 genotyping be used interchangeably in epidemiological studies? Cancer Epidemiol Biomarkers Prev. 1996; **5**(6): 449-55.
1020. Le Marchand L, Sivaraman L, Franke AA, Custer LJ, Wilkens LR, Lau AF, et al. Predictors of N-acetyltransferase activity: should caffeine phenotyping and NAT2 genotyping be used interchangeably in epidemiological studies? Cancer Epidemiology, Biomarkers & Prevention: A Publication Of The American Association For Cancer Research, Cosponsored By The American Society Of Preventive Oncology. 1996; **5**(6): 449-55.
1021. LeBaron MJ, Gollapudi BB, Terry C, Billington R, Rasoulpour RJ. Human relevance framework for rodent liver tumors induced by the insecticide sulfoxaflor. Crit Rev Toxicol. 2014; **44 Suppl 2**: 15-24.
1022. LeBaron MJ, Gollapudi BB, Terry C, Billington R, Rasoulpour RJ. Human

relevance framework for rodent liver tumors induced by the insecticide sulfoxaflor. Critical Reviews In Toxicology. 2014; **44 Suppl 2**: 15-24.
1023. Leckie RL, Weinstein AM, Hodzic JC, Erickson KI. Potential moderators of physical activity on brain health. J Aging Res. 2012; **2012**: 948981.
1024. Leckie RL, Weinstein AM, Hodzic JC, Erickson KI. Potential moderators of physical activity on brain health. Journal Of Aging Research. 2012; **2012**: 948981-.
1025. Ledent C, Vaugeois JM, Schiffmann SN, Pedrazzini T, El Yacoubi M, Vanderhaeghen JJ, et al. Aggressiveness, hypoalgesia and high blood pressure in mice lacking the adenosine A2a receptor. Nature. 1997; **388**(6643): 674-8.
1026. Ledent C, Vaugeois JM, Schiffmann SN, Pedrazzini T, El Yacoubi M, Vanderhaeghen JJ, et al. Aggressiveness, hypoalgesia and high blood pressure in mice lacking the adenosine A2a receptor. Nature. 1997; **388**(6643): 674-8.
1027. Lee AW, Hengstler H, Schwald K, Berriel-Diaz M, Loreth D, Kirsch M, et al. Functional inactivation of the genome-wide association study obesity gene neuronal growth regulator 1 in mice causes a body mass phenotype. PLoS One. 2012; **7**(7): e41537.
1028. Lee AWS, Hengstler H, Schwald K, Berriel-Diaz M, Loreth D, Kirsch M, et al. Functional inactivation of the genome-wide association study obesity gene neuronal growth regulator 1 in mice causes a body mass phenotype. PLoS One. 2012; **7**(7): e41537-e.
1029. Lee B, Sur B, Cho SG, Yeom M, Shim I, Lee H, et al. Ginsenoside Rb1 rescues anxiety-like responses in a rat model of post-traumatic stress disorder. J Nat Med. 2016; **70**(2): 133-44.
1030. Lee B, Sur B, Cho S-G, Yeom M, Shim I, Lee H, et al. Ginsenoside Rb1 rescues anxiety-like responses in a rat model of post-traumatic stress disorder. Journal Of Natural Medicines. 2016; **70**(2): 133-44.
1031. Lee HJ, Lee MS, Kang RH, Kim H, Kim SD, Kee BS, et al. Influence of the serotonin transporter promoter gene polymorphism on susceptibility to posttraumatic stress disorder. Depress Anxiety. 2005; **21**(3): 135-9.
1032. Lee H-J, Lee M-S, Kang R-H, Kim H, Kim S-D, Kee B-S, et al. Influence of the serotonin transporter promoter gene polymorphism on susceptibility to posttraumatic stress disorder. Depression And Anxiety. 2005; **21**(3): 135-9.
1033. Lee HK. Method of proof and evidences for the concept that mitochondrial genome is a thrifty genome. Diabetes Res Clin Pract. 2001; **54 Suppl 2**: S57-63.
1034. Lee HK. Method of proof and evidences for the concept that mitochondrial genome is a thrifty genome. Diabetes Research And Clinical Practice. 2001; **54 Suppl 2**: S57-S63.
1035. Lee SH, Choi TK, Lee E, Seok JH, Lee HS, Kim SJ. Serotonin transporter gene polymorphism associated with short-term treatment response to venlafaxine. Neuropsychobiology. 2010; **62**(3): 198-206.
1036. Lee SH, Lee KJ, Lee HJ, Ham BJ, Ryu SH, Lee MS. Association between the 5-HT6 receptor C267T polymorphism and response to antidepressant treatment in major depressive disorder. Psychiatry Clin Neurosci. 2005; **59**(2): 140-5.
1037. Lee S-H, Choi TK, Lee E, Seok J-H, Lee SH, Lee HS, et al. Serotonin transporter gene polymorphism associated with short-term treatment response to venlafaxine.

Neuropsychobiology. 2010; **62**(3): 198-206.
1038. Lee S-H, Lee K-J, Lee H-J, Ham B-J, Ryu S-H, Lee M-S. Association between the 5-HT6 receptor C267T polymorphism and response to antidepressant treatment in major depressive disorder. Psychiatry And Clinical Neurosciences. 2005; **59**(2): 140-5.
1039. Lee SK, Kim SH, Cho GY, Baik I, Lim HE, Park CG, et al. Obesity phenotype and incident hypertension: a prospective community-based cohort study. J Hypertens. 2013; **31**(1): 145-51.
1040. Lee SK, Kim SH, Cho G-Y, Baik I, Lim HE, Park CG, et al. Obesity phenotype and incident hypertension: a prospective community-based cohort study. Journal Of Hypertension. 2013; **31**(1): 145-51.
1041. Lee SS, Yoo JH, Kang S, Woo JH, Shin KO, Kim KB, et al. The Effects of 12 Weeks Regular Aerobic Exercise on Brain-derived Neurotrophic Factor and Inflammatory Factors in Juvenile Obesity and Type 2 Diabetes Mellitus. J Phys Ther Sci. 2014; **26**(8): 1199-204.
1042. Lee SS, Yoo JH, Kang S, Woo JH, Shin KO, Kim KB, et al. The Effects of 12 Weeks Regular Aerobic Exercise on Brain-derived Neurotrophic Factor and Inflammatory Factors in Juvenile Obesity and Type 2 Diabetes Mellitus. Journal Of Physical Therapy Science. 2014; **26**(8): 1199-204.
1043. Leggio L, Zywiak WH, McGeary JE, Edwards S, Fricchione SR, Shoaff JR, et al. A human laboratory pilot study with baclofen in alcoholic individuals. Pharmacol Biochem Behav. 2013; **103**(4): 784-91.
1044. Leggio L, Zywiak WH, McGeary JE, Edwards S, Fricchione SR, Shoaff JR, et al. A human laboratory pilot study with baclofen in alcoholic individuals. Pharmacology, Biochemistry, And Behavior. 2013; **103**(4): 784-91.
1045. Lenze EJ, Goate AM, Nowotny P, Dixon D, Shi P, Bies RR, et al. Relation of serotonin transporter genetic variation to efficacy of escitalopram for generalized anxiety disorder in older adults. J Clin Psychopharmacol. 2010; **30**(6): 672-7.
1046. Lenze EJ, Goate AM, Nowotny P, Dixon D, Shi P, Bies RR, et al. Relation of serotonin transporter genetic variation to efficacy of escitalopram for generalized anxiety disorder in older adults. Journal Of Clinical Psychopharmacology. 2010; **30**(6): 672-7.
1047. Lesch K-P. Alcohol dependence and gene x environment interaction in emotion regulation: Is serotonin the link? European Journal Of Pharmacology. 2005; **526**(1-3): 113-24.
1048. Lesch KP. Alcohol dependence and gene x environment interaction in emotion regulation: Is serotonin the link? Eur J Pharmacol. 2005; **526**(1-3): 113-24.
1049. Lester KJ, Hudson JL, Tropeano M, Creswell C, Collier DA, Farmer A, et al. Neurotrophic gene polymorphisms and response to psychological therapy. Transl Psychiatry. 2012; **2**: e108.
1050. Lester KJ, Hudson JL, Tropeano M, Creswell C, Collier DA, Farmer A, et al. Neurotrophic gene polymorphisms and response to psychological therapy. Translational Psychiatry. 2012; **2**: e108-e.
1051. Lester KJ, Roberts S, Keers R, Coleman JR, Breen G, Wong CC, et al. Non-replication of the association between 5HTTLPR and response to psychological therapy for child anxiety disorders. Br J Psychiatry. 2016; **208**(2): 182-8.
1052. Lester KJ, Roberts S, Keers R, Coleman JRI, Breen G, Wong CCY, et al. Non-

replication of the association between 5HTTLPR and response to psychological therapy for child anxiety disorders. The British Journal Of Psychiatry: The Journal Of Mental Science. 2016; **208**(2): 182-8.
1053. Leung MKK, Delong A, Frey BJ. Inference of the Human Polyadenylation Code. Bioinformatics. 2018.
1054. Leung MKK, Delong A, Frey BJ. Inference of the Human Polyadenylation Code. Bioinformatics (Oxford, England). 2018.
1055. Levada OA, Cherednichenko NV, Trailin AV, Troyan AS. Plasma Brain-Derived Neurotrophic Factor as a Biomarker for the Main Types of Mild Neurocognitive Disorders and Treatment Efficacy: A Preliminary Study. Dis Markers. 2016; **2016**: 4095723.
1056. Levada OA, Cherednichenko NV, Trailin AV, Troyan AS. Plasma Brain-Derived Neurotrophic Factor as a Biomarker for the Main Types of Mild Neurocognitive Disorders and Treatment Efficacy: A Preliminary Study. Disease Markers. 2016; **2016**: 4095723-.
1057. Levey DF, Le-Niculescu H, Frank J, Ayalew M, Jain N, Kirlin B, et al. Genetic risk prediction and neurobiological understanding of alcoholism. Transl Psychiatry. 2014; **4**: e391.
1058. Levey DF, Le-Niculescu H, Frank J, Ayalew M, Jain N, Kirlin B, et al. Genetic risk prediction and neurobiological understanding of alcoholism. Translational Psychiatry. 2014; **4**: e391-e.
1059. Levian C, Ruiz E, Yang X. The pathogenesis of obesity from a genomic and systems biology perspective. Yale J Biol Med. 2014; **87**(2): 113-26.
1060. Levian C, Ruiz E, Yang X. The pathogenesis of obesity from a genomic and systems biology perspective. The Yale Journal Of Biology And Medicine. 2014; **87**(2): 113-26.
1061. Li J, Batcha AM, Gruning B, Mansmann UR. An NGS Workflow Blueprint for DNA Sequencing Data and Its Application in Individualized Molecular Oncology. Cancer Inform. 2015; **14**(Suppl 5): 87-107.
1062. Li J, Batcha AMN, Grüning B, Mansmann UR. An NGS Workflow Blueprint for DNA Sequencing Data and Its Application in Individualized Molecular Oncology. Cancer Informatics. 2016; **14**(Suppl 5): 87-107.
1063. Li L, Gao K, Zhao J, Feng T, Yin L, Wang J, et al. Glucagon gene polymorphism modifies the effects of smoking and physical activity on risk of type 2 diabetes mellitus in Han Chinese. Gene. 2014; **534**(2): 352-5.
1064. Li L, Gao K, Zhao J, Feng T, Yin L, Wang J, et al. Glucagon gene polymorphism modifies the effects of smoking and physical activity on risk of type 2 diabetes mellitus in Han Chinese. Gene. 2014; **534**(2): 352-5.
1065. Li L, Plummer SJ, Thompson CL, Tucker TC, Casey G. Association between phosphatidylinositol 3-kinase regulatory subunit p85alpha Met326Ile genetic polymorphism and colon cancer risk. Clin Cancer Res. 2008; **14**(3): 633-7.
1066. Li L, Plummer SJ, Thompson CL, Tucker TC, Casey G. Association between phosphatidylinositol 3-kinase regulatory subunit p85alpha Met326Ile genetic polymorphism and colon cancer risk. Clinical Cancer Research: An Official Journal Of The American Association For Cancer Research. 2008; **14**(3): 633-7.

1067. Li TY, Zhang C, Asselbergs FW, Qi L, Rimm E, Hunter DJ, et al. Interaction between dietary fat intake and the cholesterol ester transfer protein TaqIB polymorphism in relation to HDL-cholesterol concentrations among US diabetic men. Am J Clin Nutr. 2007; **86**(5): 1524-9.
1068. Li TY, Zhang C, Asselbergs FW, Qi L, Rimm E, Hunter DJ, et al. Interaction between dietary fat intake and the cholesterol ester transfer protein TaqIB polymorphism in relation to HDL-cholesterol concentrations among US diabetic men. The American Journal Of Clinical Nutrition. 2007; **86**(5): 1524-9.
1069. Li WJ, Yu H, Yang JM, Gao J, Jiang H, Feng M, et al. Anxiolytic effect of music exposure on BDNFMet/Met transgenic mice. Brain Res. 2010; **1347**: 71-9.
1070. Li W-J, Yu H, Yang J-M, Gao J, Jiang H, Feng M, et al. Anxiolytic effect of music exposure on BDNFMet/Met transgenic mice. Brain Research. 2010; **1347**: 71-9.
1071. Li X, He GP, Zhang B, Chen YM, Su YX. Interactions of interleukin-6 gene polymorphisms with calcium intake and physical activity on bone mass in pre-menarche Chinese girls. Osteoporos Int. 2008; **19**(11): 1629-37.
1072. Li X, He GP, Zhang B, Chen YM, Su YX. Interactions of interleukin-6 gene polymorphisms with calcium intake and physical activity on bone mass in pre-menarche Chinese girls. Osteoporosis International: A Journal Established As Result Of Cooperation Between The European Foundation For Osteoporosis And The National Osteoporosis Foundation Of The USA. 2008; **19**(11): 1629-37.
1073. Li YJ, Luo HC, Qian RQ. [Effect of Danzhi Xiaoyao Powder on neuro-immuno-endocrine system in patients with depression]. Zhongguo Zhong Xi Yi Jie He Za Zhi. 2007; **27**(3): 197-200.
1074. Li Y-j, Luo H-c, Qian R-q. [Effect of Danzhi Xiaoyao Powder on neuro-immuno-endocrine system in patients with depression]. Zhongguo Zhong Xi Yi Jie He Za Zhi Zhongguo Zhongxiyi Jiehe Zazhi = Chinese Journal Of Integrated Traditional And Western Medicine. 2007; **27**(3): 197-200.
1075. Licinio J, O'Kirwan F, Irizarry K, Merriman B, Thakur S, Jepson R, et al. Association of a corticotropin-releasing hormone receptor 1 haplotype and antidepressant treatment response in Mexican-Americans. Mol Psychiatry. 2004; **9**(12): 1075-82.
1076. Licinio J, O'Kirwan F, Irizarry K, Merriman B, Thakur S, Jepson R, et al. Association of a corticotropin-releasing hormone receptor 1 haplotype and antidepressant treatment response in Mexican-Americans. Molecular Psychiatry. 2004; **9**(12): 1075-82.
1077. Lifanov D, Khadyeva MN, Rahmatullina L, Demenev SV, Ibragimov RR. Effect of creatine supplementation on physical performance are related to the AMPD1 and PPARG genes polymorphisms in football players. Ross Fiziol Zh Im I M Sechenova. 2014; **100**(6): 767-76.
1078. Lifanov D, Khadyeva MN, Rahmatullina LS, Demenev SV, Ibragimov RR. Effect of creatine supplementation on physical performance are related to the AMPD1 and PPARG genes polymorphisms in football players. Rossiiskii Fiziologicheskii Zhurnal Imeni IM Sechenova. 2014; **100**(6): 767-76.
1079. Limdi N, Goldstein J, Blaisdell J, Beasley T, Rivers C, Acton R. Influence of CYP2C9 Genotype on warfarin dose among African American and European

Americans. Per Med. 2007; **4**(2): 157-69.
1080. Limdi N, Goldstein J, Blaisdell J, Beasley T, Rivers C, Acton R. Influence of CYP2C9 Genotype on warfarin dose among African American and European Americans. Personalized Medicine. 2007; **4**(2): 157-69.
1081. Lin E, Tsai SJ, Kuo PH, Liu YL, Yang AC, Kao CF. Association and interaction effects of Alzheimer's disease-associated genes and lifestyle on cognitive aging in older adults in a Taiwanese population. Oncotarget. 2017; **8**(15): 24077-87.
1082. Lin E, Tsai S-J, Kuo P-H, Liu Y-L, Yang AC, Kao C-F. Association and interaction effects of Alzheimer's disease-associated genes and lifestyle on cognitive aging in older adults in a Taiwanese population. Oncotarget. 2017; **8**(15): 24077-87.
1083. Lin KM, Tsou HH, Tsai IJ, Hsiao MC, Hsiao CF, Liu CY, et al. CYP1A2 genetic polymorphisms are associated with treatment response to the antidepressant paroxetine. Pharmacogenomics. 2010; **11**(11): 1535-43.
1084. Lin K-M, Tsou H-H, Tsai IJ, Hsiao M-C, Hsiao C-F, Liu C-Y, et al. CYP1A2 genetic polymorphisms are associated with treatment response to the antidepressant paroxetine. Pharmacogenomics. 2010; **11**(11): 1535-43.
1085. Lin M, Rikihisa Y. Ehrlichia chaffeensis and Anaplasma phagocytophilum lack genes for lipid A biosynthesis and incorporate cholesterol for their survival. Infect Immun. 2003; **71**(9): 5324-31.
1086. Lin M, Rikihisa Y. Ehrlichia chaffeensis and Anaplasma phagocytophilum lack genes for lipid A biosynthesis and incorporate cholesterol for their survival. Infection And Immunity. 2003; **71**(9): 5324-31.
1087. Lin M, Zhu J, Yuan Y, Ren L, Qian M, Shen Z, et al. Association Analysis of the Brain-Derived Neurotrophic Factor Gene Val66Met Polymorphism and Gender with Efficacy of Antidepressants in the Chinese Han Population with Generalized Anxiety Disorder. Genet Test Mol Biomarkers. 2018; **22**(3): 199-206.
1088. Lin M, Zhu J, Yuan Y, Ren L, Qian M, Shen Z, et al. Association Analysis of the Brain-Derived Neurotrophic Factor Gene Val66Met Polymorphism and Gender with Efficacy of Antidepressants in the Chinese Han Population with Generalized Anxiety Disorder. Genetic Testing And Molecular Biomarkers. 2018; **22**(3): 199-206.
1089. Lin X, Eaton CB, Manson JE, Liu S. The Genetics of Physical Activity. Curr Cardiol Rep. 2017; **19**(12): 119.
1090. Lin X, Eaton CB, Manson JE, Liu S. The Genetics of Physical Activity. Current Cardiology Reports. 2017; **19**(12): 119-.
1091. Lind PA, Eriksson CJ, Wilhelmsen KC. Association between harmful alcohol consumption behavior and dopamine transporter (DAT1) gene polymorphisms in a male Finnish population. Psychiatr Genet. 2009; **19**(3): 117-25.
1092. Lind PA, Eriksson CJP, Wilhelmsen KC. Association between harmful alcohol consumption behavior and dopamine transporter (DAT1) gene polymorphisms in a male Finnish population. Psychiatric Genetics. 2009; **19**(3): 117-25.
1093. Lintel NJ, Brown DK, Schafer DT, Tsimba-Chitsva FM, Koepsell SA, Shunkwiler SM. Use of standard laboratory methods to obviate routine dithiothreitol treatment of blood samples with daratumumab interference. Immunohematology. 2017; **33**(1): 22-6.
1094. Lintel NJ, Brown DK, Schafer DT, Tsimba-Chitsva FM, Koepsell SA, Shunkwiler SM. Use of standard laboratory methods to obviate routine dithiothreitol treatment of

blood samples with daratumumab interference. Immunohematology. 2017; **33**(1): 22-6.
1095. Lipov EG, Candido K, Ritchie EC. Possible Reversal of PTSD-Related DNA Methylation by Sympathetic Blockade. J Mol Neurosci. 2017; **62**(1): 67-72.
1096. Lipov EG, Candido K, Ritchie EC. Possible Reversal of PTSD-Related DNA Methylation by Sympathetic Blockade. Journal Of Molecular Neuroscience: MN. 2017; **62**(1): 67-72.
1097. Lips MA, Syddall HE, Gaunt TR, Rodriguez S, Day IN, Cooper C, et al. Interaction between birthweight and polymorphism in the calcium-sensing receptor gene in determination of adult bone mass: the Hertfordshire cohort study. J Rheumatol. 2007; **34**(4): 769-75.
1098. Lips MA, Syddall HE, Gaunt TR, Rodriguez S, Day INM, Cooper C, et al. Interaction between birthweight and polymorphism in the calcium-sensing receptor gene in determination of adult bone mass: the Hertfordshire cohort study. The Journal Of Rheumatology. 2007; **34**(4): 769-75.
1099. Little J, Higgins JPT, Ioannidis JPA, Moher D, Gagnon F, von Elm E, et al. Strengthening the reporting of genetic association studies (STREGA): an extension of the STROBE Statement. Human Genetics. 2009; **125**(2): 131-51.
1100. Little J, Higgins JPT, Ioannidis JPA, Moher D, Gagnon F, von Elm E, et al. Strengthening the reporting of genetic association studies (STREGA): an extension of the STROBE statement. European Journal Of Epidemiology. 2009; **24**(1): 37-55.
1101. Little J, Higgins JPT, Ioannidis JPA, Moher D, Gagnon F, von Elm E, et al. STrengthening the REporting of Genetic Association Studies (STREGA): an extension of the STROBE statement. Plos Medicine. 2009; **6**(2): e22-e.
1102. Little J, Higgins JPT, Ioannidis JPA, Moher D, Gagnon F, von Elm E, et al. STrengthening the REporting of Genetic Association studies (STREGA)--an extension of the STROBE statement. European Journal Of Clinical Investigation. 2009; **39**(4): 247-66.
1103. Little J, Higgins JPT, Ioannidis JPA, Moher D, Gagnon F, von Elm E, et al. STrengthening the REporting of Genetic Association Studies (STREGA)--an extension of the STROBE statement. Genetic Epidemiology. 2009; **33**(7): 581-98.
1104. Liu A, Menon S, Colson NJ, Quinlan S, Cox H, Peterson M, et al. Analysis of the MTHFR C677T variant with migraine phenotypes. BMC Res Notes. 2010; **3**: 213.
1105. Liu A, Menon S, Colson NJ, Quinlan S, Cox H, Peterson M, et al. Analysis of the MTHFR C677T variant with migraine phenotypes. BMC Research Notes. 2010; **3**: 213-.
1106. Liu G, Zhu H, Lagou V, Gutin B, Stallmann-Jorgensen IS, Treiber FA, et al. FTO variant rs9939609 is associated with body mass index and waist circumference, but not with energy intake or physical activity in European- and African-American youth. BMC Med Genet. 2010; **11**: 57.
1107. Liu G, Zhu H, Lagou V, Gutin B, Stallmann-Jorgensen IS, Treiber FA, et al. FTO variant rs9939609 is associated with body mass index and waist circumference, but not with energy intake or physical activity in European- and African-American youth. BMC Medical Genetics. 2010; **11**: 57-.
1108. Liu M, Hammers DW, Barton ER, Sweeney HL. Activin Receptor Type IIB Inhibition Improves Muscle Phenotype and Function in a Mouse Model of Spinal Muscular Atrophy. PLoS One. 2016; **11**(11): e0166803.

1109. Liu M, Hammers DW, Barton ER, Sweeney HL. Activin Receptor Type IIB Inhibition Improves Muscle Phenotype and Function in a Mouse Model of Spinal Muscular Atrophy. PLoS One. 2016; **11**(11): e0166803-e.
1110. Liu M, Zhang J, Guo Z, Wu M, Chen Q, Zhou Z, et al. [Association and interaction between 10 SNP of peroxisome proliferator-activated receptor and non-HDL-C]. Zhonghua Yu Fang Yi Xue Za Zhi. 2015; **49**(3): 259-64.
1111. Liu M, Zhang J, Guo Z, Wu M, Chen Q, Zhou Z, et al. [Association and interaction between 10 SNP of peroxisome proliferator-activated receptor and non-HDL-C]. Zhonghua Yu Fang Yi Xue Za Zhi [Chinese Journal Of Preventive Medicine]. 2015; **49**(3): 259-64.
1112. Liu Y, Jia L, Jiang SM, Chen DY, Song JS, Xu J. Serotonin Transporter Gene (SLC6A4) Polymorphism May Be Associated with Chinese Globus Pharyngeus and Its Antidepressant Effects. Digestion. 2018; **97**(2): 146-53.
1113. Liu Y, Jia L, Jiang S-M, Chen D-Y, Song J-S, Xu J. Serotonin Transporter Gene (SLC6A4) Polymorphism May Be Associated with Chinese Globus Pharyngeus and Its Antidepressant Effects. Digestion. 2018; **97**(2): 146-53.
1114. Liu Z, Zhu F, Wang G, Xiao Z, Tang J, Liu W, et al. Association study of corticotropin-releasing hormone receptor1 gene polymorphisms and antidepressant response in major depressive disorders. Neurosci Lett. 2007; **414**(2): 155-8.
1115. Liu Z, Zhu F, Wang G, Xiao Z, Tang J, Liu W, et al. Association study of corticotropin-releasing hormone receptor1 gene polymorphisms and antidepressant response in major depressive disorders. Neuroscience Letters. 2007; **414**(2): 155-8.
1116. Livingstone KM, Celis-Morales C, Navas-Carretero S, San-Cristobal R, Forster H, O'Donovan CB, et al. Fat mass- and obesity-associated genotype, dietary intakes and anthropometric measures in European adults: the Food4Me study. Br J Nutr. 2016; **115**(3): 440-8.
1117. Livingstone KM, Celis-Morales C, Navas-Carretero S, San-Cristobal R, Forster H, O'Donovan CB, et al. Fat mass- and obesity-associated genotype, dietary intakes and anthropometric measures in European adults: the Food4Me study. The British Journal Of Nutrition. 2016; **115**(3): 440-8.
1118. Livingstone KM, Celis-Morales C, Navas-Carretero S, San-Cristobal R, Macready AL, Fallaize R, et al. Effect of an Internet-based, personalized nutrition randomized trial on dietary changes associated with the Mediterranean diet: the Food4Me Study. Am J Clin Nutr. 2016; **104**(2): 288-97.
1119. Livingstone KM, Celis-Morales C, Navas-Carretero S, San-Cristobal R, Macready AL, Fallaize R, et al. Effect of an Internet-based, personalized nutrition randomized trial on dietary changes associated with the Mediterranean diet: the Food4Me Study. The American Journal Of Clinical Nutrition. 2016; **104**(2): 288-97.
1120. Livingstone KM, Celis-Morales C, Papandonatos GD, Erar B, Florez JC, Jablonski KA, et al. FTO genotype and weight loss: systematic review and meta-analysis of 9563 individual participant data from eight randomised controlled trials. BMJ. 2016; **354**: i4707.
1121. Livingstone KM, Celis-Morales C, Papandonatos GD, Erar B, Florez JC, Jablonski KA, et al. FTO genotype and weight loss: systematic review and meta-analysis of 9563 individual participant data from eight randomised controlled trials. BMJ

(Clinical Research Ed). 2016; **354**: i4707-i.
1122. Lochner C, Hemmings SM, Kinnear CJ, Nel D, Seedat S, Moolman-Smook JC, et al. Cluster analysis of obsessive-compulsive symptomatology: identifying obsessive-compulsive disorder subtypes. Isr J Psychiatry Relat Sci. 2008; **45**(3): 164-76.
1123. Lochner C, Hemmings SMJ, Kinnear CJ, Nel D, Seedat S, Moolman-Smook JC, et al. Cluster analysis of obsessive-compulsive symptomatology: identifying obsessive-compulsive disorder subtypes. The Israel Journal Of Psychiatry And Related Sciences. 2008; **45**(3): 164-76.
1124. Lof A, Johanson G. Toxicokinetics of organic solvents: a review of modifying factors. Crit Rev Toxicol. 1998; **28**(6): 571-650.
1125. Löf A, Johanson G. Toxicokinetics of organic solvents: a review of modifying factors. Critical Reviews In Toxicology. 1998; **28**(6): 571-650.
1126. Lohoff FW, Aquino TD, Narasimhan S, Multani PK, Etemad B, Rickels K. Serotonin receptor 2A (HTR2A) gene polymorphism predicts treatment response to venlafaxine XR in generalized anxiety disorder. Pharmacogenomics J. 2013; **13**(1): 21-6.
1127. Lohoff FW, Aquino TD, Narasimhan S, Multani PK, Etemad B, Rickels K. Serotonin receptor 2A (HTR2A) gene polymorphism predicts treatment response to venlafaxine XR in generalized anxiety disorder. The Pharmacogenomics Journal. 2013; **13**(1): 21-6.
1128. Lohoff FW, Narasimhan S, Rickels K. Interaction between polymorphisms in serotonin transporter (SLC6A4) and serotonin receptor 2A (HTR2A) genes predict treatment response to venlafaxine XR in generalized anxiety disorder. Pharmacogenomics J. 2013; **13**(5): 464-9.
1129. Lohoff FW, Narasimhan S, Rickels K. Interaction between polymorphisms in serotonin transporter (SLC6A4) and serotonin receptor 2A (HTR2A) genes predict treatment response to venlafaxine XR in generalized anxiety disorder. The Pharmacogenomics Journal. 2013; **13**(5): 464-9.
1130. Lonsdorf TB, Golkar A, Lindstrom KM, Haaker J, Ohman A, Schalling M, et al. BDNFval66met affects neural activation pattern during fear conditioning and 24 h delayed fear recall. Soc Cogn Affect Neurosci. 2015; **10**(5): 664-71.
1131. Lonsdorf TB, Golkar A, Lindström KM, Haaker J, Öhman A, Schalling M, et al. BDNFval66met affects neural activation pattern during fear conditioning and 24 h delayed fear recall. Social Cognitive And Affective Neuroscience. 2015; **10**(5): 664-71.
1132. Lonsdorf TB, Kalisch R. A review on experimental and clinical genetic associations studies on fear conditioning, extinction and cognitive-behavioral treatment. Transl Psychiatry. 2011; **1**: e41.
1133. Lonsdorf TB, Kalisch R. A review on experimental and clinical genetic associations studies on fear conditioning, extinction and cognitive-behavioral treatment. Translational Psychiatry. 2011; **1**: e41-e.
1134. Lonsdorf TB, Ruck C, Bergstrom J, Andersson G, Ohman A, Lindefors N, et al. The COMTval158met polymorphism is associated with symptom relief during exposure-based cognitive-behavioral treatment in panic disorder. BMC Psychiatry. 2010; **10**: 99.
1135. Lonsdorf TB, Rück C, Bergström J, Andersson G, Ohman A, Lindefors N, et al. The COMTval158met polymorphism is associated with symptom relief during exposure-

based cognitive-behavioral treatment in panic disorder. BMC Psychiatry. 2010; **10**: 99-.
1136. Lonsdorf TB, Weike AI, Nikamo P, Schalling M, Hamm AO, Ohman A. Genetic gating of human fear learning and extinction: possible implications for gene-environment interaction in anxiety disorder. Psychol Sci. 2009; **20**(2): 198-206.
1137. Lonsdorf TB, Weike AI, Nikamo P, Schalling M, Hamm AO, Ohman A. Genetic gating of human fear learning and extinction: possible implications for gene-environment interaction in anxiety disorder. Psychological Science. 2009; **20**(2): 198-206.
1138. Loos RJ, Yeo GS. The bigger picture of FTO: the first GWAS-identified obesity gene. Nat Rev Endocrinol. 2014; **10**(1): 51-61.
1139. Loos RJF, Yeo GSH. The bigger picture of FTO: the first GWAS-identified obesity gene. Nature Reviews Endocrinology. 2014; **10**(1): 51-61.
1140. Lopera J, Miller IJ, McPhail KL, Kwan JC. Increased Biosynthetic Gene Dosage in a Genome-Reduced Defensive Bacterial Symbiont. mSystems. 2017; **2**(6).
1141. Lopera J, Miller IJ, McPhail KL, Kwan JC. Increased Biosynthetic Gene Dosage in a Genome-Reduced Defensive Bacterial Symbiont. mSystems. 2017; **2**(6).
1142. Lopresti AL, Drummond PD. Obesity and psychiatric disorders: commonalities in dysregulated biological pathways and their implications for treatment. Prog Neuropsychopharmacol Biol Psychiatry. 2013; **45**: 92-9.
1143. Lopresti AL, Drummond PD. Obesity and psychiatric disorders: commonalities in dysregulated biological pathways and their implications for treatment. Progress In Neuro-Psychopharmacology & Biological Psychiatry. 2013; **45**: 92-9.
1144. Lorentzon M, Eriksson AL, Nilsson S, Mellstrom D, Ohlsson C. Association between physical activity and BMD in young men is modulated by catechol-O-methyltransferase (COMT) genotype: the GOOD study. J Bone Miner Res. 2007; **22**(8): 1165-72.
1145. Lorentzon M, Eriksson AL, Nilsson S, Mellström D, Ohlsson C. Association between physical activity and BMD in young men is modulated by catechol-O-methyltransferase (COMT) genotype: the GOOD study. Journal Of Bone And Mineral Research: The Official Journal Of The American Society For Bone And Mineral Research. 2007; **22**(8): 1165-72.
1146. Lotan A, Fenckova M, Bralten J, Alttoa A, Dixson L, Williams RW, et al. Neuroinformatic analyses of common and distinct genetic components associated with major neuropsychiatric disorders. Front Neurosci. 2014; **8**: 331.
1147. Lotan A, Fenckova M, Bralten J, Alttoa A, Dixson L, Williams RW, et al. Neuroinformatic analyses of common and distinct genetic components associated with major neuropsychiatric disorders. Frontiers In Neuroscience. 2014; **8**: 331-.
1148. Lott DC, Kim SJ, Cook EH, Jr., de Wit H. Dopamine transporter gene associated with diminished subjective response to amphetamine. Neuropsychopharmacology. 2005; **30**(3): 602-9.
1149. Lott DC, Kim S-J, Cook EH, Jr., de Wit H. Dopamine transporter gene associated with diminished subjective response to amphetamine. Neuropsychopharmacology: Official Publication Of The American College Of Neuropsychopharmacology. 2005; **30**(3): 602-9.
1150. Lucht MJ, Kuehn KU, Schroeder W, Armbruster J, Abraham G, Schattenberg A, et al. Influence of the dopamine D2 receptor (DRD2) exon 8 genotype on efficacy of

tiapride and clinical outcome of alcohol withdrawal. Pharmacogenetics. 2001; **11**(8): 647-53.
1151. Lucht MJ, Kuehn KU, Schroeder W, Armbruster J, Abraham G, Schattenberg A, et al. Influence of the dopamine D2 receptor (DRD2) exon 8 genotype on efficacy of tiapride and clinical outcome of alcohol withdrawal. Pharmacogenetics. 2001; **11**(8): 647-53.
1152. Luciano M, Zhu G, Kirk KM, Gordon SD, Heath AC, Montgomery GW, et al. "No thanks, it keeps me awake": the genetics of coffee-attributed sleep disturbance. Sleep. 2007; **30**(10): 1378-86.
1153. Lueken U, Straube B, Wittchen HU, Konrad C, Strohle A, Wittmann A, et al. Therapygenetics: anterior cingulate cortex-amygdala coupling is associated with 5-HTTLPR and treatment response in panic disorder with agoraphobia. J Neural Transm (Vienna). 2015; **122**(1): 135-44.
1154. Lueken U, Straube B, Wittchen H-U, Konrad C, Ströhle A, Wittmann A, et al. Therapygenetics: anterior cingulate cortex-amygdala coupling is associated with 5-HTTLPR and treatment response in panic disorder with agoraphobia. Journal Of Neural Transmission (Vienna, Austria: 1996). 2015; **122**(1): 135-44.
1155. Luglio HF, Sulistyoningrum DC, Huriyati E, Lee YY, Wan Muda WAM. The Gene-Lifestyle Interaction on Leptin Sensitivity and Lipid Metabolism in Adults: A Population Based Study. Nutrients. 2017; **9**(7).
1156. Luglio HF, Sulistyoningrum DC, Huriyati E, Lee YY, Wan Muda WAM. The Gene-Lifestyle Interaction on Leptin Sensitivity and Lipid Metabolism in Adults: A Population Based Study. Nutrients. 2017; **9**(7).
1157. Lunegova OS, Kerimkulova AS, Mirrakhimov AE, Abilova SS, Zalesskaia Iu V, Alibaeva NT, et al. [An association of the 825 polymorphism of the g protein 3 subunit with obesity in Kyrgyz population]. Kardiologiia. 2013; **53**(4): 55-61.
1158. Lunegova OS, Kerimkulova AS, Mirrakhimov AE, Abilova SS, Zalesskaia IV, Alibaeva NT, et al. [An association of the 825 polymorphism of the g protein 3 subunit with obesity in Kyrgyz population]. Kardiologiia. 2013; **53**(4): 55-61.
1159. Luojus MK, Lehto SM, Tolmunen T, Brem AK, Lonnroos E, Kauhanen J. Self-reported sleep disturbance and incidence of dementia in ageing men. J Epidemiol Community Health. 2017; **71**(4): 329-35.
1160. Luojus MK, Lehto SM, Tolmunen T, Brem A-K, Lönnroos E, Kauhanen J. Self-reported sleep disturbance and incidence of dementia in ageing men. Journal Of Epidemiology And Community Health. 2017; **71**(4): 329-35.
1161. Lyman GH. Risk factors for cancer. Prim Care. 1992; **19**(3): 465-79.
1162. Lyman GH. Risk factors for cancer. Primary Care. 1992; **19**(3): 465-79.
1163. Lynch WJ, Peterson AB, Sanchez V, Abel J, Smith MA. Exercise as a novel treatment for drug addiction: a neurobiological and stage-dependent hypothesis. Neurosci Biobehav Rev. 2013; **37**(8): 1622-44.
1164. Lynch WJ, Peterson AB, Sanchez V, Abel J, Smith MA. Exercise as a novel treatment for drug addiction: a neurobiological and stage-dependent hypothesis. Neuroscience And Biobehavioral Reviews. 2013; **37**(8): 1622-44.
1165. Mac Sweeney EJ, Oades PJ, Buchdahl R, Rosenthal M, Bush A. Relation of thickening of colon wall to pancreatic-enzyme treatment in cystic fibrosis. Lancet. 1995;

345(8952): 752-6.
1166. Mac Sweeney EJ, Oades PJ, Buchdahl R, Rosenthal M, Bush A. Relation of thickening of colon wall to pancreatic-enzyme treatment in cystic fibrosis. Lancet (London, England). 1995; **345**(8952): 752-6.
1167. Macdonald HM, McGuigan FE, Stewart A, Black AJ, Fraser WD, Ralston S, et al. Large-scale population-based study shows no evidence of association between common polymorphism of the VDR gene and BMD in British women. J Bone Miner Res. 2006; **21**(1): 151-62.
1168. Macdonald HM, McGuigan FE, Stewart A, Black AJ, Fraser WD, Ralston S, et al. Large-scale population-based study shows no evidence of association between common polymorphism of the VDR gene and BMD in British women. Journal Of Bone And Mineral Research: The Official Journal Of The American Society For Bone And Mineral Research. 2006; **21**(1): 151-62.
1169. MacQueen G, Born L, Steiner M. The selective serotonin reuptake inhibitor sertraline: its profile and use in psychiatric disorders. CNS Drug Rev. 2001; **7**(1): 1-24.
1170. MacQueen G, Born L, Steiner M. The selective serotonin reuptake inhibitor sertraline: its profile and use in psychiatric disorders. CNS Drug Reviews. 2001; **7**(1): 1-24.
1171. Maemoto T, Tada M, Mihara T, Ueyama N, Matsuoka H, Harada K, et al. Pharmacological characterization of FR194921, a new potent, selective, and orally active antagonist for central adenosine A1 receptors. J Pharmacol Sci. 2004; **96**(1): 42-52.
1172. Maemoto T, Tada M, Mihara T, Ueyama N, Matsuoka H, Harada K, et al. Pharmacological characterization of FR194921, a new potent, selective, and orally active antagonist for central adenosine A1 receptors. Journal Of Pharmacological Sciences. 2004; **96**(1): 42-52.
1173. Maffeis C. Childhood obesity: the genetic-environmental interface. Baillieres Best Pract Res Clin Endocrinol Metab. 1999; **13**(1): 31-46.
1174. Maffeis C. Childhood obesity: the genetic-environmental interface. Bailliere's Best Practice & Research Clinical Endocrinology & Metabolism. 1999; **13**(1): 31-46.
1175. Magnet A, Gomes TS, Pardinas C, Garcia de Blas N, Sadaba C, Carrillo E, et al. Can artificial tears prevent Acanthamoeba keratitis? An in vitro approach. Parasit Vectors. 2018; **11**(1): 50.
1176. Magnet A, Gomes TS, Pardinas C, Garcia de Blas N, Sadaba C, Carrillo E, et al. Can artificial tears prevent Acanthamoeba keratitis? An in vitro approach. Parasites & Vectors. 2018; **11**(1): 50-.
1177. Mahan AL, Ressler KJ. Fear conditioning, synaptic plasticity and the amygdala: implications for posttraumatic stress disorder. Trends Neurosci. 2012; **35**(1): 24-35.
1178. Mahan AL, Ressler KJ. Fear conditioning, synaptic plasticity and the amygdala: implications for posttraumatic stress disorder. Trends In Neurosciences. 2012; **35**(1): 24-35.
1179. Mahut B, Plantier L, Malinvaud D, Chevalier-Bidaud B, Bonfils P, Delclaux C. Pathophysiology of airway hyperresponsiveness in patients with nasal polyposis. Respir Med. 2012; **106**(1): 68-74.
1180. Mahut B, Plantier L, Malinvaud D, Chevalier-Bidaud B, Bonfils P, Delclaux C.

Pathophysiology of airway hyperresponsiveness in patients with nasal polyposis. Respiratory Medicine. 2012; **106**(1): 68-74.
1181. Makmor-Bakry M, Sills GJ, Hitiris N, Butler E, Wilson EA, Brodie MJ. Genetic variants in microsomal epoxide hydrolase influence carbamazepine dosing. Clin Neuropharmacol. 2009; **32**(4): 205-12.
1182. Makmor-Bakry M, Sills GJ, Hitiris N, Butler E, Wilson EA, Brodie MJ. Genetic variants in microsomal epoxide hydrolase influence carbamazepine dosing. Clinical Neuropharmacology. 2009; **32**(4): 205-12.
1183. Malan-Muller S, Seedat S, Hemmings SM. Understanding posttraumatic stress disorder: insights from the methylome. Genes Brain Behav. 2014; **13**(1): 52-68.
1184. Malan-Müller S, Seedat S, Hemmings SMJ. Understanding posttraumatic stress disorder: insights from the methylome. Genes, Brain, And Behavior. 2014; **13**(1): 52-68.
1185. Mandelli L, Emiliani R, Porcelli S, Fabbri C, Albani D, Serretti A. Genes involved in neuroplasticity and stressful life events act on the short-term response to antidepressant treatment: a complex interplay between genetics and environment. Hum Psychopharmacol. 2014; **29**(4): 388-91.
1186. Mandelli L, Emiliani R, Porcelli S, Fabbri C, Albani D, Serretti A. Genes involved in neuroplasticity and stressful life events act on the short-term response to antidepressant treatment: a complex interplay between genetics and environment. Human Psychopharmacology. 2014; **29**(4): 388-91.
1187. Mandelli L, Mazza M, Martinotti G, Di Nicola M, Tavian D, Colombo E, et al. Harm avoidance moderates the influence of serotonin transporter gene variants on treatment outcome in bipolar patients. J Affect Disord. 2009; **119**(1-3): 205-9.
1188. Mandelli L, Mazza M, Martinotti G, Di Nicola M, Tavian D, Colombo E, et al. Harm avoidance moderates the influence of serotonin transporter gene variants on treatment outcome in bipolar patients. Journal Of Affective Disorders. 2009; **119**(1-3): 205-9.
1189. Mandrekar SJ, Sargent DJ. Clinical trial designs for predictive biomarker validation: one size does not fit all. J Biopharm Stat. 2009; **19**(3): 530-42.
1190. Mandrekar SJ, Sargent DJ. Clinical trial designs for predictive biomarker validation: one size does not fit all. Journal Of Biopharmaceutical Statistics. 2009; **19**(3): 530-42.
1191. Mandrekar SJ, Sargent DJ. Drug designs fulfilling the requirements of clinical trials aiming at personalizing medicine. Chin Clin Oncol. 2014; **3**(2): 14.
1192. Mandrekar SJ, Sargent DJ. Drug designs fulfilling the requirements of clinical trials aiming at personalizing medicine. Chinese Clinical Oncology. 2014; **3**(2): 14-.
1193. Mann TN, Lamberts RP, Lambert MI. High responders and low responders: factors associated with individual variation in response to standardized training. Sports Med. 2014; **44**(8): 1113-24.
1194. Mann TN, Lamberts RP, Lambert MI. High responders and low responders: factors associated with individual variation in response to standardized training. Sports Medicine (Auckland, NZ). 2014; **44**(8): 1113-24.
1195. Manolides AS, Cullen DM, Akhter MP. Effects of glucocorticoid treatment on bone strength. J Bone Miner Metab. 2010; **28**(5): 532-9.
1196. Manolides AS, Cullen DM, Akhter MP. Effects of glucocorticoid treatment on bone strength. Journal Of Bone And Mineral Metabolism. 2010; **28**(5): 532-9.

1197. Marc J, Prezelj J, Komel R, Kocijancic A. Association of vitamin D receptor gene polymorphism with bone mineral density in Slovenian postmenopausal women. Gynecol Endocrinol. 2000; **14**(1): 60-4.
1198. Marc J, Prezelj J, Komel R, Kocijancic A. Association of vitamin D receptor gene polymorphism with bone mineral density in Slovenian postmenopausal women. Gynecological Endocrinology: The Official Journal Of The International Society Of Gynecological Endocrinology. 2000; **14**(1): 60-4.
1199. Marcinkiewcz CA, Lowery-Gionta EG, Kash TL. Serotonin's Complex Role in Alcoholism: Implications for Treatment and Future Research. Alcohol Clin Exp Res. 2016; **40**(6): 1192-201.
1200. Marcinkiewcz CA, Lowery-Gionta EG, Kash TL. Serotonin's Complex Role in Alcoholism: Implications for Treatment and Future Research. Alcoholism, Clinical And Experimental Research. 2016; **40**(6): 1192-201.
1201. Markett S, Montag C, Reuter M. The nicotinic acetylcholine receptor gene CHRNA4 is associated with negative emotionality. Emotion. 2011; **11**(2): 450-5.
1202. Markett S, Montag C, Reuter M. The nicotinic acetylcholine receptor gene CHRNA4 is associated with negative emotionality. Emotion (Washington, DC). 2011; **11**(2): 450-5.
1203. Marouli E, Kanoni S, Dimitriou M, Kolovou G, Deloukas P, Dedoussis G. Lifestyle may modify the glucose-raising effect of genetic loci. A study in the Greek population. Nutr Metab Cardiovasc Dis. 2016; **26**(3): 201-6.
1204. Marouli E, Kanoni S, Dimitriou M, Kolovou G, Deloukas P, Dedoussis G. Lifestyle may modify the glucose-raising effect of genetic loci. A study in the Greek population. Nutrition, Metabolism, And Cardiovascular Diseases: NMCD. 2016; **26**(3): 201-6.
1205. Marques EA, Moreira P, Wanderley F, Pizarro AN, Leao-Rosas JP, Mota J, et al. Appendicular fat mass is positively associated with femoral neck bone mineral density in older women. Menopause. 2012; **19**(3): 311-8.
1206. Marques EA, Moreira P, Wanderley F, Pizarro AN, Leão-Rosas JP, Mota J, et al. Appendicular fat mass is positively associated with femoral neck bone mineral density in older women. Menopause (New York, NY). 2012; **19**(3): 311-8.
1207. Marshall DA, Gonzalez JM, Johnson FR, MacDonald KV, Pugh A, Douglas MP, et al. What are people willing to pay for whole-genome sequencing information, and who decides what they receive? Genet Med. 2016; **18**(12): 1295-302.
1208. Marshall DA, Gonzalez JM, Johnson FR, MacDonald KV, Pugh A, Douglas MP, et al. What are people willing to pay for whole-genome sequencing information, and who decides what they receive? Genetics In Medicine: Official Journal Of The American College Of Medical Genetics. 2016; **18**(12): 1295-302.
1209. Marti A, Moreno-Aliaga MJ, Hebebrand J, Martinez JA. Genes, lifestyles and obesity. Int J Obes Relat Metab Disord. 2004; **28 Suppl 3**: S29-36.
1210. Marti A, Moreno-Aliaga MJ, Hebebrand J, Martínez JA. Genes, lifestyles and obesity. International Journal Of Obesity And Related Metabolic Disorders: Journal Of The International Association For The Study Of Obesity. 2004; **28 Suppl 3**: S29-S36.
1211. Martin E, Morel V, Joly D, Villatte C, Delage N, Dubray C, et al. Rationale and design of a randomized double-blind clinical trial in breast cancer: dextromethorphan in chemotherapy-induced peripheral neuropathy. Contemp Clin Trials. 2015; **41**: 146-51.

1212. Martin E, Morel V, Joly D, Villatte C, Delage N, Dubray C, et al. Rationale and design of a randomized double-blind clinical trial in breast cancer: dextromethorphan in chemotherapy-induced peripheral neuropathy. Contemporary Clinical Trials. 2015; **41**: 146-51.
1213. Martin V, Allaili N, Euvrard M, Marday T, Riffaud A, Franc B, et al. Effect of agomelatine on memory deficits and hippocampal gene expression induced by chronic social defeat stress in mice. Sci Rep. 2017; **8**: 45907.
1214. Martin V, Allaïli N, Euvrard M, Marday T, Riffaud A, Franc B, et al. Effect of agomelatine on memory deficits and hippocampal gene expression induced by chronic social defeat stress in mice. Scientific Reports. 2017; **8**: 45907-.
1215. Martinez-Lapiscina EH, Clavero P, Toledo E, Estruch R, Salas-Salvado J, San Julian B, et al. Mediterranean diet improves cognition: the PREDIMED-NAVARRA randomised trial. J Neurol Neurosurg Psychiatry. 2013; **84**(12): 1318-25.
1216. Martínez-Lapiscina EH, Clavero P, Toledo E, Estruch R, Salas-Salvadó J, San Julián B, et al. Mediterranean diet improves cognition: the PREDIMED-NAVARRA randomised trial. Journal Of Neurology, Neurosurgery, And Psychiatry. 2013; **84**(12): 1318-25.
1217. Martinez-Lapiscina EH, Clavero P, Toledo E, San Julian B, Sanchez-Tainta A, Corella D, et al. Virgin olive oil supplementation and long-term cognition: the PREDIMED-NAVARRA randomized, trial. J Nutr Health Aging. 2013; **17**(6): 544-52.
1218. Martínez-Lapiscina EH, Clavero P, Toledo E, San Julián B, Sanchez-Tainta A, Corella D, et al. Virgin olive oil supplementation and long-term cognition: the PREDIMED-NAVARRA randomized, trial. The Journal Of Nutrition, Health & Aging. 2013; **17**(6): 544-52.
1219. Martinez-Levy GA, Rocha L, Lubin FD, Alonso-Vanegas MA, Nani A, Buentello-Garcia RM, et al. Increased expression of BDNF transcript with exon VI in hippocampi of patients with pharmaco-resistant temporal lobe epilepsy. Neuroscience. 2016; **314**: 12-21.
1220. Martínez-Levy GA, Rocha L, Lubin FD, Alonso-Vanegas MA, Nani A, Buentello-García RM, et al. Increased expression of BDNF transcript with exon VI in hippocampi of patients with pharmaco-resistant temporal lobe epilepsy. Neuroscience. 2016; **314**: 12-21.
1221. Martinowich K, Manji H, Lu B. New insights into BDNF function in depression and anxiety. Nat Neurosci. 2007; **10**(9): 1089-93.
1222. Martinowich K, Manji H, Lu B. New insights into BDNF function in depression and anxiety. Nature Neuroscience. 2007; **10**(9): 1089-93.
1223. Marx W, Moseley G, Berk M, Jacka F. Nutritional psychiatry: the present state of the evidence. Proc Nutr Soc. 2017; **76**(4): 427-36.
1224. Marx W, Moseley G, Berk M, Jacka F. Nutritional psychiatry: the present state of the evidence. The Proceedings Of The Nutrition Society. 2017; **76**(4): 427-36.
1225. Masuki S, Nishida K, Hashimoto S, Morikawa M, Takasugi S, Nagata M, et al. Effects of milk product intake on thigh muscle strength and NFKB gene methylation during home-based interval walking training in older women: A randomized, controlled pilot study. PLoS One. 2017; **12**(5): e0176757.
1226. Masuki S, Nishida K, Hashimoto S, Morikawa M, Takasugi S, Nagata M, et al.

Effects of milk product intake on thigh muscle strength and NFKB gene methylation during home-based interval walking training in older women: A randomized, controlled pilot study. PLoS One. 2017; **12**(5): e0176757-e.
1227. Mathias RA, Kim Y, Sung H, Yanek LR, Mantese VJ, Hererra-Galeano JE, et al. A combined genome-wide linkage and association approach to find susceptibility loci for platelet function phenotypes in European American and African American families with coronary artery disease. BMC Med Genomics. 2010; **3**: 22.
1228. Mathias RA, Kim Y, Sung H, Yanek LR, Mantese VJ, Hererra-Galeano JE, et al. A combined genome-wide linkage and association approach to find susceptibility loci for platelet function phenotypes in European American and African American families with coronary artery disease. BMC Medical Genomics. 2010; **3**: 22-.
1229. Matoba N, Yu Y, Mestan K, Pearson C, Ortiz K, Porta N, et al. Differential patterns of 27 cord blood immune biomarkers across gestational age. Pediatrics. 2009; **123**(5): 1320-8.
1230. Matoba N, Yu Y, Mestan K, Pearson C, Ortiz K, Porta N, et al. Differential patterns of 27 cord blood immune biomarkers across gestational age. Pediatrics. 2009; **123**(5): 1320-8.
1231. Matrisciano F, Bonaccorso S, Ricciardi A, Scaccianoce S, Panaccione I, Wang L, et al. Changes in BDNF serum levels in patients with major depression disorder (MDD) after 6 months treatment with sertraline, escitalopram, or venlafaxine. J Psychiatr Res. 2009; **43**(3): 247-54.
1232. Matrisciano F, Bonaccorso S, Ricciardi A, Scaccianoce S, Panaccione I, Wang L, et al. Changes in BDNF serum levels in patients with major depression disorder (MDD) after 6 months treatment with sertraline, escitalopram, or venlafaxine. Journal Of Psychiatric Research. 2009; **43**(3): 247-54.
1233. Matsui M, Takahashi Y, Takebe N, Takahashi K, Nagasawa K, Honma H, et al. Response to the dipeptidyl peptidase-4 inhibitors in Japanese patients with type 2 diabetes might be associated with a diplotype of two single nucleotide polymorphisms on the interleukin-6 promoter region under a certain level of physical activity. J Diabetes Investig. 2015; **6**(2): 173-81.
1234. Matsui M, Takahashi Y, Takebe N, Takahashi K, Nagasawa K, Honma H, et al. Response to the dipeptidyl peptidase-4 inhibitors in Japanese patients with type 2 diabetes might be associated with a diplotype of two single nucleotide polymorphisms on the interleukin-6 promoter region under a certain level of physical activity. Journal Of Diabetes Investigation. 2015; **6**(2): 173-81.
1235. Matsuo K, Wakai K, Hirose K, Ito H, Saito T, Tajima K. Alcohol dehydrogenase 2 His47Arg polymorphism influences drinking habit independently of aldehyde dehydrogenase 2 Glu487Lys polymorphism: analysis of 2,299 Japanese subjects. Cancer Epidemiol Biomarkers Prev. 2006; **15**(5): 1009-13.
1236. Matsuo K, Wakai K, Hirose K, Ito H, Saito T, Tajima K. Alcohol dehydrogenase 2 His47Arg polymorphism influences drinking habit independently of aldehyde dehydrogenase 2 Glu487Lys polymorphism: analysis of 2,299 Japanese subjects. Cancer Epidemiology, Biomarkers & Prevention: A Publication Of The American Association For Cancer Research, Cosponsored By The American Society Of Preventive Oncology. 2006; **15**(5): 1009-13.

1237. Matsuoka H, Tsurutani J, Chiba Y, Fujita Y, Terashima M, Yoshida T, et al. Selection of opioids for cancer-related pain using a biomarker: a randomized, multi-institutional, open-label trial (RELIEF study). BMC Cancer. 2017; **17**(1): 674.
1238. Matsuoka H, Tsurutani J, Chiba Y, Fujita Y, Terashima M, Yoshida T, et al. Selection of opioids for cancer-related pain using a biomarker: a randomized, multi-institutional, open-label trial (RELIEF study). BMC Cancer. 2017; **17**(1): 674-.
1239. Matsuoka H, Yoshiuchi K, Koyama A, Makimura C, Fujita Y, Tsurutani J, et al. Expectation of a Decrease in Pain Affects the Prognosis of Pain in Cancer Patients: a Prospective Cohort Study of Response to Morphine. Int J Behav Med. 2017; **24**(4): 535-41.
1240. Matsuoka H, Yoshiuchi K, Koyama A, Makimura C, Fujita Y, Tsurutani J, et al. Expectation of a Decrease in Pain Affects the Prognosis of Pain in Cancer Patients: a Prospective Cohort Study of Response to Morphine. International Journal Of Behavioral Medicine. 2017; **24**(4): 535-41.
1241. Matsushita Y, Yokoyama T, Yoshiike N, Matsumura Y, Date C, Kawahara K, et al. The Trp(64)Arg polymorphism of the beta(3)-adrenergic receptor gene is not associated with body weight or body mass index in Japanese: a longitudinal analysis. J Clin Endocrinol Metab. 2003; **88**(12): 5914-20.
1242. Matsushita Y, Yokoyama T, Yoshiike N, Matsumura Y, Date C, Kawahara K, et al. The Trp(64)Arg polymorphism of the beta(3)-adrenergic receptor gene is not associated with body weight or body mass index in Japanese: a longitudinal analysis. The Journal Of Clinical Endocrinology And Metabolism. 2003; **88**(12): 5914-20.
1243. Matthews DC, Davies M, Murray J, Williams S, Tsui WH, Li Y, et al. Physical Activity, Mediterranean Diet and Biomarkers-Assessed Risk of Alzheimer's: A Multi-Modality Brain Imaging Study. Adv J Mol Imaging. 2014; **4**(4): 43-57.
1244. Matthews DC, Davies M, Murray J, Williams S, Tsui WH, Li Y, et al. Physical Activity, Mediterranean Diet and Biomarkers-Assessed Risk of Alzheimer's: A Multi-Modality Brain Imaging Study. Advances In Molecular Imaging. 2014; **4**(4): 43-57.
1245. Maximov G, Chokoeva A, Philipov S, Cardoso J, Ivanov G, Wollina U, et al. NEVUS FLAMMEUS ASSOCIATED WITH DYSPLASTIC NEVI AND LICHEN SCLEROSUS: THE FIRST REPORT IN THE MEDICAL LITERATURE. Georgian Med News. 2016; (251): 58-64.
1246. Maximov G, Chokoeva A, Philipov S, Cardoso J, Ivanov G, Wollina U, et al. NEVUS FLAMMEUS ASSOCIATED WITH DYSPLASTIC NEVI AND LICHEN SCLEROSUS: THE FIRST REPORT IN THE MEDICAL LITERATURE. Georgian Medical News. 2016; (251): 58-64.
1247. McAdams-DeMarco MA, Law A, Tan J, Delp C, King EA, Orandi B, et al. Frailty, mycophenolate reduction, and graft loss in kidney transplant recipients. Transplantation. 2015; **99**(4): 805-10.
1248. McAdams-DeMarco MA, Law A, Tan J, Delp C, King EA, Orandi B, et al. Frailty, mycophenolate reduction, and graft loss in kidney transplant recipients. Transplantation. 2015; **99**(4): 805-10.
1249. McCaffery JM, Jablonski KA, Franks PW, Delahanty LM, Aroda V, Marrero D, et al. Replication of the Association of BDNF and MC4R Variants With Dietary Intake in the Diabetes Prevention Program. Psychosom Med. 2017; **79**(2): 224-33.

1250. McCaffery JM, Jablonski KA, Franks PW, Delahanty LM, Aroda V, Marrero D, et al. Replication of the Association of BDNF and MC4R Variants With Dietary Intake in the Diabetes Prevention Program. Psychosomatic Medicine. 2017; **79**(2): 224-33.
1251. McDermott E, de Silva P. Impaired neuronal glucose uptake in pathogenesis of schizophrenia - can GLUT 1 and GLUT 3 deficits explain imaging, post-mortem and pharmacological findings? Med Hypotheses. 2005; **65**(6): 1076-81.
1252. McDermott E, de Silva P. Impaired neuronal glucose uptake in pathogenesis of schizophrenia - can GLUT 1 and GLUT 3 deficits explain imaging, post-mortem and pharmacological findings? Medical Hypotheses. 2005; **65**(6): 1076-81.
1253. McEwen BS. Central effects of stress hormones in health and disease: Understanding the protective and damaging effects of stress and stress mediators. Eur J Pharmacol. 2008; **583**(2-3): 174-85.
1254. McEwen BS. Central effects of stress hormones in health and disease: Understanding the protective and damaging effects of stress and stress mediators. European Journal Of Pharmacology. 2008; **583**(2-3): 174-85.
1255. McEwen BS. Biomarkers for assessing population and individual health and disease related to stress and adaptation. Metabolism. 2015; **64**(3 Suppl 1): S2-S10.
1256. McEwen BS. Biomarkers for assessing population and individual health and disease related to stress and adaptation. Metabolism: Clinical And Experimental. 2015; **64**(3 Suppl 1): S2-S10.
1257. McGregor AL, D'Souza G, Kim D, Tingle MD. Varenicline improves motor and cognitive deficits and decreases depressive-like behaviour in late-stage YAC128 mice. Neuropharmacology. 2017; **116**: 233-46.
1258. McGregor AL, D'Souza G, Kim D, Tingle MD. Varenicline improves motor and cognitive deficits and decreases depressive-like behaviour in late-stage YAC128 mice. Neuropharmacology. 2017; **116**: 233-46.
1259. McGuigan FE, Macdonald HM, Bassiti A, Farmer R, Bear S, Stewart A, et al. Large-scale population-based study shows no association between common polymorphisms of the TGFB1 gene and BMD in women. J Bone Miner Res. 2007; **22**(2): 195-202.
1260. McGuigan FE, Murray L, Gallagher A, Davey-Smith G, Neville CE, Van't Hof R, et al. Genetic and environmental determinants of peak bone mass in young men and women. J Bone Miner Res. 2002; **17**(7): 1273-9.
1261. McGuigan FEA, Macdonald HM, Bassiti A, Farmer R, Bear S, Stewart A, et al. Large-scale population-based study shows no association between common polymorphisms of the TGFB1 gene and BMD in women. Journal Of Bone And Mineral Research: The Official Journal Of The American Society For Bone And Mineral Research. 2007; **22**(2): 195-202.
1262. McGuigan FEA, Murray L, Gallagher A, Davey-Smith G, Neville CE, Van't Hof R, et al. Genetic and environmental determinants of peak bone mass in young men and women. Journal Of Bone And Mineral Research: The Official Journal Of The American Society For Bone And Mineral Research. 2002; **17**(7): 1273-9.
1263. McGuire JF, Lewin AB, Storch EA. Enhancing exposure therapy for anxiety disorders, obsessive-compulsive disorder and post-traumatic stress disorder. Expert Rev Neurother. 2014; **14**(8): 893-910.

1264. McGuire JF, Lewin AB, Storch EA. Enhancing exposure therapy for anxiety disorders, obsessive-compulsive disorder and post-traumatic stress disorder. Expert Review Of Neurotherapeutics. 2014; **14**(8): 893-910.
1265. McKay MJ, Baldwin JN, Ferreira P, Simic M, Vanicek N, Hiller CE, et al. 1000 Norms Project: protocol of a cross-sectional study cataloging human variation. Physiotherapy. 2016; **102**(1): 50-6.
1266. McKay MJ, Baldwin JN, Ferreira P, Simic M, Vanicek N, Hiller CE, et al. 1000 Norms Project: protocol of a cross-sectional study cataloging human variation. Physiotherapy. 2016; **102**(1): 50-6.
1267. McRae AF, Richter MM, Lind PA. Case-control association testing of common variants from sequencing of DNA pools. PLoS One. 2013; **8**(6): e65410.
1268. McRae AF, Richter MM, Lind PA. Case-control association testing of common variants from sequencing of DNA pools. PLoS One. 2013; **8**(6): e65410-e.
1269. Mehlig K, Leander K, de Faire U, Nyberg F, Berg C, Rosengren A, et al. The association between plasma homocysteine and coronary heart disease is modified by the MTHFR 677C>T polymorphism. Heart. 2013; **99**(23): 1761-5.
1270. Mehlig K, Leander K, de Faire U, Nyberg F, Berg C, Rosengren A, et al. The association between plasma homocysteine and coronary heart disease is modified by the MTHFR 677C>T polymorphism. Heart (British Cardiac Society). 2013; **99**(23): 1761-5.
1271. Mehramiz M, Ghasemi F, Esmaily H, Tayefi M, Hassanian SM, Sadeghzade M, et al. Interaction between a variant of CDKN2A/B-gene with lifestyle factors in determining dyslipidemia and estimated cardiovascular risk: A step toward personalized nutrition. Clin Nutr. 2018; **37**(1): 254-61.
1272. Mehramiz M, Ghasemi F, Esmaily H, Tayefi M, Hassanian SM, Sadeghzade M, et al. Interaction between a variant of CDKN2A/B-gene with lifestyle factors in determining dyslipidemia and estimated cardiovascular risk: A step toward personalized nutrition. Clinical Nutrition (Edinburgh, Scotland). 2018; **37**(1): 254-61.
1273. Men S, Ming X, Wang Y, Liu R, Wei C, Li Y. Genetic transformation of two species of orchid by biolistic bombardment. Plant Cell Rep. 2003; **21**(6): 592-8.
1274. Men S, Ming X, Wang Y, Liu R, Wei C, Li Y. Genetic transformation of two species of orchid by biolistic bombardment. Plant Cell Reports. 2003; **21**(6): 592-8.
1275. Mendez-David I, Guilloux JP, Papp M, Tritschler L, Mocaer E, Gardier AM, et al. S 47445 Produces Antidepressant- and Anxiolytic-Like Effects through Neurogenesis Dependent and Independent Mechanisms. Front Pharmacol. 2017; **8**: 462.
1276. Mendez-David I, Guilloux J-P, Papp M, Tritschler L, Mocaer E, Gardier AM, et al. S 47445 Produces Antidepressant- and Anxiolytic-Like Effects through Neurogenesis Dependent and Independent Mechanisms. Frontiers In Pharmacology. 2017; **8**: 462-.
1277. Meng H, Hager K, Rivkees SA, Gruen JR. Detection of Turner syndrome using high-throughput quantitative genotyping. J Clin Endocrinol Metab. 2005; **90**(6): 3419-22.
1278. Meng H, Hager K, Rivkees SA, Gruen JR. Detection of Turner syndrome using high-throughput quantitative genotyping. The Journal Of Clinical Endocrinology And Metabolism. 2005; **90**(6): 3419-22.
1279. Mennen LI, de Maat MP, Schouten EG, Kluft C, Witteman JC, Hofman A, et al. Dietary effects on coagulation factor VII vary across genotypes of the R/Q353

polymorphism in elderly people. J Nutr. 1998; **128**(5): 870-4.
1280. Mennen LI, de Maat MP, Schouten EG, Kluft C, Witteman JC, Hofman A, et al. Dietary effects on coagulation factor VII vary across genotypes of the R/Q353 polymorphism in elderly people. The Journal Of Nutrition. 1998; **128**(5): 870-4.
1281. Merke DP, Bornstein SR, Avila NA, Chrousos GP. NIH conference. Future directions in the study and management of congenital adrenal hyperplasia due to 21-hydroxylase deficiency. Ann Intern Med. 2002; **136**(4): 320-34.
1282. Merke DP, Bornstein SR, Avila NA, Chrousos GP. NIH conference. Future directions in the study and management of congenital adrenal hyperplasia due to 21-hydroxylase deficiency. Annals Of Internal Medicine. 2002; **136**(4): 320-34.
1283. Meyerhardt JA, Niedzwiecki D, Hollis D, Saltz LB, Hu FB, Mayer RJ, et al. Association of dietary patterns with cancer recurrence and survival in patients with stage III colon cancer. JAMA. 2007; **298**(7): 754-64.
1284. Meyerhardt JA, Niedzwiecki D, Hollis D, Saltz LB, Hu FB, Mayer RJ, et al. Association of dietary patterns with cancer recurrence and survival in patients with stage III colon cancer. JAMA. 2007; **298**(7): 754-64.
1285. Meyrowitz MR, Mauro JV, Aston R, Smith DB. Prolonged succinylcholine-induced apnea caused by atypical cholinesterase: report of case. J Oral Surg. 1980; **38**(5): 387-90.
1286. Meyrowitz MR, Mauro JV, Aston R, Smith DB. Prolonged succinylcholine-induced apnea caused by atypical cholinesterase: report of case. Journal Of Oral Surgery (American Dental Association: 1965). 1980; **38**(5): 387-90.
1287. Mielenz D, Reichel M, Jia T, Quinlan EB, Stockl T, Mettang M, et al. EFhd2/Swiprosin-1 is a common genetic determinator for sensation-seeking/low anxiety and alcohol addiction. Mol Psychiatry. 2017.
1288. Mielenz D, Reichel M, Jia T, Quinlan EB, Stöckl T, Mettang M, et al. EFhd2/Swiprosin-1 is a common genetic determinator for sensation-seeking/low anxiety and alcohol addiction. Molecular Psychiatry. 2018; **23**(5): 1303-19.
1289. Miguez-Burbano MJ, Espinoza L, Vargas M, LaForest D. Mood Disorders and BDNF Relationship with Alcohol Drinking Trajectories among PLWH Receiving Care. J Alcohol Drug Depend. 2014; **2**(2): 148.
1290. Míguez-Burbano MJ, Espinoza L, Vargas M, LaForest D. Mood Disorders and BDNF Relationship with Alcohol Drinking Trajectories among PLWH Receiving Care. Journal Of Alcoholism And Drug Dependence. 2014; **2**(2): 148-.
1291. Mijderwijk H, Klimek M, van Beek S, van Schaik RH, Duivenvoorden HJ, Stolker RJ. Implication of UGT2B15 Genotype Polymorphism on Postoperative Anxiety Levels in Patients Receiving Lorazepam Premedication. Anesth Analg. 2016; **123**(5): 1109-15.
1292. Mijderwijk H, Klimek M, van Beek S, van Schaik RHN, Duivenvoorden HJ, Stolker RJ. Implication of UGT2B15 Genotype Polymorphism on Postoperative Anxiety Levels in Patients Receiving Lorazepam Premedication. Anesthesia And Analgesia. 2016; **123**(5): 1109-15.
1293. Mikuliszyn R, Kowalski W, Kowalczuk K. Impact of the lay-off length on +Gz tolerance. J Gravit Physiol. 2002; **9**(1): P333-4.
1294. Mikuliszyn R, Kowalski W, Kowalczuk K. Impact of the lay-off length on +Gz tolerance. Journal Of Gravitational Physiology: A Journal Of The International Society

For Gravitational Physiology. 2002; **9**(1): P333-P4.
1295. Milazzo L, Falvella FS, Magni C, Gervasoni C, Peri AM, Cattaneo D, et al. Seizures in patients with chronic hepatitis C treated with NS3/4A protease inhibitors: does pharmacological interaction play a role? Pharmacology. 2013; **92**(5-6): 235-7.
1296. Milazzo L, Falvella FS, Magni C, Gervasoni C, Peri AM, Cattaneo D, et al. Seizures in patients with chronic hepatitis C treated with NS3/4A protease inhibitors: does pharmacological interaction play a role? Pharmacology. 2013; **92**(5-6): 235-7.
1297. Minelli A, Magri C, Barbon A, Bonvicini C, Segala M, Congiu C, et al. Proteasome system dysregulation and treatment resistance mechanisms in major depressive disorder. Transl Psychiatry. 2015; **5**: e687.
1298. Minelli A, Magri C, Barbon A, Bonvicini C, Segala M, Congiu C, et al. Proteasome system dysregulation and treatment resistance mechanisms in major depressive disorder. Translational Psychiatry. 2015; **5**: e687-e.
1299. Miras A, Boveda MD, Leis MR, Mera A, Aldamiz-Echevarria L, Fernandez-Lorenzo JR, et al. Risk factors for developing mineral bone disease in phenylketonuric patients. Mol Genet Metab. 2013; **108**(3): 149-54.
1300. Mirás A, Bóveda MD, Leis MR, Mera A, Aldámiz-Echevarría L, Fernández-Lorenzo JR, et al. Risk factors for developing mineral bone disease in phenylketonuric patients. Molecular Genetics And Metabolism. 2013; **108**(3): 149-54.
1301. Mitchell JA, Church TS, Rankinen T, Earnest CP, Sui X, Blair SN. FTO genotype and the weight loss benefits of moderate intensity exercise. Obesity (Silver Spring). 2010; **18**(3): 641-3.
1302. Mitchell JA, Church TS, Rankinen T, Earnest CP, Sui X, Blair SN. FTO genotype and the weight loss benefits of moderate intensity exercise. Obesity (Silver Spring, Md). 2010; **18**(3): 641-3.
1303. Mitjans M, Arias B, Jimenez E, Goikolea JM, Saiz PA, Garcia-Portilla MP, et al. Exploring Genetic Variability at PI, GSK3, HPA, and Glutamatergic Pathways in Lithium Response: Association With IMPA2, INPP1, and GSK3B Genes. J Clin Psychopharmacol. 2015; **35**(5): 600-4.
1304. Mitjans M, Arias B, Jiménez E, Goikolea JM, Sáiz PA, García-Portilla MP, et al. Exploring Genetic Variability at PI, GSK3, HPA, and Glutamatergic Pathways in Lithium Response: Association With IMPA2, INPP1, and GSK3B Genes. Journal Of Clinical Psychopharmacology. 2015; **35**(5): 600-4.
1305. Mizuno S, Hamada T, Nakatani K, Kishiwada M, Usui M, Sakurai H, et al. Monitoring peripheral blood CD4+ adenosine triphosphate activity after living donor liver transplantation: impact of combination assays of immune function and CYP3A5 genotype. J Hepatobiliary Pancreat Sci. 2011; **18**(2): 226-32; discussion 32-4.
1306. Mizuno S, Hamada T, Nakatani K, Kishiwada M, Usui M, Sakurai H, et al. Monitoring peripheral blood CD4+ adenosine triphosphate activity after living donor liver transplantation: impact of combination assays of immune function and CYP3A5 genotype. Journal Of Hepato-Biliary-Pancreatic Sciences. 2011; **18**(2): 226-32.
1307. Mole SE, Williams RE. Neuronal Ceroid-Lipofuscinoses. 1993.
1308. Mole SE, Williams RE. Neuronal Ceroid-Lipofuscinoses. GeneReviews®. 1993.
1309. Molgaard C, Larnkjaer A, Cashman KD, Lamberg-Allardt C, Jakobsen J, Michaelsen KF. Does vitamin D supplementation of healthy Danish Caucasian girls

affect bone turnover and bone mineralization? Bone. 2010; **46**(2): 432-9.
1310. Mølgaard C, Larnkjaer A, Cashman KD, Lamberg-Allardt C, Jakobsen J, Michaelsen KF. Does vitamin D supplementation of healthy Danish Caucasian girls affect bone turnover and bone mineralization? Bone. 2010; **46**(2): 432-9.
1311. Mollan KR, Tierney C, Hellwege JN, Eron JJ, Hudgens MG, Gulick RM, et al. Race/Ethnicity and the Pharmacogenetics of Reported Suicidality With Efavirenz Among Clinical Trials Participants. J Infect Dis. 2017; **216**(5): 554-64.
1312. Mollan KR, Tierney C, Hellwege JN, Eron JJ, Hudgens MG, Gulick RM, et al. Race/Ethnicity and the Pharmacogenetics of Reported Suicidality With Efavirenz Among Clinical Trials Participants. The Journal Of Infectious Diseases. 2017; **216**(5): 554-64.
1313. Moonat S, Pandey SC. Stress, epigenetics, and alcoholism. Alcohol Res. 2012; **34**(4): 495-505.
1314. Moonat S, Pandey SC. Stress, epigenetics, and alcoholism. Alcohol Research: Current Reviews. 2012; **34**(4): 495-505.
1315. Moore GE, Shuldiner AR, Zmuda JM, Ferrell RE, McCole SD, Hagberg JM. Obesity gene variant and elite endurance performance. Metabolism. 2001; **50**(12): 1391-2.
1316. Moore GE, Shuldiner AR, Zmuda JM, Ferrell RE, McCole SD, Hagberg JM. Obesity gene variant and elite endurance performance. Metabolism: Clinical And Experimental. 2001; **50**(12): 1391-2.
1317. Morassut RE, Langlois C, Alyass A, Ishola AF, Yazdi FT, Mayhew AJ, et al. Rationale and design of GENEiUS: a prospective observational study on the genetic and environmental determinants of body mass index evolution in Canadian undergraduate students. BMJ Open. 2017; **7**(12): e019365.
1318. Morassut RE, Langlois C, Alyass A, Ishola AF, Yazdi FT, Mayhew AJ, et al. Rationale and design of GENEiUS: a prospective observational study on the genetic and environmental determinants of body mass index evolution in Canadian undergraduate students. BMJ Open. 2017; **7**(12): e019365-e.
1319. Morita M, Tabata S, Tajima O, Yin G, Abe H, Kono S. Genetic polymorphisms of CYP2E1 and risk of colorectal adenomas in the Self Defense Forces Health Study. Cancer Epidemiol Biomarkers Prev. 2008; **17**(7): 1800-7.
1320. Morita M, Tabata S, Tajima O, Yin G, Abe H, Kono S. Genetic polymorphisms of CYP2E1 and risk of colorectal adenomas in the Self Defense Forces Health Study. Cancer Epidemiology, Biomarkers & Prevention: A Publication Of The American Association For Cancer Research, Cosponsored By The American Society Of Preventive Oncology. 2008; **17**(7): 1800-7.
1321. Morrison FG, Ressler KJ. From the neurobiology of extinction to improved clinical treatments. Depress Anxiety. 2014; **31**(4): 279-90.
1322. Morrison FG, Ressler KJ. From the neurobiology of extinction to improved clinical treatments. Depression And Anxiety. 2014; **31**(4): 279-90.
1323. Motta AB. The role of obesity in the development of polycystic ovary syndrome. Curr Pharm Des. 2012; **18**(17): 2482-91.
1324. Motta AB. The role of obesity in the development of polycystic ovary syndrome. Current Pharmaceutical Design. 2012; **18**(17): 2482-91.
1325. Mottagui-Tabar S, Prince JA, Wahlestedt C, Zhu G, Goldman D, Heilig M. A novel

single nucleotide polymorphism of the neuropeptide Y (NPY) gene associated with alcohol dependence. Alcohol Clin Exp Res. 2005; **29**(5): 702-7.
1326. Mottagui-Tabar S, Prince JA, Wahlestedt C, Zhu G, Goldman D, Heilig M. A novel single nucleotide polymorphism of the neuropeptide Y (NPY) gene associated with alcohol dependence. Alcoholism, Clinical And Experimental Research. 2005; **29**(5): 702-7.
1327. Mouti A, Reddihough D, Marraffa C, Hazell P, Wray J, Lee K, et al. Fluoxetine for Autistic Behaviors (FAB trial): study protocol for a randomized controlled trial in children and adolescents with autism. Trials. 2014; **15**: 230.
1328. Mouti A, Reddihough D, Marraffa C, Hazell P, Wray J, Lee K, et al. Fluoxetine for Autistic Behaviors (FAB trial): study protocol for a randomized controlled trial in children and adolescents with autism. Trials. 2014; **15**: 230-.
1329. Mridha N, Chattaraj S, Chakraborty D, Anand A, Aggarwal P, Nagarajan S. Pre-sowing static magnetic field treatment for improving water and radiation use efficiency in chickpea (Cicer arietinum L.) under soil moisture stress. Bioelectromagnetics. 2016; **37**(6): 400-8.
1330. Mridha N, Chattaraj S, Chakraborty D, Anand A, Aggarwal P, Nagarajan S. Pre-sowing static magnetic field treatment for improving water and radiation use efficiency in chickpea (Cicer arietinum L.) under soil moisture stress. Bioelectromagnetics. 2016; **37**(6): 400-8.
1331. Muders K, Pilat C, Deuster V, Frech T, Krüger K, Pons-Kühnemann J, et al. Effects of Traumeel (Tr14) on Exercise-Induced Muscle Damage Response in Healthy Subjects: A Double-Blind RCT. Mediators Of Inflammation. 2016; **2016**: 1693918-.
1332. Muders K, Pilat C, Deuster V, Frech T, Kruger K, Pons-Kuhnemann J, et al. Effects of Traumeel (Tr14) on Exercise-Induced Muscle Damage Response in Healthy Subjects: A Double-Blind RCT. Mediators Inflamm. 2016; **2016**: 1693918.
1333. Mujakovic S, ter Linde JJ, de Wit NJ, van Marrewijk CJ, Fransen GA, Onland-Moret NC, et al. Serotonin receptor 3A polymorphism c.-42C > T is associated with severe dyspepsia. BMC Med Genet. 2011; **12**: 140.
1334. Mujakovic S, ter Linde JJ, de Wit NJ, van Marrewijk CJ, Fransen GA, Onland-Moret NC, et al. Serotonin receptor 3A polymorphism c.-42C > T is associated with severe dyspepsia. BMC Medical Genetics. 2011; **12**: 140-.
1335. Mulle JG, Gambello MJ, Cook EH, Rutkowski TP, Glassford M. 3q29 Recurrent Deletion. GeneReviews®. 1993.
1336. Muller P, Rehfeld K, Schmicker M, Hokelmann A, Dordevic M, Lessmann V, et al. Evolution of Neuroplasticity in Response to Physical Activity in Old Age: The Case for Dancing. Front Aging Neurosci. 2017; **9**: 56.
1337. Müller P, Rehfeld K, Schmicker M, Hökelmann A, Dordevic M, Lessmann V, et al. Evolution of Neuroplasticity in Response to Physical Activity in Old Age: The Case for Dancing. Frontiers In Aging Neuroscience. 2017; **9**: 56-.
1338. Munafo MR, Johnstone EC, Aveyard P, Marteau T. Lack of association of OPRM1 genotype and smoking cessation. Nicotine Tob Res. 2013; **15**(3): 739-44.
1339. Munafò MR, Johnstone EC, Aveyard P, Marteau T. Lack of association of OPRM1 genotype and smoking cessation. Nicotine & Tobacco Research: Official Journal Of The Society For Research On Nicotine And Tobacco. 2013; **15**(3): 739-44.

1340. Munoz X, Amiano P, Celorrio D, Dorronsoro M, Sanchez MJ, Huerta JM, et al. Association of alcohol dehydrogenase polymorphisms and life-style factors with excessive alcohol intake within the Spanish population (EPIC-Spain). Addiction. 2012; **107**(12): 2117-27.
1341. Muñoz X, Amiano P, Celorrio D, Dorronsoro M, Sánchez M-J, Huerta JM, et al. Association of alcohol dehydrogenase polymorphisms and life-style factors with excessive alcohol intake within the Spanish population (EPIC-Spain). Addiction (Abingdon, England). 2012; **107**(12): 2117-27.
1342. Murakami H, Iemitsu M, Sanada K, Gando Y, Ohmori Y, Kawakami R, et al. Associations among objectively measured physical activity, fasting plasma homocysteine concentration, and MTHFR C677T genotype. Eur J Appl Physiol. 2011; **111**(12): 2997-3005.
1343. Murakami H, Iemitsu M, Sanada K, Gando Y, Ohmori Y, Kawakami R, et al. Associations among objectively measured physical activity, fasting plasma homocysteine concentration, and MTHFR C677T genotype. European Journal Of Applied Physiology. 2011; **111**(12): 2997-3005.
1344. Murawska-Cialowicz E, Wojna J, Zuwala-Jagiello J. Crossfit training changes brain-derived neurotrophic factor and irisin levels at rest, after wingate and progressive tests, and improves aerobic capacity and body composition of young physically active men and women. J Physiol Pharmacol. 2015; **66**(6): 811-21.
1345. Murawska-Cialowicz E, Wojna J, Zuwala-Jagiello J. Crossfit training changes brain-derived neurotrophic factor and irisin levels at rest, after wingate and progressive tests, and improves aerobic capacity and body composition of young physically active men and women. Journal Of Physiology And Pharmacology: An Official Journal Of The Polish Physiological Society. 2015; **66**(6): 811-21.
1346. Murphy E, Hou L, Maher BS, Woldehawariat G, Kassem L, Akula N, et al. Race, genetic ancestry and response to antidepressant treatment for major depression. Neuropsychopharmacology. 2013; **38**(13): 2598-606.
1347. Murphy E, Hou L, Maher BS, Woldehawariat G, Kassem L, Akula N, et al. Race, genetic ancestry and response to antidepressant treatment for major depression. Neuropsychopharmacology: Official Publication Of The American College Of Neuropsychopharmacology. 2013; **38**(13): 2598-606.
1348. Murphy JL, Blakely EL, Schaefer AM, He L, Wyrick P, Haller RG, et al. Resistance training in patients with single, large-scale deletions of mitochondrial DNA. Brain. 2008; **131**(Pt 11): 2832-40.
1349. Murphy JL, Blakely EL, Schaefer AM, He L, Wyrick P, Haller RG, et al. Resistance training in patients with single, large-scale deletions of mitochondrial DNA. Brain: A Journal Of Neurology. 2008; **131**(Pt 11): 2832-40.
1350. Murtaugh MA, Ma KN, Caan BJ, Sweeney C, Wolff R, Samowitz WS, et al. Interactions of peroxisome proliferator-activated receptor {gamma} and diet in etiology of colorectal cancer. Cancer Epidemiol Biomarkers Prev. 2005; **14**(5): 1224-9.
1351. Murtaugh MA, Ma K-N, Caan BJ, Sweeney C, Wolff R, Samowitz WS, et al. Interactions of peroxisome proliferator-activated receptor {gamma} and diet in etiology of colorectal cancer. Cancer Epidemiology, Biomarkers & Prevention: A Publication Of The American Association For Cancer Research, Cosponsored By The American

Society Of Preventive Oncology. 2005; **14**(5): 1224-9.
1352. Murtaugh MA, Sweeney C, Ma KN, Potter JD, Caan BJ, Wolff RK, et al. Vitamin D receptor gene polymorphisms, dietary promotion of insulin resistance, and colon and rectal cancer. Nutr Cancer. 2006; **55**(1): 35-43.
1353. Murtaugh MA, Sweeney C, Ma K-N, Potter JD, Caan BJ, Wolff RK, et al. Vitamin D receptor gene polymorphisms, dietary promotion of insulin resistance, and colon and rectal cancer. Nutrition And Cancer. 2006; **55**(1): 35-43.
1354. Muscari A, Bianchi G, Conte C, Forti P, Magalotti D, Pandolfi P, et al. No Direct Survival Effect of Light to Moderate Alcohol Drinking in Community-Dwelling Older Adults. J Am Geriatr Soc. 2015; **63**(12): 2526-33.
1355. Muscari A, Bianchi G, Conte C, Forti P, Magalotti D, Pandolfi P, et al. No Direct Survival Effect of Light to Moderate Alcohol Drinking in Community-Dwelling Older Adults. Journal Of The American Geriatrics Society. 2015; **63**(12): 2526-33.
1356. Mustafina LJ, Naumov VA, Cieszczyk P, Popov DV, Lyubaeva EV, Kostryukova ES, et al. AGTR2 gene polymorphism is associated with muscle fibre composition, athletic status and aerobic performance. Exp Physiol. 2014; **99**(8): 1042-52.
1357. Mustafina LJ, Naumov VA, Cieszczyk P, Popov DV, Lyubaeva EV, Kostryukova ES, et al. AGTR2 gene polymorphism is associated with muscle fibre composition, athletic status and aerobic performance. Experimental Physiology. 2014; **99**(8): 1042-52.
1358. Myers AM, Siegele PB, Foss JD, Tuma RF, Ward SJ. Single and combined effects of plant-derived and synthetic cannabinoids on cognition and cannabinoid-associated withdrawal signs in mice. Br J Pharmacol. 2018.
1359. Myers AM, Siegele PB, Foss JD, Tuma RF, Ward SJ. Single and combined effects of plant-derived and synthetic cannabinoids on cognition and cannabinoid-associated withdrawal signs in mice. British Journal Of Pharmacology. 2018.
1360. Narasimhan S, Aquino TD, Hodge R, Rickels K, Lohoff FW. Association analysis between the Val66Met polymorphism in the brain-derived neurotrophic factor (BDNF) gene and treatment response to venlafaxine XR in generalized anxiety disorder. Neurosci Lett. 2011; **503**(3): 200-2.
1361. Narasimhan S, Aquino TD, Hodge R, Rickels K, Lohoff FW. Association analysis between the Val66Met polymorphism in the brain-derived neurotrophic factor (BDNF) gene and treatment response to venlafaxine XR in generalized anxiety disorder. Neuroscience Letters. 2011; **503**(3): 200-2.
1362. Naudts KH, Azevedo RT, David AS, van Heeringen K, Gibbs AA. Epistasis between 5-HTTLPR and ADRA2B polymorphisms influences attentional bias for emotional information in healthy volunteers. Int J Neuropsychopharmacol. 2012; **15**(8): 1027-36.
1363. Naudts KH, Azevedo RT, David AS, van Heeringen K, Gibbs AA. Epistasis between 5-HTTLPR and ADRA2B polymorphisms influences attentional bias for emotional information in healthy volunteers. The International Journal Of Neuropsychopharmacology. 2012; **15**(8): 1027-36.
1364. Nazir N, Jan MR, Ali A, Asif M, Idrees M, Nisar M, et al. Prevalence of hepatitis-C virus genotypes and potential transmission risks in Malakand Khyber Pakhtunkhwa, Pakistan. Virol J. 2017; **14**(1): 160.

1365. Nazir N, Jan MR, Ali A, Asif M, Idrees M, Nisar M, et al. Prevalence of hepatitis-C virus genotypes and potential transmission risks in Malakand Khyber Pakhtunkhwa, Pakistan. Virology Journal. 2017; **14**(1): 160-.
1366. Negraes PD, Cugola FR, Herai RH, Trujillo CA, Cristino AS, Chailangkarn T, et al. Modeling anorexia nervosa: transcriptional insights from human iPSC-derived neurons. Transl Psychiatry. 2017; **7**(3): e1060.
1367. Negraes PD, Cugola FR, Herai RH, Trujillo CA, Cristino AS, Chailangkarn T, et al. Modeling anorexia nervosa: transcriptional insights from human iPSC-derived neurons. Translational Psychiatry. 2017; **7**(3): e1060-e.
1368. Nehlig A. Interindividual Differences in Caffeine Metabolism and Factors Driving Caffeine Consumption. Pharmacol Rev. 2018; **70**(2): 384-411.
1369. Nehlig A. Interindividual Differences in Caffeine Metabolism and Factors Driving Caffeine Consumption. Pharmacological Reviews. 2018; **70**(2): 384-411.
1370. Neishabury M, Zamani F, Keyhani E, Azarkeivan A, Abedini SS, Eslami MS, et al. The influence of the BCL11A polymorphism on the phenotype of patients with beta thalassemia could be affected by the beta globin locus control region and/or the Xmn1-HBG2 genotypic background. Blood Cells Mol Dis. 2013; **51**(2): 80-4.
1371. Neishabury M, Zamani F, Keyhani E, Azarkeivan A, Abedini SS, Eslami MS, et al. The influence of the BCL11A polymorphism on the phenotype of patients with beta thalassemia could be affected by the beta globin locus control region and/or the Xmn1-HBG2 genotypic background. Blood Cells, Molecules & Diseases. 2013; **51**(2): 80-4.
1372. Nemeroff CB, Vale WW. The neurobiology of depression: inroads to treatment and new drug discovery. J Clin Psychiatry. 2005; **66 Suppl 7**: 5-13.
1373. Nemeroff CB, Vale WW. The neurobiology of depression: inroads to treatment and new drug discovery. The Journal Of Clinical Psychiatry. 2005; **66 Suppl 7**: 5-13.
1374. Nettleton JA, Volcik KA, Hoogeveen RC, Boerwinkle E. Carbohydrate intake modifies associations between ANGPTL4[E40K] genotype and HDL-cholesterol concentrations in White men from the Atherosclerosis Risk in Communities (ARIC) study. Atherosclerosis. 2009; **203**(1): 214-20.
1375. Nettleton JA, Volcik KA, Hoogeveen RC, Boerwinkle E. Carbohydrate intake modifies associations between ANGPTL4[E40K] genotype and HDL-cholesterol concentrations in White men from the Atherosclerosis Risk in Communities (ARIC) study. Atherosclerosis. 2009; **203**(1): 214-20.
1376. Nguyen TV, Center JR, Eisman JA. Pharmacogenetics of osteoporosis and the prospect of individualized prognosis and individualized therapy. Curr Opin Endocrinol Diabetes Obes. 2008; **15**(6): 481-8.
1377. Nguyen TV, Center JR, Eisman JA. Pharmacogenetics of osteoporosis and the prospect of individualized prognosis and individualized therapy. Current Opinion In Endocrinology, Diabetes, And Obesity. 2008; **15**(6): 481-8.
1378. Nickel JC, Covell DA, Jr., Frazier-Bowers SA, Kapila S, Huja SS, Iwasaki LR. Preface to COAST 2016 innovators' workshop on personalized and precision orthodontic therapy. Orthod Craniofac Res. 2017; **20 Suppl 1**: 5-7.
1379. Nickel JC, Covell DA, Jr., Frazier-Bowers SA, Kapila S, Huja SS, Iwasaki LR. Preface to COAST 2016 innovators' workshop on personalized and precision orthodontic therapy. Orthodontics & Craniofacial Research. 2017; **20 Suppl 1**: 5-7.

1380. Nieman DC, Williams AS, Shanely RA, Jin F, McAnulty SR, Triplett NT, et al. Quercetin's influence on exercise performance and muscle mitochondrial biogenesis. Med Sci Sports Exerc. 2010; **42**(2): 338-45.
1381. Nieman DC, Williams AS, Shanely RA, Jin F, McAnulty SR, Triplett NT, et al. Quercetin's influence on exercise performance and muscle mitochondrial biogenesis. Medicine And Science In Sports And Exercise. 2010; **42**(2): 338-45.
1382. Nobili V, Liccardo D, Bedogni G, Salvatori G, Gnani D, Bersani I, et al. Influence of dietary pattern, physical activity, and I148M PNPLA3 on steatosis severity in at-risk adolescents. Genes Nutr. 2014; **9**(3): 392.
1383. Nobili V, Liccardo D, Bedogni G, Salvatori G, Gnani D, Bersani I, et al. Influence of dietary pattern, physical activity, and I148M PNPLA3 on steatosis severity in at-risk adolescents. Genes & Nutrition. 2014; **9**(3): 392-.
1384. Nordfors L, Heimburger O, Lonnqvist F, Lindholm B, Helmrich J, Schalling M, et al. Fat tissue accumulation during peritoneal dialysis is associated with a polymorphism in uncoupling protein 2. Kidney Int. 2000; **57**(4): 1713-9.
1385. Nordfors L, Heimbürger O, Lönnqvist F, Lindholm B, Helmrich J, Schalling M, et al. Fat tissue accumulation during peritoneal dialysis is associated with a polymorphism in uncoupling protein 2. Kidney International. 2000; **57**(4): 1713-9.
1386. Oberg JA, Glade Bender JL, Cohn EG, Morris M, Ruiz J, Chung WK, et al. Overcoming challenges to meaningful informed consent for whole genome sequencing in pediatric cancer research. Pediatr Blood Cancer. 2015; **62**(8): 1374-80.
1387. Oberg JA, Glade Bender JL, Cohn EG, Morris M, Ruiz J, Chung WK, et al. Overcoming challenges to meaningful informed consent for whole genome sequencing in pediatric cancer research. Pediatric Blood & Cancer. 2015; **62**(8): 1374-80.
1388. Oberlander TF, Papsdorf M, Brain UM, Misri S, Ross C, Grunau RE. Prenatal effects of selective serotonin reuptake inhibitor antidepressants, serotonin transporter promoter genotype (SLC6A4), and maternal mood on child behavior at 3 years of age. Arch Pediatr Adolesc Med. 2010; **164**(5): 444-51.
1389. Oberlander TF, Papsdorf M, Brain UM, Misri S, Ross C, Grunau RE. Prenatal effects of selective serotonin reuptake inhibitor antidepressants, serotonin transporter promoter genotype (SLC6A4), and maternal mood on child behavior at 3 years of age. Archives Of Pediatrics & Adolescent Medicine. 2010; **164**(5): 444-51.
1390. Oertel W, Wichard T, Weissgerber A. Transformation of Ulva mutabilis (Chlorophyta) by vector plasmids integrating into the genome. J Phycol. 2015; **51**(5): 963-79.
1391. Oertel W, Wichard T, Weissgerber A. Transformation of Ulva mutabilis (Chlorophyta) by vector plasmids integrating into the genome. Journal Of Phycology. 2015; **51**(5): 963-79.
1392. O'Gara C, Knight J, Stapleton J, Luty J, Neale B, Nash M, et al. Association of the serotonin transporter gene, neuroticism and smoking behaviours. J Hum Genet. 2008; **53**(3): 239-46.
1393. O'Gara C, Knight J, Stapleton J, Luty J, Neale B, Nash M, et al. Association of the serotonin transporter gene, neuroticism and smoking behaviours. Journal Of Human Genetics. 2008; **53**(3): 239-46.
1394. Okhovat M, Chen IC, Dehghani Z, Zheng DJ, Ikpatt JE, Momoh H, et al. Genetic

variation in the developmental regulation of cortical avpr1a among prairie voles. Genes Brain Behav. 2018; **17**(1): 36-48.
1395. Okhovat M, Chen IC, Dehghani Z, Zheng DJ, Ikpatt JE, Momoh H, et al. Genetic variation in the developmental regulation of cortical avpr1a among prairie voles. Genes, Brain, And Behavior. 2018; **17**(1): 36-48.
1396. Okubo T, Harada S. Polymorphism of the neuropeptide Y gene: an association study with alcohol withdrawal. Alcohol Clin Exp Res. 2001; **25**(6 Suppl): 59S-62S.
1397. Okubo T, Harada S. Polymorphism of the neuropeptide Y gene: an association study with alcohol withdrawal. Alcoholism, Clinical And Experimental Research. 2001; **25**(6 Suppl): 59S-62S.
1398. Olfson M, Liu SM, Grant BF, Blanco C. Influence of comorbid mental disorders on time to seeking treatment for major depressive disorder. Med Care. 2012; **50**(3): 227-32.
1399. Olfson M, Liu S-M, Grant BF, Blanco C. Influence of comorbid mental disorders on time to seeking treatment for major depressive disorder. Medical Care. 2012; **50**(3): 227-32.
1400. Oliveira IO, Silva LP, Borges MC, Cruz OM, Tessmann JW, Motta JV, et al. Interactions between lifestyle and MTHFR polymorphisms on homocysteine concentrations in young adults belonging to the 1982 Pelotas Birth Cohort. Eur J Clin Nutr. 2017; **71**(2): 259-66.
1401. Oliveira IO, Silva LP, Borges MC, Cruz OM, Tessmann JW, Motta JVS, et al. Interactions between lifestyle and MTHFR polymorphisms on homocysteine concentrations in young adults belonging to the 1982 Pelotas Birth Cohort. European Journal Of Clinical Nutrition. 2017; **71**(2): 259-66.
1402. Olivier JD, Vinkers CH, Olivier B. The role of the serotonergic and GABA system in translational approaches in drug discovery for anxiety disorders. Front Pharmacol. 2013; **4**: 74.
1403. Olivier JDA, Vinkers CH, Olivier B. The role of the serotonergic and GABA system in translational approaches in drug discovery for anxiety disorders. Frontiers In Pharmacology. 2013; **4**: 74-.
1404. Olivo RE, Davenport CA, Diamantidis CJ, Bhavsar NA, Tyson CC, Hall R, et al. Obesity and synergistic risk factors for chronic kidney disease in African American adults: the Jackson Heart Study. Nephrol Dial Transplant. 2017.
1405. Olivo RE, Davenport CA, Diamantidis CJ, Bhavsar NA, Tyson CC, Hall R, et al. Obesity and synergistic risk factors for chronic kidney disease in African American adults: the Jackson Heart Study. Nephrology, Dialysis, Transplantation: Official Publication Of The European Dialysis And Transplant Association - European Renal Association. 2018; **33**(6): 992-1001.
1406. Olsen RK, Broner S, Sabaratnam R, Doktor TK, Andersen HS, Bruun GH, et al. The ETFDH c.158A>G variation disrupts the balanced interplay of ESE- and ESS-binding proteins thereby causing missplicing and multiple Acyl-CoA dehydrogenation deficiency. Hum Mutat. 2014; **35**(1): 86-95.
1407. Olsen RKJ, Brøner S, Sabaratnam R, Doktor TK, Andersen HS, Bruun GH, et al. The ETFDH c.158A>G variation disrupts the balanced interplay of ESE- and ESS-binding proteins thereby causing missplicing and multiple Acyl-CoA dehydrogenation deficiency. Human Mutation. 2014; **35**(1): 86-95.

1408. Olsson CA, Anney RJ, Lotfi-Miri M, Byrnes GB, Williamson R, Patton GC. Association between the COMT Val158Met polymorphism and propensity to anxiety in an Australian population-based longitudinal study of adolescent health. Psychiatr Genet. 2005; **15**(2): 109-15.
1409. Olsson CA, Anney RJL, Lotfi-Miri M, Byrnes GB, Williamson R, Patton GC. Association between the COMT Val158Met polymorphism and propensity to anxiety in an Australian population-based longitudinal study of adolescent health. Psychiatric Genetics. 2005; **15**(2): 109-15.
1410. Ordovas JM, Shen J. Gene-environment interactions and susceptibility to metabolic syndrome and other chronic diseases. J Periodontol. 2008; **79**(8 Suppl): 1508-13.
1411. Ordovas JM, Shen J. Gene-environment interactions and susceptibility to metabolic syndrome and other chronic diseases. Journal Of Periodontology. 2008; **79**(8 Suppl): 1508-13.
1412. Orysiak J, Zmijewski P, Klusiewicz A, Kaliszewski P, Malczewska-Lenczowska J, Gajewski J, et al. The association between ace gene variation and aerobic capacity in winter endurance disciplines. Biol Sport. 2013; **30**(4): 249-53.
1413. Orysiak J, Zmijewski P, Klusiewicz A, Kaliszewski P, Malczewska-Lenczowska J, Gajewski J, et al. The association between ace gene variation and aerobic capacity in winter endurance disciplines. Biology Of Sport. 2013; **30**(4): 249-53.
1414. Owen RT. Agomelatine: a novel pharmacological approach to treating depression. Drugs Today (Barc). 2009; **45**(8): 599-608.
1415. Owen RT. Agomelatine: a novel pharmacological approach to treating depression. Drugs Of Today (Barcelona, Spain: 1998). 2009; **45**(8): 599-608.
1416. Pae CU, Lee C, Paik IH. Therapeutic implication of cocaine- and amphetamine-regulated transcript (CART) in the treatment of depression. Med Hypotheses. 2007; **69**(1): 132-5.
1417. Pae C-U, Lee C, Paik I-H. Therapeutic implication of cocaine- and amphetamine-regulated transcript (CART) in the treatment of depression. Medical Hypotheses. 2007; **69**(1): 132-5.
1418. Pajer K, Andrus BM, Gardner W, Lourie A, Strange B, Campo J, et al. Discovery of blood transcriptomic markers for depression in animal models and pilot validation in subjects with early-onset major depression. Transl Psychiatry. 2012; **2**: e101.
1419. Pajer K, Andrus BM, Gardner W, Lourie A, Strange B, Campo J, et al. Discovery of blood transcriptomic markers for depression in animal models and pilot validation in subjects with early-onset major depression. Translational Psychiatry. 2012; **2**: e101-e.
1420. Palacios JM, Pazos A, Hoyer D. A short history of the 5-HT2C receptor: from the choroid plexus to depression, obesity and addiction treatment. Psychopharmacology (Berl). 2017; **234**(9-10): 1395-418.
1421. Palacios JM, Pazos A, Hoyer D. A short history of the 5-HT2C receptor: from the choroid plexus to depression, obesity and addiction treatment. Psychopharmacology. 2017; **234**(9-10): 1395-418.
1422. Palasz E, Bak A, Gasiorowska A, Niewiadomska G. The role of trophic factors and inflammatory processes in physical activity-induced neuroprotection in Parkinson's disease. Postepy Hig Med Dosw (Online). 2017; **71**(1): 713-26.

1423. Pałasz E, Bąk A, Gąsiorowska A, Niewiadomska G. The role of trophic factors and inflammatory processes in physical activity-induced neuroprotection in Parkinson's disease. Postepy Higieny I Medycyny Doswiadczalnej (Online). 2017; **71**(1): 713-26.
1424. Palazzo E, Marconi A, Truzzi F, Dallaglio K, Petrachi T, Humbert P, et al. Role of neurotrophins on dermal fibroblast survival and differentiation. J Cell Physiol. 2012; **227**(3): 1017-25.
1425. Palazzo E, Marconi A, Truzzi F, Dallaglio K, Petrachi T, Humbert P, et al. Role of neurotrophins on dermal fibroblast survival and differentiation. Journal Of Cellular Physiology. 2012; **227**(3): 1017-25.
1426. Pall ML. Microwave frequency electromagnetic fields (EMFs) produce widespread neuropsychiatric effects including depression. J Chem Neuroanat. 2016; **75**(Pt B): 43-51.
1427. Pall ML. Microwave frequency electromagnetic fields (EMFs) produce widespread neuropsychiatric effects including depression. Journal Of Chemical Neuroanatomy. 2016; **75**(Pt B): 43-51.
1428. Palmer BR, Frampton CM, Skelton L, Yandle TG, Doughty RN, Whalley GA, et al. KCNE5 polymorphism rs697829 is associated with QT interval and survival in acute coronary syndromes patients. J Cardiovasc Electrophysiol. 2012; **23**(3): 319-24.
1429. Palmer BR, Frampton CM, Skelton L, Yandle TG, Doughty RN, Whalley GA, et al. KCNE5 polymorphism rs697829 is associated with QT interval and survival in acute coronary syndromes patients. Journal Of Cardiovascular Electrophysiology. 2012; **23**(3): 319-24.
1430. Palmisano M, Pandey SC. Epigenetic mechanisms of alcoholism and stress-related disorders. Alcohol. 2017; **60**: 7-18.
1431. Palmisano M, Pandey SC. Epigenetic mechanisms of alcoholism and stress-related disorders. Alcohol (Fayetteville, NY). 2017; **60**: 7-18.
1432. Pandey SC. Anxiety and alcohol abuse disorders: a common role for CREB and its target, the neuropeptide Y gene. Trends Pharmacol Sci. 2003; **24**(9): 456-60.
1433. Pandey SC. Anxiety and alcohol abuse disorders: a common role for CREB and its target, the neuropeptide Y gene. Trends In Pharmacological Sciences. 2003; **24**(9): 456-60.
1434. Papadimas GK, Spengos K, Konstantinopoulou A, Vassilopoulou S, Vontzalidis A, Papadopoulos C, et al. Adult Pompe disease: clinical manifestations and outcome of the first Greek patients receiving enzyme replacement therapy. Clin Neurol Neurosurg. 2011; **113**(4): 303-7.
1435. Papadimas GK, Spengos K, Konstantinopoulou A, Vassilopoulou S, Vontzalidis A, Papadopoulos C, et al. Adult Pompe disease: clinical manifestations and outcome of the first Greek patients receiving enzyme replacement therapy. Clinical Neurology And Neurosurgery. 2011; **113**(4): 303-7.
1436. Papoutsakis C, Manios Y, Magkos F, Papaconstantinou E, Schulpis KH, Zampelas A, et al. Effect of the methylenetetrahydrofolate reductase (MTHFR 677C>T) polymorphism on plasma homocysteine concentrations in healthy children is influenced by consumption of folate-fortified foods. Nutrition. 2010; **26**(10): 969-74.
1437. Papoutsakis C, Manios Y, Magkos F, Papaconstantinou E, Schulpis KH, Zampelas A, et al. Effect of the methylenetetrahydrofolate reductase (MTHFR 677C>T)

polymorphism on plasma homocysteine concentrations in healthy children is influenced by consumption of folate-fortified foods. Nutrition (Burbank, Los Angeles County, Calif). 2010; **26**(10): 969-74.
1438. Pardo-Lozano R, Farre M, Yubero-Lahoz S, O'Mathuna B, Torrens M, Mustata C, et al. Clinical pharmacology of 3,4-methylenedioxymethamphetamine (MDMA, "ecstasy"): the influence of gender and genetics (CYP2D6, COMT, 5-HTT). PLoS One. 2012; **7**(10): e47599.
1439. Pardo-Lozano R, Farré M, Yubero-Lahoz S, O'Mathúna B, Torrens M, Mustata C, et al. Clinical pharmacology of 3,4-methylenedioxymethamphetamine (MDMA, "ecstasy"): the influence of gender and genetics (CYP2D6, COMT, 5-HTT). PLoS One. 2012; **7**(10): e47599-e.
1440. Pardon M-C. Role of neurotrophic factors in behavioral processes: implications for the treatment of psychiatric and neurodegenerative disorders. Vitamins And Hormones. 2010; **82**: 185-200.
1441. Pardon MC. Role of neurotrophic factors in behavioral processes: implications for the treatment of psychiatric and neurodegenerative disorders. Vitam Horm. 2010; **82**: 185-200.
1442. Park DI, Kim HG, Jung WR, Shin MK, Kim KL. Mecamylamine attenuates dexamethasone-induced anxiety-like behavior in association with brain derived neurotrophic factor upregulation in rat brains. Neuropharmacology. 2011; **61**(1-2): 276-82.
1443. Park DI, Kim HG, Jung WR, Shin MK, Kim KL. Mecamylamine attenuates dexamethasone-induced anxiety-like behavior in association with brain derived neurotrophic factor upregulation in rat brains. Neuropharmacology. 2011; **61**(1-2): 276-82.
1444. Park KA, Park YH, Suh MH, Choi-Kwon S. Lifestyle and Genetic Predictors of Stiffness Index in Community-dwelling Elderly Korean Men and Women. Asian Nurs Res (Korean Soc Nurs Sci). 2015; **9**(3): 251-8.
1445. Park K-A, Park Y-H, Suh M-H, Choi-Kwon S. Lifestyle and Genetic Predictors of Stiffness Index in Community-dwelling Elderly Korean Men and Women. Asian Nursing Research. 2015; **9**(3): 251-8.
1446. Park MH, Sanders E, Howe M, Singh M, Hallmayer J, Kim E, et al. Association of Anxiety Symptoms in Offspring of Bipolar Parents with Serotonin Transporter-Linked Polymorphic Region (5-HTTLPR) Genotype. J Child Adolesc Psychopharmacol. 2015; **25**(6): 458-66.
1447. Park M-H, Sanders E, Howe M, Singh M, Hallmayer J, Kim E, et al. Association of Anxiety Symptoms in Offspring of Bipolar Parents with Serotonin Transporter-Linked Polymorphic Region (5-HTTLPR) Genotype. Journal Of Child And Adolescent Psychopharmacology. 2015; **25**(6): 458-66.
1448. Park S, Daily JW, Zhang X, Jin HS, Lee HJ, Lee YH. Interactions with the MC4R rs17782313 variant, mental stress and energy intake and the risk of obesity in Genome Epidemiology Study. Nutr Metab (Lond). 2016; **13**: 38.
1449. Park S, Daily JW, Zhang X, Jin HS, Lee HJ, Lee YH. Interactions with the MC4R rs17782313 variant, mental stress and energy intake and the risk of obesity in Genome Epidemiology Study. Nutrition & Metabolism. 2016; **13**: 38-.

1450. Park S, Park K, Lee Y, Chang KT, Hong Y. New Prophylactic and Therapeutic Strategies for Spinal Cord Injury. J Lifestyle Med. 2013; **3**(1): 34-40.
1451. Park S, Park K, Lee Y, Chang K-T, Hong Y. New Prophylactic and Therapeutic Strategies for Spinal Cord Injury. Journal Of Lifestyle Medicine. 2013; **3**(1): 34-40.
1452. Parmentier C, Couttet P, Wolf A, Zaccharias T, Heyd B, Bachellier P, et al. Evaluation of transcriptomic signature as a valuable tool to study drug-induced cholestasis in primary human hepatocytes. Arch Toxicol. 2017; **91**(8): 2879-93.
1453. Parmentier C, Couttet P, Wolf A, Zaccharias T, Heyd B, Bachellier P, et al. Evaluation of transcriptomic signature as a valuable tool to study drug-induced cholestasis in primary human hepatocytes. Archives Of Toxicology. 2017; **91**(8): 2879-93.
1454. Parnell LD, Blokker BA, Dashti HS, Nesbeth PD, Cooper BE, Ma Y, et al. CardioGxE, a catalog of gene-environment interactions for cardiometabolic traits. BioData Min. 2014; **7**: 21.
1455. Parnell LD, Blokker BA, Dashti HS, Nesbeth P-D, Cooper BE, Ma Y, et al. CardioGxE, a catalog of gene-environment interactions for cardiometabolic traits. Biodata Mining. 2014; **7**: 21-.
1456. Pasagian-Macaulay A, Aston CE, Ferrell RE, McAllister AE, Wing RR, Kuller LH. A dietary and behavioral intervention designed to lower coronary heart disease. Risk factors are unaffected by variation at the APOE gene locus. Atherosclerosis. 1997; **132**(2): 221-7.
1457. Pasagian-Macaulay A, Aston CE, Ferrell RE, McAllister AE, Wing RR, Kuller LH. A dietary and behavioral intervention designed to lower coronary heart disease. Risk factors are unaffected by variation at the APOE gene locus. Atherosclerosis. 1997; **132**(2): 221-7.
1458. Pataky MW, Womack CJ, Saunders MJ, Goffe JL, D'Lugos AC, El-Sohemy A, et al. Caffeine and 3-km cycling performance: Effects of mouth rinsing, genotype, and time of day. Scand J Med Sci Sports. 2016; **26**(6): 613-9.
1459. Patas K, Penninx BW, Bus BA, Vogelzangs N, Molendijk ML, Elzinga BM, et al. Association between serum brain-derived neurotrophic factor and plasma interleukin-6 in major depressive disorder with melancholic features. Brain Behav Immun. 2014; **36**: 71-9.
1460. Patas K, Penninx BWJH, Bus BAA, Vogelzangs N, Molendijk ML, Elzinga BM, et al. Association between serum brain-derived neurotrophic factor and plasma interleukin-6 in major depressive disorder with melancholic features. Brain, Behavior, And Immunity. 2014; **36**: 71-9.
1461. Patel DR, Greydanus DE. Genes and athletes. Adolesc Med. 2002; **13**(2): 249-55, v.
1462. Patel DR, Greydanus DE. Genes and athletes. Adolescent Medicine (Philadelphia, Pa). 2002; **13**(2): 249.
1463. Paterson AH, Saranga Y, Menz M, Jiang CX, Wright RJ. QTL analysis of genotype x environment interactions affecting cotton fiber quality. Theor Appl Genet. 2003; **106**(3): 384-96.
1464. Paterson AH, Saranga Y, Menz M, Jiang CX, Wright RJ. QTL analysis of genotype x environment interactions affecting cotton fiber quality. TAG Theoretical And

Applied Genetics Theoretische Und Angewandte Genetik. 2003; **106**(3): 384-96.
1465. Patterson C, Feightner JW, Garcia A, Hsiung GY, MacKnight C, Sadovnick AD. Diagnosis and treatment of dementia: 1. Risk assessment and primary prevention of Alzheimer disease. CMAJ. 2008; **178**(5): 548-56.
1466. Patterson C, Feightner JW, Garcia A, Hsiung GYR, MacKnight C, Sadovnick AD. Diagnosis and treatment of dementia: 1. Risk assessment and primary prevention of Alzheimer disease. CMAJ: Canadian Medical Association Journal = Journal De L'association Medicale Canadienne. 2008; **178**(5): 548-56.
1467. Paula RS, Souza VC, Benedet AL, Souza ER, Toledo JO, Moraes CF, et al. Dietary fat and apolipoprotein genotypes modulate plasma lipoprotein levels in Brazilian elderly women. Mol Cell Biochem. 2010; **337**(1-2): 307-15.
1468. Paula RS, Souza VC, Benedet AL, Souza ER, Toledo JO, Moraes CF, et al. Dietary fat and apolipoprotein genotypes modulate plasma lipoprotein levels in Brazilian elderly women. Molecular And Cellular Biochemistry. 2010; **337**(1-2): 307-15.
1469. Paunescu AC, Dewailly E, Dodin S, Nieboer E, Ayotte P. Dioxin-like compounds and bone quality in Cree women of Eastern James Bay (Canada): a cross-sectional study. Environ Health. 2013; **12**(1): 54.
1470. Paunescu A-C, Dewailly E, Dodin S, Nieboer E, Ayotte P. Dioxin-like compounds and bone quality in Cree women of Eastern James Bay (Canada): a cross-sectional study. Environmental Health: A Global Access Science Source. 2013; **12**(1): 54-.
1471. Pedraz M, Martin-Velasco AI, Garcia-Marchena N, Araos P, Serrano A, Romero-Sanchiz P, et al. Plasma concentrations of BDNF and IGF-1 in abstinent cocaine users with high prevalence of substance use disorders: relationship to psychiatric comorbidity. PLoS One. 2015; **10**(3): e0118610.
1472. Pedraz M, Martín-Velasco AI, García-Marchena N, Araos P, Serrano A, Romero-Sanchiz P, et al. Plasma concentrations of BDNF and IGF-1 in abstinent cocaine users with high prevalence of substance use disorders: relationship to psychiatric comorbidity. PLoS One. 2015; **10**(3): e0118610-e.
1473. Pekmezi DW, Demark-Wahnefried W. Updated evidence in support of diet and exercise interventions in cancer survivors. Acta Oncol. 2011; **50**(2): 167-78.
1474. Pekmezi DW, Demark-Wahnefried W. Updated evidence in support of diet and exercise interventions in cancer survivors. Acta Oncologica (Stockholm, Sweden). 2011; **50**(2): 167-78.
1475. Pena-Miller R, Laehnemann D, Jansen G, Fuentes-Hernandez A, Rosenstiel P, Schulenburg H, et al. When the most potent combination of antibiotics selects for the greatest bacterial load: the smile-frown transition. PLoS Biol. 2013; **11**(4): e1001540.
1476. Pena-Miller R, Laehnemann D, Jansen G, Fuentes-Hernandez A, Rosenstiel P, Schulenburg H, et al. When the most potent combination of antibiotics selects for the greatest bacterial load: the smile-frown transition. Plos Biology. 2013; **11**(4): e1001540-e.
1477. Pence BW, Miller WC, Gaynes BN, Eron JJ, Jr. Psychiatric illness and virologic response in patients initiating highly active antiretroviral therapy. J Acquir Immune Defic Syndr. 2007; **44**(2): 159-66.
1478. Pence BW, Miller WC, Gaynes BN, Eron JJ, Jr. Psychiatric illness and virologic response in patients initiating highly active antiretroviral therapy. Journal Of Acquired

Immune Deficiency Syndromes (1999). 2007; **44**(2): 159-66.
1479. Pereira DS, Queiroz BZ, Mateo EC, Assumpcao AM, Felicio DC, Miranda AS, et al. Interaction between cytokine gene polymorphisms and the effect of physical exercise on clinical and inflammatory parameters in older women: study protocol for a randomized controlled trial. Trials. 2012; **13**: 134.
1480. Pereira DS, Queiroz BZ, Mateo ECC, Assumpção AM, Felício DC, Miranda AS, et al. Interaction between cytokine gene polymorphisms and the effect of physical exercise on clinical and inflammatory parameters in older women: study protocol for a randomized controlled trial. Trials. 2012; **13**: 134-.
1481. Perez-Schindler J, Philp A. Regulation of skeletal muscle mitochondrial function by nuclear receptors: implications for health and disease. Clin Sci (Lond). 2015; **129**(7): 589-99.
1482. Perez-Schindler J, Philp A. Regulation of skeletal muscle mitochondrial function by nuclear receptors: implications for health and disease. Clinical Science (London, England: 1979). 2015; **129**(7): 589-99.
1483. Perlis RH, Huang J, Purcell S, Fava M, Rush AJ, Sullivan PF, et al. Genome-wide association study of suicide attempts in mood disorder patients. Am J Psychiatry. 2010; **167**(12): 1499-507.
1484. Perlis RH, Huang J, Purcell S, Fava M, Rush AJ, Sullivan PF, et al. Genome-wide association study of suicide attempts in mood disorder patients. The American Journal Of Psychiatry. 2010; **167**(12): 1499-507.
1485. Perna G, Favaron E, Di Bella D, Bussi R, Bellodi L. Antipanic efficacy of paroxetine and polymorphism within the promoter of the serotonin transporter gene. Neuropsychopharmacology: Official Publication Of The American College Of Neuropsychopharmacology. 2005; **30**(12): 2230-5.
1486. Perusse L, Tremblay A, Leblanc C, Bouchard C. Genetic and environmental influences on level of habitual physical activity and exercise participation. Am J Epidemiol. 1989; **129**(5): 1012-22.
1487. Pérusse L, Tremblay A, Leblanc C, Bouchard C. Genetic and environmental influences on level of habitual physical activity and exercise participation. American Journal Of Epidemiology. 1989; **129**(5): 1012-22.
1488. Peterson K, Dieperink E, Anderson J, Boundy E, Ferguson L, Helfand M. Rapid evidence review of the comparative effectiveness, harms, and cost-effectiveness of pharmacogenomics-guided antidepressant treatment versus usual care for major depressive disorder. Psychopharmacology (Berl). 2017; **234**(11): 1649-61.
1489. Peterson K, Dieperink E, Anderson J, Boundy E, Ferguson L, Helfand M. Rapid evidence review of the comparative effectiveness, harms, and cost-effectiveness of pharmacogenomics-guided antidepressant treatment versus usual care for major depressive disorder. Psychopharmacology. 2017; **234**(11): 1649-61.
1490. Petkeviciene J, Smalinskiene A, Klumbiene J, Petkevicius V, Kriaucioniene V, Lesauskaite V. Physical activity, but not dietary intake, attenuates the effect of the FTO rs9939609 polymorphism on obesity and metabolic syndrome in Lithuanian adult population. Public Health. 2016; **135**: 23-9.
1491. Petkeviciene J, Smalinskiene A, Klumbiene J, Petkevicius V, Kriaucioniene V, Lesauskaite V. Physical activity, but not dietary intake, attenuates the effect of the FTO

rs9939609 polymorphism on obesity and metabolic syndrome in Lithuanian adult population. Public Health. 2016; **135**: 23-9.
1492. Petrovski S, Shashi V, Petrou S, Schoch K, McSweeney KM, Dhindsa RS, et al. Exome sequencing results in successful riboflavin treatment of a rapidly progressive neurological condition. Cold Spring Harb Mol Case Stud. 2015; **1**(1): a000257.
1493. Petrovski S, Shashi V, Petrou S, Schoch K, McSweeney KM, Dhindsa RS, et al. Exome sequencing results in successful riboflavin treatment of a rapidly progressive neurological condition. Cold Spring Harbor Molecular Case Studies. 2015; **1**(1): a000257-a.
1494. Pezet S, Malcangio M. Brain-derived neurotrophic factor as a drug target for CNS disorders. Expert Opin Ther Targets. 2004; **8**(5): 391-9.
1495. Pezet S, Malcangio M. Brain-derived neurotrophic factor as a drug target for CNS disorders. Expert Opinion On Therapeutic Targets. 2004; **8**(5): 391-9.
1496. Pfau ML, Purushothaman I, Feng J, Golden SA, Aleyasin H, Lorsch ZS, et al. Integrative Analysis of Sex-Specific microRNA Networks Following Stress in Mouse Nucleus Accumbens. Front Mol Neurosci. 2016; **9**: 144.
1497. Pfau ML, Purushothaman I, Feng J, Golden SA, Aleyasin H, Lorsch ZS, et al. Integrative Analysis of Sex-Specific microRNA Networks Following Stress in Mouse Nucleus Accumbens. Frontiers In Molecular Neuroscience. 2016; **9**: 144-.
1498. Phillips CM, Kesse-Guyot E, McManus R, Hercberg S, Lairon D, Planells R, et al. High dietary saturated fat intake accentuates obesity risk associated with the fat mass and obesity-associated gene in adults. J Nutr. 2012; **142**(5): 824-31.
1499. Phillips CM, Kesse-Guyot E, McManus R, Hercberg S, Lairon D, Planells R, et al. High dietary saturated fat intake accentuates obesity risk associated with the fat mass and obesity-associated gene in adults. The Journal Of Nutrition. 2012; **142**(5): 824-31.
1500. Pietras T, Witusik A, Panek M, Gałecki P, Szemraj J, Górski P. [Anxiety, depression and polymorphism of the gene encoding superoxide dismutase in patients with chronic obstructive pulmonary disease]. Polski Merkuriusz Lekarski: Organ Polskiego Towarzystwa Lekarskiego. 2010; **29**(171): 165-8.
1501. Pimenta CA, Latini FR, JM DEL, TD DAS, Felipe AV, VM DELP, et al. Study of the polymorphisms of cyclooxygenase-2 (-765G>C) and 5-lipoxygenase (1708G>A) in patients with colorectal cancer. Oncol Lett. 2014; **7**(2): 513-8.
1502. Pimenta CAM, Latini FRM, De Lima JM, Da Silva TD, Felipe AV, De Lima Pazine VM, et al. Study of the polymorphisms of cyclooxygenase-2 (-765G>C) and 5-lipoxygenase (1708G>A) in patients with colorectal cancer. Oncology Letters. 2014; **7**(2): 513-8.
1503. Pirotta S, Kidgell DJ, Daly RM. Effects of vitamin D supplementation on neuroplasticity in older adults: a double-blinded, placebo-controlled randomised trial. Osteoporos Int. 2015; **26**(1): 131-40.
1504. Pirotta S, Kidgell DJ, Daly RM. Effects of vitamin D supplementation on neuroplasticity in older adults: a double-blinded, placebo-controlled randomised trial. Osteoporosis International: A Journal Established As Result Of Cooperation Between The European Foundation For Osteoporosis And The National Osteoporosis Foundation Of The USA. 2015; **26**(1): 131-40.
1505. Plata-Salaman CR. Central nervous system mechanisms contributing to the

cachexia-anorexia syndrome. Nutrition. 2000; **16**(10): 1009-12.
1506. Plata-Salamán CR. Central nervous system mechanisms contributing to the cachexia-anorexia syndrome. Nutrition (Burbank, Los Angeles County, Calif). 2000; **16**(10): 1009-12.
1507. Plemenitas A, Kastelic M, o Porcelli S, Serretti A, Dolzan V, Kores Plesnicar B. Alcohol Dependence and Genetic Variability in the Serotonin Pathway among Currently and Formerly Alcohol-Dependent Males. Neuropsychobiology. 2015; **72**(1): 57-64.
1508. Plemenitas A, Kastelic M, o Porcelli S, Serretti A, Dolžan V, Kores Plesnicar B. Alcohol Dependence and Genetic Variability in the Serotonin Pathway among Currently and Formerly Alcohol-Dependent Males. Neuropsychobiology. 2015; **72**(1): 57-64.
1509. Pogan R, Schneider C, Reimer R, Hansman G, Uetrecht C. Norovirus-like VP1 particles exhibit isolate dependent stability profiles. J Phys Condens Matter. 2018; **30**(6): 064006.
1510. Pogan R, Schneider C, Reimer R, Hansman G, Uetrecht C. Norovirus-like VP1 particles exhibit isolate dependent stability profiles. Journal Of Physics Condensed Matter: An Institute Of Physics Journal. 2018; **30**(6): 064006-.
1511. Polanczyk G, Bigarella MP, Hutz MH, Rohde LA. Pharmacogenetic approach for a better drug treatment in children. Curr Pharm Des. 2010; **16**(22): 2462-73.
1512. Polanczyk G, Bigarella MP, Hutz MH, Rohde LA. Pharmacogenetic approach for a better drug treatment in children. Current Pharmaceutical Design. 2010; **16**(22): 2462-73.
1513. Pollock BG, Ferrell RE, Mulsant BH, Mazumdar S, Miller M, Sweet RA, et al. Allelic variation in the serotonin transporter promoter affects onset of paroxetine treatment response in late-life depression. Neuropsychopharmacology. 2000; **23**(5): 587-90.
1514. Pollock BG, Ferrell RE, Mulsant BH, Mazumdar S, Miller M, Sweet RA, et al. Allelic variation in the serotonin transporter promoter affects onset of paroxetine treatment response in late-life depression. Neuropsychopharmacology: Official Publication Of The American College Of Neuropsychopharmacology. 2000; **23**(5): 587-90.
1515. Pollock K, Dahlenburg H, Nelson H, Fink KD, Cary W, Hendrix K, et al. Human Mesenchymal Stem Cells Genetically Engineered to Overexpress Brain-derived Neurotrophic Factor Improve Outcomes in Huntington's Disease Mouse Models. Molecular Therapy: The Journal Of The American Society Of Gene Therapy. 2016; **24**(5): 965-77.
1516. Pollock K, Dahlenburg H, Nelson H, Fink KD, Cary W, Hendrix K, et al. Human Mesenchymal Stem Cells Genetically Engineered to Overexpress Brain-derived Neurotrophic Factor Improve Outcomes in Huntington's Disease Mouse Models. Molecular Therapy: The Journal Of The American Society Of Gene Therapy. 2016; **24**(5): 965-77.
1517. Pollock K, Dahlenburg H, Nelson H, Fink KD, Cary W, Hendrix K, et al. Human Mesenchymal Stem Cells Genetically Engineered to Overexpress Brain-derived Neurotrophic Factor Improve Outcomes in Huntington's Disease Mouse Models. Mol Ther. 2016; **24**(5): 965-77.
1518. Pomponio RJ, Hymes J, Pandya A, Landa B, Melone P, Javaheri R, et al.

Prenatal diagnosis of heterozygosity for biotinidase deficiency by enzymatic and molecular analyses. Prenat Diagn. 1998; **18**(2): 117-22.
1519. Pomponio RJ, Hymes J, Pandya A, Landa B, Melone P, Javaheri R, et al. Prenatal diagnosis of heterozygosity for biotinidase deficiency by enzymatic and molecular analyses. Prenatal Diagnosis. 1998; **18**(2): 117-22.
1520. Popova PV, Klyushina AA, Vasilyeva LB, Tkachuk AS, Bolotko YA, Gerasimov AS, et al. Effect of gene-lifestyle interaction on gestational diabetes risk. Oncotarget. 2017; **8**(67): 112024-35.
1521. Popova PV, Klyushina AA, Vasilyeva LB, Tkachuk AS, Bolotko YA, Gerasimov AS, et al. Effect of gene-lifestyle interaction on gestational diabetes risk. Oncotarget. 2017; **8**(67): 112024-35.
1522. Portoles O, Sorli JV, Frances F, Coltell O, Gonzalez JI, Saiz C, et al. Effect of genetic variation in the leptin gene promoter and the leptin receptor gene on obesity risk in a population-based case-control study in Spain. Eur J Epidemiol. 2006; **21**(8): 605-12.
1523. Portolés O, Sorlí JV, Francés F, Coltell O, González JI, Sáiz C, et al. Effect of genetic variation in the leptin gene promoter and the leptin receptor gene on obesity risk in a population-based case-control study in Spain. European Journal Of Epidemiology. 2006; **21**(8): 605-12.
1524. Portugal EM, Vasconcelos PG, Souza R, Lattari E, Monteiro-Junior RS, Machado S, et al. Aging process, cognitive decline and Alzheimer`s disease: can strength training modulate these responses? CNS Neurol Disord Drug Targets. 2015; **14**(9): 1209-13.
1525. Portugal EMM, Vasconcelos PGT, Souza R, Lattari E, Monteiro-Junior RS, Machado S, et al. Aging process, cognitive decline and Alzheimer`s disease: can strength training modulate these responses? CNS & Neurological Disorders Drug Targets. 2015; **14**(9): 1209-13.
1526. Post RM, Weiss SR. Sensitization and kindling phenomena in mood, anxiety, and obsessive-compulsive disorders: the role of serotonergic mechanisms in illness progression. Biol Psychiatry. 1998; **44**(3): 193-206.
1527. Post RM, Weiss SR. Sensitization and kindling phenomena in mood, anxiety, and obsessive-compulsive disorders: the role of serotonergic mechanisms in illness progression. Biological Psychiatry. 1998; **44**(3): 193-206.
1528. Poveda A, Chen Y, Brandstrom A, Engberg E, Hallmans G, Johansson I, et al. The heritable basis of gene-environment interactions in cardiometabolic traits. Diabetologia. 2017; **60**(3): 442-52.
1529. Poveda A, Chen Y, Brändström A, Engberg E, Hallmans G, Johansson I, et al. The heritable basis of gene-environment interactions in cardiometabolic traits. Diabetologia. 2017; **60**(3): 442-52.
1530. Powers MB, Medina JL, Burns S, Kauffman BY, Monfils M, Asmundson GJ, et al. Exercise Augmentation of Exposure Therapy for PTSD: Rationale and Pilot Efficacy Data. Cogn Behav Ther. 2015; **44**(4): 314-27.
1531. Powers MB, Medina JL, Burns S, Kauffman BY, Monfils M, Asmundson GJG, et al. Exercise Augmentation of Exposure Therapy for PTSD: Rationale and Pilot Efficacy Data. Cognitive Behaviour Therapy. 2015; **44**(4): 314-27.
1532. Presse F, Conductier G, Rovere C, Nahon JL. The melanin-concentrating

hormone receptors: neuronal and non-neuronal functions. Int J Obes Suppl. 2014; **4**(Suppl 1): S31-6.
1533. Presse F, Conductier G, Rovere C, Nahon JL. The melanin-concentrating hormone receptors: neuronal and non-neuronal functions. International Journal Of Obesity Supplements. 2014; **4**(Suppl 1): S31-S6.
1534. Pretet JL, Jacquard AC, Saunier M, Clavel C, Dachez R, Gondry J, et al. Human papillomavirus genotype distribution in low-grade squamous intraepithelial lesions in France and comparison with CIN2/3 and invasive cervical cancer: the EDiTH III study. Gynecol Oncol. 2008; **110**(2): 179-84.
1535. Prétet J-L, Jacquard A-C, Saunier M, Clavel C, Dachez R, Gondry J, et al. Human papillomavirus genotype distribution in low-grade squamous intraepithelial lesions in France and comparison with CIN2/3 and invasive cervical cancer: the EDiTH III study. Gynecologic Oncology. 2008; **110**(2): 179-84.
1536. Prince DA, Parada I, Graber K. Traumatic Brain Injury and Posttraumatic Epilepsy. 2012.
1537. Prince DA, Parada I, Graber K. Traumatic Brain Injury and Posttraumatic Epilepsy. Jasper's Basic Mechanisms of the Epilepsies. 2012.
1538. Puthucheary Z, Skipworth JR, Rawal J, Loosemore M, Van Someren K, Montgomery HE. The ACE gene and human performance: 12 years on. Sports Med. 2011; **41**(6): 433-48.
1539. Puthucheary Z, Skipworth JRA, Rawal J, Loosemore M, Van Someren K, Montgomery HE. The ACE gene and human performance: 12 years on. Sports Medicine (Auckland, NZ). 2011; **41**(6): 433-48.
1540. Qi M, Zhou B, Su M, Pan J, Zhang H. [Escitalopram for intervention of psychiatric adverse events during peginterferon-alfa-2a and ribavirin treatment for chronic hepatitis C]. Nan Fang Yi Ke Da Xue Xue Bao. 2013; **33**(7): 1012-6.
1541. Qi M, Zhou B, Su M, Pan J, Zhang H. [Escitalopram for intervention of psychiatric adverse events during peginterferon-alfa-2a and ribavirin treatment for chronic hepatitis C]. Nan Fang Yi Ke Da Xue Xue Bao = Journal Of Southern Medical University. 2013; **33**(7): 1012-6.
1542. Qin X, Li J, Zhang Y, Ma W, Fan F, Wang B, et al. Prevalence and associated factors of diabetes and impaired fasting glucose in Chinese hypertensive adults aged 45 to 75 years. PLoS One. 2012; **7**(8): e42538.
1543. Qin X, Li J, Zhang Y, Ma W, Fan F, Wang B, et al. Prevalence and associated factors of diabetes and impaired fasting glucose in Chinese hypertensive adults aged 45 to 75 years. PLoS One. 2012; **7**(8): e42538-e.
1544. Ragen BJ, Seidel J, Chollak C, Pietrzak RH, Neumeister A. Investigational drugs under development for the treatment of PTSD. Expert Opin Investig Drugs. 2015; **24**(5): 659-72.
1545. Ragen BJ, Seidel J, Chollak C, Pietrzak RH, Neumeister A. Investigational drugs under development for the treatment of PTSD. Expert Opinion On Investigational Drugs. 2015; **24**(5): 659-72.
1546. Rajendra Prasad B, Khadeer MA, Seeta P, Anwar SY. In vitro induction of androgenic haploids in Safflower (Carthamus tinctorius L.). Plant Cell Rep. 1991; **10**(1): 48-51.

1547. Rajendra Prasad B, Khadeer MA, Seeta P, Anwar SY. In vitro induction of androgenic haploids in Safflower (Carthamus tinctorius L.). Plant Cell Reports. 1991; **10**(1): 48-51.
1548. Ralston SH. Genetics of osteoporosis. The Proceedings Of The Nutrition Society. 2007; **66**(2): 158-65.
1549. Randall CL, McNeil DW, Shaffer JR, Crout RJ, Weyant RJ, Marazita ML. Fear of Pain Mediates the Association between MC1R Genotype and Dental Fear. J Dent Res. 2016; **95**(10): 1132-7.
1550. Randall CL, McNeil DW, Shaffer JR, Crout RJ, Weyant RJ, Marazita ML. Fear of Pain Mediates the Association between MC1R Genotype and Dental Fear. Journal Of Dental Research. 2016; **95**(10): 1132-7.
1551. Rangnekar AS, Fontana RJ. Meta-analysis: IL-28B genotype and sustained viral clearance in HCV genotype 1 patients. Aliment Pharmacol Ther. 2012; **36**(2): 104-14.
1552. Rangnekar AS, Fontana RJ. Meta-analysis: IL-28B genotype and sustained viral clearance in HCV genotype 1 patients. Alimentary Pharmacology & Therapeutics. 2012; **36**(2): 104-14.
1553. Rao YS, Mott NN, Wang Y, Chung WC, Pak TR. MicroRNAs in the aging female brain: a putative mechanism for age-specific estrogen effects. Endocrinology. 2013; **154**(8): 2795-806.
1554. Rao YS, Mott NN, Wang Y, Chung WCJ, Pak TR. MicroRNAs in the aging female brain: a putative mechanism for age-specific estrogen effects. Endocrinology. 2013; **154**(8): 2795-806.
1555. Rask-Andersen M, Karlsson T, Ek WE, Johansson A. Gene-environment interaction study for BMI reveals interactions between genetic factors and physical activity, alcohol consumption and socioeconomic status. PLoS Genet. 2017; **13**(9): e1006977.
1556. Rask-Andersen M, Karlsson T, Ek WE, Johansson Å. Gene-environment interaction study for BMI reveals interactions between genetic factors and physical activity, alcohol consumption and socioeconomic status. Plos Genetics. 2017; **13**(9): e1006977-e.
1557. Raslova K, Smolkova B, Vohnout B, Gasparovic J, Frohlich JJ. Risk factors for atherosclerosis in survivors of myocardial infarction and their spouses: comparison to controls without personal and family history of atherosclerosis. Metabolism. 2001; **50**(1): 24-9.
1558. Raslová K, Smolková B, Vohnout B, Gasparovic J, Frohlich JJ. Risk factors for atherosclerosis in survivors of myocardial infarction and their spouses: comparison to controls without personal and family history of atherosclerosis. Metabolism: Clinical And Experimental. 2001; **50**(1): 24-9.
1559. Ravaglia S, De Filippi P, Pichiecchio A, Ponzio M, Saeidi Garaghani K, Poloni GU, et al. Can genes influencing muscle function affect the therapeutic response to enzyme replacement therapy (ERT) in late-onset type II glycogenosis? Mol Genet Metab. 2012; **107**(1-2): 104-10.
1560. Ravaglia S, De Filippi P, Pichiecchio A, Ponzio M, Saeidi Garaghani K, Poloni GU, et al. Can genes influencing muscle function affect the therapeutic response to enzyme replacement therapy (ERT) in late-onset type II glycogenosis? Molecular

Genetics And Metabolism. 2012; **107**(1-2): 104-10.
1561. Rea IM. Towards ageing well: Use it or lose it: Exercise, epigenetics and cognition. Biogerontology. 2017; **18**(4): 679-91.
1562. Rea IM. Towards ageing well: Use it or lose it: Exercise, epigenetics and cognition. Biogerontology. 2017; **18**(4): 679-91.
1563. Real JT, Merchante A, Gomez JL, Chaves FJ, Ascaso JF, Carmena R. Effects of marathon running on plasma total homocysteine concentrations. Nutr Metab Cardiovasc Dis. 2005; **15**(2): 134-9.
1564. Real JT, Merchante A, Gómez JL, Chaves FJ, Ascaso JF, Carmena R. Effects of marathon running on plasma total homocysteine concentrations. Nutrition, Metabolism, And Cardiovascular Diseases: NMCD. 2005; **15**(2): 134-9.
1565. Reddon H, Pettes T, Wood E, Nosova E, Milloy MJ, Kerr T, et al. Incidence and predictors of mental health disorder diagnoses among people who inject drugs in a Canadian setting. Drug Alcohol Rev. 2017.
1566. Reddon H, Pettes T, Wood E, Nosova E, Milloy M-J, Kerr T, et al. Incidence and predictors of mental health disorder diagnoses among people who inject drugs in a Canadian setting. Drug And Alcohol Review. 2018; **37 Suppl 1**: S285-S93.
1567. Redei EE, Mehta NS. Blood transcriptomic markers for major depression: from animal models to clinical settings. Ann N Y Acad Sci. 2015; **1344**: 37-49.
1568. Redei EE, Mehta NS. Blood transcriptomic markers for major depression: from animal models to clinical settings. Annals Of The New York Academy Of Sciences. 2015; **1344**: 37-49.
1569. Reijs BLR, Vos SJB, Soininen H, Lotjonen J, Koikkalainen J, Pikkarainen M, et al. Association Between Later Life Lifestyle Factors and Alzheimer's Disease Biomarkers in Non-Demented Individuals: A Longitudinal Descriptive Cohort Study. J Alzheimers Dis. 2017; **60**(4): 1387-95.
1570. Reijs BLR, Vos SJB, Soininen H, Lötjonen J, Koikkalainen J, Pikkarainen M, et al. Association Between Later Life Lifestyle Factors and Alzheimer's Disease Biomarkers in Non-Demented Individuals: A Longitudinal Descriptive Cohort Study. Journal Of Alzheimer's Disease: JAD. 2017; **60**(4): 1387-95.
1571. Reist C, Mazzanti C, Vu R, Fujimoto K, Goldman D. Inter-relationships of intermediate phenotypes for serotonin function, impulsivity, and a 5-HT2A candidate allele: His452Tyr. Mol Psychiatry. 2004; **9**(9): 871-8.
1572. Reist C, Mazzanti C, Vu R, Fujimoto K, Goldman D. Inter-relationships of intermediate phenotypes for serotonin function, impulsivity, and a 5-HT2A candidate allele: His452Tyr. Molecular Psychiatry. 2004; **9**(9): 871-8.
1573. Repici A, Genco C, Anderloni A, Spaggiari P, Mineri R, Carlino A, et al. A case of esophageal squamous cell intraepithelial neoplasia with positivity for type 16 human papillomavirus successfully treated with radiofrequency ablation. J Gastrointest Oncol. 2014; **5**(2): E36-9.
1574. Repici A, Genco C, Anderloni A, Spaggiari P, Mineri R, Carlino A, et al. A case of esophageal squamous cell intraepithelial neoplasia with positivity for type 16 human papillomavirus successfully treated with radiofrequency ablation. Journal Of Gastrointestinal Oncology. 2014; **5**(2): E36-E9.
1575. Reuscher S, Akiyama M, Yasuda T, Makino H, Aoki K, Shibata D, et al. The sugar

transporter inventory of tomato: genome-wide identification and expression analysis. Plant Cell Physiol. 2014; **55**(6): 1123-41.
1576. Reuscher S, Akiyama M, Yasuda T, Makino H, Aoki K, Shibata D, et al. The sugar transporter inventory of tomato: genome-wide identification and expression analysis. Plant & Cell Physiology. 2014; **55**(6): 1123-41.
1577. Rice TK, Sarzynski MA, Sung YJ, Argyropoulos G, Stutz AM, Teran-Garcia M, et al. Fine mapping of a QTL on chromosome 13 for submaximal exercise capacity training response: the HERITAGE Family Study. Eur J Appl Physiol. 2012; **112**(8): 2969-78.
1578. Rice TK, Sarzynski MA, Sung YJ, Argyropoulos G, Stütz AM, Teran-Garcia M, et al. Fine mapping of a QTL on chromosome 13 for submaximal exercise capacity training response: the HERITAGE Family Study. European Journal Of Applied Physiology. 2012; **112**(8): 2969-78.
1579. Richards EM, Mathews DC, Luckenbaugh DA, Ionescu DF, Machado-Vieira R, Niciu MJ, et al. A randomized, placebo-controlled pilot trial of the delta opioid receptor agonist AZD2327 in anxious depression. Psychopharmacology (Berl). 2016; **233**(6): 1119-30.
1580. Richards EM, Mathews DC, Luckenbaugh DA, Ionescu DF, Machado-Vieira R, Niciu MJ, et al. A randomized, placebo-controlled pilot trial of the delta opioid receptor agonist AZD2327 in anxious depression. Psychopharmacology. 2016; **233**(6): 1119-30.
1581. Ricketts MH, Hamer RM, Sage JI, Manowitz P, Feng F, Menza MA. Association of a serotonin transporter gene promoter polymorphism with harm avoidance behaviour in an elderly population. Psychiatr Genet. 1998; **8**(2): 41-4.
1582. Ricketts MH, Hamer RM, Sage JI, Manowitz P, Feng F, Menza MA. Association of a serotonin transporter gene promoter polymorphism with harm avoidance behaviour in an elderly population. Psychiatric Genetics. 1998; **8**(2): 41-4.
1583. Rivkin A, Alexander RC, Knighton J, Hutson PH, Wang XJ, Snavely DB, et al. A randomized, double-blind, crossover comparison of MK-0929 and placebo in the treatment of adults with ADHD. J Atten Disord. 2012; **16**(8): 664-74.
1584. Rivkin A, Alexander RC, Knighton J, Hutson PH, Wang XJ, Snavely DB, et al. A randomized, double-blind, crossover comparison of MK-0929 and placebo in the treatment of adults with ADHD. Journal Of Attention Disorders. 2012; **16**(8): 664-74.
1585. Roberts S, Keers R, Lester KJ, Coleman JR, Breen G, Arendt K, et al. HPA AXIS RELATED GENES AND RESPONSE TO PSYCHOLOGICAL THERAPIES: GENETICS AND EPIGENETICS. Depress Anxiety. 2015; **32**(12): 861-70.
1586. Roberts S, Keers R, Lester KJ, Coleman JRI, Breen G, Arendt K, et al. HPA AXIS RELATED GENES AND RESPONSE TO PSYCHOLOGICAL THERAPIES: GENETICS AND EPIGENETICS. Depression And Anxiety. 2015; **32**(12): 861-70.
1587. Roberts S, Wong CCY, Breen G, Coleman JRI, De Jong S, Johren P, et al. Genome-wide expression and response to exposure-based psychological therapy for anxiety disorders. Transl Psychiatry. 2017; **7**(8): e1219.
1588. Roberts S, Wong CCY, Breen G, Coleman JRI, De Jong S, Jöhren P, et al. Genome-wide expression and response to exposure-based psychological therapy for anxiety disorders. Translational Psychiatry. 2017; **7**(8): e1219-e.
1589. Robinson-Hamm JN, Gersbach CA. Gene therapies that restore dystrophin expression for the treatment of Duchenne muscular dystrophy. Hum Genet. 2016;

135(9): 1029-40.
1590. Robinson-Hamm JN, Gersbach CA. Gene therapies that restore dystrophin expression for the treatment of Duchenne muscular dystrophy. Human Genetics. 2016; **135**(9): 1029-40.
1591. Rodrigues AC, Sobrino B, Genvigir FD, Willrich MA, Arazi SS, Dorea EL, et al. Genetic variants in genes related to lipid metabolism and atherosclerosis, dyslipidemia and atorvastatin response. Clin Chim Acta. 2013; **417**: 8-11.
1592. Rodrigues AC, Sobrino B, Genvigir FDV, Willrich MAV, Arazi SS, Dorea EL, et al. Genetic variants in genes related to lipid metabolism and atherosclerosis, dyslipidemia and atorvastatin response. Clinica Chimica Acta; International Journal Of Clinical Chemistry. 2013; **417**: 8-11.
1593. Rodrigues DM, Reis RS, Dalle Molle R, Machado TD, Mucellini AB, Bortoluzzi A, et al. Decreased comfort food intake and allostatic load in adolescents carrying the A3669G variant of the glucocorticoid receptor gene. Appetite. 2017; **116**: 21-8.
1594. Rodrigues DM, Reis RS, Dalle Molle R, Machado TD, Mucellini AB, Bortoluzzi A, et al. Decreased comfort food intake and allostatic load in adolescents carrying the A3669G variant of the glucocorticoid receptor gene. Appetite. 2017; **116**: 21-8.
1595. Rogers J, Renoir T, Hannan AJ. Gene-environment interactions informing therapeutic approaches to cognitive and affective disorders. Neuropharmacology. 2017.
1596. Rogers PJ, Hohoff C, Heatherley SV, Mullings EL, Maxfield PJ, Evershed RP, et al. Association of the anxiogenic and alerting effects of caffeine with ADORA2A and ADORA1 polymorphisms and habitual level of caffeine consumption. Neuropsychopharmacology. 2010; **35**(9): 1973-83.
1597. Rogers PJ, Hohoff C, Heatherley SV, Mullings EL, Maxfield PJ, Evershed RP, et al. Association of the anxiogenic and alerting effects of caffeine with ADORA2A and ADORA1 polymorphisms and habitual level of caffeine consumption. Neuropsychopharmacology: Official Publication Of The American College Of Neuropsychopharmacology. 2010; **35**(9): 1973-83.
1598. Rogowska-Kalisz A, Tkaczyk M, Szalapska-Zawodniak M. [Diabetes mellitus as a rare complication of hemolytic uremic syndrome--case report]. Pol Merkur Lekarski. 2010; **28**(163): 46-9.
1599. Rogowska-Kalisz A, Tkaczyk M, Szałapska-Zawodniak M. [Diabetes mellitus as a rare complication of hemolytic uremic syndrome--case report]. Polski Merkuriusz Lekarski: Organ Polskiego Towarzystwa Lekarskiego. 2010; **28**(163): 46-9.
1600. Roh HT, So WY. Cranial electrotherapy stimulation affects mood state but not levels of peripheral neurotrophic factors or hypothalamic- pituitary-adrenal axis regulation. Technol Health Care. 2017; **25**(3): 403-12.
1601. Roh H-T, So W-Y. Cranial electrotherapy stimulation affects mood state but not levels of peripheral neurotrophic factors or hypothalamic- pituitary-adrenal axis regulation. Technology And Health Care: Official Journal Of The European Society For Engineering And Medicine. 2017; **25**(3): 403-12.
1602. Rollison DE, Cole AL, Tung KH, Slattery ML, Baumgartner KB, Byers T, et al. Vitamin D intake, vitamin D receptor polymorphisms, and breast cancer risk among women living in the southwestern U.S. Breast Cancer Res Treat. 2012; **132**(2): 683-91.
1603. Rollison DE, Cole AL, Tung K-H, Slattery ML, Baumgartner KB, Byers T, et al.

Vitamin D intake, vitamin D receptor polymorphisms, and breast cancer risk among women living in the southwestern U.S. Breast Cancer Research And Treatment. 2012; **132**(2): 683-91.
1604. Roltsch MH, Brown MD, Hand BD, Kostek MC, Phares DA, Huberty A, et al. No association between ACE I/D polymorphism and cardiovascular hemodynamics during exercise in young women. Int J Sports Med. 2005; **26**(8): 638-44.
1605. Roltsch MH, Brown MD, Hand BD, Kostek MC, Phares DA, Huberty A, et al. No association between ACE I/D polymorphism and cardiovascular hemodynamics during exercise in young women. International Journal Of Sports Medicine. 2005; **26**(8): 638-44.
1606. Romeo B, Choucha W, Fossati P, Rotge JY. [Clinical and biological predictors of ketamine response in treatment-resistant major depression: Review]. Encephale. 2017; **43**(4): 354-62.
1607. Romeo B, Choucha W, Fossati P, Rotge JY. [Clinical and biological predictors of ketamine response in treatment-resistant major depression: Review]. L'encephale. 2017; **43**(4): 354-62.
1608. Romijn AR, Rucklidge JJ, Kuijer RG, Frampton C. A double-blind, randomized, placebo-controlled trial of Lactobacillus helveticus and Bifidobacterium longum for the symptoms of depression. Aust N Z J Psychiatry. 2017; **51**(8): 810-21.
1609. Romijn AR, Rucklidge JJ, Kuijer RG, Frampton C. A double-blind, randomized, placebo-controlled trial of Lactobacillus helveticus and Bifidobacterium longum for the symptoms of depression. The Australian And New Zealand Journal Of Psychiatry. 2017; **51**(8): 810-21.
1610. Roncari DA. Individual variations in energy utilized for biomechanical processes and molecular mobility account for diverse susceptibility to obesity. Med Hypotheses. 1987; **23**(1): 11-8.
1611. Roncari DA. Individual variations in energy utilized for biomechanical processes and molecular mobility account for diverse susceptibility to obesity. Medical Hypotheses. 1987; **23**(1): 11-8.
1612. Rosado EL, Bressan J, Martinez JA. Environmental factors and beta2-adrenergic receptor polymorphism: influence on the energy expenditure and nutritional status of obese women. Lipids. 2015; **50**(5): 459-67.
1613. Rosado EL, Bressan J, Martínez JA. Environmental factors and beta2-adrenergic receptor polymorphism: influence on the energy expenditure and nutritional status of obese women. Lipids. 2015; **50**(5): 459-67.
1614. Rosenbloom AL. Increasing incidence of type 2 diabetes in children and adolescents: treatment considerations. Paediatr Drugs. 2002; **4**(4): 209-21.
1615. Rosenbloom AL. Increasing incidence of type 2 diabetes in children and adolescents: treatment considerations. Paediatric Drugs. 2002; **4**(4): 209-21.
1616. Roth SM, Rankinen T, Hagberg JM, Loos RJ, Perusse L, Sarzynski MA, et al. Advances in exercise, fitness, and performance genomics in 2011. Med Sci Sports Exerc. 2012; **44**(5): 809-17.
1617. Roth SM, Rankinen T, Hagberg JM, Loos RJF, Pérusse L, Sarzynski MA, et al. Advances in exercise, fitness, and performance genomics in 2011. Medicine And Science In Sports And Exercise. 2012; **44**(5): 809-17.

1618. Roth SM, Zmuda JM, Cauley JA, Shea PR, Ferrell RE. Vitamin D receptor genotype is associated with fat-free mass and sarcopenia in elderly men. J Gerontol A Biol Sci Med Sci. 2004; **59**(1): 10-5.
1619. Roth SM, Zmuda JM, Cauley JA, Shea PR, Ferrell RE. Vitamin D receptor genotype is associated with fat-free mass and sarcopenia in elderly men. The Journals Of Gerontology Series A, Biological Sciences And Medical Sciences. 2004; **59**(1): 10-5.
1620. Rouleau-Mailloux E, Shahabi P, Dumas S, Feroz Zada Y, Provost S, Hu J, et al. Impact of regular physical activity on weekly warfarin dose requirement. J Thromb Thrombolysis. 2016; **41**(2): 328-35.
1621. Rouleau-Mailloux É, Shahabi P, Dumas S, Feroz Zada Y, Provost S, Hu J, et al. Impact of regular physical activity on weekly warfarin dose requirement. Journal Of Thrombosis And Thrombolysis. 2016; **41**(2): 328-35.
1622. Rubin LA, Hawker GA, Peltekova VD, Fielding LJ, Ridout R, Cole DE. Determinants of peak bone mass: clinical and genetic analyses in a young female Canadian cohort. J Bone Miner Res. 1999; **14**(4): 633-43.
1623. Rubin LA, Hawker GA, Peltekova VD, Fielding LJ, Ridout R, Cole DE. Determinants of peak bone mass: clinical and genetic analyses in a young female Canadian cohort. Journal Of Bone And Mineral Research: The Official Journal Of The American Society For Bone And Mineral Research. 1999; **14**(4): 633-43.
1624. Ruby CL, Walker DL, An J, Kim J, Choi D-S. Sex-Specific Regulation of Depression, Anxiety-Like Behaviors and Alcohol Drinking in Mice Lacking ENT1. Journal Of Addiction Research & Therapy. 2011; **S4**.
1625. Ruby CL, Walker DL, An J, Kim J, Choi DS. Sex-Specific Regulation of Depression, Anxiety-Like Behaviors and Alcohol Drinking in Mice Lacking ENT1. J Addict Res Ther. 2011; **S4**.
1626. Ruiz C, Cid VJ, Lussier M, Molina M, Nombela C. A large-scale sonication assay for cell wall mutant analysis in yeast. Yeast. 1999; **15**(10B): 1001-8.
1627. Ruiz C, Cid VJ, Lussier M, Molina M, Nombela C. A large-scale sonication assay for cell wall mutant analysis in yeast. Yeast (Chichester, England). 1999; **15**(10B): 1001-8.
1628. Rundell JR, Staab JP, Shinozaki G, McAlpine D. Serotonin transporter gene promotor polymorphism (5-HTTLPR) associations with number of psychotropic medication trials in a tertiary care outpatient psychiatric consultation practice. Psychosomatics. 2011; **52**(2): 147-53.
1629. Rundell JR, Staab JP, Shinozaki G, McAlpine D. Serotonin transporter gene promotor polymorphism (5-HTTLPR) associations with number of psychotropic medication trials in a tertiary care outpatient psychiatric consultation practice. Psychosomatics. 2011; **52**(2): 147-53.
1630. Rustad TR, Stevens DA, Pfaller MA, White TC. Homozygosity at the Candida albicans MTL locus associated with azole resistance. Microbiology. 2002; **148**(Pt 4): 1061-72.
1631. Rustad TR, Stevens DA, Pfaller MA, White TC. Homozygosity at the Candida albicans MTL locus associated with azole resistance. Microbiology (Reading, England). 2002; **148**(Pt 4): 1061-72.
1632. Rutters F, Lemmens SG, Born JM, Bouwman F, Nieuwenhuizen AG, Mariman E,

et al. Genetic associations with acute stress-related changes in eating in the absence of hunger. Patient Educ Couns. 2010; **79**(3): 367-71.
1633. Rutters F, Lemmens SGT, Born JM, Bouwman F, Nieuwenhuizen AG, Mariman E, et al. Genetic associations with acute stress-related changes in eating in the absence of hunger. Patient Education And Counseling. 2010; **79**(3): 367-71.
1634. Sabia S, Kivimaki M, Kumari M, Shipley MJ, Singh-Manoux A. Effect of Apolipoprotein E epsilon4 on the association between health behaviors and cognitive function in late midlife. Mol Neurodegener. 2010; **5**: 23.
1635. Sabia S, Kivimaki M, Kumari M, Shipley MJ, Singh-Manoux A. Effect of Apolipoprotein E epsilon4 on the association between health behaviors and cognitive function in late midlife. Molecular Neurodegeneration. 2010; **5**: 23-.
1636. Sadowska-Krepa E, Klapcinska B, Pokora I, Domaszewski P, Kempa K, Podgorski T. Effects of Six-Week Ginkgo biloba Supplementation on Aerobic Performance, Blood Pro/Antioxidant Balance, and Serum Brain-Derived Neurotrophic Factor in Physically Active Men. Nutrients. 2017; **9**(8).
1637. Sadowska-Krępa E, Kłapcińska B, Pokora I, Domaszewski P, Kempa K, Podgórski T. Effects of Six-Week Ginkgo biloba Supplementation on Aerobic Performance, Blood Pro/Antioxidant Balance, and Serum Brain-Derived Neurotrophic Factor in Physically Active Men. Nutrients. 2017; **9**(8).
1638. Saito K, Yokoyama T, Yoshiike N, Date C, Yamamoto A, Muramatsu M, et al. Do the ethanol metabolizing enzymes modify the relationship between alcohol consumption and blood pressure? J Hypertens. 2003; **21**(6): 1097-105.
1639. Saito K, Yokoyama T, Yoshiike N, Date C, Yamamoto A, Muramatsu M, et al. Do the ethanol metabolizing enzymes modify the relationship between alcohol consumption and blood pressure? Journal Of Hypertension. 2003; **21**(6): 1097-105.
1640. Saitoh S, Shimoda T, Hamamoto Y, Nakaya Y, Nakajima S. Correlations among obesity-associated gene polymorphisms, body composition, and physical activity in patients with type 2 diabetes mellitus. Indian J Endocrinol Metab. 2015; **19**(1): 66-71.
1641. Saitoh S, Shimoda T, Hamamoto Y, Nakaya Y, Nakajima S. Correlations among obesity-associated gene polymorphisms, body composition, and physical activity in patients with type 2 diabetes mellitus. Indian Journal Of Endocrinology And Metabolism. 2015; **19**(1): 66-71.
1642. Salamone LM, Glynn NW, Black DM, Ferrell RE, Palermo L, Epstein RS, et al. Determinants of premenopausal bone mineral density: the interplay of genetic and lifestyle factors. J Bone Miner Res. 1996; **11**(10): 1557-65.
1643. Salamone LM, Glynn NW, Black DM, Ferrell RE, Palermo L, Epstein RS, et al. Determinants of premenopausal bone mineral density: the interplay of genetic and lifestyle factors. Journal Of Bone And Mineral Research: The Official Journal Of The American Society For Bone And Mineral Research. 1996; **11**(10): 1557-65.
1644. Salas-Gomez D, Fernandez-Gorgojo M, Pozueta A, Diaz-Ceballos I, Lamarain M, Perez C, et al. Binge Drinking in Young University Students Is Associated with Alterations in Executive Functions Related to Their Starting Age. PLoS One. 2016; **11**(11): e0166834.
1645. Salas-Gomez D, Fernandez-Gorgojo M, Pozueta A, Diaz-Ceballos I, Lamarain M, Perez C, et al. Binge Drinking in Young University Students Is Associated with

Alterations in Executive Functions Related to Their Starting Age. PLoS One. 2016; **11**(11): e0166834-e.
1646. Salehpour M, Khodagholi F, Zeinaddini Meymand A, Nourshahi M, Ashabi G. Exercise training with concomitant nitric oxide synthase inhibition improved anxiogenic behavior, spatial cognition, and BDNF/P70S6 kinase activation in 20-month-old rats. Appl Physiol Nutr Metab. 2018; **43**(1): 45-53.
1647. Salehpour M, Khodagholi F, Zeinaddini Meymand A, Nourshahi M, Ashabi G. Exercise training with concomitant nitric oxide synthase inhibition improved anxiogenic behavior, spatial cognition, and BDNF/P70S6 kinase activation in 20-month-old rats. Applied Physiology, Nutrition, And Metabolism = Physiologie Appliquee, Nutrition Et Metabolisme. 2018; **43**(1): 45-53.
1648. Sallaberry C, Ardais AP, Rocha A, Borges MF, Fioreze GT, Mioranzza S, et al. Sex differences in the effects of pre- and postnatal caffeine exposure on behavior and synaptic proteins in pubescent rats. Prog Neuropsychopharmacol Biol Psychiatry. 2018; **81**: 416-25.
1649. Sallaberry C, Ardais AP, Rocha A, Borges MF, Fioreze GT, Mioranzza S, et al. Sex differences in the effects of pre- and postnatal caffeine exposure on behavior and synaptic proteins in pubescent rats. Progress In Neuro-Psychopharmacology & Biological Psychiatry. 2018; **81**: 416-25.
1650. Sampson SL. Strength in Diversity: Hidden Genetic Depths of Mycobacterium tuberculosis. Trends Microbiol. 2016; **24**(2): 82-4.
1651. Sampson SL. Strength in Diversity: Hidden Genetic Depths of Mycobacterium tuberculosis. Trends In Microbiology. 2016; **24**(2): 82-4.
1652. Samuels JF, Riddle MA, Greenberg BD, Fyer AJ, McCracken JT, Rauch SL, et al. The OCD collaborative genetics study: methods and sample description. Am J Med Genet B Neuropsychiatr Genet. 2006; **141B**(3): 201-7.
1653. Samuels JF, Riddle MA, Greenberg BD, Fyer AJ, McCracken JT, Rauch SL, et al. The OCD collaborative genetics study: methods and sample description. American Journal Of Medical Genetics Part B, Neuropsychiatric Genetics: The Official Publication Of The International Society Of Psychiatric Genetics. 2006; **141B**(3): 201-7.
1654. Sanchez J, Bonet ML, Keijer J, van Schothorst EM, Mollller I, Chetrit C, et al. Blood cells transcriptomics as source of potential biomarkers of articular health improvement: effects of oral intake of a rooster combs extract rich in hyaluronic acid. Genes Nutr. 2014; **9**(5): 417.
1655. Sánchez J, Bonet ML, Keijer J, van Schothorst EM, Möllller I, Chetrit C, et al. Blood cells transcriptomics as source of potential biomarkers of articular health improvement: effects of oral intake of a rooster combs extract rich in hyaluronic acid. Genes & Nutrition. 2014; **9**(5): 417-.
1656. Sanna MD, Quattrone A, Galeotti N. Antidepressant-like actions by silencing of neuronal ELAV-like RNA-binding proteins HuB and HuC in a model of depression in male mice. Neuropharmacology. 2018; **135**: 444-54.
1657. Sanna MD, Quattrone A, Galeotti N. Antidepressant-like actions by silencing of neuronal ELAV-like RNA-binding proteins HuB and HuC in a model of depression in male mice. Neuropharmacology. 2018; **135**: 444-54.
1658. Santacana M, Arias B, Mitjans M, Bonillo A, Montoro M, Rosado S, et al.

Predicting Response Trajectories during Cognitive-Behavioural Therapy for Panic Disorder: No Association with the BDNF Gene or Childhood Maltreatment. PLoS One. 2016; **11**(6): e0158224.
1659. Santacana M, Arias B, Mitjans M, Bonillo A, Montoro M, Rosado S, et al. Predicting Response Trajectories during Cognitive-Behavioural Therapy for Panic Disorder: No Association with the BDNF Gene or Childhood Maltreatment. PLoS One. 2016; **11**(6): e0158224-e.
1660. Santucci D, Francia N, Aloe L, Alleva E. Neurobehavioural responses to hypergravity environment in the CD-1 mouse. J Gravit Physiol. 2002; **9**(1): P39-40.
1661. Santucci D, Francia N, Aloe L, Alleva E. Neurobehavioural responses to hypergravity environment in the CD-1 mouse. Journal Of Gravitational Physiology: A Journal Of The International Society For Gravitational Physiology. 2002; **9**(1): P39-P40.
1662. Sasse SK, Kadiyala V, Danhorn T, Panettieri RA, Jr., Phang TL, Gerber AN. Glucocorticoid Receptor ChIP-Seq Identifies PLCD1 as a KLF15 Target that Represses Airway Smooth Muscle Hypertrophy. Am J Respir Cell Mol Biol. 2017; **57**(2): 226-37.
1663. Sasse SK, Kadiyala V, Danhorn T, Panettieri RA, Jr., Phang TL, Gerber AN. Glucocorticoid Receptor ChIP-Seq Identifies PLCD1 as a KLF15 Target that Represses Airway Smooth Muscle Hypertrophy. American Journal Of Respiratory Cell And Molecular Biology. 2017; **57**(2): 226-37.
1664. Saung WT, Narasimhan S, Lohoff FW. Lack of influence of DAT1 and DRD2 gene variants on antidepressant response in generalized anxiety disorder. Hum Psychopharmacol. 2014; **29**(4): 316-21.
1665. Saung WT, Narasimhan S, Lohoff FW. Lack of influence of DAT1 and DRD2 gene variants on antidepressant response in generalized anxiety disorder. Human Psychopharmacology. 2014; **29**(4): 316-21.
1666. Savitz J, Lucki I, Drevets WC. 5-HT(1A) receptor function in major depressive disorder. Prog Neurobiol. 2009; **88**(1): 17-31.
1667. Savitz J, Lucki I, Drevets WC. 5-HT(1A) receptor function in major depressive disorder. Progress In Neurobiology. 2009; **88**(1): 17-31.
1668. Scarmeas N, Luchsinger JA, Schupf N, Brickman AM, Cosentino S, Tang MX, et al. Physical activity, diet, and risk of Alzheimer disease. JAMA. 2009; **302**(6): 627-37.
1669. Scarmeas N, Luchsinger JA, Schupf N, Brickman AM, Cosentino S, Tang MX, et al. Physical activity, diet, and risk of Alzheimer disease. JAMA. 2009; **302**(6): 627-37.
1670. Schaefer TL, Grace CE, Skelton MR, Graham DL, Gudelsky GA, Vorhees CV, et al. Neonatal citalopram treatment inhibits the 5-HT depleting effects of MDMA exposure in rats. ACS Chem Neurosci. 2012; **3**(1): 12-21.
1671. Schaefer TL, Grace CE, Skelton MR, Graham DL, Gudelsky GA, Vorhees CV, et al. Neonatal citalopram treatment inhibits the 5-HT depleting effects of MDMA exposure in rats. ACS Chemical Neuroscience. 2012; **3**(1): 12-21.
1672. Schafer A, Scheurlen M, Weissbrich B, Schottker K, Kraus MR. Sustained virological response in the antiviral therapy of chronic hepatitis C: is there a predictive value of interferon-induced depression? Chemotherapy. 2007; **53**(4): 292-9.
1673. Schäfer A, Scheurlen M, Weissbrich B, Schöttker K, Kraus MR. Sustained virological response in the antiviral therapy of chronic hepatitis C: is there a predictive value of interferon-induced depression? Chemotherapy. 2007; **53**(4): 292-9.

1674. Scheinfeldt LB, Gharani N, Kasper RS, Schmidlen TJ, Gordon ES, Jarvis JP, et al. Using the Coriell Personalized Medicine Collaborative Data to conduct a genome-wide association study of sleep duration. Am J Med Genet B Neuropsychiatr Genet. 2015; **168**(8): 697-705.
1675. Scheinfeldt LB, Gharani N, Kasper RS, Schmidlen TJ, Gordon ES, Jarvis JP, et al. Using the Coriell Personalized Medicine Collaborative Data to conduct a genome-wide association study of sleep duration. American Journal Of Medical Genetics Part B, Neuropsychiatric Genetics: The Official Publication Of The International Society Of Psychiatric Genetics. 2015; **168**(8): 697-705.
1676. Schiepers OJ, van Boxtel MP, de Groot RH, Jolles J, Kok FJ, Verhoef P, et al. DNA methylation and cognitive functioning in healthy older adults. Br J Nutr. 2012; **107**(5): 744-8.
1677. Schiepers OJG, van Boxtel MPJ, de Groot RHM, Jolles J, Kok FJ, Verhoef P, et al. DNA methylation and cognitive functioning in healthy older adults. The British Journal Of Nutrition. 2012; **107**(5): 744-8.
1678. Schillani G, Capozzo MA, Aguglia E, De Vanna M, Grassi L, Conte MA, et al. 5-HTTLPR polymorphism of serotonin transporter and effects of sertraline in terminally ill cancer patients: report of eleven cases. Tumori. 2008; **94**(4): 563-7.
1679. Schillani G, Capozzo MA, Aguglia E, De Vanna M, Grassi L, Conte MA, et al. 5-HTTLPR polymorphism of serotonin transporter and effects of sertraline in terminally ill cancer patients: report of eleven cases. Tumori. 2008; **94**(4): 563-7.
1680. Schillani G, Capozzo MA, Era D, De Vanna M, Grassi L, Conte MA, et al. Pharmacogenetics of escitalopram and mental adaptation to cancer in palliative care: report of 18 cases. Tumori. 2011; **97**(3): 358-61.
1681. Schillani G, Capozzo MA, Era D, De Vanna M, Grassi L, Conte MA, et al. Pharmacogenetics of escitalopram and mental adaptation to cancer in palliative care: report of 18 cases. Tumori. 2011; **97**(3): 358-61.
1682. Schillani G, Martinis E, Capozzo MA, Era D, Cristante T, Mustacchi G, et al. Psychological response to cancer: role of 5-HTTLPR genetic polymorphism of serotonin transporter. Anticancer Research. 2010; **30**(9): 3823-6.
1683. Schillani G, Martinis E, Capozzo MA, Era D, Cristante T, Mustacchi G, et al. Psychological response to cancer: role of 5-HTTLPR genetic polymorphism of serotonin transporter. Anticancer Res. 2010; **30**(9): 3823-6.
1684. Schirmbeck F, Zink M. Comorbid obsessive-compulsive symptoms in schizophrenia: contributions of pharmacological and genetic factors. Front Pharmacol. 2013; **4**: 99.
1685. Schirmbeck F, Zink M. Comorbid obsessive-compulsive symptoms in schizophrenia: contributions of pharmacological and genetic factors. Frontiers In Pharmacology. 2013; **4**: 99-.
1686. Schosser A, Kasper S. The role of pharmacogenetics in the treatment of depression and anxiety disorders. Int Clin Psychopharmacol. 2009; **24**(6): 277-88.
1687. Schosser A, Kasper S. The role of pharmacogenetics in the treatment of depression and anxiety disorders. International Clinical Psychopharmacology. 2009; **24**(6): 277-88.
1688. Schosser A, Serretti A, Souery D, Mendlewicz J, Zohar J, Montgomery S, et al.

European Group for the Study of Resistant Depression (GSRD)--where have we gone so far: review of clinical and genetic findings. Eur Neuropsychopharmacol. 2012; **22**(7): 453-68.
1689. Schosser A, Serretti A, Souery D, Mendlewicz J, Zohar J, Montgomery S, et al. European Group for the Study of Resistant Depression (GSRD)--where have we gone so far: review of clinical and genetic findings. European Neuropsychopharmacology: The Journal Of The European College Of Neuropsychopharmacology. 2012; **22**(7): 453-68.
1690. Schuch FB, Dunn AL, Kanitz AC, Delevatti RS, Fleck MP. Moderators of response in exercise treatment for depression: A systematic review. J Affect Disord. 2016; **195**: 40-9.
1691. Schuch FB, Dunn AL, Kanitz AC, Delevatti RS, Fleck MP. Moderators of response in exercise treatment for depression: A systematic review. Journal Of Affective Disorders. 2016; **195**: 40-9.
1692. Schulze TG, Alda M, Adli M, Akula N, Ardau R, Bui ET, et al. The International Consortium on Lithium Genetics (ConLiGen): an initiative by the NIMH and IGSLI to study the genetic basis of response to lithium treatment. Neuropsychobiology. 2010; **62**(1): 72-8.
1693. Schulze TG, Alda M, Adli M, Akula N, Ardau R, Bui ET, et al. The International Consortium on Lithium Genetics (ConLiGen): an initiative by the NIMH and IGSLI to study the genetic basis of response to lithium treatment. Neuropsychobiology. 2010; **62**(1): 72-8.
1694. Scillitani A, Jang C, Wong BY, Hendy GN, Cole DE. A functional polymorphism in the PTHR1 promoter region is associated with adult height and BMD measured at the femoral neck in a large cohort of young caucasian women. Hum Genet. 2006; **119**(4): 416-21.
1695. Scillitani A, Jang C, Wong BYL, Hendy GN, Cole DEC. A functional polymorphism in the PTHR1 promoter region is associated with adult height and BMD measured at the femoral neck in a large cohort of young caucasian women. Human Genetics. 2006; **119**(4): 416-21.
1696. Scott RA, Bailey ME, Moran CN, Wilson RH, Fuku N, Tanaka M, et al. FTO genotype and adiposity in children: physical activity levels influence the effect of the risk genotype in adolescent males. Eur J Hum Genet. 2010; **18**(12): 1339-43.
1697. Scott RA, Bailey MES, Moran CN, Wilson RH, Fuku N, Tanaka M, et al. FTO genotype and adiposity in children: physical activity levels influence the effect of the risk genotype in adolescent males. European Journal Of Human Genetics: EJHG. 2010; **18**(12): 1339-43.
1698. Seeger G, Schloss P, Schmidt MH. Functional polymorphism within the promotor of the serotonin transporter gene is associated with severe hyperkinetic disorders. Mol Psychiatry. 2001; **6**(2): 235-8.
1699. Seeger G, Schloss P, Schmidt MH. Functional polymorphism within the promotor of the serotonin transporter gene is associated with severe hyperkinetic disorders. Molecular Psychiatry. 2001; **6**(2): 235-8.
1700. Selianina NV, Karakulova Iu V. [The effect of neurotrophic treatment on the activation of reparative processes in patients with acute traumatic brain injury]. Zh

Nevrol Psikhiatr Im S S Korsakova. 2012; **112**(5): 46-9.
1701. Selianina NV, Karakulova IV. [The effect of neurotrophic treatment on the activation of reparative processes in patients with acute traumatic brain injury]. Zhurnal Nevrologii I Psikhiatrii Imeni SS Korsakova. 2012; **112**(5): 46-9.
1702. Semplicini A, Siffert W, Sartori M, Monari A, Naber C, Frigo G, et al. G protein beta3 subunit gene 825T allele is associated with increased left ventricular mass in young subjects with mild hypertension. Am J Hypertens. 2001; **14**(12): 1191-5.
1703. Semplicini A, Siffert W, Sartori M, Monari A, Naber C, Frigo G, et al. G protein beta3 subunit gene 825T allele is associated with increased left ventricular mass in young subjects with mild hypertension. American Journal Of Hypertension. 2001; **14**(12): 1191-5.
1704. Seow A, Shi CY, Chung FL, Jiao D, Hankin JH, Lee HP, et al. Urinary total isothiocyanate (ITC) in a population-based sample of middle-aged and older Chinese in Singapore: relationship with dietary total ITC and glutathione S-transferase M1/T1/P1 genotypes. Cancer Epidemiol Biomarkers Prev. 1998; **7**(9): 775-81.
1705. Seow A, Shi CY, Chung FL, Jiao D, Hankin JH, Lee HP, et al. Urinary total isothiocyanate (ITC) in a population-based sample of middle-aged and older Chinese in Singapore: relationship with dietary total ITC and glutathione S-transferase M1/T1/P1 genotypes. Cancer Epidemiology, Biomarkers & Prevention: A Publication Of The American Association For Cancer Research, Cosponsored By The American Society Of Preventive Oncology. 1998; **7**(9): 775-81.
1706. Serretti A, Souery D, Antypa N, Calati R, Sentissi O, Amital D, et al. The impact of adverse life events on clinical features and interaction with gene variants in mood disorder patients. Psychopathology. 2013; **46**(6): 384-9.
1707. Serretti A, Souery D, Antypa N, Calati R, Sentissi O, Amital D, et al. The impact of adverse life events on clinical features and interaction with gene variants in mood disorder patients. Psychopathology. 2013; **46**(6): 384-9.
1708. Shamir R. Nutrition and growth in inflammatory bowel disease. World Rev Nutr Diet. 2013; **106**: 156-61.
1709. Shamir R. Nutrition and growth in inflammatory bowel disease. World Review Of Nutrition And Dietetics. 2013; **106**: 156-61.
1710. Sharpley CF, Christie DRH, Bitsika V, Andronicos NM, Agnew LL, Richards TM, et al. Comparing a genetic and a psychological factor as correlates of anxiety, depression, and chronic stress in men with prostate cancer. Support Care Cancer. 2018.
1711. Sharpley CF, Christie DRH, Bitsika V, Andronicos NM, Agnew LL, Richards TM, et al. Comparing a genetic and a psychological factor as correlates of anxiety, depression, and chronic stress in men with prostate cancer. Supportive Care In Cancer: Official Journal Of The Multinational Association Of Supportive Care In Cancer. 2018.
1712. Shen L, Wang DQH, Xu M, Woods SC, Liu M. BDNF/TrkB signaling mediates the anorectic action of estradiol in the nucleus tractus solitarius. Oncotarget. 2017; **8**(48): 84028-38.
1713. Shen L, Wang DQH, Xu M, Woods SC, Liu M. BDNF/TrkB signaling mediates the anorectic action of estradiol in the nucleus tractus solitarius. Oncotarget. 2017; **8**(48): 84028-38.

1714. Shen XH, Qian MC, Niu FR, Sun ST, Zhu YP, Yang JH, et al. [Dynamic level observation of brain-derived neurotrophic factor in patients with first-episode generalized anxiety disorder]. Zhonghua Yi Xue Za Zhi. 2011; **91**(41): 2939-41.
1715. Shen X-h, Qian M-c, Niu F-r, Sun S-t, Zhu Y-p, Yang J-h, et al. [Dynamic level observation of brain-derived neurotrophic factor in patients with first-episode generalized anxiety disorder]. Zhonghua Yi Xue Za Zhi. 2011; **91**(41): 2939-41.
1716. Shepard TH, Brent RL, Friedman JM, Jones KL, Miller RK, Moore CA, et al. Update on new developments in the study of human teratogens. Teratology. 2002; **65**(4): 153-61.
1717. Shepard TH, Brent RL, Friedman JM, Jones KL, Miller RK, Moore CA, et al. Update on new developments in the study of human teratogens. Teratology. 2002; **65**(4): 153-61.
1718. Shi J, Xie M, Wang J, Xu Y, Liu X. Susceptibility of N-acetyltransferase 2 slow acetylators to antituberculosis drug-induced liver injury: a meta-analysis. Pharmacogenomics. 2015; **16**(18): 2083-97.
1719. Shi J, Xie M, Wang J, Xu Y, Liu X. Susceptibility of N-acetyltransferase 2 slow acetylators to antituberculosis drug-induced liver injury: a meta-analysis. Pharmacogenomics. 2015; **16**(18): 2083-97.
1720. Shi RX, Ding HT, Li H, Yu ZG, Wu JM, Tang XZ. [Effect of Acupuncture Stimulation of Different Acupoint Groups on Levels of Stress Hormones and Serum Brain-derived Neurotrophic Factor in Depression Rats]. Zhen Ci Yan Jiu. 2015; **40**(6): 444-8.
1721. Shi R-x, Ding H-t, Li H, Yu Z-g, Wu J-m, Tang X-z. [Effect of Acupuncture Stimulation of Different Acupoint Groups on Levels of Stress Hormones and Serum Brain-derived Neurotrophic Factor in Depression Rats]. Zhen Ci Yan Jiu = Acupuncture Research. 2015; **40**(6): 444-8.
1722. Shibata S, Iinuma M, Soumiya H, Fukumitsu H, Furukawa Y, Furukawa S. A novel 2-decenoic acid thioester ameliorates corticosterone-induced depression- and anxiety-like behaviors and normalizes reduced hippocampal signal transduction in treated mice. Pharmacol Res Perspect. 2015; **3**(2): e00132.
1723. Shibata S, Iinuma M, Soumiya H, Fukumitsu H, Furukawa Y, Furukawa S. A novel 2-decenoic acid thioester ameliorates corticosterone-induced depression- and anxiety-like behaviors and normalizes reduced hippocampal signal transduction in treated mice. Pharmacology Research & Perspectives. 2015; **3**(2): e00132-e.
1724. Shim H, Laurent S, Matuszewski S, Foll M, Jensen JD. Detecting and Quantifying Changing Selection Intensities from Time-Sampled Polymorphism Data. G3 (Bethesda). 2016; **6**(4): 893-904.
1725. Shim H, Laurent S, Matuszewski S, Foll M, Jensen JD. Detecting and Quantifying Changing Selection Intensities from Time-Sampled Polymorphism Data. G3 (Bethesda, Md). 2016; **6**(4): 893-904.
1726. Shimada-Sugimoto M, Otowa T, Hettema JM. Genetics of anxiety disorders: Genetic epidemiological and molecular studies in humans. Psychiatry Clin Neurosci. 2015; **69**(7): 388-401.
1727. Shimada-Sugimoto M, Otowa T, Hettema JM. Genetics of anxiety disorders: Genetic epidemiological and molecular studies in humans. Psychiatry And Clinical

Neurosciences. 2015; **69**(7): 388-401.
1728. Shore SA, Johnston RA. Obesity and asthma. Pharmacol Ther. 2006; **110**(1): 83-102.
1729. Shore SA, Johnston RA. Obesity and asthma. Pharmacology & Therapeutics. 2006; **110**(1): 83-102.
1730. Shrubsole MJ, Lu W, Chen Z, Shu XO, Zheng Y, Dai Q, et al. Drinking green tea modestly reduces breast cancer risk. J Nutr. 2009; **139**(2): 310-6.
1731. Shrubsole MJ, Lu W, Chen Z, Shu XO, Zheng Y, Dai Q, et al. Drinking green tea modestly reduces breast cancer risk. The Journal Of Nutrition. 2009; **139**(2): 310-6.
1732. Shuldiner AR, Munir KM. Genetics of obesity: more complicated than initially thought. Lipids. 2003; **38**(2): 97-101.
1733. Shuldiner AR, Munir KM. Genetics of obesity: more complicated than initially thought. Lipids. 2003; **38**(2): 97-101.
1734. Shupak A, Gordon CR. Motion sickness: advances in pathogenesis, prediction, prevention, and treatment. Aviat Space Environ Med. 2006; **77**(12): 1213-23.
1735. Shupak A, Gordon CR. Motion sickness: advances in pathogenesis, prediction, prevention, and treatment. Aviation, Space, And Environmental Medicine. 2006; **77**(12): 1213-23.
1736. Sierksma AS, Rutten K, Sydlik S, Rostamian S, Steinbusch HW, van den Hove DL, et al. Chronic phosphodiesterase type 2 inhibition improves memory in the APPswe/PS1dE9 mouse model of Alzheimer's disease. Neuropharmacology. 2013; **64**: 124-36.
1737. Sierksma ASR, Rutten K, Sydlik S, Rostamian S, Steinbusch HWM, van den Hove DLA, et al. Chronic phosphodiesterase type 2 inhibition improves memory in the APPswe/PS1dE9 mouse model of Alzheimer's disease. Neuropharmacology. 2013; **64**: 124-36.
1738. Silva H, Iturra P, Solari A, Villarroel J, Jerez S, Jimenez M, et al. Fluoxetine response in impulsive-aggressive behavior and serotonin transporter polymorphism in personality disorder. Psychiatr Genet. 2010; **20**(1): 25-30.
1739. Silva H, Iturra P, Solari A, Villarroel J, Jerez S, Jiménez M, et al. Fluoxetine response in impulsive-aggressive behavior and serotonin transporter polymorphism in personality disorder. Psychiatric Genetics. 2010; **20**(1): 25-30.
1740. Silverstein JH, Rosenbloom AL. Type 2 diabetes in children. Curr Diab Rep. 2001; **1**(1): 19-27.
1741. Silverstein JH, Rosenbloom AL. Type 2 diabetes in children. Current Diabetes Reports. 2001; **1**(1): 19-27.
1742. Simmons DA, Belichenko NP, Yang T, Condon C, Monbureau M, Shamloo M, et al. A small molecule TrkB ligand reduces motor impairment and neuropathology in R6/2 and BACHD mouse models of Huntington's disease. J Neurosci. 2013; **33**(48): 18712-27.
1743. Simmons DA, Belichenko NP, Yang T, Condon C, Monbureau M, Shamloo M, et al. A small molecule TrkB ligand reduces motor impairment and neuropathology in R6/2 and BACHD mouse models of Huntington's disease. The Journal Of Neuroscience: The Official Journal Of The Society For Neuroscience. 2013; **33**(48): 18712-27.
1744. Simonato M. Gene therapy for epilepsy. Epilepsy Behav. 2014; **38**: 125-30.
1745. Simonato M. Gene therapy for epilepsy. Epilepsy & Behavior: E&B. 2014; **38**:

125-30.
1746. Singewald N, Schmuckermair C, Whittle N, Holmes A, Ressler KJ. Pharmacology of cognitive enhancers for exposure-based therapy of fear, anxiety and trauma-related disorders. Pharmacol Ther. 2015; **149**: 150-90.
1747. Singewald N, Schmuckermair C, Whittle N, Holmes A, Ressler KJ. Pharmacology of cognitive enhancers for exposure-based therapy of fear, anxiety and trauma-related disorders. Pharmacology & Therapeutics. 2015; **149**: 150-90.
1748. Singhal A, Davies P, Wierenga KJ, Thomas P, Serjeant G. Is there an energy deficiency in homozygous sickle cell disease? Am J Clin Nutr. 1997; **66**(2): 386-90.
1749. Singhal A, Davies P, Wierenga KJ, Thomas P, Serjeant G. Is there an energy deficiency in homozygous sickle cell disease? The American Journal Of Clinical Nutrition. 1997; **66**(2): 386-90.
1750. Siper PM, De Rubeis S, Trelles MDP, Durkin A, Di Marino D, Muratet F, et al. Prospective investigation of FOXP1 syndrome. Mol Autism. 2017; **8**: 57.
1751. Siper PM, De Rubeis S, Trelles MDP, Durkin A, Di Marino D, Muratet F, et al. Prospective investigation of FOXP1 syndrome. Molecular Autism. 2017; **8**: 57-.
1752. Sirota M, Schaub MA, Batzoglou S, Robinson WH, Butte AJ. Autoimmune disease classification by inverse association with SNP alleles. PLoS Genet. 2009; **5**(12): e1000792.
1753. Sirota M, Schaub MA, Batzoglou S, Robinson WH, Butte AJ. Autoimmune disease classification by inverse association with SNP alleles. Plos Genetics. 2009; **5**(12): e1000792-e.
1754. Slattery ML, Murtaugh M, Caan B, Ma KN, Neuhausen S, Samowitz W. Energy balance, insulin-related genes and risk of colon and rectal cancer. Int J Cancer. 2005; **115**(1): 148-54.
1755. Slattery ML, Murtaugh M, Caan B, Ma KN, Neuhausen S, Samowitz W. Energy balance, insulin-related genes and risk of colon and rectal cancer. International Journal Of Cancer. 2005; **115**(1): 148-54.
1756. Slattery ML, Murtaugh M, Caan B, Ma KN, Wolff R, Samowitz W. Associations between BMI, energy intake, energy expenditure, VDR genotype and colon and rectal cancers (United States). Cancer Causes Control. 2004; **15**(9): 863-72.
1757. Slattery ML, Murtaugh M, Caan B, Ma KN, Wolff R, Samowitz W. Associations between BMI, energy intake, energy expenditure, VDR genotype and colon and rectal cancers (United States). Cancer Causes & Control: CCC. 2004; **15**(9): 863-72.
1758. Slattery ML, Murtaugh MA, Sweeney C, Ma KN, Potter JD, Caan BJ, et al. PPARgamma, energy balance, and associations with colon and rectal cancer. Nutr Cancer. 2005; **51**(2): 155-61.
1759. Slattery ML, Murtaugh MA, Sweeney C, Ma K-N, Potter JD, Caan BJ, et al. PPARgamma, energy balance, and associations with colon and rectal cancer. Nutrition And Cancer. 2005; **51**(2): 155-61.
1760. Smith MA, Fronk GE, Abel JM, Lacy RT, Bills SE, Lynch WJ. Resistance exercise decreases heroin self-administration and alters gene expression in the nucleus accumbens of heroin-exposed rats. Psychopharmacology (Berl). 2018; **235**(4): 1245-55.
1761. Smith MA, Fronk GE, Abel JM, Lacy RT, Bills SE, Lynch WJ. Resistance exercise decreases heroin self-administration and alters gene expression in the nucleus

accumbens of heroin-exposed rats. Psychopharmacology. 2018; **235**(4): 1245-55.
1762. Smits JA, Powers MB, Rosenfield D, Zvolensky MJ, Jacquart J, Davis ML, et al. BDNF Val66Met Polymorphism as a Moderator of Exercise Enhancement of Smoking Cessation Treatment in Anxiety Vulnerable Adults. Ment Health Phys Act. 2016; **10**: 73-7.
1763. Smits JAJ, Powers MB, Rosenfield D, Zvolensky MJ, Jacquart J, Davis ML, et al. BDNF Val66Met Polymorphism as a Moderator of Exercise Enhancement of Smoking Cessation Treatment in Anxiety Vulnerable Adults. Mental Health And Physical Activity. 2016; **10**: 73-7.
1764. Soares RN, Schneider A, Valle SC, Schenkel PC. The influence of CYP1A2 genotype in the blood pressure response to caffeine ingestion is affected by physical activity status and caffeine consumption level. Vascul Pharmacol. 2018.
1765. Sober S, Org E, Kepp K, Juhanson P, Eyheramendy S, Gieger C, et al. Targeting 160 candidate genes for blood pressure regulation with a genome-wide genotyping array. PLoS One. 2009; **4**(6): e6034.
1766. Sõber S, Org E, Kepp K, Juhanson P, Eyheramendy S, Gieger C, et al. Targeting 160 candidate genes for blood pressure regulation with a genome-wide genotyping array. PLoS One. 2009; **4**(6): e6034-e.
1767. Son JW, Lee SS, Kim SR, Yoo SJ, Cha BY, Son HY, et al. Low muscle mass and risk of type 2 diabetes in middle-aged and older adults: findings from the KoGES. Diabetologia. 2017; **60**(5): 865-72.
1768. Son JW, Lee SS, Kim SR, Yoo SJ, Cha BY, Son HY, et al. Low muscle mass and risk of type 2 diabetes in middle-aged and older adults: findings from the KoGES. Diabetologia. 2017; **60**(5): 865-72.
1769. Son KY, Son HY, Chae J, Hwang J, Jang S, Yun JM, et al. Genetic association of APOA5 and APOE with metabolic syndrome and their interaction with health-related behavior in Korean men. Lipids Health Dis. 2015; **14**: 105.
1770. Son KY, Son H-Y, Chae J, Hwang J, Jang S, Yun JM, et al. Genetic association of APOA5 and APOE with metabolic syndrome and their interaction with health-related behavior in Korean men. Lipids In Health And Disease. 2015; **14**: 105-.
1771. Sonestedt E, Gullberg B, Ericson U, Wirfalt E, Hedblad B, Orho-Melander M. Association between fat intake, physical activity and mortality depending on genetic variation in FTO. Int J Obes (Lond). 2011; **35**(8): 1041-9.
1772. Sonestedt E, Gullberg B, Ericson U, Wirfält E, Hedblad B, Orho-Melander M. Association between fat intake, physical activity and mortality depending on genetic variation in FTO. International Journal Of Obesity (2005). 2011; **35**(8): 1041-9.
1773. Sonestedt E, Roos C, Gullberg B, Ericson U, Wirfalt E, Orho-Melander M. Fat and carbohydrate intake modify the association between genetic variation in the FTO genotype and obesity. Am J Clin Nutr. 2009; **90**(5): 1418-25.
1774. Sonestedt E, Roos C, Gullberg B, Ericson U, Wirfält E, Orho-Melander M. Fat and carbohydrate intake modify the association between genetic variation in the FTO genotype and obesity. The American Journal Of Clinical Nutrition. 2009; **90**(5): 1418-25.
1775. Song C, Liu BP, Zhang YP, Peng Z, Wang J, Collier AD, et al. Modeling consequences of prolonged strong unpredictable stress in zebrafish: Complex effects on behavior and physiology. Prog Neuropsychopharmacol Biol Psychiatry. 2018; **81**:

384-94.
1776. Song C, Liu B-P, Zhang Y-P, Peng Z, Wang J, Collier AD, et al. Modeling consequences of prolonged strong unpredictable stress in zebrafish: Complex effects on behavior and physiology. Progress In Neuro-Psychopharmacology & Biological Psychiatry. 2018; **81**: 384-94.
1777. Song J, Chang HJ, Tirodkar M, Chang RW, Manheim LM, Dunlop DD. Racial/ethnic differences in activities of daily living disability in older adults with arthritis: a longitudinal study. Arthritis Rheum. 2007; **57**(6): 1058-66.
1778. Song J, Chang HJ, Tirodkar M, Chang RW, Manheim LM, Dunlop DD. Racial/ethnic differences in activities of daily living disability in older adults with arthritis: a longitudinal study. Arthritis And Rheumatism. 2007; **57**(6): 1058-66.
1779. Song M, Gong J, Giovannucci EL, Berndt SI, Brenner H, Chang-Claude J, et al. Genetic variants of adiponectin and risk of colorectal cancer. Int J Cancer. 2015; **137**(1): 154-64.
1780. Song M, Gong J, Giovannucci EL, Berndt SI, Brenner H, Chang-Claude J, et al. Genetic variants of adiponectin and risk of colorectal cancer. International Journal Of Cancer. 2015; **137**(1): 154-64.
1781. Sorensen MD, Chi T, Shara NM, Wang H, Hsi RS, Orchard T, et al. Activity, energy intake, obesity, and the risk of incident kidney stones in postmenopausal women: a report from the Women's Health Initiative. J Am Soc Nephrol. 2014; **25**(2): 362-9.
1782. Sorensen MD, Chi T, Shara NM, Wang H, Hsi RS, Orchard T, et al. Activity, energy intake, obesity, and the risk of incident kidney stones in postmenopausal women: a report from the Women's Health Initiative. Journal Of The American Society Of Nephrology: JASN. 2014; **25**(2): 362-9.
1783. Sridharan K, Modi T, Bendkhale S, Kulkarni D, Gogtay NJ, Thatte UM. Association of Genetic Polymorphisms of CYP2C9 and VKORC1 with Bleeding Following Warfarin: A Case-Control Study. Curr Clin Pharmacol. 2016; **11**(1): 62-8.
1784. Sridharan K, Modi T, Bendkhale S, Kulkarni D, Gogtay NJ, Thatte UM. Association of Genetic Polymorphisms of CYP2C9 and VKORC1 with Bleeding Following Warfarin: A Case-Control Study. Current Clinical Pharmacology. 2016; **11**(1): 62-8.
1785. Stein MB, Seedat S, Gelernter J. Serotonin transporter gene promoter polymorphism predicts SSRI response in generalized social anxiety disorder. Psychopharmacology (Berl). 2006; **187**(1): 68-72.
1786. Stein MB, Seedat S, Gelernter J. Serotonin transporter gene promoter polymorphism predicts SSRI response in generalized social anxiety disorder. Psychopharmacology. 2006; **187**(1): 68-72.
1787. Steinhart AH, Girgrah N, McLeod RS. Reliability of a Crohn's disease clinical classification scheme based on disease behavior. Inflamm Bowel Dis. 1998; **4**(3): 228-34.
1788. Steinhart AH, Girgrah N, McLeod RS. Reliability of a Crohn's disease clinical classification scheme based on disease behavior. Inflammatory Bowel Diseases. 1998; **4**(3): 228-34.
1789. Straube B, Reif A, Richter J, Lueken U, Weber H, Arolt V, et al. The functional

-1019C/G HTR1A polymorphism and mechanisms of fear. Transl Psychiatry. 2014; **4**: e490.
1790. Straube B, Reif A, Richter J, Lueken U, Weber H, Arolt V, et al. The functional -1019C/G HTR1A polymorphism and mechanisms of fear. Translational Psychiatry. 2014; **4**: e490-e.
1791. Su L, Gao S, Unverzagt FW, Cheng Y, Hake AM, Xin P, et al. Selenium Level and Dyslipidemia in Rural Elderly Chinese. PLoS One. 2015; **10**(9): e0136706.
1792. Su L, Gao S, Unverzagt FW, Cheng Y, Hake AM, Xin P, et al. Selenium Level and Dyslipidemia in Rural Elderly Chinese. PLoS One. 2015; **10**(9): e0136706-e.
1793. Su YA, Li JT, Dai WJ, Liao XM, Dong LC, Lu TL, et al. Genetic variation in the tryptophan hydroxylase 2 gene moderates depressive symptom trajectories and remission over 8 weeks of escitalopram treatment. Int Clin Psychopharmacol. 2016; **31**(3): 127-33.
1794. Su Y-A, Li J-T, Dai W-J, Liao X-M, Dong L-C, Lu T-L, et al. Genetic variation in the tryptophan hydroxylase 2 gene moderates depressive symptom trajectories and remission over 8 weeks of escitalopram treatment. International Clinical Psychopharmacology. 2016; **31**(3): 127-33.
1795. Suchanek P, Hubacek JA, Kralova Lesna I, Pinekerova V, Adamkova V. Actigenetic of ACE gene polymorphism in Czech obese sedentary females. Physiol Res. 2009; **58 Suppl 1**: S47-52.
1796. Suchánek P, Hubácek JA, Králová Lesná I, Pinekerová V, Adámková V. Actigenetic of ACE gene polymorphism in Czech obese sedentary females. Physiological Research. 2009; **58 Suppl 1**: S47-S52.
1797. Suchanek P, Kralova-Lesna I, Poledne R, Lanska V, Hubacek JA. An AHSG gene variant modulates basal metabolic rate and body composition development after a short-time lifestyle intervention. Neuro Endocrinol Lett. 2011; **32 Suppl 2**: 32-6.
1798. Suchanek P, Kralova-Lesna I, Poledne R, Lanska V, Hubacek JA. An AHSG gene variant modulates basal metabolic rate and body composition development after a short-time lifestyle intervention. Neuro Endocrinology Letters. 2011; **32 Suppl 2**: 32-6.
1799. Suchanek P, Lanska V, Hubacek JA. Body Composition Changes in Adult Females after Lifestyle Intervention Are Influenced by the NYD-SP18 Variant. Cent Eur J Public Health. 2015; **23 Suppl**: S19-22.
1800. Suchánek P, Lánská V, Hubáček JA. Body Composition Changes in Adult Females after Lifestyle Intervention Are Influenced by the NYD-SP18 Variant. Central European Journal Of Public Health. 2015; **23 Suppl**: S19-S22.
1801. Sukhatme PV. Nutritional adaptation and variability. Eur J Clin Nutr. 1989; **43**(2): 75-87.
1802. Sukhatme PV. Nutritional adaptation and variability. European Journal Of Clinical Nutrition. 1989; **43**(2): 75-87.
1803. Suma S, Naito M, Wakai K, Sasakabe T, Hattori Y, Okada R, et al. Effects of IL6 C-634G polymorphism on tooth loss and their interaction with smoking habits. Oral Dis. 2015; **21**(6): 807-13.
1804. Suma S, Naito M, Wakai K, Sasakabe T, Hattori Y, Okada R, et al. Effects of IL6 C-634G polymorphism on tooth loss and their interaction with smoking habits. Oral Diseases. 2015; **21**(6): 807-13.

1805. Summers J, Jones SE, Anderson MJ. Characterization of the genome of the agent of erythrocyte aplasia permits its classification as a human parvovirus. J Gen Virol. 1983; **64 (Pt 11)**: 2527-32.
1806. Summers J, Jones SE, Anderson MJ. Characterization of the genome of the agent of erythrocyte aplasia permits its classification as a human parvovirus. The Journal Of General Virology. 1983; **64 (Pt 11)**: 2527-32.
1807. Sun X, Patnode CD, Williams C, Senger CA, Kapka TJ, Whitlock EP. 2012.
1808. Surget A, Belzung C. Involvement of vasopressin in affective disorders. Eur J Pharmacol. 2008; **583**(2-3): 340-9.
1809. Surget A, Belzung C. Involvement of vasopressin in affective disorders. European Journal Of Pharmacology. 2008; **583**(2-3): 340-9.
1810. Suzuki A, Kondo T, Mihara K, Yasui-Furukori N, Ishida M, Furukori H, et al. The -141C Ins/Del polymorphism in the dopamine D2 receptor gene promoter region is associated with anxiolytic and antidepressive effects during treatment with dopamine antagonists in schizophrenic patients. Pharmacogenetics. 2001; **11**(6): 545-50.
1811. Suzuki A, Kondo T, Mihara K, Yasui-Furukori N, Ishida M, Furukori H, et al. The -141C Ins/Del polymorphism in the dopamine D2 receptor gene promoter region is associated with anxiolytic and antidepressive effects during treatment with dopamine antagonists in schizophrenic patients. Pharmacogenetics. 2001; **11**(6): 545-50.
1812. Suzuki A, Mihara K, Kondo T, Tanaka O, Nagashima U, Otani K, et al. The relationship between dopamine D2 receptor polymorphism at the Taq1 A locus and therapeutic response to nemonapride, a selective dopamine antagonist, in schizophrenic patients. Pharmacogenetics. 2000; **10**(4): 335-41.
1813. Suzuki A, Mihara K, Kondo T, Tanaka O, Nagashima U, Otani K, et al. The relationship between dopamine D2 receptor polymorphism at the Taq1 A locus and therapeutic response to nemonapride, a selective dopamine antagonist, in schizophrenic patients. Pharmacogenetics. 2000; **10**(4): 335-41.
1814. Swainston Harrison T, Perry CM. Aripiprazole: a review of its use in schizophrenia and schizoaffective disorder. Drugs. 2004; **64**(15): 1715-36.
1815. Swainston Harrison T, Perry CM. Aripiprazole: a review of its use in schizophrenia and schizoaffective disorder. Drugs. 2004; **64**(15): 1715-36.
1816. Takeuchi T, Oota K, Harada S, Edogawa S, Kojima Y, Sanomura M, et al. Characteristics of refractory gastroesophageal reflux disease (GERD) symptoms -is switching proton pump inhibitors based on the patient's CYP2C19 genotype an effective management strategy? Intern Med. 2015; **54**(2): 97-105.
1817. Takeuchi T, Oota K, Harada S, Edogawa S, Kojima Y, Sanomura M, et al. Characteristics of refractory gastroesophageal reflux disease (GERD) symptoms -is switching proton pump inhibitors based on the patient's CYP2C19 genotype an effective management strategy? Internal Medicine (Tokyo, Japan). 2015; **54**(2): 97-105.
1818. Tan EK, Lu ZY, Fook-Chong SM, Tan E, Shen H, Chua E, et al. Exploring an interaction of adenosine A2A receptor variability with coffee and tea intake in Parkinson's disease. Am J Med Genet B Neuropsychiatr Genet. 2006; **141B**(6): 634-6.
1819. Tan EK, Lu ZY, Fook-Chong SMC, Tan E, Shen H, Chua E, et al. Exploring an interaction of adenosine A2A receptor variability with coffee and tea intake in Parkinson's disease. American Journal Of Medical Genetics Part B, Neuropsychiatric

Genetics: The Official Publication Of The International Society Of Psychiatric Genetics. 2006; **141B**(6): 634-6.
1820. Tang LS, Masur J, Sims Z, Nelson A, Osinusi A, Kohli A, et al. Safe and effective sofosbuvir-based therapy in patients with mental health disease on hepatitis C virus treatment. World J Hepatol. 2016; **8**(31): 1318-26.
1821. Tang LSY, Masur J, Sims Z, Nelson A, Osinusi A, Kohli A, et al. Safe and effective sofosbuvir-based therapy in patients with mental health disease on hepatitis C virus treatment. World Journal Of Hepatology. 2016; **8**(31): 1318-26.
1822. Tang N, Yin S, Sun Z, Xu X, Qin J. The relationship between on-clopidogrel platelet reactivity, genotype, and post-percutaneous coronary intervention outcomes in Chinese patients. Scand J Clin Lab Invest. 2015; **75**(3): 223-9.
1823. Tang N, Yin S, Sun Z, Xu X, Qin J. The relationship between on-clopidogrel platelet reactivity, genotype, and post-percutaneous coronary intervention outcomes in Chinese patients. Scandinavian Journal Of Clinical And Laboratory Investigation. 2015; **75**(3): 223-9.
1824. Tanisawa K, Ito T, Sun X, Ise R, Oshima S, Cao ZB, et al. Strong influence of dietary intake and physical activity on body fatness in elderly Japanese men: age-associated loss of polygenic resistance against obesity. Genes Nutr. 2014; **9**(5): 416.
1825. Tanisawa K, Ito T, Sun X, Ise R, Oshima S, Cao Z-B, et al. Strong influence of dietary intake and physical activity on body fatness in elderly Japanese men: age-associated loss of polygenic resistance against obesity. Genes & Nutrition. 2014; **9**(5): 416-.
1826. Tarnopolsky MA, Safdar A. The potential benefits of creatine and conjugated linoleic acid as adjuncts to resistance training in older adults. Appl Physiol Nutr Metab. 2008; **33**(1): 213-27.
1827. Tarnopolsky MA, Safdar A. The potential benefits of creatine and conjugated linoleic acid as adjuncts to resistance training in older adults. Applied Physiology, Nutrition, And Metabolism = Physiologie Appliquee, Nutrition Et Metabolisme. 2008; **33**(1): 213-27.
1828. Tartaglia NR, Howell S, Sutherland A, Wilson R, Wilson L. A review of trisomy X (47,XXX). Orphanet J Rare Dis. 2010; **5**: 8.
1829. Tartaglia NR, Howell S, Sutherland A, Wilson R, Wilson L. A review of trisomy X (47,XXX). Orphanet Journal Of Rare Diseases. 2010; **5**: 8-.
1830. Taylor JY, Maddox R, Wu CY. Genetic and environmental risks for high blood pressure among African American mothers and daughters. Biol Res Nurs. 2009; **11**(1): 53-65.
1831. Taylor JY, Maddox R, Wu CY. Genetic and environmental risks for high blood pressure among African American mothers and daughters. Biological Research For Nursing. 2009; **11**(1): 53-65.
1832. Teede H, Deeks A, Moran L. Polycystic ovary syndrome: a complex condition with psychological, reproductive and metabolic manifestations that impacts on health across the lifespan. BMC Med. 2010; **8**: 41.
1833. Teede H, Deeks A, Moran L. Polycystic ovary syndrome: a complex condition with psychological, reproductive and metabolic manifestations that impacts on health across the lifespan. BMC Medicine. 2010; **8**: 41-.

1834. Telch MJ, Beevers CG, Rosenfield D, Lee HJ, Reijntjes A, Ferrell RE, et al. 5-HTTLPR genotype potentiates the effects of war zone stressors on the emergence of PTSD, depressive and anxiety symptoms in soldiers deployed to iraq. World Psychiatry. 2015; **14**(2): 198-206.
1835. Telch MJ, Beevers CG, Rosenfield D, Lee H-J, Reijntjes A, Ferrell RE, et al. 5-HTTLPR genotype potentiates the effects of war zone stressors on the emergence of PTSD, depressive and anxiety symptoms in soldiers deployed to iraq. World Psychiatry: Official Journal Of The World Psychiatric Association (WPA). 2015; **14**(2): 198-206.
1836. Temmingh H, Stein DJ. Anxiety in Patients with Schizophrenia: Epidemiology and Management. CNS Drugs. 2015; **29**(10): 819-32.
1837. Temmingh H, Stein DJ. Anxiety in Patients with Schizophrenia: Epidemiology and Management. CNS Drugs. 2015; **29**(10): 819-32.
1838. Tempier A, He J, Zhu S, Zhang R, Kong L, Tan Q, et al. Quetiapine modulates conditioned anxiety and alternation behavior in Alzheimer's transgenic mice. Current Alzheimer Research. 2013; **10**(2): 199-206.
1839. Tempier A, He J, Zhu S, Zhang R, Kong L, Tan Q, et al. Quetiapine modulates conditioned anxiety and alternation behavior in Alzheimer's transgenic mice. Curr Alzheimer Res. 2013; **10**(2): 199-206.
1840. Terracciano C, Rastelli E, Morello M, Celi M, Bucci E, Antonini G, et al. Vitamin D deficiency in myotonic dystrophy type 1. J Neurol. 2013; **260**(9): 2330-4.
1841. Terracciano C, Rastelli E, Morello M, Celi M, Bucci E, Antonini G, et al. Vitamin D deficiency in myotonic dystrophy type 1. Journal Of Neurology. 2013; **260**(9): 2330-4.
1842. Theodoridi A, Tsalafouta A, Pavlidis M. Acute Exposure to Fluoxetine Alters Aggressive Behavior of Zebrafish and Expression of Genes Involved in Serotonergic System Regulation. Front Neurosci. 2017; **11**: 223.
1843. Theodoridi A, Tsalafouta A, Pavlidis M. Acute Exposure to Fluoxetine Alters Aggressive Behavior of Zebrafish and Expression of Genes Involved in Serotonergic System Regulation. Frontiers In Neuroscience. 2017; **11**: 223-.
1844. Theppeang K, Glass TA, Bandeen-Roche K, Todd AC, Rohde CA, Schwartz BS. Gender and race/ethnicity differences in lead dose biomarkers. Am J Public Health. 2008; **98**(7): 1248-55.
1845. Theppeang K, Glass TA, Bandeen-Roche K, Todd AC, Rohde CA, Schwartz BS. Gender and race/ethnicity differences in lead dose biomarkers. American Journal Of Public Health. 2008; **98**(7): 1248-55.
1846. Thomas GN, Tomlinson B, Chan JC, Young RP, Critchley JA. The Trp64Arg polymorphism of the beta3-adrenergic receptor gene and obesity in Chinese subjects with components of the metabolic syndrome. Int J Obes Relat Metab Disord. 2000; **24**(5): 545-51.
1847. Thomas GN, Tomlinson B, Chan JC, Young RP, Critchley JA. The Trp64Arg polymorphism of the beta3-adrenergic receptor gene and obesity in Chinese subjects with components of the metabolic syndrome. International Journal Of Obesity And Related Metabolic Disorders: Journal Of The International Association For The Study Of Obesity. 2000; **24**(5): 545-51.
1848. Thomas RM, Algrain HA, Ryan EJ, Popojas A, Carrigan P, Abdulrahman A, et al. Influence of a CYP1A2 polymorphism on post-exercise heart rate variability in response

to caffeine intake: a double-blind, placebo-controlled trial. Ir J Med Sci. 2017; **186**(2): 285-91.
1849. Thomas RM, Algrain HA, Ryan EJ, Popojas A, Carrigan P, Abdulrahman A, et al. Influence of a CYP1A2 polymorphism on post-exercise heart rate variability in response to caffeine intake: a double-blind, placebo-controlled trial. Irish Journal Of Medical Science. 2017; **186**(2): 285-91.
1850. Thomas SA, Weeks JW, Dougherty LR, Lipton MF, Daruwala SE, Kline K, et al. Allelic Variation of Risk for Anxiety Symptoms Moderates the Relation Between Adolescent Safety Behaviors and Social Anxiety Symptoms. J Psychopathol Behav Assess. 2015; **37**(4): 597-610.
1851. Thomas SA, Weeks JW, Dougherty LR, Lipton MF, Daruwala SE, Kline K, et al. Allelic Variation of Risk for Anxiety Symptoms Moderates the Relation Between Adolescent Safety Behaviors and Social Anxiety Symptoms. Journal Of Psychopathology And Behavioral Assessment. 2015; **37**(4): 597-610.
1852. Thompson AJ, Santoro R, Piazzolla V, Clark PJ, Naggie S, Tillmann HL, et al. Inosine triphosphatase genetic variants are protective against anemia during antiviral therapy for HCV2/3 but do not decrease dose reductions of RBV or increase SVR. Hepatology. 2011; **53**(2): 389-95.
1853. Thompson AJ, Santoro R, Piazzolla V, Clark PJ, Naggie S, Tillmann HL, et al. Inosine triphosphatase genetic variants are protective against anemia during antiviral therapy for HCV2/3 but do not decrease dose reductions of RBV or increase SVR. Hepatology (Baltimore, Md). 2011; **53**(2): 389-95.
1854. Thompson AK, Shaw DI, Minihane AM, Williams CM. Trans-fatty acids and cancer: the evidence reviewed. Nutr Res Rev. 2008; **21**(2): 174-88.
1855. Thompson AK, Shaw DI, Minihane AM, Williams CM. Trans-fatty acids and cancer: the evidence reviewed. Nutrition Research Reviews. 2008; **21**(2): 174-88.
1856. Timmons JA, Knudsen S, Rankinen T, Koch LG, Sarzynski M, Jensen T, et al. Using molecular classification to predict gains in maximal aerobic capacity following endurance exercise training in humans. J Appl Physiol (1985). 2010; **108**(6): 1487-96.
1857. Timmons JA, Knudsen S, Rankinen T, Koch LG, Sarzynski M, Jensen T, et al. Using molecular classification to predict gains in maximal aerobic capacity following endurance exercise training in humans. Journal Of Applied Physiology (Bethesda, Md: 1985). 2010; **108**(6): 1487-96.
1858. Tiwari AK, Rodgers JB, Sicard M, Zai CC, Likhodi O, Freeman N, et al. Association study of polymorphisms in cholecystokinin gene and its receptors with antipsychotic induced weight gain in schizophrenia patients. Prog Neuropsychopharmacol Biol Psychiatry. 2010; **34**(8): 1484-90.
1859. Tiwari AK, Rodgers JB, Sicard M, Zai CC, Likhodi O, Freeman N, et al. Association study of polymorphisms in cholecystokinin gene and its receptors with antipsychotic induced weight gain in schizophrenia patients. Progress In Neuro-Psychopharmacology & Biological Psychiatry. 2010; **34**(8): 1484-90.
1860. Tiwari AK, Souza RP, Muller DJ. Pharmacogenetics of anxiolytic drugs. J Neural Transm (Vienna). 2009; **116**(6): 667-77.
1861. Tiwari AK, Souza RP, Müller DJ. Pharmacogenetics of anxiolytic drugs. Journal Of Neural Transmission (Vienna, Austria: 1996). 2009; **116**(6): 667-77.

1862. Tognon G, Nilsson LM, Shungin D, Lissner L, Jansson JH, Renstrom F, et al. Nonfermented milk and other dairy products: associations with all-cause mortality. Am J Clin Nutr. 2017; **105**(6): 1502-11.
1863. Tognon G, Nilsson LM, Shungin D, Lissner L, Jansson J-H, Renström F, et al. Nonfermented milk and other dairy products: associations with all-cause mortality. The American Journal Of Clinical Nutrition. 2017; **105**(6): 1502-11.
1864. Togsverd M, Werge TM, Tanko LB, Bagger YZ, Qin GG, Hansen T, et al. Cognitive performance in elderly women: significance of the 19bp insertion/deletion polymorphism in the 5' flank of the dopamine beta-hydroxylase gene, educational level, body fat measures, serum triglyceride, alcohol consumption and age. Int J Geriatr Psychiatry. 2007; **22**(9): 883-9.
1865. Togsverd M, Werge TM, Tankó LB, Bagger YZ, Qin GG, Hansen T, et al. Cognitive performance in elderly women: significance of the 19bp insertion/deletion polymorphism in the 5' flank of the dopamine beta-hydroxylase gene, educational level, body fat measures, serum triglyceride, alcohol consumption and age. International Journal Of Geriatric Psychiatry. 2007; **22**(9): 883-9.
1866. Tolonen S, Laaksonen M, Mikkila V, Sievanen H, Mononen N, Rasanen L, et al. Lactase gene c/t(-13910) polymorphism, calcium intake, and pQCT bone traits in Finnish adults. Calcif Tissue Int. 2011; **88**(2): 153-61.
1867. Tolonen S, Laaksonen M, Mikkilä V, Sievänen H, Mononen N, Räsänen L, et al. Lactase gene c/t(-13910) polymorphism, calcium intake, and pQCT bone traits in Finnish adults. Calcified Tissue International. 2011; **88**(2): 153-61.
1868. Tolonen S, Mikkila V, Laaksonen M, Sievanen H, Mononen N, Hernesniemi J, et al. Association of apolipoprotein E promoter polymorphisms with bone structural traits is modified by dietary saturated fat intake - the Cardiovascular Risk in Young Finns study. Bone. 2011; **48**(5): 1058-65.
1869. Tolonen S, Mikkilä V, Laaksonen M, Sievänen H, Mononen N, Hernesniemi J, et al. Association of apolipoprotein E promoter polymorphisms with bone structural traits is modified by dietary saturated fat intake - the Cardiovascular Risk in Young Finns study. Bone. 2011; **48**(5): 1058-65.
1870. Tomezsko JL, Stallings VA, Kawchak DA, Goin JE, Diamond G, Scanlin TF. Energy expenditure and genotype of children with cystic fibrosis. Pediatr Res. 1994; **35**(4 Pt 1): 451-60.
1871. Tomezsko JL, Stallings VA, Kawchak DA, Goin JE, Diamond G, Scanlin TF. Energy expenditure and genotype of children with cystic fibrosis. Pediatric Research. 1994; **35**(4 Pt 1): 451-60.
1872. Trautmann S, Richter J, Muehlhan M, Hofler M, Wittchen HU, Domschke K, et al. Does prior traumatization affect the treatment outcome of CBT for panic disorder? The potential role of the MAOA gene and depression symptoms. Eur Arch Psychiatry Clin Neurosci. 2017.
1873. Trautmann S, Richter J, Muehlhan M, Höfler M, Wittchen H-U, Domschke K, et al. Does prior traumatization affect the treatment outcome of CBT for panic disorder? The potential role of the MAOA gene and depression symptoms. European Archives Of Psychiatry And Clinical Neuroscience. 2017.
1874. Treiber KA, Lyketsos CG, Corcoran C, Steinberg M, Norton M, Green RC, et al.

Vascular factors and risk for neuropsychiatric symptoms in Alzheimer's disease: the Cache County Study. Int Psychogeriatr. 2008; **20**(3): 538-53.
1875. Treiber KA, Lyketsos CG, Corcoran C, Steinberg M, Norton M, Green RC, et al. Vascular factors and risk for neuropsychiatric symptoms in Alzheimer's disease: the Cache County Study. International Psychogeriatrics. 2008; **20**(3): 538-53.
1876. Tremblay A, Bouchard L, Bouchard C, Despres JP, Drapeau V, Perusse L. Long-term adiposity changes are related to a glucocorticoid receptor polymorphism in young females. J Clin Endocrinol Metab. 2003; **88**(7): 3141-5.
1877. Tremblay A, Bouchard L, Bouchard C, Després J-P, Drapeau V, Pérusse L. Long-term adiposity changes are related to a glucocorticoid receptor polymorphism in young females. The Journal Of Clinical Endocrinology And Metabolism. 2003; **88**(7): 3141-5.
1878. Trueb RM, Dias M. Alopecia Areata: a Comprehensive Review of Pathogenesis and Management. Clin Rev Allergy Immunol. 2018; **54**(1): 68-87.
1879. Trüeb RM, Dias MFRG. Alopecia Areata: a Comprehensive Review of Pathogenesis and Management. Clinical Reviews In Allergy & Immunology. 2018; **54**(1): 68-87.
1880. Tsai CL, Ukropec J, Ukropcova B, Pai MC. An acute bout of aerobic or strength exercise specifically modifies circulating exerkine levels and neurocognitive functions in elderly individuals with mild cognitive impairment. Neuroimage Clin. 2018; **17**: 272-84.
1881. Tsai C-L, Ukropec J, Ukropcová B, Pai M-C. An acute bout of aerobic or strength exercise specifically modifies circulating exerkine levels and neurocognitive functions in elderly individuals with mild cognitive impairment. Neuroimage Clinical. 2017; **17**: 272-84.
1882. Tseng YT, Chen CS, Jong YJ, Chang FR, Lo YC. Loganin possesses neuroprotective properties, restores SMN protein and activates protein synthesis positive regulator Akt/mTOR in experimental models of spinal muscular atrophy. Pharmacol Res. 2016; **111**: 58-75.
1883. Tseng Y-T, Chen C-S, Jong Y-J, Chang F-R, Lo Y-C. Loganin possesses neuroprotective properties, restores SMN protein and activates protein synthesis positive regulator Akt/mTOR in experimental models of spinal muscular atrophy. Pharmacological Research. 2016; **111**: 58-75.
1884. Turner JR, Ray R, Lee B, Everett L, Xiang J, Jepson C, et al. Evidence from mouse and man for a role of neuregulin 3 in nicotine dependence. Mol Psychiatry. 2014; **19**(7): 801-10.
1885. Turner JR, Ray R, Lee B, Everett L, Xiang J, Jepson C, et al. Evidence from mouse and man for a role of neuregulin 3 in nicotine dependence. Molecular Psychiatry. 2014; **19**(7): 801-10.
1886. Tyson C, Sharp AJ, Hrynchak M, Yong SL, Hollox EJ, Warburton P, et al. Expansion of a 12-kb VNTR containing the REXO1L1 gene cluster underlies the microscopically visible euchromatic variant of 8q21.2. Eur J Hum Genet. 2014; **22**(4): 458-63.
1887. Tyson C, Sharp AJ, Hrynchak M, Yong SL, Hollox EJ, Warburton P, et al. Expansion of a 12-kb VNTR containing the REXO1L1 gene cluster underlies the microscopically visible euchromatic variant of 8q21.2. European Journal Of Human Genetics: EJHG. 2014; **22**(4): 458-63.

1888. Udina M, Moreno-Espana J, Navines R, Gimenez D, Langohr K, Gratacos M, et al. Serotonin and interleukin-6: the role of genetic polymorphisms in IFN-induced neuropsychiatric symptoms. Psychoneuroendocrinology. 2013; **38**(9): 1803-13.
1889. Udina M, Moreno-España J, Navinés R, Giménez D, Langohr K, Gratacòs M, et al. Serotonin and interleukin-6: the role of genetic polymorphisms in IFN-induced neuropsychiatric symptoms. Psychoneuroendocrinology. 2013; **38**(9): 1803-13.
1890. Udina M, Navines R, Egmond E, Oriolo G, Langohr K, Gimenez D, et al. Glucocorticoid Receptors, Brain-Derived Neurotrophic Factor, Serotonin and Dopamine Neurotransmission are Associated with Interferon-Induced Depression. Int J Neuropsychopharmacol. 2016; **19**(4).
1891. Udina M, Navinés R, Egmond E, Oriolo G, Langohr K, Gimenez D, et al. Glucocorticoid Receptors, Brain-Derived Neurotrophic Factor, Serotonin and Dopamine Neurotransmission are Associated with Interferon-Induced Depression. The International Journal Of Neuropsychopharmacology. 2016; **19**(4).
1892. Uhl GR, Drgonova J, Hall FS. Curious cases: Altered dose-response relationships in addiction genetics. Pharmacol Ther. 2014; **141**(3): 335-46.
1893. Uhl GR, Drgonova J, Hall FS. Curious cases: Altered dose-response relationships in addiction genetics. Pharmacology & Therapeutics. 2014; **141**(3): 335-46.
1894. Urry E, Jetter A, Holst SC, Berger W, Spinas GA, Langhans W, et al. A case-control field study on the relationships among type 2 diabetes, sleepiness and habitual caffeine intake. Journal Of Psychopharmacology (Oxford, England). 2017; **31**(2): 233-42.
1895. Urry E, Jetter A, Holst SC, Berger W, Spinas GA, Langhans W, et al. A case-control field study on the relationships among type 2 diabetes, sleepiness and habitual caffeine intake. J Psychopharmacol. 2017; **31**(2): 233-42.
1896. Urry E, Jetter A, Landolt HP. Assessment of CYP1A2 enzyme activity in relation to type-2 diabetes and habitual caffeine intake. Nutr Metab (Lond). 2016; **13**: 66.
1897. Uutela M, Lindholm J, Rantamaki T, Umemori J, Hunter K, Voikar V, et al. Distinctive behavioral and cellular responses to fluoxetine in the mouse model for Fragile X syndrome. Front Cell Neurosci. 2014; **8**: 150.
1898. Uutela M, Lindholm J, Rantamäki T, Umemori J, Hunter K, Võikar V, et al. Distinctive behavioral and cellular responses to fluoxetine in the mouse model for Fragile X syndrome. Frontiers In Cellular Neuroscience. 2014; **8**: 150-.
1899. Valsalan R, Manoj N. Evolutionary history of the neuropeptide S receptor/neuropeptide S system. Gen Comp Endocrinol. 2014; **209**: 11-20.
1900. Valsalan R, Manoj N. Evolutionary history of the neuropeptide S receptor/neuropeptide S system. General And Comparative Endocrinology. 2014; **209**: 11-20.
1901. Valtuena J, Gonzalez-Gross M, Huybrechts I, Breidenassel C, Ferrari M, Mouratidou T, et al. Factors associated with vitamin D deficiency in European adolescents: the HELENA study. J Nutr Sci Vitaminol (Tokyo). 2013; **59**(3): 161-71.
1902. Valtueña J, González-Gross M, Huybrechts I, Breidenassel C, Ferrari M, Mouratidou T, et al. Factors associated with vitamin D deficiency in European adolescents: the HELENA study. Journal Of Nutritional Science And Vitaminology. 2013; **59**(3): 161-71.

1903. van Rossum CT, Hoebee B, Seidell JC, Bouchard C, van Baak MA, de Groot CP, et al. Genetic factors as predictors of weight gain in young adult Dutch men and women. Int J Obes Relat Metab Disord. 2002; **26**(4): 517-28.
1904. van Rossum CTM, Hoebee B, Seidell JC, Bouchard C, van Baak MA, de Groot CPGM, et al. Genetic factors as predictors of weight gain in young adult Dutch men and women. International Journal Of Obesity And Related Metabolic Disorders: Journal Of The International Association For The Study Of Obesity. 2002; **26**(4): 517-28.
1905. van Schie RM, el Khedr N, Verhoef TI, Teichert M, Stricker BH, Hofman A, et al. Validation of the acenocoumarol EU-PACT algorithms: similar performance in the Rotterdam Study cohort as in the original study. Pharmacogenomics. 2012; **13**(11): 1239-45.
1906. van Schie RMF, el Khedr N, Verhoef TI, Teichert M, Stricker BH, Hofman A, et al. Validation of the acenocoumarol EU-PACT algorithms: similar performance in the Rotterdam Study cohort as in the original study. Pharmacogenomics. 2012; **13**(11): 1239-45.
1907. van Tilburg J, van Haeften TW, Pearson P, Wijmenga C. Defining the genetic contribution of type 2 diabetes mellitus. J Med Genet. 2001; **38**(9): 569-78.
1908. van Tilburg J, van Haeften TW, Pearson P, Wijmenga C. Defining the genetic contribution of type 2 diabetes mellitus. Journal Of Medical Genetics. 2001; **38**(9): 569-78.
1909. Vardy J, Dhillon HM, Pond GR, Rourke SB, Xu W, Dodd A, et al. Cognitive function and fatigue after diagnosis of colorectal cancer. Ann Oncol. 2014; **25**(12): 2404-12.
1910. Vardy J, Dhillon HM, Pond GR, Rourke SB, Xu W, Dodd A, et al. Cognitive function and fatigue after diagnosis of colorectal cancer. Annals Of Oncology: Official Journal Of The European Society For Medical Oncology. 2014; **25**(12): 2404-12.
1911. Velez Edwards DR, Naj AC, Monda K, North KE, Neuhouser M, Magvanjav O, et al. Gene-environment interactions and obesity traits among postmenopausal African-American and Hispanic women in the Women's Health Initiative SHARe Study. Hum Genet. 2013; **132**(3): 323-36.
1912. Velez Edwards DR, Naj AC, Monda K, North KE, Neuhouser M, Magvanjav O, et al. Gene-environment interactions and obesity traits among postmenopausal African-American and Hispanic women in the Women's Health Initiative SHARe Study. Human Genetics. 2013; **132**(3): 323-36.
1913. Vellers HL, Kleeberger SR, Lightfoot JT. Inter-individual variation in adaptations to endurance and resistance exercise training: genetic approaches towards understanding a complex phenotype. Mamm Genome. 2018; **29**(1-2): 48-62.
1914. Vellers HL, Kleeberger SR, Lightfoot JT. Inter-individual variation in adaptations to endurance and resistance exercise training: genetic approaches towards understanding a complex phenotype. Mammalian Genome: Official Journal Of The International Mammalian Genome Society. 2018; **29**(1-2): 48-62.
1915. Verhoeven W, Egger J, Brunner H, de Leeuw N. A patient with a de novo distal 22q11.2 microdeletion and anxiety disorder. Am J Med Genet A. 2011; **155A**(2): 392-7.
1916. Verhoeven W, Egger J, Brunner H, de Leeuw N. A patient with a de novo distal 22q11.2 microdeletion and anxiety disorder. American Journal Of Medical Genetics Part

A. 2011; **155A**(2): 392-7.
1917. Veronesi F, Dallari D, Sabbioni G, Carubbi C, Martini L, Fini M. Polydeoxyribonucleotides (PDRNs) From Skin to Musculoskeletal Tissue Regeneration via Adenosine A2A Receptor Involvement. J Cell Physiol. 2017; **232**(9): 2299-307.
1918. Veronesi F, Dallari D, Sabbioni G, Carubbi C, Martini L, Fini M. Polydeoxyribonucleotides (PDRNs) From Skin to Musculoskeletal Tissue Regeneration via Adenosine A2A Receptor Involvement. Journal Of Cellular Physiology. 2017; **232**(9): 2299-307.
1919. Vicente-Rodriguez G, Libersa C, Mesana MI, Beghin L, Iliescu C, Moreno Aznar LA, et al. Healthy lifestyle by nutrition in adolescence (HELENA). A new EU funded project. Therapie. 2007; **62**(3): 259-70.
1920. Vicente-Rodriguez G, Libersa C, Mesana MI, Béghin L, Iliescu C, Moreno Aznar LA, et al. Healthy lifestyle by nutrition in adolescence (HELENA). A new EU funded project. Therapie. 2007; **62**(3): 259-70.
1921. Viikki M, Kampman O, Seppala N, Mononen N, Lehtimaki T, Leinonen E. CYP1A2 polymorphism -1545C > T (rs2470890) is associated with increased side effects to clozapine. BMC Psychiatry. 2014; **14**: 50.
1922. Viikki M, Kampman O, Seppälä N, Mononen N, Lehtimäki T, Leinonen E. CYP1A2 polymorphism -1545C > T (rs2470890) is associated with increased side effects to clozapine. BMC Psychiatry. 2014; **14**: 50-.
1923. Vimaleswaran KS, Bodhini D, Lakshmipriya N, Ramya K, Anjana RM, Sudha V, et al. Interaction between FTO gene variants and lifestyle factors on metabolic traits in an Asian Indian population. Nutr Metab (Lond). 2016; **13**: 39.
1924. Vimaleswaran KS, Bodhini D, Lakshmipriya N, Ramya K, Anjana RM, Sudha V, et al. Interaction between FTO gene variants and lifestyle factors on metabolic traits in an Asian Indian population. Nutrition & Metabolism. 2016; **13**: 39-.
1925. Vimaleswaran KS, Cavadino A, Verweij N, Nolte IM, Mateo Leach I, Auvinen J, et al. Interactions between uncoupling protein 2 gene polymorphisms, obesity and alcohol intake on liver function: a large meta-analysed population-based study. Eur J Endocrinol. 2015; **173**(6): 863-72.
1926. Vimaleswaran KS, Cavadino A, Verweij N, Nolte IM, Mateo Leach I, Auvinen J, et al. Interactions between uncoupling protein 2 gene polymorphisms, obesity and alcohol intake on liver function: a large meta-analysed population-based study. European Journal Of Endocrinology. 2015; **173**(6): 863-72.
1927. Vinberg M. Risk. Impact of having a first-degree relative with affective disorder: a 7-year follow-up study. Dan Med J. 2016; **63**(10).
1928. Vinberg M. Risk. Impact of having a first-degree relative with affective disorder: a 7-year follow-up study. Danish Medical Journal. 2016; **63**(10).
1929. Virk J, Liew Z, Olsen J, Nohr EA, Catov JM, Ritz B. Pre-conceptual and prenatal supplementary folic acid and multivitamin intake, behavioral problems, and hyperkinetic disorders: A study based on the Danish National Birth Cohort (DNBC). Nutr Neurosci. 2017: 1-9.
1930. Virk J, Liew Z, Olsen J, Nohr EA, Catov JM, Ritz B. Pre-conceptual and prenatal supplementary folic acid and multivitamin intake, behavioral problems, and hyperkinetic disorders: A study based on the Danish National Birth Cohort (DNBC). Nutritional

Neuroscience. 2018; **21**(5): 352-60.
1931. Voisey J, Young RM, Lawford BR, Morris CP. Progress towards understanding the genetics of posttraumatic stress disorder. J Anxiety Disord. 2014; **28**(8): 873-83.
1932. Voisey J, Young RM, Lawford BR, Morris CP. Progress towards understanding the genetics of posttraumatic stress disorder. Journal Of Anxiety Disorders. 2014; **28**(8): 873-83.
1933. Volkers KM, Scherder EJ. The effect of regular walks on various health aspects in older people with dementia: protocol of a randomized-controlled trial. BMC Geriatr. 2011; **11**: 38.
1934. Volkers KM, Scherder EJA. The effect of regular walks on various health aspects in older people with dementia: protocol of a randomized-controlled trial. BMC Geriatrics. 2011; **11**: 38-.
1935. von Lubitz DK. Adenosine and cerebral ischemia: therapeutic future or death of a brave concept? Eur J Pharmacol. 1999; **371**(1): 85-102.
1936. Von Lubitz DK. Adenosine and cerebral ischemia: therapeutic future or death of a brave concept? Eur J Pharmacol. 1999; **365**(1): 9-25.
1937. von Lubitz DK. Adenosine and cerebral ischemia: therapeutic future or death of a brave concept? European Journal Of Pharmacology. 1999; **371**(1): 85-102.
1938. Von Lubitz DK. Adenosine and cerebral ischemia: therapeutic future or death of a brave concept? European Journal Of Pharmacology. 1999; **365**(1): 9-25.
1939. von Muhlen D, Barrett-Connor E, Kritz-Silverstein D. Apolipoprotein E genotype and response of lipid levels to postmenopausal estrogen use. Atherosclerosis. 2002; **161**(1): 209-14.
1940. von Muhlen D, Barrett-Connor E, Kritz-Silverstein D. Apolipoprotein E genotype and response of lipid levels to postmenopausal estrogen use. Atherosclerosis. 2002; **161**(1): 209-14.
1941. Voora D, Shah SH, Spasojevic I, Ali S, Reed CR, Salisbury BA, et al. The SLCO1B1*5 genetic variant is associated with statin-induced side effects. J Am Coll Cardiol. 2009; **54**(17): 1609-16.
1942. Voora D, Shah SH, Spasojevic I, Ali S, Reed CR, Salisbury BA, et al. The SLCO1B1*5 genetic variant is associated with statin-induced side effects. Journal Of The American College Of Cardiology. 2009; **54**(17): 1609-16.
1943. Wagoner LE, Craft LL, Zengel P, McGuire N, Rathz DA, Dorn GW, 2nd, et al. Polymorphisms of the beta1-adrenergic receptor predict exercise capacity in heart failure. Am Heart J. 2002; **144**(5): 840-6.
1944. Wagoner LE, Craft LL, Zengel P, McGuire N, Rathz DA, Dorn GW, 2nd, et al. Polymorphisms of the beta1-adrenergic receptor predict exercise capacity in heart failure. American Heart Journal. 2002; **144**(5): 840-6.
1945. Wahrmann M, Dohler B, Ruhenstroth A, Haslacher H, Perkmann T, Exner M, et al. Genotypic diversity of complement component C4 does not predict kidney transplant outcome. J Am Soc Nephrol. 2011; **22**(2): 367-76.
1946. Wahrmann M, Döhler B, Ruhenstroth A, Haslacher H, Perkmann T, Exner M, et al. Genotypic diversity of complement component C4 does not predict kidney transplant outcome. Journal Of The American Society Of Nephrology: JASN. 2011; **22**(2): 367-76.
1947. Waisbren SE, Potter NL, Gordon CM, Green RC, Greenstein P, Gubbels CS, et

al. The adult galactosemic phenotype. J Inherit Metab Dis. 2012; **35**(2): 279-86.
1948. Waisbren SE, Potter NL, Gordon CM, Green RC, Greenstein P, Gubbels CS, et al. The adult galactosemic phenotype. Journal Of Inherited Metabolic Disease. 2012; **35**(2): 279-86.
1949. Walia GK, Gupta V, Aggarwal A, Asghar M, Dudbridge F, Timpson N, et al. Association of common genetic variants with lipid traits in the Indian population. PLoS One. 2014; **9**(7): e101688.
1950. Walia GK, Gupta V, Aggarwal A, Asghar M, Dudbridge F, Timpson N, et al. Association of common genetic variants with lipid traits in the Indian population. PLoS One. 2014; **9**(7): e101688-e.
1951. Wang C, Wang B, He H, Li X, Wei D, Zhang J, et al. Association between insulin receptor gene polymorphism and the metabolic syndrome in Han and Yi Chinese. Asia Pac J Clin Nutr. 2012; **21**(3): 457-63.
1952. Wang C, Wang B, He H, Li X, Wei D, Zhang J, et al. Association between insulin receptor gene polymorphism and the metabolic syndrome in Han and Yi Chinese. Asia Pacific Journal Of Clinical Nutrition. 2012; **21**(3): 457-63.
1953. Wang HX, Jin Y, Hendrie HC, Liang C, Yang L, Cheng Y, et al. Late life leisure activities and risk of cognitive decline. J Gerontol A Biol Sci Med Sci. 2013; **68**(2): 205-13.
1954. Wang H-X, Jin Y, Hendrie HC, Liang C, Yang L, Cheng Y, et al. Late life leisure activities and risk of cognitive decline. The Journals Of Gerontology Series A, Biological Sciences And Medical Sciences. 2013; **68**(2): 205-13.
1955. Wang P, Lin XQ, Cai WK, Xu GL, Zhou MD, Yang M, et al. Effect of UGT2B7 genotypes on plasma concentration of valproic acid: a meta-analysis. Eur J Clin Pharmacol. 2018; **74**(4): 433-42.
1956. Wang P, Lin X-Q, Cai W-K, Xu G-L, Zhou M-D, Yang M, et al. Effect of UGT2B7 genotypes on plasma concentration of valproic acid: a meta-analysis. European Journal Of Clinical Pharmacology. 2018; **74**(4): 433-42.
1957. Wang PW, Chen IY, Juo SH, Hsi E, Liu RT, Hsieh CJ. Genotype and phenotype predictors of relapse of graves' disease after antithyroid drug withdrawal. Eur Thyroid J. 2013; **1**(4): 251-8.
1958. Wang P-W, Chen IY, Juo S-HH, Hsi E, Liu R-T, Hsieh C-J. Genotype and phenotype predictors of relapse of graves' disease after antithyroid drug withdrawal. European Thyroid Journal. 2013; **1**(4): 251-8.
1959. Wang RS, Hall KT, Giulianini F, Passow D, Kaptchuk TJ, Loscalzo J. Network analysis of the genomic basis of the placebo effect. JCI Insight. 2017; **2**(11).
1960. Wang R-S, Hall KT, Giulianini F, Passow D, Kaptchuk TJ, Loscalzo J. Network analysis of the genomic basis of the placebo effect. JCI Insight. 2017; **2**(11).
1961. Wang X, Feng Q, Xiao Y, Li P. Radix Bupleuri ameliorates depression by increasing nerve growth factor and brain-derived neurotrophic factor. Int J Clin Exp Med. 2015; **8**(6): 9205-17.
1962. Wang X, Feng Q, Xiao Y, Li P. Radix Bupleuri ameliorates depression by increasing nerve growth factor and brain-derived neurotrophic factor. International Journal Of Clinical And Experimental Medicine. 2015; **8**(6): 9205-17.
1963. Wang Y, Li X, Qin X, Cai Y, He M, Sun L, et al. Prevalence of

hyperhomocysteinaemia and its major determinants in rural Chinese hypertensive patients aged 45-75 years. Br J Nutr. 2013; **109**(7): 1284-93.
1964. Wang Y, Li X, Qin X, Cai Y, He M, Sun L, et al. Prevalence of hyperhomocysteinaemia and its major determinants in rural Chinese hypertensive patients aged 45-75 years. The British Journal Of Nutrition. 2013; **109**(7): 1284-93.
1965. Wang YS, Lee SY, Chen SL, Chang YH, Wang TY, Lin SH, et al. Role of DRD2 and ALDH2 genes in bipolar II disorder with and without comorbid anxiety disorder. Eur Psychiatry. 2014; **29**(3): 142-8.
1966. Wang YS, Lee SY, Chen SL, Chang YH, Wang TY, Lin SH, et al. Role of DRD2 and ALDH2 genes in bipolar II disorder with and without comorbid anxiety disorder. European Psychiatry: The Journal Of The Association Of European Psychiatrists. 2014; **29**(3): 142-8.
1967. Wang ZY, Chen M, Zhu LL, Yu LS, Zeng S, Xiang MX, et al. Pharmacokinetic drug interactions with clopidogrel: updated review and risk management in combination therapy. Ther Clin Risk Manag. 2015; **11**: 449-67.
1968. Wang Z-Y, Chen M, Zhu L-L, Yu L-S, Zeng S, Xiang M-X, et al. Pharmacokinetic drug interactions with clopidogrel: updated review and risk management in combination therapy. Therapeutics And Clinical Risk Management. 2015; **11**: 449-67.
1969. Ward H, Mitrou PN, Bowman R, Luben R, Wareham NJ, Khaw KT, et al. APOE genotype, lipids, and coronary heart disease risk: a prospective population study. Arch Intern Med. 2009; **169**(15): 1424-9.
1970. Ward H, Mitrou PN, Bowman R, Luben R, Wareham NJ, Khaw K-T, et al. APOE genotype, lipids, and coronary heart disease risk: a prospective population study. Archives Of Internal Medicine. 2009; **169**(15): 1424-9.
1971. Ward JL, Baker JM, Miller SJ, Deborde C, Maucourt M, Biais B, et al. An inter-laboratory comparison demonstrates that [H]-NMR metabolite fingerprinting is a robust technique for collaborative plant metabolomic data collection. Metabolomics. 2010; **6**(2): 263-73.
1972. Ward JL, Baker JM, Miller SJ, Deborde C, Maucourt M, Biais B, et al. An inter-laboratory comparison demonstrates that [H]-NMR metabolite fingerprinting is a robust technique for collaborative plant metabolomic data collection. Metabolomics: Official Journal Of The Metabolomic Society. 2010; **6**(2): 263-73.
1973. Wardle J, Llewellyn C, Sanderson S, Plomin R. The FTO gene and measured food intake in children. Int J Obes (Lond). 2009; **33**(1): 42-5.
1974. Wardle J, Llewellyn C, Sanderson S, Plomin R. The FTO gene and measured food intake in children. International Journal Of Obesity (2005). 2009; **33**(1): 42-5.
1975. Wareham NJ, Young EH, Loos RJ. Epidemiological study designs to investigate gene-behavior interactions in the context of human obesity. Obesity (Silver Spring). 2008; **16 Suppl 3**: S66-71.
1976. Wareham NJ, Young EH, Loos RJF. Epidemiological study designs to investigate gene-behavior interactions in the context of human obesity. Obesity (Silver Spring, Md). 2008; **16 Suppl 3**: S66-S71.
1977. Wark PA, Grubben MJ, Peters WH, Nagengast FM, Kampman E, Kok FJ, et al. Habitual consumption of fruits and vegetables: associations with human rectal glutathione S-transferase. Carcinogenesis. 2004; **25**(11): 2135-42.

1978. Wark PA, Grubben MJAL, Peters WHM, Nagengast FM, Kampman E, Kok FJ, et al. Habitual consumption of fruits and vegetables: associations with human rectal glutathione S-transferase. Carcinogenesis. 2004; **25**(11): 2135-42.
1979. Warwick JM, Carey PD, Cassimjee N, Lochner C, Hemmings S, Moolman-Smook H, et al. Dopamine transporter binding in social anxiety disorder: the effect of treatment with escitalopram. Metab Brain Dis. 2012; **27**(2): 151-8.
1980. Warwick JM, Carey PD, Cassimjee N, Lochner C, Hemmings S, Moolman-Smook H, et al. Dopamine transporter binding in social anxiety disorder: the effect of treatment with escitalopram. Metabolic Brain Disease. 2012; **27**(2): 151-8.
1981. Waschbisch A, Wenny I, Tallner A, Schwab S, Pfeifer K, Maurer M. Physical activity in multiple sclerosis: a comparative study of vitamin D, brain-derived neurotrophic factor and regulatory T cell populations. Eur Neurol. 2012; **68**(2): 122-8.
1982. Waschbisch A, Wenny I, Tallner A, Schwab S, Pfeifer K, Mäurer M. Physical activity in multiple sclerosis: a comparative study of vitamin D, brain-derived neurotrophic factor and regulatory T cell populations. European Neurology. 2012; **68**(2): 122-8.
1983. Wasserman L, Flatt SW, Natarajan L, Laughlin G, Matusalem M, Faerber S, et al. Correlates of obesity in postmenopausal women with breast cancer: comparison of genetic, demographic, disease-related, life history and dietary factors. Int J Obes Relat Metab Disord. 2004; **28**(1): 49-56.
1984. Wasserman L, Flatt SW, Natarajan L, Laughlin G, Matusalem M, Faerber S, et al. Correlates of obesity in postmenopausal women with breast cancer: comparison of genetic, demographic, disease-related, life history and dietary factors. International Journal Of Obesity And Related Metabolic Disorders: Journal Of The International Association For The Study Of Obesity. 2004; **28**(1): 49-56.
1985. Watanabe Y, Tsujimura A, Takao K, Nishi K, Ito Y, Yasuhara Y, et al. Relaxin-3-deficient mice showed slight alteration in anxiety-related behavior. Front Behav Neurosci. 2011; **5**: 50.
1986. Watanabe Y, Tsujimura A, Takao K, Nishi K, Ito Y, Yasuhara Y, et al. Relaxin-3-deficient mice showed slight alteration in anxiety-related behavior. Frontiers In Behavioral Neuroscience. 2011; **5**: 50-.
1987. Watenpaugh DE. The role of sleep dysfunction in physical inactivity and its relationship to obesity. Curr Sports Med Rep. 2009; **8**(6): 331-8.
1988. Watenpaugh DE. The role of sleep dysfunction in physical inactivity and its relationship to obesity. Current Sports Medicine Reports. 2009; **8**(6): 331-8.
1989. Weaver Cargin J, Collie A, Masters C, Maruff P. The nature of cognitive complaints in healthy older adults with and without objective memory decline. J Clin Exp Neuropsychol. 2008; **30**(2): 245-57.
1990. Weaver Cargin J, Collie A, Masters C, Maruff P. The nature of cognitive complaints in healthy older adults with and without objective memory decline. Journal Of Clinical And Experimental Neuropsychology. 2008; **30**(2): 245-57.
1991. Weinstein G, Preis SR, Beiser AS, Kaess B, Chen TC, Satizabal C, et al. Clinical and Environmental Correlates of Serum BDNF: A Descriptive Study with Plausible Implications for AD Research. Curr Alzheimer Res. 2017; **14**(7): 722-30.
1992. Weinstein G, Preis SR, Beiser AS, Kaess B, Chen TC, Satizabal C, et al. Clinical

and Environmental Correlates of Serum BDNF: A Descriptive Study with Plausible Implications for AD Research. Current Alzheimer Research. 2017; **14**(7): 722-30.
1993. Weizman A, Weizman R. Serotonin transporter polymorphism and response to SSRIs in major depression and relevance to anxiety disorders and substance abuse. Pharmacogenomics. 2000; **1**(3): 335-41.
1994. Weizman A, Weizman R. Serotonin transporter polymorphism and response to SSRIs in major depression and relevance to anxiety disorders and substance abuse. Pharmacogenomics. 2000; **1**(3): 335-41.
1995. Westman JO, Mapelli V, Taherzadeh MJ, Franzen CJ. Flocculation causes inhibitor tolerance in Saccharomyces cerevisiae for second-generation bioethanol production. Appl Environ Microbiol. 2014; **80**(22): 6908-18.
1996. Westman JO, Mapelli V, Taherzadeh MJ, Franzén CJ. Flocculation causes inhibitor tolerance in Saccharomyces cerevisiae for second-generation bioethanol production. Applied And Environmental Microbiology. 2014; **80**(22): 6908-18.
1997. Whittle N, Singewald N. HDAC inhibitors as cognitive enhancers in fear, anxiety and trauma therapy: where do we stand? Biochem Soc Trans. 2014; **42**(2): 569-81.
1998. Whittle N, Singewald N. HDAC inhibitors as cognitive enhancers in fear, anxiety and trauma therapy: where do we stand? Biochemical Society Transactions. 2014; **42**(2): 569-81.
1999. Wilhelm FH, Roth WT. Using minute ventilation for ambulatory estimation of additional heart rate. Biol Psychol. 1998; **49**(1-2): 137-50.
2000. Wilhelm FH, Roth WT. Using minute ventilation for ambulatory estimation of additional heart rate. Biological Psychology. 1998; **49**(1-2): 137-50.
2001. Wimmerova S, van den Berg M, Chovancova J, Patayova H, Jusko TA, van Duursen MB, et al. Relative effect potency estimates of dioxin-like activity for dioxins, furans, and dioxin-like PCBs in adults based on cytochrome P450 1A1 and 1B1 gene expression in blood. Environ Int. 2016; **96**: 24-33.
2002. Wimmerová S, van den Berg M, Chovancová J, Patayová H, Jusko TA, van Duursen MBM, et al. Relative effect potency estimates of dioxin-like activity for dioxins, furans, and dioxin-like PCBs in adults based on cytochrome P450 1A1 and 1B1 gene expression in blood. Environment International. 2016; **96**: 24-33.
2003. Winner J, Allen JD, Altar CA, Spahic-Mihajlovic A. Psychiatric pharmacogenomics predicts health resource utilization of outpatients with anxiety and depression. Transl Psychiatry. 2013; **3**: e242.
2004. Winner J, Allen JD, Altar CA, Spahic-Mihajlovic A. Psychiatric pharmacogenomics predicts health resource utilization of outpatients with anxiety and depression. Translational Psychiatry. 2013; **3**: e242-e.
2005. Wojcik SM, Tantra M, Stepniak B, Man KN, Muller-Ribbe K, Begemann M, et al. Genetic markers of a Munc13 protein family member, BAIAP3, are gender specifically associated with anxiety and benzodiazepine abuse in mice and humans. Mol Med. 2013; **19**: 135-48.
2006. Wojcik SM, Tantra M, Stepniak B, Man K-NM, Müller-Ribbe K, Begemann M, et al. Genetic markers of a Munc13 protein family member, BAIAP3, are gender specifically associated with anxiety and benzodiazepine abuse in mice and humans. Molecular Medicine (Cambridge, Mass). 2013; **19**: 135-48.

2007. Wolfgram PM, Carrel AL, Allen DB. Long-term effects of recombinant human growth hormone therapy in children with Prader-Willi syndrome. Curr Opin Pediatr. 2013; **25**(4): 509-14.
2008. Wolfgram PM, Carrel AL, Allen DB. Long-term effects of recombinant human growth hormone therapy in children with Prader-Willi syndrome. Current Opinion In Pediatrics. 2013; **25**(4): 509-14.
2009. Womack CJ, Saunders MJ, Bechtel MK, Bolton DJ, Martin M, Luden ND, et al. The influence of a CYP1A2 polymorphism on the ergogenic effects of caffeine. J Int Soc Sports Nutr. 2012; **9**(1): 7.
2010. Wong ML, Dong C, Flores DL, Ehrhart-Bornstein M, Bornstein S, Arcos-Burgos M, et al. Clinical outcomes and genome-wide association for a brain methylation site in an antidepressant pharmacogenetics study in Mexican Americans. Am J Psychiatry. 2014; **171**(12): 1297-309.
2011. Wong M-L, Dong C, Flores DL, Ehrhart-Bornstein M, Bornstein S, Arcos-Burgos M, et al. Clinical outcomes and genome-wide association for a brain methylation site in an antidepressant pharmacogenetics study in Mexican Americans. The American Journal Of Psychiatry. 2014; **171**(12): 1297-309.
2012. Woo J, Tang NL, Leung J, Kwok T. The Alu polymorphism of angiotensin I converting enzyme (ACE) and atherosclerosis, incident chronic diseases and mortality in an elderly Chinese population. J Nutr Health Aging. 2012; **16**(3): 262-8.
2013. Woo J, Tang NLS, Leung J, Kwok T. The Alu polymorphism of angiotensin I converting enzyme (ACE) and atherosclerosis, incident chronic diseases and mortality in an elderly Chinese population. The Journal Of Nutrition, Health & Aging. 2012; **16**(3): 262-8.
2014. Woo JM, Yoon KS, Choi YH, Oh KS, Lee YS, Yu BH. The association between panic disorder and the L/L genotype of catechol-O-methyltransferase. J Psychiatr Res. 2004; **38**(4): 365-70.
2015. Woo J-M, Yoon K-S, Choi Y-H, Oh K-S, Lee Y-S, Yu B-H. The association between panic disorder and the L/L genotype of catechol-O-methyltransferase. Journal Of Psychiatric Research. 2004; **38**(4): 365-70.
2016. Wood NI, Carta V, Milde S, Skillings EA, McAllister CJ, Ang YL, et al. Responses to environmental enrichment differ with sex and genotype in a transgenic mouse model of Huntington's disease. PLoS One. 2010; **5**(2): e9077.
2017. Wood NI, Carta V, Milde S, Skillings EA, McAllister CJ, Ang YLM, et al. Responses to environmental enrichment differ with sex and genotype in a transgenic mouse model of Huntington's disease. PLoS One. 2010; **5**(2): e9077-e.
2018. Wu BW, Skidmore PM, Orta OR, Faulkner J, Lambrick D, Signal L, et al. Genotype vs. Phenotype and the Rise of Non-Communicable Diseases: The Importance of Lifestyle Behaviors During Childhood. Cureus. 2016; **8**(1): e458.
2019. Wu BW, Skidmore PM, Orta OR, Faulkner J, Lambrick D, Signal L, et al. Genotype vs. Phenotype and the Rise of Non-Communicable Diseases: The Importance of Lifestyle Behaviors During Childhood. Cureus. 2016; **8**(1): e458-e.
2020. Wu JJ, Cui Y, Yang YS, Kang MS, Jung SC, Park HK, et al. Modulatory effects of aromatherapy massage intervention on electroencephalogram, psychological assessments, salivary cortisol and plasma brain-derived neurotrophic factor.

Complement Ther Med. 2014; **22**(3): 456-62.
2021. Wu J-J, Cui Y, Yang Y-S, Kang M-S, Jung S-C, Park HK, et al. Modulatory effects of aromatherapy massage intervention on electroencephalogram, psychological assessments, salivary cortisol and plasma brain-derived neurotrophic factor. Complementary Therapies In Medicine. 2014; **22**(3): 456-62.
2022. Wu SL, Leung AW, Yew DT. Acupuncture for Detoxification in Treatment of Opioid Addiction. East Asian Arch Psychiatry. 2016; **26**(2): 70-6.
2023. Wu SL, Leung AW, Yew DT. Acupuncture for Detoxification in Treatment of Opioid Addiction. East Asian Archives Of Psychiatry: Official Journal Of The Hong Kong College Of Psychiatrists = Dong Ya Jing Shen Ke Xue Zhi: Xianggang Jing Shen Ke Yi Xue Yuan Qi Kan. 2016; **26**(2): 70-6.
2024. Wyce A, Bai Y, Nagpal S, Thompson CC. Research Resource: The androgen receptor modulates expression of genes with critical roles in muscle development and function. Mol Endocrinol. 2010; **24**(8): 1665-74.
2025. Wyce A, Bai Y, Nagpal S, Thompson CC. Research Resource: The androgen receptor modulates expression of genes with critical roles in muscle development and function. Molecular Endocrinology (Baltimore, Md). 2010; **24**(8): 1665-74.
2026. Xiao L, Correll CU, Feng L, Xiang YT, Feng Y, Hu CQ, et al. Rhythmic low-field magnetic stimulation may improve depression by increasing brain-derived neurotrophic factor. CNS Spectr. 2018: 1-9.
2027. Xiao L, Correll CU, Feng L, Xiang Y-T, Feng Y, Hu C-Q, et al. Rhythmic low-field magnetic stimulation may improve depression by increasing brain-derived neurotrophic factor. CNS Spectrums. 2018: 1-9.
2028. Xu X, Gammon MD, Wetmur JG, Rao M, Gaudet MM, Teitelbaum SL, et al. A functional 19-base pair deletion polymorphism of dihydrofolate reductase (DHFR) and risk of breast cancer in multivitamin users. Am J Clin Nutr. 2007; **85**(4): 1098-102.
2029. Xu X, Gammon MD, Wetmur JG, Rao M, Gaudet MM, Teitelbaum SL, et al. A functional 19-base pair deletion polymorphism of dihydrofolate reductase (DHFR) and risk of breast cancer in multivitamin users. The American Journal Of Clinical Nutrition. 2007; **85**(4): 1098-102.
2030. Yajnik CS. Early life origins of insulin resistance and type 2 diabetes in India and other Asian countries. J Nutr. 2004; **134**(1): 205-10.
2031. Yajnik CS. Early life origins of insulin resistance and type 2 diabetes in India and other Asian countries. The Journal Of Nutrition. 2004; **134**(1): 205-10.
2032. Yan Z, Jiao F, Yan X, Ou H. Maternal Chronic Folate Supplementation Ameliorates Behavior Disorders Induced by Prenatal High-Fat Diet Through Methylation Alteration of BDNF and Grin2b in Offspring Hippocampus. Mol Nutr Food Res. 2017; **61**(12).
2033. Yan Z, Jiao F, Yan X, Ou H. Maternal Chronic Folate Supplementation Ameliorates Behavior Disorders Induced by Prenatal High-Fat Diet Through Methylation Alteration of BDNF and Grin2b in Offspring Hippocampus. Molecular Nutrition & Food Research. 2017; **61**(12).
2034. Yang A, Palmer AA, de Wit H. Genetics of caffeine consumption and responses to caffeine. Psychopharmacology (Berl). 2010; **211**(3): 245-57.
2035. Yang A, Palmer AA, de Wit H. Genetics of caffeine consumption and responses

to caffeine. Psychopharmacology. 2010; **211**(3): 245-57.
2036. Yang M, Kang MJ, Choi Y, Kim CS, Lee SM, Park CW, et al. Associations between XPC expression, genotype, and the risk of head and neck cancer. Environ Mol Mutagen. 2005; **45**(4): 374-9.
2037. Yang W, Elankumaran S, Marr LC. Concentrations and size distributions of airborne influenza A viruses measured indoors at a health centre, a day-care centre and on aeroplanes. J R Soc Interface. 2011; **8**(61): 1176-84.
2038. Yang W, Elankumaran S, Marr LC. Concentrations and size distributions of airborne influenza A viruses measured indoors at a health centre, a day-care centre and on aeroplanes. Journal Of The Royal Society, Interface. 2011; **8**(61): 1176-84.
2039. Yang W, Kelly T, He J. Genetic epidemiology of obesity. Epidemiol Rev. 2007; **29**: 49-61.
2040. Yang W, Kelly T, He J. Genetic epidemiology of obesity. Epidemiologic Reviews. 2007; **29**: 49-61.
2041. Yasui-Furukori N, Saito M, Nakagami T, Kaneda A, Tateishi T, Kaneko S. Association between multidrug resistance 1 (MDR1) gene polymorphisms and therapeutic response to bromperidol in schizophrenic patients: a preliminary study. Prog Neuropsychopharmacol Biol Psychiatry. 2006; **30**(2): 286-91.
2042. Yasui-Furukori N, Saito M, Nakagami T, Kaneda A, Tateishi T, Kaneko S. Association between multidrug resistance 1 (MDR1) gene polymorphisms and therapeutic response to bromperidol in schizophrenic patients: a preliminary study. Progress In Neuro-Psychopharmacology & Biological Psychiatry. 2006; **30**(2): 286-91.
2043. Yevtushenko OO, Oros MM, Reynolds GP. Early response to selective serotonin reuptake inhibitors in panic disorder is associated with a functional 5-HT1A receptor gene polymorphism. J Affect Disord. 2010; **123**(1-3): 308-11.
2044. Yevtushenko OO, Oros MM, Reynolds GP. Early response to selective serotonin reuptake inhibitors in panic disorder is associated with a functional 5-HT1A receptor gene polymorphism. Journal Of Affective Disorders. 2010; **123**(1-3): 308-11.
2045. Yi Y, Dongmei L, Phares DA, Weiss EP, Brandauer J, Hagberg JM. Association between KCNJ11 E23K genotype and cardiovascular and glucose metabolism phenotypes in older men and women. Exp Physiol. 2008; **93**(1): 95-103.
2046. Yi Y, Dongmei L, Phares DA, Weiss EP, Brandauer J, Hagberg JM. Association between KCNJ11 E23K genotype and cardiovascular and glucose metabolism phenotypes in older men and women. Experimental Physiology. 2008; **93**(1): 95-103.
2047. Yokoyama JS, Bonham LW, Sturm VE, Adhimoolam B, Karydas A, Coppola G, et al. The 5-HTTLPR variant in the serotonin transporter gene modifies degeneration of brain regions important for emotion in behavioral variant frontotemporal dementia. Neuroimage Clin. 2015; **9**: 283-90.
2048. Yokoyama JS, Bonham LW, Sturm VE, Adhimoolam B, Karydas A, Coppola G, et al. The 5-HTTLPR variant in the serotonin transporter gene modifies degeneration of brain regions important for emotion in behavioral variant frontotemporal dementia. Neuroimage Clinical. 2015; **9**: 283-90.
2049. Yoon EJ, Goussard S, Touchon M, Krizova L, Cerqueira G, Murphy C, et al. Origin in Acinetobacter guillouiae and dissemination of the aminoglycoside-modifying enzyme Aph(3')-VI. MBio. 2014; **5**(5): e01972-14.

2050. Yoon E-J, Goussard S, Touchon M, Krizova L, Cerqueira G, Murphy C, et al. Origin in Acinetobacter guillouiae and dissemination of the aminoglycoside-modifying enzyme Aph(3')-VI. MBio. 2014; **5**(5): e01972-e14.
2051. Yoshimura R, Kishi T, Hori H, Atake K, Katsuki A, Nakano-Umene W, et al. Serum proBDNF/BDNF and response to fluvoxamine in drug-naive first-episode major depressive disorder patients. Ann Gen Psychiatry. 2014; **13**: 19.
2052. Young EE, Kelly DL, Shim I, Baumbauer KM, Starkweather A, Lyon DE. Variations in COMT and NTRK2 Influence Symptom Burden in Women Undergoing Breast Cancer Treatment. Biol Res Nurs. 2017; **19**(3): 318-28.
2053. Young EE, Kelly DL, Shim I, Baumbauer KM, Starkweather A, Lyon DE. Variations in COMT and NTRK2 Influence Symptom Burden in Women Undergoing Breast Cancer Treatment. Biological Research For Nursing. 2017; **19**(3): 318-28.
2054. Younossi ZM, Stepanova M, Balistreri W, Schwarz K, Murray KF, Rosenthal P, et al. Health-related Quality of Life in Adolescent Patients With Hepatitis C Genotype 1 Treated With Sofosbuvir and Ledipasvir. J Pediatr Gastroenterol Nutr. 2018; **66**(1): 112-6.
2055. Younossi ZM, Stepanova M, Balistreri W, Schwarz K, Murray KF, Rosenthal P, et al. Health-related Quality of Life in Adolescent Patients With Hepatitis C Genotype 1 Treated With Sofosbuvir and Ledipasvir. Journal Of Pediatric Gastroenterology And Nutrition. 2018; **66**(1): 112-6.
2056. Younossi ZM, Stepanova M, Charlton M, Curry MP, O'Leary JG, Brown RS, et al. Patient-reported outcomes with sofosbuvir and velpatasvir with or without ribavirin for hepatitis C virus-related decompensated cirrhosis: an exploratory analysis from the randomised, open-label ASTRAL-4 phase 3 trial. Lancet Gastroenterol Hepatol. 2016; **1**(2): 122-32.
2057. Younossi ZM, Stepanova M, Charlton M, Curry MP, O'Leary JG, Brown RS, et al. Patient-reported outcomes with sofosbuvir and velpatasvir with or without ribavirin for hepatitis C virus-related decompensated cirrhosis: an exploratory analysis from the randomised, open-label ASTRAL-4 phase 3 trial. The Lancet Gastroenterology & Hepatology. 2016; **1**(2): 122-32.
2058. Younossi ZM, Stepanova M, Sulkowski M, Wyles D, Kottilil S, Hunt S. Patient-reported outcomes in patients co-infected with hepatitis C virus and human immunodeficiency virus treated with sofosbuvir and velpatasvir: The ASTRAL-5 study. Liver Int. 2017; **37**(12): 1796-804.
2059. Younossi ZM, Stepanova M, Sulkowski M, Wyles D, Kottilil S, Hunt S. Patient-reported outcomes in patients co-infected with hepatitis C virus and human immunodeficiency virus treated with sofosbuvir and velpatasvir: The ASTRAL-5 study. Liver International: Official Journal Of The International Association For The Study Of The Liver. 2017; **37**(12): 1796-804.
2060. Youssef SS, Abbas EA, Abd el Aal AM, Omran MH, Barakat A, Seif SM. IL28B rs 12979860 predicts response to treatment in Egyptian hepatitis C virus genotype 4 patients and alpha fetoprotein increases its predictive strength. J Interferon Cytokine Res. 2014; **34**(7): 505-9.
2061. Youssef SS, Abbas EAE-R, Abd el Aal AM, Omran MH, Barakat A, Seif SM. IL28B rs 12979860 predicts response to treatment in Egyptian hepatitis C virus

genotype 4 patients and alpha fetoprotein increases its predictive strength. Journal Of Interferon & Cytokine Research: The Official Journal Of The International Society For Interferon And Cytokine Research. 2014; **34**(7): 505-9.
2062. Yun YH, Kim YA, Sim JA, Shin AS, Chang YJ, Lee J, et al. Prognostic value of quality of life score in disease-free survivors of surgically-treated lung cancer. BMC Cancer. 2016; **16**: 505.
2063. Yun YH, Kim YA, Sim JA, Shin AS, Chang YJ, Lee J, et al. Prognostic value of quality of life score in disease-free survivors of surgically-treated lung cancer. BMC Cancer. 2016; **16**: 505-.
2064. Zakavi M, Askari H, Irvani N. Optimizing micropropagation of drought resistant Pyrus boissieriana Buhse. Physiol Mol Biol Plants. 2016; **22**(4): 583-93.
2065. Zakavi M, Askari H, Irvani N. Optimizing micropropagation of drought resistant Pyrus boissieriana Buhse. Physiology And Molecular Biology Of Plants: An International Journal Of Functional Plant Biology. 2016; **22**(4): 583-93.
2066. Zandi R, van der Schoot P, Reguera D, Kegel W, Reiss H. Classical nucleation theory of virus capsids. Biophys J. 2006; **90**(6): 1939-48.
2067. Zandi R, van der Schoot P, Reguera D, Kegel W, Reiss H. Classical nucleation theory of virus capsids. Biophysical Journal. 2006; **90**(6): 1939-48.
2068. Zarcone D, Corbetta S. Shared mechanisms of epilepsy, migraine and affective disorders. Neurol Sci. 2017; **38**(Suppl 1): 73-6.
2069. Zarcone D, Corbetta S. Shared mechanisms of epilepsy, migraine and affective disorders. Neurological Sciences: Official Journal Of The Italian Neurological Society And Of The Italian Society Of Clinical Neurophysiology. 2017; **38**(Suppl 1): 73-6.
2070. Zempo H, Tanabe K, Murakami H, Iemitsu M, Maeda S, Kuno S. ACTN3 polymorphism affects thigh muscle area. Int J Sports Med. 2010; **31**(2): 138-42.
2071. Zempo H, Tanabe K, Murakami H, Iemitsu M, Maeda S, Kuno S. ACTN3 polymorphism affects thigh muscle area. International Journal Of Sports Medicine. 2010; **31**(2): 138-42.
2072. Zhang B, Zhou N, Liu YM, Liu C, Lou CB, Jiang CY, et al. Ribosome binding site libraries and pathway modules for shikimic acid synthesis with Corynebacterium glutamicum. Microb Cell Fact. 2015; **14**: 71.
2073. Zhang B, Zhou N, Liu Y-M, Liu C, Lou C-B, Jiang C-Y, et al. Ribosome binding site libraries and pathway modules for shikimic acid synthesis with Corynebacterium glutamicum. Microbial Cell Factories. 2015; **14**: 71-.
2074. Zhang C, Lopez-Ridaura R, Rimm EB, Rifai N, Hunter DJ, Hu FB. Interactions between the -514C->T polymorphism of the hepatic lipase gene and lifestyle factors in relation to HDL concentrations among US diabetic men. Am J Clin Nutr. 2005; **81**(6): 1429-35.
2075. Zhang C, Lopez-Ridaura R, Rimm EB, Rifai N, Hunter DJ, Hu FB. Interactions between the -514C->T polymorphism of the hepatic lipase gene and lifestyle factors in relation to HDL concentrations among US diabetic men. The American Journal Of Clinical Nutrition. 2005; **81**(6): 1429-35.
2076. Zhang K, Jiang H, Zhang Q, Du J, Wang Y, Zhao M. Brain-derived neurotrophic factor serum levels in heroin-dependent patients after 26weeks of withdrawal. Compr Psychiatry. 2016; **65**: 150-5.

2077. Zhang K, Jiang H, Zhang Q, Du J, Wang Y, Zhao M. Brain-derived neurotrophic factor serum levels in heroin-dependent patients after 26weeks of withdrawal. Comprehensive Psychiatry. 2016; **65**: 150-5.
2078. Zhang X, Yin JF, Zhang J, Kong SJ, Zhang HY, Chen XM. UGT1A1*6 polymorphisms are correlated with irinotecan-induced neutropenia: a systematic review and meta-analysis. Cancer Chemother Pharmacol. 2017; **80**(1): 135-49.
2079. Zhang X, Yin J-F, Zhang J, Kong S-J, Zhang H-Y, Chen X-M. UGT1A1*6 polymorphisms are correlated with irinotecan-induced neutropenia: a systematic review and meta-analysis. Cancer Chemotherapy And Pharmacology. 2017; **80**(1): 135-49.
2080. Zhang Y, Gu N, Miao L, Yuan H, Wang R, Jiang H. Alcohol dehydrogenase-1B Arg47His polymorphism is associated with head and neck cancer risk in Asian: a meta-analysis. Tumour Biol. 2015; **36**(2): 1023-7.
2081. Zhang Y, Gu N, Miao L, Yuan H, Wang R, Jiang H. Alcohol dehydrogenase-1B Arg47His polymorphism is associated with head and neck cancer risk in Asian: a meta-analysis. Tumour Biology: The Journal Of The International Society For Oncodevelopmental Biology And Medicine. 2015; **36**(2): 1023-7.
2082. Zhang Z, Qiu S, Zhang X, Chen W. Optimized DNA electroporation for primary human T cell engineering. BMC Biotechnol. 2018; **18**(1): 4.
2083. Zhang Z, Qiu S, Zhang X, Chen W. Optimized DNA electroporation for primary human T cell engineering. BMC Biotechnology. 2018; **18**(1): 4-.
2084. Zhao H, Liang B, Yu L, Xu Y. Anti-depressant-like effects of Jieyu chufan capsules in a mouse model of unpredictable chronic mild stress. Exp Ther Med. 2017; **14**(2): 1086-94.
2085. Zhao H, Liang B, Yu L, Xu Y. Anti-depressant-like effects of Jieyu chufan capsules in a mouse model of unpredictable chronic mild stress. Experimental And Therapeutic Medicine. 2017; **14**(2): 1086-94.
2086. Zhao J, Goldberg J, Bremner JD, Vaccarino V. Association between promoter methylation of serotonin transporter gene and depressive symptoms: a monozygotic twin study. Psychosom Med. 2013; **75**(6): 523-9.
2087. Zhao J, Goldberg J, Bremner JD, Vaccarino V. Association between promoter methylation of serotonin transporter gene and depressive symptoms: a monozygotic twin study. Psychosomatic Medicine. 2013; **75**(6): 523-9.
2088. Zhao Y, Liu W, Hua M, Shi R, Wang H, Yang W. Relationship between CATSPERB, NR5A2 gene polymorphisms and Peak Bone Mineral Density in College Students in China. Iran J Public Health. 2014; **43**(8): 1060-9.
2089. Zhao Y, Liu W, Hua M, Shi R, Wang H, Yang W. Relationship between CATSPERB, NR5A2 gene polymorphisms and Peak Bone Mineral Density in College Students in China. Iranian Journal Of Public Health. 2014; **43**(8): 1060-9.
2090. Zheng Z, Zeng Y, Yang W, Wu J. Irritable bowel syndrome may be induced by decreased neuroplasticity. Neuro Endocrinol Lett. 2014; **35**(8): 655-65.
2091. Zheng Z, Zeng Y, Yang W, Wu J. Irritable bowel syndrome may be induced by decreased neuroplasticity. Neuro Endocrinology Letters. 2014; **35**(8): 655-65.
2092. Zhivolupov SA, Samartsev IN, Marchenko AA, Puliatkina OV. [The prognostic significance of brain-derived neurotrophic factor (BDNF) for phobic anxiety disorders, vegetative and cognitive impairments during conservative treatment including adaptol of

some functional and organic diseases of nervous system]. Zh Nevrol Psikhiatr Im S S Korsakova. 2012; **112**(4): 37-41.
2093. Zhivolupov SA, Samartsev IN, Marchenko AA, Puliatkina OV. [The prognostic significance of brain-derived neurotrophic factor (BDNF) for phobic anxiety disorders, vegetative and cognitive impairments during conservative treatment including adaptol of some functional and organic diseases of nervous system]. Zhurnal Nevrologii I Psikhiatrii Imeni SS Korsakova. 2012; **112**(4): 37-41.
2094. Zhivolupov SA, Samartsev IN, Marchenko AA, Puljatkina OV. [Neurophysiological monitoring of the efficacy of the complex therapy of vascular and posttraumatic encephalopathy]. Zh Nevrol Psikhiatr Im S S Korsakova. 2013; **113**(4): 25-9.
2095. Zhivolupov SA, Samartsev IN, Marchenko AA, Puljatkina OV. [Neurophysiological monitoring of the efficacy of the complex therapy of vascular and posttraumatic encephalopathy]. Zhurnal Nevrologii I Psikhiatrii Imeni SS Korsakova. 2013; **113**(4): 25-9.
2096. Zhou D, Sunzel M, Ribadeneira MD, Smith MA, Desai D, Lin J, et al. A clinical study to assess CYP1A2 and CYP3A4 induction by AZD7325, a selective GABA(A) receptor modulator - an in vitro and in vivo comparison. Br J Clin Pharmacol. 2012; **74**(1): 98-108.
2097. Zhou D, Sunzel M, Ribadeneira MD, Smith MA, Desai D, Lin J, et al. A clinical study to assess CYP1A2 and CYP3A4 induction by AZD7325, a selective GABA(A) receptor modulator - an in vitro and in vivo comparison. British Journal Of Clinical Pharmacology. 2012; **74**(1): 98-108.
2098. Zhou S, Kestell P, Baguley BC, Paxton JW. 5,6-dimethylxanthenone-4-acetic acid (DMXAA): a new biological response modifier for cancer therapy. Invest New Drugs. 2002; **20**(3): 281-95.
2099. Zhou S, Kestell P, Baguley BC, Paxton JW. 5,6-dimethylxanthenone-4-acetic acid (DMXAA): a new biological response modifier for cancer therapy. Investigational New Drugs. 2002; **20**(3): 281-95.
2100. Zhu QQ, Zhang XL, Zhang SM, Tang SW, Min HY, Yi L, et al. Association Between the MUC5B Promoter Polymorphism rs35705950 and Idiopathic Pulmonary Fibrosis: A Meta-analysis and Trial Sequential Analysis in Caucasian and Asian Populations. Medicine (Baltimore). 2015; **94**(43): e1901.
2101. Zhu Q-Q, Zhang X-L, Zhang S-M, Tang S-W, Min H-Y, Yi L, et al. Association Between the MUC5B Promoter Polymorphism rs35705950 and Idiopathic Pulmonary Fibrosis: A Meta-analysis and Trial Sequential Analysis in Caucasian and Asian Populations. Medicine. 2015; **94**(43): e1901-e.
2102. Zill P, Preuss UW, Koller G, Bondy B, Soyka M. Lack of association between SNPs in the NEUROD2 gene and alcohol dependence in a German patient sample. Psychiatry Res. 2011; **187**(1-2): 220-3.
2103. Zill P, Preuss UW, Koller G, Bondy B, Soyka M. Lack of association between SNPs in the NEUROD2 gene and alcohol dependence in a German patient sample. Psychiatry Research. 2011; **187**(1-2): 220-3.
2104. Zink M. Comorbid Obsessive-Compulsive Symptoms in Schizophrenia: Insight into Pathomechanisms Facilitates Treatment. Adv Med. 2014; **2014**: 317980.
2105. Zink M. Comorbid Obsessive-Compulsive Symptoms in Schizophrenia: Insight

into Pathomechanisms Facilitates Treatment. Advances In Medicine. 2014; **2014**: 317980-.
2106. Zlatohlavek L, Hubacek JA, Vrablik M, Pejsova H, Lanska V, Ceska R. The Impact of Physical Activity and Dietary Measures on the Biochemical and Anthropometric Parameters in Obese Children. Is There Any Genetic Predisposition? Cent Eur J Public Health. 2015; **23 Suppl**: S62-6.
2107. Zlatohlávek L, Hubáček JA, Vrablík M, Pejšová H, Lánská V, Češka R. The Impact of Physical Activity and Dietary Measures on the Biochemical and Anthropometric Parameters in Obese Children. Is There Any Genetic Predisposition? Central European Journal Of Public Health. 2015; **23 Suppl**: S62-S6.
2108. Zmuda JM, Cauley JA, Ferrell RE. Recent progress in understanding the genetic susceptibility to osteoporosis. Genet Epidemiol. 1999; **16**(4): 356-67.
2109. Zmuda JM, Cauley JA, Ferrell RE. Recent progress in understanding the genetic susceptibility to osteoporosis. Genetic Epidemiology. 1999; **16**(4): 356-67.
2110. Zourkova A, Slanar O, Jarkovsky J, Palcikova I, Pindurova E, Cvanova M. MDR1 in paroxetine-induced sexual dysfunction. J Sex Marital Ther. 2013; **39**(1): 71-8.
2111. Zourková A, Slanař O, Jarkovský J, Palčíková I, Pindurová E, Cvanová M. MDR1 in paroxetine-induced sexual dysfunction. Journal Of Sex & Marital Therapy. 2013; **39**(1): 71-8.
2112. Zsuga J, Tajti G, Papp C, Juhasz B, Gesztelyi R. FNDC5/irisin, a molecular target for boosting reward-related learning and motivation. Med Hypotheses. 2016; **90**: 23-8.
2113. Zsuga J, Tajti G, Papp C, Juhasz B, Gesztelyi R. FNDC5/irisin, a molecular target for boosting reward-related learning and motivation. Medical Hypotheses. 2016; **90**: 23-8.
2114. Zwart MP, Daros JA, Elena SF. One is enough: in vivo effective population size is dose-dependent for a plant RNA virus. PLoS Pathog. 2011; **7**(7): e1002122.
2115. Zwart MP, Daròs J-A, Elena SF. One is enough: in vivo effective population size is dose-dependent for a plant RNA virus. Plos Pathogens. 2011; **7**(7): e1002122-e.
2116. Prospective association of a genetic risk score and lifestyle intervention with cardiovascular morbidity and mortality among individuals with type 2 diabetes: the Look AHEAD randomised controlled trial. Diabetologia. 2015; **58**(8): 1803-13.
2117. Adami A, Hobbs BD, McDonald M-L, Casaburi R, Rossiter HB. Genetic variants predicting aerobic capacity response to training are also associated with skeletal muscle oxidative capacity in moderate-to-severe COPD. Physiological Genomics. 2018.
2118. Adams D, Coelho T, Obici L, Merlini G, Mincheva Z, Suanprasert N, et al. Rapid progression of familial amyloidotic polyneuropathy: a multinational natural history study. Neurology. 2015; **85**(8): 675-82.
2119. Agrawal R, Tyagi E, Vergnes L, Reue K, Gomez-Pinilla F. Coupling energy homeostasis with a mechanism to support plasticity in brain trauma. Biochimica Et Biophysica Acta. 2014; **1842**(4): 535-46.
2120. Ahammad SZ, Bereslawski JL, Dolfing J, Mota C, Graham DW. Anaerobic-aerobic sequencing bioreactors improve energy efficiency for treatment of personal care product industry wastes. Bioresource Technology. 2013; **139**: 73-9.
2121. Ahola S, Auranen M, Isohanni P, Niemisalo S, Urho N, Buzkova J, et al. Modified Atkins diet induces subacute selective ragged-red-fiber lysis in mitochondrial

myopathy patients. EMBO Molecular Medicine. 2016; **8**(11): 1234-47.
2122. Akhmetov II, Popov DV, Missina SS, Vinogradova OL, Rogozkin VA. [Association of the mitochondrial transcription factor (TFAM) gene polymorphism with physical performance of athletes]. Fiziologiia Cheloveka. 2010; **36**(2): 121-5.
2123. Akimova E, Lanzenberger R, Kasper S. The serotonin-1A receptor in anxiety disorders. Biological Psychiatry. 2009; **66**(7): 627-35.
2124. Alam I, Sun Q, Liu L, Koller DL, Liu Y, Edenberg HJ, et al. Genomic expression analysis of rat chromosome 4 for skeletal traits at femoral neck. Physiological Genomics. 2008; **35**(2): 191-6.
2125. Aldhoon B, Zamrazilová H, Aldhoon Hainerová I, Srámková P, Spálová J, Kunesová M, et al. Role of the PPARalpha Leu162Val and PPARgamma2 Pro12Ala gene polymorphisms in weight change after 2.5-year follow-up in Czech obese women. Folia Biologica. 2010; **56**(3): 116-23.
2126. Allard JS, Ntekim O, Johnson SP, Ngwa JS, Bond V, Pinder D, et al. APOEepsilon4 impacts up-regulation of brain-derived neurotrophic factor after a six-month stretch and aerobic exercise intervention in mild cognitively impaired elderly African Americans: A pilot study. Exp Gerontol. 2017; **87**(Pt A): 129-36.
2127. Allard JS, Ntekim O, Johnson SP, Ngwa JS, Bond V, Pinder D, et al. APOEε4 impacts up-regulation of brain-derived neurotrophic factor after a six-month stretch and aerobic exercise intervention in mild cognitively impaired elderly African Americans: A pilot study. Experimental Gerontology. 2017; **87**(Pt A): 129-36.
2128. Aller E, Mariman ECM, Bouwman FG, van Baak MA. Genetic Predictors of >/=5% Weight Loss by Multidisciplinary Advice to Severely Obese Subjects. J Nutrigenet Nutrigenomics. 2017; **10**(1-2): 32-42.
2129. Aller EEJG, Mariman ECM, Bouwman FG, van Baak MA. Genetic Predictors of ≥5% Weight Loss by Multidisciplinary Advice to Severely Obese Subjects. Journal Of Nutrigenetics And Nutrigenomics. 2017; **10**(1-2): 32-42.
2130. Alli S, Figueiredo CA, Golbourn B, Sabha N, Wu MY, Bondoc A, et al. Brainstem blood brain barrier disruption using focused ultrasound: A demonstration of feasibility and enhanced doxorubicin delivery. Journal Of Controlled Release: Official Journal Of The Controlled Release Society. 2018; **281**: 29-41.
2131. Almeida JA, Boullosa DA, Pardono E, Lima RM, Morais PK, Denadai BS, et al. The influence of ACE genotype on cardiovascular fitness of moderately active young men. Arquivos Brasileiros De Cardiologia. 2012; **98**(4): 315-20.
2132. Almeida OP, Hankey GJ, Yeap BB, Golledge J, Flicker L. Alcohol consumption and cognitive impairment in older men: a mendelian randomization study. Neurology. 2014; **82**(12): 1038-44.
2133. Alves AJ, Goldhammer E, Ribeiro F, Eynon N, Ben-Zaken Cohen S, Duarte JA, et al. GNAS A-1121G variant is associated with improved diastolic dysfunction in response to exercise training in heart failure patients. International Journal Of Sports Medicine. 2013; **34**(3): 274-80.
2134. Alves CR, Fernandes T, Lemos JR, Jr., Magalhães FdC, Trombetta IC, Alves GB, et al. Aerobic exercise training differentially affects ACE C- and N-domain activities in humans: Interactions with ACE I/D polymorphism and association with vascular reactivity. Journal Of The Renin-Angiotensin-Aldosterone System: JRAAS. 2018; **19**(2):

1470320318761725-.
2135. Alves GB, Oliveira EM, Alves CR, Rached HRS, Mota GFA, Pereira AC, et al. Influence of angiotensinogen and angiotensin-converting enzyme polymorphisms on cardiac hypertrophy and improvement on maximal aerobic capacity caused by exercise training. European Journal Of Cardiovascular Prevention And Rehabilitation: Official Journal Of The European Society Of Cardiology, Working Groups On Epidemiology & Prevention And Cardiac Rehabilitation And Exercise Physiology. 2009; **16**(4): 487-92.
2136. Ananthakrishnan AN, Huang H, Nguyen DD, Sauk J, Yajnik V, Xavier RJ. Differential effect of genetic burden on disease phenotypes in Crohn's disease and ulcerative colitis: analysis of a North American cohort. The American Journal Of Gastroenterology. 2014; **109**(3): 395-400.
2137. Andersson ML, Eliasson E, Lindh JD. A clinically significant interaction between warfarin and simvastatin is unique to carriers of the CYP2C9*3 allele. Pharmacogenomics. 2012; **13**(7): 757-62.
2138. Antoun A, Vekaria D, Salama RA, Pratt G, Jobson S, Cook M, et al. The genotype of RAET1L (ULBP6), a ligand for human NKG2D (KLRK1), markedly influences the clinical outcome of allogeneic stem cell transplantation. British Journal Of Haematology. 2012; **159**(5): 589-98.
2139. Anttila V, Bulik-Sullivan B, Finucane HK, Walters RK, Bras J, Duncan L, et al. Analysis of shared heritability in common disorders of the brain. Science (New York, NY). 2018; **360**(6395).
2140. Aouad M, Zell V, Juif P-E, Lacaud A, Goumon Y, Darbon P, et al. Etifoxine analgesia in experimental monoarthritis: a combined action that protects spinal inhibition and limits central inflammatory processes. Pain. 2014; **155**(2): 403-12.
2141. Apalasamy YD, Ming MF, Rampal S, Bulgiba A, Mohamed Z. Association of melanocortin-4 receptor gene polymorphisms with obesity-related parameters in Malaysian Malays. Annals Of Human Biology. 2013; **40**(1): 102-6.
2142. Aranjuelo I, Cabrera-Bosquet L, Araus JL, Nogués S. Carbon and nitrogen partitioning during the post-anthesis period is conditioned by N fertilisation and sink strength in three cereals. Plant Biology (Stuttgart, Germany). 2013; **15**(1): 135-43.
2143. Aref MW, McNerny EM, Brown D, Jepsen KJ, Allen MR. Zoledronate treatment has different effects in mouse strains with contrasting baseline bone mechanical phenotypes. Osteoporos Int. 2016; **27**(12): 3637-43.
2144. Arendash GW, Jensen MT, Salem N, Jr., Hussein N, Cracchiolo J, Dickson A, et al. A diet high in omega-3 fatty acids does not improve or protect cognitive performance in Alzheimer's transgenic mice. Neuroscience. 2007; **149**(2): 286-302.
2145. Arold G, Donath F, Maurer A, Diefenbach K, Bauer S, Henneicke-von Zepelin H-H, et al. No relevant interaction with alprazolam, caffeine, tolbutamide, and digoxin by treatment with a low-hyperforin St John's wort extract. Planta Medica. 2005; **71**(4): 331-7.
2146. Arolt V, Zwanzger P, Ströhle A, Hamm A, Gerlach A, Kircher T, et al. [The research network PANIC-NET: improving the treatment of panic disorder - from a better understanding of fear circuit mechanisms to more effective psychological treatment and routine care]. Psychotherapie, Psychosomatik, Medizinische Psychologie. 2009; **59**(3-4): 124-31.

2147. Arruda VR, Fields PA, Milner R, Wainwright L, De Miguel MP, Donovan PJ, et al. Lack of germline transmission of vector sequences following systemic administration of recombinant AAV-2 vector in males. Molecular Therapy: The Journal Of The American Society Of Gene Therapy. 2001; **4**(6): 586-92.
2148. Arvizu RR, Domínguez IA, Rubio MS, Bórquez JL, Pinos-Rodríguez JM, González M, et al. Effects of genotype, level of supplementation, and organic chromium on growth performance, carcass, and meat traits grazing lambs. Meat Science. 2011; **88**(3): 404-8.
2149. Astrup A, Toubro S, Dalgaard LT, Urhammer SA, Sorensen TI, Pedersen O. Impact of the v/v 55 polymorphism of the uncoupling protein 2 gene on 24-h energy expenditure and substrate oxidation. International Journal Of Obesity And Related Metabolic Disorders: Journal Of The International Association For The Study Of Obesity. 1999; **23**(10): 1030-4.
2150. Atabaki-Pasdar N, Ohlsson M, Shungin D, Kurbasic A, Ingelsson E, Pearson ER, et al. Statistical power considerations in genotype-based recall randomized controlled trials. Scientific Reports. 2016; **6**: 37307-.
2151. Awandu SS, Raman J, Makhanthisa TI, Kruger P, Frean J, Bousema T, et al. Understanding human genetic factors influencing primaquine safety and efficacy to guide primaquine roll-out in a pre-elimination setting in southern Africa. Malaria Journal. 2018; **17**(1): 120-.
2152. Aziz I, Trott N, Briggs R, North JR, Hadjivassiliou M, Sanders DS. Efficacy of a Gluten-Free Diet in Subjects With Irritable Bowel Syndrome-Diarrhea Unaware of Their HLA-DQ2/8 Genotype. Clinical Gastroenterology And Hepatology: The Official Clinical Practice Journal Of The American Gastroenterological Association. 2016; **14**(5): 696-703.e1.
2153. Azogu I, Plamondon H. Inhibition of TrkB at the nucleus accumbens, using ANA-12, regulates basal and stress-induced orexin A expression within the mesolimbic system and affects anxiety, sociability and motivation. Neuropharmacology. 2017; **125**: 129-45.
2154. Babaei P, Shirkouhi SG, Hosseini R, Soltani Tehrani B. Vitamin D is associated with metabotropic but not neurotrophic effects of exercise in ovariectomized rats. Diabetology & Metabolic Syndrome. 2017; **9**: 91-.
2155. Baker LD, Frank LL, Foster-Schubert K, Green PS, Wilkinson CW, McTiernan A, et al. Effects of aerobic exercise on mild cognitive impairment: a controlled trial. Archives Of Neurology. 2010; **67**(1): 71-9.
2156. Balant-Gorgia AE, Balant LP, Genet C, Dayer P, Aeschlimann JM, Garrone G. Importance of oxidative polymorphism and levomepromazine treatment on the steady-state blood concentrations of clomipramine and its major metabolites. European Journal Of Clinical Pharmacology. 1986; **31**(4): 449-55.
2157. Balcerczyk A, Gajewska A, Macierzyńska-Piotrowska E, Pawelczyk T, Bartosz G, Szemraj J. Enhanced antioxidant capacity and anti-ageing biomarkers after diet micronutrient supplementation. Molecules (Basel, Switzerland). 2014; **19**(9): 14794-808.
2158. Bandele OJ, Wang X, Campbell MR, Pittman GS, Bell DA. Human single-nucleotide polymorphisms alter p53 sequence-specific binding at gene regulatory elements. Nucleic Acids Research. 2011; **39**(1): 178-89.

2159. Banerjee B, Vadiraj HS, Ram A, Rao R, Jayapal M, Gopinath KS, et al. Effects of an integrated yoga program in modulating psychological stress and radiation-induced genotoxic stress in breast cancer patients undergoing radiotherapy. Integrative Cancer Therapies. 2007; **6**(3): 242-50.
2160. Baño Otalora B, Popovic N, Gambini J, Popovic M, Viña J, Bonet-Costa V, et al. Circadian system functionality, hippocampal oxidative stress, and spatial memory in the APPswe/PS1dE9 transgenic model of Alzheimer disease: effects of melatonin or ramelteon. Chronobiology International. 2012; **29**(7): 822-34.
2161. Bansi J, Bloch W, Gamper U, Kesselring J. Training in MS: influence of two different endurance training protocols (aquatic versus overland) on cytokine and neurotrophin concentrations during three week randomized controlled trial. Multiple Sclerosis (Houndmills, Basingstoke, England). 2013; **19**(5): 613-21.
2162. Baptista T, ElFakih Y, Uzcátegui E, Sandia I, Tálamo E, Araujo de Baptista E, et al. Pharmacological management of atypical antipsychotic-induced weight gain. CNS Drugs. 2008; **22**(6): 477-95.
2163. Barbano RL, Hill DF, Snively BM, Light LS, Boggs N, McCall WV, et al. New triggers and non-motor findings in a family with rapid-onset dystonia-parkinsonism. Parkinsonism & Related Disorders. 2012; **18**(6): 737-41.
2164. Barkley-Levenson AM, Crabbe JC. Genotypic and sex differences in anxiety-like behavior and alcohol-induced anxiolysis in High Drinking in the Dark selected mice. Alcohol (Fayetteville, NY). 2015; **49**(1): 29-36.
2165. Barkley-Levenson AM, Lagarda FA, Palmer AA. Glyoxalase (GLO1) inhibition or genetic overexpression does not alter ethanol's locomotor effects: implications for GLO1 as a therapeutic target in alcohol use disorders. Alcohol Clin Exp Res. 2018.
2166. Barkley-Levenson AM, Lagarda FA, Palmer AA. Glyoxalase 1 (GLO1) Inhibition or Genetic Overexpression Does Not Alter Ethanol's Locomotor Effects: Implications for GLO1 as a Therapeutic Target in Alcohol Use Disorders. Alcoholism, Clinical And Experimental Research. 2018; **42**(5): 869-78.
2167. Beaver LM, Truong L, Barton CL, Chase TT, Gonnerman GD, Wong CP, et al. Combinatorial effects of zinc deficiency and arsenic exposure on zebrafish (Danio rerio) development. PLoS One. 2017; **12**(8): e0183831-e.
2168. Belfer I, Hipp H, McKnight C, Evans C, Buzas B, Bollettino A, et al. Association of galanin haplotypes with alcoholism and anxiety in two ethnically distinct populations. Molecular Psychiatry. 2006; **11**(3): 301-11.
2169. Bendlová B, Vejrazková D, Vcelák J, Lukásová P, Burkonová D, Kunesová M, et al. PPARgamma2 Pro12Ala polymorphism in relation to free fatty acids concentration and composition in lean healthy Czech individuals with and without family history of diabetes type 2. Physiological Research. 2008; **57 Suppl 1**: S77-S90.
2170. Benedetti F, Poletti S, Radaelli D, Bernasconi A, Cavallaro R, Falini A, et al. Temporal lobe grey matter volume in schizophrenia is associated with a genetic polymorphism influencing glycogen synthase kinase 3-beta activity. Genes Brain Behav. 2010; **9**(4): 365-71.
2171. Benedetti F, Poletti S, Radaelli D, Bernasconi A, Cavallaro R, Falini A, et al. Temporal lobe grey matter volume in schizophrenia is associated with a genetic polymorphism influencing glycogen synthase kinase 3-β activity. Genes, Brain, And

Behavior. 2010; **9**(4): 365-71.
2172. Benser J, Valtueña J, Ruiz JR, Mielgo-Ayuso J, Breidenassel C, Vicente-Rodriguez G, et al. Impact of physical activity and cardiovascular fitness on total homocysteine concentrations in European adolescents: The HELENA study. Journal Of Nutritional Science And Vitaminology. 2015; **61**(1): 45-54.
2173. Bentzen J, Jørgensen T, Fenger M. The effect of six polymorphisms in the Apolipoprotein B gene on parameters of lipid metabolism in a Danish population. Clinical Genetics. 2002; **61**(2): 126-34.
2174. Berentzen T, Kring SII, Holst C, Zimmermann E, Jess T, Hansen T, et al. Lack of association of fatness-related FTO gene variants with energy expenditure or physical activity. The Journal Of Clinical Endocrinology And Metabolism. 2008; **93**(7): 2904-8.
2175. Berg JP, Lehmann EH, Stakkestad JA, Haug E, Halse J. The Sp1 binding site polymorphism in the collagen type I alpha 1 (COLIA1) gene is not associated with bone mineral density in healthy children, adolescents, and young adults. European Journal Of Endocrinology. 2000; **143**(2): 261-5.
2176. Berger SM, Weber T, Perreau-Lenz S, Vogt MA, Gartside SE, Maser-Gluth C, et al. A functional Tph2 C1473G polymorphism causes an anxiety phenotype via compensatory changes in the serotonergic system. Neuropsychopharmacology: Official Publication Of The American College Of Neuropsychopharmacology. 2012; **37**(9): 1986-98.
2177. Berkun Y, Abou Atta I, Rubinow A, Orbach H, Levartovsky D, Aamar S, et al. 2756GG genotype of methionine synthase reductase gene is more prevalent in rheumatoid arthritis patients treated with methotrexate and is associated with methotrexate-induced nodulosis. The Journal Of Rheumatology. 2007; **34**(8): 1664-9.
2178. Berkun Y, Levartovsky D, Rubinow A, Orbach H, Aamar S, Grenader T, et al. Methotrexate related adverse effects in patients with rheumatoid arthritis are associated with the A1298C polymorphism of the MTHFR gene. Annals Of The Rheumatic Diseases. 2004; **63**(10): 1227-31.
2179. Bethea CL, Streicher JM, Coleman K, Pau FKY, Moessner R, Cameron JL. Anxious behavior and fenfluramine-induced prolactin secretion in young rhesus macaques with different alleles of the serotonin reuptake transporter polymorphism (5HTTLPR). Behavior Genetics. 2004; **34**(3): 295-307.
2180. Bey K, Lennertz L, Markett S, Petrovsky N, Gallinat J, Gründer G, et al. Replication of the association between CHRNA4 rs1044396 and harm avoidance in a large population-based sample. European Neuropsychopharmacology: The Journal Of The European College Of Neuropsychopharmacology. 2016; **26**(1): 150-5.
2181. Bhagat N, Agrawal M, Luthra K, Vikram NK, Misra A, Gupta R. Evaluation of single nucleotide polymorphisms of Pro12Ala in peroxisome proliferator-activated receptor-γ and Gly308Ala in tumor necrosis factor-α genes in obese Asian Indians: a population-based study. Diabetes, Metabolic Syndrome And Obesity: Targets And Therapy. 2010; **3**: 349-56.
2182. Bienertova-Vasku J, Bienert P, Slovackova L, Sablikova L, Piskackova Z, Forejt M, et al. Variability in CNR1 locus influences protein intake and smoking status in the Central-European population. Nutritional Neuroscience. 2012; **15**(4): 163-70.
2183. Binkley K, King N, Poonai N, Seeman P, Ulpian C, Kennedy J. Idiopathic

environmental intolerance: increased prevalence of panic disorder-associated cholecystokinin B receptor allele 7. The Journal Of Allergy And Clinical Immunology. 2001; **107**(5): 887-90.
2184. Bjørnland T, Langaas M, Grill V, Mostad IL. Assessing gene-environment interaction effects of FTO, MC4R and lifestyle factors on obesity using an extreme phenotype sampling design: Results from the HUNT study. PLoS One. 2017; **12**(4): e0175071-e.
2185. Blaauwwiekel EE, Beusekamp BJ, Sluiter WJ, Hoogenberg K, Dullaart RP. Apolipoprotein E genotype is a determinant of low-density lipoprotein cholesterol and of its response to a low-cholesterol diet in Type 1 diabetic patients with elevated urinary albumin excretion. Diabetic Medicine: A Journal Of The British Diabetic Association. 1998; **15**(12): 1031-5.
2186. Blättler W, Lüscher D, Brizzio E, Willenberg T, Baumgartner I, Amsler F. Healing of chronic venous leg ulcers could be affected by an interaction of the hemochromatosis gene polymorphism HFE H63D with the strength of compression treatment--a re-analysis of patients from previous studies. Wound Repair And Regeneration: Official Publication Of The Wound Healing Society [And] The European Tissue Repair Society. 2012; **20**(1): 123-4.
2187. Bloss CS, Berrettini W, Bergen AW, Magistretti P, Duvvuri V, Strober M, et al. Genetic association of recovery from eating disorders: the role of GABA receptor SNPs. Neuropsychopharmacology: Official Publication Of The American College Of Neuropsychopharmacology. 2011; **36**(11): 2222-32.
2188. Bloss CS, Schork NJ, Topol EJ. Effect of direct-to-consumer genomewide profiling to assess disease risk. The New England Journal Of Medicine. 2011; **364**(6): 524-34.
2189. Boenigk J, Beisser D, Zimmermann S, Bock C, Jakobi J, Grabner D, et al. Effects of silver nitrate and silver nanoparticles on a planktonic community: general trends after short-term exposure. PLoS One. 2014; **9**(4): e95340-e.
2190. Boer JM, Ehnholm C, Menzel HJ, Havekes LM, Rosseneu M, O'Reilly DS, et al. Interactions between lifestyle-related factors and the ApoE polymorphism on plasma lipids and apolipoproteins. The EARS Study. European Atherosclerosis Research Study. Arteriosclerosis, Thrombosis, And Vascular Biology. 1997; **17**(9): 1675-81.
2191. Booth-Gauthier EA, Alcoser TA, Yang G, Dahl KN. Force-induced changes in subnuclear movement and rheology. Biophysical Journal. 2012; **103**(12): 2423-31.
2192. Bosia M, Bechi M, Marino E, Anselmetti S, Poletti S, Cocchi F, et al. Influence of catechol-O-methyltransferase Val158Met polymorphism on neuropsychological and functional outcomes of classical rehabilitation and cognitive remediation in schizophrenia. Neuroscience Letters. 2007; **417**(3): 271-4.
2193. Bossé Y, Vohl MC, Dumont M, Brochu M, Bergeron J, Després JP, et al. Influence of the angiotensin-converting enzyme gene insertion/deletion polymorphism on lipoprotein/lipid response to gemfibrozil. Clinical Genetics. 2002; **62**(1): 45-52.
2194. Bouchard C, An P, Rice T, Skinner JS, Wilmore JH, Gagnon J, et al. Familial aggregation of VO(2max) response to exercise training: results from the HERITAGE Family Study. Journal Of Applied Physiology (Bethesda, Md: 1985). 1999; **87**(3): 1003-8.

2195. Bouchard C, Daw EW, Rice T, Pérusse L, Gagnon J, Province MA, et al. Familial resemblance for VO2max in the sedentary state: the HERITAGE family study. Medicine And Science In Sports And Exercise. 1998; **30**(2): 252-8.
2196. Bouchard C, Pérusse L, Leblanc C. Using MZ twins in experimental research to test for the presence of a genotype-environment interaction effect. Acta Geneticae Medicae Et Gemellologiae. 1990; **39**(1): 85-9.
2197. Bouchard C, Rankinen T, Chagnon YC, Rice T, Pérusse L, Gagnon J, et al. Genomic scan for maximal oxygen uptake and its response to training in the HERITAGE Family Study. Journal Of Applied Physiology (Bethesda, Md: 1985). 2000; **88**(2): 551-9.
2198. Bouchard C, Sarzynski MA, Rice TK, Kraus WE, Church TS, Sung YJ, et al. Genomic predictors of the maximal O_2 uptake response to standardized exercise training programs. Journal Of Applied Physiology (Bethesda, Md: 1985). 2011; **110**(5): 1160-70.
2199. Bouchard C, Tremblay A. Genetic effects in human energy expenditure components. International Journal Of Obesity. 1990; **14 Suppl 1**: 49-55.
2200. Bouchard C, Tremblay A. Genetic influences on the response of body fat and fat distribution to positive and negative energy balances in human identical twins. The Journal Of Nutrition. 1997; **127**(5 Suppl): 943S-7S.
2201. Bouchard C, Tremblay A, Després JP, Nadeau A, Lupien PJ, Thériault G, et al. The response to long-term overfeeding in identical twins. The New England Journal Of Medicine. 1990; **322**(21): 1477-82.
2202. Bouchard C, Tremblay A, Després JP, Thériault G, Nadeau A, Lupien PJ, et al. The response to exercise with constant energy intake in identical twins. Obesity Research. 1994; **2**(5): 400-10.
2203. Boucher AA, Arnold JC, Duffy L, Schofield PR, Micheau J, Karl T. Heterozygous neuregulin 1 mice are more sensitive to the behavioural effects of Delta9-tetrahydrocannabinol. Psychopharmacology. 2007; **192**(3): 325-36.
2204. Brant F, Miranda AS, Esper L, Gualdrón-López M, Cisalpino D, de Souza DdG, et al. Suppressor of cytokine signaling 2 modulates the immune response profile and development of experimental cerebral malaria. Brain, Behavior, And Immunity. 2016; **54**: 73-85.
2205. Breen MS, Uhlmann A, Nday CM, Glatt SJ, Mitt M, Metsalpu A, et al. Candidate gene networks and blood biomarkers of methamphetamine-associated psychosis: an integrative RNA-sequencing report. Translational Psychiatry. 2016; **6**: e802-e.
2206. Brehm JM, Ramratnam SK, Tse SM, Croteau-Chonka DC, Pino-Yanes M, Rosas-Salazar C, et al. Stress and Bronchodilator Response in Children with Asthma. American Journal Of Respiratory And Critical Care Medicine. 2015; **192**(1): 47-56.
2207. Brenmoehl J, Walz C, Spitschak M, Wirthgen E, Walz M, Langhammer M, et al. Partial phenotype conversion and differential trait response to conditions of husbandry in mice. J Comp Physiol B. 2017.
2208. Brouwer-Brolsma EM, Vaes AMM, van der Zwaluw NL, van Wijngaarden JP, Swart KMA, Ham AC, et al. Relative importance of summer sun exposure, vitamin D intake, and genes to vitamin D status in Dutch older adults: The B-PROOF study. The Journal Of Steroid Biochemistry And Molecular Biology. 2016; **164**: 168-76.
2209. Brunkwall L, Chen Y, Hindy G, Rukh G, Ericson U, Barroso I, et al. Sugar-

sweetened beverage consumption and genetic predisposition to obesity in 2 Swedish cohorts. The American Journal Of Clinical Nutrition. 2016; **104**(3): 809-15.
2210. Brunoni AR, Lotufo PA, Sabbag C, Goulart AC, Santos IS, Benseñor IM. Decreased brain-derived neurotrophic factor plasma levels in psoriasis patients. Brazilian Journal Of Medical And Biological Research = Revista Brasileira De Pesquisas Medicas E Biologicas. 2015; **48**(8): 711-4.
2211. Bryan AD, Magnan RE, Hooper AEC, Harlaar N, Hutchison KE. Physical activity and differential methylation of breast cancer genes assayed from saliva: a preliminary investigation. Annals Of Behavioral Medicine: A Publication Of The Society Of Behavioral Medicine. 2013; **45**(1): 89-98.
2212. Brzęk P, Gębczyński AK, Książek A, Konarzewski M. Effect of calorie restriction on spontaneous physical activity and body mass in mice divergently selected for basal metabolic rate (BMR). Physiology & Behavior. 2016; **161**: 116-22.
2213. Buchwald P, Juhász A, Bell C, Pátfalusi M, Howes J, Bodor N. Unified pharmacogenetics-based parent-metabolite pharmacokinetic model incorporating acetylation polymorphism for talampanel in humans. Journal Of Pharmacokinetics And Pharmacodynamics. 2005; **32**(3-4): 377-400.
2214. Buemann B, Schierning B, Toubro S, Bibby BM, Sørensen T, Dalgaard L, et al. The association between the val/ala-55 polymorphism of the uncoupling protein 2 gene and exercise efficiency. International Journal Of Obesity And Related Metabolic Disorders: Journal Of The International Association For The Study Of Obesity. 2001; **25**(4): 467-71.
2215. Bueno Júnior CR, Pantaleão LC, Voltarelli VA, Bozi LHM, Brum PC, Zatz M. Combined effect of AMPK/PPAR agonists and exercise training in mdx mice functional performance. PLoS One. 2012; **7**(9): e45699-e.
2216. Buga A-M, Scholz CJ, Kumar S, Herndon JG, Alexandru D, Cojocaru GR, et al. Identification of new therapeutic targets by genome-wide analysis of gene expression in the ipsilateral cortex of aged rats after stroke. PLoS One. 2012; **7**(12): e50985-e.
2217. Bulmer DC, Botha CA, Wheeldon A, Grey K, Mein CA, Lee K, et al. Evidence of a role for GTP cyclohydrolase-1 in visceral pain. Neurogastroenterology And Motility: The Official Journal Of The European Gastrointestinal Motility Society. 2015; **27**(5): 656-62.
2218. Burokienė N, Domarkienė I, Ambrozaitytė L, Uktverytė I, Meškienė R, Karčiauskaitė D, et al. Classical rather than genetic risk factors account for high cardiovascular disease prevalence in Lithuania: A cross-sectional population study. Advances In Medical Sciences. 2017; **62**(1): 121-8.
2219. Burrows EL, Laskaris L, Koyama L, Churilov L, Bornstein JC, Hill-Yardin EL, et al. A neuroligin-3 mutation implicated in autism causes abnormal aggression and increases repetitive behavior in mice. Mol Autism. 2015; **6**: 62.
2220. Byrne EM, Carrillo-Roa T, Henders AK, Bowdler L, McRae AF, Heath AC, et al. Monozygotic twins affected with major depressive disorder have greater variance in methylation than their unaffected co-twin. Translational Psychiatry. 2013; **3**: e269-e.
2221. Cabanero M, Laje G, Detera-Wadleigh S, McMahon FJ. Association study of phosphodiesterase genes in the Sequenced Treatment Alternatives to Relieve Depression sample. Pharmacogenetics And Genomics. 2009; **19**(3): 235-8.
2222. Callaghan CK, Rouine J, O'Mara SM. Exercise prevents IFN-alpha-induced

mood and cognitive dysfunction and increases BDNF expression in the rat. Physiol Behav. 2017; **179**: 377-83.
2223. Callaghan CK, Rouine J, O'Mara SM. Exercise prevents IFN-α-induced mood and cognitive dysfunction and increases BDNF expression in the rat. Physiology & Behavior. 2017; **179**: 377-83.
2224. Cam FS, Colakoglu M, Sekuri C, Colakoglu S, Sahan C, Berdeli A. Association between the ACE I/D gene polymorphism and physical performance in a homogeneous non-elite cohort. Canadian Journal Of Applied Physiology = Revue Canadienne De Physiologie Appliquee. 2005; **30**(1): 74-86.
2225. Capello AEM, Markus CR. Differential influence of the 5-HTTLPR genotype, neuroticism and real-life acute stress exposure on appetite and energy intake. Appetite. 2014; **77**: 83-93.
2226. Capello AEM, Markus CR. Effect of sub chronic tryptophan supplementation on stress-induced cortisol and appetite in subjects differing in 5-HTTLPR genotype and trait neuroticism. Psychoneuroendocrinology. 2014; **45**: 96-107.
2227. Carlsson B, Holmgren A, Ahlner J, Bengtsson F. Enantioselective analysis of citalopram and escitalopram in postmortem blood together with genotyping for CYP2D6 and CYP2C19. Journal Of Analytical Toxicology. 2009; **33**(2): 65-76.
2228. Carneiro AMD, Airey DC, Thompson B, Zhu C-B, Lu L, Chesler EJ, et al. Functional coding variation in recombinant inbred mouse lines reveals multiple serotonin transporter-associated phenotypes. Proceedings Of The National Academy Of Sciences Of The United States Of America. 2009; **106**(6): 2047-52.
2229. Carnero E, Barriocanal M, Segura V, Guruceaga E, Prior C, Börner K, et al. Type I Interferon Regulates the Expression of Long Non-Coding RNAs. Frontiers In Immunology. 2014; **5**: 548-.
2230. Carreño Gutiérrez H, O'Leary A, Freudenberg F, Fedele G, Wilkinson R, Markham E, et al. Nitric oxide interacts with monoamine oxidase to modulate aggression and anxiety-like behaviour. European Neuropsychopharmacology: The Journal Of The European College Of Neuropsychopharmacology. 2017.
2231. Cavaco S, da Silva AM, Pinto P, Coutinho E, Santos E, Bettencourt A, et al. Cognitive functioning in Behçet's disease. Annals Of The New York Academy Of Sciences. 2009; **1173**: 217-26.
2232. Ceglia L. Vitamin D and skeletal muscle tissue and function. Molecular Aspects Of Medicine. 2008; **29**(6): 407-14.
2233. Celorrio D, Muñoz X, Amiano P, Dorronsoro M, Bujanda L, Sánchez M-J, et al. Influence of Dopaminergic System Genetic Variation and Lifestyle Factors on Excessive Alcohol Consumption. Alcohol And Alcoholism (Oxford, Oxfordshire). 2016; **51**(3): 258-67.
2234. Chan LG, Ho MJ, Kaur P, Singh J, Ng OT, Lee CC, et al. Differences in clinical and psychiatric outcomes between prevalent HIV-1 molecular subtypes in a multiethnic Southeast Asian sample. General Hospital Psychiatry. 2016; **38**: 4-8.
2235. Chappell MA, Hayes JP, Snyder LRG. HEMOGLOBIN POLYMORPHISMS IN DEER MICE (PEROMYSCUS MANICULATUS): PHYSIOLOGY OF BETA-GLOBIN VARIANTS AND ALPHA-GLOBIN RECOMBINANTS. Evolution; International Journal Of Organic Evolution. 1988; **42**(4): 681-8.

2236. Charney DS. Neuroanatomical circuits modulating fear and anxiety behaviors. Acta Psychiatrica Scandinavica Supplementum. 2003; (417): 38-50.
2237. Chen C-Y, Yeh H-H, Huang N, Lin Y-C. Socioeconomic and clinical characteristics associated with repeat suicide attempts among young people. The Journal Of Adolescent Health: Official Publication Of The Society For Adolescent Medicine. 2014; **54**(5): 550-7.
2238. Chen G, Henter ID, Manji HK. Translational research in bipolar disorder: emerging insights from genetically based models. Molecular Psychiatry. 2010; **15**(9): 883-95.
2239. Chen J-H, Muo C-H, Kao C-H, Tsai C-H, Tseng C-H. Increased Risk of New-Onset Fibromyalgia Among Chronic Osteomyelitis Patients: Evidence From a Taiwan Cohort Study. The Journal Of Pain: Official Journal Of The American Pain Society. 2017; **18**(2): 222-7.
2240. Chen NG, Yu YA, Zhang Q, Szalay AA. Replication efficiency of oncolytic vaccinia virus in cell cultures prognosticates the virulence and antitumor efficacy in mice. Journal Of Translational Medicine. 2011; **9**: 164-.
2241. Chia N-Y, Deng N, Das K, Huang D, Hu L, Zhu Y, et al. Regulatory crosstalk between lineage-survival oncogenes KLF5, GATA4 and GATA6 cooperatively promotes gastric cancer development. Gut. 2015; **64**(5): 707-19.
2242. Chiu C-T, Liu G, Leeds P, Chuang D-M. Combined treatment with the mood stabilizers lithium and valproate produces multiple beneficial effects in transgenic mouse models of Huntington's disease. Neuropsychopharmacology: Official Publication Of The American College Of Neuropsychopharmacology. 2011; **36**(12): 2406-21.
2243. Choi H-Y, Kim GE, Kong KA, Lee YJ, Lim W-J, Park S-H, et al. Psychological and genetic risk factors associated with suicidal behavior in Korean patients with mood disorders. Journal Of Affective Disorders. 2018; **235**: 489-98.
2244. Choi YJ, Nam YS, Yun JM, Park JH, Cho BL, Son HY, et al. Association between salivary amylase (AMY1) gene copy numbers and insulin resistance in asymptomatic Korean men. Diabetic Medicine: A Journal Of The British Diabetic Association. 2015; **32**(12): 1588-95.
2245. Chomistek AK, Chasman DI, Cook NR, Rimm EB, Lee IM. Physical activity, genes for physical fitness, and risk of coronary heart disease. Medicine And Science In Sports And Exercise. 2013; **45**(4): 691-7.
2246. Chong SA, Tan EC, Tan CH, Mahendren R, Tay AH, Chua HC. Tardive dyskinesia is not associated with the serotonin gene polymorphism (5-HTTLPR) in Chinese. American Journal Of Medical Genetics. 2000; **96**(6): 712-5.
2247. Chowdhury MAZ, Jahan I, Karim N, Alam MK, Abdur Rahman M, Moniruzzaman M, et al. Determination of carbamate and organophosphorus pesticides in vegetable samples and the efficiency of gamma-radiation in their removal. Biomed Research International. 2014; **2014**: 145159-.
2248. Chu C-S, Li D-J, Chu C-L, Wu C-C, Lu T. Decreased IL-1ra and NCAM-1/CD56 Serum Levels in Unmedicated Patients with Schizophrenia Before and After Antipsychotic Treatment. Psychiatry Investigation. 2018.
2249. Chuang Y-H, Quach A, Absher D, Assimes T, Horvath S, Ritz B. Coffee consumption is associated with DNA methylation levels of human blood. European

Journal Of Human Genetics: EJHG. 2017; **25**(5): 608-16.
2250. Chung K-H, Li C-Y, Kuo S-Y, Sithole T, Liu W-W, Chung M-H. Risk of psychiatric disorders in patients with chronic insomnia and sedative-hypnotic prescription: a nationwide population-based follow-up study. Journal Of Clinical Sleep Medicine: JCSM: Official Publication Of The American Academy Of Sleep Medicine. 2015; **11**(5): 543-51.
2251. Church C, Lee S, Bagg EAL, McTaggart JS, Deacon R, Gerken T, et al. A mouse model for the metabolic effects of the human fat mass and obesity associated FTO gene. Plos Genetics. 2009; **5**(8): e1000599-e.
2252. Cladel NM, Hu J, Balogh KK, Christensen ND. Differences in methodology, but not differences in viral strain, account for variable experimental outcomes in laboratories utilizing the cottontail rabbit papillomavirus model. Journal Of Virological Methods. 2010; **165**(1): 36-41.
2253. Claghorn GC, Fonseca IAT, Thompson Z, Barber C, Garland T, Jr. Serotonin-mediated central fatigue underlies increased endurance capacity in mice from lines selectively bred for high voluntary wheel running. Physiology & Behavior. 2016; **161**: 145-54.
2254. Coates RF, Gardner J-A, Gao Y, Cortright VM, Mitchell JM, Ashikaga T, et al. Significance of positive and inhibitory regulators in the TGF-β signaling pathway in colorectal cancers. Human Pathology. 2017; **66**: 34-9.
2255. Cohen H, Neumann L, Glazer Y, Ebstein RP, Buskila D. The relationship between a common catechol-O-methyltransferase (COMT) polymorphism val(158) met and fibromyalgia. Clinical And Experimental Rheumatology. 2009; **27**(5 Suppl 56): S51-S6.
2256. Cohen H, Zohar J, Kaplan Z, Arnt J. Adjunctive treatment with brexpiprazole and escitalopram reduces behavioral stress responses and increase hypothalamic NPY immunoreactivity in a rat model of PTSD-like symptoms. Eur Neuropsychopharmacol. 2018; **28**(1): 63-74.
2257. Coker RH, Deutz NE, Schutzler S, Beggs M, Miller S, Wolfe RR, et al. Nutritional Supplementation with Essential Amino Acids and Phytosterols May Reduce Risk for Metabolic Syndrome and Cardiovascular Disease in Overweight Individuals with Mild Hyperlipidemia. Journal Of Endocrinology, Diabetes & Obesity. 2015; **3**(2).
2258. Comer SD, Sullivan MA, Vosburg SK, Kowalczyk WJ, Houser J. Abuse liability of oxycodone as a function of pain and drug use history. Drug And Alcohol Dependence. 2010; **109**(1-3): 130-8.
2259. Comings DE, Dietz G, Gade-Andavolu R, Blake H, Muhleman D, Huss M, et al. Association of the neutral endopeptidase (MME) gene with anxiety. Psychiatric Genetics. 2000; **10**(2): 91-4.
2260. Conceição LLd, Dias MDM, Pessoa MC, Pena GDG, Mendes MCS, Neves CVB, et al. Difference in fatty acids composition of breast adipose tissue in women with breast cancer and benign breast disease. Nutricion Hospitalaria. 2016; **33**(6): 1354-60.
2261. Cong X, Doering J, Mazala DAG, Chin ER, Grange RW, Jiang H. The SH3 and cysteine-rich domain 3 (Stac3) gene is important to growth, fiber composition, and calcium release from the sarcoplasmic reticulum in postnatal skeletal muscle. Skeletal Muscle. 2016; **6**: 17-.
2262. Connarn JN, Flowers S, Kelly M, Luo R, Ward KM, Harrington G, et al. Pharmacokinetics and Pharmacogenomics of Bupropion in Three Different Formulations

with Different Release Kinetics in Healthy Human Volunteers. The AAPS Journal. 2017; **19**(5): 1513-22.
2263. Connor DA, Gould TJ. Chronic fluoxetine ameliorates adolescent chronic nicotine exposure-induced long-term adult deficits in trace conditioning. Neuropharmacology. 2017; **125**: 272-83.
2264. Contin M, Sangiorgi S, Riva R, Parmeggiani A, Albani F, Baruzzi A. Evidence of polymorphic CYP2C19 involvement in the human metabolism of N-desmethylclobazam. Therapeutic Drug Monitoring. 2002; **24**(6): 737-41.
2265. Contreras J, Hare E, Chavarría G, Raventós H. Quantitative genetic analysis of anxiety trait in bipolar disorder. Journal Of Affective Disorders. 2018; **225**: 395-8.
2266. Cook MN, Baker JA, Heldt SA, Williams RW, Hamre KM, Lu L. Identification of candidate genes that underlie the QTL on chromosome 1 that mediates genetic differences in stress-ethanol interactions. Physiological Genomics. 2015; **47**(8): 308-17.
2267. Cooper AJ, Narasimhan S, Rickels K, Lohoff FW. Genetic polymorphisms in the PACAP and PAC1 receptor genes and treatment response to venlafaxine XR in generalized anxiety disorder. Psychiatry Research. 2013; **210**(3): 1299-300.
2268. Cooper AJ, Rickels K, Lohoff FW. Association analysis between the A118G polymorphism in the OPRM1 gene and treatment response to venlafaxine XR in generalized anxiety disorder. Human Psychopharmacology. 2013; **28**(3): 258-62.
2269. Corella D, Arnett DK, Tucker KL, Kabagambe EK, Tsai M, Parnell LD, et al. A high intake of saturated fatty acids strengthens the association between the fat mass and obesity-associated gene and BMI. The Journal Of Nutrition. 2011; **141**(12): 2219-25.
2270. Correa-Rodríguez M, Schmidt-RioValle J, Rueda-Medina B. RANKL/RANK/OPG Polymorphisms and Heel Quantitative Ultrasound in Young Adults. Nursing Research. 2017; **66**(2): 145-51.
2271. Costa-Urrutia P, Abud C, Franco-Trecu V, Colistro V, Rodríguez-Arellano ME, Vázquez-Pérez J, et al. Genetic Obesity Risk and Attenuation Effect of Physical Fitness in Mexican-Mestizo Population: a Case-Control Study. Annals Of Human Genetics. 2017; **81**(3): 106-16.
2272. Couly S, Paucard A, Bonneaud N, Maurice T, Benigno L, Jourdan C, et al. Improvement of BDNF signalling by P42 peptide in Huntington's disease. Human Molecular Genetics. 2018.
2273. Crocco P, Montesanto A, Passarino G, Rose G. Polymorphisms Falling Within Putative miRNA Target Sites in the 3'UTR Region of SIRT2 and DRD2 Genes Are Correlated With Human Longevity. The Journals Of Gerontology Series A, Biological Sciences And Medical Sciences. 2016; **71**(5): 586-92.
2274. Cserép Z, Balog P, Székely J, Treszl A, Kopp MS, Thayer JF, et al. Psychosocial factors and major adverse cardiac and cerebrovascular events after cardiac surgery. Interactive Cardiovascular And Thoracic Surgery. 2010; **11**(5): 567-72.
2275. Currie J, Ramsbottom R, Ludlow H, Nevill A, Gilder M. Cardio-respiratory fitness, habitual physical activity and serum brain derived neurotrophic factor (BDNF) in men and women. Neuroscience Letters. 2009; **451**(2): 152-5.
2276. Curti ML, Pires MM, Barros CR, Siqueira-Catania A, Rogero MM, Ferreira SR. Associations of the TNF-alpha -308 G/A, IL6 -174 G/C and AdipoQ 45 T/G polymorphisms with inflammatory and metabolic responses to lifestyle intervention in

Brazilians at high cardiometabolic risk. Diabetol Metab Syndr. 2012; **4**(1): 49.
2277. Curti ML, Pires MM, Barros CR, Siqueira-Catania A, Rogero MM, Ferreira SR. Associations of the TNF-alpha -308 G/A, IL6 -174 G/C and AdipoQ 45 T/G polymorphisms with inflammatory and metabolic responses to lifestyle intervention in Brazilians at high cardiometabolic risk. Diabetology & Metabolic Syndrome. 2012; **4**(1): 49-.
2278. Curti ML, Rogero MM, Baltar VT, Barros CR, Siqueira-Catania A, Ferreira SR. FTO T/A and peroxisome proliferator-activated receptor-gamma Pro12Ala polymorphisms but not ApoA1 -75 are associated with better response to lifestyle intervention in Brazilians at high cardiometabolic risk. Metab Syndr Relat Disord. 2013; **11**(3): 169-76.
2279. Curti MLR, Rogero MM, Baltar VT, Barros CR, Siqueira-Catania A, Ferreira SRG. FTO T/A and peroxisome proliferator-activated receptor-γ Pro12Ala polymorphisms but not ApoA1 -75 are associated with better response to lifestyle intervention in Brazilians at high cardiometabolic risk. Metabolic Syndrome And Related Disorders. 2013; **11**(3): 169-76.
2280. Cutler JA, Rush AJ, McMahon FJ, Laje G. Common genetic variation in the indoleamine-2,3-dioxygenase genes and antidepressant treatment outcome in major depressive disorder. Journal Of Psychopharmacology (Oxford, England). 2012; **26**(3): 360-7.
2281. da Silva JRD, Freire IV, Ribeiro ÍJS, Dos Santos CS, Casotti CA, Dos Santos DB, et al. Improving the comprehension of sarcopenic state determinants: An multivariate approach involving hormonal, nutritional, lifestyle and genetic variables. Mechanisms Of Ageing And Development. 2018; **173**: 21-8.
2282. da Silva VC, Ramos FJdC, Freitas EM, de Brito-Marques PR, Cavalcanti MNdH, D'Almeida V, et al. Alzheimer's disease in Brazilian elderly has a relation with homocysteine but not with MTHFR polymorphisms. Arquivos De Neuro-Psiquiatria. 2006; **64**(4): 941-5.
2283. Dai F, Belfer I, Schwartz CE, Banco R, Martha JF, Tighioughart H, et al. Association of catechol-O-methyltransferase genetic variants with outcome in patients undergoing surgical treatment for lumbar degenerative disc disease. The Spine Journal: Official Journal Of The North American Spine Society. 2010; **10**(11): 949-57.
2284. Dallongeville J, Helbecque N, Cottel D, Amouyel P, Meirhaeghe A. The Gly16-->Arg16 and Gln27-->Glu27 polymorphisms of beta2-adrenergic receptor are associated with metabolic syndrome in men. The Journal Of Clinical Endocrinology And Metabolism. 2003; **88**(10): 4862-6.
2285. Danis A, Kyriazis Y, Klissouras V. The effect of training in male prepubertal and pubertal monozygotic twins. European Journal Of Applied Physiology. 2003; **89**(3-4): 309-18.
2286. Davidsen ES, Liseth K, Omvik P, Hervig T, Gerdts E. Reduced exercise capacity in genetic haemochromatosis. European Journal Of Cardiovascular Prevention And Rehabilitation: Official Journal Of The European Society Of Cardiology, Working Groups On Epidemiology & Prevention And Cardiac Rehabilitation And Exercise Physiology. 2007; **14**(3): 470-5.
2287. Davidson S, Miller KA, Dowell A, Gildea A, Mackenzie A. A remote and highly

conserved enhancer supports amygdala specific expression of the gene encoding the anxiogenic neuropeptide substance-P. Molecular Psychiatry. 2006; **11**(4): 323.
2288. Davis W, van Rensburg SJ, Cronje FJ, Whati L, Fisher LR, van der Merwe L, et al. The fat mass and obesity-associated FTO rs9939609 polymorphism is associated with elevated homocysteine levels in patients with multiple sclerosis screened for vascular risk factors. Metabolic Brain Disease. 2014; **29**(2): 409-19.
2289. Dawson NM, Hamid EH, Egan MF, Meredith GE. Changes in the pattern of brain-derived neurotrophic factor immunoreactivity in the rat brain after acute and subchronic haloperidol treatment. Synapse (New York, NY). 2001; **39**(1): 70-81.
2290. Day SH, Gohlke P, Dhamrait SS, Williams AG. No correlation between circulating ACE activity and VO2max or mechanical efficiency in women. European Journal Of Applied Physiology. 2007; **99**(1): 11-8.
2291. Day SH, Williams C, Folland JP, Gohlke P, Williams AG. The acute effects of exercise and glucose ingestion on circulating angiotensin-converting enzyme in humans. European Journal Of Applied Physiology. 2004; **92**(4-5): 579-83.
2292. de Almeida Magalhaes T, Correia D, de Carvalho LM, Damasceno S, Brunialti Godard AL. Maternal separation affects expression of stress response genes and increases vulnerability to ethanol consumption. Brain Behav. 2018; **8**(1): e00841.
2293. de Klerk OL, Nolte IM, Bet PM, Bosker FJ, Snieder H, den Boer JA, et al. ABCB1 gene variants influence tolerance to selective serotonin reuptake inhibitors in a large sample of Dutch cases with major depressive disorder. The Pharmacogenomics Journal. 2013; **13**(4): 349-53.
2294. de Luis D, Izaola O, Primo D, Gomez E, Lopez JJ, Ortola A, et al. Association of a cholesteryl ester transfer protein variant (rs1800777) with fat mass, HDL cholesterol levels, and metabolic syndrome. Endocrinologia, Diabetes Y Nutricion. 2018.
2295. De Mello Costa MF, Anderson GA, Davies HM, Slocombe RF. Effects of acute exercise on angiotensin I-converting enzyme (ACE) activity in horses. Equine Veterinary Journal. 2012; **44**(4): 487-9.
2296. de Oliveira FF, Pivi GAK, Chen ES, Smith MC, Bertolucci PHF. Risk factors for cognitive and functional change in one year in patients with Alzheimer's disease dementia from São Paulo, Brazil. Journal Of The Neurological Sciences. 2015; **359**(1-2): 127-32.
2297. de Vries JM, van der Beek NAME, Kroos MA, Ozkan L, van Doorn PA, Richards SM, et al. High antibody titer in an adult with Pompe disease affects treatment with alglucosidase alfa. Molecular Genetics And Metabolism. 2010; **101**(4): 338-45.
2298. Decroix L, Tonoli C, Soares DD, Tagougui S, Heyman E, Meeusen R. Acute cocoa flavanol improves cerebral oxygenation without enhancing executive function at rest or after exercise. Applied Physiology, Nutrition, And Metabolism = Physiologie Appliquee, Nutrition Et Metabolisme. 2016; **41**(12): 1225-32.
2299. Deforche K, Cozzi-Lepri A, Theys K, Clotet B, Camacho RJ, Kjaer J, et al. Modelled in vivo HIV fitness under drug selective pressure and estimated genetic barrier towards resistance are predictive for virological response. Antiviral Therapy. 2008; **13**(3): 399-407.
2300. Dekeyne A, Mannoury la Cour C, Gobert A, Brocco M, Lejeune F, Serres F, et al. S32006, a novel 5-HT2C receptor antagonist displaying broad-based antidepressant

and anxiolytic properties in rodent models. Psychopharmacology. 2008; **199**(4): 549-68.
2301. Dériaz O, Dionne F, Pérusse L, Tremblay A, Vohl MC, Côté G, et al. DNA variation in the genes of the Na,K-adenosine triphosphatase and its relation with resting metabolic rate, respiratory quotient, and body fat. The Journal Of Clinical Investigation. 1994; **93**(2): 838-43.
2302. Dew-Budd K, Jarnigan J, Reed LK. Genetic and Sex-Specific Transgenerational Effects of a High Fat Diet in Drosophila melanogaster. PLoS One. 2016; **11**(8): e0160857-e.
2303. Diane A, Vine DF, Heth CD, Russell JC, Proctor SD, Pierce WD. Prior caloric restriction increases survival of prepubertal obese- and PCOS-prone rats exposed to a challenge of time-limited feeding and physical activity. Journal Of Applied Physiology (Bethesda, Md: 1985). 2013; **114**(9): 1158-64.
2304. Dias RG, Silva MSM, Duarte NE, Bolani W, Alves CR, Junior JRL, et al. PBMCs express a transcriptome signature predictor of oxygen uptake responsiveness to endurance exercise training in men. Physiological Genomics. 2015; **47**(2): 13-23.
2305. Dias VT, Vey LT, Rosa HZ, D'Avila L F, Barcelos RCS, Burger ME. Could Modafinil Prevent Psychostimulant Addiction? An Experimental Study in Rats. Basic Clin Pharmacol Toxicol. 2017; **121**(5): 400-8.
2306. Diercke K, Kohl A, Lux CJ, Erber R. Compression of human primary cementoblasts leads to apoptosis: A possible cause of dental root resorption? Journal Of Orofacial Orthopedics = Fortschritte Der Kieferorthopadie: Organ/Official Journal Deutsche Gesellschaft Fur Kieferorthopadie. 2014; **75**(6): 430-45.
2307. Dimatelis JJ, Stein DJ, Russell VA. Behavioral changes after maternal separation are reversed by chronic constant light treatment. Brain Research. 2012; **1480**: 61-71.
2308. Dimitriou M, Rallidis LS, Theodoraki EV, Kalafati IP, Kolovou G, Dedoussis GV. Exclusive olive oil consumption has a protective effect on coronary artery disease; overview of the THISEAS study. Public Health Nutrition. 2016; **19**(6): 1081-7.
2309. Dionne FT, Turcotte L, Thibault MC, Boulay MR, Skinner JS, Bouchard C. Mitochondrial DNA sequence polymorphism, VO2max, and response to endurance training. Medicine And Science In Sports And Exercise. 1993; **25**(7): 766-74.
2310. Dionne IJ, Garant MJ, Nolan AA, Pollin TI, Lewis DG, Shuldiner AR, et al. Association between obesity and a polymorphism in the beta(1)-adrenoceptor gene (Gly389Arg ADRB1) in Caucasian women. International Journal Of Obesity And Related Metabolic Disorders: Journal Of The International Association For The Study Of Obesity. 2002; **26**(5): 633-9.
2311. Dionne IJ, Turner AN, Tchernof A, Pollin TI, Avrithi D, Gray D, et al. Identification of an interactive effect of beta3- and alpha2b-adrenoceptor gene polymorphisms on fat mass in Caucasian women. Diabetes. 2001; **50**(1): 91-5.
2312. Djarova T, Bardarev D, Boyanov D, Kaneva R, Atanasov P. Performance enhancing genetic variants, oxygen uptake, heart rate, blood pressure and body mass index of elite high altitude mountaineers. Acta Physiologica Hungarica. 2013; **100**(3): 289-301.
2313. Dlouhá D, Suchánek P, Lánská V, Hubáček JA. Body mass index change in females after short-time life style intervention is not dependent on the FTO polymorphisms. Physiological Research. 2011; **60**(1): 199-202.

2314. Do H-K, Lee J-H, Lim J-Y. Polydeoxyribonucleotide injection in the patients with partial-thickness tear of supraspinatus tendon: a prospective and pilot study using ultrasound. The Physician And Sportsmedicine. 2018; **46**(2): 213-20.
2315. Dodd JD, Barry SC, Barry RBM, Cawood TJ, McKenna MJ, Gallagher CG. Bone mineral density in cystic fibrosis: benefit of exercise capacity. Journal Of Clinical Densitometry: The Official Journal Of The International Society For Clinical Densitometry. 2008; **11**(4): 537-42.
2316. Doehring A, Freynhagen R, Griessinger N, Zimmermann M, Sittl R, Hentig Nv, et al. Cross-sectional assessment of the consequences of a GTP cyclohydrolase 1 haplotype for specialized tertiary outpatient pain care. The Clinical Journal Of Pain. 2009; **25**(9): 781-5.
2317. Domschke K, Dannlowski U, Hohoff C, Ohrmann P, Bauer J, Kugel H, et al. Neuropeptide Y (NPY) gene: Impact on emotional processing and treatment response in anxious depression. European Neuropsychopharmacology: The Journal Of The European College Of Neuropsychopharmacology. 2010; **20**(5): 301-9.
2318. Domschke K, Lawford B, Laje G, Berger K, Young R, Morris P, et al. Brain-derived neurotrophic factor (BDNF) gene: no major impact on antidepressant treatment response. The International Journal Of Neuropsychopharmacology. 2010; **13**(1): 93-101.
2319. Döring F, Onur S, Kürbitz C, Boulay MR, Pérusse L, Rankinen T, et al. Single nucleotide polymorphisms in the myostatin (MSTN) and muscle creatine kinase (CKM) genes are not associated with elite endurance performance. Scandinavian Journal Of Medicine & Science In Sports. 2011; **21**(6): 841-5.
2320. Doron R, Lotan D, Versano Z, Benatav L, Franko M, Armoza S, et al. Escitalopram or novel herbal mixture treatments during or following exposure to stress reduce anxiety-like behavior through corticosterone and BDNF modifications. PLoS One. 2014; **9**(4): e91455-e.
2321. dos Santos VA, Chatkin JM, Bau CHD, Paixão-Côrtes VR, Sun Y, Zamel N, et al. Glutamate and synaptic plasticity systems and smoking behavior: results from a genetic association study. PLoS One. 2012; **7**(6): e38666-e.
2322. Douet V, Tanizaki N, Franke A, Li X, Chang L. Polymorphism of Kynurenine Pathway-Related Genes, Kynurenic Acid, and Psychopathological Symptoms in HIV. Journal Of Neuroimmune Pharmacology: The Official Journal Of The Society On Neuroimmune Pharmacology. 2016; **11**(3): 549-61.
2323. Drozdovs'ka SB, Lysenko OM, Dosenko VI, Il'ïn VM, Moïbenko OO. [T(-786) --> C-polymorphism of the endothelial nitric oxide synthase promoter gene (eNOS) and exercise performance in sport]. Fiziolohichnyi Zhurnal (Kiev, Ukraine: 1994). 2013; **59**(6): 63-71.
2324. Duan Z, Chen G, Chen L, Stolzenberg-Solomon R, Weinstein SJ, Mannisto S, et al. Determinants of concentrations of N(epsilon)-carboxymethyl-lysine and soluble receptor for advanced glycation end products and their associations with risk of pancreatic cancer. Int J Mol Epidemiol Genet. 2014; **5**(3): 152-63.
2325. Duan Z, Chen G, Chen L, Stolzenberg-Solomon R, Weinstein SJ, Mannisto S, et al. Determinants of concentrations of N(ε)-carboxymethyl-lysine and soluble receptor for advanced glycation end products and their associations with risk of pancreatic cancer.

International Journal Of Molecular Epidemiology And Genetics. 2014; **5**(3): 152-63.
2326. Duman CH, Schlesinger L, Russell DS, Duman RS. Voluntary exercise produces antidepressant and anxiolytic behavioral effects in mice. Brain Research. 2008; **1199**: 148-58.
2327. Duzli Y, Grossman E, Gaides M, Segev S, Gal N, Ben-Dov I. Do angiotensin-converting enzyme (ACE) inhibitors enhance the effect of exercise rehabilitation in patients with hypertension and ACE DD and DI genotypes? Archives Of Physical Medicine And Rehabilitation. 2007; **88**(2): 262-4.
2328. Dzialanski Z, Barany M, Engfeldt P, Magnuson A, Olsson LA, Nilsson TK. Lactase persistence versus lactose intolerance: Is there an intermediate phenotype? Clinical Biochemistry. 2016; **49**(3): 248-52.
2329. Eaton RP, Sibbitt WL, Jr., Shah VO, Dorin RI, Zager PG, Bicknell JM. A commentary on 10 years of aldose reductase inhibition for limited joint mobility in diabetes. Journal Of Diabetes And Its Complications. 1998; **12**(1): 34-8.
2330. Eaves L, Silberg J, Erkanli A. Resolving multiple epigenetic pathways to adolescent depression. Journal Of Child Psychology And Psychiatry, And Allied Disciplines. 2003; **44**(7): 1006-14.
2331. Eberhard J, Ståhl O, Cohn-Cedermark G, Cavallin-Ståhl E, Giwercman Y, Rastkhani H, et al. Emotional disorders in testicular cancer survivors in relation to hypogonadism, androgen receptor polymorphism and treatment modality. Journal Of Affective Disorders. 2010; **122**(3): 260-6.
2332. Echeverria M, Robert D, Carde JP, Litvak S. Isolation from wheat mitochondria of a membrane-associated high molecular weight complex involved in DNA synthesis. Plant Molecular Biology. 1991; **16**(2): 301-15.
2333. Echeverry S, Shi XQ, Yang M, Huang H, Wu Y, Lorenzo LE, et al. Spinal microglia are required for long-term maintenance of neuropathic pain. Pain. 2017; **158**(9): 1792-801.
2334. Edwards TL, Velez Edwards DR, Villegas R, Cohen SS, Buchowski MS, Fowke JH, et al. HTR1B, ADIPOR1, PPARGC1A, and CYP19A1 and obesity in a cohort of Caucasians and African Americans: an evaluation of gene-environment interactions and candidate genes. American Journal Of Epidemiology. 2012; **175**(1): 11-21.
2335. Einat H, Yuan P, Gould TD, Li J, Du J, Zhang L, et al. The role of the extracellular signal-regulated kinase signaling pathway in mood modulation. The Journal Of Neuroscience: The Official Journal Of The Society For Neuroscience. 2003; **23**(19): 7311-6.
2336. El Yacoubi M, Ledent C, Parmentier M, Costentin J, Vaugeois JM. The anxiogenic-like effect of caffeine in two experimental procedures measuring anxiety in the mouse is not shared by selective A(2A) adenosine receptor antagonists. Psychopharmacology. 2000; **148**(2): 153-63.
2337. ElBatsh MM, Assareh N, Marsden CA, Kendall DA. Anxiogenic-like effects of chronic cannabidiol administration in rats. Psychopharmacology. 2012; **221**(2): 239-47.
2338. Eley TC, Hudson JL, Creswell C, Tropeano M, Lester KJ, Cooper P, et al. Therapygenetics: the 5HTTLPR and response to psychological therapy. Molecular Psychiatry. 2012; **17**(3): 236-7.
2339. Ellingrod VL, Taylor SF, Brook RD, Evans SJ, Zöllner SK, Grove TB, et al.

Dietary, lifestyle and pharmacogenetic factors associated with arteriole endothelial-dependent vasodilatation in schizophrenia patients treated with atypical antipsychotics (AAPs). Schizophrenia Research. 2011; **130**(1-3): 20-6.
2340. Engelmann R, Eggert M, Neeck G, Mueller-Hilke B. The impact of HLA-DRB alleles on the subclass titres of antibodies against citrullinated peptides. Rheumatology (Oxford, England). 2010; **49**(10): 1862-6.
2341. Erhardt A, Lucae S, Unschuld PG, Ising M, Kern N, Salyakina D, et al. Association of polymorphisms in P2RX7 and CaMKKb with anxiety disorders. Journal Of Affective Disorders. 2007; **101**(1-3): 159-68.
2342. Eussen SJPM, Vollset SE, Hustad S, Midttun Ø, Meyer K, Fredriksen A, et al. Plasma vitamins B2, B6, and B12, and related genetic variants as predictors of colorectal cancer risk. Cancer Epidemiology, Biomarkers & Prevention: A Publication Of The American Association For Cancer Research, Cosponsored By The American Society Of Preventive Oncology. 2010; **19**(10): 2549-61.
2343. Fabre LF, Harris RT. Pilot open-label study of lenperone (AHR 2277), a butyrophenone, in anxiety. Current Therapeutic Research, Clinical And Experimental. 1976; **19**(3): 328-31.
2344. Fahey B, Hickey B, Kelleher D, O'Dwyer A-M, O'Mara SM. The widely-used anti-viral drug interferon-alpha induces depressive- and anxiogenic-like effects in healthy rats. Behavioural Brain Research. 2007; **182**(1): 80-7.
2345. Fajemiroye JO, Adam K, Jordan K Z, Alves CE, Aderoju AA. Evaluation of Anxiolytic and Antidepressant-like Activity of Aqueous Leaf Extract of Nymphaea Lotus Linn. in Mice. Iranian Journal Of Pharmaceutical Research: IJPR. 2018; **17**(2): 613-26.
2346. Falcone M, Jepson C, Sanborn P, Cappella JN, Lerman C, Strasser AA. Association of BDNF and COMT genotypes with cognitive processing of anti-smoking PSAs. Genes, Brain, And Behavior. 2011; **10**(8): 862-7.
2347. Falkowski S, Woillard J-B, Postil D, Tubiana-Mathieu N, Terrebonne E, Pariente A, et al. Common variants in glucuronidation enzymes and membrane transporters as potential risk factors for colorectal cancer: a case control study. BMC Cancer. 2017; **17**(1): 901-.
2348. Fang Y, Qiu Q, Zhang S, Sun L, Li G, Xiao S, et al. Changes in miRNA-132 and miR-124 levels in non-treated and citalopram-treated patients with depression. Journal Of Affective Disorders. 2018; **227**: 745-51.
2349. Faruque MU, Millis RM, Dunston GM, Kwagyan J, Bond V, Jr., Rotimi CN, et al. Association of GNB3 C825T polymorphism with peak oxygen consumption. International Journal Of Sports Medicine. 2009; **30**(5): 315-9.
2350. Fatemi I, Heydari S, Kaeidi A, Shamsizadeh A, Hakimizadeh E, Khaluoi A, et al. Metformin ameliorates the age-related changes of D-galactose administration in ovariectomized mice. Fundam Clin Pharmacol. 2018.
2351. Fatemi I, Khaluoi A, Kaeidi A, Shamsizadeh A, Heydari S, Allahtavakoli MA. Protective effect of metformin on D-galactose-induced aging model in mice. Iran J Basic Med Sci. 2018; **21**(1): 19-25.
2352. Feairheller DL, Brown MD, Park J-Y, Brinkley TE, Basu S, Hagberg JM, et al. Exercise training, NADPH oxidase p22phox gene polymorphisms, and hypertension.

Medicine And Science In Sports And Exercise. 2009; **41**(7): 1421-8.
2353. Fedotovskaia ON, Popov DV, Vinogradova OL, Akhmetov II. [Association of the muscle-specific creatine kinase (CKMM) gene polymorphism with physical performance of athletes]. Fiziologiia Cheloveka. 2012; **38**(1): 105-9.
2354. Fehr C, Szegedi A, Anghelescu I, Klawe C, Hiemke C, Dahmen N. Sex differences in allelic frequencies of the 5-HT2C Cys23Ser polymorphism in psychiatric patients and healthy volunteers: findings from an association study. Psychiatric Genetics. 2000; **10**(2): 59-65.
2355. Feitosa MF, Gaskill SE, Rice T, Rankinen T, Bouchard C, Rao DC, et al. Major gene effects on exercise ventilatory threshold: the HERITAGE Family Study. Journal Of Applied Physiology (Bethesda, Md: 1985). 2002; **93**(3): 1000-6.
2356. Feldstein Ewing SW, Mead HK, Yezhuvath U, Dewitt S, Hutchison KE, Filbey FM. A preliminary examination of how serotonergic polymorphisms influence brain response following an adolescent cannabis intervention. Psychiatry Research. 2012; **204**(2-3): 112-6.
2357. Fernandes LMP, Cartágenes SC, Barros MA, Carvalheiro TCVS, Castro NCF, Schamne MG, et al. Repeated cycles of binge-like ethanol exposure induce immediate and delayed neurobehavioral changes and hippocampal dysfunction in adolescent female rats. Behavioural Brain Research. 2018; **350**: 99-108.
2358. Fernández-Real JM, Corella D, Goumidi L, Mercader JM, Valdés S, Rojo Martínez G, et al. Thyroid hormone receptor alpha gene variants increase the risk of developing obesity and show gene-diet interactions. International Journal Of Obesity (2005). 2013; **37**(11): 1499-505.
2359. Figler RA, Wang G, Srinivasan S, Jung DY, Zhang Z, Pankow JS, et al. Links between insulin resistance, adenosine A2B receptors, and inflammatory markers in mice and humans. Diabetes. 2011; **60**(2): 669-79.
2360. Filchak KE, Feder JL, Roethele JB, Stolz U, Mallet J. A FIELD TEST FOR HOST-PLANT DEPENDENT SELECTION ON LARVAE OF THE APPLE MAGGOT FLY, RHAGOLETIS POMONELLA. Evolution; International Journal Of Organic Evolution. 1999; **53**(1): 187-200.
2361. Filho PRM, Vercelino R, Cioato SG, Medeiros LF, de Oliveira C, Scarabelot VL, et al. Transcranial direct current stimulation (tDCS) reverts behavioral alterations and brainstem BDNF level increase induced by neuropathic pain model: Long-lasting effect. Progress In Neuro-Psychopharmacology & Biological Psychiatry. 2016; **64**: 44-51.
2362. Fite PJ, Brown S, Hossain W, Manzardo A, Butler MG, Bortolato M. Tobacco and cannabis use in college students are predicted by sex-dimorphic interactions between MAOA genotype and child abuse. CNS Neuroscience & Therapeutics. 2018.
2363. Fitzpatrick CR, Agrawal AA, Basiliko N, Hastings AP, Isaac ME, Preston M, et al. The importance of plant genotype and contemporary evolution for terrestrial ecosystem processes. Ecology. 2015; **96**(10): 2632-42.
2364. Flöel A, Ruscheweyh R, Krüger K, Willemer C, Winter B, Völker K, et al. Physical activity and memory functions: are neurotrophins and cerebral gray matter volume the missing link? Neuroimage. 2010; **49**(3): 2756-63.
2365. Flueck M. Myocellular limitations of human performance and their modification through genome-dependent responses at altitude. Experimental Physiology. 2010;

95(3): 451-62.
2366. Foley AR, Menezes MP, Pandraud A, Gonzalez MA, Al-Odaib A, Abrams AJ, et al. Treatable childhood neuronopathy caused by mutations in riboflavin transporter RFVT2. Brain: A Journal Of Neurology. 2014; **137**(Pt 1): 44-56.
2367. Fontaine-Bisson B, Thorburn J, Gregory A, Zhang H, Sun G. Melanin-concentrating hormone receptor 1 polymorphisms are associated with components of energy balance in the Complex Diseases in the Newfoundland Population: Environment and Genetics (CODING) study. The American Journal Of Clinical Nutrition. 2014; **99**(2): 384-91.
2368. Foraita R, Günther F, Gwozdz W, Reisch LA, Russo P, Lauria F, et al. Does the FTO gene interact with the socioeconomic status on the obesity development among young European children? Results from the IDEFICS study. International Journal Of Obesity (2005). 2015; **39**(1): 1-6.
2369. Forti LN, Van Roie E, Njemini R, Coudyzer W, Beyer I, Delecluse C, et al. Dose- and gender-specific effects of resistance training on circulating levels of brain derived neurotrophic factor (BDNF) in community-dwelling older adults. Experimental Gerontology. 2015; **70**: 144-9.
2370. Foschini D, Araújo RC, Bacurau RFP, De Piano A, De Almeida SS, Carnier J, et al. Treatment of obese adolescents: the influence of periodization models and ACE genotype. Obesity (Silver Spring, Md). 2010; **18**(4): 766-72.
2371. Fowke JH, Chung F-L, Jin F, Qi D, Cai Q, Conaway C, et al. Urinary isothiocyanate levels, brassica, and human breast cancer. Cancer Research. 2003; **63**(14): 3980-6.
2372. Fowke JH, Shu X-O, Dai Q, Shintani A, Conaway CC, Chung F-L, et al. Urinary isothiocyanate excretion, brassica consumption, and gene polymorphisms among women living in Shanghai, China. Cancer Epidemiology, Biomarkers & Prevention: A Publication Of The American Association For Cancer Research, Cosponsored By The American Society Of Preventive Oncology. 2003; **12**(12): 1536-9.
2373. Francès F, Guillen M, Verdú F, Portolés O, Castelló A, Sorlí JV, et al. The 1258 G>A polymorphism in the neuropeptide Y gene is associated with greater alcohol consumption in a Mediterranean population. Alcohol (Fayetteville, NY). 2011; **45**(2): 131-6.
2374. Franks PW, Barroso I, Luan Ja, Ekelund U, Crowley VEF, Brage S, et al. PGC-1alpha genotype modifies the association of volitional energy expenditure with [OV0312]O2max. Medicine And Science In Sports And Exercise. 2003; **35**(12): 1998-2004.
2375. Franks PW, Ravussin E, Hanson RL, Harper IT, Allison DB, Knowler WC, et al. Habitual physical activity in children: the role of genes and the environment. The American Journal Of Clinical Nutrition. 2005; **82**(4): 901-8.
2376. Frederiksen H, Bathum L, Worm C, Christensen K, Puggaard L. ACE genotype and physical training effects: a randomized study among elderly Danes. Aging Clinical And Experimental Research. 2003; **15**(4): 284-91.
2377. Fuentes RM, Perola M, Nissinen A, Tuomilehto J. ACE gene and physical activity, blood pressure, and hypertension: a population study in Finland. Journal Of Applied Physiology (Bethesda, Md: 1985). 2002; **92**(6): 2508-12.

2378. Fuku N, Murakami H, Iemitsu M, Sanada K, Tanaka M, Miyachi M. Mitochondrial macrohaplogroup associated with muscle power in healthy adults. International Journal Of Sports Medicine. 2012; **33**(5): 410-4.
2379. Fukuda K-i, Hayashida M, Ikeda K, Koukita Y, Ichinohe T, Kaneko Y. Diversity of opioid requirements for postoperative pain control following oral surgery--is it affected by polymorphism of the μ-opioid receptor? Anesthesia Progress. 2010; **57**(4): 145-9.
2380. Furiga A, Pierre G, Glories M, Aimar P, Roques C, Causserand C, et al. Effects of ionic strength on bacteriophage MS2 behavior and their implications for the assessment of virus retention by ultrafiltration membranes. Applied And Environmental Microbiology. 2011; **77**(1): 229-36.
2381. Furmark T. Neurobiological aspects of social anxiety disorder. The Israel Journal Of Psychiatry And Related Sciences. 2009; **46**(1): 5-12.
2382. Furmark T, Appel L, Henningsson S, Ahs F, Faria V, Linnman C, et al. A link between serotonin-related gene polymorphisms, amygdala activity, and placebo-induced relief from social anxiety. The Journal Of Neuroscience: The Official Journal Of The Society For Neuroscience. 2008; **28**(49): 13066-74.
2383. Galdiano RF, Jr., de Macedo Lemos EG, de Faria RT, Vendrame WA. Seedling development and evaluation of genetic stability of cryopreserved Dendrobium hybrid mature seeds. Applied Biochemistry And Biotechnology. 2014; **172**(5): 2521-9.
2384. Gale P, Hill A, Kelly L, Bassett J, McClure P, Le Marc Y, et al. Applications of omics approaches to the development of microbiological risk assessment using RNA virus dose-response models as a case study. Journal Of Applied Microbiology. 2014; **117**(6): 1537-48.
2385. Ganguli M, Chandra V, Kamboh MI, Johnston JM, Dodge HH, Thelma BK, et al. Apolipoprotein E polymorphism and Alzheimer disease: The Indo-US Cross-National Dementia Study. Archives Of Neurology. 2000; **57**(6): 824-30.
2386. Garaulet M, Smith CE, Hernandez-Gonzalez T, Lee YC, Ordovas JM. PPARgamma Pro12Ala interacts with fat intake for obesity and weight loss in a behavioural treatment based on the Mediterranean diet. Mol Nutr Food Res. 2011; **55**(12): 1771-9.
2387. Garaulet M, Smith CE, Hernández-González T, Lee Y-C, Ordovás JM. PPARγ Pro12Ala interacts with fat intake for obesity and weight loss in a behavioural treatment based on the Mediterranean diet. Molecular Nutrition & Food Research. 2011; **55**(12): 1771-9.
2388. Garcia LSB, Comim CM, Valvassori SS, Réus GZ, Stertz L, Kapczinski F, et al. Ketamine treatment reverses behavioral and physiological alterations induced by chronic mild stress in rats. Progress In Neuro-Psychopharmacology & Biological Psychiatry. 2009; **33**(3): 450-5.
2389. Garcia-Garcia MR, Morales-Lanuza MA, Campos-Perez WY, Ruiz-Madrigal B, Maldonado-Gonzalez M, Vizmanos B, et al. Effect of the ADIPOQ Gene -11391G/A Polymorphism Is Modulated by Lifestyle Factors in Mexican Subjects. Journal Of Nutrigenetics And Nutrigenomics. 2014; **7**(4-6): 212-24.
2390. Garcia-Rios A, Gomez-Delgado FJ, Garaulet M, Alcala-Diaz JF, Delgado-Lista FJ, Marin C, et al. Beneficial effect of CLOCK gene polymorphism rs1801260 in combination with low-fat diet on insulin metabolism in the patients with metabolic

syndrome. Chronobiology International. 2014; **31**(3): 401-8.
2391. Garfield LD, Dixon D, Nowotny P, Lotrich FE, Pollock BG, Kristjansson SD, et al. Common selective serotonin reuptake inhibitor side effects in older adults associated with genetic polymorphisms in the serotonin transporter and receptors: data from a randomized controlled trial. The American Journal Of Geriatric Psychiatry: Official Journal Of The American Association For Geriatric Psychiatry. 2014; **22**(10): 971-9.
2392. Gari MA, AlKaff M, Alsehli HS, Dallol A, Gari A, Abu-Elmagd M, et al. Identification of novel genetic variations affecting osteoarthritis patients. BMC Medical Genetics. 2016; **17**(Suppl 1): 68-.
2393. Gashaw I, Kirchheiner J, Goldammer M, Bauer S, Seidemann J, Zoller K, et al. Cytochrome p450 3A4 messenger ribonucleic acid induction by rifampin in human peripheral blood mononuclear cells: correlation with alprazolam pharmacokinetics. Clinical Pharmacology And Therapeutics. 2003; **74**(5): 448-57.
2394. Gaskill SE, Rice T, Bouchard C, Gagnon J, Rao DC, Skinner JS, et al. Familial resemblance in ventilatory threshold: the HERITAGE Family Study. Medicine And Science In Sports And Exercise. 2001; **33**(11): 1832-40.
2395. Gassó P, Rodríguez N, Blázquez A, Monteagudo A, Boloc D, Plana MT, et al. Epigenetic and genetic variants in the HTR1B gene and clinical improvement in children and adolescents treated with fluoxetine. Progress In Neuro-Psychopharmacology & Biological Psychiatry. 2017; **75**: 28-34.
2396. Gebreab SY, Manna ZG, Khan RJ, Riestra P, Xu R, Davis SK. Less Than Ideal Cardiovascular Health Is Associated With Shorter Leukocyte Telomere Length: The National Health and Nutrition Examination Surveys, 1999-2002. Journal Of The American Heart Association. 2017; **6**(2).
2397. Gee DG, Fetcho RN, Jing D, Li A, Glatt CE, Drysdale AT, et al. Individual differences in frontolimbic circuitry and anxiety emerge with adolescent changes in endocannabinoid signaling across species. Proceedings Of The National Academy Of Sciences Of The United States Of America. 2016; **113**(16): 4500-5.
2398. Gehrmann J, Siegler D, Ignacy E, Reimer I. [Narcolepsy in childhood and adolescence: symptoms, diagnosis, and therapy. A case report]. Zeitschrift Fur Kinder-Und Jugendpsychiatrie Und Psychotherapie. 2017; **45**(2): 149-57.
2399. Gelegen C, van den Heuvel J, Collier DA, Campbell IC, Oppelaar H, Hessel E, et al. Dopaminergic and brain-derived neurotrophic factor signalling in inbred mice exposed to a restricted feeding schedule. Genes, Brain, And Behavior. 2008; **7**(5): 552-9.
2400. Gelineau RR, Arruda NL, Hicks JA, Monteiro De Pina I, Hatzidis A, Seggio JA. The behavioral and physiological effects of high-fat diet and alcohol consumption: Sex differences in C57BL6/J mice. Brain Behav. 2017; **7**(6): e00708.
2401. Gersner R, Toth E, Isserles M, Zangen A. Site-specific antidepressant effects of repeated subconvulsive electrical stimulation: potential role of brain-derived neurotrophic factor. Biological Psychiatry. 2010; **67**(2): 125-32.
2402. Ghosh S, Vivar JC, Sarzynski MA, Sung YJ, Timmons JA, Bouchard C, et al. Integrative pathway analysis of a genome-wide association study of (V)O(2max) response to exercise training. Journal Of Applied Physiology (Bethesda, Md: 1985). 2013; **115**(9): 1343-59.

2403. Glazer L, Hahn ME, Aluru N. Delayed effects of developmental exposure to low levels of the aryl hydrocarbon receptor agonist 3,3',4,4',5-pentachlorobiphenyl (PCB126) on adult zebrafish behavior. Neurotoxicology. 2016; **52**: 134-43.
2404. Gnad T, Scheibler S, von Kügelgen I, Scheele C, Kilić A, Glöde A, et al. Adenosine activates brown adipose tissue and recruits beige adipocytes via A2A receptors. Nature. 2014; **516**(7531): 395-9.
2405. Goldfield GS, Kenny GP, Prud'homme D, Holcik M, Alberga AS, Fahnestock M, et al. Effects of aerobic training, resistance training, or both on brain-derived neurotrophic factor in adolescents with obesity: The hearty randomized controlled trial. Physiology & Behavior. 2018; **191**: 138-45.
2406. Goldman D, Oroszi G, O'Malley S, Anton R. COMBINE genetics study: the pharmacogenetics of alcoholism treatment response: genes and mechanisms. Journal Of Studies On Alcohol Supplement. 2005; (15): 56-64.
2407. Gomes WF, Lacerda ACR, Mendonça VA, Arrieiro AN, Fonseca SF, Amorim MR, et al. Effect of exercise on the plasma BDNF levels in elderly women with knee osteoarthritis. Rheumatology International. 2014; **34**(6): 841-6.
2408. Gómez-Gallego F, Santiago C, Morán M, Pérez M, Maté-Muñoz JL, del Valle MF, et al. The I allele of the ACE gene is associated with improved exercise capacity in women with McArdle disease. British Journal Of Sports Medicine. 2008; **42**(2): 134-40.
2409. Goni L, Cuervo M, Milagro FI, Martinez JA. A genetic risk tool for obesity predisposition assessment and personalized nutrition implementation based on macronutrient intake. Genes Nutr. 2015; **10**(1): 445.
2410. Goni L, García-Granero M, Milagro FI, Cuervo M, Martínez JA. Phenotype and genotype predictors of BMI variability among European adults. Nutrition & Diabetes. 2018; **8**(1): 27-.
2411. Gonzales TK, Yonker JA, Chang V, Roan CL, Herd P, Atwood CS. Myocardial infarction in the Wisconsin Longitudinal Study: the interaction among environmental, health, social, behavioural and genetic factors. BMJ Open. 2017; **7**(1): e011529-e.
2412. Goodrich GG, Goodman PH, Budhecha SK, Pritsos CA. Functional polymorphism of detoxification gene NQO1 predicts intensity of empirical treatment of childhood asthma. Mutation Research. 2009; **674**(1-2): 55-61.
2413. Gosadi IM, Goyder EC, Teare MD. Investigating the potential effect of consanguinity on type 2 diabetes susceptibility in a Saudi population. Human Heredity. 2014; **77**(1-4): 197-206.
2414. Götschel F, Berg D, Gruber W, Bender C, Eberl M, Friedel M, et al. Synergism between Hedgehog-GLI and EGFR signaling in Hedgehog-responsive human medulloblastoma cells induces downregulation of canonical Hedgehog-target genes and stabilized expression of GLI1. PLoS One. 2013; **8**(6): e65403-e.
2415. Gozdzik A, Zhu J, Wong BYL, Fu L, Cole DEC, Parra EJ. Association of vitamin D binding protein (VDBP) polymorphisms and serum 25(OH)D concentrations in a sample of young Canadian adults of different ancestry. The Journal Of Steroid Biochemistry And Molecular Biology. 2011; **127**(3-5): 405-12.
2416. Gragnoli C. Proteasome modulator 9 gene SNPs, responsible for anti-depressant response, are in linkage with generalized anxiety disorder. Journal Of Cellular Physiology. 2014; **229**(9): 1157-9.

2417. Grarup N, Andreasen CH, Andersen MK, Albrechtsen A, Sandbaek A, Lauritzen T, et al. The -250G>A promoter variant in hepatic lipase associates with elevated fasting serum high-density lipoprotein cholesterol modulated by interaction with physical activity in a study of 16,156 Danish subjects. The Journal Of Clinical Endocrinology And Metabolism. 2008; **93**(6): 2294-9.
2418. Greenblatt DJ, von Moltke LL, Harmatz JS, Ciraulo DA, Shader RI. Alprazolam pharmacokinetics, metabolism, and plasma levels: clinical implications. The Journal Of Clinical Psychiatry. 1993; **54 Suppl**: 4-11.
2419. Gregg EW, Kriska AM, Salamone LM, Wolf RL, Roberts MM, Ferrell RE, et al. Correlates of quantitative ultrasound in the Women's Healthy Lifestyle Project. Osteoporosis International: A Journal Established As Result Of Cooperation Between The European Foundation For Osteoporosis And The National Osteoporosis Foundation Of The USA. 1999; **10**(5): 416-24.
2420. Grimnes G, Emaus N, Cashman KD, Jorde R. The effect of high-dose vitamin D supplementation on muscular function and quality of life in postmenopausal women-A randomized controlled trial. Clinical Endocrinology. 2017; **87**(1): 20-8.
2421. Grone BP, Qu T, Baraban SC. Behavioral Comorbidities and Drug Treatments in a Zebrafish scn1lab Model of Dravet Syndrome. Eneuro. 2017; **4**(4).
2422. Gu B, Huang YZ, He XP, Joshi RB, Jang W, McNamara JO. A Peptide Uncoupling BDNF Receptor TrkB from Phospholipase Cgamma1 Prevents Epilepsy Induced by Status Epilepticus. Neuron. 2015; **88**(3): 484-91.
2423. Gu B, Huang YZ, He X-P, Joshi RB, Jang W, McNamara JO. A Peptide Uncoupling BDNF Receptor TrkB from Phospholipase Cγ1 Prevents Epilepsy Induced by Status Epilepticus. Neuron. 2015; **88**(3): 484-91.
2424. Guclu-Geyik F, Onat A, Coban N, Komurcu-Bayrak E, Sansoy V, Can G, et al. Minor allele of the APOA4 gene T347S polymorphism predisposes to obesity in postmenopausal Turkish women. Molecular Biology Reports. 2012; **39**(12): 10907-14.
2425. Guclu-Geyik F, Onat A, Yuzbasıogulları AB, Coban N, Can G, Lehtimäki T, et al. Risk of obesity and metabolic syndrome associated with FTO gene variants discloses clinically relevant gender difference among Turks. Molecular Biology Reports. 2016; **43**(6): 485-94.
2426. Gudayol-Ferré E, Herrera-Guzmán I, Camarena B, Cortés-Penagos C, Herrera-Abarca JE, Martínez-Medina P, et al. The role of clinical variables, neuropsychological performance and SLC6A4 and COMT gene polymorphisms on the prediction of early response to fluoxetine in major depressive disorder. Journal Of Affective Disorders. 2010; **127**(1-3): 343-51.
2427. Gudnason V, Stansbie D, Scott J, Bowron A, Nicaud V, Humphries S. C677T (thermolabile alanine/valine) polymorphism in methylenetetrahydrofolate reductase (MTHFR): its frequency and impact on plasma homocysteine concentration in different European populations. EARS group. Atherosclerosis. 1998; **136**(2): 347-54.
2428. Guillén M, Corella D, Portolés O, González JI, Mulet F, Sáiz C. Prevalence of the methylenetetrahydrofolate reductase 677C > T mutation in the Mediterranean Spanish population. Association with cardiovascular risk factors. European Journal Of Epidemiology. 2001; **17**(3): 255-61.
2429. Guimaraes HC, Cascardo JL, Beato RG, Barbosa MT, Machado TH, de Almeida

MA, et al. Features associated with cognitive impairment and dementia in a community-based sample of illiterate elderly aged 75+ years: the Pieta study. Dement Neuropsychol. 2014; **8**(2): 126-31.
2430. Guimarães HC, Cascardo JL, Beato RG, Barbosa MT, Machado TH, de Almeida MA, et al. Features associated with cognitive impairment and dementia in a community-based sample of illiterate elderly aged 75+ years: the Pietà study. Dementia & Neuropsychologia. 2014; **8**(2): 126-31.
2431. Gupta V, Vinay DG, Sovio U, Rafiq S, Kranthi Kumar MV, Janipalli CS, et al. Association study of 25 type 2 diabetes related Loci with measures of obesity in Indian sib pairs. PLoS One. 2013; **8**(1): e53944-e.
2432. Hagberg JM, Zmuda JM, McCole SD, Rodgers KS, Ferrell RE, Wilund KR, et al. Moderate physical activity is associated with higher bone mineral density in postmenopausal women. Journal Of The American Geriatrics Society. 2001; **49**(11): 1411-7.
2433. Hai B, Ni C, Xie H, Guo Z, Wu M, Chen Q, et al. [Association between peroxisome proliferator-activated receptor and gene-gene interactions with the apolipoprotein A I/apolipoprotein B100 ratio]. Zhonghua Xin Xue Guan Bing Za Zhi. 2015; **43**(4): 328-33.
2434. Haile CN, Murrough JW, Iosifescu DV, Chang LC, Al Jurdi RK, Foulkes A, et al. Plasma brain derived neurotrophic factor (BDNF) and response to ketamine in treatment-resistant depression. The International Journal Of Neuropsychopharmacology. 2014; **17**(2): 331-6.
2435. Hama Y, Mori-Yoshimura M, Komaki H, Suzuki S, Kohsaka H, Nishino I, et al. Childhood-onset anti-3-hydroxy-3-methylglutaryl-coenzyme A reductase (anti-HMGCR) necrotizing myopathy needs to be distinguished from muscular dystrophy: A case study. Rinsho Shinkeigaku = Clinical Neurology. 2017; **57**(10): 567-72.
2436. Hambsch B, Chen B-G, Brenndörfer J, Meyer M, Avrabos C, Maccarrone G, et al. Methylglyoxal-mediated anxiolysis involves increased protein modification and elevated expression of glyoxalase 1 in the brain. Journal Of Neurochemistry. 2010; **113**(5): 1240-51.
2437. Hamel P, Simoneau JA, Lortie G, Boulay MR, Bouchard C. Heredity and muscle adaptation to endurance training. Medicine And Science In Sports And Exercise. 1986; **18**(6): 690-6.
2438. Hamilton SP, Heiman GA, Haghighi F, Mick S, Klein DF, Hodge SE, et al. Lack of genetic linkage or association between a functional serotonin transporter polymorphism and panic disorder. Psychiatric Genetics. 1999; **9**(1): 1-6.
2439. Han L, Ma W, Sun D, Heianza Y, Wang T, Zheng Y, et al. Genetic variation of habitual coffee consumption and glycemic changes in response to weight-loss diet intervention: the Preventing Overweight Using Novel Dietary Strategies (POUNDS LOST) trial. The American Journal Of Clinical Nutrition. 2017; **106**(5): 1321-6.
2440. Hand BD, McCole SD, Brown MD, Park JJ, Ferrell RE, Huberty A, et al. NOS3 gene polymorphisms and exercise hemodynamics in postmenopausal women. International Journal Of Sports Medicine. 2006; **27**(12): 951-8.
2441. Happi TC, Thomas SM, Gbotosho GO, Falade CO, Akinboye DO, Gerena L, et al. Point mutations in the pfcrt and pfmdr-1 genes of Plasmodium falciparum and clinical

response to chloroquine, among malaria patients from Nigeria. Annals Of Tropical Medicine And Parasitology. 2003; **97**(5): 439-51.
2442. Harada N, Hatakeyama A, Okuyama M, Miyatake Y, Nakagawa T, Kuroda M, et al. Readthrough of ACTN3 577X nonsense mutation produces full-length α-actinin-3 protein. Biochemical And Biophysical Research Communications. 2018; **502**(3): 422-8.
2443. Haram M, Tesli M, Dieset I, Steen NE, Røssberg JI, Djurovic S, et al. An attempt to identify single nucleotide polymorphisms contributing to possible relationships between personality traits and oxytocin-related genes. Neuropsychobiology. 2014; **69**(1): 25-30.
2444. Harbron J, van der Merwe L, Zaahl MG, Kotze MJ, Senekal M. Fat mass and obesity-associated (FTO) gene polymorphisms are associated with physical activity, food intake, eating behaviors, psychological health, and modeled change in body mass index in overweight/obese Caucasian adults. Nutrients. 2014; **6**(8): 3130-52.
2445. Haring R, Ernst F, Schurmann C, Homuth G, Völker U, Völzke H, et al. The androgen receptor CAG repeat polymorphism as a risk factor of low serum testosterone and its cardiometabolic effects in men. International Journal Of Andrology. 2012; **35**(4): 511-20.
2446. Hartley CA, McKenna MC, Salman R, Holmes A, Casey BJ, Phelps EA, et al. Serotonin transporter polyadenylation polymorphism modulates the retention of fear extinction memory. Proceedings Of The National Academy Of Sciences Of The United States Of America. 2012; **109**(14): 5493-8.
2447. Harvey BG, Hackett NR, Ely S, Crystal RG. Host responses and persistence of vector genome following intrabronchial administration of an E1(-)E3(-) adenovirus gene transfer vector to normal individuals. Molecular Therapy: The Journal Of The American Society Of Gene Therapy. 2001; **3**(2): 206-15.
2448. Hasler G, Drevets WC, Gould TD, Gottesman II, Manji HK. Toward constructing an endophenotype strategy for bipolar disorders. Biological Psychiatry. 2006; **60**(2): 93-105.
2449. Hasselbalch AL. Genetics of dietary habits and obesity - a twin study. Danish Medical Bulletin. 2010; **57**(9): B4182-B.
2450. He Z, Hu Y, Feng L, Bao D, Wang L, Li Y, et al. Is there an association between PPARGC1A genotypes and endurance capacity in Chinese men? Scandinavian Journal Of Medicine & Science In Sports. 2008; **18**(2): 195-204.
2451. He Z, Hu Y, Feng L, Li Y, Liu G, Xi Y, et al. NRF-1 genotypes and endurance exercise capacity in young Chinese men. British Journal Of Sports Medicine. 2008; **42**(5): 361-6.
2452. He Z, Hu Y, Feng L, Lu Y, Liu G, Xi Y, et al. NRF2 genotype improves endurance capacity in response to training. International Journal Of Sports Medicine. 2007; **28**(9): 717-21.
2453. He Z, Hu Y, Feng L, Lu Y, Liu G, Xi Y, et al. Polymorphisms in the HBB gene relate to individual cardiorespiratory adaptation in response to endurance training. British Journal Of Sports Medicine. 2006; **40**(12): 998-1002.
2454. He ZH, Ma LH. The aerobic fitness (VO2 peak) and alpha-fibrinogen genetic polymorphism in obese and non-obese Chinese boys. International Journal Of Sports Medicine. 2005; **26**(4): 253-7.

2455. He Z-H, Hu Y, Wang H-Y, Li Y-C, Lu Y-L, Zhang L, et al. Are calcineurin genes associated with endurance phenotype traits? European Journal Of Applied Physiology. 2010; **109**(3): 359-69.
2456. Heberlein A, Büscher P, Schuster R, Kleimann A, Lichtinghagen R, Rhein M, et al. Do changes in the BDNF promoter methylation indicate the risk of alcohol relapse? European Neuropsychopharmacology: The Journal Of The European College Of Neuropsychopharmacology. 2015; **25**(11): 1892-7.
2457. Heberlein A, Käser M, Lichtinghagen R, Rhein M, Lenz B, Kornhuber J, et al. TNF-α and IL-6 serum levels: neurobiological markers of alcohol consumption in alcohol-dependent patients? Alcohol (Fayetteville, NY). 2014; **48**(7): 671-6.
2458. Hedman E, Andersson E, Ljótsson B, Andersson G, Schalling M, Lindefors N, et al. Clinical and genetic outcome determinants of Internet- and group-based cognitive behavior therapy for social anxiety disorder. Acta Psychiatrica Scandinavica. 2012; **126**(2): 126-36.
2459. Hegarty D, Shorten G. Multivariate prognostic modeling of persistent pain following lumbar discectomy. Pain Physician. 2012; **15**(5): 421-34.
2460. Heid IM, Vollmert C, Kronenberg F, Huth C, Ankerst DP, Luchner A, et al. Association of the MC4R V103I polymorphism with the metabolic syndrome: the KORA Study. Obesity (Silver Spring, Md). 2008; **16**(2): 369-76.
2461. Heilig M, Zachrisson O, Thorsell A, Ehnvall A, Mottagui-Tabar S, Sjögren M, et al. Decreased cerebrospinal fluid neuropeptide Y (NPY) in patients with treatment refractory unipolar major depression: preliminary evidence for association with preproNPY gene polymorphism. Journal Of Psychiatric Research. 2004; **38**(2): 113-21.
2462. Heiman-Patterson TD, Argov Z, Chavin JM, Kalman B, Alder H, DiMauro S, et al. Biochemical and genetic studies in a family with mitochondrial myopathy. Muscle & Nerve. 1997; **20**(10): 1219-24.
2463. Heitland I, Klumpers F, Oosting RS, Evers DJJ, Leon Kenemans J, Baas JMP. Failure to extinguish fear and genetic variability in the human cannabinoid receptor 1. Translational Psychiatry. 2012; **2**: e162-e.
2464. Heller J, Mirzazade S, Romanzetti S, Habel U, Derntl B, Freitag NM, et al. Impact of gender and genetics on emotion processing in Parkinson's disease - A multimodal study. Neuroimage Clinical. 2018; **18**: 305-14.
2465. Herbeth B, Gueguen S, Leroy P, Siest G, Visvikis-Siest S. The lipoprotein lipase serine 447 stop polymorphism is associated with altered serum carotenoid concentrations in the Stanislas Family Study. Journal Of The American College Of Nutrition. 2007; **26**(6): 655-62.
2466. Herzog H, Elmenhorst D, Winz O, Bauer A. Biodistribution and radiation dosimetry of the A1 adenosine receptor ligand 18F-CPFPX determined from human whole-body PET. European Journal Of Nuclear Medicine And Molecular Imaging. 2008; **35**(8): 1499-506.
2467. Hettema JM, Chen X, Sun C, Brown TA. Direct, indirect and pleiotropic effects of candidate genes on internalizing disorder psychopathology. Psychological Medicine. 2015; **45**(10): 2227-36.
2468. Heuser K, Taubøll E, Nagelhus EA, Cvancarova M, Petter Ottersen O, Gjerstad L. Phenotypic characteristics of temporal lobe epilepsy: the impact of hippocampal

sclerosis. Acta Neurologica Scandinavica Supplementum. 2009; (189): 8-13.
2469. Heyer NJ, Echeverria D, Farin FM, Woods JS. The association between serotonin transporter gene promoter polymorphism (5-HTTLPR), self-reported symptoms, and dental mercury exposure. Journal Of Toxicology And Environmental Health Part A. 2008; **71**(19): 1318-26.
2470. Ho GY, Burk RD, Fleming I, Klein RS. Risk of genital human papillomavirus infection in women with human immunodeficiency virus-induced immunosuppression. International Journal Of Cancer. 1994; **56**(6): 788-92.
2471. Hoffman JR, Cohen H, Ostfeld I, Kaplan Z, Zohar J, Cohen H. Exercise Maintains Dendritic Complexity in an Animal Model of Posttraumatic Stress Disorder. Medicine And Science In Sports And Exercise. 2016; **48**(12): 2487-94.
2472. Hohoff C, McDonald JM, Baune BT, Cook EH, Deckert J, de Wit H. Interindividual variation in anxiety response to amphetamine: possible role for adenosine A2A receptor gene variants. American Journal Of Medical Genetics Part B, Neuropsychiatric Genetics: The Official Publication Of The International Society Of Psychiatric Genetics. 2005; **139B**(1): 42-4.
2473. Holdys J, Gronek P, Kryściak J, Stanisławski D. Genetic variants of uncoupling proteins-2 and -3 in relation to maximal oxygen uptake in different sports. Acta Biochimica Polonica. 2013; **60**(1): 71-5.
2474. Holm KE, Plaufcan MR, Ford DW, Sandhaus RA, Strand M, Strange C, et al. The impact of age on outcomes in chronic obstructive pulmonary disease differs by relationship status. Journal Of Behavioral Medicine. 2014; **37**(4): 654-63.
2475. Holopainen T, Räsänen M, Anisimov A, Tuomainen T, Zheng W, Tvorogov D, et al. Endothelial Bmx tyrosine kinase activity is essential for myocardial hypertrophy and remodeling. Proceedings Of The National Academy Of Sciences Of The United States Of America. 2015; **112**(42): 13063-8.
2476. Hou L, Guo Y, Lian B, Wang Y, Li C, Wang G, et al. Synaptic Ultrastructure Might Be Involved in HCN1-Related BDNF mRNA in Withdrawal-Anxiety After Ethanol Dependence. Frontiers In Psychiatry. 2018; **9**: 215-.
2477. Houlahan KE, Prokopec SD, Sun RX, Moffat ID, Lindén J, Lensu S, et al. Transcriptional profiling of rat white adipose tissue response to 2,3,7,8-tetrachlorodibenzo-ρ-dioxin. Toxicology And Applied Pharmacology. 2015; **288**(2): 223-31.
2478. Hoyle E, Genn RF, Fernandes C, Stolerman IP. Impaired performance of alpha7 nicotinic receptor knockout mice in the five-choice serial reaction time task. Psychopharmacology. 2006; **189**(2): 211-23.
2479. Huang L, Cai X, Lian F, Zhang L, Kong Y, Cao C, et al. Interactions between ALDH2 rs671 polymorphism and lifestyle behaviors on coronary artery disease risk in a Chinese Han population with dyslipidemia: A guide to targeted heart health management. Environmental Health And Preventive Medicine. 2018; **23**(1): 29-.
2480. Huang X, Wang Z, Wu Y, Fan T, Wang S, Wang X. Variety of molecular conformation of plasmid pUC18 DNA and solenoidally supercoiled DNA. Science In China Series C, Life Sciences. 1996; **39**(6): 571-83.
2481. Huang X-F, Jiang W-T, Liu L, Song F-C, Zhu X, Shi G-L, et al. A novel PDE9 inhibitor WYQ-C36D ameliorates corticosterone-induced neurotoxicity and depression-

like behaviors by cGMP-CREB-related signaling. CNS Neuroscience & Therapeutics. 2018.
2482. Huang Z, Hoffman CA, Chelette BM, Thiebaud N, Fadool DA. Elevated Anxiety and Impaired Attention in Super-Smeller, Kv1.3 Knockout Mice. Frontiers In Behavioral Neuroscience. 2018; **12**: 49-.
2483. Hubáček JA, Pikhart H, Peasey A, Kubínová R, Bobák M. FTO variant, energy intake, physical activity and basal metabolic rate in Caucasians. The HAPIEE study. Physiological Research. 2011; **60**(1): 175-83.
2484. Hudson JL, Lester KJ, Lewis CM, Tropeano M, Creswell C, Collier DA, et al. Predicting outcomes following cognitive behaviour therapy in child anxiety disorders: the influence of genetic, demographic and clinical information. Journal Of Child Psychology And Psychiatry, And Allied Disciplines. 2013; **54**(10): 1086-94.
2485. Hulmi JJ, Isola V, Suonpää M, Järvinen NJ, Kokkonen M, Wennerström A, et al. The Effects of Intensive Weight Reduction on Body Composition and Serum Hormones in Female Fitness Competitors. Frontiers In Physiology. 2017; **7**: 689-.
2486. Hulmi JJ, Nissinen TA, Räsänen M, Degerman J, Lautaoja JH, Hemanthakumar KA, et al. Prevention of chemotherapy-induced cachexia by ACVR2B ligand blocking has different effects on heart and skeletal muscle. Journal Of Cachexia, Sarcopenia And Muscle. 2018; **9**(2): 417-32.
2487. Huuskonen A, Lappalainen J, Tanskanen M, Oksala N, Kyröläinen H, Atalay M. Genetic variations of leptin and leptin receptor are associated with body composition changes in response to physical training. Cell Biochemistry And Function. 2010; **28**(4): 306-12.
2488. Hwang J, Castelli DM, Gonzalez-Lima F. The positive cognitive impact of aerobic fitness is associated with peripheral inflammatory and brain-derived neurotrophic biomarkers in young adults. Physiology & Behavior. 2017; **179**: 75-89.
2489. Ibáñez-Sanz G, Díez-Villanueva A, Alonso MH, Rodríguez-Moranta F, Pérez-Gómez B, Bustamante M, et al. Risk Model for Colorectal Cancer in Spanish Population Using Environmental and Genetic Factors: Results from the MCC-Spain study. Scientific Reports. 2017; **7**: 43263-.
2490. Iemitsu M, Fujie S, Murakami H, Sanada K, Kawano H, Gando Y, et al. Higher cardiorespiratory fitness attenuates the risk of atherosclerosis associated with ADRB3 Trp64Arg polymorphism. European Journal Of Applied Physiology. 2014; **114**(7): 1421-8.
2491. Iglesias-Gutiérrez E, Egan B, Díaz-Martínez ÁE, Peñalvo JL, González-Medina A, Martínez-Camblor P, et al. Transient increase in homocysteine but not hyperhomocysteinemia during acute exercise at different intensities in sedentary individuals. PLoS One. 2012; **7**(12): e51185-e.
2492. Ikeda K, Klinkosz B, Greene T, Cedarbaum JM, Wong V, Lindsay RM, et al. Effects of brain-derived neurotrophic factor on motor dysfunction in wobbler mouse motor neuron disease. Annals Of Neurology. 1995; **37**(4): 505-11.
2493. Inci F, Filippini C, Baday M, Ozen MO, Calamak S, Durmus NG, et al. Multitarget, quantitative nanoplasmonic electrical field-enhanced resonating device (NE2RD) for diagnostics. Proceedings Of The National Academy Of Sciences Of The United States Of America. 2015; **112**(32): E4354-E63.

2494. Inoue K, Murofushi T, Nagaoka K, Ando N, Hakamata Y, Suzuki A, et al. Influence of Genetic Polymorphisms and Concomitant Anxiolytic Doses on Antidepressant Maintenance Doses in Japanese Patients with Depression. Biological & Pharmaceutical Bulletin. 2016; **39**(9): 1508-13.
2495. Ishii T, Wakabayashi R, Kurosaki H, Gemma A, Kida K. Association of serotonin transporter gene variation with smoking, chronic obstructive pulmonary disease, and its depressive symptoms. Journal Of Human Genetics. 2011; **56**(1): 41-6.
2496. Ishikawa C, Shiga T. The postnatal 5-HT1A receptor regulates adult anxiety and depression differently via multiple molecules. Progress In Neuro-Psychopharmacology & Biological Psychiatry. 2017; **78**: 66-74.
2497. Ising M, Depping A-M, Siebertz A, Lucae S, Unschuld PG, Kloiber S, et al. Polymorphisms in the FKBP5 gene region modulate recovery from psychosocial stress in healthy controls. The European Journal Of Neuroscience. 2008; **28**(2): 389-98.
2498. Ising M, Lucae S, Binder EB, Bettecken T, Uhr M, Ripke S, et al. A genomewide association study points to multiple loci that predict antidepressant drug treatment outcome in depression. Archives Of General Psychiatry. 2009; **66**(9): 966-75.
2499. Iyalomhe O, Chen Y, Allard J, Ntekim O, Johnson S, Bond V, et al. A standardized randomized 6-month aerobic exercise-training down-regulated pro-inflammatory genes, but up-regulated anti-inflammatory, neuron survival and axon growth-related genes. Experimental Gerontology. 2015; **69**: 159-69.
2500. Izzicupo P, Di Valerio V, D' Amico MA, Di Mauro M, Pennelli A, Falone S, et al. NAD(P)H oxidase and pro-inflammatory response during maximal exercise: role of C242T polymorphism of the P22PHOX subunit. International Journal Of Immunopathology And Pharmacology. 2010; **23**(1): 203-11.
2501. Jääskeläinen T, Paananen J, Lindström J, Eriksson JG, Tuomilehto J, Uusitupa M. Genetic predisposition to obesity and lifestyle factors--the combined analyses of twenty-six known BMI- and fourteen known waist:hip ratio (WHR)-associated variants in the Finnish Diabetes Prevention Study. The British Journal Of Nutrition. 2013; **110**(10): 1856-65.
2502. Jacobs S, Kröger J, Floegel A, Boeing H, Drogan D, Pischon T, et al. Evaluation of various biomarkers as potential mediators of the association between coffee consumption and incident type 2 diabetes in the EPIC-Potsdam Study. The American Journal Of Clinical Nutrition. 2014; **100**(3): 891-900.
2503. Jacobsen S, Garred P, Madsen HO, Heegaard NHH, Hetland ML, Stengaard-Pedersen K, et al. Mannose-binding lectin gene polymorphisms are associated with disease activity and physical disability in untreated, anti-cyclic citrullinated peptide-positive patients with early rheumatoid arthritis. The Journal Of Rheumatology. 2009; **36**(4): 731-5.
2504. Jacoby AS, Munkholm K, Vinberg M, Pedersen BK, Kessing LV. Cytokines, brain-derived neurotrophic factor and C-reactive protein in bipolar I disorder - Results from a prospective study. Journal Of Affective Disorders. 2016; **197**: 167-74.
2505. Jacquier M, Arango D, Villareal E, Torres O, Serrano ML, Cruts M, et al. APOE epsilon4 and Alzheimer's disease: positive association in a Colombian clinical series and review of the Latin-American studies. Arquivos De Neuro-Psiquiatria. 2001; **59**(1): 11-7.

2506. Jamali Z, Asadikaram G, Mahmoodi M, Sayadi A, Jamalizadeh A, Saleh-Moghadam M, et al. Vitamin D status in female students and its relation to calcium metabolism markers, lifestyles, and polymorphism in vitamin D receptor. Clinical Laboratory. 2013; **59**(3-4): 407-13.
2507. Jamnik J, García-Bailo B, Borchers CH, El-Sohemy A. Gluten Intake Is Positively Associated with Plasma α2-Macroglobulin in Young Adults. The Journal Of Nutrition. 2015; **145**(6): 1256-62.
2508. Janelidze S, Suchankova P, Ekman A, Erhardt S, Sellgren C, Samuelsson M, et al. Low IL-8 is associated with anxiety in suicidal patients: genetic variation and decreased protein levels. Acta Psychiatrica Scandinavica. 2015; **131**(4): 269-78.
2509. Järvinen TL, Järvinen TA, Sievänen H, Heinonen A, Tanner M, Huang XH, et al. Vitamin D receptor alleles and bone's response to physical activity. Calcified Tissue International. 1998; **62**(5): 413-7.
2510. Javed A, Leuchte N, Salinas G, Opitz L, Stahl-Hennig C, Sopper S, et al. Pre-infection transcript levels of FAM26F in peripheral blood mononuclear cells inform about overall plasma viral load in acute and post-acute phase after simian immunodeficiency virus infection. The Journal Of General Virology. 2016; **97**(12): 3400-12.
2511. Jenkins NT, McKenzie JA, Damcott CM, Witkowski S, Hagberg JM. Endurance exercise training effects on body fatness, VO2max, HDL-C subfractions, and glucose tolerance are influenced by a PLIN haplotype in older Caucasians. Journal Of Applied Physiology (Bethesda, Md: 1985). 2010; **108**(3): 498-506.
2512. Jeong Y, Daghlas SA, Xie Y, Hulbert MA, Pfeiffer FM, Dallas MR, et al. Skeletal Response to Soluble Activin Receptor Type IIB in Mouse Models of Osteogenesis Imperfecta. Journal Of Bone And Mineral Research: The Official Journal Of The American Society For Bone And Mineral Research. 2018.
2513. Ji LL, Peng JB, Fu CH, Tong L, Wang ZY. Sigma-1 receptor activation ameliorates anxiety-like behavior through NR2A-CREB-BDNF signaling pathway in a rat model submitted to single-prolonged stress. Mol Med Rep. 2017; **16**(4): 4987-93.
2514. Jiang YM, Li XJ, Meng ZZ, Liu YY, Zhao HB, Li N, et al. Effects of Xiaoyaosan on Stress-Induced Anxiety-Like Behavior in Rats: Involvement of CRF1 Receptor. Evid Based Complement Alternat Med. 2016; **2016**: 1238426.
2515. Jimenez PA, Rampy MA. Keratinocyte growth factor-2 accelerates wound healing in incisional wounds. The Journal Of Surgical Research. 1999; **81**(2): 238-42.
2516. Jin U-H, Park H, Li X, Davidson LA, Allred C, Patil B, et al. Structure-Dependent Modulation of Aryl Hydrocarbon Receptor-Mediated Activities by Flavonoids. Toxicological Sciences: An Official Journal Of The Society Of Toxicology. 2018; **164**(1): 205-17.
2517. Jindal A, Mahesh R, Bhatt S, Pandey D. Molecular modifications by regulating cAMP signaling and oxidant-antioxidant defence mechanisms, produce antidepressant-like effect: A possible mechanism of etazolate aftermaths of impact accelerated traumatic brain injury in rat model. Neurochem Int. 2017; **111**: 3-11.
2518. Jindal GA, Goyal Y, Yamaya K, Futran AS, Kountouridis I, Balgobin CA, et al. In vivo severity ranking of Ras pathway mutations associated with developmental disorders. Proceedings Of The National Academy Of Sciences Of The United States Of America. 2017; **114**(3): 510-5.

2519. Jing FC, Zhang J, Feng C, Nian YY, Wang JH, Hu H, et al. Potential rat model of anxiety-like gastric hypersensitivity induced by sequential stress. World J Gastroenterol. 2017; **23**(42): 7594-608.
2520. Joosen AMCP, Gielen M, Vlietinck R, Westerterp KR. Genetic analysis of physical activity in twins. The American Journal Of Clinical Nutrition. 2005; **82**(6): 1253-9.
2521. Jukić MM, Opel N, Ström J, Carrillo-Roa T, Miksys S, Novalen M, et al. Elevated CYP2C19 expression is associated with depressive symptoms and hippocampal homeostasis impairment. Molecular Psychiatry. 2017; **22**(8): 1155-63.
2522. Kabagambe EK, Beasley TM, Limdi NA. Vitamin K intake, body mass index and warfarin maintenance dose. Cardiology. 2013; **126**(4): 214-8.
2523. Kabir ZD, Che A, Fischer DK, Rice RC, Rizzo BK, Byrne M, et al. Rescue of impaired sociability and anxiety-like behavior in adult cacna1c-deficient mice by pharmacologically targeting eIF2α. Molecular Psychiatry. 2017; **22**(8): 1096-109.
2524. Kadioglu E, Kocabas NA, Demircigil GC, Coskun E, Ozcagli E, Durmaz E, et al. Assessment of individual susceptibility to baseline DNA and cytogenetic damage in a healthy Turkish population: evaluation with lifestyle factors. Genetic Testing And Molecular Biomarkers. 2012; **16**(10): 1157-64.
2525. Kah J, Volz T, Lütgehetmann M, Groth A, Lohse AW, Tiegs G, et al. Haem oxygenase-1 polymorphisms can affect HCV replication and treatment responses with different efficacy in humanized mice. Liver International: Official Journal Of The International Association For The Study Of The Liver. 2017; **37**(8): 1128-37.
2526. Kahara T, Takamura T, Hayakawa T, Nagai Y, Yamaguchi H, Katsuki T, et al. PPARgamma gene polymorphism is associated with exercise-mediated changes of insulin resistance in healthy men. Metabolism: Clinical And Experimental. 2003; **52**(2): 209-12.
2527. Kalaska B, Pawlak K, Domaniewski T, Oksztulska-Kolanek E, Znorko B, Roszczenko A, et al. Elevated Levels of Peripheral Kynurenine Decrease Bone Strength in Rats with Chronic Kidney Disease. Front Physiol. 2017; **8**: 836.
2528. Kalsen A, Hostrup M, Bangsbo J, Backer V. Combined inhalation of beta2 -agonists improves swim ergometer sprint performance but not high-intensity swim performance. Scandinavian Journal Of Medicine & Science In Sports. 2014; **24**(5): 814-22.
2529. Kamal SM, Fehr J, Roesler B, Peters T, Rasenack JW. Peginterferon alone or with ribavirin enhances HCV-specific CD4 T-helper 1 responses in patients with chronic hepatitis C. Gastroenterology. 2002; **123**(4): 1070-83.
2530. Kang BY, Kang CY, Oh SD, Bae JS, Kim KT, Lee KO. The protein polymorphism of haptoglobin in Korean elite athletes. Medical Principles And Practice: International Journal Of The Kuwait University, Health Science Centre. 2003; **12**(3): 151-5.
2531. Kang JI, Chung HC, Jeung H-C, Kim SJ, An SK, Namkoong K. FKBP5 polymorphisms as vulnerability to anxiety and depression in patients with advanced gastric cancer: a controlled and prospective study. Psychoneuroendocrinology. 2012; **37**(9): 1569-76.
2532. Kantartzis K, Machicao F, Machann J, Schick F, Fritsche A, Häring H-U, et al. The DGAT2 gene is a candidate for the dissociation between fatty liver and insulin

resistance in humans. Clinical Science (London, England: 1979). 2009; **116**(6): 531-7.
2533. Kaphingst KA, Lobb R, Fay ME, Hunt MK, Suarez EG, Fletcher RH, et al. Impact of intervention dose on cancer-related health behaviors among working-class, multiethnic, community health center patients. American Journal Of Health Promotion: AJHP. 2007; **21**(4): 262-6.
2534. Kaplan TA, Moccia-Loos G, Rabin M, McKey RM, Jr. Lack of effect of delta F508 mutation on aerobic capacity in patients with cystic fibrosis. Clinical Journal Of Sport Medicine: Official Journal Of The Canadian Academy Of Sport Medicine. 1996; **6**(4): 226-31.
2535. Kasai S, Yoshihara T, Lopatina O, Ishihara K, Higashida H. Selegiline Ameliorates Depression-Like Behavior in Mice Lacking the CD157/BST1 Gene, a Risk Factor for Parkinson's Disease. Front Behav Neurosci. 2017; **11**: 75.
2536. Kashuba AD, Nafziger AN, Kearns GL, Leeder JS, Gotschall R, Rocci ML, Jr., et al. Effect of fluvoxamine therapy on the activities of CYP1A2, CYP2D6, and CYP3A as determined by phenotyping. Clinical Pharmacology And Therapeutics. 1998; **64**(3): 257-68.
2537. Kästner N, Richter SH, Lesch K-P, Schreiber RS, Kaiser S, Sachser N. Benefits of a "vulnerability gene"? A study in serotonin transporter knockout mice. Behavioural Brain Research. 2015; **283**: 116-20.
2538. Kato M, Zanardi R, Rossini D, De Ronchi D, Okugawa G, Kinoshita T, et al. 5-HT2A gene variants influence specific and different aspects of antidepressant response in Japanese and Italian mood disorder patients. Psychiatry Research. 2009; **167**(1-2): 97-105.
2539. Katzmarzyk PT, Rankinen T, Pérusse L, Dériaz O, Tremblay A, Borecki I, et al. Linkage and association of the sodium potassium-adenosine triphosphatase alpha2 and beta1 genes with respiratory quotient and resting metabolic rate in the Québec Family Study. The Journal Of Clinical Endocrinology And Metabolism. 1999; **84**(6): 2093-7.
2540. Kaunisto MA, Jokela R, Tallgren M, Kambur O, Tikkanen E, Tasmuth T, et al. Pain in 1,000 women treated for breast cancer: a prospective study of pain sensitivity and postoperative pain. Anesthesiology. 2013; **119**(6): 1410-21.
2541. Kawamoto T, Kokaze A, Ishikawa M, Matsunaga N, Karita K, Yoshida M, et al. Joint effect of longevity-associated mitochondrial DNA 5178 C/A polymorphism and alcohol consumption on risk of hyper-LDL cholesterolemia in middle-aged Japanese men. Lipids In Health And Disease. 2011; **10**: 105-.
2542. Keller AS, Diederich L, Panknin C, DeLalio LJ, Drake JC, Sherman R, et al. Possible roles for ATP release from RBCs exclude the cAMP-mediated Panx1 pathway. American Journal Of Physiology Cell Physiology. 2017; **313**(6): C593-C603.
2543. Kelley EF, Johnson BD, Snyder EM. Beta-2 Adrenergic Receptor Genotype Influences Power Output in Healthy Subjects. Journal Of Strength And Conditioning Research. 2017; **31**(8): 2053-9.
2544. Kenney MC, Chwa M, Atilano SR, Falatoonzadeh P, Ramirez C, Malik D, et al. Molecular and bioenergetic differences between cells with African versus European inherited mitochondrial DNA haplogroups: implications for population susceptibility to diseases. Biochimica Et Biophysica Acta. 2014; **1842**(2): 208-19.
2545. Keskitalo K, Knaapila A, Kallela M, Palotie A, Wessman M, Sammalisto S, et al.

Sweet taste preferences are partly genetically determined: identification of a trait locus on chromosome 16. The American Journal Of Clinical Nutrition. 2007; **86**(1): 55-63.
2546. Khalilitehrani A, Qorbani M, Hosseini S, Pishva H. The association of MC4R rs17782313 polymorphism with dietary intake in Iranian adults. Gene. 2015; **563**(2): 125-9.
2547. Khan RJ, Gebreab SY, Sims M, Riestra P, Xu R, Davis SK. Prevalence, associated factors and heritabilities of metabolic syndrome and its individual components in African Americans: the Jackson Heart Study. BMJ Open. 2015; **5**(10): e008675-e.
2548. Kim S, Cho YS, Bhak J, O'Brian SJ, Yeo J-H. Perspectives provided by leopard and other cat genomes: how diet determined the evolutionary history of carnivores, omnivores, and herbivores. BMB Reports. 2017; **50**(1): 3-4.
2549. Kim T-H, Jung JW, Ha BG, Hong JM, Park EK, Kim H-J, et al. The effects of luteolin on osteoclast differentiation, function in vitro and ovariectomy-induced bone loss. The Journal Of Nutritional Biochemistry. 2011; **22**(1): 8-15.
2550. Kimhy D, Vakhrusheva J, Bartels MN, Armstrong HF, Ballon JS, Khan S, et al. The Impact of Aerobic Exercise on Brain-Derived Neurotrophic Factor and Neurocognition in Individuals With Schizophrenia: A Single-Blind, Randomized Clinical Trial. Schizophrenia Bulletin. 2015; **41**(4): 859-68.
2551. Kitano S, Hisatomi H, Hibi N, Kawano K, Harada S. Improved method of plasma 8-Isoprostane measurement and association analyses with habitual drinking and smoking. World Journal Of Gastroenterology. 2006; **12**(36): 5846-52.
2552. Kliethermes CL, Finn DA, Crabbe JC. Validation of a modified mirrored chamber sensitive to anxiolytics and anxiogenics in mice. Psychopharmacology. 2003; **169**(2): 190-7.
2553. Klimentidis YC, Arora A, Chougule A, Zhou J, Raichlen DA. FTO association and interaction with time spent sitting. International Journal Of Obesity (2005). 2016; **40**(3): 411-6.
2554. Klimentidis YC, Bea JW, Lohman T, Hsieh PS, Going S, Chen Z. High genetic risk individuals benefit less from resistance exercise intervention. International Journal Of Obesity (2005). 2015; **39**(9): 1371-5.
2555. Knecht AL, Truong L, Marvel SW, Reif DM, Garcia A, Lu C, et al. Transgenerational inheritance of neurobehavioral and physiological deficits from developmental exposure to benzo[a]pyrene in zebrafish. Toxicology And Applied Pharmacology. 2017; **329**: 148-57.
2556. Kocabas NA, Antonijevic I, Faghel C, Forray C, Kasper S, Lecrubier Y, et al. Dysbindin gene (DTNBP1) in major depressive disorder (MDD) patients: lack of association with clinical phenotypes. The World Journal Of Biological Psychiatry: The Official Journal Of The World Federation Of Societies Of Biological Psychiatry. 2010; **11**(8): 985-90.
2557. Kohen R, Tracy JH, Haugen E, Cain KC, Jarrett ME, Heitkemper MM. Rare Variants of the Serotonin Transporter Are Associated With Psychiatric Comorbidity in Irritable Bowel Syndrome. Biological Research For Nursing. 2016; **18**(4): 394-400.
2558. Kokaze A, Ishikawa M, Matsunaga N, Karita K, Yoshida M, Ohtsu T, et al. Unexpected combined effects of NADH dehydrogenase subunit-2 237 Leu/Met

polymorphism and green tea consumption on renal function in male Japanese health check-up examinees: a cross-sectional study. Journal Of Negative Results In Biomedicine. 2013; **12**: 17-.
2559. Kokaze A, Ishikawa M, Matsunaga N, Karita K, Yoshida M, Ohtsu T, et al. Longevity-associated mitochondrial DNA 5178 C/A polymorphism modulates the effects of coffee consumption on erythrocytic parameters in Japanese men: an exploratory cross-sectional analysis. Journal Of Physiological Anthropology. 2014; **33**: 37-.
2560. Kokaze A, Ishikawa M, Matsunaga N, Karita K, Yoshida M, Ohtsu T, et al. NADH dehydrogenase subunit-2 237 Leu/Met polymorphism modulates the effects of coffee consumption on the risk of hypertension in middle-aged Japanese men. Journal Of Epidemiology. 2009; **19**(5): 231-6.
2561. Kokaze A, Ishikawa M, Matsunaga N, Karita K, Yoshida M, Shimada N, et al. Combined effect of longevity-associated mitochondrial DNA 5178 C/A polymorphism and coffee consumption on the risk of hyper-LDL cholesterolemia in middle-aged Japanese men. Journal Of Human Genetics. 2010; **55**(9): 577-81.
2562. Kokaze A, Yoshida M, Ishikawa M, Matsunaga N, Karita K, Ochiai H, et al. Mitochondrial DNA 5178 C/A polymorphism modulates the effects of coffee consumption on elevated levels of serum liver enzymes in male Japanese health check-up examinees: an exploratory cross-sectional study. Journal Of Physiological Anthropology. 2016; **35**(1): 15-.
2563. Kokaze A, Yoshida M, Ishikawa M, Matsunaga N, Makita R, Satoh M, et al. Longevity-associated mitochondrial DNA 5178 A/C polymorphism is associated with intraocular pressure in Japanese men. Clinical & Experimental Ophthalmology. 2004; **32**(2): 131-6.
2564. Kolár J, Senková J. Reduction of mineral nutrient availability accelerates flowering of Arabidopsis thaliana. Journal Of Plant Physiology. 2008; **165**(15): 1601-9.
2565. Kondo T, Mihara K, Suzuki A, Yasui-Furukori N, Kaneko S. Combination of dopamine D2 receptor gene polymorphisms as a possible predictor of treatment-resistance to dopamine antagonists in schizophrenic patients. Progress In Neuro-Psychopharmacology & Biological Psychiatry. 2003; **27**(6): 921-6.
2566. Koo T, Lu-Nguyen NB, Malerba A, Kim E, Kim D, Cappellari O, et al. Functional Rescue of Dystrophin Deficiency in Mice Caused by Frameshift Mutations Using Campylobacter jejuni Cas9. Molecular Therapy: The Journal Of The American Society Of Gene Therapy. 2018; **26**(6): 1529-38.
2567. Korade Z, Folkes OM, Harrison FE. Behavioral and serotonergic response changes in the Dhcr7-HET mouse model of Smith-Lemli-Opitz syndrome. Pharmacology, Biochemistry, And Behavior. 2013; **106**: 101-8.
2568. Kostić M, Munjiza A, Pesic D, Peljto A, Novakovic I, Dobricic V, et al. A pilot study on predictors of brainstem raphe abnormality in patients with major depressive disorder. Journal Of Affective Disorders. 2017; **209**: 66-70.
2569. Koyama T, Matsui D, Kuriyama N, Ozaki E, Tanaka K, Oze I, et al. Genetic variants of SLC17A1 are associated with cholesterol homeostasis and hyperhomocysteinaemia in Japanese men. Scientific Reports. 2015; **5**: 15888-.
2570. Kramer DB, Mitchell SL, Monteiro J, Jones PW, Normand S-L, Hayes DL, et al. Patient Activity and Survival Following Implantable Cardioverter-Defibrillator

Implantation: The ALTITUDE Activity Study. Journal Of The American Heart Association. 2015; **4**(5).
2571. Kroeze Y, Peeters D, Boulle F, Pawluski JL, van den Hove DLA, van Bokhoven H, et al. Long-term consequences of chronic fluoxetine exposure on the expression of myelination-related genes in the rat hippocampus. Translational Psychiatry. 2015; **5**: e642-e.
2572. Kronenberg S, Apter A, Brent D, Schirman S, Melhem N, Pick N, et al. Serotonin transporter polymorphism (5-HTTLPR) and citalopram effectiveness and side effects in children with depression and/or anxiety disorders. Journal Of Child And Adolescent Psychopharmacology. 2007; **17**(6): 741-50.
2573. Kulesskaya N, Karpova NN, Ma L, Tian L, Voikar V. Mixed housing with DBA/2 mice induces stress in C57BL/6 mice: implications for interventions based on social enrichment. Front Behav Neurosci. 2014; **8**: 257.
2574. Kullo IJ, Jouni H, Austin EE, Brown S-A, Kruisselbrink TM, Isseh IN, et al. Incorporating a Genetic Risk Score Into Coronary Heart Disease Risk Estimates: Effect on Low-Density Lipoprotein Cholesterol Levels (the MI-GENES Clinical Trial). Circulation. 2016; **133**(12): 1181-8.
2575. Kullo IJ, Jouni H, Olson JE, Montori VM, Bailey KR. Design of a randomized controlled trial of disclosing genomic risk of coronary heart disease: the Myocardial Infarction Genes (MI-GENES) study. BMC Medical Genomics. 2015; **8**: 51-.
2576. Kumar P, Kumar A, Sagar R, Misra S, Faruq M, Suroliya V, et al. Association between interleukin-6 (G174C and C572G) promoter gene polymorphisms and risk of ischemic stroke in North Indian population: a case-control study. Neurological Research. 2016; **38**(1): 69-74.
2577. Kumar P, Misra S, Kumar A, Faruq M, Shakya S, Vardhan G, et al. Transforming growth factor-β1 (C509T, G800A, and T869C) gene polymorphisms and risk of ischemic stroke in North Indian population: A hospital-based case-control study. Annals Of Indian Academy Of Neurology. 2017; **20**(1): 5-12.
2578. Kumar R, Bisht NC. Duplicated RGS (Regulator of G-protein signaling) proteins exhibit conserved biochemical but differential transcriptional regulation of heterotrimeric G-protein signaling in Brassica species. Scientific Reports. 2018; **8**(1): 2176-.
2579. Kurpius MP, Alexander B. Rates of in vivo methylation of desipramine and nortriptyline. Pharmacotherapy. 2006; **26**(4): 505-10.
2580. Kwan ML, Ambrosone CB, Lee MM, Barlow J, Krathwohl SE, Ergas IJ, et al. The Pathways Study: a prospective study of breast cancer survivorship within Kaiser Permanente Northern California. Cancer Causes & Control: CCC. 2008; **19**(10): 1065-76.
2581. Kwok MK, Leung GM, Schooling CM. Habitual coffee consumption and risk of type 2 diabetes, ischemic heart disease, depression and Alzheimer's disease: a Mendelian randomization study. Scientific Reports. 2016; **6**: 36500-.
2582. Labayen I, Ruiz JR, Huybrechts I, Ortega FB, Arenaza L, González-Gross M, et al. Dietary fat intake modifies the influence of the FTO rs9939609 polymorphism on adiposity in adolescents: The HELENA cross-sectional study. Nutrition, Metabolism, And Cardiovascular Diseases: NMCD. 2016; **26**(10): 937-43.
2583. Labayen I, Ruiz JR, Ortega FB, Dallongeville J, Jiménez-Pavón D, Castillo MJ, et

al. Association between the FTO rs9939609 polymorphism and leptin in European adolescents: a possible link with energy balance control. The HELENA study. International Journal Of Obesity (2005). 2011; **35**(1): 66-71.
2584. Lacha J, Hribova P, Kotsch K, Brabcova I, Bartosova K, Volk HD, et al. Effect of cytokines and chemokines (TGF-beta, TNF-alpha, IL-6, IL-10, MCP-1, RANTES) gene polymorphisms in kidney recipients on posttransplantation outcome: influence of donor-recipient match. Transplantation Proceedings. 2005; **37**(2): 764-6.
2585. Lagou V, Liu G, Zhu H, Stallmann-Jorgensen IS, Gutin B, Dong Y, et al. Lifestyle and socioeconomic-status modify the effects of ADRB2 and NOS3 on adiposity in European-American and African-American adolescents. Obesity (Silver Spring, Md). 2011; **19**(3): 595-603.
2586. Laje G, Allen AS, Akula N, Manji H, John Rush A, McMahon FJ. Genome-wide association study of suicidal ideation emerging during citalopram treatment of depressed outpatients. Pharmacogenetics And Genomics. 2009; **19**(9): 666-74.
2587. Laje G, Cannon DM, Allen AS, Klaver JM, Peck SA, Liu X, et al. Genetic variation in HTR2A influences serotonin transporter binding potential as measured using PET and [11C]DASB. The International Journal Of Neuropsychopharmacology. 2010; **13**(6): 715-24.
2588. Laje G, Paddock S, Manji H, Rush AJ, Wilson AF, Charney D, et al. Genetic markers of suicidal ideation emerging during citalopram treatment of major depression. The American Journal Of Psychiatry. 2007; **164**(10): 1530-8.
2589. Laje G, Perlis RH, Rush AJ, McMahon FJ. Pharmacogenetics studies in STAR*D: strengths, limitations, and results. Psychiatric Services (Washington, DC). 2009; **60**(11): 1446-57.
2590. Lakshmi A, Muralidhar S, Kalyan Kumar C, Pavan Kumar A, Kalyana Chakravarthy P, Anjaneyulu V, et al. Cyclooxygenase-2-765G>C functional promoter polymorphism and its association with oral squamous cell carcinoma. Journal Of Investigative And Clinical Dentistry. 2012; **3**(3): 182-8.
2591. Lammers CH, Deuschle M, Weigmann H, Härtter S, Hiemke C, Heese C, et al. Coadministration of clozapine and fluvoxamine in psychotic patients--clinical experience. Pharmacopsychiatry. 1999; **32**(2): 76-7.
2592. Landgraf R, Wigger A. High vs low anxiety-related behavior rats: an animal model of extremes in trait anxiety. Behavior Genetics. 2002; **32**(5): 301-14.
2593. Langdon KD, Corbett D. Improved working memory following novel combinations of physical and cognitive activity. Neurorehabilitation And Neural Repair. 2012; **26**(5): 523-32.
2594. Langen B, Rudqvist N, Parris TZ, Helou K, Forssell-Aronsson E. Circadian rhythm influences genome-wide transcriptional responses to (131)I in a tissue-specific manner in mice. EJNMMI Res. 2015; **5**(1): 75.
2595. Lau C-I, Barbarulo A, Solanki A, Saldaña JI, Crompton T. The kinesin motor protein Kif7 is required for T-cell development and normal MHC expression on thymic epithelial cells (TEC) in the thymus. Oncotarget. 2017; **8**(15): 24163-76.
2596. Laugsand EA, Skorpen F, Kaasa S, Sabatowski R, Strasser F, Fayers P, et al. Genetic and Non-genetic Factors Associated With Constipation in Cancer Patients Receiving Opioids. Clinical And Translational Gastroenterology. 2015; **6**: e90-e.

2597. Lebret B. Effects of feeding and rearing systems on growth, carcass composition and meat quality in pigs. Animal: An International Journal Of Animal Bioscience. 2008; **2**(10): 1548-58.
2598. Lee B, Sur B, Yeom M, Shim I, Lee H, Hahm DH. Alpha-Asarone, a Major Component of Acorus gramineus, Attenuates Corticosterone-Induced Anxiety-Like Behaviours via Modulating TrkB Signaling Process. Korean J Physiol Pharmacol. 2014; **18**(3): 191-200.
2599. Lee H-J, Kim IK, Kang JH, Ahn Y, Han B-G, Lee J-Y, et al. Effects of common FTO gene variants associated with BMI on dietary intake and physical activity in Koreans. Clinica Chimica Acta; International Journal Of Clinical Chemistry. 2010; **411**(21-22): 1716-22.
2600. Lee J-H, Hong S-M, Shin Y-A. Effects of exercise training on stroke risk factors, homocysteine concentration, and cognitive function according the APOE genotype in stroke patients. Journal Of Exercise Rehabilitation. 2018; **14**(2): 267-74.
2601. Lee JKW, Koh ACH, Koh SXT, Liu GJX, Nio AQX, Fan PWP. Neck cooling and cognitive performance following exercise-induced hyperthermia. European Journal Of Applied Physiology. 2014; **114**(2): 375-84.
2602. Lee JS, Kawakubo K, Inoue S, Akabayashi A. Effect of β(3)-adrenergic receptor gene polymorphism on body weight change in middle-aged, overweight women. Environmental Health And Preventive Medicine. 2006; **11**(2): 69-74.
2603. Lee M, Kwon DY, Kim M-S, Choi CR, Park M-Y, Kim A-J. Genome-wide association study for the interaction between BMR and BMI in obese Korean women including overweight. Nutrition Research And Practice. 2016; **10**(1): 115-24.
2604. Lee P-C, Yang Y-Y, Lin M-W, Hou M-C, Huang C-S, Lee K-C, et al. Benzodiazepine-associated hepatic encephalopathy significantly increased healthcare utilization and medical costs of Chinese cirrhotic patients: 7-year experience. Digestive Diseases And Sciences. 2014; **59**(7): 1603-16.
2605. Lee SY, Masaoka T, Han HS, Matsuzaki J, Hong MJ, Fukuhara S, et al. A prospective study on symptom generation according to spicy food intake and TRPV1 genotypes in functional dyspepsia patients. Neurogastroenterology And Motility: The Official Journal Of The European Gastrointestinal Motility Society. 2016; **28**(9): 1401-8.
2606. Lee T-W, Yu YWY, Hong C-J, Tsai S-J, Wu H-C, Chen T-J. The effects of catechol-O-methyl-transferase polymorphism Val158Met on functional connectivity in healthy young females: a resting EEG study. Brain Research. 2011; **1377**: 21-31.
2607. Leggio GM, Torrisi SA, Castorina A, Platania CBM, Impellizzeri AAR, Fidilio A, et al. Dopamine D3 receptor-dependent changes in alpha6 GABAA subunit expression in striatum modulate anxiety-like behaviour: Responsiveness and tolerance to diazepam. European Neuropsychopharmacology: The Journal Of The European College Of Neuropsychopharmacology. 2015; **25**(9): 1427-36.
2608. Le-Niculescu H, Balaraman Y, Patel SD, Ayalew M, Gupta J, Kuczenski R, et al. Convergent functional genomics of anxiety disorders: translational identification of genes, biomarkers, pathways and mechanisms. Translational Psychiatry. 2011; **1**: e9-e.
2609. Lenze EJ, Dixon D, Nowotny P, Lotrich FE, Doré PM, Pollock BG, et al. Escitalopram reduces attentional performance in anxious older adults with high-expression genetic variants at serotonin 2A and 1B receptors. The International Journal

Of Neuropsychopharmacology. 2013; **16**(2): 279-88.
2610. Lenze EJ, Mantella RC, Shi P, Goate AM, Nowotny P, Butters MA, et al. Elevated cortisol in older adults with generalized anxiety disorder is reduced by treatment: a placebo-controlled evaluation of escitalopram. The American Journal Of Geriatric Psychiatry: Official Journal Of The American Association For Geriatric Psychiatry. 2011; **19**(5): 482-90.
2611. Lenze EJ, Sheffrin M, Driscoll HC, Mulsant BH, Pollock BG, Dew MA, et al. Incomplete response in late-life depression: getting to remission. Dialogues In Clinical Neuroscience. 2008; **10**(4): 419-30.
2612. Leon AS, Togashi K, Rankinen T, Després J-P, Rao DC, Skinner JS, et al. Association of apolipoprotein E polymorphism with blood lipids and maximal oxygen uptake in the sedentary state and after exercise training in the HERITAGE family study. Metabolism: Clinical And Experimental. 2004; **53**(1): 108-16.
2613. Lesch KP, Mossner R. Knockout Corner: 5-HT(1A) receptor inactivation: anxiety or depression as a murine experience. Int J Neuropsychopharmacol. 1999; **2**(4): 327-31.
2614. Lester KJ, Coleman JRI, Roberts S, Keers R, Breen G, Bögels S, et al. Genetic variation in the endocannabinoid system and response to Cognitive Behavior Therapy for child anxiety disorders. American Journal Of Medical Genetics Part B, Neuropsychiatric Genetics: The Official Publication Of The International Society Of Psychiatric Genetics. 2017; **174**(2): 144-55.
2615. Leussis MP, Berry-Scott EM, Saito M, Jhuang H, de Haan G, Alkan O, et al. The ANK3 bipolar disorder gene regulates psychiatric-related behaviors that are modulated by lithium and stress. Biological Psychiatry. 2013; **73**(7): 683-90.
2616. Levitan RD, Masellis M, Basile VS, Lam RW, Kaplan AS, Davis C, et al. The dopamine-4 receptor gene associated with binge eating and weight gain in women with seasonal affective disorder: an evolutionary perspective. Biological Psychiatry. 2004; **56**(9): 665-9.
2617. Li C, Li M, Yu H, Shen X, Wang J, Sun X, et al. Neuropeptide VGF C-Terminal Peptide TLQP-62 Alleviates Lipopolysaccharide-Induced Memory Deficits and Anxiety-like and Depression-like Behaviors in Mice: The Role of BDNF/TrkB Signaling. ACS Chem Neurosci. 2017; **8**(9): 2005-18.
2618. Li F, Shao Q, Ji D, Li B, Chen G. Genetic association between CD44 polymorphisms and chronic hepatitis B virus infection in a Chinese Han population. International Journal Of Clinical And Experimental Pathology. 2015; **8**(9): 11675-9.
2619. Li J, Wang Y, Zhou R, Zhang H, Yang L, Wang B, et al. Serotonin 5-HT1B receptor gene and attention deficit hyperactivity disorder in Chinese Han subjects. American Journal Of Medical Genetics Part B, Neuropsychiatric Genetics: The Official Publication Of The International Society Of Psychiatric Genetics. 2005; **132B**(1): 59-63.
2620. Li J, Xie X, Li Y, Liu X, Liao X, Su YA, et al. Differential Behavioral and Neurobiological Effects of Chronic Corticosterone Treatment in Adolescent and Adult Rats. Front Mol Neurosci. 2017; **10**: 25.
2621. Li J-H, Chen W, Zhu X-J, Lin Y-J, Qiu L-Q, Cai C-X, et al. Associations of nicotinamide N-methyltransferase gene single nucleotide polymorphisms with sport performance and relative maximal oxygen uptake. Journal Of Sports Sciences. 2017;

35(22): 2185-90.
2622. Li M, Li C, Yu H, Cai X, Shen X, Sun X, et al. Lentivirus-mediated interleukin-1beta (IL-1beta) knock-down in the hippocampus alleviates lipopolysaccharide (LPS)-induced memory deficits and anxiety- and depression-like behaviors in mice. J Neuroinflammation. 2017; **14**(1): 190.
2623. Li S, Wang Q, Pan L, Li H, Yang X, Jiang F, et al. The association of dopamine pathway gene score, nicotine dependence and smoking cessation in a rural male population of Shandong, China. The American Journal On Addictions. 2016; **25**(6): 493-8.
2624. Li X-G, Ma N, Sun S-S, Xu Z, Li W, Wang Y-J, et al. Association of genetic variant and platelet function in patients undergoing neuroendovascular stenting. Postgraduate Medical Journal. 2017; **93**(1103): 555-9.
2625. Li Y, Yang P, Wu S-l, Yuan J-x, Wu Y, Zhao D-d, et al. [Effect of CYP11B2 gene -344T/C polymorphism on renin-angiotensin-aldosterone system activity and blood pressure response to hydrochlorothiazide]. Zhonghua Yi Xue Yi Chuan Xue Za Zhi = Zhonghua Yixue Yichuanxue Zazhi = Chinese Journal Of Medical Genetics. 2012; **29**(1): 68-71.
2626. Lienert F, Torella JP, Chen J-H, Norsworthy M, Richardson RR, Silver PA. Two- and three-input TALE-based AND logic computation in embryonic stem cells. Nucleic Acids Research. 2013; **41**(21): 9967-75.
2627. Lim JM, Lee YJ, Cho HR, Park DC, Jung GW, Ku SK, et al. Extracellular polysaccharides purified from Aureobasidium pullulans SM2001 (Polycan) inhibit dexamethasoneinduced muscle atrophy in mice. Int J Mol Med. 2018; **41**(3): 1245-64.
2628. Lim J-M, Lee YJ, Cho H-R, Park D-C, Jung G-W, Ku SK, et al. Extracellular polysaccharides purified from Aureobasidium pullulans SM-2001 (Polycan) inhibit dexamethasone-induced muscle atrophy in mice. International Journal Of Molecular Medicine. 2018; **41**(3): 1245-64.
2629. Lim LC, Tan MH, Eng C, Teh BT, Rajasoorya RC. Thymic carcinoid in multiple endocrine neoplasia 1: genotype-phenotype correlation and prevention. Journal Of Internal Medicine. 2006; **259**(4): 428-32.
2630. Lin E, Kuo P-H, Liu Y-L, Yang AC, Kao C-F, Tsai S-J. Association and interaction of APOA5, BUD13, CETP, LIPA and health-related behavior with metabolic syndrome in a Taiwanese population. Scientific Reports. 2016; **6**: 36830-.
2631. Lin E, Kuo P-H, Liu Y-L, Yang AC, Kao C-F, Tsai S-J. Effects of circadian clock genes and environmental factors on cognitive aging in old adults in a Taiwanese population. Oncotarget. 2017; **8**(15): 24088-98.
2632. Lin E, Kuo P-H, Liu Y-L, Yang AC, Kao C-F, Tsai S-J. Effects of circadian clock genes and health-related behavior on metabolic syndrome in a Taiwanese population: Evidence from association and interaction analysis. PLoS One. 2017; **12**(3): e0173861-e.
2633. Lin J, Wang J, Greisinger AJ, Grossman HB, Forman MR, Dinney CP, et al. Energy balance, the PI3K-AKT-mTOR pathway genes, and the risk of bladder cancer. Cancer Prevention Research (Philadelphia, Pa). 2010; **3**(4): 505-17.
2634. Lin K-M, Chiu Y-F, Tsai IJ, Chen C-H, Shen WW, Liu SC, et al. ABCB1 gene polymorphisms are associated with the severity of major depressive disorder and its

response to escitalopram treatment. Pharmacogenetics And Genomics. 2011; **21**(4): 163-70.
2635. Lin W-T, Liao Y-J, Peng Y-C, Chang C-H, Lin C-H, Yeh H-Z, et al. Relationship between use of selective serotonin reuptake inhibitors and irritable bowel syndrome: A population-based cohort study. World Journal Of Gastroenterology. 2017; **23**(19): 3513-21.
2636. Lin YF, Huang MC, Liu HC. Glycogen synthase kinase 3beta gene polymorphisms may be associated with bipolar I disorder and the therapeutic response to lithium. J Affect Disord. 2013; **147**(1-3): 401-6.
2637. Lin Y-F, Huang M-C, Liu H-C. Glycogen synthase kinase 3β gene polymorphisms may be associated with bipolar I disorder and the therapeutic response to lithium. Journal Of Affective Disorders. 2013; **147**(1-3): 401-6.
2638. Lind PA, Eriksson CJP, Wilhelmsen KC. The role of aldehyde dehydrogenase-1 (ALDH1A1) polymorphisms in harmful alcohol consumption in a Finnish population. Human Genomics. 2008; **3**(1): 24-35.
2639. Lindholm JSO, Autio H, Vesa L, Antila H, Lindemann L, Hoener MC, et al. The antidepressant-like effects of glutamatergic drugs ketamine and AMPA receptor potentiator LY 451646 are preserved in bdnf^{+}/$^{-}$ heterozygous null mice. Neuropharmacology. 2012; **62**(1): 391-7.
2640. Lisoway AJ, Zai G, Tiwari AK, Zai CC, Wigg K, Goncalves V, et al. Pharmacogenetic evaluation of a DISP1 gene variant in antidepressant treatment of obsessive-compulsive disorder. Human Psychopharmacology. 2018: e2659-e.
2641. Lit L, Belanger JM, Boehm D, Lybarger N, Haverbeke A, Diederich C, et al. Characterization of a dopamine transporter polymorphism and behavior in Belgian Malinois. BMC Genetics. 2013; **14**: 45-.
2642. Liu G, Gu B, He X-P, Joshi RB, Wackerle HD, Rodriguiz RM, et al. Transient inhibition of TrkB kinase after status epilepticus prevents development of temporal lobe epilepsy. Neuron. 2013; **79**(1): 31-8.
2643. Liu J, Hu D, Jiang Y, Xi H, Li W. Association between single nucleotide polymorphisms in the OPRM1 gene and intraoperative remifentanil consumption in northern Chinese women. Pharmacology. 2014; **94**(5-6): 273-9.
2644. Liu J-C, Hsu Y-P, Kao P-F, Hao W-R, Liu S-H, Lin C-F, et al. Influenza Vaccination Reduces Dementia Risk in Chronic Kidney Disease Patients: A Population-Based Cohort Study. Medicine. 2016; **95**(9): e2868-e.
2645. Liu P, Tang Z, Lan G, Zhu Q, Chen H, You Y, et al. Early antiretroviral therapy on reducing HIV transmission in China: strengths, weaknesses and next focus of the program. Scientific Reports. 2018; **8**(1): 3431-.
2646. Liu Q, Xiao L, Zhou Y, Deng K, Tan G, Han Y, et al. Development of Streptomyces sp. FR-008 as an emerging chassis. Synthetic And Systems Biotechnology. 2016; **1**(3): 207-14.
2647. Liu Z-H, Chuang D-M, Smith CB. Lithium ameliorates phenotypic deficits in a mouse model of fragile X syndrome. The International Journal Of Neuropsychopharmacology. 2011; **14**(5): 618-30.
2648. Lochner C, Hemmings SMJ, Kinnear CJ, Niehaus DJH, Nel DG, Corfield VA, et al. Cluster analysis of obsessive-compulsive spectrum disorders in patients with

obsessive-compulsive disorder: clinical and genetic correlates. Comprehensive Psychiatry. 2005; **46**(1): 14-9.
2649. Lochner C, Seedat S, Hemmings SMJ, Moolman-Smook JC, Kidd M, Stein DJ. Investigating the possible effects of trauma experiences and 5-HTT on the dissociative experiences of patients with OCD using path analysis and multiple regression. Neuropsychobiology. 2007; **56**(1): 6-13.
2650. Lochner C, Serebro P, van der Merwe L, Hemmings S, Kinnear C, Seedat S, et al. Comorbid obsessive-compulsive personality disorder in obsessive-compulsive disorder (OCD): a marker of severity. Progress In Neuro-Psychopharmacology & Biological Psychiatry. 2011; **35**(4): 1087-92.
2651. Lopez JP, Diallo A, Cruceanu C, Fiori LM, Laboissiere S, Guillet I, et al. Biomarker discovery: quantification of microRNAs and other small non-coding RNAs using next generation sequencing. BMC Medical Genomics. 2015; **8**: 35-.
2652. López-Alarcón M, Hunter GR, Gower BA, Fernández JR. IGF-I polymorphism is associated with lean mass, exercise economy, and exercise performance among premenopausal women. Archives Of Medical Research. 2007; **38**(1): 56-63.
2653. López-Cancio E, Ricciardi AC, Sobrino T, Cortés J, de la Ossa NP, Millán M, et al. Reported Prestroke Physical Activity Is Associated with Vascular Endothelial Growth Factor Expression and Good Outcomes after Stroke. Journal Of Stroke And Cerebrovascular Diseases: The Official Journal Of National Stroke Association. 2017; **26**(2): 425-30.
2654. Lotrich FE, Albusaysi S, Ferrell RE. Brain-derived neurotrophic factor serum levels and genotype: association with depression during interferon-alpha treatment. Neuropsychopharmacology. 2013; **38**(6): 985-95.
2655. Lotrich FE, Albusaysi S, Ferrell RE. Brain-derived neurotrophic factor serum levels and genotype: association with depression during interferon-α treatment. Neuropsychopharmacology: Official Publication Of The American College Of Neuropsychopharmacology. 2013; **38**(6): 985-95.
2656. Louie JCY, Flood VM, Burlutsky G, Rangan AM, Gill TP, Mitchell P. Dairy consumption and the risk of 15-year cardiovascular disease mortality in a cohort of older Australians. Nutrients. 2013; **5**(2): 441-54.
2657. Lovett ST, Mortimer RK. Characterization of null mutants of the RAD55 gene of Saccharomyces cerevisiae: effects of temperature, osmotic strength and mating type. Genetics. 1987; **116**(4): 547-53.
2658. Lucia A, Esteve-Lanao J, Oliván J, Gómez-Gallego F, San Juan AF, Santiago C, et al. Physiological characteristics of the best Eritrean runners-exceptional running economy. Applied Physiology, Nutrition, And Metabolism = Physiologie Appliquee, Nutrition Et Metabolisme. 2006; **31**(5): 530-40.
2659. Lucia A, Gómez-Gallego F, Santiago C, Pérez M, Maté-Muñoz JL, Chamorro-Viña C, et al. The 577X allele of the ACTN3 gene is associated with improved exercise capacity in women with McArdle's disease. Neuromuscular Disorders: NMD. 2007; **17**(8): 603-10.
2660. Luglio HF, Inggriyani CG, Huriyati E, Julia M, Susilowati R. Association of SNPs in GHSR rs292216 and rs509035 on dietary intake in Indonesian obese female adolescents. International Journal Of Molecular Epidemiology And Genetics. 2014; **5**(4):

195-9.
2661. Luhrs L, Manlapaz C, Kedzie K, Rao S, Cabrera-Ghayouri S, Donello J, et al. Function of brain α2B-adrenergic receptor characterized with subtype-selective α2B antagonist and KO mice. Neuroscience. 2016; **339**: 608-21.
2662. Luo GQ, Liu L, Gao QW, Wu XN, Xiang W, Deng WT. Mangiferin prevents corticosterone-induced behavioural deficits via alleviation of oxido-nitrosative stress and down-regulation of indoleamine 2,3-dioxygenase (IDO) activity. Neurol Res. 2017; **39**(8): 709-18.
2663. Lv ZJ, Wang XF, Zhai Y, Song SX. [RNA responsible for conferring a DNase I sensitive structure on albumin gene in assembled chromatin]. Yi Chuan. 2003; **25**(1): 30-6.
2664. Ma C, Wang L, Lee UY, Tanabe K, Kang S, Zhang CX. Pre-harvest foliar application of ethephon strengthens gibberellins-induced fruit expansion in Pyrus pyrifolia. Genetics And Molecular Research: GMR. 2016; **15**(4).
2665. Macdonald HM, McGuigan FE, Lanham-New SA, Fraser WD, Ralston SH, Reid DM. Vitamin K1 intake is associated with higher bone mineral density and reduced bone resorption in early postmenopausal Scottish women: no evidence of gene-nutrient interaction with apolipoprotein E polymorphisms. The American Journal Of Clinical Nutrition. 2008; **87**(5): 1513-20.
2666. Macedo IC, de Oliveira C, Vercelino R, Souza A, Laste G, Medeiros LF, et al. Repeated transcranial direct current stimulation reduces food craving in Wistar rats. Appetite. 2016; **103**: 29-37.
2667. Machado-Vieira R, Pivovarova NB, Stanika RI, Yuan P, Wang Y, Zhou R, et al. The Bcl-2 gene polymorphism rs956572AA increases inositol 1,4,5-trisphosphate receptor-mediated endoplasmic reticulum calcium release in subjects with bipolar disorder. Biological Psychiatry. 2011; **69**(4): 344-52.
2668. Machado-Vieira R, Yuan P, Brutsche N, DiazGranados N, Luckenbaugh D, Manji HK, et al. Brain-derived neurotrophic factor and initial antidepressant response to an N-methyl-D-aspartate antagonist. The Journal Of Clinical Psychiatry. 2009; **70**(12): 1662-6.
2669. Macho-Azcárate T, Calabuig J, Martí A, Martínez JA. A maximal effort trial in obese women carrying the beta2-adrenoceptor Gln27Glu polymorphism. Journal Of Physiology And Biochemistry. 2002; **58**(2): 103-8.
2670. Macho-Azcarate T, Marti A, González A, Martinez JA, Ibañez J. Gln27Glu polymorphism in the beta2 adrenergic receptor gene and lipid metabolism during exercise in obese women. International Journal Of Obesity And Related Metabolic Disorders: Journal Of The International Association For The Study Of Obesity. 2002; **26**(11): 1434-41.
2671. Madra M, Zeltser LM. BDNF-Val66Met variant and adolescent stress interact to promote susceptibility to anorexic behavior in mice. Translational Psychiatry. 2016; **6**: e776-e.
2672. Maina G, Rosso G, Zanardini R, Bogetto F, Gennarelli M, Bocchio-Chiavetto L. Serum levels of brain-derived neurotrophic factor in drug-naïve obsessive-compulsive patients: a case-control study. Journal Of Affective Disorders. 2010; **122**(1-2): 174-8.
2673. Malczewska-Lenczowska J, Orysiak J, Majorczyk E, Zdanowicz R, Szczepańska

B, Starczewski M, et al. Total Hemoglobin Mass, Aerobic Capacity, and HBB Gene in Polish Road Cyclists. Journal Of Strength And Conditioning Research. 2016; **30**(12): 3512-9.
2674. Malloul H, Bennis M, Bonzano S, Gambarotta G, Perroteau I, De Marchis S, et al. Decreased Hippocampal Neuroplasticity and Behavioral Impairment in an Animal Model of Inhalant Abuse. Front Neurosci. 2018; **12**: 35.
2675. Marais L, Stein DJ, Daniels WMU. Exercise increases BDNF levels in the striatum and decreases depressive-like behavior in chronically stressed rats. Metabolic Brain Disease. 2009; **24**(4): 587-97.
2676. Markert C, Schweizer Y, Hellwig R, Wirsching T, Riedel K-D, Burhenne J, et al. Clarithromycin substantially increases steady-state bosentan exposure in healthy volunteers. British Journal Of Clinical Pharmacology. 2014; **77**(1): 141-8.
2677. Marques-Vidal P, Bochud M, Paccaud F, Waterworth D, Bergmann S, Preisig M, et al. No interaction between alcohol consumption and HDL-related genes on HDL cholesterol levels. Atherosclerosis. 2010; **211**(2): 551-7.
2678. Marsaux CF, Celis-Morales C, Fallaize R, Macready AL, Kolossa S, Woolhead C, et al. Effects of a Web-Based Personalized Intervention on Physical Activity in European Adults: A Randomized Controlled Trial. Journal Of Medical Internet Research. 2015; **17**(10): e231-e.
2679. Marsh AA, Finger EC, Buzas B, Soliman N, Richell RA, Vythilingham M, et al. Impaired recognition of fear facial expressions in 5-HTTLPR S-polymorphism carriers following tryptophan depletion. Psychopharmacology. 2006; **189**(3): 387-94.
2680. Marsh DG, Meyers DA, Freidhoff LR, Ehrlich-Kautzky E, Roebber M, Norman PS, et al. HLA-Dw2: a genetic marker for human immune response to short ragweed pollen allergen Ra5. II. Response after ragweed immunotherapy. The Journal Of Experimental Medicine. 1982; **155**(5): 1452-63.
2681. Marshall KG, Howell S, Badaloo AV, Reid M, Farrall M, Forrester T, et al. Polymorphisms in genes involved in folate metabolism as risk factors for oedematous severe childhood malnutrition: a hypothesis-generating study. Annals Of Tropical Paediatrics. 2006; **26**(2): 107-14.
2682. Martínez C, Albet C, Agúndez JA, Herrero E, Carrillo JA, Márquez M, et al. Comparative in vitro and in vivo inhibition of cytochrome P450 CYP1A2, CYP2D6, and CYP3A by H2-receptor antagonists. Clinical Pharmacology And Therapeutics. 1999; **65**(4): 369-76.
2683. Martinez-Gomez D, Gomez-Martinez S, Puertollano MA, Nova E, Wärnberg J, Veiga OL, et al. Design and evaluation of a treatment programme for Spanish adolescents with overweight and obesity. The EVASYON Study. BMC Public Health. 2009; **9**: 414-.
2684. Martínez-González I, Cruz M-J, Moreno R, Morell F, Muñoz X, Aran JM. Human mesenchymal stem cells resolve airway inflammation, hyperreactivity, and histopathology in a mouse model of occupational asthma. Stem Cells And Development. 2014; **23**(19): 2352-63.
2685. Marzotto M, Olioso D, Brizzi M, Tononi P, Cristofoletti M, Bellavite P. Extreme sensitivity of gene expression in human SH-SY5Y neurocytes to ultra-low doses of Gelsemium sempervirens. BMC Complementary And Alternative Medicine. 2014; **14**:

104-.
2686. Mas S, Blázquez A, Rodríguez N, Boloc D, Lafuente A, Arnaiz JA, et al. Pharmacogenetic study focused on fluoxetine pharmacodynamics in children and adolescent patients: impact of the serotonin pathway. Pharmacogenetics And Genomics. 2016; **26**(11): 487-96.
2687. Massart F, Marini F, Menegato A, Del Monte F, Nuti M, Butitta F, et al. Allelic genes involved in artery compliance and susceptibility to sporadic abdominal aortic aneurysm. The Journal Of Steroid Biochemistry And Molecular Biology. 2004; **92**(5): 413-8.
2688. Masschelein E, Puype J, Broos S, Van Thienen R, Deldicque L, Lambrechts D, et al. A genetic predisposition score associates with reduced aerobic capacity in response to acute normobaric hypoxia in lowlanders. High Altitude Medicine & Biology. 2015; **16**(1): 34-42.
2689. Masuo K, Katsuya T, Kawaguchi H, Fu Y, Rakugi H, Ogihara T, et al. Rebound weight gain as associated with high plasma norepinephrine levels that are mediated through polymorphisms in the beta2-adrenoceptor. American Journal Of Hypertension. 2005; **18**(11): 1508-16.
2690. Mateus M, Ilg MM, Stebbeds WJ, Christopher N, Muneer A, Ralph DJ, et al. Understanding the Role of Adenosine Receptors in the Myofibroblast Transformation in Peyronie's Disease. The Journal Of Sexual Medicine. 2018; **15**(7): 947-57.
2691. Mathé C, Weill CO, Mattioli TA, Berthomieu C, Houée-Levin C, Tremey E, et al. Assessing the role of the active-site cysteine ligand in the superoxide reductase from Desulfoarculus baarsii. The Journal Of Biological Chemistry. 2007; **282**(30): 22207-16.
2692. Mauriège P, Després JP, Marcotte M, Tremblay A, Nadeau A, Moorjani S, et al. Adipose tissue lipolysis after long-term overfeeding in identical twins. International Journal Of Obesity And Related Metabolic Disorders: Journal Of The International Association For The Study Of Obesity. 1992; **16**(3): 219-25.
2693. Maximino C, Lima MG, Olivera KRM, Picanço-Diniz DLW, Herculano AM. Adenosine A1, but not A2, receptor blockade increases anxiety and arousal in Zebrafish. Basic & Clinical Pharmacology & Toxicology. 2011; **109**(3): 203-7.
2694. McAlpine DE, O'Kane DJ, Black JL, Mrazek DA. Cytochrome P450 2D6 genotype variation and venlafaxine dosage. Mayo Clinic Proceedings. 2007; **82**(9): 1065-8.
2695. McCaffery JM, Ordovas JM, Huggins GS, Lai CQ, Espeland MA, Tate DF, et al. Weight gain prevention buffers the impact of CETP rs3764261 on high density lipoprotein cholesterol in young adulthood: The Study of Novel Approaches to Weight Gain Prevention (SNAP). Nutrition, Metabolism, And Cardiovascular Diseases: NMCD. 2018.
2696. McCaffery JM, Papandonatos GD, Huggins GS, Peter I, Erar B, Kahn SE, et al. Human cardiovascular disease IBC chip-wide association with weight loss and weight regain in the look AHEAD trial. Human Heredity. 2013; **75**(2-4): 160-74.
2697. McCole SD, Brown MD, Moore GE, Ferrell RE, Wilund KR, Huberty A, et al. Angiotensinogen M235T polymorphism associates with exercise hemodynamics in postmenopausal women. Physiological Genomics. 2002; **10**(2): 63-9.
2698. McCole SD, Shuldiner AR, Brown MD, Moore GE, Ferrell RE, Wilund KR, et al.

Beta2- and beta3-adrenergic receptor polymorphisms and exercise hemodynamics in postmenopausal women. Journal Of Applied Physiology (Bethesda, Md: 1985). 2004; **96**(2): 526-30.
2699. McDougle CJ, Epperson CN, Price LH, Gelernter J. Evidence for linkage disequilibrium between serotonin transporter protein gene (SLC6A4) and obsessive compulsive disorder. Molecular Psychiatry. 1998; **3**(3): 270-3.
2700. McKenzie JA, Witkowski S, Ludlow AT, Roth SM, Hagberg JM. AKT1 G205T genotype influences obesity-related metabolic phenotypes and their responses to aerobic exercise training in older Caucasians. Experimental Physiology. 2011; **96**(3): 338-47.
2701. McMahon FJ, Buervenich S, Charney D, Lipsky R, Rush AJ, Wilson AF, et al. Variation in the gene encoding the serotonin 2A receptor is associated with outcome of antidepressant treatment. American Journal Of Human Genetics. 2006; **78**(5): 804-14.
2702. McMullan RC, Kelly SA, Hua K, Buckley BK, Faber JE, Pardo-Manuel de Villena F, et al. Long-term exercise in mice has sex-dependent benefits on body composition and metabolism during aging. Physiological Reports. 2016; **4**(21).
2703. Meeks KAC, Stronks K, Adeyemo A, Addo J, Bahendeka S, Beune E, et al. Peripheral insulin resistance rather than beta cell dysfunction accounts for geographical differences in impaired fasting blood glucose among sub-Saharan African individuals: findings from the RODAM study. Diabetologia. 2017; **60**(5): 854-64.
2704. Menshikova EV, Ritov VB, Ferrell RE, Azuma K, Goodpaster BH, Kelley DE. Characteristics of skeletal muscle mitochondrial biogenesis induced by moderate-intensity exercise and weight loss in obesity. Journal Of Applied Physiology (Bethesda, Md: 1985). 2007; **103**(1): 21-7.
2705. Meyer K, Korz V. Estrogen receptor α functions in the regulation of motivation and spatial cognition in young male rats. PLoS One. 2013; **8**(11): e79303-e.
2706. Miao N, Jin J, Kim S-N, Sun T. Hippocampal MicroRNAs Respond to Administration of Antidepressant Fluoxetine in Adult Mice. International Journal Of Molecular Sciences. 2018; **19**(3).
2707. Milgrom Y, Milgrom C, Constantini N, Applbaum Y, Radeva-Petrova D, Finestone AS. The effect of very high versus very low sustained loading on the lower back and knees in middle life. Biomed Research International. 2013; **2013**: 921830-.
2708. Millan MJ, Brocco M, Papp M, Serres F, La Rochelle CD, Sharp T, et al. S32504, a novel naphtoxazine agonist at dopamine D3/D2 receptors: III. Actions in models of potential antidepressive and anxiolytic activity in comparison with ropinirole. The Journal Of Pharmacology And Experimental Therapeutics. 2004; **309**(3): 936-50.
2709. Miller A, Brown LK, Pastores GM, Desnick RJ. Pulmonary involvement in type 1 Gaucher disease: functional and exercise findings in patients with and without clinical interstitial lung disease. Clinical Genetics. 2003; **63**(5): 368-76.
2710. Miller JS, Diggle PK. Diversification of andromonoecy in Solanum section Lasiocarpa (Solanaceae): the roles of phenotypic plasticity and architecture. American Journal Of Botany. 2003; **90**(5): 707-15.
2711. Miller M, Bales KL, Taylor SL, Yoon J, Hostetler CM, Carter CS, et al. Oxytocin and vasopressin in children and adolescents with autism spectrum disorders: sex differences and associations with symptoms. Autism Research: Official Journal Of The

International Society For Autism Research. 2013; **6**(2): 91-102.
2712. Minic Z, Zhang Y, Mao G, Goshgarian HG. Transporter Protein-Coupled DPCPX Nanoconjugates Induce Diaphragmatic Recovery after SCI by Blocking Adenosine A1 Receptors. The Journal Of Neuroscience: The Official Journal Of The Society For Neuroscience. 2016; **36**(12): 3441-52.
2713. Miranda-Vilela AL, Ribeiro IF, Grisolia CK. Association between interleukin 6 -174 G/C promoter gene polymorphism and runners' responses to the dietary ingestion of antioxidant supplementation based on pequi (Caryocar brasiliense Camb.) oil: a before-after study. Genetics And Molecular Biology. 2016; **39**(4): 554-66.
2714. Miscevic F, Foong J, Schmitt B, Blaser S, Brudno M, Schulze A. An MRspec database query and visualization engine with applications as a clinical diagnostic and research tool. Molecular Genetics And Metabolism. 2016; **119**(4): 300-6.
2715. Miyaki K, Sutani S, Kikuchi H, Takei I, Murata M, Watanabe K, et al. Increased risk of obesity resulting from the interaction between high energy intake and the Trp64Arg polymorphism of the beta3-adrenergic receptor gene in healthy Japanese men. Journal Of Epidemiology. 2005; **15**(6): 203-10.
2716. Miyamoto T, Kou K, Yanamoto H, Hashimoto S, Ikawa M, Sekiyama T, et al. Effect of Neuromuscular Electrical Stimulation on Brain-derived Neurotrophic Factor. International Journal Of Sports Medicine. 2018; **39**(1): 5-11.
2717. Mizoo T, Taira N, Nishiyama K, Nogami T, Iwamoto T, Motoki T, et al. Effects of lifestyle and single nucleotide polymorphisms on breast cancer risk: a case-control study in Japanese women. BMC Cancer. 2013; **13**: 565-.
2718. Młyniec K, Nowak G. Up-regulation of the GPR39 Zn2+-sensing receptor and CREB/BDNF/TrkB pathway after chronic but not acute antidepressant treatment in the frontal cortex of zinc-deficient mice. Pharmacological Reports: PR. 2015; **67**(6): 1135-40.
2719. Mocelin R, Herrmann AP, Marcon M, Rambo CL, Rohden A, Bevilaqua F, et al. N-acetylcysteine prevents stress-induced anxiety behavior in zebrafish. Pharmacology, Biochemistry, And Behavior. 2015; **139 Pt B**: 121-6.
2720. Moises HW, Zoega T, Gottesman II. The glial growth factors deficiency and synaptic destabilization hypothesis of schizophrenia. BMC Psychiatry. 2002; **2**: 8-.
2721. Molendijk ML, Bus BAA, Spinhoven P, Penninx BWJH, Kenis G, Prickaerts J, et al. Serum levels of brain-derived neurotrophic factor in major depressive disorder: state-trait issues, clinical features and pharmacological treatment. Molecular Psychiatry. 2011; **16**(11): 1088-95.
2722. Montesinos J, Pascual M, Rodríguez-Arias M, Miñarro J, Guerri C. Involvement of TLR4 in the long-term epigenetic changes, rewarding and anxiety effects induced by intermittent ethanol treatment in adolescence. Brain, Behavior, And Immunity. 2016; **53**: 159-71.
2723. Moradi S, Khorrami-Nezhad L, Ali-Akbar S, Zare F, Alipour T, Dehghani Kari Bozorg A, et al. The associations between dietary patterns and bone health, according to the TGF-β1 T869→C polymorphism, in postmenopausal Iranian women. Aging Clinical And Experimental Research. 2018; **30**(6): 563-71.
2724. Morita A, Iki M, Dohi Y, Ikeda Y, Kagamimori S, Kagawa Y, et al. Effects of the Cdx-2 polymorphism of the vitamin D receptor gene and lifestyle factors on bone

mineral density in a representative sample of Japanese women: the Japanese Population-based Osteoporosis (JPOS) Study. Calcified Tissue International. 2005; **77**(6): 339-47.
2725. Morita M, Le Marchand L, Kono S, Yin G, Toyomura K, Nagano J, et al. Genetic polymorphisms of CYP2E1 and risk of colorectal cancer: the Fukuoka Colorectal Cancer Study. Cancer Epidemiology, Biomarkers & Prevention: A Publication Of The American Association For Cancer Research, Cosponsored By The American Society Of Preventive Oncology. 2009; **18**(1): 235-41.
2726. Morshedi M, Valenlia KB, Hosseinifard ES, Shahabi P, Abbasi MM, Ghorbani M, et al. Beneficial psychological effects of novel psychobiotics in diabetic rats: the interaction among the gut, blood and amygdala. The Journal Of Nutritional Biochemistry. 2018; **57**: 145-52.
2727. Mössner R, Daniel S, Schmitt A, Albert D, Lesch KP. Modulation of serotonin transporter function by interleukin-4. Life Sciences. 2001; **68**(8): 873-80.
2728. Mouro FM, Batalha VL, Ferreira DG, Coelho JE, Baqi Y, Muller CE, et al. Chronic and acute adenosine A2A receptor blockade prevents long-term episodic memory disruption caused by acute cannabinoid CB1 receptor activation. Neuropharmacology. 2017; **117**: 316-27.
2729. Mukherjee K, Edgett BA, Burrows HW, Castro C, Griffin JL, Schwertani AG, et al. Whole blood transcriptomics and urinary metabolomics to define adaptive biochemical pathways of high-intensity exercise in 50-60 year old masters athletes. PLoS One. 2014; **9**(3): e92031-e.
2730. Mulle JG, Gambello MJ, Cook EH, Rutkowski TP, Glassford M. 3q29 Recurrent Deletion. 1993.
2731. Muller JM, Morelli E, Ansorge M, Gingrich JA. Serotonin transporter deficient mice are vulnerable to escape deficits following inescapable shocks. Genes, Brain, And Behavior. 2011; **10**(2): 166-75.
2732. Munch IC, Toft U, Linneberg A, Larsen M. Precursors of age-related macular degeneration: associations with vitamin A and interaction with CFHY402H in the Inter99 Eye Study. Acta Ophthalmologica. 2016; **94**(7): 657-62.
2733. Murakami H, Ota A, Simojo H, Okada M, Ajisaka R, Kuno S. Polymorphisms in control region of mtDNA relates to individual differences in endurance capacity or trainability. The Japanese Journal Of Physiology. 2002; **52**(3): 247-56.
2734. Murakami H, Soma R, Hayashi J, Katsuta S, Matsuda M, Ajisaka R, et al. Relationship between mitochondrial DNA polymorphism and the individual differences in aerobic performance. The Japanese Journal Of Physiology. 2001; **51**(5): 563-8.
2735. Murata Y, Kobayashi D, Imuta N, Haraguchi K, Ieiri I, Nishimura R, et al. Effects of the serotonin 1A, 2A, 2C, 3A, and 3B and serotonin transporter gene polymorphisms on the occurrence of paroxetine discontinuation syndrome. Journal Of Clinical Psychopharmacology. 2010; **30**(1): 11-7.
2736. Musumeci M, Vadalà G, Tringali G, Insirello E, Roccazzello AM, Simpore J, et al. Genetic and environmental factors in human osteoporosis from Sub-Saharan to Mediterranean areas. Journal Of Bone And Mineral Metabolism. 2009; **27**(4): 424-34.
2737. Nader GA, Dastmalchi M, Alexanderson H, Grundtman C, Gernapudi R, Esbjörnsson M, et al. A longitudinal, integrated, clinical, histological and mRNA profiling

study of resistance exercise in myositis. Molecular Medicine (Cambridge, Mass). 2010; **16**(11-12): 455-64.
2738. Nakagawa-Senda H, Hachiya T, Shimizu A, Hosono S, Oze I, Watanabe M, et al. A genome-wide association study in the Japanese population identifies the 12q24 locus for habitual coffee consumption: The J-MICC Study. Scientific Reports. 2018; **8**(1): 1493-.
2739. Nakazono K, Watanabe Y, Nakaya S, Asami Y, Masuhara K, Itoh F, et al. Impairment state of cognitive performance and the affecting factors in outpatients following gastrointestinal endoscopy after single-dose diazepam. Yakugaku Zasshi: Journal Of The Pharmaceutical Society Of Japan. 2005; **125**(3): 307-14.
2740. Narasimhan S, Aquino TD, Multani PK, Rickels K, Lohoff FW. Variation in the catechol-O-methyltransferase (COMT) gene and treatment response to venlafaxine XR in generalized anxiety disorder. Psychiatry Research. 2012; **198**(1): 112-5.
2741. Nascimento CMC, Pereira JR, Pires de Andrade L, Garuffi M, Ayan C, Kerr DS, et al. Physical exercise improves peripheral BDNF levels and cognitive functions in mild cognitive impairment elderly with different bdnf Val66Met genotypes. Journal Of Alzheimer's Disease: JAD. 2015; **43**(1): 81-91.
2742. Nelson TL, Fingerlin TE, Moss L, Barmada MM, Ferrell RE, Norris JM. The peroxisome proliferator-activated receptor gamma coactivator-1 alpha gene (PGC-1alpha) is not associated with type 2 diabetes mellitus or body mass index among Hispanic and non Hispanic Whites from Colorado. Experimental And Clinical Endocrinology & Diabetes: Official Journal, German Society Of Endocrinology [And] German Diabetes Association. 2007; **115**(4): 268-75.
2743. Nelson TL, Fingerlin TE, Moss L, Barmada MM, Ferrell RE, Norris JM. The PPARgamma Pro12Ala polymorphism is not associated with body mass index or waist circumference among Hispanics from Colorado. Annals Of Nutrition & Metabolism. 2007; **51**(3): 252-7.
2744. Nelson TL, Stevens JR, Hickey MS. Adiponectin levels are reduced, independent of polymorphisms in the adiponectin gene, after supplementation with alpha-linolenic acid among healthy adults. Metabolism: Clinical And Experimental. 2007; **56**(9): 1209-15.
2745. Nentwig TB, Wilson DE, Rhinehart EM, Grisel JE. Sex differences in binge-like EtOH drinking, corticotropin-releasing hormone and corticosterone: effects of β-endorphin. Addiction Biology. 2018.
2746. Ng SK, Higashimori H, Tolman M, Yang Y. Suppression of adenosine 2a receptor (A2aR)-mediated adenosine signaling improves disease phenotypes in a mouse model of amyotrophic lateral sclerosis. Experimental Neurology. 2015; **267**: 115-22.
2747. Nichol K, Deeny SP, Seif J, Camaclang K, Cotman CW. Exercise improves cognition and hippocampal plasticity in APOE epsilon4 mice. Alzheimer's & Dementia: The Journal Of The Alzheimer's Association. 2009; **5**(4): 287-94.
2748. Nickels S, Truong T, Hein R, Stevens K, Buck K, Behrens S, et al. Evidence of gene-environment interactions between common breast cancer susceptibility loci and established environmental risk factors. Plos Genetics. 2013; **9**(3): e1003284-e.
2749. Nicoletti CF, de Oliveira APRP, Brochado MJF, de Oliveira BP, Pinhel MAdS, Marchini JS, et al. UCP1 -3826 A>G polymorphism affects weight, fat mass, and risk of

type 2 diabetes mellitus in grade III obese patients. Nutrition (Burbank, Los Angeles County, Calif). 2016; **32**(1): 83-7.
2750. Nikpay M, Šeda O, Tremblay J, Petrovich M, Gaudet D, Kotchen TA, et al. Genetic mapping of habitual substance use, obesity-related traits, responses to mental and physical stress, and heart rate and blood pressure measurements reveals shared genes that are overrepresented in the neural synapse. Hypertension Research: Official Journal Of The Japanese Society Of Hypertension. 2012; **35**(6): 585-91.
2751. Nishida Y, Iyadomi M, Higaki Y, Tanaka H, Kondo Y, Otsubo H, et al. Association between the PPARGC1A polymorphism and aerobic capacity in Japanese middle-aged men. Internal Medicine (Tokyo, Japan). 2015; **54**(4): 359-66.
2752. Nolan SO, Reynolds CD, Smith GD, Holley AJ, Escobar B, Chandler MA, et al. Deletion of Fmr1 results in sex-specific changes in behavior. Brain Behav. 2017; **7**(10): e00800.
2753. Nomura A, Zhang M, Sakamoto T, Ishii Y, Morishima Y, Mochizuki M, et al. Anti-inflammatory activity of creatine supplementation in endothelial cells in vitro. British Journal Of Pharmacology. 2003; **139**(4): 715-20.
2754. Nordstrom SM, Carleton SM, Carson WL, Eren M, Phillips CL, Vaughan DE. Transgenic over-expression of plasminogen activator inhibitor-1 results in age-dependent and gender-specific increases in bone strength and mineralization. Bone. 2007; **41**(6): 995-1004.
2755. Novoselova TV, Larder R, Rimmington D, Lelliott C, Wynn EH, Gorrigan RJ, et al. Loss of Mrap2 is associated with Sim1 deficiency and increased circulating cholesterol. The Journal Of Endocrinology. 2016; **230**(1): 13-26.
2756. Odebrecht Vargas Nunes S, Pizzo de Castro MR, Ehara Watanabe MA, Losi Guembarovski R, Odebrecht Vargas H, Reiche EMV, et al. Genetic polymorphisms in glutathione-S-transferases are associated with anxiety and mood disorders in nicotine dependence. Psychiatric Genetics. 2014; **24**(3): 87-93.
2757. Oftedal KN, Tepper BJ. Influence of the PROP bitter taste phenotype and eating attitudes on energy intake and weight status in pre-adolescents: a 6-year follow-up study. Physiology & Behavior. 2013; **118**: 103-11.
2758. Okayama M, Takeshima T, Ae R, Harada M, Kajii E. Primary care patient willingness for genetic testing for salt-sensitive hypertension: a cross sectional study. BMC Family Practice. 2013; **14**: 149-.
2759. O'Leary RE, Shih JC, Hyland K, Kramer N, Asher YJT, Graham JM, Jr. De novo microdeletion of Xp11.3 exclusively encompassing the monoamine oxidase A and B genes in a male infant with episodic hypotonia: a genomics approach to personalized medicine. European Journal Of Medical Genetics. 2012; **55**(5): 349-53.
2760. Olive MF, Mehmert KK, Koenig HN, Camarini R, Kim JA, Nannini MA, et al. A role for corticotropin releasing factor (CRF) in ethanol consumption, sensitivity, and reward as revealed by CRF-deficient mice. Psychopharmacology. 2003; **165**(2): 181-7.
2761. Oliveira FF, Machado FC, Sampaio G, Marin SMC, Chen ES, Smith MC, et al. Contrasts Between Patients With Lewy Body Dementia Syndromes and APOE-ε3/ε3 Patients With Late-onset Alzheimer Disease Dementia. The Neurologist. 2015; **20**(2): 35-41.
2762. Oliveira TdQ, de Sousa CNS, Vasconcelos GS, de Sousa LC, de Oliveira AA,

Patrocínio CFV, et al. Brain antioxidant effect of mirtazapine and reversal of sedation by its combination with alpha-lipoic acid in a model of depression induced by corticosterone. Journal Of Affective Disorders. 2017; **219**: 49-57.
2763. Olsson CA, Byrnes GB, Anney RJL, Collins V, Hemphill SA, Williamson R, et al. COMT Val(158)Met and 5HTTLPR functional loci interact to predict persistence of anxiety across adolescence: results from the Victorian Adolescent Health Cohort Study. Genes, Brain, And Behavior. 2007; **6**(7): 647-52.
2764. Olsson CA, Byrnes GB, Lotfi-Miri M, Collins V, Williamson R, Patton C, et al. Association between 5-HTTLPR genotypes and persisting patterns of anxiety and alcohol use: results from a 10-year longitudinal study of adolescent mental health. Molecular Psychiatry. 2005; **10**(9): 868-76.
2765. Omair A, Lie BA, Reikeras O, Holden M, Brox JI. Genetic contribution of catechol-O-methyltransferase variants in treatment outcome of low back pain: a prospective genetic association study. BMC Musculoskeletal Disorders. 2012; **13**: 76-.
2766. Onkelinx S, Cornelissen V, Defoor J, Matthijs G, Thomaes T, Coeckelberghs E, et al. The CAREGENE study: genetic variants of the endothelium and aerobic power in patients with coronary artery disease. Acta Cardiologica. 2011; **66**(4): 407-14.
2767. Oppert JM, Dussault JH, Tremblay A, Després JP, Thériault G, Bouchard C. Thyroid hormones and thyrotropin variations during long term overfeeding in identical twins. The Journal Of Clinical Endocrinology And Metabolism. 1994; **79**(2): 547-53.
2768. Østergård T, Ek J, Hamid Y, Saltin B, Pedersen OB, Hansen T, et al. Influence of the PPAR-gamma2 Pro12Ala and ACE I/D polymorphisms on insulin sensitivity and training effects in healthy offspring of type 2 diabetic subjects. Hormone And Metabolic Research = Hormon- Und Stoffwechselforschung = Hormones Et Metabolisme. 2005; **37**(2): 99-105.
2769. Ott C, Schwarz T, Hilgers KF, Kreutz R, Schlaich MP, Schmieder RE. Left-ventricular structure and function are influenced by angiotensinogen gene polymorphism (-20 A/C) in young male patients. American Journal Of Hypertension. 2007; **20**(9): 974-80.
2770. Outhred T, Das P, Dobson-Stone C, Felmingham KL, Bryant RA, Nathan PJ, et al. The impact of 5-HTTLPR on acute serotonin transporter blockade by escitalopram on emotion processing: preliminary findings from a randomised, crossover fMRI study. The Australian And New Zealand Journal Of Psychiatry. 2014; **48**(12): 1115-25.
2771. Oz T, Guvenek A, Yildiz S, Karaboga E, Tamer YT, Mumcuyan N, et al. Strength of selection pressure is an important parameter contributing to the complexity of antibiotic resistance evolution. Molecular Biology And Evolution. 2014; **31**(9): 2387-401.
2772. Paddock S, Laje G, Charney D, Rush AJ, Wilson AF, Sorant AJM, et al. Association of GRIK4 with outcome of antidepressant treatment in the STAR*D cohort. The American Journal Of Psychiatry. 2007; **164**(8): 1181-8.
2773. Pakhale S, Baron J, Dent R, Vandemheen K, Aaron SD. Effects of weight loss on airway responsiveness in obese adults with asthma: does weight loss lead to reversibility of asthma? Chest. 2015; **147**(6): 1582-90.
2774. Panek M, Pietras T, Witusik A, Wieteska Ł, Małachowska B, Mokros Ł, et al. Identification and association of relationships between selected personal and environmental factors and formal components of temperament and strategies of coping

with stress in asthmatic patients. Physiology & Behavior. 2015; **149**: 269-78.
2775. Panetta P, Berry A, Bellisario V, Capoccia S, Raggi C, Luoni A, et al. Long-Term Sex-Dependent Vulnerability to Metabolic challenges in Prenatally Stressed Rats. Front Behav Neurosci. 2017; **11**: 113.
2776. Papolos D, Mattis S, Golshan S, Molay F. Fear of harm, a possible phenotype of pediatric bipolar disorder: a dimensional approach to diagnosis for genotyping psychiatric syndromes. Journal Of Affective Disorders. 2009; **118**(1-3): 28-38.
2777. Park ER, Kleimann S, Pelan JA, Shields AE. Anticipating clinical integration of genetically tailored tobacco dependence treatment: perspectives of primary care physicians. Nicotine & Tobacco Research: Official Journal Of The Society For Research On Nicotine And Tobacco. 2007; **9**(2): 271-9.
2778. Park J-Y, Kim K-A, Park P-W, Lee O-J, Kang D-K, Shon J-H, et al. Effect of CYP3A5*3 genotype on the pharmacokinetics and pharmacodynamics of alprazolam in healthy subjects. Clinical Pharmacology And Therapeutics. 2006; **79**(6): 590-9.
2779. Partadiredja G, Karima N, Utami KP, Agustiningsih D, Sofro ZM. THE EFFECTS OF LIGHT AND MODERATE INTENSITY EXERCISE ON THE FEMORAL BONE AND CEREBELLUM OF D-GALACTOSE-EXPOSED RATS. Rejuvenation Research. 2018.
2780. Pasaje CFA, Bae JS, Park B-L, Cheong HS, Kim J-H, Jang A-S, et al. DCBLD2 gene variations correlate with nasal polyposis in Korean asthma patients. Lung. 2012; **190**(2): 199-207.
2781. Pascual R, Valencia M, Bustamante C. Antenatal betamethasone produces protracted changes in anxiety-like behaviors and in the expression of microtubule-associated protein 2, brain-derived neurotrophic factor and the tyrosine kinase B receptor in the rat cerebellar cortex. International Journal Of Developmental Neuroscience: The Official Journal Of The International Society For Developmental Neuroscience. 2015; **43**: 78-85.
2782. Pastore A, Jurinovic V, Kridel R, Hoster E, Staiger AM, Szczepanowski M, et al. Integration of gene mutations in risk prognostication for patients receiving first-line immunochemotherapy for follicular lymphoma: a retrospective analysis of a prospective clinical trial and validation in a population-based registry. The Lancet Oncology. 2015; **16**(9): 1111-22.
2783. Patel SS, Ray RS, Sharma A, Mehta V, Katyal A, Udayabanu M. Antidepressant and anxiolytic like effects of Urtica dioica leaves in streptozotocin induced diabetic mice. Metabolic Brain Disease. 2018.
2784. Pattaro C, Gögele M, Mascalzoni D, Melotti R, Schwienbacher C, De Grandi A, et al. The Cooperative Health Research in South Tyrol (CHRIS) study: rationale, objectives, and preliminary results. Journal Of Translational Medicine. 2015; **13**: 348-.
2785. Pearson R, Palmer RHC, Brick LA, McGeary JE, Knopik VS, Beevers CG. Additive genetic contribution to symptom dimensions in major depressive disorder. Journal Of Abnormal Psychology. 2016; **125**(4): 495-501.
2786. Pedersen NH, Tarp J, Andersen LB, Gejl AK, Huang T, Peijs L, et al. The association between serum brain-derived neurotrophic factor and a cluster of cardiovascular risk factors in adolescents: The CHAMPS-study DK. PLoS One. 2017; **12**(10): e0186384-e.
2787. Peeters RP, van den Beld AW, van Toor H, Uitterlinden AG, Janssen JAMJL,

Lamberts SWJ, et al. A polymorphism in type I deiodinase is associated with circulating free insulin-like growth factor I levels and body composition in humans. The Journal Of Clinical Endocrinology And Metabolism. 2005; **90**(1): 256-63.
2788. Pekkinen M, Saarnio E, Viljakainen HT, Kokkonen E, Jakobsen J, Cashman K, et al. Vitamin D binding protein genotype is associated with serum 25-hydroxyvitamin D and PTH concentrations, as well as bone health in children and adolescents in Finland. PLoS One. 2014; **9**(1): e87292-e.
2789. Pelletier R, Pravica V, Perrey C, Xia D, Ferguson RM, Hutchinson I, et al. Evidence for a genetic predisposition towards acute rejection after kidney and simultaneous kidney-pancreas transplantation. Transplantation. 2000; **70**(4): 674-80.
2790. Pereira A, Costa AM, Leitão JC, Monteiro AM, Izquierdo M, Silva AJ, et al. The influence of ACE ID and ACTN3 R577X polymorphisms on lower-extremity function in older women in response to high-speed power training. BMC Geriatrics. 2013; **13**: 131-.
2791. Pereira M, Martynhak BJ, Andreatini R, Svenningsson P. 5-HT6 receptor agonism facilitates emotional learning. Front Pharmacol. 2015; **6**: 200.
2792. Perlis RH, Fijal B, Dharia S, Houston JP. Pharmacogenetic investigation of response to duloxetine treatment in generalized anxiety disorder. The Pharmacogenomics Journal. 2013; **13**(3): 280-5.
2793. Persi A, Maltese PE, Bertelli M, Cecchin S, Ciaghi M, Guarnieri MC, et al. Polymorphisms of alpha-actinin-3 and ciliary neurotrophic factor in national-level Italian athletes. Panminerva Medica. 2013; **55**(2): 217-24.
2794. Perwitasari DA, van der Straaten RJHM, Mustofa M, Wessels JAM, Gelderblom H, Guchelaar H-J. Differences in 5-hydroxytryptamine-3B haplotype frequencies between Asians and Caucasians. The International Journal Of Biological Markers. 2012; **27**(1): 34-8.
2795. Pescatello LS, Blanchard BE, Tsongalis GJ, O'Connell AA, Gordish-Dressman H, Maresh CM, et al. A comparison of the genetic and clinical profile of men that respond and do not respond to the immediate antihypertensive effects of aerobic exercise. The Application Of Clinical Genetics. 2008; **1**: 7-17.
2796. Pescatello LS, Turner D, Rodriguez N, Blanchard BE, Tsongalis GJ, Maresh CM, et al. Dietary calcium intake and renin angiotensin system polymorphisms alter the blood pressure response to aerobic exercise: a randomized control design. Nutrition & Metabolism. 2007; **4**: 1-.
2797. Peter I, Papandonatos GD, Belalcazar LM, Yang Y, Erar B, Jakicic JM, et al. Genetic modifiers of cardiorespiratory fitness response to lifestyle intervention. Medicine And Science In Sports And Exercise. 2014; **46**(2): 302-11.
2798. Petersen RA, Larsen LH, Damsgaard CT, Sørensen LB, Hjorth MF, Andersen R, et al. Common genetic variants are associated with lower serum 25-hydroxyvitamin D concentrations across the year among children at northern latitudes. The British Journal Of Nutrition. 2017; **117**(6): 829-38.
2799. Pflanz D, Birkhold AI, Albiol L, Thiele T, Julien C, Seliger A, et al. Sost deficiency led to a greater cortical bone formation response to mechanical loading and altered gene expression. Scientific Reports. 2017; **7**(1): 9435-.
2800. Picanço JB, Raimann PE, Motta CHASd, Rodenbusch R, Gusmão L, Alho CS. Identification of the third/extra allele for forensic application in cases with TPOX tri-allelic

pattern. Forensic Science International Genetics. 2015; **16**: 88-93.
2801. Pickering C. Caffeine, CYP1A2 genotype, and sports performance: is timing important? Irish Journal Of Medical Science. 2018.
2802. Pietras T, Witusik A, Panek M, Galecki P, Szemraj J, Gorski P. [Anxiety, depression and polymorphism of the gene encoding superoxide dismutase in patients with chronic obstructive pulmonary disease]. Pol Merkur Lekarski. 2010; **29**(171): 165-8.
2803. Pietrzak RH, Lim YY, Neumeister A, Ames D, Ellis KA, Harrington K, et al. Amyloid-beta, anxiety, and cognitive decline in preclinical Alzheimer disease: a multicenter, prospective cohort study. JAMA Psychiatry. 2015; **72**(3): 284-91.
2804. Pietrzak RH, Lim YY, Neumeister A, Ames D, Ellis KA, Harrington K, et al. Amyloid-β, anxiety, and cognitive decline in preclinical Alzheimer disease: a multicenter, prospective cohort study. JAMA Psychiatry. 2015; **72**(3): 284-91.
2805. Pilling LC, Harries LW, Powell J, Llewellyn DJ, Ferrucci L, Melzer D. Genomics and successful aging: grounds for renewed optimism? The Journals Of Gerontology Series A, Biological Sciences And Medical Sciences. 2012; **67**(5): 511-9.
2806. Pimenta EM, Coelho DB, Veneroso CE, Barros Coelho EJ, Cruz IR, Morandi RF, et al. Effect of ACTN3 gene on strength and endurance in soccer players. Journal Of Strength And Conditioning Research. 2013; **27**(12): 3286-92.
2807. Pisciotta L, Cantafora A, Piana A, Masturzo P, Cerone R, Minniti G, et al. Physical activity modulates effects of some genetic polymorphisms affecting cardiovascular risk in men aged over 40 years. Nutrition, Metabolism, And Cardiovascular Diseases: NMCD. 2003; **13**(4): 202-10.
2808. Poehlman ET, Tremblay A, Marcotte M, Pérusse L, Thériault G, Bouchard C. Heredity and changes in body composition and adipose tissue metabolism after short-term exercise-training. European Journal Of Applied Physiology And Occupational Physiology. 1987; **56**(4): 398-402.
2809. Pollin TI, Isakova T, Jablonski KA, de Bakker PIW, Taylor A, McAteer J, et al. Genetic modulation of lipid profiles following lifestyle modification or metformin treatment: the Diabetes Prevention Program. Plos Genetics. 2012; **8**(8): e1002895-e.
2810. Pomara N, Willoughby LM, Hashim A, Sershen H, Sidtis JJ, Wesnes K, et al. Effects of acute lorazepam administration on aminergic activity in normal elderly subjects: relationship to performance effects and apolipoprotein genotype. Neurochemical Research. 2004; **29**(7): 1391-8.
2811. Pompeia S, Gouveia JR, Galduróz JCF. Acute mood effect of donepezil in young, healthy volunteers. Human Psychopharmacology. 2013; **28**(3): 263-9.
2812. Popoli M, Gennarelli M, Racagni G. Modulation of synaptic plasticity by stress and antidepressants. Bipolar Disorders. 2002; **4**(3): 166-82.
2813. Poppe LK, Chunda-Liyoka C, Kwon EH, Gondwe C, West JT, Kankasa C, et al. HIV drug resistance in infants increases with changing prevention of mother-to-child transmission regimens. AIDS (London, England). 2017; **31**(13): 1885-9.
2814. Potapov AL, Boiarkina AV. [M1-OPIOID RECEPTOR AND CATECHOL-O-METILTRANSFERASE GENES POLYMORPHISM EFFECTS ON PERIOPERATIVE PSYCHOLOGICAL CONDITION OF THE PATIENTS AND THE EFFECTIVENESS OF POSTOPERATIVE ANALGESIA WITH OPIOIDS]. Anesteziologiia I Reanimatologiia. 2015; **60**(3): 48-51.

2815. Potkin SG, Preskorn S, Hochfeld M, Meng X. A thorough QTc study of 3 doses of iloperidone including metabolic inhibition via CYP2D6 and/or CYP3A4 and a comparison to quetiapine and ziprasidone. Journal Of Clinical Psychopharmacology. 2013; **33**(1): 3-10.
2816. Pouwels S, de Boer A, Javaid MK, Hilton-Jones D, Verschuuren J, Cooper C, et al. Fracture rate in patients with myasthenia gravis: the general practice research database. Osteoporosis International: A Journal Established As Result Of Cooperation Between The European Foundation For Osteoporosis And The National Osteoporosis Foundation Of The USA. 2013; **24**(2): 467-76.
2817. Pratt JD, Datu A, Tran T, Sheng DC, Mooney KA. Genetically based latitudinal clines in Artemisia californica drive parallel clines in arthropod communities. Ecology. 2017; **98**(1): 79-91.
2818. Prediger RDS, da Silva GE, Batista LC, Bittencourt AL, Takahashi RN. Activation of adenosine A1 receptors reduces anxiety-like behavior during acute ethanol withdrawal (hangover) in mice. Neuropsychopharmacology: Official Publication Of The American College Of Neuropsychopharmacology. 2006; **31**(10): 2210-20.
2819. Prior SJ, Goldberg AP, Ryan AS. ADRB2 haplotype is associated with glucose tolerance and insulin sensitivity in obese postmenopausal women. Obesity (Silver Spring, Md). 2011; **19**(2): 396-401.
2820. Prior SJ, Hagberg JM, Paton CM, Douglass LW, Brown MD, McLenithan JC, et al. DNA sequence variation in the promoter region of the VEGF gene impacts VEGF gene expression and maximal oxygen consumption. American Journal Of Physiology Heart And Circulatory Physiology. 2006; **290**(5): H1848-H55.
2821. Prior SJ, Hagberg JM, Phares DA, Brown MD, Fairfull L, Ferrell RE, et al. Sequence variation in hypoxia-inducible factor 1alpha (HIF1A): association with maximal oxygen consumption. Physiological Genomics. 2003; **15**(1): 20-6.
2822. Pytka K, Gluch-Lutwin M, Kotanska M, Zmudzka E, Jakubczyk M, Waszkielewicz A, et al. HBK-15 protects mice from stress-induced behavioral disturbances and changes in corticosterone, BDNF, and NGF levels. Behav Brain Res. 2017; **333**: 54-66.
2823. Qi L, Li T, Rimm E, Zhang C, Rifai N, Hunter D, et al. The +276 polymorphism of the APM1 gene, plasma adiponectin concentration, and cardiovascular risk in diabetic men. Diabetes. 2005; **54**(5): 1607-10.
2824. Qin XP, Xie HG, Wang W, He N, Huang SL, Xu ZH, et al. Effect of the gene dosage of CgammaP2C19 on diazepam metabolism in Chinese subjects. Clinical Pharmacology And Therapeutics. 1999; **66**(6): 642-6.
2825. Queiroz DR, Farias FJC, Cavalcanti JJV, Carvalho LP, Neder DG, Souza LSS, et al. Diallel analysis for technological traits in upland cotton. Genetics And Molecular Research: GMR. 2017; **16**(3).
2826. Raghuram GV, Gupta D, Subramaniam S, Gaikwad A, Khare NK, Nobre M, et al. Physical shearing imparts biological activity to DNA and ability to transmit itself horizontally across species and kingdom boundaries. BMC Molecular Biology. 2017; **18**(1): 21-.
2827. Rampersaud E, Nathanson L, Farmer J, Meshbane K, Belton RL, Dressen A, et al. Genomic signatures of a global fitness index in a multi-ethnic cohort of women. Annals Of Human Genetics. 2013; **77**(2): 147-57.

2828. Randolph GP, Simon JS, Arreaza MG, Qiu P, Lachowicz JE, Duffy RA. Identification of single-nucleotide polymorphisms of the human neurokinin 1 receptor gene and pharmacological characterization of a Y192H variant. The Pharmacogenomics Journal. 2004; **4**(6): 394-402.
2829. Rankinen T, Pérusse L, Borecki I, Chagnon YC, Gagnon J, Leon AS, et al. The Na(+)-K(+)-ATPase alpha2 gene and trainability of cardiorespiratory endurance: the HERITAGE family study. Journal Of Applied Physiology (Bethesda, Md: 1985). 2000; **88**(1): 346-51.
2830. Rankinen T, Pérusse L, Gagnon J, Chagnon YC, Leon AS, Skinner JS, et al. Angiotensin-converting enzyme ID polymorphism and fitness phenotype in the HERITAGE Family Study. Journal Of Applied Physiology (Bethesda, Md: 1985). 2000; **88**(3): 1029-35.
2831. Raskind WH, Bolin T, Wolff J, Fink J, Matsushita M, Litt M, et al. Further localization of a gene for paroxysmal dystonic choreoathetosis to a 5-cM region on chromosome 2q34. Human Genetics. 1998; **102**(1): 93-7.
2832. Rasmussen A, Alonso E, Ochoa A, De Biase I, Familiar I, Yescas P, et al. Uptake of genetic testing and long-term tumor surveillance in von Hippel-Lindau disease. BMC Medical Genetics. 2010; **11**: 4-.
2833. Rasmussen MA, Skov J, Bladbjerg E-M, Sidelmann JJ, Vamosi M, Jespersen J. Multivariate analysis of the relation between diet and warfarin dose. European Journal Of Clinical Pharmacology. 2012; **68**(3): 321-8.
2834. Rauch F, Radermacher A, Danz A, Schiedermaier U, Golücke A, Michalk D, et al. Vitamin D receptor genotypes and changes of bone density in physically active German women with high calcium intake. Experimental And Clinical Endocrinology & Diabetes: Official Journal, German Society Of Endocrinology [And] German Diabetes Association. 1997; **105**(2): 103-8.
2835. Ravegnini G, Zolezzi Moraga JM, Maffei F, Musti M, Zenesini C, Simeon V, et al. Simultaneous Analysis of SEPT9 Promoter Methylation Status, Micronuclei Frequency, and Folate-Related Gene Polymorphisms: The Potential for a Novel Blood-Based Colorectal Cancer Biomarker. International Journal Of Molecular Sciences. 2015; **16**(12): 28486-97.
2836. Ravussin E. Energy metabolism in obesity. Studies in the Pima Indians. Diabetes Care. 1993; **16**(1): 232-8.
2837. Ray LA, MacKillop J, Hutchison KE. Pharmacogenetics of the μ-opioid receptor and the treatment of addictions. Personalized Medicine. 2007; **4**(2): 217-20.
2838. Rees JR, Mott LA, Barry EL, Baron JA, Bostick RM, Figueiredo JC, et al. Lifestyle and Other Factors Explain One-Half of the Variability in the Serum 25-Hydroxyvitamin D Response to Cholecalciferol Supplementation in Healthy Adults. The Journal Of Nutrition. 2016; **146**(11): 2312-24.
2839. Reif A, Richter J, Straube B, Höfler M, Lueken U, Gloster AT, et al. MAOA and mechanisms of panic disorder revisited: from bench to molecular psychotherapy. Molecular Psychiatry. 2014; **19**(1): 122-8.
2840. Rendeiro C, Foley A, Lau VC, Ring R, Rodriguez-Mateos A, Vauzour D, et al. A role for hippocampal PSA-NCAM and NMDA-NR2B receptor function in flavonoid-induced spatial memory improvements in young rats. Neuropharmacology. 2014; **79**:

335-44.
2841. Renton M, Diggle A, Manalil S, Powles S. Does cutting herbicide rates threaten the sustainability of weed management in cropping systems? Journal Of Theoretical Biology. 2011; **283**(1): 14-27.
2842. Rétey JV, Adam M, Honegger E, Khatami R, Luhmann UFO, Jung HH, et al. A functional genetic variation of adenosine deaminase affects the duration and intensity of deep sleep in humans. Proceedings Of The National Academy Of Sciences Of The United States Of America. 2005; **102**(43): 15676-81.
2843. Rétey JV, Adam M, Khatami R, Luhmann UFO, Jung HH, Berger W, et al. A genetic variation in the adenosine A2A receptor gene (ADORA2A) contributes to individual sensitivity to caffeine effects on sleep. Clinical Pharmacology And Therapeutics. 2007; **81**(5): 692-8.
2844. Revera M, van der Merwe L, Heradien M, Goosen A, Corfield VA, Brink PA, et al. Troponin T and beta-myosin mutations have distinct cardiac functional effects in hypertrophic cardiomyopathy patients without hypertrophy. Cardiovascular Research. 2008; **77**(4): 687-94.
2845. Ribeiro RV, Machado EC, Magalhães Filho JR, Lobo AKM, Martins MO, Silveira JAG, et al. Increased sink strength offsets the inhibitory effect of sucrose on sugarcane photosynthesis. Journal Of Plant Physiology. 2017; **208**: 61-9.
2846. Richards ML, Davies PS, Bell SC. Energy cost of physical activity in cystic fibrosis. European Journal Of Clinical Nutrition. 2001; **55**(8): 690-7.
2847. Richardson-Jones JW, Craige CP, Guiard BP, Stephen A, Metzger KL, Kung HF, et al. 5-HT1A autoreceptor levels determine vulnerability to stress and response to antidepressants. Neuron. 2010; **65**(1): 40-52.
2848. Rico-Sanz J, Rankinen T, Joanisse DR, Leon AS, Skinner JS, Wilmore JH, et al. Associations between cardiorespiratory responses to exercise and the C34T AMPD1 gene polymorphism in the HERITAGE Family Study. Physiological Genomics. 2003; **14**(2): 161-6.
2849. Rico-Sanz J, Rankinen T, Rice T, Leon AS, Skinner JS, Wilmore JH, et al. Quantitative trait loci for maximal exercise capacity phenotypes and their responses to training in the HERITAGE Family Study. Physiological Genomics. 2004; **16**(2): 256-60.
2850. Rivera MA, Dionne FT, Simoneau JA, Pérusse L, Chagnon M, Chagnon Y, et al. Muscle-specific creatine kinase gene polymorphism and VO2max in the HERITAGE Family Study. Medicine And Science In Sports And Exercise. 1997; **29**(10): 1311-7.
2851. Rivera MA, Echegaray M, Rankinen T, Pérusse L, Rice T, Gagnon J, et al. Angiogenin gene-race interaction for resting and exercise BP phenotypes: the HERITAGE Family Study. Journal Of Applied Physiology (Bethesda, Md: 1985). 2001; **90**(4): 1232-8.
2852. Rivera MA, Echegaray M, Rankinen T, Pérusse L, Rice T, Gagnon J, et al. TGF-beta(1) gene-race interactions for resting and exercise blood pressure in the HERITAGE Family Study. Journal Of Applied Physiology (Bethesda, Md: 1985). 2001; **91**(4): 1808-13.
2853. Rivkees SA. Ending the late diagnosis of Turner syndrome through a novel high-throughput assay. Pediatric Endocrinology Reviews: PER. 2012; **9 Suppl 2**: 698-700.
2854. Rivkees SA, Hager K, Hosono S, Wise A, Li P, Rinder HM, et al. A highly

sensitive, high-throughput assay for the detection of Turner syndrome. The Journal Of Clinical Endocrinology And Metabolism. 2011; **96**(3): 699-705.
2855. Rodrigues C, Costa E, Vieira E, De Carvalho J, Santos R, Rocha-Pereira P, et al. Bilirubin dependence on UGT1A1 polymorphisms, hemoglobin, fasting time and body mass index. The American Journal Of The Medical Sciences. 2012; **343**(2): 114-8.
2856. Rodríguez-Navarro JA, Rodríguez L, Casarejos MJ, Solano RM, Gómez A, Perucho J, et al. Trehalose ameliorates dopaminergic and tau pathology in parkin deleted/tau overexpressing mice through autophagy activation. Neurobiology Of Disease. 2010; **39**(3): 423-38.
2857. Roe CR, Yang BZ, Brunengraber H, Roe DS, Wallace M, Garritson BK. Carnitine palmitoyltransferase II deficiency: successful anaplerotic diet therapy. Neurology. 2008; **71**(4): 260-4.
2858. Rojana-udomsart A, Needham M, Luo YB, Fabian V, Walters S, Zilko PJ, et al. The association of sporadic inclusion body myositis and Sjögren's syndrome in carriers of HLA-DR3 and the 8.1 MHC ancestral haplotype. Clinical Neurology And Neurosurgery. 2011; **113**(7): 559-63.
2859. Romero-Moraleda B, Cupeiro R, González-Lamuño D, Amigo T, Szendrei B, Rojo-Tirado MÁ, et al. Impact of APOE2allele on lipid profile change after a weight loss program. Nutricion Hospitalaria. 2018; **0**(0): 305-11.
2860. Rönn T, Volkov P, Tornberg A, Elgzyri T, Hansson O, Eriksson KF, et al. Extensive changes in the transcriptional profile of human adipose tissue including genes involved in oxidative phosphorylation after a 6-month exercise intervention. Acta Physiologica (Oxford, England). 2014; **211**(1): 188-200.
2861. Rotberg B, Kronenberg S, Carmel M, Frisch A, Brent D, Zalsman G, et al. Additive effects of 5-HTTLPR (serotonin transporter) and tryptophan hydroxylase 2 G-703T gene polymorphisms on the clinical response to citalopram among children and adolescents with depression and anxiety disorders. Journal Of Child And Adolescent Psychopharmacology. 2013; **23**(2): 117-22.
2862. Rubio JC, Pérez M, Maté-Muñoz JL, García-Consuegra I, Chamorro-Viña C, Fernández del Valle M, et al. AMPD1 genotypes and exercise capacity in McArdle patients. International Journal Of Sports Medicine. 2008; **29**(4): 331-5.
2863. Ruixing Y, Jinzhen W, Shangling P, Weixiong L, Dezhai Y, Yuming C. Sex differences in environmental and genetic factors for hypertension. The American Journal Of Medicine. 2008; **121**(9): 811-9.
2864. Ruiz JR, Buxens A, Artieda M, Arteta D, Santiago C, Rodríguez-Romo G, et al. The -174 G/C polymorphism of the IL6 gene is associated with elite power performance. Journal Of Science And Medicine In Sport. 2010; **13**(5): 549-53.
2865. Rush DW, Epstein E. Genotypic Responses to Salinity: Differences between Salt-sensitive and Salt-tolerant Genotypes of the Tomato. Plant Physiology. 1976; **57**(2): 162-6.
2866. Russo A, Buratta L, Pippi R, Aiello C, Ranucci C, Reginato E, et al. Effect of Training Exercise on Urinary Brain-derived Neurotrophic Factor Levels and Cognitive Performances in Overweight and Obese Subjects. Psychological Reports. 2017; **120**(1): 70-87.
2867. Ryan J, Scali J, Carrière I, Scarabin P-Y, Ritchie K, Ancelin M-L. Estrogen

receptor gene variants are associated with anxiety disorders in older women. Psychoneuroendocrinology. 2011; **36**(10): 1582-6.
2868. Ryu Y-H, Kim Y-H, Lee J-Y, Shim G-B, Uhm H-S, Park G, et al. Effects of background fluid on the efficiency of inactivating yeast with non-thermal atmospheric pressure plasma. PLoS One. 2013; **8**(6): e66231-e.
2869. Saba NF, Wilson M, Doho G, DaSilva J, Benjamin Isett R, Newman S, et al. Mutation and Transcriptional Profiling of Formalin-Fixed Paraffin Embedded Specimens as Companion Methods to Immunohistochemistry for Determining Therapeutic Targets in Oropharyngeal Squamous Cell Carcinoma (OPSCC): A Pilot of Proof of Principle. Head And Neck Pathology. 2015; **9**(2): 223-35.
2870. Sagarkar S, Bhamburkar T, Shelkar G, Choudhary A, Kokare DM, Sakharkar AJ. Minimal traumatic brain injury causes persistent changes in DNA methylation at BDNF gene promoters in rat amygdala: A possible role in anxiety-like behaviors. Neurobiol Dis. 2017; **106**: 101-9.
2871. Sailer C, Schmid V, Fritsche L, Gerter T, Machicao F, Niess A, et al. FTO Genotype Interacts with Improvement in Aerobic Fitness on Body Weight Loss During Lifestyle Intervention. Obesity Facts. 2016; **9**(3): 174-81.
2872. Saito N, Fu J, Zheng S, Yao J, Wang S, Liu DD, et al. A high Notch pathway activation predicts response to gamma secretase inhibitors in proneural subtype of glioma tumor-initiating cells. Stem Cells. 2014; **32**(1): 301-12.
2873. Saito N, Fu J, Zheng S, Yao J, Wang S, Liu DD, et al. A high Notch pathway activation predicts response to γ secretase inhibitors in proneural subtype of glioma tumor-initiating cells. Stem Cells (Dayton, Ohio). 2014; **32**(1): 301-12.
2874. Sakurai T, Dorr NP, Takahashi N, McInnes LA, Elder GA, Buxbaum JD. Haploinsufficiency of Gtf2i, a gene deleted in Williams Syndrome, leads to increases in social interactions. Autism Research: Official Journal Of The International Society For Autism Research. 2011; **4**(1): 28-39.
2875. Salmen T, Heikkinen A-M, Mahonen A, Kröger H, Komulainen M, Pallonen H, et al. Relation of aromatase gene polymorphism and hormone replacement therapy to serum estradiol levels, bone mineral density, and fracture risk in early postmenopausal women. Annals Of Medicine. 2003; **35**(4): 282-8.
2876. Samaan Z, Bawor M, Dennis BB, Plater C, Varenbut M, Daiter J, et al. Genetic influence on methadone treatment outcomes in patients undergoing methadone maintenance treatment for opioid addiction: a pilot study. Neuropsychiatric Disease And Treatment. 2014; **10**: 1503-8.
2877. Samochowiec J, Kucharska-Mazur J, Grzywacz A, Jabłoński M, Rommelspacher H, Samochowiec A, et al. Family-based and case-control study of DRD2, DAT, 5HTT, COMT genes polymorphisms in alcohol dependence. Neuroscience Letters. 2006; **410**(1): 1-5.
2878. Sánchez-Iglesias S, García-Solaesa V, García-Berrocal B, Sanchez-Martín A, Lorenzo-Romo C, Martín-Pinto T, et al. Role of Pharmacogenetics in Improving the Safety of Psychiatric Care by Predicting the Potential Risks of Mania in CYP2D6 Poor Metabolizers Diagnosed With Bipolar Disorder. Medicine. 2016; **95**(6): e2473-e.
2879. Sandkovsky U, Podany AT, Fletcher CV, Owen A, Felton-Coleman A, Winchester LC, et al. Impact of efavirenz pharmacokinetics and pharmacogenomics on

neuropsychological performance in older HIV-infected patients. The Journal Of Antimicrobial Chemotherapy. 2017; **72**(1): 200-4.
2880. Santangelo AM, Ito M, Shiba Y, Clarke HF, Schut EH, Cockcroft G, et al. Novel Primate Model of Serotonin Transporter Genetic Polymorphisms Associated with Gene Expression, Anxiety and Sensitivity to Antidepressants. Neuropsychopharmacology: Official Publication Of The American College Of Neuropsychopharmacology. 2016; **41**(9): 2366-76.
2881. Santoro A, Siviero P, Minicuci N, Bellavista E, Mishto M, Olivieri F, et al. Effects of donepezil, galantamine and rivastigmine in 938 Italian patients with Alzheimer's disease: a prospective, observational study. CNS Drugs. 2010; **24**(2): 163-76.
2882. Santoro M, Nociti V, De Fino C, Caprara A, Giordano R, Palomba N, et al. Depression in multiple sclerosis: effect of brain derived neurotrophic factor Val66Met polymorphism and disease perception. European Journal Of Neurology. 2016; **23**(3): 630-40.
2883. Sarabi B, Bolandnazar S, Ghaderi N, Ghashghaie J. Genotypic differences in physiological and biochemical responses to salinity stress in melon (Cucumis melo L.) plants: Prospects for selection of salt tolerant landraces. Plant Physiology And Biochemistry: PPB. 2017; **119**: 294-311.
2884. Sarris J, Scholey A, Schweitzer I, Bousman C, Laporte E, Ng C, et al. The acute effects of kava and oxazepam on anxiety, mood, neurocognition; and genetic correlates: a randomized, placebo-controlled, double-blind study. Human Psychopharmacology. 2012; **27**(3): 262-9.
2885. Sarris J, Stough C, Bousman CA, Wahid ZT, Murray G, Teschke R, et al. Kava in the treatment of generalized anxiety disorder: a double-blind, randomized, placebo-controlled study. Journal Of Clinical Psychopharmacology. 2013; **33**(5): 643-8.
2886. Sarris J, Stough C, Teschke R, Wahid ZT, Bousman CA, Murray G, et al. Kava for the treatment of generalized anxiety disorder RCT: analysis of adverse reactions, liver function, addiction, and sexual effects. Phytotherapy Research: PTR. 2013; **27**(11): 1723-8.
2887. Savitz JB, Drevets WC. Imaging phenotypes of major depressive disorder: genetic correlates. Neuroscience. 2009; **164**(1): 300-30.
2888. Schaser AJ, Ciucci MR, Connor NP. Cross-activation and detraining effects of tongue exercise in aged rats. Behavioural Brain Research. 2016; **297**: 285-96.
2889. Schega L, Peter B, Brigadski T, Leßmann V, Isermann B, Hamacher D, et al. Effect of intermittent normobaric hypoxia on aerobic capacity and cognitive function in older people. Journal Of Science And Medicine In Sport. 2016; **19**(11): 941-5.
2890. Schepers R, Markus CR. The interaction between 5-HTTLPR genotype and ruminative thinking on BMI. The British Journal Of Nutrition. 2017; **118**(8): 629-37.
2891. Schmechel DE, Browndyke J, Ghio A. Strategies for dissecting genetic-environmental interactions in neurodegenerative disorders. Neurotoxicology. 2006; **27**(5): 637-57.
2892. Schmidt ME, Wiskemann J, Johnson T, Habermann N, Schneeweiss A, Steindorf K. L-Thyroxine intake as a potential risk factor for the development of fatigue in breast cancer patients undergoing chemotherapy. Supportive Care In Cancer: Official Journal Of The Multinational Association Of Supportive Care In Cancer. 2018; **26**(8): 2561-9.

2893. Schnurr TM, Gjesing AP, Sandholt CH, Jonsson A, Mahendran Y, Have CT, et al. Genetic Correlation between Body Fat Percentage and Cardiorespiratory Fitness Suggests Common Genetic Etiology. PLoS One. 2016; **11**(11): e0166738-e.
2894. Schosser A, Gaysina D, Cohen-Woods S, Domenici E, Perry J, Tozzi F, et al. A follow-up case-control association study of tractable (druggable) genes in recurrent major depression. American Journal Of Medical Genetics Part B, Neuropsychiatric Genetics: The Official Publication Of The International Society Of Psychiatric Genetics. 2011; **156B**(6): 640-50.
2895. Schruers K, Esquivel G, van Duinen M, Wichers M, Kenis G, Colasanti A, et al. Genetic moderation of CO2-induced fear by 5-HTTLPR genotype. Journal Of Psychopharmacology (Oxford, England). 2011; **25**(1): 37-42.
2896. Schunck RVA, Macedo IC, Laste G, de Souza A, Valle MTC, Salomón JLO, et al. Standardized Passiflora incarnata L. Extract Reverts the Analgesia Induced by Alcohol Withdrawal in Rats. Phytotherapy Research: PTR. 2017; **31**(8): 1199-208.
2897. Schüßler A, Krüger C, Urgiles N. Phylogenetically diverse AM fungi from Ecuador strongly improve seedling growth of native potential crop trees. Mycorrhiza. 2016; **26**(3): 199-207.
2898. Seale SM, Feng Q, Agarwal AK, El-Alfy AT. Neurobehavioral and transcriptional effects of acrylamide in juvenile rats. Pharmacology, Biochemistry, And Behavior. 2012; **101**(1): 77-84.
2899. Secco M, Bueno C, Jr., Vieira NM, Almeida C, Pelatti M, Zucconi E, et al. Systemic delivery of human mesenchymal stromal cells combined with IGF-1 enhances muscle functional recovery in LAMA2 dy/2j dystrophic mice. Stem Cell Reviews. 2013; **9**(1): 93-109.
2900. Segala E, Guo D, Cheng RKY, Bortolato A, Deflorian F, Doré AS, et al. Controlling the Dissociation of Ligands from the Adenosine A2A Receptor through Modulation of Salt Bridge Strength. Journal Of Medicinal Chemistry. 2016; **59**(13): 6470-9.
2901. Seng K-Y, Hee K-H, Soon GH, Sapari NS, Soong R, Goh B-C, et al. CYP3A5*3 and bilirubin predict midazolam population pharmacokinetics in Asian cancer patients. Journal Of Clinical Pharmacology. 2014; **54**(2): 215-24.
2902. Seo MK, Ly NN, Lee CH, Cho HY, Choi CM, Nhu LH, et al. Early life stress increases stress vulnerability through BDNF gene epigenetic changes in the rat hippocampus. Neuropharmacology. 2016; **105**: 388-97.
2903. Seretti A, Cusin C, Lattuada E, Di Bella D, Catalano M, Smeraldi E. Serotonin transporter gene (5-HTTLPR) is not associated with depressive symptomatology in mood disorders. Molecular Psychiatry. 1999; **4**(3): 280-3.
2904. Serretti A, Liappas I, Mandelli L, Albani D, Forloni G, Malitas P, et al. TPH2 gene variants and anxiety during alcohol detoxification outcome. Psychiatry Research. 2009; **167**(1-2): 106-14.
2905. Serretti A, Mandelli L, Lorenzi C, Pirovano A, Olgiati P, Colombo C, et al. Serotonin transporter gene influences the time course of improvement of "core" depressive and somatic anxiety symptoms during treatment with SSRIs for recurrent mood disorders. Psychiatry Research. 2007; **149**(1-3): 185-93.
2906. Shahin MH, Sá AC, Webb A, Gong Y, Langaee T, McDonough CW, et al.

Genome-Wide Prioritization and Transcriptomics Reveal Novel Signatures Associated With Thiazide Diuretics Blood Pressure Response. Circulation Cardiovascular Genetics. 2017; **10**(1).
2907. Shalev I, Moffitt TE, Braithwaite AW, Danese A, Fleming NI, Goldman-Mellor S, et al. Internalizing disorders and leukocyte telomere erosion: a prospective study of depression, generalized anxiety disorder and post-traumatic stress disorder. Molecular Psychiatry. 2014; **19**(11): 1163-70.
2908. Sharan K, Mishra JS, Swarnkar G, Siddiqui JA, Khan K, Kumari R, et al. A novel quercetin analogue from a medicinal plant promotes peak bone mass achievement and bone healing after injury and exerts an anabolic effect on osteoporotic bone: the role of aryl hydrocarbon receptor as a mediator of osteogenic action. Journal Of Bone And Mineral Research: The Official Journal Of The American Society For Bone And Mineral Research. 2011; **26**(9): 2096-111.
2909. Sharma GB, Robertson DD, Laney DA, Gambello MJ, Terk M. Machine learning based analytics of micro-MRI trabecular bone microarchitecture and texture in type 1 Gaucher disease. Journal Of Biomechanics. 2016; **49**(9): 1961-8.
2910. Sharpley CF, Christie DRH, Bitsika V, Andronicos NM, Agnew LL, McMillan ME. Does psychological resilience buffer against the link between the 5-HTTLPR polymorphism and depression following stress. Physiology & Behavior. 2017; **180**: 53-9.
2911. Shen J, Xu L, Qu C, Sun H, Zhang J. Resveratrol prevents cognitive deficits induced by chronic unpredictable mild stress: Sirt1/miR-134 signalling pathway regulates CREB/BDNF expression in hippocampus in vivo and in vitro. Behavioural Brain Research. 2018; **349**: 1-7.
2912. Shi H, Yu X, Li Q, Ye X, Gao Y, Ma J, et al. Association between PPAR-γ and RXR-α gene polymorphism and metabolic syndrome risk: a case-control study of a Chinese Han population. Archives Of Medical Research. 2012; **43**(3): 233-42.
2913. Shi Y, Ullrich SJ, Zhang J, Connolly K, Grzegorzewski KJ, Barber MC, et al. A novel cytokine receptor-ligand pair. Identification, molecular characterization, and in vivo immunomodulatory activity. The Journal Of Biological Chemistry. 2000; **275**(25): 19167-76.
2914. Shichi D, Matsumori A, Naruse TK, Inoko H, Kimura A. HLA-DPbeta chain may confer the susceptibility to hepatitis C virus-associated hypertrophic cardiomyopathy. International Journal Of Immunogenetics. 2008; **35**(1): 37-43.
2915. Shickh S, Clausen M, Mighton C, Casalino S, Joshi E, Glogowski E, et al. Evaluation of a decision aid for incidental genomic results, the Genomics ADvISER: protocol for a mixed methods randomised controlled trial. BMJ Open. 2018; **8**(4): e021876-e.
2916. Shin D, Lee KW, Kim M-H, Kim HJ, An YS, Chung H-K. Identifying Dietary Patterns Associated with Mild Cognitive Impairment in Older Korean Adults Using Reduced Rank Regression. International Journal Of Environmental Research And Public Health. 2018; **15**(1).
2917. Shinomoto M, Kawasaki T, Sugahara T, Nakata K, Kotani T, Yoshitake H, et al. First report of human parechovirus type 3 infection in a pregnant woman. International Journal Of Infectious Diseases: IJID: Official Publication Of The International Society For Infectious Diseases. 2017; **59**: 22-4.

2918. Shishkina GT, Lanshakov DA, Bannova AV, Kalinina TS, Agarina NP, Dygalo NN. Doxycycline Used for Control of Transgene Expression has its Own Effects on Behaviors and Bcl-xL in the Rat Hippocampus. Cell Mol Neurobiol. 2018; **38**(1): 281-8.
2919. Shu X, Lin J, Wood CG, Tannir NM, Wu X. Energy balance, polymorphisms in the mTOR pathway, and renal cell carcinoma risk. Journal Of The National Cancer Institute. 2013; **105**(6): 424-32.
2920. Sibille E, Su J, Leman S, Le Guisquet AM, Ibarguen-Vargas Y, Joeyen-Waldorf J, et al. Lack of serotonin1B receptor expression leads to age-related motor dysfunction, early onset of brain molecular aging and reduced longevity. Molecular Psychiatry. 2007; **12**(11): 1042.
2921. Silva BAE, Cassilhas RC, Attux C, Cordeiro Q, Gadelha AL, Telles BA, et al. A 20-week program of resistance or concurrent exercise improves symptoms of schizophrenia: results of a blind, randomized controlled trial. Revista Brasileira De Psiquiatria (Sao Paulo, Brazil: 1999). 2015; **37**(4): 271-9.
2922. Silva BM, Neves FJ, Negrão MV, Alves CR, Dias RG, Alves GB, et al. Endothelial nitric oxide synthase polymorphisms and adaptation of parasympathetic modulation to exercise training. Medicine And Science In Sports And Exercise. 2011; **43**(9): 1611-8.
2923. Silveira PP, Portella AK, Kennedy JL, Gaudreau H, Davis C, Steiner M, et al. Association between the seven-repeat allele of the dopamine-4 receptor gene (DRD4) and spontaneous food intake in pre-school children. Appetite. 2014; **73**: 15-22.
2924. Simsek M, Cetin Z, Bilgen T, Taskin O, Luleci G, Keser I. Effects of hormone replacement therapy on bone mineral density in Turkish patients with or without COL1A1 Sp1 binding site polymorphism. The Journal Of Obstetrics And Gynaecology Research. 2008; **34**(1): 73-7.
2925. Sinder BP, Lloyd WR, Salemi JD, Marini JC, Caird MS, Morris MD, et al. Effect of anti-sclerostin therapy and osteogenesis imperfecta on tissue-level properties in growing and adult mice while controlling for tissue age. Bone. 2016; **84**: 222-9.
2926. Sinder BP, Salemi JD, Ominsky MS, Caird MS, Marini JC, Kozloff KM. Rapidly growing Brtl/+ mouse model of osteogenesis imperfecta improves bone mass and strength with sclerostin antibody treatment. Bone. 2015; **71**: 115-23.
2927. Sindwani S, Singal DP. Polymorphism in the Y box controls level of cytokine-mediated expression of HLA-DRB1 genes. Tissue Antigens. 2001; **58**(5): 315-23.
2928. Sjöholm LK, Melas PA, Forsell Y, Lavebratt C. PreproNPY Pro7 protects against depression despite exposure to environmental risk factors. Journal Of Affective Disorders. 2009; **118**(1-3): 124-30.
2929. Smith JE, Lawrence AD, Diukova A, Wise RG, Rogers PJ. Storm in a coffee cup: caffeine modifies brain activation to social signals of threat. Social Cognitive And Affective Neuroscience. 2012; **7**(7): 831-40.
2930. Søberg S, Sandholt CH, Jespersen NZ, Toft U, Madsen AL, von Holstein-Rathlou S, et al. FGF21 Is a Sugar-Induced Hormone Associated with Sweet Intake and Preference in Humans. Cell Metabolism. 2017; **25**(5): 1045-53.e6.
2931. Söderqvist S, Matsson H, Peyrard-Janvid M, Kere J, Klingberg T. Polymorphisms in the dopamine receptor 2 gene region influence improvements during working memory training in children and adolescents. Journal Of Cognitive Neuroscience. 2014; **26**(1): 54-62.

2932. Soliman F, Glatt CE, Bath KG, Levita L, Jones RM, Pattwell SS, et al. A genetic variant BDNF polymorphism alters extinction learning in both mouse and human. Science (New York, NY). 2010; **327**(5967): 863-6.
2933. Song P, Zhu H, Zhang D, Chu H, Wu D, Kang M, et al. A genetic variant of miR-148a binding site in the SCRN1 3'-UTR is associated with susceptibility and prognosis of gastric cancer. Scientific Reports. 2014; **4**: 7080-.
2934. Song X, Liu B, Cui L, Zhou B, Liu W, Xu F, et al. Silibinin ameliorates anxiety/depression-like behaviors in amyloid beta-treated rats by upregulating BDNF/TrkB pathway and attenuating autophagy in hippocampus. Physiol Behav. 2017; **179**: 487-93.
2935. Songpatanasilp T, Chailurkit L-O, Nichachotsalid A, Chantarasorn M. Combination of alfacalcidol with calcium can improve quadriceps muscle strength in elderly ambulatory Thai women who have hypovitaminosis D: a randomized controlled trial. Journal Of The Medical Association Of Thailand = Chotmaihet Thangphaet. 2009; **92 Suppl5**: S30-S41.
2936. Songserm N, Promthet S, Pientong C, Ekalaksananan T, Chopjitt P, Wiangnon S. Gene-environment interaction involved in cholangiocarcinoma in the Thai population: polymorphisms of DNA repair genes, smoking and use of alcohol. BMJ Open. 2014; **4**(10): e005447-e.
2937. Sonna LA, Sharp MA, Knapik JJ, Cullivan M, Angel KC, Patton JF, et al. Angiotensin-converting enzyme genotype and physical performance during US Army basic training. Journal Of Applied Physiology (Bethesda, Md: 1985). 2001; **91**(3): 1355-63.
2938. Soto M, Herzog C, Pacheco JA, Fujisaka S, Bullock K, Clish CB, et al. Gut microbiota modulate neurobehavior through changes in brain insulin sensitivity and metabolism. Molecular Psychiatry. 2018.
2939. Soyka M, Preuss UW, Koller G, Zill P, Bondy B. Association of 5-HT1B receptor gene and antisocial behavior in alcoholism. Journal Of Neural Transmission (Vienna, Austria: 1996). 2004; **111**(1): 101-9.
2940. Sparks S, Rakocevic G, Joe G, Manoli I, Shrader J, Harris-Love M, et al. Intravenous immune globulin in hereditary inclusion body myopathy: a pilot study. BMC Neurology. 2007; **7**: 3-.
2941. Speakman JR. The 'Fat Mass and Obesity Related' (FTO) gene: Mechanisms of Impact on Obesity and Energy Balance. Current Obesity Reports. 2015; **4**(1): 73-91.
2942. Speakman JR, Rance KA, Johnstone AM. Polymorphisms of the FTO gene are associated with variation in energy intake, but not energy expenditure. Obesity (Silver Spring, Md). 2008; **16**(8): 1961-5.
2943. Sponton CHG, Rezende TM, Mallagrino PA, Franco-Penteado CF, Bezerra MAC, Zanesco A. Women with TT genotype for eNOS gene are more responsive in lowering blood pressure in response to exercise. European Journal Of Cardiovascular Prevention And Rehabilitation: Official Journal Of The European Society Of Cardiology, Working Groups On Epidemiology & Prevention And Cardiac Rehabilitation And Exercise Physiology. 2010; **17**(6): 676-81.
2944. Steiger H, Joober R, Gauvin L, Bruce KR, Richardson J, Israel M, et al. Serotonin-system polymorphisms (5-HTTLPR and -1438G/A) and responses of patients with bulimic syndromes to multimodal treatments. The Journal Of Clinical Psychiatry.

2008; **69**(10): 1565-71.
2945. Stein MB, Keshaviah A, Haddad SA, Van Ameringen M, Simon NM, Pollack MH, et al. Influence of RGS2 on sertraline treatment for social anxiety disorder. Neuropsychopharmacology: Official Publication Of The American College Of Neuropsychopharmacology. 2014; **39**(6): 1340-6.
2946. Stepanow S, Reichwald K, Huse K, Gausmann U, Nebel A, Rosenstiel P, et al. Allele-specific, age-dependent and BMI-associated DNA methylation of human MCHR1. PLoS One. 2011; **6**(5): e17711-e.
2947. Stift M, Hunter BD, Shaw B, Adam A, Hoebe PN, Mable BK. Inbreeding depression in self-incompatible North-American Arabidopsis lyrata: disentangling genomic and S-locus-specific genetic load. Heredity (Edinb). 2013; **110**(1): 19-28.
2948. Stocks T, Angquist L, Banasik K, Harder MN, Taylor MA, Hager J, et al. TFAP2B influences the effect of dietary fat on weight loss under energy restriction. PLoS One. 2012; **7**(8): e43212-e.
2949. Streetman DS, Bleakley JF, Kim JS, Nafziger AN, Leeder JS, Gaedigk A, et al. Combined phenotypic assessment of CYP1A2, CYP2C19, CYP2D6, CYP3A, N-acetyltransferase-2, and xanthine oxidase with the "Cooperstown cocktail". Clinical Pharmacology And Therapeutics. 2000; **68**(4): 375-83.
2950. Strickland JC, Abel JM, Lacy RT, Beckmann JS, Witte MA, Lynch WJ, et al. The effects of resistance exercise on cocaine self-administration, muscle hypertrophy, and BDNF expression in the nucleus accumbens. Drug And Alcohol Dependence. 2016; **163**: 186-94.
2951. Strimpakos G, Corbi N, Pisani C, Di Certo MG, Onori A, Luvisetto S, et al. Novel adeno-associated viral vector delivering the utrophin gene regulator jazz counteracts dystrophic pathology in mdx mice. Journal Of Cellular Physiology. 2014; **229**(9): 1283-91.
2952. Strobush L, Berg R, Cross D, Foth W, Kitchner T, Coleman L, et al. Dietary intake in the Personalized Medicine Research Project: a resource for studies of gene-diet interaction. Nutrition Journal. 2011; **10**: 13-.
2953. Strul H, Barenboim E, Leshno M, Gartner M, Kariv R, Aljadeff E, et al. The I1307K adenomatous polyposis coli gene variant does not contribute in the assessment of the risk for colorectal cancer in Ashkenazi Jews. Cancer Epidemiology, Biomarkers & Prevention: A Publication Of The American Association For Cancer Research, Cosponsored By The American Society Of Preventive Oncology. 2003; **12**(10): 1012-5.
2954. Stumvoll M, Fritsche A, t'Hart LM, Machann J, Thamer C, Tschritter O, et al. The Gly482Ser variant in the peroxisome proliferator-activated receptor gamma coactivator-1 is not associated with diabetes-related traits in non-diabetic German and Dutch populations. Experimental And Clinical Endocrinology & Diabetes: Official Journal, German Society Of Endocrinology [And] German Diabetes Association. 2004; **112**(5): 253-7.
2955. Su J, Ekman C, Oskolkov N, Lahti L, Ström K, Brazma A, et al. A novel atlas of gene expression in human skeletal muscle reveals molecular changes associated with aging. Skeletal Muscle. 2015; **5**: 35-.
2956. Su SC, Hsieh MJ, Lin CW, Chuang CY, Liu YF, Yeh CM, et al. Impact of HOTAIR Gene Polymorphism and Environmental Risk on Oral Cancer. Journal Of Dental

Research. 2018; **97**(6): 717-24.
2957. Suchanek P, Lorenzova A, Poledne R, Hubacek JA. Changes of plasma lipids during weight reduction in females depends on APOA5 variants. Annals Of Nutrition & Metabolism. 2008; **53**(2): 104-8.
2958. Suda S, Segi-Nishida E, Newton SS, Duman RS. A postpartum model in rat: behavioral and gene expression changes induced by ovarian steroid deprivation. Biological Psychiatry. 2008; **64**(4): 311-9.
2959. Sulakhiya K, Kumar P, Gurjar SS, Barua CC, Hazarika NK. Beneficial effect of honokiol on lipopolysaccharide induced anxiety-like behavior and liver damage in mice. Pharmacol Biochem Behav. 2015; **132**: 79-87.
2960. Suri P, Boyko EJ, Smith NL, Jarvik JG, Williams FMK, Jarvik GP, et al. Modifiable risk factors for chronic back pain: insights using the co-twin control design. The Spine Journal: Official Journal Of The North American Spine Society. 2017; **17**(1): 4-14.
2961. Suwazono Y, Kobayashi E, Uetani M, Miura K, Morikawa Y, Ishizaki M, et al. G-protein beta 3 subunit polymorphism C1429T and low-density lipoprotein receptor-related protein 5 polymorphism A1330V are risk factors for hypercholesterolemia in Japanese males--a prospective study over 5 years. Metabolism: Clinical And Experimental. 2006; **55**(6): 751-7.
2962. Suzuki A, Kondo T, Mihara K, Yasui-Furukori N, Otani K, Furukori H, et al. Association between TaqI A dopamine D2 receptor polymorphism and therapeutic response to bromperidol: a preliminary report. European Archives Of Psychiatry And Clinical Neuroscience. 2001; **251**(2): 57-9.
2963. Suzuki K, Takahashi M, Li C-Y, Lin S-P, Tomari M, Shing CM, et al. The acute effects of green tea and carbohydrate coingestion on systemic inflammation and oxidative stress during sprint cycling. Applied Physiology, Nutrition, And Metabolism = Physiologie Appliquee, Nutrition Et Metabolisme. 2015; **40**(10): 997-1003.
2964. Suzuki N, Mori-Yoshimura M, Yamashita S, Nakano S, Murata K-Y, Inamori Y, et al. Multicenter questionnaire survey for sporadic inclusion body myositis in Japan. Orphanet Journal Of Rare Diseases. 2016; **11**(1): 146-.
2965. Swart KMA, Enneman AW, van Wijngaarden JP, van Dijk SC, Brouwer-Brolsma EM, Ham AC, et al. Homocysteine and the methylenetetrahydrofolate reductase 677C-->T polymorphism in relation to muscle mass and strength, physical performance and postural sway. European Journal Of Clinical Nutrition. 2013; **67**(7): 743-8.
2966. Swindell WR, Johnston A, Carbajal S, Han G, Wohn C, Lu J, et al. Genome-wide expression profiling of five mouse models identifies similarities and differences with human psoriasis. PLoS One. 2011; **6**(4): e18266-e.
2967. Szendrei B, González-Lamuño D, Amigo T, Wang G, Pitsiladis Y, Benito PJ, et al. Influence of ADRB2 Gln27Glu and ADRB3 Trp64Arg polymorphisms on body weight and body composition changes after a controlled weight-loss intervention. Applied Physiology, Nutrition, And Metabolism = Physiologie Appliquee, Nutrition Et Metabolisme. 2016; **41**(3): 307-14.
2968. Takamichi M, Nishimiya Y, Miura A, Tsuda S. Effect of annealing time of an ice crystal on the activity of type III antifreeze protein. The FEBS Journal. 2007; **274**(24): 6469-76.
2969. Takekita Y, Fabbri C, Kato M, Nonen S, Sakai S, Sunada N, et al. HTR1A Gene

Polymorphisms and 5-HT1A Receptor Partial Agonist Antipsychotics Efficacy in Schizophrenia. Journal Of Clinical Psychopharmacology. 2015; **35**(3): 220-7.
2970. Tamaji A, Iwamoto K, Kawamura Y, Takahashi M, Ebe K, Kawano N, et al. Differential effects of diazepam, tandospirone, and paroxetine on plasma brain-derived neurotrophic factor level under mental stress. Human Psychopharmacology. 2012; **27**(3): 329-33.
2971. Tambs K, Harris JR, Magnus P. Genetic and environmental contributions to the correlation between alcohol consumption and symptoms of anxiety and depression. Results from a bivariate analysis of Norwegian twin data. Behavior Genetics. 1997; **27**(3): 241-50.
2972. Tamman AJF, Sippel LM, Han S, Neria Y, Krystal JH, Southwick SM, et al. Attachment style moderates effects of FKBP5 polymorphisms and childhood abuse on post-traumatic stress symptoms: Results from the National Health and Resilience in Veterans Study. The World Journal Of Biological Psychiatry: The Official Journal Of The World Federation Of Societies Of Biological Psychiatry. 2017: 1-12.
2973. Tan L, Yang H, Pang W, Li H, Liu W, Sun S, et al. Investigation on the Role of BDNF in the Benefits of Blueberry Extracts for the Improvement of Learning and Memory in Alzheimer's Disease Mouse Model. Journal Of Alzheimer's Disease: JAD. 2017; **56**(2): 629-40.
2974. Tan WC, Kuppusamy UR, Phan CW, Sabaratnam V. Cell Proliferation and DNA Repair Ability of Ganoderma neo-japonicum (Agaricomycetes): An Indigenous Medicinal Mushroom from Malaysia. International Journal Of Medicinal Mushrooms. 2018; **20**(2): 155-63.
2975. Tanaka M, Kobayashi D, Murakami Y, Ozaki N, Suzuki T, Iwata N, et al. Genetic polymorphisms in the 5-hydroxytryptamine type 3B receptor gene and paroxetine-induced nausea. The International Journal Of Neuropsychopharmacology. 2008; **11**(2): 261-7.
2976. Tang BK, Kadar D, Qian L, Iriah J, Yip J, Kalow W. Caffeine as a metabolic probe: validation of its use for acetylator phenotyping. Clinical Pharmacology And Therapeutics. 1991; **49**(6): 648-57.
2977. Tanisawa K, Ito T, Sun X, Cao Z-B, Sakamoto S, Tanaka M, et al. Polygenic risk for hypertriglyceridemia is attenuated in Japanese men with high fitness levels. Physiological Genomics. 2014; **46**(6): 207-15.
2978. Taylor AMW, Castonguay A, Taylor AJ, Murphy NP, Ghogha A, Cook C, et al. Microglia disrupt mesolimbic reward circuitry in chronic pain. The Journal Of Neuroscience: The Official Journal Of The Society For Neuroscience. 2015; **35**(22): 8442-50.
2979. Taylor JY, Wu CY. Effects of genetic counseling for hypertension on changes in lifestyle behaviors among African-American women. Journal Of National Black Nurses' Association: JNBNA. 2009; **20**(1): 1-10.
2980. Taylor-Tolbert NS, Dengel DR, Brown MD, McCole SD, Pratley RE, Ferrell RE, et al. Ambulatory blood pressure after acute exercise in older men with essential hypertension. American Journal Of Hypertension. 2000; **13**(1 Pt 1): 44-51.
2981. Teicher MH, Samson JA. Childhood maltreatment and psychopathology: A case for ecophenotypic variants as clinically and neurobiologically distinct subtypes. The

American Journal Of Psychiatry. 2013; **170**(10): 1114-33.
2982. Teske JA, Perez-Leighton CE, Noble EE, Wang C, Billington CJ, Kotz CM. Effect of Housing Types on Growth, Feeding, Physical Activity, and Anxiety-Like Behavior in Male Sprague-Dawley Rats. Front Nutr. 2016; **3**: 4.
2983. Tessman ES, Peterson P. Plaque color method for rapid isolation of novel recA mutants of Escherichia coli K-12: new classes of protease-constitutive recA mutants. Journal Of Bacteriology. 1985; **163**(2): 677-87.
2984. Thomaes T, Thomis M, Onkelinx S, Fagard R, Matthijs G, Buys R, et al. A genetic predisposition score for muscular endophenotypes predicts the increase in aerobic power after training: the CAREGENE study. BMC Genetics. 2011; **12**: 84-.
2985. Thomas S, Yingling L, Adu-Brimpong J, Mitchell V, Ayers CR, Wallen GR, et al. Mobile Health Technology Can Objectively Capture Physical Activity (PA) Targets Among African-American Women Within Resource-Limited Communities-the Washington, D.C. Cardiovascular Health and Needs Assessment. Journal Of Racial And Ethnic Health Disparities. 2016.
2986. Thomazeau J, Rouquette A, Martinez V, Rabuel C, Prince N, Laplanche JL, et al. Acute pain Factors predictive of post-operative pain and opioid requirement in multimodal analgesia following knee replacement. European Journal Of Pain (London, England). 2016; **20**(5): 822-32.
2987. Thompson PD, Tsongalis GJ, Seip RL, Bilbie C, Miles M, Zoeller R, et al. Apolipoprotein E genotype and changes in serum lipids and maximal oxygen uptake with exercise training. Metabolism: Clinical And Experimental. 2004; **53**(2): 193-202.
2988. Tiemeier H, Verhulst FC. [Violent behavior in men due to genetic predisposition and childhood abuse: an hypothesis]. Nederlands Tijdschrift Voor Geneeskunde. 2003; **147**(18): 844-6.
2989. Tiemersma EW, Wark PA, Ocké MC, Bunschoten A, Otten MH, Kok FJ, et al. Alcohol consumption, alcohol dehydrogenase 3 polymorphism, and colorectal adenomas. Cancer Epidemiology, Biomarkers & Prevention: A Publication Of The American Association For Cancer Research, Cosponsored By The American Society Of Preventive Oncology. 2003; **12**(5): 419-25.
2990. Toennesen LL, Soerensen ED, Hostrup M, Porsbjerg C, Bangsbo J, Backer V. Feasibility of high-intensity training in asthma. European Clinical Respiratory Journal. 2018; **5**(1): 1468714-.
2991. Tonoli C, Heyman E, Buyse L, Roelands B, Piacentini MF, Bailey S, et al. Neurotrophins and cognitive functions in T1D compared with healthy controls: effects of a high-intensity exercise. Applied Physiology, Nutrition, And Metabolism = Physiologie Appliquee, Nutrition Et Metabolisme. 2015; **40**(1): 20-7.
2992. Tonoli C, Heyman E, Roelands B, Buyse L, Piacentini F, Berthoin S, et al. BDNF, IGF-I, Glucose and Insulin during Continuous and Interval Exercise in Type 1 Diabetes. International Journal Of Sports Medicine. 2015; **36**(12): 955-9.
2993. Torabi-Nami M, Nasehi M, Zarrindast MR. [Not Available]. EXCLI J. 2013; **12**: 347-72.
2994. Torres-Lista V, López-Pousa S, Giménez-Llort L. Marble-burying is enhanced in 3xTg-AD mice, can be reversed by risperidone and it is modulable by handling. Behavioural Processes. 2015; **116**: 69-74.

2995. Tõru I, Maron E, Raag M, Vasar V, Nutt DJ, Shlik J. The effect of 6-week treatment with escitalopram on CCK-4 challenge: a placebo-controlled study in CCK-4-sensitive healthy volunteers. European Neuropsychopharmacology: The Journal Of The European College Of Neuropsychopharmacology. 2013; **23**(7): 645-52.
2996. Touma C, Gassen NC, Herrmann L, Cheung-Flynn J, Büll DR, Ionescu IA, et al. FK506 binding protein 5 shapes stress responsiveness: modulation of neuroendocrine reactivity and coping behavior. Biological Psychiatry. 2011; **70**(10): 928-36.
2997. Touvier M, Deschasaux M, Montourcy M, Sutton A, Charnaux N, Kesse-Guyot E, et al. Determinants of vitamin D status in Caucasian adults: influence of sun exposure, dietary intake, sociodemographic, lifestyle, anthropometric, and genetic factors. The Journal Of Investigative Dermatology. 2015; **135**(2): 378-88.
2998. Tremblay A, Poehlman ET, Despres JP, Theriault G, Danforth E, Bouchard C. Endurance training with constant energy intake in identical twins: changes over time in energy expenditure and related hormones. Metabolism: Clinical And Experimental. 1997; **46**(5): 499-503.
2999. Tremblay A, Poehlman ET, Nadeau A, Dussault J, Bouchard C. Heredity and overfeeding-induced changes in submaximal exercise VO2. Journal Of Applied Physiology (Bethesda, Md: 1985). 1987; **62**(2): 539-44.
3000. Tsai C-L, Pan C-Y, Chen F-C, Wang C-H, Chou F-Y. Effects of acute aerobic exercise on a task-switching protocol and brain-derived neurotrophic factor concentrations in young adults with different levels of cardiorespiratory fitness. Experimental Physiology. 2016; **101**(7): 836-50.
3001. Tsai VW-W, Macia L, Johnen H, Kuffner T, Manadhar R, Jørgensen SB, et al. TGF-b superfamily cytokine MIC-1/GDF15 is a physiological appetite and body weight regulator. PLoS One. 2013; **8**(2): e55174-e.
3002. Tung YCL, Rimmington D, O'Rahilly S, Coll AP. Pro-opiomelanocortin modulates the thermogenic and physical activity responses to high-fat feeding and markedly influences dietary fat preference. Endocrinology. 2007; **148**(11): 5331-8.
3003. Tupinambás U, Aleixo A, Greco D. HIV-1 genotypes related to failure of nelfinavir as the first protease inhibitor treatment. The Brazilian Journal Of Infectious Diseases: An Official Publication Of The Brazilian Society Of Infectious Diseases. 2005; **9**(4): 324-9.
3004. Tural E, Kara N, Agaoglu SA, Elbistan M, Tasmektepligil MY, Imamoglu O. PPAR-alpha and PPARGC1A gene variants have strong effects on aerobic performance of Turkish elite endurance athletes. Mol Biol Rep. 2014; **41**(9): 5799-804.
3005. Tural E, Kara N, Agaoglu SA, Elbistan M, Tasmektepligil MY, Imamoglu O. PPAR-α and PPARGC1A gene variants have strong effects on aerobic performance of Turkish elite endurance athletes. Molecular Biology Reports. 2014; **41**(9): 5799-804.
3006. Turner BJ, Murray SS, Piccenna LG, Lopes EC, Kilpatrick TJ, Cheema SS. Effect of p75 neurotrophin receptor antagonist on disease progression in transgenic amyotrophic lateral sclerosis mice. Journal Of Neuroscience Research. 2004; **78**(2): 193-9.
3007. Turpault S, Brian W, Van Horn R, Santoni A, Poitiers F, Donazzolo Y, et al. Pharmacokinetic assessment of a five-probe cocktail for CYPs 1A2, 2C9, 2C19, 2D6 and 3A. British Journal Of Clinical Pharmacology. 2009; **68**(6): 928-35.

3008. Umene W, Yoshimura R, Hori H, Nakano H, Sugita A, Shimono M, et al. Blood levels of catecholamine metabolites and brain-derived neurotrophic factor in a case of Sydenham's chorea. The World Journal Of Biological Psychiatry: The Official Journal Of The World Federation Of Societies Of Biological Psychiatry. 2009; **10**(3): 248-51.
3009. Unschuld PG, Ising M, Roeske D, Erhardt A, Specht M, Kloiber S, et al. Gender-specific association of galanin polymorphisms with HPA-axis dysregulation, symptom severity, and antidepressant treatment response. Neuropsychopharmacology: Official Publication Of The American College Of Neuropsychopharmacology. 2010; **35**(7): 1583-92.
3010. Uomori T, Horimoto Y, Mogushi K, Matsuoka J, Saito M. Relationship between alcohol metabolism and chemotherapy-induced emetic events in breast cancer patients. Breast Cancer (Tokyo, Japan). 2017; **24**(5): 702-7.
3011. Urabe M, Ding C, Kotin RM. Insect cells as a factory to produce adeno-associated virus type 2 vectors. Human Gene Therapy. 2002; **13**(16): 1935-43.
3012. Uveges TE, Kozloff KM, Ty JM, Ledgard F, Raggio CL, Gronowicz G, et al. Alendronate treatment of the brtl osteogenesis imperfecta mouse improves femoral geometry and load response before fracture but decreases predicted material properties and has detrimental effects on osteoblasts and bone formation. Journal Of Bone And Mineral Research: The Official Journal Of The American Society For Bone And Mineral Research. 2009; **24**(5): 849-59.
3013. Väisänen S, Rauramaa R, Rankinen T, Gagnon J, Couchard C. Physical activity, fitness, and plasma fibrinogen with reference to fibrinogen genotypes. Medicine And Science In Sports And Exercise. 1996; **28**(9): 1165-70.
3014. Välimäki V-V, Alfthan H, Lehmuskallio E, Löyttyniemi E, Sahi T, Suominen H, et al. Risk factors for clinical stress fractures in male military recruits: a prospective cohort study. Bone. 2005; **37**(2): 267-73.
3015. van Dalfsen JH, Markus CR. Interaction between 5-HTTLPR genotype and cognitive stress vulnerability on sleep quality: effects of sub-chronic tryptophan administration. The International Journal Of Neuropsychopharmacology. 2015; **18**(3).
3016. Van den Hout JMP, Kamphoven JHJ, Winkel LPF, Arts WFM, De Klerk JBC, Loonen MCB, et al. Long-term intravenous treatment of Pompe disease with recombinant human alpha-glucosidase from milk. Pediatrics. 2004; **113**(5): e448-e57.
3017. van den Oord EJCG, Kuo P-H, Hartmann AM, Webb BT, Möller H-J, Hettema JM, et al. Genomewide association analysis followed by a replication study implicates a novel candidate gene for neuroticism. Archives Of General Psychiatry. 2008; **65**(9): 1062-71.
3018. van der Voorn B, Wit JM, van der Pal SM, Rotteveel J, Finken MJJ. Antenatal glucocorticoid treatment and polymorphisms of the glucocorticoid and mineralocorticoid receptors are associated with IQ and behavior in young adults born very preterm. The Journal Of Clinical Endocrinology And Metabolism. 2015; **100**(2): 500-7.
3019. van Dijk T, Hwang S, Krug J, de Visser JAGM, Zwart MP. Mutation supply and the repeatability of selection for antibiotic resistance. Physical Biology. 2017; **14**(5): 055005-.
3020. van Ginkel S, Amami M, Dela F, Niederseer D, Narici MV, Niebauer J, et al. Adjustments of muscle capillarity but not mitochondrial protein with skiing in the elderly.

Scandinavian Journal Of Medicine & Science In Sports. 2015; **25**(4): e360-e7.
3021. Van Kempen TA, Gorecka J, Gonzalez AD, Soeda F, Milner TA, Waters EM. Characterization of neural estrogen signaling and neurotrophic changes in the accelerated ovarian failure mouse model of menopause. Endocrinology. 2014; **155**(9): 3610-23.
3022. van Vulpen JK, Schmidt ME, Velthuis MJ, Wiskemann J, Schneeweiss A, Vermeulen RCH, et al. Effects of physical exercise on markers of inflammation in breast cancer patients during adjuvant chemotherapy. Breast Cancer Research And Treatment. 2018; **168**(2): 421-31.
3023. Varbo A, Benn M, Tybjærg-Hansen A, Grande P, Nordestgaard BG. TRIB1 and GCKR polymorphisms, lipid levels, and risk of ischemic heart disease in the general population. Arteriosclerosis, Thrombosis, And Vascular Biology. 2011; **31**(2): 451-7.
3024. Varley I, Hughes DC, Greeves JP, Stellingwerff T, Ranson C, Fraser WD, et al. The association of novel polymorphisms with stress fracture injury in Elite Athletes: Further insights from the SFEA cohort. Journal Of Science And Medicine In Sport. 2018; **21**(6): 564-8.
3025. Vassos E, Panas M, Kladi A, Vassilopoulos D. Effect of CAG repeat length on psychiatric disorders in Huntington's disease. Journal Of Psychiatric Research. 2008; **42**(7): 544-9.
3026. Vaughan S, Wallis M, Polit D, Steele M, Shum D, Morris N. The effects of multimodal exercise on cognitive and physical functioning and brain-derived neurotrophic factor in older women: a randomised controlled trial. Age And Ageing. 2014; **43**(5): 623-9.
3027. Vera-Llonch M, Martin M, Aggarwal J, Donepudi M, Bayliss M, Goss T, et al. Health-related quality of life in genotype 1 treatment-naive chronic hepatitis C patients receiving telaprevir combination treatment in the ADVANCE study. Aliment Pharmacol Ther. 2013; **38**(2): 124-33.
3028. Vera-Llonch M, Martin M, Aggarwal J, Donepudi M, Bayliss M, Goss T, et al. Health-related quality of life in genotype 1 treatment-naïve chronic hepatitis C patients receiving telaprevir combination treatment in the ADVANCE study. Alimentary Pharmacology & Therapeutics. 2013; **38**(2): 124-33.
3029. Vermeulen BAN, Hogen Esch CE, Yuksel Z, Koning F, Verduijn W, Doxiadis IIN, et al. Phenotypic variance in childhood coeliac disease and the HLA-DQ/DR dose effect. Scandinavian Journal Of Gastroenterology. 2009; **44**(1): 40-5.
3030. Vieira-Potter VJ, Padilla J, Park Y-M, Welly RJ, Scroggins RJ, Britton SL, et al. Female rats selectively bred for high intrinsic aerobic fitness are protected from ovariectomy-associated metabolic dysfunction. American Journal Of Physiology Regulatory, Integrative And Comparative Physiology. 2015; **308**(6): R530-R42.
3031. Vieitez FJ, Ballester A, Vieitez AM. Somatic embryogenesis and plantlet regeneration from cell suspension cultures of Fagus sylvatica L. Plant Cell Reports. 1992; **11**(12): 609-13.
3032. Vigano A, Trutschnigg B, Kilgour RD, Hamel N, Hornby L, Lucar E, et al. Relationship between angiotensin-converting enzyme gene polymorphism and body composition, functional performance, and blood biomarkers in advanced cancer patients. Clinical Cancer Research: An Official Journal Of The American Association For

Cancer Research. 2009; **15**(7): 2442-7.
3033. Viitanen A, Kärkkäinen M, Laitinen K, Lamberg-Allardt C, Kainulainen K, Räsänen L, et al. Common polymorphism of the vitamin D receptor gene is associated with variation of peak bone mass in young finns. Calcified Tissue International. 1996; **59**(4): 231-4.
3034. Villarini M, Dominici L, Piccinini R, Fatigoni C, Ambrogi M, Curti G, et al. Assessment of primary, oxidative and excision repaired DNA damage in hospital personnel handling antineoplastic drugs. Mutagenesis. 2011; **26**(3): 359-69.
3035. Vinberg M, Miskowiak K, Hoejman P, Pedersen M, Kessing LV. The effect of recombinant erythropoietin on plasma brain derived neurotrophic factor levels in patients with affective disorders: a randomised controlled study. PLoS One. 2015; **10**(5): e0127629-e.
3036. Vishram JKK, Hansen TW, Torp-Pedersen C, Madsbad S, Jørgensen T, Fenger M, et al. Relationship Between Two Common Lipoprotein Lipase Variants and the Metabolic Syndrome and Its Individual Components. Metabolic Syndrome And Related Disorders. 2016; **14**(9): 442-8.
3037. Vissing J, Duno M, Schwartz M, Haller RG. Splice mutations preserve myophosphorylase activity that ameliorates the phenotype in McArdle disease. Brain: A Journal Of Neurology. 2009; **132**(Pt 6): 1545-52.
3038. Vitha S, Zhao L, Sack FD. Interaction of root gravitropism and phototropism in Arabidopsis wild-type and starchless mutants. Plant Physiology. 2000; **122**(2): 453-62.
3039. Vlam L, Cats EA, Willemse E, Franssen H, Medic J, Piepers S, et al. Pharmacokinetics of intravenous immunoglobulin in multifocal motor neuropathy. Journal Of Neurology, Neurosurgery, And Psychiatry. 2014; **85**(10): 1145-8.
3040. Vogels N, Posthumus DLA, Mariman ECM, Bouwman F, Kester ADM, Rump P, et al. Determinants of overweight in a cohort of Dutch children. The American Journal Of Clinical Nutrition. 2006; **84**(4): 717-24.
3041. von Richter O, Lahu G, Huennemeyer A, Herzog R, Zech K, Hermann R. Effect of fluvoxamine on the pharmacokinetics of roflumilast and roflumilast N-oxide. Clinical Pharmacokinetics. 2007; **46**(7): 613-22.
3042. Vouk K, Ribič-Pucelj M, Adamski J, Rižner TL. Altered levels of acylcarnitines, phosphatidylcholines, and sphingomyelins in peritoneal fluid from ovarian endometriosis patients. The Journal Of Steroid Biochemistry And Molecular Biology. 2016; **159**: 60-9.
3043. Waller KA, Zhang LX, Elsaid KA, Fleming BC, Warman ML, Jay GD. Role of lubricin and boundary lubrication in the prevention of chondrocyte apoptosis. Proceedings Of The National Academy Of Sciences Of The United States Of America. 2013; **110**(15): 5852-7.
3044. Wang C, Tang J, Song W, Lobashevsky E, Wilson CM, Kaslow RA. HLA and cytokine gene polymorphisms are independently associated with responses to hepatitis B vaccination. Hepatology (Baltimore, Md). 2004; **39**(4): 978-88.
3045. Wang HY, Hu Y, Wang SH, Shan QL, Li YC, Nie J, et al. Association of androgen receptor CAG repeat polymorphism with VO(2)max response to hypoxic training in North China Han men. Int J Androl. 2010; **33**(6): 794-9.
3046. Wang HY, Hu Y, Wang SH, Shan QL, Li YC, Nie J, et al. Association of androgen receptor CAG repeat polymorphism with VO_2max response to hypoxic training in North

China Han men. International Journal Of Andrology. 2010; **33**(6): 794-9.
3047. Wang S, Song J, Shang X, Chawla N, Yang Y, Meng X, et al. Physical activity and sedentary behavior can modulate the effect of the PNPLA3 variant on childhood NAFLD: a case-control study in a Chinese population. BMC Medical Genetics. 2016; **17**(1): 90-.
3048. Wang T, Liu Y, Sima L, Shi L, Wang Z, Ni C, et al. Association between MLH1 -93G>a polymorphism and risk of colorectal cancer. PLoS One. 2012; **7**(11): e50449-e.
3049. Wang Y, Mathews CA, Li Y, Lin Z, Xiao Z. Brain-derived neurotrophic factor (BDNF) plasma levels in drug-naive OCD patients are lower than those in healthy people, but are not lower than those in drug-treated OCD patients. J Affect Disord. 2011; **133**(1-2): 305-10.
3050. Wang Y, Mathews CA, Li Y, Lin Z, Xiao Z. Brain-derived neurotrophic factor (BDNF) plasma levels in drug-naïve OCD patients are lower than those in healthy people, but are not lower than those in drug-treated OCD patients. Journal Of Affective Disorders. 2011; **133**(1-2): 305-10.
3051. Wang Z, Zhang C, Huang J, Yuan C, Hong W, Chen J, et al. MiRNA-206 and BDNF genes interacted in bipolar I disorder. Journal Of Affective Disorders. 2014; **162**: 116-9.
3052. Warrilow AGS, Jackson CJ, Parker JE, Marczylo TH, Kelly DE, Lamb DC, et al. Identification, characterization, and azole-binding properties of Mycobacterium smegmatis CYP164A2, a homolog of ML2088, the sole cytochrome P450 gene of Mycobacterium leprae. Antimicrobial Agents And Chemotherapy. 2009; **53**(3): 1157-64.
3053. Wasilewska A, Zalewski G, Chyczewski L, Zoch-Zwierz W. MDR-1 gene polymorphisms and clinical course of steroid-responsive nephrotic syndrome in children. Pediatric Nephrology (Berlin, Germany). 2007; **22**(1): 44-51.
3054. Weber G, Kartodihardjo W, Klissouras V. Growth and physical training with reference to heredity. Journal Of Applied Physiology. 1976; **40**(2): 211-5.
3055. Weber H, Richter J, Straube B, Lueken U, Domschke K, Schartner C, et al. Allelic variation in CRHR1 predisposes to panic disorder: evidence for biased fear processing. Molecular Psychiatry. 2016; **21**(6): 813-22.
3056. Wedekind D, Herchenhein T, Kirchhainer J, Bandelow B, Falkai P, Engel K, et al. Serotonergic function, substance craving, and psychopathology in detoxified alcohol-addicted males undergoing tryptophan depletion. Journal Of Psychiatric Research. 2010; **44**(16): 1163-9.
3057. Wei J, Hemmings GP. TNXB locus may be a candidate gene predisposing to schizophrenia. American Journal Of Medical Genetics Part B, Neuropsychiatric Genetics: The Official Publication Of The International Society Of Psychiatric Genetics. 2004; **125B**(1): 43-9.
3058. Weintraub A, Singaravelu J, Bhatnagar S. Enduring and sex-specific effects of adolescent social isolation in rats on adult stress reactivity. Brain Research. 2010; **1343**: 83-92.
3059. Weiss E, Regnier E, Oudet P. Restriction enzyme accessibility and RNA polymerase localization on transcriptionally active SV40 minichromosomes isolated late in infection. Virology. 1987; **159**(1): 84-93.
3060. Weiss EP, Brandauer J, Kulaputana O, Ghiu IA, Wohn CR, Phares DA, et al.

FABP2 Ala54Thr genotype is associated with glucoregulatory function and lipid oxidation after a high-fat meal in sedentary nondiabetic men and women. The American Journal Of Clinical Nutrition. 2007; **85**(1): 102-8.
3061. Wens I, Keytsman C, Deckx N, Cools N, Dalgas U, Eijnde BO. Brain derived neurotrophic factor in multiple sclerosis: effect of 24 weeks endurance and resistance training. European Journal Of Neurology. 2016; **23**(6): 1028-35.
3062. Wenzel RR, Mitchell A, Siffert W, Bührmann S, Philipp T, Schäfers RF. The I1-imidazoline agonist moxonidine decreases sympathetic tone under physical and mental stress. British Journal Of Clinical Pharmacology. 2004; **57**(5): 545-51.
3063. Whitfield JB, O'Brien ME, Nightingale BN, Zhu G, Heath AC, Martin NG. ADH genotype does not modify the effects of alcohol on high-density lipoprotein. Alcoholism, Clinical And Experimental Research. 2003; **27**(3): 509-14.
3064. Wilhelm CJ, Fuller BE, Huckans M, Loftis JM. Peripheral immune factors are elevated in women with current or recent alcohol dependence and associated with altered mood and memory. Drug And Alcohol Dependence. 2017; **176**: 71-8.
3065. Williams AD, Anderson MJ, Selig S, Carey MF, Febbraio MA, Hayes A, et al. Differential response to resistance training in CHF according to ACE genotype. International Journal Of Cardiology. 2011; **149**(3): 330-4.
3066. Williams PT. Attenuating effect of vigorous physical activity on the risk for inherited obesity: a study of 47,691 runners. PLoS One. 2012; **7**(2): e31436-e.
3067. Wilson LE, Gravitt P, Tobian AAR, Kigozi G, Serwadda D, Nalugoda F, et al. Male circumcision reduces penile high-risk human papillomavirus viral load in a randomised clinical trial in Rakai, Uganda. Sexually Transmitted Infections. 2013; **89**(3): 262-6.
3068. Wilund KR, Ferrell RE, Phares DA, Goldberg AP, Hagberg JM. Changes in high-density lipoprotein-cholesterol subfractions with exercise training may be dependent on cholesteryl ester transfer protein (CETP) genotype. Metabolism: Clinical And Experimental. 2002; **51**(6): 774-8.
3069. Wirström T, Hilding A, Gu HF, Östenson C-G, Björklund A. Consumption of whole grain reduces risk of deteriorating glucose tolerance, including progression to prediabetes. The American Journal Of Clinical Nutrition. 2013; **97**(1): 179-87.
3070. Wittenbecher C, Mühlenbruch K, Kröger J, Jacobs S, Kuxhaus O, Floegel A, et al. Amino acids, lipid metabolites, and ferritin as potential mediators linking red meat consumption to type 2 diabetes. The American Journal Of Clinical Nutrition. 2015; **101**(6): 1241-50.
3071. Witzig M, Ingelmann CJ, Möhring J, Rodehutscord M. Variability of prececal phosphorus digestibility of triticale and wheat in broiler chickens. Poultry Science. 2018; **97**(3): 910-9.
3072. Wolf RL, Cauley JA, Baker CE, Ferrell RE, Charron M, Caggiula AW, et al. Factors associated with calcium absorption efficiency in pre- and perimenopausal women. The American Journal Of Clinical Nutrition. 2000; **72**(2): 466-71.
3073. Wong SL, Locke C, Staser J, Granneman GR. Lack of multiple dosing effect of sertindole on the pharmacokinetics of alprazolam in healthy volunteers. Psychopharmacology. 1998; **135**(3): 236-41.
3074. Wong WM, Hasemann S, Schwarz M, Zill P, Koller G, Soyka M, et al. Citalopram neuropharmacological challenge in alcohol-dependent patients and controls:

pharmacogenetic, endocrine and psychobehavioral results. Pharmacopsychiatry. 2008; **41**(2): 72-8.
3075. Wong-Kisiel LC, Kuntz NL. Two siblings with limb-girdle muscular dystrophy type 2E responsive to deflazacort. Neuromuscular Disorders: NMD. 2010; **20**(2): 122-4.
3076. Woodhouse LJ, Reisz-Porszasz S, Javanbakht M, Storer TW, Lee M, Zerounian H, et al. Development of models to predict anabolic response to testosterone administration in healthy young men. American Journal Of Physiology Endocrinology And Metabolism. 2003; **284**(5): E1009-E17.
3077. Woods DR, World M, Rayson MP, Williams AG, Jubb M, Jamshidi Y, et al. Endurance enhancement related to the human angiotensin I-converting enzyme I-D polymorphism is not due to differences in the cardiorespiratory response to training. European Journal Of Applied Physiology. 2002; **86**(3): 240-4.
3078. Wright B, Alexander D, Aghahoseini A. Does preoperative depression and/or serotonin transporter gene polymorphism predict outcome after laparoscopic cholecystectomy? BMJ Open. 2016; **6**(9): e007969-e.
3079. Wu GF, Ren S, Tang RY, Xu C, Zhou JQ, Lin SM, et al. Antidepressant effect of taurine in chronic unpredictable mild stress-induced depressive rats. Sci Rep. 2017; **7**(1): 4989.
3080. Wu W-N, McKown LA, Reitz AB. Metabolism of the new anxiolytic agent, a pyrido[1,2-]benzimidazole (PBI) analog (RWJ-53050), in rat and human hepatic S9 fractions, and in dog; identification of cytochrome p450 isoforms mediated in the human microsomal metabolism. European Journal Of Drug Metabolism And Pharmacokinetics. 2006; **31**(4): 277-83.
3081. Würtz P, Cook S, Wang Q, Tiainen M, Tynkkynen T, Kangas AJ, et al. Metabolic profiling of alcohol consumption in 9778 young adults. International Journal Of Epidemiology. 2016; **45**(5): 1493-506.
3082. Xu F, Ding E, Liao SX, Migone F, Dai J, Schneider A, et al. Improved efficacy of gene therapy approaches for Pompe disease using a new, immune-deficient GSD-II mouse model. Gene Therapy. 2004; **11**(21): 1590-8.
3083. Xu K, Hong KA, Zhou Z, Hauger RL, Goldman D, Sinha R. Genetic modulation of plasma NPY stress response is suppressed in substance abuse: association with clinical outcomes. Psychoneuroendocrinology. 2012; **37**(4): 554-64.
3084. Xu X, Ji H, Liu G, Wang Q, Liu H, Shen W, et al. A significant association between BDNF promoter methylation and the risk of drug addiction. Gene. 2016; **584**(1): 54-9.
3085. Xu Y, Cui S-Y, Ma Q, Shi J, Yu Y, Li J-X, et al. trans-Resveratrol Ameliorates Stress-Induced Irritable Bowel Syndrome-Like Behaviors by Regulation of Brain-Gut Axis. Frontiers In Pharmacology. 2018; **9**: 631-.
3086. Xue Y, Nie M, Wang O, Wang CY, Han GY, Shen Q, et al. [Association of α-actinin-3 gene polymorphism and muscle strength of postmenopausal women]. Zhonghua Yi Xue Za Zhi. 2018; **98**(18): 1408-13.
3087. Yamada H, Yoshimura C, Nakajima T, Nagata T. Recovery of low plasma BDNF over the course of treatment among patients with bulimia nervosa. Psychiatry Research. 2012; **198**(3): 448-51.
3088. Yamaguchi H, Hara Y, Ago Y, Takano E, Hasebe S, Nakazawa T, et al.

Environmental enrichment attenuates behavioral abnormalities in valproic acid-exposed autism model mice. Behav Brain Res. 2017; **333**: 67-73.
3089. Yan TC, McQuillin A, Thapar A, Asherson P, Hunt SP, Stanford SC, et al. NK1 (TACR1) receptor gene 'knockout' mouse phenotype predicts genetic association with ADHD. Journal Of Psychopharmacology (Oxford, England). 2010; **24**(1): 27-38.
3090. Yang B, Thyagarajan B, Gross MD, Fedirko V, Goodman M, Bostick RM. No evidence that associations of incident, sporadic colorectal adenoma with its major modifiable risk factors differ by chromosome 8q24 region rs6983267 genotype. Molecular Carcinogenesis. 2014; **53 Suppl 1**: E193-E200.
3091. Yang G, Fu Y, Lu X, Wang M, Dong H, Li Q. The interactive effects of genetic polymorphisms within LFA-1/ICAM-1/GSK-3β pathway and environmental hazards on the development of Graves' opthalmopathy. Experimental Eye Research. 2018; **174**: 161-72.
3092. Yang M, Kang M-J, Choi Y, Kim C-S, Lee S-M, Park C-W, et al. Associations between XPC expression, genotype, and the risk of head and neck cancer. Environmental And Molecular Mutagenesis. 2005; **45**(4): 374-9.
3093. Yang X, Ewald ER, Huo Y, Tamashiro KL, Salvatori R, Sawa A, et al. Glucocorticoid-induced loss of DNA methylation in non-neuronal cells and potential involvement of DNMT1 in epigenetic regulation of Fkbp5. Biochemical And Biophysical Research Communications. 2012; **420**(3): 570-5.
3094. Yao D, Kim J, Yu F, Nielsen PE, Sinner E-K, Knoll W. Surface density dependence of PCR amplicon hybridization on PNA/DNA probe layers. Biophysical Journal. 2005; **88**(4): 2745-51.
3095. Yazbek SN, Spiezio SH, Nadeau JH, Buchner DA. Ancestral paternal genotype controls body weight and food intake for multiple generations. Human Molecular Genetics. 2010; **19**(21): 4134-44.
3096. Yihunie Akalu T, Muchie KF, Alemu Gelaye K. Time to sputum culture conversion and its determinants among Multi-drug resistant Tuberculosis patients at public hospitals of the Amhara Regional State: A multicenter retrospective follow up study. PLoS One. 2018; **13**(6): e0199320-e.
3097. Yilmaz Z, Davis C, Loxton NJ, Kaplan AS, Levitan RD, Carter JC, et al. Association between MC4R rs17782313 polymorphism and overeating behaviors. International Journal Of Obesity (2005). 2015; **39**(1): 114-20.
3098. Yoo J, Kim B-H, Kim S-H, Kim Y, Yim S-V. Genetic polymorphisms to predict gains in maximal O2 uptake and knee peak torque after a high intensity training program in humans. European Journal Of Applied Physiology. 2016; **116**(5): 947-57.
3099. Yoshida T, Shin-ya H, Nakai S, Yorimoto A, Morimoto T, Suyama T, et al. Genomic and non-genomic effects of aldosterone on the individual variation of the sweat Na+ concentration during exercise in trained athletes. European Journal Of Applied Physiology. 2006; **98**(5): 466-71.
3100. Yoshimura R, Kishi T, Hori H, Atake K, Katsuki A, Nakano-Umene W, et al. Serum proBDNF/BDNF and response to fluvoxamine in drug-naïve first-episode major depressive disorder patients. Annals Of General Psychiatry. 2014; **13**: 19-.
3101. Yoshizawa T, Yamakawa-Kobayashi K, Komatsuzaki Y, Arinami T, Oguni E, Mizusawa H, et al. Dose-dependent association of apolipoprotein E allele epsilon 4 with

late-onset, sporadic Alzheimer's disease. Annals Of Neurology. 1994; **36**(4): 656-9.
3102. Younossi ZM, Stepanova M, Jacobson IM, Asselah T, Gane EJ, Lawitz E, et al. Sofosbuvir and velpatasvir with or without voxilaprevir in direct-acting antiviral-naïve chronic hepatitis C: patient-reported outcomes from POLARIS 2 and 3. Alimentary Pharmacology & Therapeutics. 2018; **47**(2): 259-67.
3103. Yu D, Zhou H, Yang Y, Jiang Y, Wang T, Lv L, et al. The bidirectional effects of hypothyroidism and hyperthyroidism on anxiety- and depression-like behaviors in rats. Hormones And Behavior. 2015; **69**: 106-15.
3104. Yu H, Wang D-D, Wang Y, Liu T, Lee FS, Chen Z-Y. Variant brain-derived neurotrophic factor Val66Met polymorphism alters vulnerability to stress and response to antidepressants. The Journal Of Neuroscience: The Official Journal Of The Society For Neuroscience. 2012; **32**(12): 4092-101.
3105. Yu H, Wang Y, Pattwell S, Jing D, Liu T, Zhang Y, et al. Variant BDNF Val66Met polymorphism affects extinction of conditioned aversive memory. The Journal Of Neuroscience: The Official Journal Of The Society For Neuroscience. 2009; **29**(13): 4056-64.
3106. Yvert T, Santiago C, Santana-Sosa E, Verde Z, Gómez-Gallego F, López-Mojares LM, et al. Physical-capacity-related genetic polymorphisms in children with cystic fibrosis. Pediatric Exercise Science. 2015; **27**(1): 102-12.
3107. Zago AS, Park J-Y, Fenty-Stewart N, Kokubun E, Brown MD. Effects of aerobic exercise on the blood pressure, oxidative stress and eNOS gene polymorphism in pre-hypertensive older people. European Journal Of Applied Physiology. 2010; **110**(4): 825-32.
3108. Zanchet RC, Chagas AMA, Melo JS, Watanabe PY, Simões-Barbosa A, Feijo G. Influence of the technique of re-educating thoracic and abdominal muscles on respiratory muscle strength in patients with cystic fibrosis. Jornal Brasileiro De Pneumologia: Publicacao Oficial Da Sociedade Brasileira De Pneumologia E Tisilogia. 2006; **32**(2): 123-9.
3109. Zapata L, Pich O, Serrano L, Kondrashov FA, Ossowski S, Schaefer MH. Negative selection in tumor genome evolution acts on essential cellular functions and the immunopeptidome. Genome Biology. 2018; **19**(1): 67-.
3110. Zembron-Lacny A, Dziubek W, Rynkiewicz M, Morawin B, Woźniewski M. Peripheral brain-derived neurotrophic factor is related to cardiovascular risk factors in active and inactive elderly men. Brazilian Journal Of Medical And Biological Research = Revista Brasileira De Pesquisas Medicas E Biologicas. 2016; **49**(7).
3111. Zhang FF, Santella RM, Wolff M, Kappil MA, Markowitz SB, Morabia A. White blood cell global methylation and IL-6 promoter methylation in association with diet and lifestyle risk factors in a cancer-free population. Epigenetics. 2012; **7**(6): 606-14.
3112. Zhang S-Y, Hu Q, Tang T, Liu C, Li C-C, Yang X-G, et al. Role of CACNA1C gene polymorphisms and protein expressions in the pathogenesis of schizophrenia: a case-control study in a Chinese population. Neurological Sciences: Official Journal Of The Italian Neurological Society And Of The Italian Society Of Clinical Neurophysiology. 2017; **38**(8): 1393-403.
3113. Zhang W, Wu L, Wu X, Ding Y, Li G, Li J, et al. Lodging Resistance of Japonica Rice (Oryza Sativa L.): Morphological and Anatomical Traits due to top-Dressing

Nitrogen Application Rates. Rice (New York, NY). 2016; **9**(1): 31-.
3114. Zhang X, Wang C, Dai H, Lin Y, Zhang J. Association between angiotensin-converting enzyme gene polymorphisms and exercise performance in patients with COPD. Respirology (Carlton, Vic). 2008; **13**(5): 683-8.
3115. Zhao B, Moochhala SM, Tham Sy, Lu J, Chia M, Byrne C, et al. Relationship between angiotensin-converting enzyme ID polymorphism and VO(2max) of Chinese males. Life Sciences. 2003; **73**(20): 2625-30.
3116. Zhao H, Sonada S, Yoshikawa A, Ohinata K, Yoshikawa M. Rubimetide, humanin, and MMK1 exert anxiolytic-like activities via the formyl peptide receptor 2 in mice followed by the successive activation of DP1, A2A, and GABAA receptors. Peptides. 2016; **83**: 16-20.
3117. Zhao K, Li Z, Tian H. Twenty-gene-based prognostic model predicts lung adenocarcinoma survival. Oncotargets And Therapy. 2018; **11**: 3415-24.
3118. Zhao T, Li Y, Wei W, Savage S, Zhou L, Ma D. Ketamine administered to pregnant rats in the second trimester causes long-lasting behavioral disorders in offspring. Neurobiology Of Disease. 2014; **68**: 145-55.
3119. Zhou A, Taylor AE, Karhunen V, Zhan Y, Rovio SP, Lahti J, et al. Habitual coffee consumption and cognitive function: a Mendelian randomization meta-analysis in up to 415,530 participants. Scientific Reports. 2018; **8**(1): 7526-.
3120. Zhu D, Liu G-y, Lv Z, Wen S-r, Bi S, Wang W-z. Inverse associations of outdoor activity and vitamin D intake with the risk of Parkinson's disease. Journal Of Zhejiang University Science B. 2014; **15**(10): 923-7.
3121. Zhu Q, Zhu P, Zhang Y, Li J, Ma X, Li N, et al. Analysis of Social and Genetic Factors Influencing Heterosexual Transmission of HIV within Serodiscordant Couples in the Henan Cohort. PLoS One. 2015; **10**(6): e0129979-e.
3122. Ziv-Gal A, Flaws JA. Factors that may influence the experience of hot flushes by healthy middle-aged women. Journal Of Women's Health (2002). 2010; **19**(10): 1905-14.
3123. Zohar J, Yahalom H, Kozlovsky N, Cwikel-Hamzany S, Matar MA, Kaplan Z, et al. High dose hydrocortisone immediately after trauma may alter the trajectory of PTSD: interplay between clinical and animal studies. European Neuropsychopharmacology: The Journal Of The European College Of Neuropsychopharmacology. 2011; **21**(11): 796-809.
3124. Zubillaga P, Vidales MC, Zubillaga I, Ormaechea V, García-Urkía N, Vitoria JC. HLA-DQA1 and HLA-DQB1 genetic markers and clinical presentation in celiac disease. Journal Of Pediatric Gastroenterology And Nutrition. 2002; **34**(5): 548-54.
3125. Züst T, Agrawal AA. Plant chemical defense indirectly mediates aphid performance via interactions with tending ants. Ecology. 2017; **98**(3): 601-7.
3126. Roflumilast: doubtful efficacy but clear harms in COPD. Prescrire International. 2013; **22**(134): 5-9.
3127. Ağırbaşlı D, Ülman YI. [Coronary artery disease from a perspective of genomic risk score, ethical approaches and suggestions]. Anadolu Kardiyoloji Dergisi: AKD = The Anatolian Journal Of Cardiology. 2012; **12**(2): 171-7.
3128. Ali S, Drendel AL, Kircher J, Beno S. Pain management of musculoskeletal injuries in children: current state and future directions. Pediatric Emergency Care. 2010;

26(7): 518-24.
3129. Almli LM, Fani N, Smith AK, Ressler KJ. Genetic approaches to understanding post-traumatic stress disorder. The International Journal Of Neuropsychopharmacology. 2014; **17**(2): 355-70.
3130. An X, Hu J, Do K-A. SIFORM: shared informative factor models for integration of multi-platform bioinformatic data. Bioinformatics (Oxford, England). 2016; **32**(21): 3279-90.
3131. Andersson U, McKean-Cowdin R, Hjalmars U, Malmer B. Genetic variants in association studies--review of strengths and weaknesses in study design and current knowledge of impact on cancer risk. Acta Oncologica (Stockholm, Sweden). 2009; **48**(7): 948-54.
3132. Archer J, Baillie G, Watson SJ, Kellam P, Rambaut A, Robertson DL. Analysis of high-depth sequence data for studying viral diversity: a comparison of next generation sequencing platforms using Segminator II. BMC Bioinformatics. 2012; **13**: 47-.
3133. Archer T, Kostrzewa RM. Physical exercise alleviates ADHD symptoms: regional deficits and development trajectory. Neurotoxicity Research. 2012; **21**(2): 195-209.
3134. Ashton K, Hooper L, Harvey LJ, Hurst R, Casgrain A, Fairweather-Tait SJ. Methods of assessment of selenium status in humans: a systematic review. The American Journal Of Clinical Nutrition. 2009; **89**(6): 2025S-39S.
3135. Asif M, Jamal MS, Khan AR, Naseer MI, Hussain A, Choudhry H, et al. A Novel Four-Way Complex Variant Translocation Involving Chromosome 46,XY,t(4;9;19;22)(q25:q34;p13.3;q11.2) in a Chronic Myeloid Leukemia Patient. Frontiers In Oncology. 2016; **6**: 124-.
3136. Assimes TL, Salfati EL, Del Gobbo LC. Leveraging information from genetic risk scores of coronary atherosclerosis. Current Opinion In Lipidology. 2017; **28**(2): 104-12.
3137. Aziz K, Nowsheen S, Pantelias G, Iliakis G, Gorgoulis VG, Georgakilas AG. Targeting DNA damage and repair: embracing the pharmacological era for successful cancer therapy. Pharmacology & Therapeutics. 2012; **133**(3): 334-50.
3138. Bajer B, Vlcek M, Galusova A, Imrich R, Penesova A. Exercise associated hormonal signals as powerful determinants of an effective fat mass loss. Endocrine Regulations. 2015; **49**(3): 151-63.
3139. Band MM, Sumukadas D, Struthers AD, Avenell A, Donnan PT, Kemp PR, et al. Leucine and ACE inhibitors as therapies for sarcopenia (LACE trial): study protocol for a randomised controlled trial. Trials. 2018; **19**(1): 6-.
3140. Banks AP, Lawless C, Lydall DA. A quantitative fitness analysis workflow. Journal Of Visualized Experiments: Jove. 2012; (66).
3141. Baron R, Kneissel M. WNT signaling in bone homeostasis and disease: from human mutations to treatments. Nature Medicine. 2013; **19**(2): 179-92.
3142. Barr R, Macdonald H, Stewart A, McGuigan F, Rogers A, Eastell R, et al. Association between vitamin D receptor gene polymorphisms, falls, balance and muscle power: results from two independent studies (APOSS and OPUS). Osteoporosis International: A Journal Established As Result Of Cooperation Between The European Foundation For Osteoporosis And The National Osteoporosis Foundation Of The USA. 2010; **21**(3): 457-66.
3143. Bartlett AA, Singh R, Hunter RG. Anxiety and Epigenetics. Advances In

Experimental Medicine And Biology. 2017; **978**: 145-66.
3144. Bar-Zeev N, Kapanda L, King C, Beard J, Phiri T, Mvula H, et al. Methods and challenges in measuring the impact of national pneumococcal and rotavirus vaccine introduction on morbidity and mortality in Malawi. Vaccine. 2015; **33**(23): 2637-45.
3145. Battery L, Solomon A, Gould D. Gene doping: Olympic genes for Olympic dreams. Journal Of The Royal Society Of Medicine. 2011; **104**(12): 494-500.
3146. Beer CP. Caffeine: The forgotten variable. International Journal Of Psychiatry In Clinical Practice. 2001; **5**(4): 231-6.
3147. Benedetti F, Carlino E, Piedimonte A. Increasing uncertainty in CNS clinical trials: the role of placebo, nocebo, and Hawthorne effects. The Lancet Neurology. 2016; **15**(7): 736-47.
3148. Bienkowska JR, Dalgin GS, Batliwalla F, Allaire N, Roubenoff R, Gregersen PK, et al. Convergent Random Forest predictor: methodology for predicting drug response from genome-scale data applied to anti-TNF response. Genomics. 2009; **94**(6): 423-32.
3149. Billeci AMR, Agnelli G, Caso V. Stroke pharmacogenomics. Expert Opinion On Pharmacotherapy. 2009; **10**(18): 2947-57.
3150. Binder EB. The role of FKBP5, a co-chaperone of the glucocorticoid receptor in the pathogenesis and therapy of affective and anxiety disorders. Psychoneuroendocrinology. 2009; **34 Suppl 1**: S186-S95.
3151. Binder EB, Holsboer F. Pharmacogenomics. Handbook Of Experimental Pharmacology. 2005; (169): 527-46.
3152. Birch P, Friedman JM. Utility and limitations of genetic disease databases in clinical genetics research: a neurofibromatosis 1 database example. American Journal Of Medical Genetics Part C, Seminars In Medical Genetics. 2004; **125C**(1): 42-9.
3153. Bonjour J-P, Chevalley T, Rizzoli R, Ferrari S. Gene-environment interactions in the skeletal response to nutrition and exercise during growth. Medicine And Sport Science. 2007; **51**: 64-80.
3154. Booth FW, Laye MJ. The future: genes, physical activity and health. Acta Physiologica (Oxford, England). 2010; **199**(4): 549-56.
3155. Bouchard C. Genetic factors in obesity. The Medical Clinics Of North America. 1989; **73**(1): 67-81.
3156. Bouchard C. Current understanding of the etiology of obesity: genetic and nongenetic factors. The American Journal Of Clinical Nutrition. 1991; **53**(6 Suppl): 1561S-5S.
3157. Bouchard C. Gene-environment interactions in the etiology of obesity: defining the fundamentals. Obesity (Silver Spring, Md). 2008; **16 Suppl 3**: S5-S10.
3158. Bouchard C, Després JP, Mauriège P. Genetic and nongenetic determinants of regional fat distribution. Endocrine Reviews. 1993; **14**(1): 72-93.
3159. Bouchard C, Leon AS, Rao DC, Skinner JS, Wilmore JH, Gagnon J. The HERITAGE family study. Aims, design, and measurement protocol. Medicine And Science In Sports And Exercise. 1995; **27**(5): 721-9.
3160. Boyle P, Severi G, Giles GG. The epidemiology of prostate cancer. The Urologic Clinics Of North America. 2003; **30**(2): 209-17.
3161. Bracha HS, Lenze SM. Why are we using surgery as a first line of treatment for an anxiety disorder? Clinical Autonomic Research: Official Journal Of The Clinical

Autonomic Research Society. 2006; **16**(6): 406-.
3162. Broderick PJ, Benjamin AB, Dennis LW. Caffeine and psychiatric medication interactions: a review. The Journal Of The Oklahoma State Medical Association. 2005; **98**(8): 380-4.
3163. Brommage R, Ohlsson C. Translational studies provide insights for the etiology and treatment of cortical bone osteoporosis. Best Practice & Research Clinical Endocrinology & Metabolism. 2018; **32**(3): 329-40.
3164. Brouwer CAJ, Gietema JA, Kamps WA, de Vries EGE, Postma A. Changes in body composition after childhood cancer treatment: impact on future health status--a review. Critical Reviews In Oncology/Hematology. 2007; **63**(1): 32-46.
3165. Brüne M. On the role of oxytocin in borderline personality disorder. The British Journal Of Clinical Psychology. 2016; **55**(3): 287-304.
3166. Brun-Vézinet F, Costagliola D, Khaled MA, Calvez V, Clavel F, Clotet B, et al. Clinically validated genotype analysis: guiding principles and statistical concerns. Antiviral Therapy. 2004; **9**(4): 465-78.
3167. Burton CL, Arnold PD, Soreni N. Three Reasons why Studying Hoarding in Children and Adolescents is Important. Journal Of The Canadian Academy Of Child And Adolescent Psychiatry = Journal De L'academie Canadienne De Psychiatrie De L'enfant Et De L'adolescent. 2015; **24**(2): 128-30.
3168. Butali A, Mossey P, Tiffin N, Adeyemo W, Eshete M, Mumena C, et al. Multidisciplinary approach to genomics research in Africa: the AfriCRAN model. The Pan African Medical Journal. 2015; **21**: 229-.
3169. Butelman ER, Yuferov V, Kreek MJ. κ-opioid receptor/dynorphin system: genetic and pharmacotherapeutic implications for addiction. Trends In Neurosciences. 2012; **35**(10): 587-96.
3170. Cacabelos R. Pharmacogenomics and therapeutic prospects in dementia. European Archives Of Psychiatry And Clinical Neuroscience. 2008; **258 Suppl 1**: 28-47.
3171. Cai X, Shi X, Zhang X, Zhang A, Zheng M, Fang Y. The association between brain-derived neurotrophic factor gene polymorphism and migraine: a meta-analysis. The Journal Of Headache And Pain. 2017; **18**(1): 13-.
3172. Campos C, Rocha NBF, Lattari E, Paes F, Nardi AE, Machado S. Exercise-induced neuroprotective effects on neurodegenerative diseases: the key role of trophic factors. Expert Review Of Neurotherapeutics. 2016; **16**(6): 723-34.
3173. Cao Y, Wang X, Cao Z, Cheng X. Association of Vitamin D receptor gene TaqI polymorphisms with tuberculosis susceptibility: a meta-analysis. International Journal Of Clinical And Experimental Medicine. 2015; **8**(6): 10187-203.
3174. Cao Y, Wang X, Cao Z, Cheng X. Vitamin D receptor gene FokI polymorphisms and tuberculosis susceptibility: a meta-analysis. Archives Of Medical Science: AMS. 2016; **12**(5): 1118-34.
3175. Carraro U, Kern H, Gava P, Hofer C, Loefler S, Gargiulo P, et al. Biology of Muscle Atrophy and of its Recovery by FES in Aging and Mobility Impairments: Roots and By-Products. European Journal Of Translational Myology. 2015; **25**(4): 221-30.
3176. Cavagna L, Boffini N, Cagnotto G, Inverardi F, Grosso V, Caporali R. Atherosclerosis and rheumatoid arthritis: more than a simple association. Mediators Of Inflammation. 2012; **2012**: 147354-.

3177. Caviedes A, Lafourcade C, Soto C, Wyneken U. BDNF/NF-κB Signaling in the Neurobiology of Depression. Current Pharmaceutical Design. 2017; **23**(21): 3154-63.
3178. Cedarbaum JM, Stambler N. Performance of the Amyotrophic Lateral Sclerosis Functional Rating Scale (ALSFRS) in multicenter clinical trials. Journal Of The Neurological Sciences. 1997; **152 Suppl 1**: S1-S9.
3179. Chakraborty R, Sankaranarayanan K. Mutations in the BRCA1 gene: implications of inter-population differences for predicting the risk of radiation-induced breast cancers. Genetical Research. 1998; **72**(3): 191-8.
3180. Charles BA, Shriner D, Rotimi CN. Accounting for linkage disequilibrium in association analysis of diverse populations. Genetic Epidemiology. 2014; **38**(3): 265-73.
3181. Chartoff EH, Mavrikaki M. Sex Differences in Kappa Opioid Receptor Function and Their Potential Impact on Addiction. Frontiers In Neuroscience. 2015; **9**: 466-.
3182. Chen G-L, Miller GM. Tryptophan hydroxylase-2: an emerging therapeutic target for stress disorders. Biochemical Pharmacology. 2013; **85**(9): 1227-33.
3183. Chen P-Y, Wang S-C, Poland RE, Lin K-M. Biological variations in depression and anxiety between East and West. CNS Neuroscience & Therapeutics. 2009; **15**(3): 283-94.
3184. Chen S, Sutiman N, Zhang CZ, Yu Y, Lam S, Khor CC, et al. Pharmacogenetics of irinotecan, doxorubicin and docetaxel transporters in Asian and Caucasian cancer patients: a comparative review. Drug Metabolism Reviews. 2016; **48**(4): 502-40.
3185. Chiu C-T, Chuang D-M. Molecular actions and therapeutic potential of lithium in preclinical and clinical studies of CNS disorders. Pharmacology & Therapeutics. 2010; **128**(2): 281-304.
3186. Cholongitas E, Pipili C, Papatheodoridis GV. Interferon-free regimens in patients with hepatitis C infection and renal dysfunction or kidney transplantation. World Journal Of Hepatology. 2017; **9**(4): 180-90.
3187. Chu H, Wang M, Shi D, Ma L, Zhang Z, Tong N, et al. Hsa-miR-196a2 Rs11614913 polymorphism contributes to cancer susceptibility: evidence from 15 case-control studies. PLoS One. 2011; **6**(3): e18108-e.
3188. Chu H, Wang M, Zhong D, Shi D, Ma L, Tong N, et al. AdipoQ polymorphisms are associated with type 2 diabetes mellitus: a meta-analysis study. Diabetes/Metabolism Research And Reviews. 2013; **29**(7): 532-45.
3189. Chuang D-M. Neuroprotective and neurotrophic actions of the mood stabilizer lithium: can it be used to treat neurodegenerative diseases? Critical Reviews In Neurobiology. 2004; **16**(1-2): 83-90.
3190. Chuang D-M. The antiapoptotic actions of mood stabilizers: molecular mechanisms and therapeutic potentials. Annals Of The New York Academy Of Sciences. 2005; **1053**: 195-204.
3191. Chuang D-M, Manji HK. In search of the Holy Grail for the treatment of neurodegenerative disorders: has a simple cation been overlooked? Biological Psychiatry. 2007; **62**(1): 4-6.
3192. Chung WK, Leibel RL. Considerations regarding the genetics of obesity. Obesity (Silver Spring, Md). 2008; **16 Suppl 3**: S33-S9.
3193. Cobianchi S, Arbat-Plana A, Lopez-Alvarez VM, Navarro X. Neuroprotective Effects of Exercise Treatments After Injury: The Dual Role of Neurotrophic Factors.

Current Neuropharmacology. 2017; **15**(4): 495-518.
3194. Colagiuri B, Schenk LA, Kessler MD, Dorsey SG, Colloca L. The placebo effect: From concepts to genes. Neuroscience. 2015; **307**: 171-90.
3195. Coleman DC, Bennett DE, Sullivan DJ, Gallagher PJ, Henman MC, Shanley DB, et al. Oral Candida in HIV infection and AIDS: new perspectives/new approaches. Critical Reviews In Microbiology. 1993; **19**(2): 61-82.
3196. Collins M, Raleigh SM. Genetic risk factors for musculoskeletal soft tissue injuries. Medicine And Sport Science. 2009; **54**: 136-49.
3197. Cong Y, Ru J-Y, Bao N-R, Guo T, Zhao J-N. A single nucleotide polymorphism in the TGF-β1 gene (rs1982073 C>T) may contribute to increased risks of bone fracture, osteoporosis, and osteoarthritis: a meta-analysis. Clinical Rheumatology. 2016; **35**(4): 973-85.
3198. Connor KM, Zhang W. Recent advances in the understanding and treatment of anxiety disorders. Resilience: determinants, measurement, and treatment responsiveness. CNS Spectrums. 2006; **11**(10 Suppl 12): 5-12.
3199. Correa M, Font L. Is there a major role for adenosine A2A receptors in anxiety? Frontiers In Bioscience: A Journal And Virtual Library. 2008; **13**: 4058-70.
3200. Costa MA, Barbosa A, Neto E, Sá-e-Sousa A, Freitas R, Neves JM, et al. On the role of subtype selective adenosine receptor agonists during proliferation and osteogenic differentiation of human primary bone marrow stromal cells. Journal Of Cellular Physiology. 2011; **226**(5): 1353-66.
3201. Crabbe JC, Phillips TJ, Belknap JK. The complexity of alcohol drinking: studies in rodent genetic models. Behavior Genetics. 2010; **40**(6): 737-50.
3202. Crawley JN. Mouse behavioral assays relevant to the symptoms of autism. Brain Pathology (Zurich, Switzerland). 2007; **17**(4): 448-59.
3203. Crona J, Taïeb D, Pacak K. New Perspectives on Pheochromocytoma and Paraganglioma: Toward a Molecular Classification. Endocrine Reviews. 2017; **38**(6): 489-515.
3204. Cuciureanu M, Vlase L, Muntean D, Varlan I, Cuciureanu R. Grapefruit juice--drug interactions: importance for pharmacotherapy. Revista Medico-Chirurgicala A Societatii De Medici Si Naturalisti Din Iasi. 2010; **114**(3): 885-91.
3205. Cunha RA, Ferré S, Vaugeois J-M, Chen J-F. Potential therapeutic interest of adenosine A2A receptors in psychiatric disorders. Current Pharmaceutical Design. 2008; **14**(15): 1512-24.
3206. D'Abbraccio M, Busto A, De Marco M, Figoni M, Maddaloni A, Abrescia N. Efficacy and Tolerability of Integrase Inhibitors in Antiretroviral-Naive Patients. AIDS Reviews. 2015; **17**(3): 171-85.
3207. Dahabreh IJ, Moorthy D, Lamont JL, Chen ML, Kent DM, Lau J. Testing of CYP2C19 Variants and Platelet Reactivity for Guiding Antiplatelet Treatment. 2013.
3208. Dalvie S, Koen N, McGregor N, O'Connell K, Warnich L, Ramesar R, et al. Toward a Global Roadmap for Precision Medicine in Psychiatry: Challenges and Opportunities. Omics: A Journal Of Integrative Biology. 2016; **20**(10): 557-64.
3209. Dato S, Crocco P, D'Aquila P, de Rango F, Bellizzi D, Rose G, et al. Exploring the role of genetic variability and lifestyle in oxidative stress response for healthy aging and longevity. International Journal Of Molecular Sciences. 2013; **14**(8): 16443-72.

3210. Dauncey MJ. Recent advances in nutrition, genes and brain health. The Proceedings Of The Nutrition Society. 2012; **71**(4): 581-91.
3211. Dean L. Diazepam Therapy and CYP2C19 Genotype. Medical Genetics Summaries. 2012.
3212. Deckert J. The adenosine A(2A) receptor knockout mouse: a model for anxiety? The International Journal Of Neuropsychopharmacology. 1998; **1**(2): 187-90.
3213. Deng W, Cheung ST, Tsao SW, Wang XM, Tiwari AFY. Telomerase activity and its association with psychological stress, mental disorders, lifestyle factors and interventions: A systematic review. Psychoneuroendocrinology. 2016; **64**: 150-63.
3214. Denham J, Marques FZ, O'Brien BJ, Charchar FJ. Exercise: putting action into our epigenome. Sports Medicine (Auckland, NZ). 2014; **44**(2): 189-209.
3215. Divari S, Valetti F, Caposio P, Pessione E, Cavaletto M, Griva E, et al. The oxygenase component of phenol hydroxylase from Acinetobacter radioresistens S13. European Journal Of Biochemistry. 2003; **270**(10): 2244-53.
3216. Dlouhá D, Hubáček JA. [FTO gene and his role in genetic determination of obesity]. Vnitrni Lekarstvi. 2012; **58**(3): 208-15.
3217. Do EK, Maes HH. Genotype x Environment Interaction in Smoking Behaviors: A Systematic Review. Nicotine Tob Res. 2017; **19**(4): 387-400.
3218. Do EK, Maes HH. Genotype × Environment Interaction in Smoking Behaviors: A Systematic Review. Nicotine & Tobacco Research: Official Journal Of The Society For Research On Nicotine And Tobacco. 2017; **19**(4): 387-400.
3219. Domschke K, Dannlowski U, Ohrmann P, Lawford B, Bauer J, Kugel H, et al. Cannabinoid receptor 1 (CNR1) gene: impact on antidepressant treatment response and emotion processing in major depression. European Neuropsychopharmacology: The Journal Of The European College Of Neuropsychopharmacology. 2008; **18**(10): 751-9.
3220. Domschke K, Deckert J. Genetics. Current Topics In Behavioral Neurosciences. 2010; **2**: 63-75.
3221. Donnelly EM, Quach ET, Hillery TM, Heeke BL, Snyder BR, Handy CR, et al. Characterization of a murine model of SMA. Neurobiology Of Disease. 2012; **45**(3): 992-8.
3222. Drago A, Ronchi DD, Serretti A. 5-HT1A gene variants and psychiatric disorders: a review of current literature and selection of SNPs for future studies. The International Journal Of Neuropsychopharmacology. 2008; **11**(5): 701-21.
3223. Du J, Machado-Vieira R, Khairova R. Synaptic plasticity in the pathophysiology and treatment of bipolar disorder. Curr Top Behav Neurosci. 2011; **5**: 167-85.
3224. Duttaroy AK. Postprandial activation of hemostatic factors: role of dietary fatty acids. Prostaglandins, Leukotrienes, And Essential Fatty Acids. 2005; **72**(6): 381-91.
3225. Ejlertsen B. Adjuvant chemotherapy in early breast cancer. Danish Medical Journal. 2016; **63**(5).
3226. Engin E, Liu J, Rudolph U. α2-containing GABA(A) receptors: a target for the development of novel treatment strategies for CNS disorders. Pharmacology & Therapeutics. 2012; **136**(2): 142-52.
3227. Ennour-Idrissi K, Maunsell E, Diorio C. Telomere Length and Breast Cancer Prognosis: A Systematic Review. Cancer Epidemiology, Biomarkers & Prevention: A

Publication Of The American Association For Cancer Research, Cosponsored By The American Society Of Preventive Oncology. 2017; **26**(1): 3-10.
3228. Enoch M-A, Schuckit MA, Johnson BA, Goldman D. Genetics of alcoholism using intermediate phenotypes. Alcoholism, Clinical And Experimental Research. 2003; **27**(2): 169-76.
3229. Esteves SC, Roque M, Garrido N. Use of testicular sperm for intracytoplasmic sperm injection in men with high sperm DNA fragmentation: a SWOT analysis. Asian Journal Of Andrology. 2018; **20**(1): 1-8.
3230. Evans JC, Mizrahi V. The application of tetracyclineregulated gene expression systems in the validation of novel drug targets in Mycobacterium tuberculosis. Frontiers In Microbiology. 2015; **6**: 812-.
3231. Everson RB. Scientific horizons in cancer control: genomes, informatics and personalized medicine. Connecticut Medicine. 2012; **76**(6): 359-64.
3232. Fabbri C, Di Girolamo G, Serretti A. Pharmacogenetics of antidepressant drugs: an update after almost 20 years of research. American Journal Of Medical Genetics Part B, Neuropsychiatric Genetics: The Official Publication Of The International Society Of Psychiatric Genetics. 2013; **162B**(6): 487-520.
3233. Fan W, Evans RM. Exercise Mimetics: Impact on Health and Performance. Cell Metabolism. 2017; **25**(2): 242-7.
3234. Feduccia AA, Mithoefer MC. MDMA-assisted psychotherapy for PTSD: Are memory reconsolidation and fear extinction underlying mechanisms? Prog Neuropsychopharmacol Biol Psychiatry. 2018; **84**(Pt A): 221-8.
3235. Ferreira-Garcia R, Mochcovitch M, Costa do Cabo M, Nardi AE, Christophe Freire R. Predictors of Pharmacotherapy Response in Generalized Anxiety Disorder: A Systematic Review. Harvard Review Of Psychiatry. 2017; **25**(2): 65-79.
3236. Fischetto G, Bermon S. From gene engineering to gene modulation and manipulation: can we prevent or detect gene doping in sports? Sports Medicine (Auckland, NZ). 2013; **43**(10): 965-77.
3237. Fister K, Polasek O, Vuletić S, Kern J. Single nucleotide polymorphisms and health behaviours related to obesity - trawling the evidence in the prospect of personalised prevention. Studies In Health Technology And Informatics. 2009; **150**: 762-6.
3238. Fitch KD. An overview of asthma and airway hyper-responsiveness in Olympic athletes. British Journal Of Sports Medicine. 2012; **46**(6): 413-6.
3239. Flandre P, O'Quigley J. Predictive strength of Jonckheere's test for trend: an application to genotypic scores in HIV infection. Statistics In Medicine. 2007; **26**(24): 4441-54.
3240. Flueck M, Eilers W. Training modalities: impact on endurance capacity. Endocrinology And Metabolism Clinics Of North America. 2010; **39**(1): 183.
3241. Ford C, Yusim K, Ioerger T, Feng S, Chase M, Greene M, et al. Mycobacterium tuberculosis--heterogeneity revealed through whole genome sequencing. Tuberculosis (Edinburgh, Scotland). 2012; **92**(3): 194-201.
3242. Fornaro M, Solmi M, Veronese N, De Berardis D, Buonaguro EF, Tomasetti C, et al. The burden of mood-disorder/cerebrovascular disease comorbidity: essential neurobiology, psychopharmacology, and physical activity interventions. International

Review Of Psychiatry (Abingdon, England). 2017; **29**(5): 425-35.
3243. Foster PP, Rosenblatt KP, Kuljiš RO. Exercise-induced cognitive plasticity, implications for mild cognitive impairment and Alzheimer's disease. Frontiers In Neurology. 2011; **2**: 28-.
3244. Fowler B. [Homocystein--an independent risk factor for cardiovascular and thrombotic diseases]. Therapeutische Umschau Revue Therapeutique. 2005; **62**(9): 641-6.
3245. Francies HE, Garnett MJ. What role could organoids play in the personalization of cancer treatment? Pharmacogenomics. 2015; **16**(14): 1523-6.
3246. Franco-Tormo MJ, Salas-Crisostomo M, Rocha NB, Budde H, Machado S, Murillo-Rodríguez E. CRISPR/Cas9, the Powerful New Genome-Editing Tool for Putative Therapeutics in Obesity. Journal Of Molecular Neuroscience: MN. 2018; **65**(1): 10-6.
3247. Franks PW, Ling C. Epigenetics and obesity: the devil is in the details. BMC Medicine. 2010; **8**: 88-.
3248. Freudenthal B, Logan J, Croucher PI, Williams GR, Bassett JHD. Rapid phenotyping of knockout mice to identify genetic determinants of bone strength. The Journal Of Endocrinology. 2016; **231**(1): R31-R46.
3249. García de Diego L, Cuervo M, Martínez JA. [Software for performing a global phenotypic and genotypic nutritional assessment]. Nutricion Hospitalaria. 2013; **28**(5): 1622-32.
3250. Garner M, Möhler H, Stein DJ, Mueggler T, Baldwin DS. Research in anxiety disorders: from the bench to the bedside. European Neuropsychopharmacology: The Journal Of The European College Of Neuropsychopharmacology. 2009; **19**(6): 381-90.
3251. Gasser T, Hardy J, Mizuno Y. Milestones in PD genetics. Movement Disorders: Official Journal Of The Movement Disorder Society. 2011; **26**(6): 1042-8.
3252. Gershenfeld HK, Philibert RA, Boehm GW. Looking forward in geriatric anxiety and depression: implications of basic science for the future. The American Journal Of Geriatric Psychiatry: Official Journal Of The American Association For Geriatric Psychiatry. 2005; **13**(12): 1027-40.
3253. Gilbody S, Lewis S, Lightfoot T. Methylenetetrahydrofolate reductase (MTHFR) genetic polymorphisms and psychiatric disorders: a HuGE review. American Journal Of Epidemiology. 2007; **165**(1): 1-13.
3254. Gillihan SJ, Parens E. Should we expect "neural signatures" for DSM diagnoses? The Journal Of Clinical Psychiatry. 2011; **72**(10): 1383-9.
3255. Girgis CM, Clifton-Bligh RJ, Turner N, Lau SL, Gunton JE. Effects of vitamin D in skeletal muscle: falls, strength, athletic performance and insulin sensitivity. Clinical Endocrinology. 2014; **80**(2): 169-81.
3256. Gladstone JP, Dodick DW. Migraine and cerebral white matter lesions: when to suspect cerebral autosomal dominant arteriopathy with subcortical infarcts and leukoencephalopathy (CADASIL). The Neurologist. 2005; **11**(1): 19-29.
3257. Goldfeder RL, Wall DP, Khoury MJ, Ioannidis JPA, Ashley EA. Human Genome Sequencing at the Population Scale: A Primer on High-Throughput DNA Sequencing and Analysis. American Journal Of Epidemiology. 2017; **186**(8): 1000-9.
3258. Gonem S, Raj V, Wardlaw AJ, Pavord ID, Green R, Siddiqui S. Phenotyping

airways disease: an A to E approach. Clinical And Experimental Allergy: Journal Of The British Society For Allergy And Clinical Immunology. 2012; **42**(12): 1664-83.
3259. Gorman KF, Julien C, Moreau A. The genetic epidemiology of idiopathic scoliosis. European Spine Journal: Official Publication Of The European Spine Society, The European Spinal Deformity Society, And The European Section Of The Cervical Spine Research Society. 2012; **21**(10): 1905-19.
3260. Grimaldi KA, Paoli A, Smith GJ. Personal genetics: sports utility vehicle? Recent Patents On DNA & Gene Sequences. 2012; **6**(3): 209-15.
3261. Guo X-F, Wang J, Lei X-F, Zeng Y-P, Lv X-G, Dong W-G. XPD Asp312Asn polymorphism and esophageal cancer risk: an update meta-analysis based on 3928 cases and 6012 controls. International Journal Of Clinical And Experimental Medicine. 2014; **7**(9): 2443-52.
3262. Gupta D, Sheikh S, Pallagatti S, Kasariya K, Buttan A, Gupta M. Burning Mouth Syndrome due to Television Moans, an Enigma for Oral Physician: Treatment with Counseling. Journal Of Dental Research, Dental Clinics, Dental Prospects. 2014; **8**(2): 118-22.
3263. Hackett CS, Geurts AM, Hackett PB. Predicting preferential DNA vector insertion sites: implications for functional genomics and gene therapy. Genome Biology. 2007; **8 Suppl 1**: S12-S.
3264. Haferlach C, Bacher U, Tiu R, Maciejewski JP, List A. Myelodysplastic syndromes with del(5q): indications and strategies for cytogenetic testing. Cancer Genetics And Cytogenetics. 2008; **187**(2): 101-11.
3265. Haga SB, Mills R, Bosworth H. Striking a balance in communicating pharmacogenetic test results: promoting comprehension and minimizing adverse psychological and behavioral response. Patient Education And Counseling. 2014; **97**(1): 10-5.
3266. Hagberg JM, Moore GE, Ferrell RE. Specific genetic markers of endurance performance and VO2max. Exercise And Sport Sciences Reviews. 2001; **29**(1): 15-9.
3267. Hagberg JM, Park JJ, Brown MD. The role of exercise training in the treatment of hypertension: an update. Sports Medicine (Auckland, NZ). 2000; **30**(3): 193-206.
3268. Hamdani N, Ades J, Gorwood P. [Heritability and candidate genes in tobacco use]. L'encephale. 2006; **32**(6 Pt 1): 966-75.
3269. Hamilton SR. Targeted therapy of cancer: new roles for pathologists in colorectal cancer. Modern Pathology: An Official Journal Of The United States And Canadian Academy Of Pathology, Inc. 2008; **21 Suppl 2**: S23-S30.
3270. Hans S, Gimpel M, Glauche F, Neubauer P, Cruz-Bournazou MN. Automated Cell Treatment for Competence and Transformation of Escherichia coli in a High-Throughput Quasi-Turbidostat Using Microtiter Plates. Microorganisms. 2018; **6**(3).
3271. Hansen BC. Obesity, diabetes, and insulin resistance: implications from molecular biology, epidemiology, and experimental studies in humans and animals. Synopsis of the American Diabetes Association's 29th Research Symposium and Satellite Conference of the 7th International Congress on Obesity, Boston, Massachusetts. Diabetes Care. 1995; **18**(6): A2-A9.
3272. Hao Z, Li X, Dai J, Zhao B, Jiang Q. Genetic effects of rs3740199 polymorphism in ADAM12 gene on knee osteoarthritis: a meta-analysis. Journal Of Orthopaedic

Surgery And Research. 2017; **12**(1): 94-.
3273. Hariri AR, Weinberger DR. Functional neuroimaging of genetic variation in serotonergic neurotransmission. Genes, Brain, And Behavior. 2003; **2**(6): 341-9.
3274. Harmon KG, Drezner JA, Gammons M, Guskiewicz KM, Halstead M, Herring SA, et al. American Medical Society for Sports Medicine position statement: concussion in sport. British Journal Of Sports Medicine. 2013; **47**(1): 15-26.
3275. Harrison NR, Laroche FJF, Gutierrez A, Feng H. Zebrafish Models of Human Leukemia: Technological Advances and Mechanistic Insights. Advances In Experimental Medicine And Biology. 2016; **916**: 335-69.
3276. Hartman SJ, Natarajan L, Palmer BW, Parker B, Patterson RE, Sears DD. Impact of increasing physical activity on cognitive functioning in breast cancer survivors: Rationale and study design of Memory & Motion. Contemporary Clinical Trials. 2015; **45**(Pt B): 371-6.
3277. Hauger RL, Risbrough V, Oakley RH, Olivares-Reyes JA, Dautzenberg FM. Role of CRF receptor signaling in stress vulnerability, anxiety, and depression. Annals Of The New York Academy Of Sciences. 2009; **1179**: 120-43.
3278. He J, Yu G, Li Z, Liang H. Influence of interleukin-28B polymorphism on progression to hepatitis virus-induced hepatocellular carcinoma. Tumour Biology: The Journal Of The International Society For Oncodevelopmental Biology And Medicine. 2014; **35**(9): 8757-63.
3279. Heavey PM, McKenna D, Rowland IR. Colorectal cancer and the relationship between genes and the environment. Nutrition And Cancer. 2004; **48**(2): 124-41.
3280. Heilmann-Heimbach S, Hochfeld LM, Paus R, Nöthen MM. Hunting the genes in male-pattern alopecia: how important are they, how close are we and what will they tell us? Experimental Dermatology. 2016; **25**(4): 251-7.
3281. Helton SG, Lohoff FW. Serotonin pathway polymorphisms and the treatment of major depressive disorder and anxiety disorders. Pharmacogenomics. 2015; **16**(5): 541-53.
3282. Henquet C, Di Forti M, Morrison P, Kuepper R, Murray RM. Gene-environment interplay between cannabis and psychosis. Schizophrenia Bulletin. 2008; **34**(6): 1111-21.
3283. Hermes GDA, Zoetendal EG, Smidt H. Molecular ecological tools to decipher the role of our microbial mass in obesity. Beneficial Microbes. 2015; **6**(1): 61-81.
3284. Herpfer I, Lieb K. Substance P and Substance P receptor antagonists in the pathogenesis and treatment of affective disorders. The World Journal Of Biological Psychiatry: The Official Journal Of The World Federation Of Societies Of Biological Psychiatry. 2003; **4**(2): 56-63.
3285. Herring MP, Sailors MH, Bray MS. Genetic factors in exercise adoption, adherence and obesity. Obesity Reviews: An Official Journal Of The International Association For The Study Of Obesity. 2014; **15**(1): 29-39.
3286. Holmes RD, Tiwari AK, Kennedy JL. Mechanisms of the placebo effect in pain and psychiatric disorders. The Pharmacogenomics Journal. 2016; **16**(6): 491-500.
3287. Hsing AW, Chokkalingam AP. Prostate cancer epidemiology. Frontiers In Bioscience: A Journal And Virtual Library. 2006; **11**: 1388-413.
3288. Hu J, Zhou G-W, Wang N, Wang Y-J. MTRR A66G polymorphism and breast

cancer risk: a meta-analysis. Breast Cancer Research And Treatment. 2010; **124**(3): 779-84.
3289. Hu M, Mak VWL, Tomlinson B. Simvastatin-induced myopathy, the role of interaction with diltiazem and genetic predisposition. Journal Of Clinical Pharmacy And Therapeutics. 2011; **36**(3): 419-25.
3290. Hu Q, Zhang S-Y, Liu F, Zhang XJ, Cui G-C, Yu E-Q, et al. Influence of GNB3 C825T polymorphism on the efficacy of antidepressants in the treatment of major depressive disorder: A meta-analysis. Journal Of Affective Disorders. 2015; **172**: 103-9.
3291. Hubka P. Neural network plasticity, BDNF and behavioral interventions in Alzheimer's disease. Bratislavske Lekarske Listy. 2006; **107**(9-10): 395-401.
3292. Hughes RA, Pritchard J, Hadden RD. Pharmacological treatment other than corticosteroids, intravenous immunoglobulin and plasma exchange for Guillain Barre syndrome. Cochrane Database Syst Rev. 2011; (3): CD008630.
3293. Hughes RAC, Pritchard J, Hadden RDM. Pharmacological treatment other than corticosteroids, intravenous immunoglobulin and plasma exchange for Guillain-Barré syndrome. The Cochrane Database Of Systematic Reviews. 2013; (2): CD008630.
3294. Hunsberger J, Austin DR, Henter ID, Chen G. The neurotrophic and neuroprotective effects of psychotropic agents. Dialogues In Clinical Neuroscience. 2009; **11**(3): 333-48.
3295. Iizuka K, Machida T, Hirafuji M. Skeletal muscle is an endocrine organ. Journal Of Pharmacological Sciences. 2014; **125**(2): 125-31.
3296. Intlekofer KA, Cotman CW. Exercise counteracts declining hippocampal function in aging and Alzheimer's disease. Neurobiology Of Disease. 2013; **57**: 47-55.
3297. Introne WJ, Gahl WA. Alkaptonuria. GeneReviews®. 1993.
3298. Inzaule SC, Hamers RL, Paredes R, Yang C, Schuurman R, Rinke de Wit TF. The Evolving Landscape of HIV Drug Resistance Diagnostics for Expanding Testing in Resource-Limited Settings. AIDS Reviews. 2017; **19**(4): 219-30.
3299. Iwasaki LR, Covell DA, Jr., Frazier-Bowers SA, Kapila S, Huja SS, Nickel JC. Personalized and precision orthodontic therapy. Orthodontics & Craniofacial Research. 2015; **18 Suppl 1**: 1-7.
3300. Jalal S, Earley JN, Turchi JJ. DNA repair: from genome maintenance to biomarker and therapeutic target. Clinical Cancer Research: An Official Journal Of The American Association For Cancer Research. 2011; **17**(22): 6973-84.
3301. Janssens PLHR, Hursel R, Westerterp-Plantenga MS. Nutraceuticals for body-weight management: The role of green tea catechins. Physiology & Behavior. 2016; **162**: 83-7.
3302. Jiang G, Yu K, Shao L, Yu X, Hu C, Qian P, et al. Association between epidermal growth factor gene +61A/G polymorphism and the risk of hepatocellular carcinoma: a meta-analysis based on 16 studies. BMC Cancer. 2015; **15**: 314-.
3303. Jung SY, Sobel EM, Papp JC, Crandall CJ, Fu AN, Zhang Z-F. Obesity and associated lifestyles modify the effect of glucose metabolism-related genetic variants on impaired glucose homeostasis among postmenopausal women. Genetic Epidemiology. 2016; **40**(6): 520-30.
3304. Kapoor R, Tan-Koi WC, Teo Y-Y. Role of pharmacogenetics in public health and clinical health care: a SWOT analysis. European Journal Of Human Genetics: EJHG.

2016; **24**(12): 1651-7.
3305. Keers R, Aitchison KJ. Gender differences in antidepressant drug response. International Review Of Psychiatry (Abingdon, England). 2010; **22**(5): 485-500.
3306. Khan RJ, Gebreab SY, Riestra P, Xu R, Davis SK. Parent-offspring association of metabolic syndrome in the Framingham Heart Study. Diabetology & Metabolic Syndrome. 2014; **6**(1): 140-.
3307. Kilpeläinen TO, Franks PW. Gene-physical activity interactions and their impact on diabetes. Medicine And Sport Science. 2014; **60**: 94-103.
3308. Kim H, Clark D, Dionne RA. Genetic contributions to clinical pain and analgesia: avoiding pitfalls in genetic research. The Journal Of Pain: Official Journal Of The American Pain Society. 2009; **10**(7): 663-93.
3309. Klionsky DJ, Abdelmohsen K, Abe A, Abedin MJ, Abeliovich H, Acevedo Arozena A, et al. Guidelines for the use and interpretation of assays for monitoring autophagy (3rd edition). Autophagy. 2016; **12**(1): 1-222.
3310. Koch WH. Technology platforms for pharmacogenomic diagnostic assays. Nature Reviews Drug Discovery. 2004; **3**(9): 749-61.
3311. Konopka A, Mak M, Grzywacz A, Murawiec S, Samochowiec J. Genetic and psychosocial factors for benzodiazepine addiction. An analysis based on the results of the authors' own research conducted in a group of benzodiazepine addicted and non-addicted individuals. Postepy Higieny I Medycyny Doswiadczalnej (Online). 2017; **71**(0): 205-13.
3312. Kronenberg S, Frisch A, Rotberg B, Carmel M, Apter A, Weizman A. Pharmacogenetics of selective serotonin reuptake inhibitors in pediatric depression and anxiety. Pharmacogenomics. 2008; **9**(11): 1725-36.
3313. Kurian P, Obisesan TO, Craddock TJA. Oxidative species-induced excitonic transport in tubulin aromatic networks: Potential implications for neurodegenerative disease. Journal Of Photochemistry And Photobiology B, Biology. 2017; **175**: 109-24.
3314. Kuzelova H, Ptacek R, Macek M. The serotonin transporter gene (5-HTT) variant and psychiatric disorders: review of current literature. Neuro Endocrinology Letters. 2010; **31**(1): 4-10.
3315. Laje G. Pharmacogenetics of mood disorders: what clinicians need to know. CNS Spectrums. 2013; **18**(5): 272-84.
3316. Laje G, McMahon FJ. Genome-wide association studies of antidepressant outcome: a brief review. Progress In Neuro-Psychopharmacology & Biological Psychiatry. 2011; **35**(7): 1553-7.
3317. Landgraf R. The involvement of the vasopressin system in stress-related disorders. CNS & Neurological Disorders Drug Targets. 2006; **5**(2): 167-79.
3318. Langdon SP. Animal modeling of cancer pathology and studying tumor response to therapy. Current Drug Targets. 2012; **13**(12): 1535-47.
3319. Lapik IA, Gapparova KM, Chekhonina YG, Sorikina EY, Borodina SV. [Current trends in nutrigenomics of obesity]. Voprosy Pitaniia. 2016; **85**(6): 6-13.
3320. Lara DR. Caffeine, mental health, and psychiatric disorders. Journal Of Alzheimer's Disease: JAD. 2010; **20 Suppl 1**: S239-S48.
3321. Lazary J, Juhasz G, Hunyady L, Bagdy G. Personalized medicine can pave the way for the safe use of CB_1 receptor antagonists. Trends In Pharmacological Sciences.

2011; **32**(5): 270-80.
3322. Le François B, Czesak M, Steubl D, Albert PR. Transcriptional regulation at a HTR1A polymorphism associated with mental illness. Neuropharmacology. 2008; **55**(6): 977-85.
3323. Leibel RL. Single gene obesities in rodents: possible relevance to human obesity. The Journal Of Nutrition. 1997; **127**(9): 1908S-S.
3324. Levine JA, Vander Weg MW, Hill JO, Klesges RC. Non-exercise activity thermogenesis: the crouching tiger hidden dragon of societal weight gain. Arteriosclerosis, Thrombosis, And Vascular Biology. 2006; **26**(4): 729-36.
3325. Li D-J, Xiao D. Association between the XRCC1 polymorphisms and clinical outcomes of advanced NSCLC treated with platinum-based chemotherapy: a meta-analysis based on the PRISMA statement. BMC Cancer. 2017; **17**(1): 501-.
3326. Liao R-x, Yu M, Jiang Y, Xia W. Management of osteoporosis with calcitriol in elderly Chinese patients: a systematic review. Clinical Interventions In Aging. 2014; **9**: 515-26.
3327. Lin M, Wu R. Theoretical basis for the identification of allelic variants that encode drug efficacy and toxicity. Genetics. 2005; **170**(2): 919-28.
3328. Lin Z, Zhang J, Ma X, Yang S, Tian N, Lin X, et al. The Role of Interferon Lambda 3 Genetic Polymorphisms in Response to Interferon Therapy in Chronic Hepatitis B Patients: An Updated Meta-Analysis. Hepatitis Monthly. 2016; **16**(7): e37534-e.
3329. Little J, Higgins JPT, Ioannidis JPA, Moher D, Gagnon F, von Elm E, et al. Strengthening the reporting of genetic association studies (STREGA): an extension of the strengthening the reporting of observational studies in epidemiology (STROBE) statement. Journal Of Clinical Epidemiology. 2009; **62**(6): 597-608.e4.
3330. Liu D, Tian Y, Sun D, Sun H, Jin Y, Dong M. The FCGR3A polymorphism predicts the response to rituximab-based therapy in patients with non-Hodgkin lymphoma: a meta-analysis. Annals Of Hematology. 2016; **95**(9): 1483-90.
3331. Lojovich JM. The relationship between aerobic exercise and cognition: is movement medicinal? The Journal Of Head Trauma Rehabilitation. 2010; **25**(3): 184-92.
3332. Looker AC. Interaction of science, consumer practices and policy: calcium and bone health as a case study. The Journal Of Nutrition. 2003; **133**(6): 1987S-91S.
3333. Lopes LV, Sebastião AM, Ribeiro JA. Adenosine and related drugs in brain diseases: present and future in clinical trials. Current Topics In Medicinal Chemistry. 2011; **11**(8): 1087-101.
3334. López-Cruz L, Salamone JD, Correa M. Caffeine and Selective Adenosine Receptor Antagonists as New Therapeutic Tools for the Motivational Symptoms of Depression. Frontiers In Pharmacology. 2018; **9**: 526-.
3335. Lopez-Miranda J, Williams C, Lairon D. Dietary, physiological, genetic and pathological influences on postprandial lipid metabolism. The British Journal Of Nutrition. 2007; **98**(3): 458-73.
3336. Lu B, Nagappan G, Lu Y. BDNF and synaptic plasticity, cognitive function, and dysfunction. Handbook Of Experimental Pharmacology. 2014; **220**: 223-50.
3337. Lueken U, Zierhut KC, Hahn T, Straube B, Kircher T, Reif A, et al. Neurobiological markers predicting treatment response in anxiety disorders: A systematic review and implications for clinical application. Neuroscience And Biobehavioral Reviews. 2016; **66**:

143-62.
3338. Luo Y-Q, Wang D, Gong T, Zhu J. An updated meta-analysis of 37 case-control studies on the association between NFKB1 -94ins/del ATTG promoter polymorphism and cancer susceptibility. Oncotarget. 2016; **7**(36): 58659-70.
3339. Madan A, Walker CR, Weinstein B, Fowler JC. Pharmacogenomics in practice: a case report of personalized inpatient psychiatric care. Pharmacogenomics. 2015; **16**(5): 433-9.
3340. Makizako H, Tsutsumimoto K, Doi T, Hotta R, Nakakubo S, Liu-Ambrose T, et al. Effects of exercise and horticultural intervention on the brain and mental health in older adults with depressive symptoms and memory problems: study protocol for a randomized controlled trial [UMIN000018547]. Trials. 2015; **16**: 499-.
3341. Manji HK, Quiroz JA, Payne JL, Singh J, Lopes BP, Viegas JS, et al. The underlying neurobiology of bipolar disorder. World Psychiatry: Official Journal Of The World Psychiatric Association (WPA). 2003; **2**(3): 136-46.
3342. Marcelin A-G, Flandre P, Peytavin G, Calvez V. Predictors of virologic response to ritonavir-boosted protease inhibitors. AIDS Reviews. 2005; **7**(4): 225-32.
3343. Marichalar-Mendia X, Rodriguez-Tojo MJ, Acha-Sagredo A, Rey-Barja N, Aguirre-Urizar JM. Oral cancer and polymorphism of ethanol metabolising genes. Oral Oncology. 2010; **46**(1): 9-13.
3344. Marti A, Martinez-González MA, Martinez JA. Interaction between genes and lifestyle factors on obesity. The Proceedings Of The Nutrition Society. 2008; **67**(1): 1-8.
3345. Massoud F, Belleville S, Bergman H, Kirk J, Chertkow H, Nasreddine Z, et al. Mild cognitive impairment and cognitive impairment, no dementia: Part B, therapy. Alzheimer's & Dementia: The Journal Of The Alzheimer's Association. 2007; **3**(4): 283-91.
3346. Masuda R, Itoh M, Suzuki T. [Duloxetine for chronic pain management: pharmacology and clinical use]. Masui The Japanese Journal Of Anesthesiology. 2013; **62**(7): 814-21.
3347. Mathew SJ, Ho S. Etiology and neurobiology of social anxiety disorder. The Journal Of Clinical Psychiatry. 2006; **67 Suppl 12**: 9-13.
3348. Matteucci E, Giampietro O. Building a bridge between clinical and basic research: the phenotypic elements of familial predisposition to type 1 diabetes. Current Medicinal Chemistry. 2007; **14**(5): 555-67.
3349. Mattson MP, Duan W, Chan SL, Cheng A, Haughey N, Gary DS, et al. Neuroprotective and neurorestorative signal transduction mechanisms in brain aging: modification by genes, diet and behavior. Neurobiology Of Aging. 2002; **23**(5): 695-705.
3350. McArdle JJ, Prescott CA. Contemporary Modeling of Gene × Environment Effects in Randomized Multivariate Longitudinal Studies. Perspectives On Psychological Science: A Journal Of The Association For Psychological Science. 2010; **5**(5): 606-21.
3351. McClain MR, Palomaki GE, Piper M, Haddow JE. A rapid-ACCE review of CYP2C9 and VKORC1 alleles testing to inform warfarin dosing in adults at elevated risk for thrombotic events to avoid serious bleeding. Genetics In Medicine: Official Journal Of The American College Of Medical Genetics. 2008; **10**(2): 89-98.
3352. McCoy JG, Marugan JJ, Liu K, Zheng W, Southall N, Huang W, et al. Selective Modulation of Gq/Gs pathways by Naphtho Pyrano Pyrimidines as antagonists of the

Neuropeptide S Receptor. ACS Chemical Neuroscience. 2010; **1**(8): 559-74.
3353. McLean AR, Adlen EK, Cardis E, Elliott A, Goodhead DT, Harms-Ringdahl M, et al. A restatement of the natural science evidence base concerning the health effects of low-level ionizing radiation. Proceedings Biological Sciences. 2017; **284**(1862).
3354. Meltzer HY, Brennan MD, Woodward ND, Jayathilake K. Association of Sult4A1 SNPs with psychopathology and cognition in patients with schizophrenia or schizoaffective disorder. Schizophrenia Research. 2008; **106**(2-3): 258-64.
3355. Mendelson TC, Shaw KL. Use of AFLP markers in surveys of arthropod diversity. Methods In Enzymology. 2005; **395**: 161-77.
3356. Mendez-David I, Hen R, Gardier AM, David DJ. Adult hippocampal neurogenesis: an actor in the antidepressant-like action. Annales Pharmaceutiques Francaises. 2013; **71**(3): 143-9.
3357. Mignini LE, Latthe PM, Villar J, Kilby MD, Carroli G, Khan KS. Mapping the theories of preeclampsia: the role of homocysteine. Obstetrics And Gynecology. 2005; **105**(2): 411-25.
3358. Mijajlović MD, Pavlović A, Brainin M, Heiss W-D, Quinn TJ, Ihle-Hansen HB, et al. Post-stroke dementia - a comprehensive review. BMC Medicine. 2017; **15**(1): 11-.
3359. Millan MJ. Dual- and triple-acting agents for treating core and co-morbid symptoms of major depression: novel concepts, new drugs. Neurotherapeutics: The Journal Of The American Society For Experimental Neurotherapeutics. 2009; **6**(1): 53-77.
3360. Mitroi N, Moţa M. Nutrigenomics/Nutrigenetics. Romanian Journal Of Internal Medicine = Revue Roumaine De Medecine Interne. 2008; **46**(4): 295-304.
3361. Modi ME, Young LJ. The oxytocin system in drug discovery for autism: animal models and novel therapeutic strategies. Hormones And Behavior. 2012; **61**(3): 340-50.
3362. Moeller BJ, Pasqualini R, Arap W. Targeting cancer-specific synthetic lethality in double-strand DNA break repair. Cell Cycle (Georgetown, Tex). 2009; **8**(12): 1872-6.
3363. Monteiro-Junior RS, Cevada T, Oliveira BRR, Lattari E, Portugal EMM, Carvalho A, et al. We need to move more: Neurobiological hypotheses of physical exercise as a treatment for Parkinson's disease. Medical Hypotheses. 2015; **85**(5): 537-41.
3364. Montgomery H, Brull D. Gene-environment interactions and the response to exercise. International Journal Of Experimental Pathology. 2000; **81**(5): 283-7.
3365. Mössner R, Freitag CM, Gutknecht L, Reif A, Tauber R, Franke P, et al. The novel brain-specific tryptophan hydroxylase-2 gene in panic disorder. Journal Of Psychopharmacology (Oxford, England). 2006; **20**(4): 547-52.
3366. Murck H. Ketamine, magnesium and major depression--from pharmacology to pathophysiology and back. Journal Of Psychiatric Research. 2013; **47**(7): 955-65.
3367. Nam HW, Bruner RC, Choi D-S. Adenosine signaling in striatal circuits and alcohol use disorders. Molecules And Cells. 2013; **36**(3): 195-202.
3368. Narasimhan S, Lohoff FW. Pharmacogenetics of antidepressant drugs: current clinical practice and future directions. Pharmacogenomics. 2012; **13**(4): 441-64.
3369. Narimatsu H, Nakata Y, Nakamura S, Sato H, Sho R, Otani K, et al. Applying data envelopment analysis to preventive medicine: a novel method for constructing a personalized risk model of obesity. PLoS One. 2015; **10**(5): e0126443-e.
3370. Nesterova G, Gahl WA. Cystinosis. GeneReviews®. 1993.

3371. Neumeister A. Tryptophan depletion, serotonin, and depression: where do we stand? Psychopharmacology Bulletin. 2003; **37**(4): 99-115.
3372. Nin MS, Martinez LA, Pibiri F, Nelson M, Pinna G. Neurosteroids reduce social isolation-induced behavioral deficits: a proposed link with neurosteroid-mediated upregulation of BDNF expression. Front Endocrinol (Lausanne). 2011; **2**: 73.
3373. Nogales-Gadea G, Godfrey R, Santalla A, Coll-Cantí J, Pintos-Morell G, Pinós T, et al. Genes and exercise intolerance: insights from McArdle disease. Physiological Genomics. 2016; **48**(2): 93-100.
3374. Okafor MT, Nwagha TU, Anusiem C, Okoli UA, Nubila NI, Al-Alloosh F, et al. Cancer prevention, the need to preserve the integrity of the genome at all cost. Nigerian Journal Of Clinical Practice. 2018; **21**(5): 539-45.
3375. O'Keefe JH, Vogel R, Lavie CJ, Cordain L. Achieving hunter-gatherer fitness in the 21(st) century: back to the future. The American Journal Of Medicine. 2010; **123**(12): 1082-6.
3376. O'Keefe JH, Vogel R, Lavie CJ, Cordain L. Organic fitness: physical activity consistent with our hunter-gatherer heritage. The Physician And Sportsmedicine. 2010; **38**(4): 11-8.
3377. Oliveira de Carvalho A, Filho ASS, Murillo-Rodriguez E, Rocha NB, Carta MG, Machado S. Physical Exercise For Parkinson's Disease: Clinical And Experimental Evidence. Clinical Practice And Epidemiology In Mental Health: CP & EMH. 2018; **14**: 89-98.
3378. Onder G, Della Vedova C, Landi F. Validated treatments and therapeutics prospectives regarding pharmacological products for sarcopenia. The Journal Of Nutrition, Health & Aging. 2009; **13**(8): 746-56.
3379. Onder G, Vedova CD, Pahor M. Effects of ACE inhibitors on skeletal muscle. Current Pharmaceutical Design. 2006; **12**(16): 2057-64.
3380. O'Neill J, McMahon SB, Undem BJ. Chronic cough and pain: Janus faces in sensory neurobiology? Pulmonary Pharmacology & Therapeutics. 2013; **26**(5): 476-85.
3381. Orosz A, Federspiel A, Haisch S, Seeher C, Dierks T, Cattapan K. A biological perspective on differences and similarities between burnout and depression. Neuroscience And Biobehavioral Reviews. 2017; **73**: 112-22.
3382. Owen DR, Rupprecht R, Nutt DJ. Stratified medicine in psychiatry: a worrying example or new opportunity in the treatment of anxiety? Journal Of Psychopharmacology (Oxford, England). 2013; **27**(2): 119-22.
3383. Oze I, Matsuo K, Wakai K, Nagata C, Mizoue T, Tanaka K, et al. Alcohol drinking and esophageal cancer risk: an evaluation based on a systematic review of epidemiologic evidence among the Japanese population. Japanese Journal Of Clinical Oncology. 2011; **41**(5): 677-92.
3384. Pabalan N, Trevisan CM, Peluso C, Jarjanazi H, Christofolini DM, Barbosa CP, et al. Evaluating influence of the genotypes in the follicle-stimulating hormone receptor (FSHR) Ser680Asn (rs6166) polymorphism on poor and hyper-responders to ovarian stimulation: a meta-analysis. Journal Of Ovarian Research. 2014; **7**: 285-.
3385. Palotie A, Ripatti S. Finland establishing the internet of genomics and health data. Duodecim; Laaketieteellinen Aikakauskirja. 2017; **133**(8): 771-5.
3386. Partonen T. Clock genes in human alcohol abuse and comorbid conditions.

Alcohol (Fayetteville, NY). 2015; **49**(4): 359-65.
3387. Peeri M, Amiri S. Protective effects of exercise in metabolic disorders are mediated by inhibition of mitochondrial-derived sterile inflammation. Medical Hypotheses. 2015; **85**(6): 707-9.
3388. Peeters MW, Thomis MAI, Beunen GP, Malina RM. Genetics and sports: an overview of the pre-molecular biology era. Medicine And Sport Science. 2009; **54**: 28-42.
3389. Penn L, Boeing H, Boushey CJ, Dragsted LO, Kaput J, Scalbert A, et al. Assessment of dietary intake: NuGO symposium report. Genes & Nutrition. 2010; **5**(3): 205-13.
3390. Perfect JR. Fungal diagnosis: how do we do it and can we do better? Current Medical Research And Opinion. 2013; **29 Suppl 4**: 3-11.
3391. Pergamin-Hight L, Bakermans-Kranenburg MJ, van Ijzendoorn MH, Bar-Haim Y. Variations in the promoter region of the serotonin transporter gene and biased attention for emotional information: a meta-analysis. Biological Psychiatry. 2012; **71**(4): 373-9.
3392. Phillips C. Brain-Derived Neurotrophic Factor, Depression, and Physical Activity: Making the Neuroplastic Connection. Neural Plasticity. 2017; **2017**: 7260130-.
3393. Pickering C, Kiely J. Are the Current Guidelines on Caffeine Use in Sport Optimal for Everyone? Inter-individual Variation in Caffeine Ergogenicity, and a Move Towards Personalised Sports Nutrition. Sports Medicine (Auckland, NZ). 2018; **48**(1): 7-16.
3394. Pike LJ, Sadler JE. Proteomics, genomics and the future of medical education. Missouri Medicine. 2004; **101**(5): 496-9.
3395. Pizza F, Magnani M, Indrio C, Plazzi G. The hypocretin system and psychiatric disorders. Current Psychiatry Reports. 2014; **16**(2): 433-.
3396. Pomeroy J, Soderberg AM, Franks PW. Gene-lifestyle interactions and their consequences on human health. Medicine And Sport Science. 2009; **54**: 110-35.
3397. Potter JD. Colorectal cancer: molecules and populations. Journal Of The National Cancer Institute. 1999; **91**(11): 916-32.
3398. Privitera AP, Distefano R, Wefer HA, Ferro A, Pulvirenti A, Giugno R. OCDB: a database collecting genes, miRNAs and drugs for obsessive-compulsive disorder. Database: The Journal Of Biological Databases And Curation. 2015; **2015**: bav069-bav.
3399. Prokopec SD, Houlahan KE, Sun RX, Watson JD, Yao CQ, Lee J, et al. Compendium of TCDD-mediated transcriptomic response datasets in mammalian model systems. BMC Genomics. 2017; **18**(1): 78-.
3400. Przeliorz-Pyszczek A, Regulska-Ilow B. The role of macronutrient intake in reducing the risk of obesity and overweight among carriers of different polymorphisms of FTO gene. A review. Roczniki Panstwowego Zakladu Higieny. 2017; **68**(1): 5-13.
3401. Qiu M, Xu L, Yang X, Ding X, Hu J, Jiang F, et al. XRCC3 Thr241Met is associated with response to platinum-based chemotherapy but not survival in advanced non-small cell lung cancer. PLoS One. 2013; **8**(10): e77005-e.
3402. Qixing M, Gaochao D, Wenjie X, Rong Y, Feng J, Lin X, et al. Predictive Value of Ercc1 and Xpd Polymorphisms for Clinical Outcomes of Patients Receiving Neoadjuvant Therapy: A Prisma-Compliant Meta-Analysis. Medicine. 2015; **94**(39): e1593-e.
3403. Ramamurthy M, Sankar S, Kannangai R, Nandagopal B, Sridharan G.

Application of viromics: a new approach to the understanding of viral infections in humans. Virusdisease. 2017; **28**(4): 349-59.
3404. Rathnayake D, Sinclair R. Male androgenetic alopecia. Expert Opinion On Pharmacotherapy. 2010; **11**(8): 1295-304.
3405. Razquin C, Marti A, Martinez JA. Evidences on three relevant obesogenes: MC4R, FTO and PPARgamma. Approaches for personalized nutrition. Mol Nutr Food Res. 2011; **55**(1): 136-49.
3406. Razquin C, Marti A, Martinez JA. Evidences on three relevant obesogenes: MC4R, FTO and PPARγ. Approaches for personalized nutrition. Molecular Nutrition & Food Research. 2011; **55**(1): 136-49.
3407. Reif A, Weber H, Domschke K, Klauke B, Baumann C, Jacob CP, et al. Meta-analysis argues for a female-specific role of MAOA-uVNTR in panic disorder in four European populations. American Journal Of Medical Genetics Part B, Neuropsychiatric Genetics: The Official Publication Of The International Society Of Psychiatric Genetics. 2012; **159B**(7): 786-93.
3408. Ren Z-Y, Xu X-Q, Bao Y-P, He J, Shi L, Deng J-H, et al. The impact of genetic variation on sensitivity to opioid analgesics in patients with postoperative pain: a systematic review and meta-analysis. Pain Physician. 2015; **18**(2): 131-52.
3409. Rida PCG, Cantuaria G, Reid MD, Kucuk O, Aneja R. How to be good at being bad: centrosome amplification and mitotic propensity drive intratumoral heterogeneity. Cancer Metastasis Reviews. 2015; **34**(4): 703-13.
3410. Robertson HR, Feng G. Annual Research Review: Transgenic mouse models of childhood-onset psychiatric disorders. Journal Of Child Psychology And Psychiatry, And Allied Disciplines. 2011; **52**(4): 442-75.
3411. Robson B, Mushlin R. Clinical and pharmacogenomic data mining: 2. A simple method for the combination of information from associations and multivariances to facilitate analysis, decision, and design in clinical research and practice. Journal Of Proteome Research. 2004; **3**(4): 697-711.
3412. Roth SM. Genetic aspects of skeletal muscle strength and mass with relevance to sarcopenia. Bonekey Reports. 2012; **1**: 58-.
3413. Rust P, Ekmekcioglu C. Impact of Salt Intake on the Pathogenesis and Treatment of Hypertension. Advances In Experimental Medicine And Biology. 2017; **956**: 61-84.
3414. Ryan J, Ancelin M-L. Polymorphisms of estrogen receptors and risk of depression: therapeutic implications. Drugs. 2012; **72**(13): 1725-38.
3415. S P, S A, H A, M JD, Foods A, Pharmaceuticals B. Literature Abstracts. Journal Of Texture Studies. 1971; **2**(2): 249-56.
3416. Saddler CA, Wu Y, Valckenborgh F, Tanaka MM. Epidemiological control of drug resistance and compensatory mutation under resistance testing and second-line therapy. Epidemics. 2013; **5**(4): 164-73.
3417. Saito M. [Daily practice using the guidelines for prevention and treatment of osteoporosis. How do we realize the bone quality in routine practice using Japanese guideline for osteoporosis prevention and treatment?]. Clinical Calcium. 2008; **18**(8): 1104-13.
3418. Sanderson S, Emery J, Higgins J. CYP2C9 gene variants, drug dose, and bleeding risk in warfarin-treated patients: a HuGEnet systematic review and meta-

analysis. Genetics In Medicine: Official Journal Of The American College Of Medical Genetics. 2005; **7**(2): 97-104.
3419. Sankaranarayanan K, Chakraborty R. Impact of cancer predisposition and radiosensitivity on the population risk of radiation-induced cancers. Radiation Research. 2001; **156**(5 Pt 2): 648-56.
3420. Sasi M, Vignoli B, Canossa M, Blum R. Neurobiology of local and intercellular BDNF signaling. Pflugers Archiv: European Journal Of Physiology. 2017; **469**(5-6): 593-610.
3421. Savage KM, Stough CK, Byrne GJ, Scholey A, Bousman C, Murphy J, et al. Kava for the treatment of generalised anxiety disorder (K-GAD): study protocol for a randomised controlled trial. Trials. 2015; **16**: 493-.
3422. Schäfer A, Scheurlen M, Kraus MR. [Managing psychiatric side effects of antiviral therapy in chronic hepatitis C]. Zeitschrift Fur Gastroenterologie. 2012; **50**(10): 1108-13.
3423. Schneider BP, Shen F, Miller KD. Pharmacogenetic biomarkers for the prediction of response to antiangiogenic treatment. The Lancet Oncology. 2012; **13**(10): e427-e36.
3424. Schoeller DA. The importance of clinical research: the role of thermogenesis in human obesity. The American Journal Of Clinical Nutrition. 2001; **73**(3): 511-6.
3425. Schuit F. Comparative genomics: beyond the horizon of the next research grant. Diabetologia. 2015; **58**(8): 1720-4.
3426. Shaltiel G, Chen G, Manji HK. Neurotrophic signaling cascades in the pathophysiology and treatment of bipolar disorder. Current Opinion In Pharmacology. 2007; **7**(1): 22-6.
3427. Shchelochkov OA, Carrillo N, Venditti C. Propionic Acidemia. GeneReviews®. 1993.
3428. Shen H-Y, Chen J-F. Adenosine A(2A) receptors in psychopharmacology: modulators of behavior, mood and cognition. Current Neuropharmacology. 2009; **7**(3): 195-206.
3429. Shimada T, Fujita N, Maeda M, Ishihama A. Systematic search for the Cra-binding promoters using genomic SELEX system. Genes To Cells: Devoted To Molecular & Cellular Mechanisms. 2005; **10**(9): 907-18.
3430. Shui I, Giovannucci E. Vitamin D status and cancer incidence and mortality. Advances In Experimental Medicine And Biology. 2014; **810**: 33-51.
3431. Simchovitz A, Heneka MT, Soreq H. Personalized genetics of the cholinergic blockade of neuroinflammation. Journal Of Neurochemistry. 2017; **142 Suppl 2**: 178-87.
3432. Skelton K, Ressler KJ, Norrholm SD, Jovanovic T, Bradley-Davino B. PTSD and gene variants: new pathways and new thinking. Neuropharmacology. 2012; **62**(2): 628-37.
3433. Skelton MR, Williams MT, Vorhees CV. Developmental effects of 3,4-methylenedioxymethamphetamine: a review. Behavioural Pharmacology. 2008; **19**(2): 91-111.
3434. Small DM. Individual differences in the neurophysiology of reward and the obesity epidemic. International Journal Of Obesity (2005). 2009; **33 Suppl 2**: S44-S8.
3435. Smith JP, Prince MA, Achua JK, Robertson JM, Anderson RT, Ronan PJ, et al. Intensity of anxiety is modified via complex integrative stress circuitries. Psychoneuroendocrinology. 2016; **63**: 351-61.

3436. Smith T, Sharp S, Manzardo AM, Butler MG. Pharmacogenetics informed decision making in adolescent psychiatric treatment: a clinical case report. International Journal Of Molecular Sciences. 2015; **16**(3): 4416-28.
3437. Smoller JW, Block SR, Young MM. Genetics of anxiety disorders: the complex road from DSM to DNA. Depression And Anxiety. 2009; **26**(11): 965-75.
3438. Sobanski T, Wagner G. Functional neuroanatomy in panic disorder: Status quo of the research. World Journal Of Psychiatry. 2017; **7**(1): 12-33.
3439. Sodhi MSK, Sanders-Bush E. Serotonin and brain development. International Review Of Neurobiology. 2004; **59**: 111-74.
3440. Solomon MB, Herman JP. Sex differences in psychopathology: of gonads, adrenals and mental illness. Physiology & Behavior. 2009; **97**(2): 250-8.
3441. Sontag MK, Accurso FJ. Gene modifiers in pediatrics: application to cystic fibrosis. Advances In Pediatrics. 2004; **51**: 5-36.
3442. Sproule BA, Naranjo CA, Brenmer KE, Hassan PC. Selective serotonin reuptake inhibitors and CNS drug interactions. A critical review of the evidence. Clinical Pharmacokinetics. 1997; **33**(6): 454-71.
3443. Srivastava S, Reid BJ, Ghosh S, Kramer BS. Research Needs for Understanding the Biology of Overdiagnosis in Cancer Screening. Journal Of Cellular Physiology. 2016; **231**(9): 1870-5.
3444. St Jeor ST, Brownell KD, Atkinson RL, Bouchard C, Dwyer J, Foreyt JP, et al. Obesity. Workshop III. AHA Prevention Conference III. Behavior change and compliance: keys to improving cardiovascular health. Circulation. 1993; **88**(3): 1391-6.
3445. Stein DJ. Advances in the neurobiology of obsessive-compulsive disorder. Implications for conceptualizing putative obsessive-compulsive and spectrum disorders. The Psychiatric Clinics Of North America. 2000; **23**(3): 545-62.
3446. Stein J. The current status of the magnocellular theory of developmental dyslexia. Neuropsychologia. 2018.
3447. Stinchcombe JR, Agrawal AF, Hohenlohe PA, Arnold SJ, Blows MW. Estimating nonlinear selection gradients using quadratic regression coefficients: double or nothing? Evolution; International Journal Of Organic Evolution. 2008; **62**(9): 2435-40.
3448. Suh Y, Vijg J. SNP discovery in associating genetic variation with human disease phenotypes. Mutation Research. 2005; **573**(1-2): 41-53.
3449. Suk K-T, Kim H-S. [Biomarkers for colorectal cancer treatment]. The Korean Journal Of Gastroenterology = Taehan Sohwagi Hakhoe Chi. 2009; **53**(2): 68-75.
3450. Sun X, Lai R, Li J, Luo M, Wang Y, Sheng W. The -7351C/T polymorphism in the TPA gene and ischemic stroke risk: a meta-analysis. PLoS One. 2013; **8**(1): e53558-e.
3451. Sun X, Patnode CD, Williams C, Senger CA, Kapka TJ, Whitlock EP. Interventions to Improve Patient Adherence to Hepatitis C Treatment: Comparative Effectiveness. 2012.
3452. Tanev KS, Pentel KZ, Kredlow MA, Charney ME. PTSD and TBI co-morbidity: scope, clinical presentation and treatment options. Brain Injury. 2014; **28**(3): 261-70.
3453. Tang Z, Chen Z, Tang B, Jiang H. Primary erythromelalgia: a review. Orphanet Journal Of Rare Diseases. 2015; **10**: 127-.
3454. Terrault N, Monto A, Stinchon MR, Rusie E, Moreo K. New Therapies, Evidence, and Guidance in Hepatitis C Management: Expert Practices and Insights from an

Educational Symposium at the AMCP 27th Annual Meeting Expo. Journal Of Managed Care & Specialty Pharmacy. 2015; **21**(9): S1-S14.
3455. Thoeringer CK, Ripke S, Unschuld PG, Lucae S, Ising M, Bettecken T, et al. The GABA transporter 1 (SLC6A1): a novel candidate gene for anxiety disorders. Journal Of Neural Transmission (Vienna, Austria: 1996). 2009; **116**(6): 649-57.
3456. Thomas DR, Hagan JJ. 5-HT7 receptors. Current Drug Targets CNS And Neurological Disorders. 2004; **3**(1): 81-90.
3457. Thomas PPM, Alshehri SM, van Kranen HJ, Ambrosino E. The impact of personalized medicine of Type 2 diabetes mellitus in the global health context. Personalized Medicine. 2016; **13**(4): 381-93.
3458. Tucker R, Collins M. What makes champions? A review of the relative contribution of genes and training to sporting success. British Journal Of Sports Medicine. 2012; **46**(8): 555-61.
3459. Ucińska R, Damps-Konstańska I, Siemińska A, Jassem E. [Chronic obstructive pulmonary disease in women]. Pneumonologia I Alergologia Polska. 2012; **80**(2): 140-5.
3460. Uher R. Genes, environment, and individual differences in responding to treatment for depression. Harvard Review Of Psychiatry. 2011; **19**(3): 109-24.
3461. Uhlig K, Earley A, Lamont J, Dahabreh IJ, Avendano EE, Cowan JM, et al. Fluorescence In Situ Hybridization (FISH) or Other In Situ Hybridization (ISH) Testing of Uterine Cervical Cells to Predict Precancer and Cancer. 2013.
3462. Uleckiene S, Didziapetriene J, Griciūte LL, Urbeliene J, Kasiulevicius V, Sapoka V. [Risk factors of main cancer sites]. Medicina (Kaunas, Lithuania). 2008; **44**(12): 989-95.
3463. Van Damme P, Laleman W, Stärkel P, Van Vlierberghe H, Vandijck D, Hindman SJ, et al. Hepatitis C epidemiology in Belgium. Acta Gastro-Enterologica Belgica. 2014; **77**(2): 277-9.
3464. van de Vondervoort I, Poelmans G, Aschrafi A, Pauls DL, Buitelaar JK, Glennon JC, et al. An integrated molecular landscape implicates the regulation of dendritic spine formation through insulin-related signalling in obsessive-compulsive disorder. Journal Of Psychiatry & Neuroscience: JPN. 2016; **41**(4): 280-5.
3465. Van Loo P, Voet T. Single cell analysis of cancer genomes. Current Opinion In Genetics & Development. 2014; **24**: 82-91.
3466. VanItallie TB. Resistance to weight gain during overfeeding: a NEAT explanation. Nutrition Reviews. 2001; **59**(2): 48-51.
3467. Vaughan S, Morris N, Shum D, O'Dwyer S, Polit D. Study protocol: a randomised controlled trial of the effects of a multi-modal exercise program on cognition and physical functioning in older women. BMC Geriatrics. 2012; **12**: 60-.
3468. Verma M, Khoury MJ, Ioannidis JPA. Opportunities and challenges for selected emerging technologies in cancer epidemiology: mitochondrial, epigenomic, metabolomic, and telomerase profiling. Cancer Epidemiology, Biomarkers & Prevention: A Publication Of The American Association For Cancer Research, Cosponsored By The American Society Of Preventive Oncology. 2013; **22**(2): 189-200.
3469. Vincent G. [Silvicultural treatments and their selection effects]. TAG Theoretical And Applied Genetics Theoretische Und Angewandte Genetik. 1973; **43**(8): 364-73.
3470. Volders P, Van Hove J, Lories RJU, Vandekerckhove P, Matthijs G, De Vos R, et

al. Niemann-Pick disease type B: an unusual clinical presentation with multiple vertebral fractures. American Journal Of Medical Genetics. 2002; **109**(1): 42-51.
3471. Völker K. [Physical activity, hypertension, and cardiac and circulatory system]. Medizinische Klinik (Munich, Germany: 1983). 2006; **101 Suppl 1**: 61-5.
3472. Vorderstrasse AA, Ginsburg GS, Kraus WE, Maldonado MCJ, Wolever RQ. Health coaching and genomics-potential avenues to elicit behavior change in those at risk for chronic disease: protocol for personalized medicine effectiveness study in air force primary care. Global Advances In Health And Medicine. 2013; **2**(3): 26-38.
3473. Wang E, Zaman N, McGee S, Milanese J-S, Masoudi-Nejad A, O'Connor-McCourt M. Predictive genomics: a cancer hallmark network framework for predicting tumor clinical phenotypes using genome sequencing data. Seminars In Cancer Biology. 2015; **30**: 4-12.
3474. Wang Q, Xia J, Jia P, Pao W, Zhao Z. Application of next generation sequencing to human gene fusion detection: computational tools, features and perspectives. Briefings In Bioinformatics. 2013; **14**(4): 506-19.
3475. Wang X, Oldani MJ, Zhao X, Huang X, Qian D. A review of cancer risk prediction models with genetic variants. Cancer Informatics. 2014; **13**(Suppl 2): 19-28.
3476. Ware JJ, van den Bree MBM, Munafò MR. Association of the CHRNA5-A3-B4 gene cluster with heaviness of smoking: a meta-analysis. Nicotine & Tobacco Research: Official Journal Of The Society For Research On Nicotine And Tobacco. 2011; **13**(12): 1167-75.
3477. Weber WW, Hein DW. N-acetylation pharmacogenetics. Pharmacological Reviews. 1985; **37**(1): 25-79.
3478. Weiner L, Brissette JL, Model P. Stress-induced expression of the Escherichia coli phage shock protein operon is dependent on sigma 54 and modulated by positive and negative feedback mechanisms. Genes & Development. 1991; **5**(10): 1912-23.
3479. Weishaupt N, Blesch A, Fouad K. BDNF: the career of a multifaceted neurotrophin in spinal cord injury. Experimental Neurology. 2012; **238**(2): 254-64.
3480. Weitz JI, Linkins L-A. Beyond heparin and warfarin: the new generation of anticoagulants. Expert Opinion On Investigational Drugs. 2007; **16**(3): 271-82.
3481. Wermter A-K, Laucht M, Schimmelmann BG, Banaschewski T, Sonuga-Barke EJS, Rietschel M, et al. From nature versus nurture, via nature and nurture, to gene x environment interaction in mental disorders. European Child & Adolescent Psychiatry. 2010; **19**(3): 199-210.
3482. Wiersinga WM. Paradigm shifts in thyroid hormone replacement therapies for hypothyroidism. Nature Reviews Endocrinology. 2014; **10**(3): 164-74.
3483. Wilber RL, Pitsiladis YP. Kenyan and Ethiopian distance runners: what makes them so good? International Journal Of Sports Physiology And Performance. 2012; **7**(2): 92-102.
3484. Williams CJ, Williams MG, Eynon N, Ashton KJ, Little JP, Wisloff U, et al. Genes to predict VO2max trainability: a systematic review. BMC Genomics. 2017; **18**(Suppl 8): 831-.
3485. Wilson CJ, de la Haye K, Coveney J, Hughes DL, Hutchinson A, Miller C, et al. Protocol for a randomized controlled trial testing the impact of feedback on familial risk of chronic diseases on family-level intentions to participate in preventive lifestyle

behaviors. BMC Public Health. 2016; **16**: 965-.
3486. Wilson JR, Patel AA, Brodt ED, Dettori JR, Brodke DS, Fehlings MG. Genetics and heritability of cervical spondylotic myelopathy and ossification of the posterior longitudinal ligament: results of a systematic review. Spine. 2013; **38**(22 Suppl 1): S123-S46.
3487. Winickoff DE. Partnership in U.K. Biobank: a third way for genomic property? The Journal Of Law, Medicine & Ethics: A Journal Of The American Society Of Law, Medicine & Ethics. 2007; **35**(3): 440-56.
3488. Winkler S, Picó C, Ahrens W. [Physiological mechanisms in the development of adiposity]. Bundesgesundheitsblatt, Gesundheitsforschung, Gesundheitsschutz. 2010; **53**(7): 681-9.
3489. Wlodek D, Gonzales M. Decreased energy levels can cause and sustain obesity. Journal Of Theoretical Biology. 2003; **225**(1): 33-44.
3490. Woody OZ, Nadon R. The Shivplot: a graphical display for trend elucidation and exploratory analysis of microarray data. Source Code For Biology And Medicine. 2006; **1**: 6-.
3491. Wright RO, Christiani D. Gene-environment interaction and children's health and development. Current Opinion In Pediatrics. 2010; **22**(2): 197-201.
3492. Wu FL, Wang LY, Huang YQ, Guo WB, Liu CD, Li SG. Interleukin-1β +3954 polymorphisms and risk of external apical root resorption in orthodontic treatment: a meta-analysis. Genetics And Molecular Research: GMR. 2013; **12**(4): 4678-86.
3493. Xiang T, Kang X, Gong Z, Bai W, Chen C, Zhang W. XPG genetic polymorphisms and clinical outcome of patients with advanced non-small cell lung cancer under platinum-based treatment: a meta-analysis of 12 studies. Cancer Chemotherapy And Pharmacology. 2017; **79**(4): 791-800.
3494. Xie W, Meng X, Zhai Y, Zhou P, Ye T, Wang Z, et al. Panax Notoginseng Saponins: A Review of Its Mechanisms of Antidepressant or Anxiolytic Effects and Network Analysis on Phytochemistry and Pharmacology. Molecules (Basel, Switzerland). 2018; **23**(4).
3495. Xu MY, Umbach DM, Murphy E, McMahon F, Shugart YY. A Novel Mixture Model to Estimate the Time to Drug Effect Onset and Its Association with Covariates. Human Heredity. 2015; **80**(2): 90-9.
3496. Yamada K, Kobayashi M, Kanda T. Involvement of adenosine A2A receptors in depression and anxiety. International Review Of Neurobiology. 2014; **119**: 373-93.
3497. Yang A, Childs E, Palmer AA, de Wit H. More on ADORA. Psychopharmacology. 2010; **212**(4): 699-700.
3498. Yang Y, Xian L. The association between the GSTP1 A313G and GSTM1 null/present polymorphisms and the treatment response of the platinum-based chemotherapy in non-small cell lung cancer (NSCLC) patients: a meta-analysis. Tumour Biology: The Journal Of The International Society For Oncodevelopmental Biology And Medicine. 2014; **35**(7): 6791-9.
3499. Yilmaz Y, Younossi ZM. Obesity-associated nonalcoholic fatty liver disease. Clinics In Liver Disease. 2014; **18**(1): 19-31.
3500. Yu D, Yang L, Shen S, Fan C, Zhang W, Mo X. Association between methionine synthase reductase A66G polymorphism and the risk of congenital heart defects:

evidence from eight case-control studies. Pediatric Cardiology. 2014; **35**(7): 1091-8.
3501. Zaïr ZM, Singer DR. Efflux transporter variants as predictors of drug toxicity in lung cancer patients: systematic review and meta-analysis. Pharmacogenomics. 2016; **17**(9): 1089-112.
3502. Zamora Navarro S, Pérez-Llamas F. Errors and myths in feeding and nutrition: impact on the problems of obesity. Nutricion Hospitalaria. 2013; **28 Suppl 5**: 81-8.
3503. Zarbl H. DNA microarrays: an overview of technologies and applications to toxicology. Current Protocols In Toxicology. 2001; **Chapter 1**: Unit1.4.
3504. Zhang R, Wang J, Yang R, Sun J, Chen R, Luo H, et al. Effects of Pro12Ala polymorphism in peroxisome proliferator-activated receptor-gamma2 gene on metabolic syndrome risk: a meta-analysis. Gene. 2014; **535**(1): 79-87.
3505. Zhang R, Wang J, Yang R, Sun J, Chen R, Luo H, et al. Effects of Pro12Ala polymorphism in peroxisome proliferator-activated receptor-γ2 gene on metabolic syndrome risk: a meta-analysis. Gene. 2014; **535**(1): 79-87.
3506. Zhang Y, Pan W. Principal component regression and linear mixed model in association analysis of structured samples: competitors or complements? Genetic Epidemiology. 2015; **39**(3): 149-55.
3507. Zhao J, Yang T-H, Huang Y, Holme P. Ranking candidate disease genes from gene expression and protein interaction: a Katz-centrality based approach. PLoS One. 2011; **6**(9): e24306-e.
3508. Zhao Y, Li X, Kong X. MTHFR C677T Polymorphism is Associated with Tumor Response to Preoperative Chemoradiotherapy: A Result Based on Previous Reports. Medical Science Monitor: International Medical Journal Of Experimental And Clinical Research. 2015; **21**: 3068-76.
3509. Zhou Y, Zhou W, Liu Q, Fan Z, Yang Z, Tu Q, et al. XRCC1 R399Q polymorphism and risk of normal tissue injury after radiotherapy in breast cancer patients. Tumour Biology: The Journal Of The International Society For Oncodevelopmental Biology And Medicine. 2014; **35**(1): 21-5.
3510. Zinck JWR, MacFarlane AJ. Approaches for the identification of genetic modifiers of nutrient dependent phenotypes: examples from folate. Frontiers In Nutrition. 2014; **1**: 8-.
3511. Zong L, Abe M, Ji J, Zhu W-G, Yu D. Tracking the Correlation Between CpG Island Methylator Phenotype and Other Molecular Features and Clinicopathological Features in Human Colorectal Cancers: A Systematic Review and Meta-Analysis. Clinical And Translational Gastroenterology. 2016; **7**: e151-e.
3512. Zwart H. From Utopia to Science: Challenges of Personalised Genomics Information for Health Management and Health Enhancement. Medicine Studies. 2009; **1**(2): 155-66.
