# Supplementary figures and images for: Impact of Genetic Variability on Physiological Responses to Caffeine in Humans: A Systematic Review
Source: Nutrients. 2018 Sep 25;10(10):1373. doi: 10.3390/nu10101373 (PMC6212886; doi:10.3390/nu10101373)

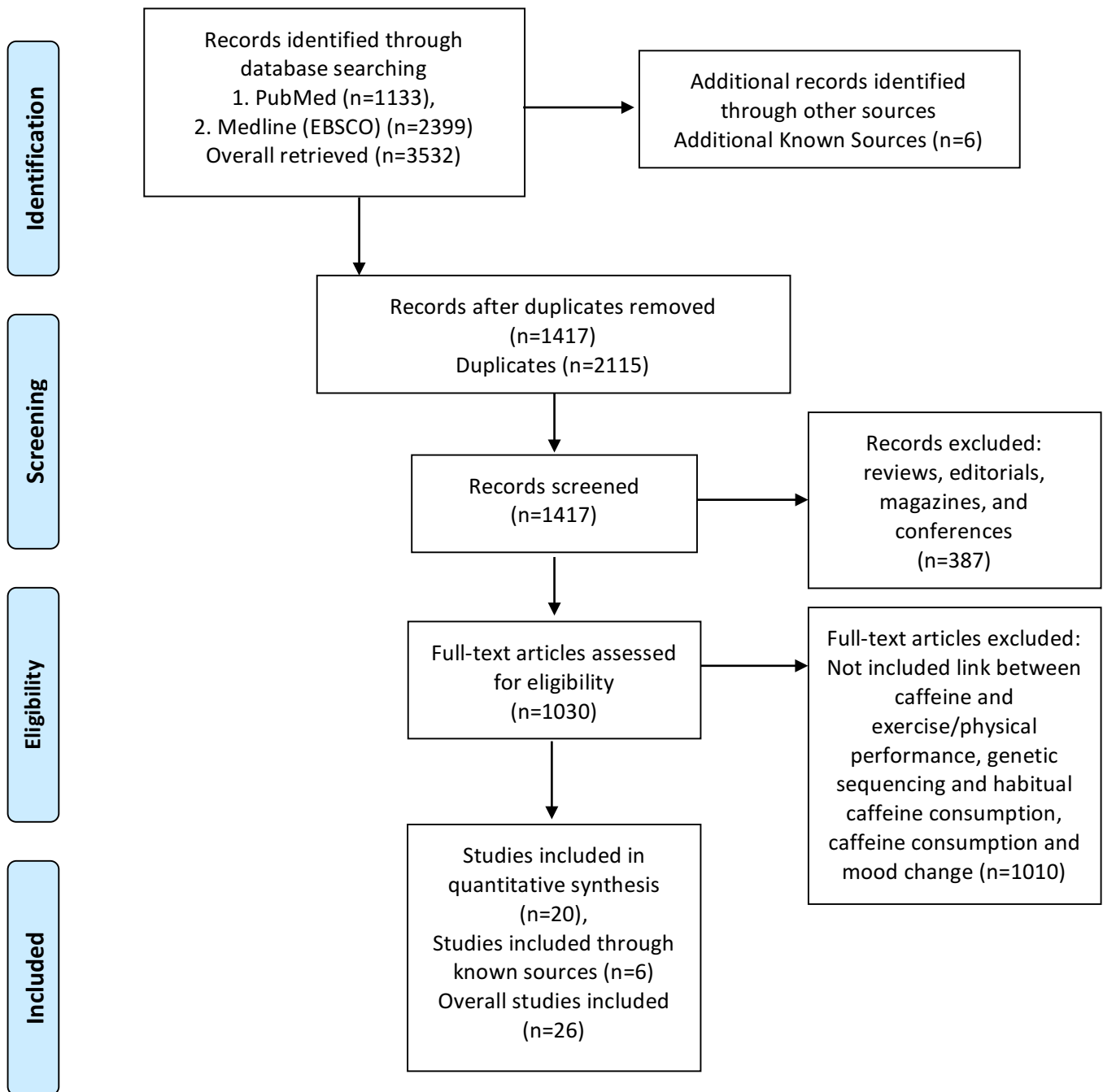

Supplement: Supplementary file 1 [file nutrients-10-01373-s001.zip › FigureS4 PRISMA Flowchart (1).pdf]
